# Ring-opening fluorination of bicyclic azaarenes†

**DOI:** 10.1039/d1sc06273e

**Published:** 2021-12-21

**Authors:** Masaaki Komatsuda, Ayane Suto, Hiroki Kondo, Hiroyuki Takada, Kenta Kato, Bunnai Saito, Junichiro Yamaguchi

**Affiliations:** Department of Applied Chemistry, Waseda University 513, Wasedatsurumakicho, Shinjuku Tokyo 169-8555 Japan junyamaguchi@waseda.jp; Research, Takeda Pharmaceutical Company Limited 26-1, Muraoka-Higashi 2-chome Fujisawa Kanagawa 251-8555 Japan

## Abstract

We have discovered a ring-opening fluorination of bicyclic azaarenes. Upon treatment of bicyclic azaarenes such as pyrazolo[1,5-*a*]pyridines with electrophilic fluorinating agents, fluorination of the aromatic ring is followed by a ring-opening reaction. Although this overall transformation can be classified as an electrophilic fluorination of an aromatic ring, it is a novel type of fluorination that results in construction of tertiary carbon–fluorine bonds. The present protocol can be applied to a range of bicyclic azaarenes, tolerating azines and a variety of functional groups. Additionally, mechanistic studies and enantioselective fluorination have been examined.

## Abstract 2

A ring-opening fluorination of bicyclic azaarenes was developed. Although this overall transformation can be classified as an electrophilic fluorination of aromatics, it is a novel type of fluorination that results in construction of *tert*-C–F bonds.

## Graphical abstract

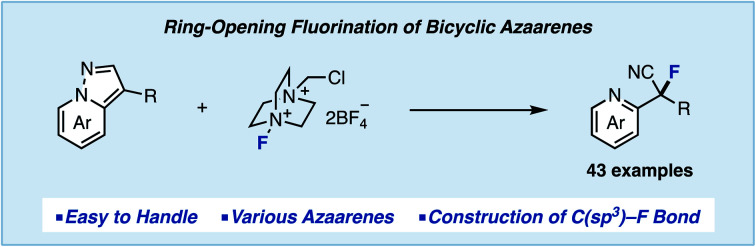

## Introduction

Fluorine is one of the most important elements that could be installed onto hydrocarbon frameworks in pharmaceuticals, agrochemicals, and materials science.^1^ Particularly, in medicinal chemistry, fluorine has been incorporated into drug molecules to improve their liposolubility and metabolic stability.^2^ The effect of fluorine atoms in molecules has been well-studied,^3^ and in turn, fluorination methodology has flourished as well.^4^ One of the most conventional ways to achieve fluorination is electrophilic fluorination. Nucleophiles used in electrophilic fluorinations can be broadly classified into carbanions (*e.g.*, 1,3-dicarbonyls), electron-rich unsaturated bonds (*e.g.*, alkenes and alkynes), and aromatics.^5^ However, in these existing methods, fluorination proceeds while retaining the carbon skeleton of the starting material, and fluorinations involving skeletal transformations are rare.

**Fig. 1:**
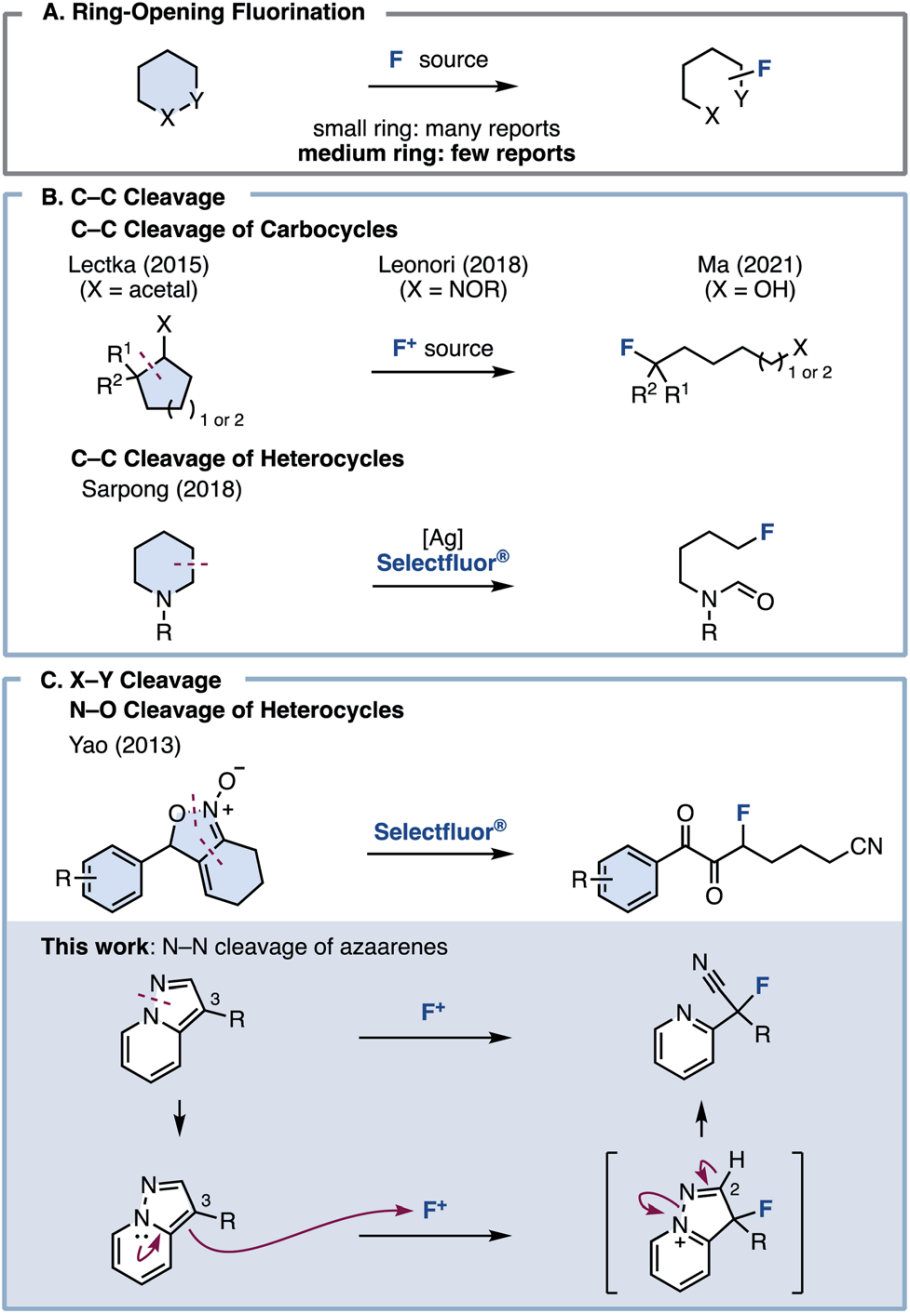
(A) Ring-opening fluorination. (B) Fluorination of cyclic compounds *via* C–C bond cleavage. (C) Fluorination of cyclic compounds *via* X–Y bond cleavage.

Ring-opening fluorination, in which a fluorine atom is introduced onto a cyclic compound with concomitant ring cleavage, has recently attracted attention as a useful method for efficiently constructing complex fluorine-containing skeletons (Fig. 1A). Although ring-opening fluorinations have recently been reported, most are limited to three- or four-membered ring starting materials such as epoxides, cyclopropanes/butanes, and aziridines, which have strained chemical bonds.^6^ Fluorinations involving bond cleavage in a ring size ≥5 are rare. The Lectka, Leonori, and Ma group reported ring-opening fluorinations *via* C–C bond cleavage of carbocycles (Fig. 1B).^7,8^ Very recently, the Lim group disclosed acyl fluoride synthesis through C–C bond cleavage of carbocycles and cyclic amides.^9^ In 2018, the Sarpong group reported an elegant ring-opening fluorination of cyclic amines (Fig. 1B).^9,10^

In the heteroatom–heteroatom (X–Y) bond paradigm, the Yao group reported a ring-opening fluorination using isoxazoline *N*-oxides *via* O–N bond cleavage (Fig. 1C).^11^ However, all these methods require the use of highly specific substrates, and fluorinations involving the ring opening of aromatic rings or asymmetric fluorination have not yet been reported. In contrast to existing methods, we planned to develop a ring-opening fluorination of bicyclic azaarenes such as pyrazolo[1,5-*a*]pyridines. We hypothesized that treating bicyclic azaarenes with an electrophilic fluorinating agent would result in fluorination at the C3 position, followed by deprotonation at the C2 position and pyrazole ring opening *via* N–N bond cleavage. Although this can be considered as a simple electrophilic fluorination using an electron-rich heteroaromatic system as a nucleophile, the resulting compound is an sp^3^-fluorinated compound (C(sp^3^)–F bond) instead of a fluorine-substituted heteroarene (C(sp^2^)–F bond). In other words, we thought that this would be a novel type of fluorination reaction with accompanying skeletal transformation.

## Results and discussion

First, we selected 3-phenylpyrazolo[1,5-*a*]pyridine (1A) as the model substrate (which was readily prepared in three steps from a commercially available compound) to examine electrophilic fluorinating agents and reaction conditions (Table 1). When *N*-fluoropyridinium salts (F1–F3) were used in MeCN at 80 °C, ring-opening fluorinated product 2A was successfully obtained, albeit in low yields (entries 1–3).^12^ The use of other non-pyridinium based electrophilic fluorinating agents such as NFSI and Selectfluor® gave fluorinated products in high yields (entries 4 and 5).^13^

**Table 1:** Screening of reaction conditions^a^

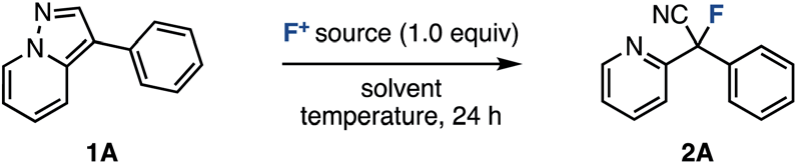					
Entry	F^+^ source	Temp/°C	Solvent	2A/%	1A/%
1	F1	80	MeCN	39	37
2	F2	80	MeCN	27	43
3	F3	80	MeCN	5	3
4	NFSI	80	MeCN	94	0
5	Selectfluor®	80	MeCN	>99	0
6	Selectfluor®	50	MeCN	68	0
7	Selectfluor®	60	MeCN	74	0
8	Selectfluor®	70	MeCN	87	0
9	Selectfluor®	80	Acetone	65	0
10	Selectfluor®	80	DMF	64	0
11	Selectfluor®	80	MeOH	58	0
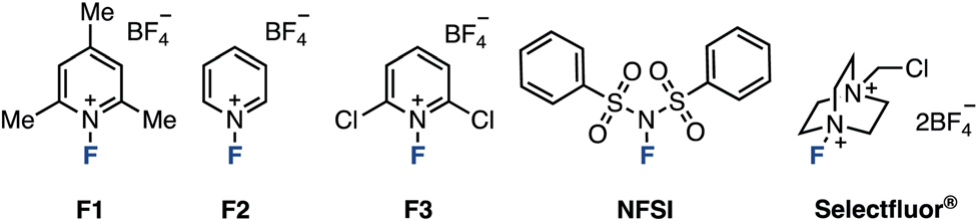					

a Conditions: 1A (0.20 mmol), F^+^ source (1.0 equiv.), solvent (1.0 mL), 50–80 °C, 24 h. NFSI = *N*-fluorobenzenesulfonimide.

**Scheme 1:**
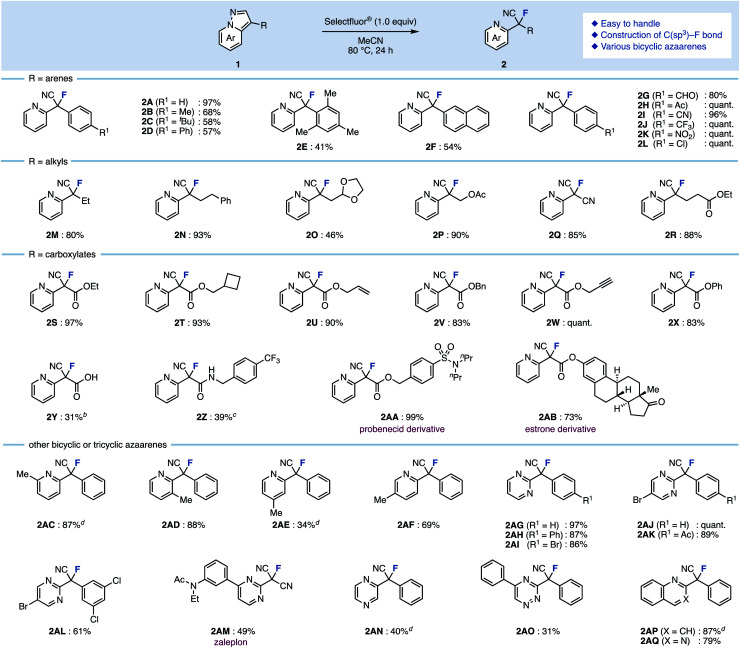
Substrate Scope. ^*a*^ Conditions. 1 (0.20 mmol), Selectfluor® (1.0 equiv.), MeCN (1.0 mL), 80 °C, 24 h. ^*b*^ Selectfluor® (5.0 equiv.) was added. ^*c*^ The reaction was performed at −30 °C. ^*d*^ NaClO_4_ (1.0 equiv.) was added.

As for the reaction temperature, the yield of 2A was 68% even at 50 °C. The yield increased as the temperature was increased, and the fluorinated product was obtained quantitatively at 80 °C (entries 6–8 *vs.* entry 5). The reaction proceeded in polar solvents such as acetone and DMF (which is able to dissolve Selectfluor®), and gave the fluorinated product 2A (entries 9–11). Finally, we conformed the optimal conditions: Selectfluor® (1.0 equiv.) at 80 °C in MeCN for 24 h. With the optimal conditions in hand, the substrate scope was investigated (Scheme 1). Various 3-arylpyrazolo[1,5-*a*]pyridines were examined: Methyl (1B), *tert*-butyl (1C), and phenyl (1D) at the *para*-position on the aryl group gave the ring-opening fluorinated products 2B–2D in moderate yields. It should be noted that fluorination of these aryl groups was detected. When using trimethylphenyl (1E) and naphthyl (1F) starting materials, the corresponding products 2E and 2F were obtained in moderate yields, and occurred decomposition of 1E or fluorinated the aryl group of 1F (less than 10% yields). The reaction showed good functional group tolerance in the presence of formyl (1G), acetyl (1H), cyano (1I), trifluoromethyl (1J), nitro (1K), and chloro (1L) groups, as the reaction worked to give the corresponding products 2G–2L in excellent yields. Next, 3-alkylpyrazolo[1,5-*a*]pyridines were investigated. The fluorination using bicyclic azaarenes bearing alkyl groups (1M and 1N) or acetal (1O) proceeded smoothly to give the corresponding fluorinated products 2M–2O in moderate to excellent yields. Pyrazolo[1,5-*a*]pyridines with alkyl acetate (1P), cyano (1Q), and ethylcarboxylate (1R) afforded the corresponding products (2P–2R) in good yields. Pyrazolo[1,5-*a*]pyridine carboxylates were also examined. Substrates with alkyl groups including alkene (1U) and alkyne (1W) remained intact to give products 2S–2X in high yields. Carboxylic acid 1Y also reacted well, but the product was difficult to purify, resulting in a low yield of 2Y. In the case of compounds with amides such as 1Z, deamidation occurred to give 3-fluoropyrazolo[1,5-*a*]pyridine as a byproduct. Therefore, the fluorinated product 2Z was obtained in moderate yield (39%) by reacting at a lower temperature (−30 °C). Furthermore, azaarenes 1AA and 1AB derived from probenecid and estrone also gave fluorinated compounds 2AA and 2AB in high yields. Of note, in the case of an unsaturated ester or iodine at the C3 position, the desired fluorinated product could not be obtained, giving a complex mixture.

Substituted bicyclic arenes gave fluorination products 2AC–2AF, however, for some substrates such as 1AC and 1AE, the fluorination reactions were more difficult. After extensive screening of additives, we found that NaClO_4_ (1.0 equiv.) was effective for increasing yields (see the ESI† for details). For example, without this additive, 1AC gave 2AC in only 51% yield, but with the additive, the yield improved to 87%. The role of the additive remains unclear, but we hypothesize that the counter anion exchange in the intermediate might affect the acidity of the proton at the C3 position.^14^

This fluorination was also applicable to other azaarenes: pyrazolo[1,5-*a*]pyrimidine with a phenyl group at the C3 position gave fluorinated compounds 2AG–2AI in high yields. 6-Bromopyrazolo[1,5-*a*]pyrimidine with various aryl groups at the C3 position gave fluorinated compounds 2AJ–2AL as well. The ring-opening fluorination proceeded well even when using zaleplon, a hypnotic agent, for which the desired product 2AM was obtained. The reaction was also applicable to pyrazolopyrazine, triazine, quinoline, and quinazoline, giving fluorinated products 2AN–2AQ in moderate yields.

**Fig. 2:**
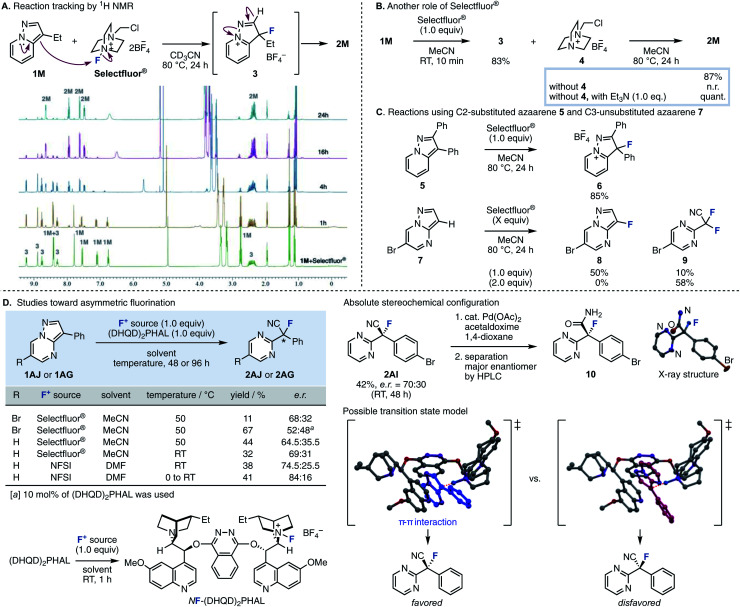
(A) Reaction tracking by ^1^H NMR. (B) The role of Selectfluor®. (C) Reactions using C2-substituted azaarene 5 and C3-unsubstituted azaarene 7. (D) Studies toward asymmetric fluorination.

In order to elucidate the reaction mechanism, we performed reaction tracking by ^1^H NMR analysis using 1M (Fig. 2A). When Selectfluor® was added to 1M in an NMR tube without stirring, 1M was immediately consumed at room temperature to produce tetrafluoroborate 3 as the intermediate, which is thought to be the result of electrophilic fluorination at the C3 position. After 2 to 4 hours of reaction time at 80 °C, 1M almost entirely disappeared, and NMR peaks showed a mixture of 2M and 3; finally, practically only 2M resulted in the ^1^H NMR spectrum. This experiment indicated that the fluorination and the cleavage of the N–N bond proceeds in a stepwise fashion. When the reaction was stirred in a flask, 1M disappeared after 10 min at room temperature, giving intermediate 3 and the residue 4 of Selectfluor® (Fig. 2B). Upon removal of 4 from the resulting mixture, further reaction did not procced by heating at 80 °C for 24 h (see the ESI† for experimental details). Therefore, triethylamine (1.0 equiv.) was added, and the reaction proceeded quickly to give the desired 2M quantitatively. This supports the role of Selectfluor® as the fluorinating agent in the reaction and the conjugate base of 4 as the base that promotes the N–N bond cleavage. These results also suggested that the basicity of the conjugate base of the fluorinating agent was crucial for this ring-opening fluorination. In the case of F1–F3, the consumption of 1A was increased as the electrophilicity of the fluorinating agents was higher (F3 > F2 ≈ F1), but the yield of 2A was proportional to the basicity of the conjugate base (F1 > F2 > F3). Furthermore, the reaction proceeded efficiently with the use of base with higher p*K*_a_ such as triethylamine and 4. Next, the fluorination reaction was carried out with 5, where the C2 position was substituted (Fig. 2C). As a result, only trifluoroborate salt 6 was obtained in a good yield, with no ring-opened product was obtained upon heating. When the fluorination reaction was attempted using 7, which is unsubstituted at the C3 position, one equivalent of Selectfluor® gave the fluorinated compound 8 as the main product (50%) and the ring-opened compound 9 as a byproduct, demonstrating further fluorination. When the amount of Selectfluor® was increased to two equivalents, 9 became the main product (58%). These results indicated that a substituent is required at the C3 position because deprotonation of an acidic C–H (adjacent to F) to regain aromaticity occurs preferentially over deprotonation/ring-opening.

We then studied the enantioselective version of this fluorination reaction (Fig. 2D).^15^ We attempted asymmetric fluorinations using chiral fluorinating agents. Shibata reported that a chiral fluorinating agent, *N*F–(DHQD)_2_PHAL, can be prepared by mixing (DHQD)_2_PHAL and Selectfluor® at room temperature.^16^ A reaction using stoichiometric amounts of these agents with 3-phenyl-6-bromopyrazolo[1,5-*a*]pyrimidine 1AJ in MeCN at 50 °C gave the corresponding product in 68 : 32 *e. r.*, albeit in a low yield. However, when (DHQD)_2_PHAL was reduced to catalytic amount, enantioselectivity was dropped whereas the yield was increased. The substrate without a bromo atom at the C6 position (1AG) gave the fluorinated product in moderate yield (44%) and 65 : 35 *e. r.* By lowering the temperature, changing the fluorinating agent, and changing the solvent, we finally succeeded in obtaining the fluorinated compound 2AJ with an enantioselectivity of 84 : 16 *e. r*.

**Scheme 2:**
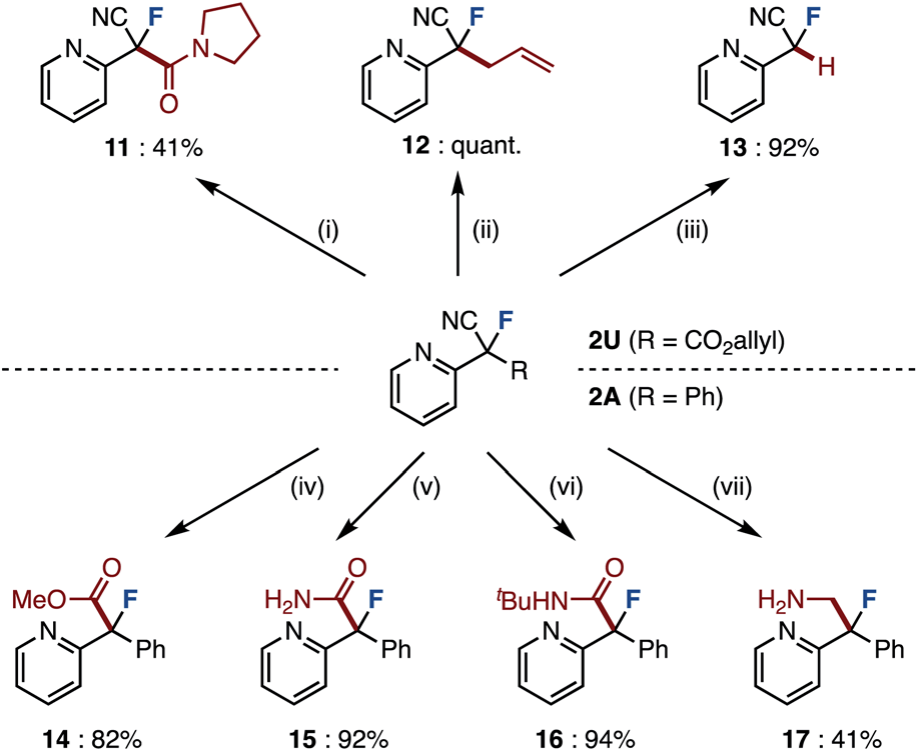
Derivatization of the products. Conditions: (i) pyrrolidine (5.0 equiv.), MeCN, RT, 12 h; (ii) Pd(PPh_3_)_4_ (5.0 mol%), toluene, RT, 1 h; (iii) Pd_2_(dba)_3_ (5.0 mol%), PPh_3_ (20 mol%), pyridine (3.0 equiv.), MeCN, RT, 1 h; (iv) TMSCl (5.0 equiv.), MeOH, 50 °C, 6 h; (v) Pd(OAc)_2_ (4.0 mol%), acetaldoxime (10 equiv.), 1,4-dioxane, reflux, 1 h; (vi) ^*t*^BuOAc (6.0 equiv.), conc. H_2_SO_4_ (10 μL), 40 °C, 2 h; (vii) BH_3_·SMe_2_ (3.0 equiv.), THF, 0 °C to RT, 19 h.

The absolute stereochemical configuration was determined by derivatization to give optically pure amide 10, recrystallization, and then X-ray structural analysis. In the crystal structure, the C=O bond of amide 10 is align antiparallel to the C–F bond, a conformation in which the amide dipole opposes the C–F dipole due to a dipole minimization effect. The enantioselectivity of this asymmetric fluorination could be explained using the proposed transition state model. Although the direction in which the substrate reacts with the chiral fluorinating agent determines the enantioselectivity, we believe that the transition state of the desired compound has a π–π interaction between the substrate and the methoxyquinoline moiety of (DHQD)_2_PHAL, which fixes the conformation.^16*c*,17^ Finally, the obtained fluorinated compounds were derivatized into various compounds (Scheme 2). The ring-opened fluorinated products of pyrazolo[1,5-*a*]pyridine 2U (R = CO_2_allyl) were condense with amines to give amide 11 in 41% yield. Palladium-catalyzed decarboxylative allylation and removal of allyl esters proceeded to give derivatives 12 and 13 in high yields. Furthermore, we attempted to convert the cyano group of the product of the fluorination reaction. Fluorinated product 2A (R = Ph) was converted to methyl ester 14 by methanolysis. 2A was also converted to amides 15 and 16 by hydrolysis and Ritter reaction.^18^ Furthermore, borane reduction gave amine 17. In this way, we have succeeded in synthesizing a variety of fluorine-containing compounds by orthogonal functional group transformations following ring-opening fluorination.

## Conclusions

In summary, we developed a ring-opening fluorination of bicyclic azaarenes leading to sp^3^-fluorinated compounds *via* N–N bond cleavage. Studies revealed that the electrophilic fluorinating reagent functioned not only as the fluorine source, but also as the base required for ring opening. Expanding the range of substrates and other electrophiles for this type of transformation is currently underway in our laboratory.^19^

## Data availability

All experimental data is available in the ESI.†

## Author contributions

B. S. and J. Y. conceived and designed the study. M. K., A. S. and H. K. performed the chemical experiments and analyzed the data. K. K. performed the X-ray crystallography experiments and analyzed the obtained data. H. T. performed the preliminary experimental studies. J. Y. wrote the manuscript and all authors discussed the results and commented on the final manuscript.

## Conflicts of interest

There are no conflicts to declare.

## Supplementary Material

SC-013-D1SC06273E-s001

SC-013-D1SC06273E-s002
