# Supplementary material for: Ring-opening fluorination of bicyclic azaarenes
Source: Chem Sci. 2021 Dec 21;13(3):665–70. doi: 10.1039/d1sc06273e (PMC8768879; doi:10.1039/d1sc06273e)

Electronic Supplementary Material (ESI) for Chemical Science.
This journal is © The Royal Society of Chemistry 2021

Supplementary Information

Ring-Opening Fluorination of Bicyclic Azaarenes

Masaaki Komatsuda,[1] Ayane Suto,[1] Hiroki Kondo Jr.,[1] Hiroyuki Takada,[2] Kenta Kato,[1] Bunnai Saito,[2] and Junichiro Yamaguchi*

[1] *Department of Applied Chemistry, Waseda University, 513 Wasedatsurumakicho, Shinjuku, Tokyo 162-0041, Japan*

[2] *Research, Takeda Pharmaceutical Company Limited, 26-1, Muraoka-Higashi 2-chome, Fujisawa, Kanagawa 251-8555, Japan*

E-mail: junyamaguchi@waseda.jp

Table of Contents

1.	General	S2
2.	Preparation of Pyrazolo[1,5-*a*]pyridines **1**	S3–S15
3.	Preparation of Other Bicyclic or Tricyclic Azaarens **1**	S15–S20
4.	Ring-Opening Fluorination of Bicyclic Azaarenes **1**	S20–S34
5.	Effect of Parameters	S35–S37
6.	Fluorination Tracking by ^{1}H NMR	S38
7.	Another Role of Selectfluor®	S39–S40
8.	Fluorination of **5** and **7**	S40–S41
9.	Studies Toward Asymmetric Fluorination	S42–S55
10.	Derivatization of Products	S55–S58
11.	References	S59–S60
12.	^{1}H, ^{13}C, and ^{19}F NMR Spectra	S60–S283

1. General

Unless otherwise noted, all reactants or reagents including dry solvents were obtained from commercial suppliers and used as received. Selectfluor® and *N*-fluorobenzenesulfonimide (NFSI) were obtained from TCI. $(DHQD)_2$PHAL was obtained from KANTO chemical. 6-Bromopyrazolo[1,5-*a*]pyrimidine (**7**) was obtained from Combi-Blocks. 3-Phenylpyrazolo[1,5-*a*]pyridine (**1A**),[1] 3-(*p*-tolyl)pyrazolo[1,5-*a*]pyridine (**1B**),[1] 3-(4-(*tert*-butyl)phenyl)pyrazolo[1,5-*a*]pyridine (**1C**),[1] 3-mesitylpyrazolo[1,5-*a*]pyridine (**1E**),[1] 3-(naphthalen-2-yl)pyrazolo[1,5-*a*]pyridine (**1F**),[1] 3-(4-(trifluoromethyl)phenyl)pyrazolo[1,5-*a*]pyridine (**1J**),[1] 3-(4-chlorophenyl)pyrazolo[1,5-*a*]pyridine (**1L**),[2] pyrazolo[1,5-*a*]pyridine-3-carbonitrile (**1Q**),[3] ethyl pyrazolo[1,5-*a*]pyridine-3-carboxylate (**1S**),[4] pyrazolo[1,5-*a*]pyridine-3-carboxylic acid (**1Y**),[1] 3-phenylpyrazolo[1,5-*a*]pyrimidine (**1AG**),[5] zaleplon (**1AM**),[6] and 2,3-diphenylpyrazolo[1,5-*a*]pyridine (**5**)[1] were synthesized according to procedures and the spectra matched with those of compounds reported in the literature. Unless otherwise noted, all reactions were performed with dry solvents under an atmosphere of N_2 in dried glassware using standard vacuum-line techniques. All ring-opening fluorinations of azaarenes were performed in an 8-mL glass vessel tube equipped with a screw cap and heated (IKA Plate RCT digital) in a 16-well aluminum reaction block (IKA DB4.3 Block) unless otherwise noted. All work-up and purification procedures were carried out with reagent-grade solvents in air. Analytical thin-layer chromatography (TLC) was performed using Silica-gel 70 TLC Plate-Wako (0.25 mm). The developed chromatogram was analyzed by UV lamp (254, 365 nm). Flash column chromatography was performed with Biotage Isolera® equipped with Biotage Sfär Cartridge Silica D columns and hexane/EtOAc as an eluent. Preparative thin-layer chromatography (PTLC) was performed using Wakogel B5-F silica coated plates (0.75 mm) prepared in our laboratory. Preparative recycling gel permeation chromatography (GPC) was performed with a JAI LaboACE LC-5060 instrument equipped with JAIGEL-2HR columns using chloroform as an eluent. High-resolution mass spectra (HRMS) were conducted on Thermo Fisher Scientific ExactivePlus (ESI and DART). Chiral high performance liquid chromatography (HPLC) was performed using SHIMADZU Prominence-i LC-2030C Plus® equipped with DAICEL Chiralcel® and Chiralpak®. Details of chromatographic conditions on the separation of the products are described with compound data. Nuclear magnetic resonance (NMR) spectra were recorded on JEOL JNM-ECS-400 and JNM-ECZ-400S (^{1}H 400 MHz, ^{13}C 101 MHz, ^{19}F 376 MHz) spectrometers. Chemical shifts for ^{1}H NMR are expressed in parts per million (ppm) relative to tetramethylsilane (δ 0.00 ppm) or acetonitrile-d_3 (δ 1.94 ppm). Chemical shifts for ^{13}C NMR are expressed in ppm relative to $CDCl_3$ (δ 77.0 ppm) or acetonitrile-d_3 (δ 1.32 ppm). Chemical shifts for ^{19}F NMR are expressed in ppm relative to PhF (δ –113.15 ppm). Data are reported as follows: chemical shift, multiplicity (s = singlet, d = doublet, dd = doublet of doublets, ddd = doublet of doublets of doublets, dt = doublet of triplets, t = triplet, td = triplet of doublets, q = quartet, m = multiplet, brs = broad singlet), coupling constant (Hz), and integration.

2. Preparation of Pyrazolo[1,5-*a*]pyridines 1

2-1. Synthesis of 1D and 1G

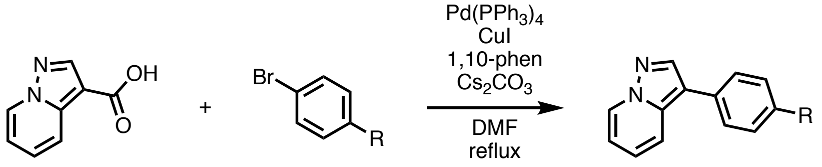

A round-bottom flask containing a magnetic stirring bar and cesium carbonate (244 mg, 0.750 mmol, 1.5 equiv) was dried with a heat-gun *in vacuo* and filled with N_2 gas after cooling to room temperature. To this flask were added $Pd(PPh_3)_4$ (28.9 mg, 25.0 µmol, 5.0 mol%), copper iodide (9.5 mg, 50.0 µmol, 10 mol%), 1,10-phenanthroline (9.5 mg, 50.0 µmol, 10 mol%), and pyrazolo[1,5-*a*]pyridine-3-carboxylic acid (**1L**: 81.1 mg, 0.500 mmol, 1.0 equiv). The flask was placed under vacuum and refilled with N_2 gas three times. To this flask were added aryl bromide (0.750 mmol, 1.5 equiv) and DMF (2.5 mL). The mixture was heated under reflux and stirred for several hours. After cooling the reaction mixture to room temperature, the mixture was passed through a short silica-gel pad with EtOAc as an eluent, and then concentrated *in vacuo*. The residue was purified by Isolera® to afford the corresponding 3-arylpyrazolo[1,5-*a*]pyridine **1**.

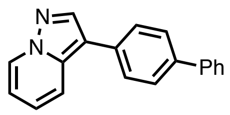

3-([1,1'-Biphenyl]-4-yl)pyrazolo[1,5-*a*]pyridine (1D)

Purification by Isolera® (hexane/EtOAc = 9:1 to 1:1) afforded **1D** (69.8 mg, 52% yield) as a white solid. ^{1}H NMR (400 MHz, $CDCl_3$) δ 8.52 (d, *J* = 7.2 Hz, 1H), 8.20 (s, 1H), 7.88 (d, *J* = 9.2 Hz, 1H), 7.72–7.62 (m, 6H), 7.47 (t, *J* = 7.6 Hz, 2H), 7.37 (t, *J* = 7.6 Hz, 1H), 7.21 (dd, *J* = 9.2, 6.8 Hz, 1H), 6.82 (t, *J* = 7.6 Hz, 1H); ^{13}C NMR (101 MHz, $CDCl_3$) δ 140.7, 140.4, 138.9, 136.9, 132.1, 129.0, 128.8, 127.6, 127.23, 127.18, 126.9, 124.0, 117.5, 112.4, 112.0; HRMS (ESI) *m*/*z* calcd for $C_{19}H_{15}N_2$ $[M+H]^+$: 271.1230 found 271.1227.

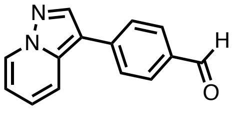

4-(Pyrazolo[1,5-*a*]pyridin-3-yl)benzaldehyde (1G)

Purification by Isolera® (hexane/EtOAc = 9:1 to 1:1) afforded **1G** (61.1 mg, 55% yield) as a yellow solid. ^{1}H NMR (400 MHz, $CDCl_3$) δ 10.0 (s, 1H), 8.54 (d, *J* = 6.8 Hz, 1H), 8.25 (s, 1H), 7.97 (d, *J* = 8.0 Hz, 2H), 7.90 (d, *J* = 8.8 Hz, 1H), 7.78 (d, *J* = 8.0 Hz, 2H), 7.29 (ddd, *J* = 8.8, 6.8, 0.8 Hz, 1H), 6.88 (td, *J* = 6.8, 0.8 Hz, 1H); ^{13}C NMR (101 MHz, $CDCl_3$) δ 191.5, 140.9, 139.6, 137.2, 133.9, 130.5, 129.3, 126.7, 125.2, 117.3, 112.6, 111.5; HRMS (ESI) *m*/*z* calcd for $C_{14}H_{11}N_2O$ $[M+H]^+$: 223.0866 found 223.0865.

2-2. Synthesis of 1H, 1I, and 1K

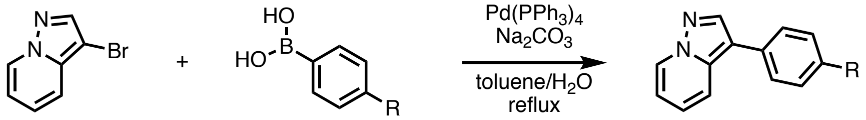

A round-bottom flask containing a magnetic stirring bar was dried with a heat-gun *in vacuo* and filled with N_2 gas after cooling to room temperature. To this flask were added $Pd(PPh_3)_4$ (10 mol%), 3-bromopyrazolo[1,5-*a*]pyridine[7] (1.0 equiv), and arylboronic acid (1.5 equiv). The flask was placed under vacuum and refilled with N_2 gas three times. To the flask were added degassed 1.0 M Na_2CO_3 aq. (3.0 equiv), and toluene (0.13 M) under stream of N_2 gas. The mixture was heated at 130 °C and stirred for several hours. After cooling the reaction mixture to room temperature, the reaction was quenched with H_2O. The mixture was extracted with Et_2O. The combined organic layer was dried over $MgSO_4$, filtered, and concentrated *in vacuo*. The residue was purified by Isolera® to afford the corresponding 3-arylpyrazolo[1,5-*a*]pyridine **1**.

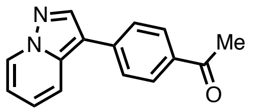

1-(4-(Pyrazolo[1,5-*a*]pyridin-3-yl)phenyl)ethan-1-one (1H)

Purification by Isolera® (hexane/EtOAc = 9:1 to 4:1) afforded **1H** (47.9 mg, 25% yield, 0.800 mmol scale) as a yellow solid. ^{1}H NMR (400 MHz, $CDCl_3$) δ 8.53 (d, *J* = 7.2 Hz, 1H), 8.23 (s, 1H), 8.05 (dt, *J* = 8.8, 1.6 Hz, 2H), 7.87 (dt, *J* = 8.8, 1.2 Hz, 1H), 7.70 (d, *J* = 8.0 Hz, 2H), 7.26 (td, *J* = 8.8, 1.2 Hz, 1H), 6.86 (td, *J* = 7.2, 0.8 Hz, 1H), 2.64 (s, 3H); ^{13}C NMR (101 MHz, $CDCl_3$) δ 197.3, 140.7, 138.1, 137.1, 134.5, 129.2, 129.1, 126.3, 124.9, 117.3, 112.4, 111.6, 26.5; HRMS (ESI) *m*/*z* calcd for $C_{15}H_{13}N_2O$ $[M+H]^+$: 237.1022 found 237.1021.

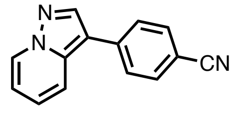

4-(Pyrazolo[1,5-*a*]pyridin-3-yl)benzonitrile (1I)[8]

Purification by Isolera® (hexane/EtOAc = 9:1 to 1:1) to afford **1I** (129 mg, 59% yield, 1.00 mmol scale) as a yellow solid. The spectra were in accordance with those of the compounds reported in the literature.

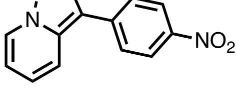

3-(4-Nitrophenyl)pyrazolo[1,5-*a*]pyridine (1K)

Purification by Isolera® (hexane/EtOAc = 9:1 to 1:1) afforded **1K** (55.5 mg, 29% yield, 0.800 mmol scale) as a yellow solid. ^{1}H NMR (400 MHz, $CDCl_3$) δ 8.56 (d, *J* = 6.8 Hz, 1H), 8.32 (d, *J* = 8.8 Hz,

2H), 8.25 (s, 1H), 7.88 (d, *J* = 9.2 Hz, 1H), 7.76 (d, *J* = 8.8 Hz, 2H), 7.36–7.29 (m, 1H), 6.91 (t, *J* = 6.8 Hz, 1H); ^{13}C NMR (101 MHz, $CDCl_3$) δ 145.6, 141.1, 140.2, 137.3, 129.6, 126.6, 125.7, 124.6, 117.2, 112.9, 110.7; HRMS (ESI) *m*/*z* calcd for $C_{13}H_{10}N_3O_2$ $[M+H]^+$: 240.0768 found 240.0767.

2-3. Synthesis of 1M and 1N

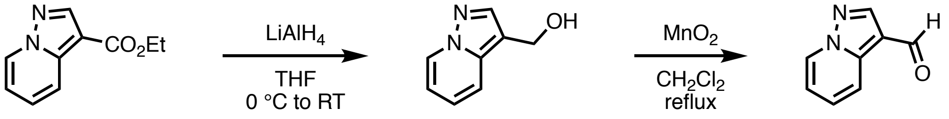

S1 S2

To a solution of **1F**[4] (965 mg, 5.10 mmol, 1.0 equiv) in THF (50 mL) was added lithium aluminum hydride ($LiAlH_4$: 212 mg, 5.58 mmol, 1.1 equiv) at 0 °C. After consumption of the starting material, the reaction was quenched with H_2O (0.30 mL), 3.0 M NaOH aq. (0.30 mL), and H_2O (0.90 mL). The precipitates were removed by filtration with Celite®. The filtrate was concentrated *in vacuo*. The residue was purified by Isolera® (hexane/EtOAc = 4:1 to 1:1) to afford pyrazolo[1,5-*a*]pyridin-3-ylmethanol (**S1**: 752 mg, quant.) as a yellow oil.

To a solution of **S1** (889 mg, 6.00 mmol, 1.0 equiv) in CH_2Cl_2 (24 mL) was added MnO_2 (5.22 g, 60.0 mmol, 10 equiv). The mixture was stirred with refluxing for 2 h. After cooling the reaction mixture to room temperature, the mixture was passed through a pad of Celite® with EtOAc as an eluent. The filtrate was concentrated *in vacuo*. The residue was purified by Isolera® (hexane/EtOAc = 9:1 to 4:1) to afford pyrazolo[1,5-*a*]pyridine-3-carbaldehyde (**S2**: 744 mg, 85% yield) as a white solid. The spectra were in accordance with those of the compounds reported in the literature.[9]

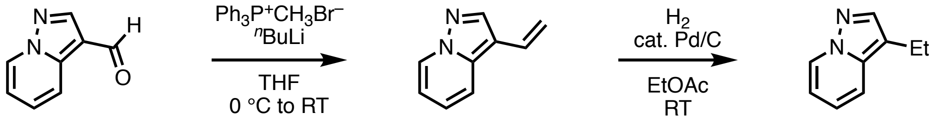

S2 1M

A two-necked flask containing a magnetic stirring bar was dried with a heat-gun *in vacuo* and filled with N_2 gas after cooling to room temperature. To this flask were added methyltriphenylphosphonium bromide (1.18 g, 3.30 mmol, 1.1 equiv) and THF (10 mL). The mixture was cooled to 0 °C and then *n*-butyllithium (1.57 M in hexane, 2.10 mL, 3.30 mmol, 1.1 equiv) was slowly added. After stirring at 0 °C for 15 min, **S2** (438 mg, 3.00 mmol, 1.0 equiv) was added. The mixture was further stirred at room temperature for 12 h. The reaction was quenched with NH_4Cl aq. The mixture was extracted with EtOAc and washed with brine. The combined organic layer was dried over Na_2SO_4, filtered, and concentrated *in vacuo*. The residue was purified by Isolera® (hexane/EtOAc = 19:1 to 15:1) to afford 3-vinylpyrazolo[1,5-*a*]pyridine (300 mg, 69% yield) as a yellow oil.

To a solution of 3-vinylpyrazolo[1,5-*a*]pyridine (300 mg, 2.08 mmol, 1.0 equiv) in EtOAc (20 mL) was added Pd/C (15.1 mg, 5 wt%). The flask was subjected to H_2 gas with a balloon (1 atm), then the mixture was stirred overnight at room temperature. The mixture was passed through a pad of Celite®.

The filtrate was concentrated *in vacuo*, and purified by Isolera® (hexane/EtOAc = 19:1 to 15:1) to afford 3-ethylpyrazolo[1,5-*a*]pyridine (**1M**: 267 mg, 88% yield) as a colorless oil. ^{1}H NMR (400 MHz, $CDCl_3$) δ 8.40 (d, J = 7.6 Hz, 1H), 7.80 (s, 1H), 7.45 (dt, J = 8.8, 1.2 Hz, 1H), 7.03 (ddd, J = 8.8, 7.6, 1.2 Hz, 1H), 6.69 (td, J = 7.6, 1.2 Hz, 1H), 2.75 (q, J = 7.6 Hz, 2H), 1.31 (t, J = 7.6 Hz, 3H); ^{13}C NMR (101 MHz, $CDCl_3$) δ 140.2, 137.6, 128.5, 121.7, 116.7, 113.0, 111.1, 16.7, 15.0; HRMS (ESI) *m/z* calcd for $C_9H_{11}N_2$ [M+H]$^+$: 147.0917 found 147.0916.

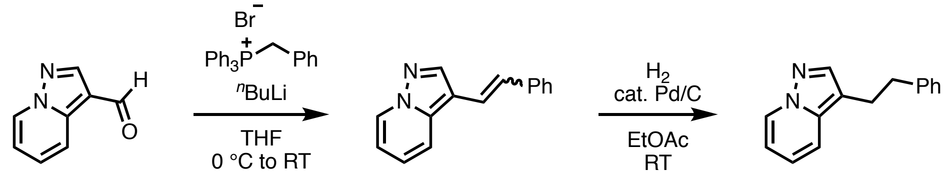

S2 **1N**

A two-necked flask containing a magnetic stirring bar was dried with a heat-gun *in vacuo* and filled with N_2 gas after cooling to room temperature. To this flask were added benzyltriphenylphosphonium bromide (953 mg, 2.20 mmol, 1.1 equiv) and THF (6.7 mL). The mixture was cooled to 0 °C and then *n*-butyllithium (1.57 M in hexane, 1.40 mL, 2.20 mmol, 1.1 equiv) was slowly added. After stirring at 0 °C for 15 min, **S2** (292 mg, 2.00 mmol, 1.0 equiv) was added. The mixture was further stirred at room temperature for 12 h. The reaction was quenched with NH_4Cl aq. The mixture was extracted with EtOAc and washed with brine. The combined organic layer was dried over Na_2SO_4, filtered, and concentrated *in vacuo*. The residue was purified by Isolera® (hexane/EtOAc = 19:1 to 15:1) to afford (*E*)-3-styrylpyrazolo[1,5-*a*]pyridine (174 mg, 40% yield) as a colorless oil and (*Z*)-3-styrylpyrazolo[1,5-*a*]pyridine (193 mg, 44% yield) as a colorless oil.

To a solution of (*E*)-3-styrylpyrazolo[1,5-*a*]pyridine (174 mg, 0.790 mmol, 1.0 equiv) in EtOAc (7.9 mL) was added Pd/C (8.70 mg, 5 wt%). The flask was subjected to H_2 gas with a balloon (1 atm). After stirred overnight at room temperature, the mixture was passed through a pad of Celite®. The filtrate was concentrated *in vacuo*, and purified by Isolera® (hexane/EtOAc = 19:1 to 15:1) to afford 3-phenethylpyrazolo[1,5-*a*]pyridine (**1N**: 146 mg, 84% yield) as a yellow oil. ^{1}H NMR (400 MHz, $CDCl_3$) δ 8.39 (d, J = 6.8 Hz, 1H), 7.74 (s, 1H), 7.31–7.24 (m, 3H), 7.23–7.15 (m, 3H), 7.02–6.96 (m, 1H), 6.68 (td, J = 6.8, 0.8 Hz, 1H), 3.07–3.00 (m, 2H), 2.99–2.92 (m, 2H); ^{13}C NMR (101 MHz, $CDCl_3$) δ 141.6, 140.9, 138.0, 128.5, 128.3, 126.0, 121.9, 116.6, 111.2, 110.4, 37.1, 25.6 (one peak is missing due to overlapping); HRMS (ESI) *m/z* calcd for $C_{15}H_{15}N_2$ [M+H]$^+$: 223.1230 found 223.1228.

2-4. Synthesis of 1O

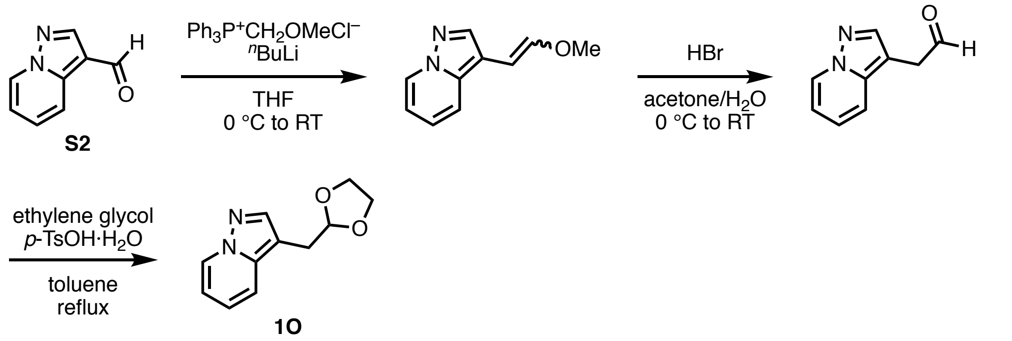

A two-necked flask containing a magnetic stirring bar was dried with a heat-gun *in vacuo* and filled with N_2 gas after cooling to room temperature. To this flask were added (methoxymethyl)triphenylphosphonium chloride (1.22 g, 3.57 mmol, 1.1 equiv) and THF (12 mL). The mixture was cooled to 0 °C and then *n*-butyllithium (1.57 M in hexane, 2.30 mL, 3.57 mmol, 1.1 equiv) was slowly added. After stirring the mixture at 0 °C for 15 min, **S2** (475.0 mg, 3.25 mmol, 1.0 equiv) was added. After stirring at room temperature for 12 h, the reaction was quenched with NH_4Cl aq. The mixture was extracted with EtOAc and washed with brine. The combined organic layer was dried over Na_2SO_4, filtered, and concentrated *in vacuo*. The crude material was used for the next step without further purification.

To a round-bottom flask containing the crude material in acetone (5.0 mL) was added HBr aq. (3.0 mL) slowly at 0 °C. The mixture was stirred at room temperature for 18 h. The reaction was quenched with $NaHCO_3$ aq. The mixture was extracted with EtOAc and washed with brine. The combined organic layer was dried over Na_2SO_4, filtered, and concentrated *in vacuo*. The residue was purified by Isolera® (hexane/EtOAc = 3:1 to 1:1) to afford 2-(pyrazolo[1,5-*a*]pyridin-3-yl)acetaldehyde (102 mg, 21% yield over two steps) as a white solid.

To a round-bottom flask containing 2-(pyrazolo[1,5-*a*]pyridin-3-yl)acetaldehyde (109 mg, 0.686 mmol, 1.0 equiv) were added *p*-toluenesulfonic acid monohydrate (6.50 mg, 34.0 μmol, 5.0 mol%), ethylene glycol (1.10 mL, 20.4 mmol, 30 equiv), and toluene (2.3 mL). The reaction mixture was stirred with refluxing for 5 h. After cooled to room temperature, the reaction was quenched with $NaHCO_3$ aq. The mixture was extracted with EtOAc and washed with brine. The combined organic layer was dried over Na_2SO_4, filtered, and concentrated *in vacuo*. The residue was purified by Isolera® (hexane/EtOAc = 3:1 to 1:1) to afford 3-((1,3-dioxolan-2-yl)methyl)pyrazolo[1,5-*a*]pyridine (**1O**: 88.2 mg, 63% yield) as a yellow oil. ^{1}H NMR (400 MHz, $CDCl_3$) δ 8.41 (d, *J* = 7.2 Hz, 1H), 7.88 (s, 1H), 7.56 (d, *J* = 9.2 Hz, 1H), 7.07 (ddd, *J* = 9.2, 6.8, 1.2 Hz, 1H), 6.71 (td, *J* = 6.8, 1.2 Hz, 1H), 5.09 (t, *J* = 4.8 Hz, 1H), 3.97–3.81 (m, 4H), 3.09 (d, *J* = 4.8 Hz, 2H); ^{13}C NMR (101 MHz, $CDCl_3$) δ 142.1, 138.9, 128.4, 122.3, 117.1, 111.3, 104.6, 104.1, 65.0, 28.9; HRMS (ESI) *m*/*z* calcd for $C_{11}H_{13}N_2O_2$ $[M+H]^+$: 205.0972 found 205.0971.

2-5. Synthesis of 1P

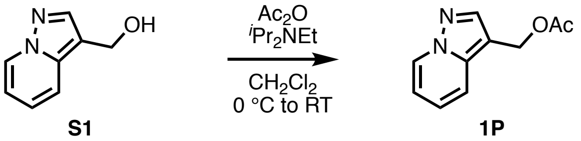

To a solution of **S1** (190 mg, 1.28 mmol, 1.0 equiv) in CH_2Cl_2 (6.4 mL) were added to acetic anhydride (130 μL, 1.41 mmol, 1.1 equiv) and *N,N*-diisopropylethylamine (268 μL, 1.54 mmol, 1.2 equiv) at 0 °C. The mixture was stirred at room temperature for 3 h. The reaction was quenched with H_2O. The mixture was extracted with EtOAc and washed with brine. The combined organic layer was dried over Na_2SO_4, filtered, and concentrated *in vacuo*. Purification by Isolera® (hexane/EtOAc = 19:1 to 9:1) afforded pyrazolo[1,5-*a*]pyridin-3-ylmethyl acetate (**1P**: 144 mg, 59% yield) as a colorless oil. ^{1}H NMR (400 MHz, $CDCl_3$) δ 8.46 (d, *J* = 7.2 Hz, 1H), 8.00 (s, 1H), 7.67 (d, *J* = 8.8 Hz, 1H), 7.19 (ddd, *J* = 8.8, 7.2, 1.2 Hz, 1H), 6.80 (td, *J* = 7.2, 1.2 Hz, 1H), 5.30 (s, 2H), 2.05 (s, 3H); ^{13}C NMR (101 MHz, $CDCl_3$) δ 171.1, 142.8, 139.3, 128.7, 123.9, 116.8, 112.1, 105.8, 56.5, 21.0; HRMS (ESI) *m*/*z* calcd for $C_{10}H_{11}N_2O_2$ $[M+H]^+$: 191.0815 found 191.0814.

2-6. Synthesis of 1R

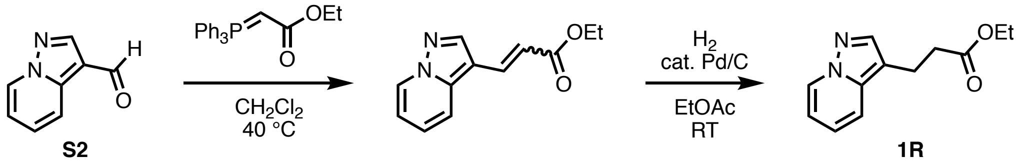

To a solution of **S2** (496 mg, 3.39 mmol, 1.0 equiv) in CH_2Cl_2 (11.3 mL) was added (carbethoxymethylene)triphenylphosphorane (2.36 g, 6.78 mmol, 2.0 equiv). The mixture was stirred at 40 °C for 12 h. After completion of the reaction, the solvent was removed under reduced pressure. The residue was purified by Isolera® (hexane/EtOAc = 19:1 to 9:1) to afford ethyl 3-(pyrazolo[1,5-*a*]pyridin-3-yl)acrylate (699 mg, 95% yield) as a white solid.

To a solution of ethyl 3-(pyrazolo[1,5-*a*]pyridin-3-yl)acrylate (659 mg, 3.05 mmol, 1.0 equiv) in EtOAc (15 mL) was added Pd/C (32.5 mg, 5 wt%). The flask was subjected to H_2 gas with a balloon (1 atm). After stirred for 16 h at room temperature, the mixture was passed through a pad of Celite®. The filtrate was concentrated *in vacuo*, and purified by Isolera® (hexane/EtOAc = 19:1 to 15:1) to afford ethyl 3-(pyrazolo[1,5-*a*]pyridin-3-yl)propanoate (**1R**: 462 mg, 69% yield) as a colorless oil. ^{1}H NMR (400 MHz, $CDCl_3$) δ 8.40 (d, *J* = 6.8 Hz, 1H), 7.80 (s, 1H), 7.49 (d, *J* = 9.2 Hz, 1H), 7.06 (ddd, *J* = 9.2, 6.8, 1.2 Hz, 1H), 6.71 (td, *J* = 6.8, 1.2 Hz, 1H), 4.12 (q, *J* = 7.2 Hz, 2H), 3.07 (t, *J* = 7.6 Hz, 2H), 2.66 (t, *J* = 7.6 Hz, 2H), 1.22 (t, *J* = 7.2 Hz, 3H); ^{13}C NMR (101 MHz, $CDCl_3$) δ 172.8, 140.7, 137.9, 128.5, 122.2, 116.6, 111.3, 109.3, 60.3, 35.3, 18.8, 14.1; HRMS (ESI) *m*/*z* calcd for $C_{12}H_{14}N_2O_2Na$ $[M+Na]^+$: 241.0948 found 241.0947.

2-7. Synthesis of 1T–1W

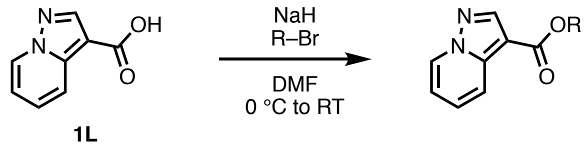

To a solution of **1L** (81.1 mg, 0.500 mmol, 1.0 equiv) in DMF (2.0 mL) was added sodium hydride (60% oil dispersion; 30.0 mg, 0.750 mmol, 1.5 equiv) at 0 °C. The mixture was stirred for 30 min at 0 °C and then alkyl bromide (0.750 mmol, 1.5 equiv) was added. The mixture was stirred for several hours at room temperature with monitoring the reaction progress by TLC. The reaction was quenched with NH_4Cl aq. at 0 °C. The mixture was extracted with EtOAc and washed with brine. The combined organic layer was dried over Na_2SO_4, filtered, and concentrated *in vacuo*. The residue was purified by Isolera® to afford the corresponding alkyl pyrazolo[1,5-*a*]pyridine-3-carboxylate **1**.

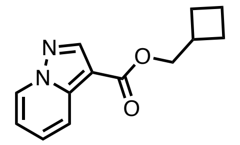

Cyclobutylmethyl pyrazolo[1,5-*a*]pyridine-3-carboxylate (1T)

Purification by Isolera® (hexane/EtOAc = 19:1 to 4:1) afforded **1T** (67.0 mg, 58% yield) as a colorless oil. ^{1}H NMR (400 MHz, $CDCl_3$) δ 8.53 (d, *J* = 6.8 Hz, 1H), 8.41 (s, 1H), 8.14 (d, *J* = 9.2 Hz, 1H), 7.40 (ddd, *J* = 9.2, 6.8, 1.2 Hz, 1H), 6.94 (td, *J* = 6.8, 1.2 Hz, 1H), 4.31 (d, *J* = 7.2 Hz, 2H), 2.86–2.72 (m, 1H), 2.23–2.07 (m, 2H), 2.85–1.84 (m, 4H); ^{13}C NMR (101 MHz, $CDCl_3$) δ 163.5, 144.8, 140.7, 129.2, 127.2, 119.0, 113.6, 103.9, 67.7, 34.3, 24.8, 18.4; HRMS (ESI) *m*/*z* calcd for $C_{13}H_{15}N_2O_2$ $[M+H]^+$: 231.1128 found 231.1127.

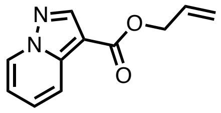

Allyl pyrazolo[1,5-*a*]pyridine-3-carboxylate (1U)

Purification by Isolera® (hexane/EtOAc = 19:1 to 9:1) afforded **1U** (65.7 mg, 65% yield) as a colorless oil. ^{1}H NMR (400 MHz, $CDCl_3$) δ 8.54 (d, *J* = 6.8 Hz, 1H), 8.43 (s, 1H), 8.18 (d, *J* = 9.2 Hz, 1H), 7.42 (ddd, *J* = 9.2, 6.8, 1.2 Hz, 1H), 6.96 (td, *J* = 6.8, 1.2 Hz, 1H), 6.13–6.01 (m, 1H), 5.49–5.38 (m, 1H), 5.34–5.26 (m, 1H), 4.85 (dt, *J* = 5.2, 1.6 Hz, 2H); ^{13}C NMR (101 MHz, $CDCl_3$) δ 163.0, 144.9, 140.9, 132.6, 129.3, 127.4, 119.1, 118.0, 113.7, 103.5, 64.6; HRMS (ESI) *m*/*z* calcd for $C_{11}H_{11}N_2O_2$ $[M+H]^+$: 203.0815 found 203.0815.

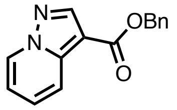

Benzyl pyrazolo[1,5-*a*]pyridine-3-carboxylate (1V)

Purification by Isolera® (hexane/EtOAc = 19:1 to 4:1) afforded **1V** (82.0 mg, 65% yield) as a yellow oil. ^{1}H NMR (400 MHz, $CDCl_3$) δ 8.51 (d, *J* = 7.2 Hz, 1H), 8.43 (s, 1H), 8.14 (d, *J* = 8.4 Hz, 1H), 7.47 (d, *J* = 7.2 Hz, 2H), 7.42–7.31 (m, 4H), 6.93 (td, *J* = 6.8, 0.8 Hz, 1H), 5.38 (s, 2H); ^{13}C NMR (101 MHz, $CDCl_3$) δ 163.1, 144.9, 140.9, 136.4, 129.3, 128.5, 128.1, 127.4, 119.0, 113.7, 103.5, 65.7 (one peak is missing due to overlapping); HRMS (ESI) *m*/*z* calcd for $C_{15}H_{13}N_2O_2$ $[M+H]^+$: 253.0972 found 253.0970.

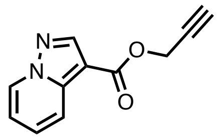

Prop-2-yn-1-yl pyrazolo[1,5-*a*]pyridine-3-carboxylate (1W)

Purification by Isolera® (hexane/EtOAc = 19:1 to 4:1) afforded **1W** (80.2 mg, 64% yield) as a white solid. ^{1}H NMR (400 MHz, $CDCl_3$) δ 8.55 (d, *J* = 6.8 Hz, 1H), 8.44 (s, 1H), 8.19 (d, *J* = 8.8 Hz, 1H), 7.45 (ddd, *J* = 8.8, 6.8, 0.8 Hz, 1H), 6.93 (td, *J* = 6.8, 0.8 Hz, 1H), 4.94 (d, *J* = 2.4 Hz, 2H), 2.52 (t, *J* = 2.4 Hz, 1H); ^{13}C NMR (101 MHz, $CDCl_3$) δ 162.3, 145.0, 141.0, 129.4, 127.7, 119.0, 113.9, 102.7, 78.1, 74.7, 51.5; HRMS (ESI) *m*/*z* calcd for $C_{11}H_9N_2O_2$ $[M+H]^+$: 201.0659 found 201.0658.

2-8. Synthesis of 1X

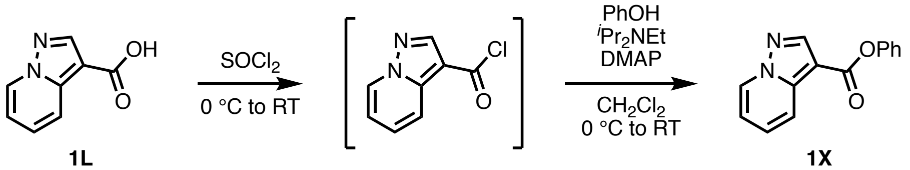

1L **1X**

To a round-bottom flask with **1L** (162 mg, 1.00 mmol, 1.0 equiv) was added thionyl chloride (2.40 mL, 4.00 mmol, 4.0 equiv). After the mixture was stirred at room temperature for 1 h, the solution was concentrated *in vacuo*. To the resulting mixture were added *N,N*-dimethylaminopyridine (DMAP: 11.0 mg, 50.0 μmol, 5.0 mol%), phenol (104 mg, 1.10 mmol, 1.1 equiv), CH_2Cl_2 (4.0 mL), and then *N,N*-diisopropylethylamine (210 μL, 1.20 mmol, 1.2 equiv) slowly at 0 °C. The mixture was stirred for 12 h. After the reaction was quenched with $NaHCO_3$ aq., the mixture was extracted with CH_2Cl_2. The combined organic layer was dried over Na_2SO_4, filtered, and concentrated *in vacuo*. The residue was purified by Isolera® (hexane/EtOAc = 19:1 to 9:1) to afford phenyl pyrazolo[1,5-*a*]pyridine-3-carboxylate (**1X**: 196 mg, 82% yield) as a white solid. ^{1}H NMR (400 MHz, $CDCl_3$) δ 8.61–8.54 (m, 2H), 8.22 (d, *J* = 9.2 Hz, 1H), 7.49–7.39 (m, 3H), 7.30–7.22 (m, 3H), 7.00 (t, *J* = 6.8 Hz, 1H); ^{13}C NMR (101 MHz, $CDCl_3$) δ 161.6, 150.6, 145.3, 141.3, 129.43, 129.37, 127.9, 125.6, 121.9, 119.1, 114.0, 102.8; HRMS (ESI) *m*/*z* calcd for $C_{14}H_{11}N_2O_2$ $[M+H]^+$: 239.0815 found 239.0815.

2-9. Synthesis of 1Z

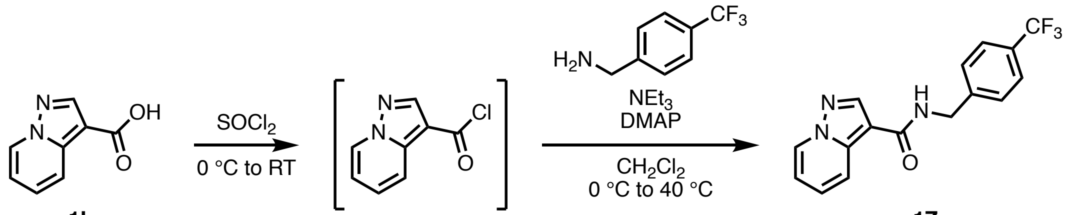

To a round-bottom flask containing a magnetic stirring bar and **1L** (361 mg, 2.00 mmol, 1.0 equiv) was added thionyl chloride (4.90 mL, 8.00 mmol, 4.0 equiv). After the mixture was stirred at room temperature for 1 h, the solution was concentrated *in vacuo*. To the resulting mixture were added *N,N*-dimethylaminopyridine (DMAP: 12.2 mg, 0.100 mmol, 5.0 mol%), 4-(trifluoromethyl)benzylamine (450 μL, 3.00 mmol, 1.5 equiv), CH_2Cl_2 (8.0 mL), and then triethylamine (340 μL, 2.40 mmol, 1.2 equiv) slowly at 0 °C. The reaction mixture was stirred at 40 °C for 4 h. After the reaction was quenched with $NaHCO_3$ aq., the mixture was extracted with CH_2Cl_2. The combined organic layer was dried over Na_2SO_4, filtered, and concentrated *in vacuo*. The residue was purified by Isolera® (hexane/EtOAc = 19:1 to 9:1) to afford *N*-(4-(trifluoromethyl)benzyl)pyrazolo[1,5-*a*]pyridine-3-carboxamide (**1Z**: 449 mg, 66% yield) as a white solid. ^{1}H NMR (400 MHz, $CDCl_3$) δ 8.49 (d, J = 6.8 Hz, 1H), 8.32 (d, J = 8.8 Hz, 1H), 8.18 (s, 1H), 7.59 (d, J = 8.4 Hz, 2H), 7.48 (d, J = 8.4 Hz, 2H), 7.37 (dd, J = 8.8, 6.8 Hz, 1H), 6.94 (t, J = 6.8 Hz, 1H), 6.40 (brs, 1H), 4.71 (d, J = 6.0 Hz, 2H); ^{13}C NMR (101 MHz, $CDCl_3$) δ 163.3, 142.8, 140.7, 140.4, 129.5 (q, J_{C-F} = 33.0 Hz), 128.9, 127.8, 126.7, 125.5 (q, J_{C-F} = 3.8 Hz), 124.0 (q, J_{C-F} = 273.5 Hz), 119.5, 113.8, 106.2, 42.8; ^{19}F NMR (376 MHz, $CDCl_3$) δ –62.5; HRMS (ESI) *m*/*z* calcd for $C_{16}H_{13}F_3N_3O$ $[M+H]^+$: 320.1005 found 320.1005.

2-10. Synthesis of Probenecid Derivative 1AA

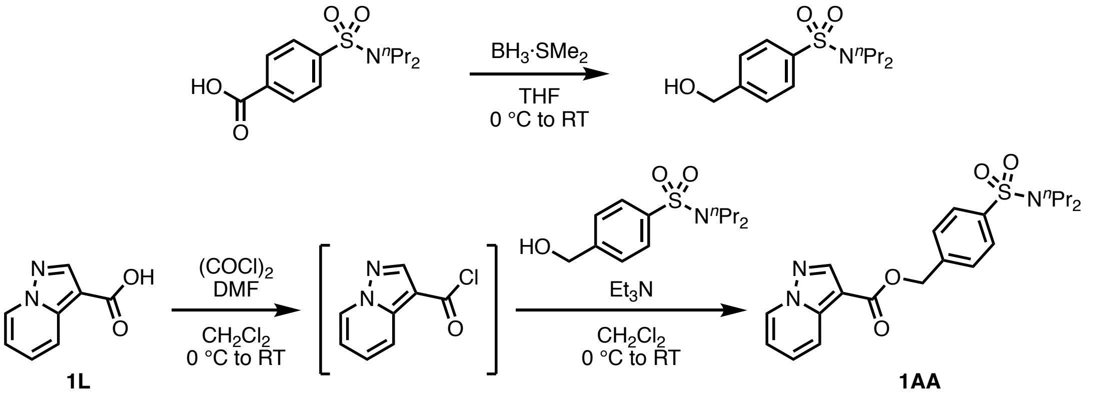

To a solution of probenecid (856 mg, 3.00 mmol, 1.0 equiv) in THF (30 mL) was added $BH_3 \cdot SMe_2$ (570 μL, 6.00 mmol, 2.0 equiv) dropwise at 0 °C. The mixture was stirred at room temperature for 24 h. The reaction was quenched with MeOH carefully at 0 °C. Solvent was removed *in vacuo* and the

residue was purified by Isolera® (hexane/EtOAc = 4:1 to 1:1) to afford 4-(hydroxymethyl)-*N*,*N*-dipropylbenzenesulfonamide (814 mg, quant.) as a colorless oil.

To a solution of **1L** (81.1 mg, 0.500 mmol, 1.0 equiv) in CH_2Cl_2 (5.0 mL) were added oxalyl chloride (52.0 μL, 0.600 mmol, 1.2 equiv) and one drop of DMF. After the mixture was stirred at room temperature for 1.5 h, the solution was concentrated *in vacuo*. To the resulting mixture were added 4-(hydroxymethyl)-*N*,*N*-dipropylbenzenesulfonamide (163 mg, 0.600 mmol, 1.2 equiv), CH_2Cl_2 (5.0 mL), and then triethylamine (200 μL, 1.50 mmol, 3.0 equiv) slowly at 0 °C. The mixture was stirred at room temperature for 12 h. After the reaction was quenched with $NaHCO_3$ aq., the mixture was extracted with CH_2Cl_2. The combined organic layer was dried over Na_2SO_4, filtered, and concentrated *in vacuo*. The residue was purified by PTLC (toluene/EtOAc = 2:1) to afford 4-(*N*,*N*-dipropylsulfamoyl)benzyl pyrazolo[1,5-*a*]pyridine-3-carboxylate (**1AA**: 112 mg, 54% yield) as a white solid. ^{1}H NMR (400 MHz, $CDCl_3$) δ 8.56 (dt, *J* = 6.8, 1.2 Hz, 1H), 8.45 (s, 1H), 8.15 (dd, *J* = 8.8, 1.2 Hz, 1H), 7.83 (d, *J* = 8.0 Hz, 2H), 7.58 (d, *J* = 8.0 Hz, 2H), 7.42 (ddd, *J* = 8.8, 6.8, 1.2 Hz, 1H), 6.99 (td, *J* = 6.8, 1.2 Hz, 1H), 5.44 (s, 2H), 3.11–3.05 (m, 4H), 1.62–1.51 (m, 4H), 0.88 (t, *J* = 7.2 Hz, 6H); ^{13}C NMR (101 MHz, $CDCl_3$) δ 162.8, 144.9, 141.0, 140.9, 139.7, 129.4, 128.1, 127.7, 127.3, 118.9, 113.9, 103.0, 64.5, 50.1, 22.0, 11.1; HRMS (ESI) *m*/*z* calcd for $C_{21}H_{26}N_3O_4S$ $[M+H]^+$: 416.1639 found 416.1635.

2-11. Synthesis of Estrone Derivative 1AB

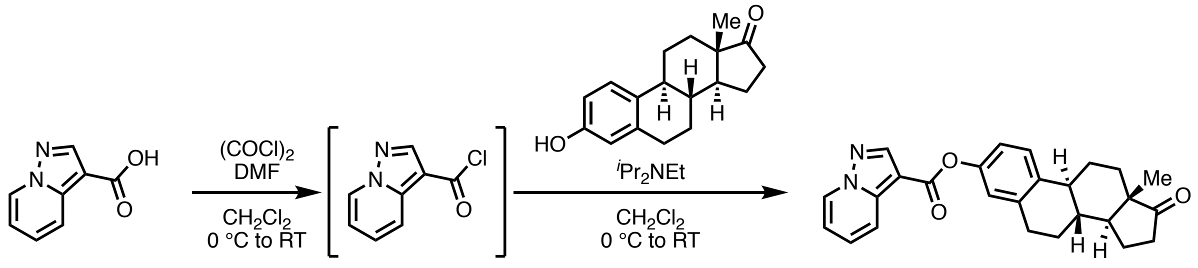

To a solution of **1L** (162 mg, 1.00 mmol, 1.0 equiv) in CH_2Cl_2 (5.0 mL) were added oxalyl chloride (103 μL, 1.20 mmol, 1.2 equiv) and one drop of DMF. After the mixture was stirred at room temperature for 1.5 h, the solution was concentrated *in vacuo*. To the resulting mixture were added estrone (324 mg, 1.20 mmol, 1.2 equiv), CH_2Cl_2 (5.0 mL), and then *N*,*N*-diisopropylethylamine (440 μL, 2.50 mmol, 2.5 equiv) slowly at 0 °C. The mixture was stirred at room temperature for 12 h. After the reaction was quenched with $NaHCO_3$ aq., the mixture was extracted with CH_2Cl_2. The combined organic layer was dried over Na_2SO_4, filtered, and concentrated *in vacuo*. The residue was purified by PTLC (hexane/EtOAc = 2:1) to afford (8*R*,9*S*,13*S*,14*S*)-13-methyl-17-oxo-7,8,9,11,12,13,14,15,16,17-decahydro-6*H*-cyclopenta[*a*]phenanth-ren-3-yl pyrazolo[1,5-*a*]pyridine-3-carboxylate (**1AB**: 281 mg, 68% yield) as a white solid. ^{1}H NMR (400 MHz, $CDCl_3$) δ 8.59 (d, *J* = 6.8 Hz, 1H), 8.55 (s, 1H), 8.22 (d, *J* = 8.8 Hz, 1H), 7.47 (ddd, *J* = 8.8, 6.8, 1.2 Hz, 1H), 7.35 (d, *J* = 8.8 Hz, 1H), 7.05–6.96 (m, 3H), 2.95 (dd, *J* = 8.8, 4.4 Hz, 2H), 2.52 (dd, *J* = 18.8, 8.4 Hz, 1H), 2.48–2.40 (m, 1H), 2.38–2.28 (m, 1H),

2.22–2.15 (m, 4H), 1.71–1.42 (m, 6H), 0.93 (s, 3H); ^{13}C NMR (101 MHz, $CDCl_3$) δ 220.7, 161.8, 148.4, 145.2, 141.3, 137.9, 137.2, 129.4, 127.8, 126.3, 121.9, 119.1, 114.0, 102.9, 50.3, 47.9, 44.1, 37.9, 35.8, 31.5, 29.6, 29.4, 26.3, 25.7, 21.5, 13.8; HRMS (ESI) *m/z* calcd for $C_{26}H_{27}N_2O_3$ $[M+H]^+$: 415.2016 found 415.2013.

2-12. Synthesis of 1AC–1AF

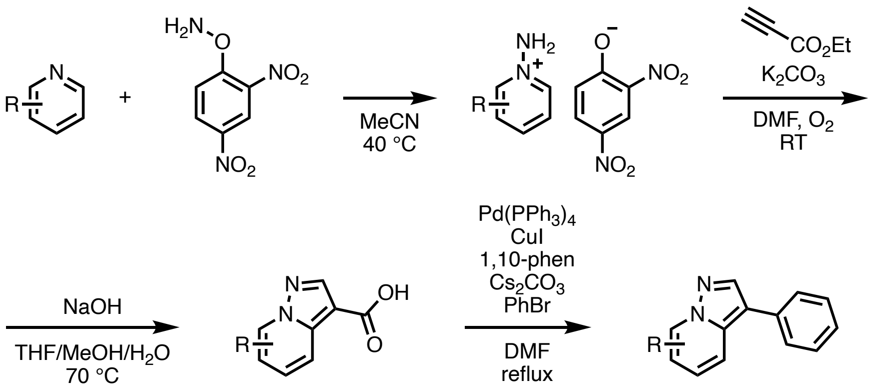

To a round-bottom flask containing a magnetic stirring bar were added methylpyridine (446 mg, 5.00 mmol, 1.0 equiv) and MeCN (4.0 mL). To this mixture was added *O*-(2,4-dinitrophenyl)hydroxylamine[10] (996 mg, 5.00 mmol, 1.0 equiv) in one portion. The mixture was stirred at 40 °C for 24 h. The solvent was removed under reduced pressure and the residue was triturated with Et_2O. The resulting solid was filtered and dried *in vacuo*. The obtained solid containing *N*-aminopyridinium salt was used for the next step without further purification.

To a round-bottom flask containing a magnetic stirring bar were added a solution of the crude material in DMF (2.0 mL), potassium carbonate (967 mg, 7.00 mmol, 1.4 equiv) and ethyl propiolate (562 μL, 5.50 mmol, 1.1 equiv) at room temperature. Air was introduced to the reaction mixture by diaphragm pump. The reaction mixture was stirred for 24 h. The solvent was removed *in vacuo*. The mixture was extracted with Et_2O and washed with brine. The combined organic layer was dried over $MgSO_4$, filtered, and concentrated *in vacuo*. The residue was purified by Isolera® to afford the corresponding ethyl methylpyrazolo[1,5-*a*]pyridine-3-carboxylate.

To a round-bottom flask containing a magnetic stirring bar and ethyl methylpyrazolo[1,5-*a*]pyridine-3-carboxylate (1.0 equiv) were added THF (0.9 M), MeOH (0.9 M), H_2O (0.9 M), and 6.0 M NaOH aq. (2.0 equiv) at room temperature. The reaction mixture was stirred at 70 °C. After completion of the reaction, the mixture was adjusted to pH = 7 with DOWEX® 50W. The mixture was filtered, washed with MeOH, and the filtrate was concentrated *in vacuo* to afford the corresponding methylpyrazolo[1,5-*a*]pyridine-3-carboxylic acid. The crude material was used for the next step without further purification.

A round-bottom flask containing a magnetic stirring bar and cesium carbonate (1.5 equiv) was dried with a heat-gun *in vacuo* and filled with N_2 gas after cooling to room temperature. To this flask were added $Pd(PPh_3)_4$ (5.0 mol%), copper iodide (10 mol%), 1,10-phenanthroline (10 mol%), and

methylpyrazolo[1,5-*a*]pyridine-3-carboxylic acid (1.0 equiv). The flask was placed under vacuum and refilled with N_2 gas three times. To this flask were added bromobenzene (1.0 equiv) and DMF (0.2 M). The mixture was heated under reflux and stirred for several hours. After cooled to room temperature, the mixture was passed through a short silica-gel pad with EtOAc as an eluent, and then concentrated *in vacuo*. The residue was purified by Isolera® to afford the corresponding methyl-3-phenylpyrazolo[1,5-*a*]pyridine **1**.

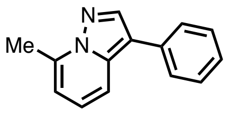

7-Methyl-3-phenylpyrazolo[1,5-*a*]pyridine (1AC)

Purification by Isolera® (hexane/EtOAc = 49:1 to 19:1) afforded **1AC** (533 mg, 51% yield over 4 steps) as a colorless oil. ^{1}H NMR (400 MHz, $CDCl_3$) δ 8.19 (s, 1H), 7.75 (d, *J* = 8.8 Hz, 1H), 7.61 (dd, *J* = 8.0, 1.2 Hz, 2H), 7.45 (t, *J* = 8.0 Hz, 2H), 7.29 (t, *J* = 7.6 Hz, 1H), 7.14 (dd, *J* = 8.8, 6.8 Hz, 1H), 6.67 (d, *J* = 6.8 Hz, 1H), 2.78 (s, 3H); ^{13}C NMR (101 MHz, $CDCl_3$) δ 139.9, 138.5, 137.3, 133.5, 128.9, 127.1, 126.1, 124.0, 115.0, 113.1, 111.4, 17.9; HRMS (ESI) *m*/*z* calcd for $C_{14}H_{13}N_2$ $[M+H]^+$: 209.1073 found 209.1072.

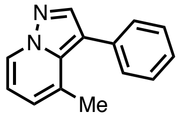

4-Methyl-3-phenylpyrazolo[1,5-*a*]pyridine (1AD)

Purification by Isolera® (hexane/EtOAc = 49:1 to 19:1) afforded **1AD** (294 mg, 28% yield over 4 steps) as a colorless oil. ^{1}H NMR (400 MHz, $CDCl_3$) δ 8.37 (d, *J* = 7.2 Hz, 1H), 7.89 (s, 1H), 7.41–7.34 (m, 5H), 6.84 (dt, *J* = 7.2, 1.2 Hz, 1H), 6.68 (t, *J* = 7.2 Hz, 1H), 2.21 (s, 3H); ^{13}C NMR (101 MHz, $CDCl_3$) δ 142.0, 137.2, 134.1, 130.9, 128.8, 127.7, 126.8, 123.4, 114.3, 111.6, 19.8 (one peak is missing due to overlapping); HRMS (ESI) *m*/*z* calcd for $C_{14}H_{13}N_2$ $[M+H]^+$: 209.1073 found 209.1072.

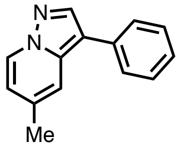

5-Methyl-3-phenylpyrazolo[1,5-*a*]pyridine (1AE)

Purification by Isolera® (hexane/EtOAc = 49:1 to 19:1) afforded **1AE** (167 mg, 16% yield over 4 steps) as a colorless oil. ^{1}H NMR (400 MHz, $CDCl_3$) δ 8.36 (d, *J* = 6.8 Hz, 1H), 8.09 (s, 1H), 7.60–7.57 (m, 3H), 7.45 (t, *J* = 7.8 Hz, 2H), 7.28 (t, *J* = 7.8 Hz, 1H), 6.61 (dd, *J* = 6.8, 1.6 Hz, 1H), 2.40 (s, 3H); ^{13}C NMR (101 MHz, $CDCl_3$) δ 140.6, 137.1, 134.8, 133.5, 128.9, 128.2, 126.9, 126.0, 115.7, 114.6, 111.7, 21.4; HRMS (ESI) *m*/*z* calcd for $C_{14}H_{13}N_2$ $[M+H]^+$: 209.1073 found 209.1073.

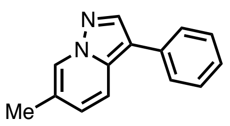

6-Methyl-3-phenylpyrazolo[1,5-*a*]pyridine (1AF)

Purification by Isolera® (hexane/EtOAc = 49:1 to 19:1) afforded **1AF** (127 mg, 12% yield over 4 steps) as a white solid. ^{1}H NMR (400 MHz, $CDCl_3$) δ 8.29 (s, 1H), 8.08 (s, 1H), 7.73 (d, J = 9.2 Hz, 1H), 7.59 (dd, J = 8.0, 1.2 Hz, 2H), 7.44 (t, J = 8.0 Hz, 2H), 7.29 (t, J = 8.0 Hz, 1H), 7.04 (d, J = 9.2 Hz, 1H), 2.36 (s, 3H); ^{13}C NMR (101 MHz, $CDCl_3$) δ 139.7, 135.5, 133.4, 128.9, 127.1, 126.90, 126.87, 126.0, 121.8, 116.8, 112.5, 18.1; HRMS (ESI) *m/z* calcd for $C_{14}H_{13}N_2$ $[M+H]^+$: 209.1073 found 209.1072.

3. Preparation of Other Bicyclic or Tricyclic Azaarenes 1

3-1. Synthesis of 1AH and 1AI

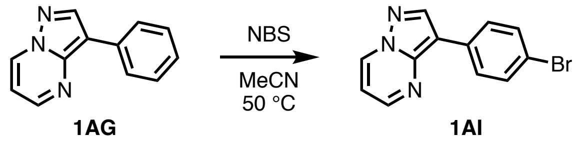

To a solution of **1AG** (78.1 mg, 0.400 mol, 1.0 equiv) in MeCN (2.0 mL) was added *N*-bromosuccinimide (NBS: 78.3 mg, 0.440 mmol, 1.1 equiv) in one portion at room temperature. The mixture was stirred at 50 °C for 24 h. After cooling the mixture to room temperature, the mixture was concentrated *in vacuo*. The residue was purified by Isolera® (hexane/EtOAc = 49:1 to 19:1) to afford 3-(4-bromophenyl)pyrazolo[1,5-*a*]pyrimidine (**1AI**: 109 mg, quant.) as a yellow solid. ^{1}H NMR (400 MHz, $CDCl_3$) δ 8.70 (dd, J = 7.2, 1.6 Hz, 1H), 8.57 (dd, J = 3.6, 1.6 Hz, 1H), 8.42 (s, 1H), 7.93 (d, J = 8.8 Hz, 2H), 7.57 (d, J = 8.8 Hz, 2H), 6.87 (dd, J = 7.2, 3.6 Hz, 1H); ^{13}C NMR (101 MHz, $CDCl_3$) δ 149.4, 144.8, 142.6, 135.4, 131.8, 130.8, 127.8, 120.1, 109.7, 108.2; HRMS (ESI) *m/z* calcd for $C_{12}H_9BrN_3$ $[M+H]^+$: 273.9974 found 273.9973.

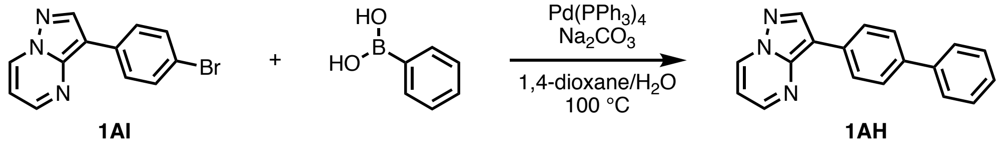

A round-bottom flask containing a magnetic stirring bar was dried with a heat-gun *in vacuo* and filled with N_2 gas after cooling to room temperature. To this flask were added $Pd(PPh_3)_4$ (23.1 mg, 20.0 μmol, 5.0 mol%), **1AI** (109 mg, 0.400 mmol, 1.0 equiv), and phenylboronic acid (48.8 mg, 0.400 mmol, 1.0 equiv). The flask was placed under vacuum and refilled with N_2 gas three times. To the flask were added degassed 1.0 M Na_2CO_3 aq. (800 μL, 0.800 mmol, 2.0 equiv) and 1,4-dioxane (3.2 mL) under stream of N_2 gas. The mixture was stirred at 100 °C for 18 h. After cooling the reaction mixture to room temperature, H_2O was added. The mixture was extracted with Et_2O and washed with brine. The

combined organic layer was dried over $MgSO_4$, filtered, and concentrated *in vacuo*. The residue was purified by Isolera® (hexane/EtOAc = 19:1 to 4:1) to afford 3-([1,1'-biphenyl]-4-yl)pyrazolo[1,5-*a*]pyrimidine (**1AH**: 86.8 mg, 80% yield) as a yellow solid. ^1H NMR (400 MHz, $CDCl_3$) δ 8.71 (dd, *J* = 6.8, 1.2 Hz, 1H), 8.59 (dd, *J* = 4.0, 1.2 Hz, 1H), 8.49 (s, 1H), 8.12 (d, *J* = 8.0 Hz, 2H), 7.70 (d, *J* = 8.0 Hz, 2H), 7.65 (d, *J* = 7.2 Hz, 2H), 7.46 (t, *J* = 7.2 Hz, 2H), 7.35 (t, *J* = 7.2 Hz, 1H), 6.87 (dd, *J* = 6.8, 4.0 Hz, 1H); ^{13}C NMR (101 MHz, $CDCl_3$) δ 149.2, 144.9, 142.8, 140.9, 139.1, 135.4, 130.9, 128.8, 127.5, 127.2, 126.9, 126.7, 110.5, 108.1; HRMS (ESI) *m*/*z* calcd for $C_{18}H_{14}N_3$ $[M+H]^+$: 272.1182 found 272.1180.

3-2. Synthesis of 1AJ–1AL

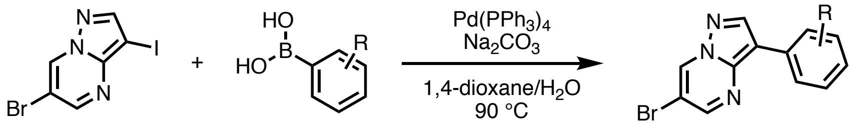

A round-bottom flask containing a magnetic stirring bar was dried with a heat-gun *in vacuo* and filled with N_2 gas after cooling to room temperature. To this flask were added $Pd(PPh_3)_4$ (5.0 mol%), 6-bromo-3-iodopyrazolo[1,5-*a*]pyrimidine[11] (1.0 equiv), and aryl boronic acid (1.0 equiv). The flask was placed under vacuum and refilled with N_2 gas three times. To the flask were added degassed 1.0 M Na_2CO_3 aq. (2.0 equiv) and 1,4-dioxane (0.10 M) under stream of N_2 gas. The mixture was stirred at 90 °C for 24 h. After cooling the reaction mixture to room temperature, H_2O was added. The mixture was extracted with Et_2O and washed with brine. The combined organic layer was dried over $MgSO_4$, filtered, and concentrated *in vacuo*. The residue was purified by Isolera® to afford the corresponding 3-aryl-6-bromopyrazolo[1,5-*a*]pyrimidine **1**.

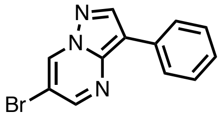

6-Bromo-3-phenylpyrazolo[1,5-*a*]pyrimidine (1AJ)

Purification by Isolera® (hexane/EtOAc = 99:1 to 19:1) afforded **1AJ** (203 mg, 74% yield, 1.00 mmol scale) as a yellow solid. ^1H NMR (400 MHz, $CDCl_3$) δ 8.83 (d, *J* = 2.4 Hz, 1H), 8.53 (d, *J* = 2.4 Hz, 1H), 8.41 (s, 1H), 8.00 (dd, *J* = 7.6, 1.2 Hz, 2H), 7.46 (t, *J* = 7.6 Hz, 2H), 7.30 (t, *J* = 7.6 Hz, 1H); ^{13}C NMR (101 MHz, $CDCl_3$) δ 149.8, 143.1, 142.9, 135.0, 131.1, 128.8, 126.7, 126.3, 111.7, 103.7; HRMS (ESI) *m*/*z* calcd for $C_{12}H_9BrN_3$ $[M+H]^+$: 273.9974 found 273.9974.

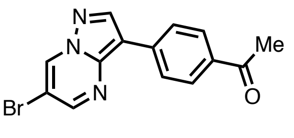

1-(4-(6-Bromopyrazolo[1,5-*a*]pyrimidin-3-yl)phenyl)ethan-1-one (1AK)

Purification by Isolera® (hexane/EtOAc = 49:1 to 19:1) afforded **1AK** (128 mg, 51% yield, 0.800 mmol scale) as a yellow solid. ^{1}H NMR (400 MHz, $CDCl_3$) δ 8.86 (d, *J* = 2.0 Hz, 1H), 8.60 (d, *J* = 2.0 Hz, 1H), 8.48 (s, 1H), 8.14 (d, *J* = 8.8 Hz, 2H), 8.05 (d, *J* = 8.8 Hz, 2H), 2.64 (s, 3H); ^{13}C NMR (101 MHz, $CDCl_3$) δ 197.6, 150.6, 143.5, 143.4, 136.1, 135.3, 135.1, 129.0, 126.0, 110.5, 104.3, 26.6; HRMS (ESI) *m*/*z* calcd for $C_{14}H_{11}BrN_3O$ $[M+H]^+$: 316.0080 found 316.0081.

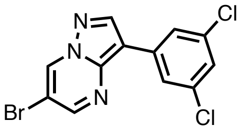

6-Bromo-3-(3,5-dichlorophenyl)pyrazolo[1,5-*a*]pyrimidine (1AL)

Purification by Isolera® (hexane/EtOAc = 49:1 to 19:1) afforded **1AL** (189 mg, 69% yield, 0.800 mmol scale) as a yellow solid. ^{1}H NMR (400 MHz, $CDCl_3$) δ 8.85 (d, *J* = 2.4 Hz, 1H), 8.59 (d, *J* = 2.4 Hz, 1H), 8.39 (s, 1H), 7.95 (d, *J* = 2.0 Hz, 2H), 7.27 (t, *J* = 2.0 Hz, 1H); ^{13}C NMR (101 MHz, $CDCl_3$) δ 150.7, 143.1, 135.4, 134.1, 126.5, 124.2, 114.7, 108.9, 104.6; HRMS (ESI) *m*/*z* calcd for $C_{12}H_6BrCl_2N_3$ $[M]^+$: 340.9117 found 340.9115.

3-3. Synthesis of 1AN

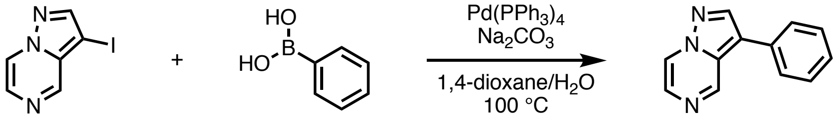

A round-bottom flask containing a magnetic stirring bar was dried with a heat-gun *in vacuo* and filled with N_2 gas after cooling to room temperature. To this flask were added $Pd(PPh_3)_4$ (57.7 mg, 50.0 µmol, 5.0 mol%), 3-iodopyrazolo[1,5-*a*]pyrazine[12] (245 mg, 1.00 mmol, 1.0 equiv), and phenylboronic acid (121 mg, 1.00 mmol, 1.0 equiv). The flask was placed under vacuum and refilled with N_2 gas three times. To the flask were added degassed 1.0 M Na_2CO_3 aq. (2.0 mL, 2.00 mmol, 2.0 equiv) and 1,4-dioxane (8.0 mL) under stream of N_2 gas. The mixture was stirred at 100 °C for 12 h. After cooling the reaction mixture to room temperature, H_2O was added. The mixture was extracted with Et_2O and washed with brine. The combined organic layer was dried over $MgSO_4$, filtered, and concentrated *in vacuo*. The residue was purified by Isolera® (hexane/EtOAc = 19:1 to 4:1) to afford 3-phenylpyrazolo[1,5-*a*]pyrazine (**1AN**: 195 mg, quant.) as a pale yellow solid. ^{1}H NMR (400 MHz, $CDCl_3$) δ 9.30 (d, *J* = 1.6 Hz, 1H), 8.40 (dd, *J* = 4.8, 1.6 Hz, 1H), 8.21 (s, 1H), 7.90 (d, *J* = 4.8 Hz, 1H), 7.66–7.63 (m, 2H), 7.53–7.49 (m, 2H), 7.40–7.36 (m, 1H); ^{13}C NMR (101 MHz, $CDCl_3$) δ 144.5, 140.3, 132.4, 131.4, 129.5, 129.3, 127.5, 127.4, 122.0, 115.7; HRMS (ESI) *m*/*z* calcd for $C_{12}H_{10}N_3$ $[M+H]^+$: 196.0869 found 196.0869.

3-4. Synthesis of 1AO

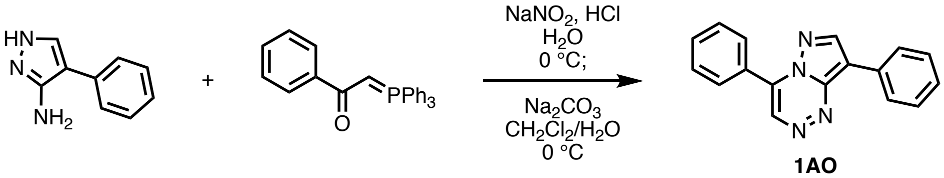

1AO

To a round-bottom flask containing a magnetic stirring bar were added 4-phenyl-1*H*-pyrazol-3-amine[13] (79.6 mg, 0.500 mmol, 1.0 equiv) and conc. HCl (0.20 mL) and H_2O (0.40 mL). To the mixture was added a solution of $NaNO_2$ (38.0 mg, 0.550 mmol, 1.1 equiv) in H_2O (0.13 mL) dropwise at 0 °C. After the mixture was stirred for 30 min at 0 °C, CH_2Cl_2 (4.0 mL) and Na_2CO_3 aq. (2.2 mL) were added. To the resulting mixture was added a solution of 1-phenyl-2-(triphenyl-λ^5-phosphaneylidene)ethan-1-one[14] (209 mg, 0.550 mmol, 1.1 equiv) in CH_2Cl_2 (1.9 mL) dropwise at 0 °C. After stirring for 5 min, CH_2Cl_2 and H_2O added and the mixture was extracted with CH_2Cl_2. The combined organic layer was dried over Na_2SO_4, filtered, and concentrated *in vacuo*. The residue was purified by Isolera® (hexane/EtOAc = 19:1 to 4:1) to afford 4,8-diphenylpyrazolo[5,1-*c*][1,2,4]triazine (**1AO**: 91.4 mg, 68% yield) as a yellow solid. ^{1}H NMR (400 MHz, $CDCl_3$) δ 8.96 (s, 1H), 8.61 (s, 1H), 8.26 (dd, *J* = 8.8, 1.2 Hz, 2H), 8.23–8.21 (m, 2H), 7.68–7.63 (m, 3H), 7.53 (t, *J* = 7.6 Hz, 2H), 7.39 (t, *J* = 7.6 Hz, 1H); ^{13}C NMR (101 MHz, $CDCl_3$) δ 147.6, 142.8, 134.0, 132.1, 131.8, 130.3, 129.4, 129.0, 128.9, 127.5, 127.4, 127.1, 113.5; HRMS (ESI) *m*/*z* calcd for $C_{17}H_{13}N_4$ $[M+H]^+$: 273.1135 found 273.1133.

3-5. Preparation of 1AP

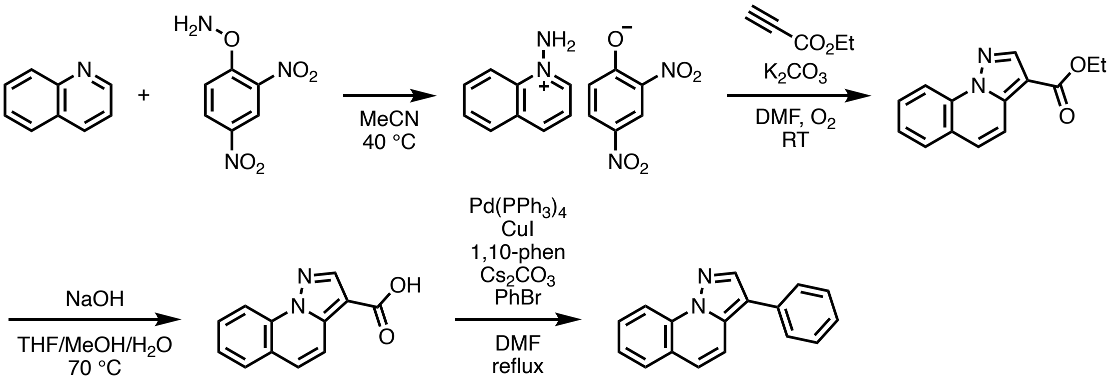

1AP

To a round-bottom flask containing a magnetic stirring bar were added quinoline (646 mg, 5.00 mmol, 1.0 equiv) and MeCN (4.0 mL). To this mixture was added *O*-(2,4-dinitrophenyl)hydroxylamine[10] (996 mg, 5.00 mmol, 1.0 equiv) in one portion. The mixture was stirred at 40 °C for 24 h. The solvent was removed under reduced pressure and the residue was triturated with Et_2O. The resulting solid was filtered and dried *in vacuo*. The obtained solid containing *N*-aminoquinolinium salt was used for the next step without further purification.

To a round-bottom flask containing a magnetic stirring bar and a solution of the crude material in DMF (2.0 mL) were added potassium carbonate (967 mg, 7.00 mmol, 1.4 equiv) and ethyl propiolate (562 μL, 5.50 mmol, 1.1 equiv) at room temperature. Air was introduced to the reaction mixture by diaphragm

pump. The reaction mixture was stirred for 24 h. The solvent was removed *in vacuo*, and the mixture was extracted with Et_2O. The combined organic layer was dried over $MgSO_4$, filtered, and concentrated *in vacuo*. The residue was purified by Isolera® (hexane/EtOAc = 19:1 to 4:1) to afford ethyl pyrazolo[1,5-*a*]quinoline-3-carboxylate (420 mg, 35% yield over two steps) as a pale yellow solid.

To a round-bottom flask containing a magnetic stirring bar and ethyl pyrazolo[1,5-*a*]quinoline-3-carboxylate (365 mg, 1.52 mmol, 1.0 equiv) were added THF (1.7 mL), MeOH (1.7 mL), H_2O (1.7 mL), and 6.0 M NaOH aq. (500 µL, 3.04 mmol, 2.0 equiv) at room temperature. The reaction mixture was stirred at 70 °C. After completion of the reaction, the mixture was adjusted to pH = 7 with DOWEX® 50W. The mixture was filtered and washed with MeOH. The filtrate was concentrated *in vacuo* to afford pyrazolo[1,5-*a*]quinoline-3-carboxylic acid. The crude material was used for the next step without further purification.

A round-bottom flask containing a magnetic stirring bar and cesium carbonate (744 mg, 2.28 mmol, 1.5 equiv) was dried with a heat-gun *in vacuo* and filled with N_2 gas after cooling to room temperature. To this flask were added $Pd(PPh_3)_4$ (87.8 mg, 76.0 µmol, 5.0 mol%), copper iodide (28.9 mg, 1.52 mmol, 10 mol%), 1,10-phenanthroline (27.3 mg, 1.52 mmol, 10 mol%), and the crude material containing pyrazolo[1,5-*a*]quinoline-3-carboxylic acid. The flask was placed under vacuum and refilled with N_2 gas three times. To this flask were added bromobenzene (160 µL, 1.52 mmol, 1.0 equiv) and DMF (7.1 mL). The mixture was heated under reflux and stirred for 18 h. After cooled to room temperature, the mixture was passed through a short silica-gel pad with EtOAc as an eluent, and then concentrated *in vacuo*. The residue was purified by Isolera® (hexane/EtOAc = 49:1 to 19:1) to afford 3-phenylpyrazolo[1,5-*a*]quinoline (**1AP**: 87.9 mg, 24% yield over two steps) as a pale yellow solid. ^1H NMR (400 MHz, $CDCl_3$) δ 8.61 (d, J = 8.8 Hz, 1H), 8.20 (s, 1H), 7.78 (d, J = 7.8 Hz, 1H), 7.72 (d, J = 8.8 Hz, 1H), 7.70 (td, J = 8.8, 1.2 Hz, 1H), 7.63 (d, J = 6.8 Hz, 2H), 7.50–7.45 (m, 4H), 7.34 (t, J = 7.2 Hz, 1H); ^{13}C NMR (101 MHz, $CDCl_3$) δ 139.9, 134.7, 133.0, 129.6, 129.0, 128.3, 127.5, 126.6, 125.1, 124.9, 123.4, 115.93, 115.86, 115.5 (one peak is missing due to overlapping); HRMS (ESI) *m*/*z* calcd for $C_{17}H_{13}N_2$ $[M+H]^+$: 245.1073 found 245.1071.

3-6. Synthesis of 1AQ

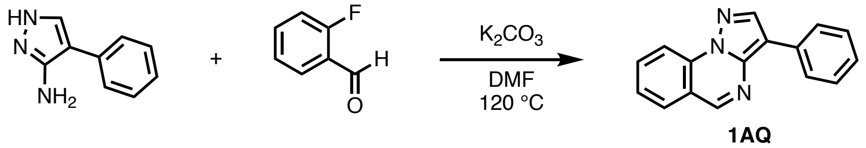

To a round-bottom flask containing a magnetic stirring bar were added 4-phenyl-1*H*-pyrazol-3-amine[13] (477 mg, 3.00 mmol, 1.0 equiv), 2-fluorobenzaldehyde (372 mg, 3.00 mmol, 1.0 equiv), potassium carbonate (1.24 g, 9.00 mmol, 3.0 equiv), and DMF (10 mL) at room temperature. The mixture was stirred at 120 °C for 24 h. After cooling the mixture to room temperature, H_2O was added. The mixture was extracted with Et_2O and washed with brine. The combined organic layer was dried

over $MgSO_4$, filtered, and concentrated *in vacuo*. The residue was purified by Isolera® (hexane/EtOAc = 19:1 to 4:1) to afford 3-phenylpyrazolo[1,5-*a*]quinazoline (**1AQ**: 147 mg, 20% yield) as a yellow solid. ^{1}H NMR (400 MHz, $CDCl_3$) δ 8.94 (s, 1H), 8.48 (d, J = 8.4 Hz, 1H), 8.40 (s, 1H), 8.06 (dd, J = 8.4, 1.2 Hz, 2H), 7.96 (dd, J = 8.0, 0.8 Hz, 1H), 7.91 (ddd, J = 8.4, 7.2, 1.6 Hz, 1H), 7.56 (ddd, J = 8.4, 7.2, 1.6 Hz, 1H), 7.48 (dd, J = 8.4, 7.2 Hz, 2H), 7.31 (t, J = 7.2 Hz, 1H); ^{13}C NMR (101 MHz, $CDCl_3$) δ 151.6, 142.0, 140.7, 136.4, 134.2, 131.9, 128.8, 128.3, 126.7, 126.6, 125.4, 118.7, 114.7, 113.7; HRMS (ESI) *m*/*z* calcd for $C_{16}H_{12}N_3$ [M+H]$^+$: 246.1025 found 246.1025.

4. Ring-Opening Fluorination of Bicyclic Azaarenes

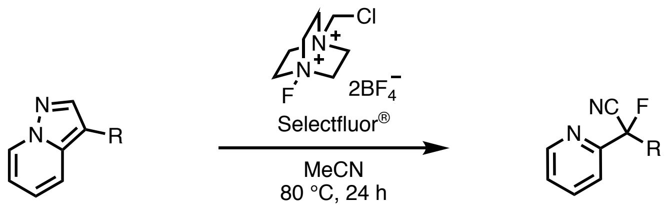

General Procedure

An 8-mL glass tube equipped with a screw cap containing a magnetic stirring bar was dried with a heat-gun *in vacuo* and filled with N_2 gas after cooling to room temperature. To this tube were added azaarenes **1** (0.200 mmol, 1.0 equiv) and Selectfluor® (70.8 mg, 0.200 mmol, 1.0 equiv). The tube was placed under vacuum and refilled with N_2 gas three times. To this tube was added MeCN (1.0 mL). The tube was sealed with a screw cap and then heated at 80 °C for 24 h with stirring. After cooling the reaction mixture to room temperature, the mixture was concentrated *in vacuo*. The residue was purified to afford the corresponding fluorinated compound **2**.

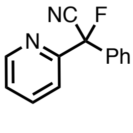

2-Fluoro-2-phenyl-2-(pyridin-2-yl)acetonitrile (2A)

Purification by PTLC (hexane/EtOAc = 4:1) afforded **2A** (41.2 mg, 97% yield) as a colorless solid. ^{1}H NMR (400 MHz, $CDCl_3$) δ 8.67 (dt, J = 4.4, 0.8 Hz, 1H), 7.82 (td, J = 7.6, 1.2 Hz, 1H), 7.60 (dd, J = 7.6, 1.2 Hz, 1H), 7.58–7.53 (m, 2H), 7.46–7.40 (m, 3H), 7.38–7.32 (m, 1H); ^{13}C NMR (101 MHz, $CDCl_3$) δ 155.1 (d, $J_{C–F}$ = 28.9 Hz), 149.7, 137.6, 135.8 (d, $J_{C–F}$ = 24.1 Hz), 130.2, 128.9, 125.8 (d, $J_{C–F}$ = 5.9 Hz), 124.5, 119.6 (d, $J_{C–F}$ = 5.9 Hz), 116.7 (d, $J_{C–F}$ = 33.7 Hz), 92.0 (d, $J_{C–F}$ = 186.9 Hz); ^{19}F NMR (376 MHz, $CDCl_3$) δ –142.2; HRMS (ESI) *m*/*z* calcd for $C_{13}H_{10}FN_2$ [M+H]$^+$: 213.0823 found 213.0821.

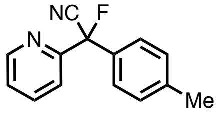

2-Fluoro-2-(pyridin-2-yl)-2-(*p*-tolyl)acetonitrile (2B)

Purification by PTLC (hexane/EtOAc = 4:1) afforded **2B** (30.8 mg, 68% yield) as a colorless oil. ^{1}H NMR (400 MHz, $CDCl_3$) δ 8.67 (d, *J* = 4.8 Hz, 1H), 7.82 (td, *J* = 8.0, 1.2 Hz, 1H), 7.61 (dd, *J* = 7.6, 1.2 Hz, 1H), 7.43 (d, *J* = 8.0 Hz, 2H), 7.35 (ddd, *J* = 7.6, 4.8, 1.2 Hz, 1H), 7.23 (d, *J* = 8.0 Hz, 2H), 2.36 (s, 3H); ^{13}C NMR (101 MHz, $CDCl_3$) δ 155.4 (d, $J_{C–F}$ = 28.9 Hz), 149.7, 140.5, 137.5, 133.0 (d, $J_{C–F}$ = 24.0 Hz), 129.6, 125.9 (d, $J_{C–F}$ = 5.8 Hz), 124.4, 119.7 (d, $J_{C–F}$ = 4.8 Hz), 116.8 (d, $J_{C–F}$ = 34.6 Hz), 92.0 (d, $J_{C–F}$ = 185.8 Hz), 21.2; ^{19}F NMR (376 MHz, $CDCl_3$) δ –140.3; HRMS (ESI) *m*/*z* calcd for $C_{14}H_{11}FN_2Na$ $[M+Na]^+$: 249.0799 found 249.0798.

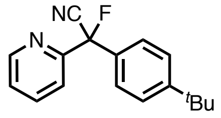

2-(4-(*tert*-Butyl)phenyl)-2-fluoro-2-(pyridin-2-yl)acetonitrile (2C)

Purification by PTLC (hexane/EtOAc = 4:1) afforded **2C** (31.1 mg, 58% yield) as a yellow oil. ^{1}H NMR (400 MHz, $CDCl_3$) δ 8.68 (d, *J* = 5.2 Hz, 1H), 7.82 (td, *J* = 7.6, 1.6 Hz, 1H), 7.62 (dd, *J* = 7.6, 1.2 Hz, 1H), 7.49–7.41 (m, 4H), 7.35 (ddd, *J* = 7.6, 5.2, 0.8 Hz, 1H), 1.30 (s, 9H); ^{13}C NMR (101 MHz, $CDCl_3$) δ 155.3 (d, $J_{C–F}$ = 29.1 Hz), 153.5, 149.7, 137.5, 132.9 (d, $J_{C–F}$ = 24.2 Hz), 125.9, 125.7 (d, $J_{C–F}$ = 4.8 Hz), 124.4, 119.7 (d, $J_{C–F}$ = 4.8 Hz), 116.8 (d, $J_{C–F}$ = 33.0 Hz), 92.0 (d, $J_{C–F}$ = 185.2 Hz), 34.7, 31.1; ^{19}F NMR (376 MHz, $CDCl_3$) δ –140.3; HRMS (ESI) *m*/*z* calcd for $C_{17}H_{17}FN_2Na$ $[M+Na]^+$: 291.1268 found 291.1266.

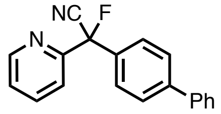

2-([1,1'-Biphenyl]-4-yl)-2-fluoro-2-(pyridin-2-yl)acetonitrile (2D)

Purification by PTLC (hexane/EtOAc = 4:1) afforded **2D** (32.9 mg, 57% yield) as a white solid. ^{1}H NMR (400 MHz, $CDCl_3$) δ 8.70 (dd, *J* = 4.8, 1.2 Hz, 1H), 7.85 (td, *J* = 7.6, 1.6 Hz, 1H), 7.67–7.61 (m, 5H), 7.58–7.55 (m, 2H), 7.47–7.43 (m, 2H), 7.39–7.36 (m, 2H); ^{13}C NMR (101 MHz, $CDCl_3$) δ 155.2 (d, $J_{C–F}$ = 29.1 Hz), 149.8, 143.2, 139.8, 137.6, 134.6 (d, $J_{C–F}$ = 23.2 Hz), 128.9, 128.0, 127.7, 127.2, 126.4 (d, $J_{C–F}$ = 5.8 Hz), 124.6, 119.7 (d, $J_{C–F}$ = 5.8 Hz), 116.7 (d, $J_{C–F}$ = 34.9 Hz), 91.9 (d, $J_{C–F}$ = 187.2 Hz); ^{19}F NMR (376 MHz, $CDCl_3$) δ –141.6; HRMS (ESI) *m*/*z* calcd for $C_{19}H_{14}FN_2$ $[M+H]^+$: 289.1136 found 289.1133.

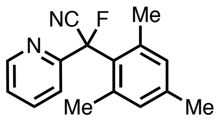

2-Fluoro-2-mesityl-2-(pyridin-2-yl)acetonitrile (2E)

Purification by PTLC (hexane/EtOAc = 4:1) afforded **2E** (20.9 mg, 41% yield) as a yellow solid. ^{1}H NMR (400 MHz, $CDCl_3$) δ 8.73 (d, J = 4.4 Hz, 1H), 7.76 (t, J = 7.6 Hz, 1H), 7.41–7.35 (m, 2H), 6.90 (s, 2H), 2.30–2.26 (m, 9H); ^{13}C NMR (101 MHz, $CDCl_3$) δ 155.4 (d, $J_{C–F}$ = 23.1 Hz), 149.8, 139.2, 137.6, 136.3, 131.7, 128.6 (d, $J_{C–F}$ = 21.2 Hz), 124.9 (d, $J_{C–F}$ = 2.8 Hz), 121.6 (d, $J_{C–F}$ = 2.9 Hz), 116.6 (d, $J_{C–F}$ = 35.7 Hz), 94.8 (d, $J_{C–F}$ = 182.0 Hz), 22.5 (d, $J_{C–F}$ = 7.8 Hz), 20.7; ^{19}F NMR (376 MHz, $CDCl_3$) δ –128.4; HRMS (ESI) *m*/*z* calcd for $C_{16}H_{16}FN_2$ $[M+H]^+$: 255.1292 found 255.1291.

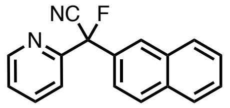

2-Fluoro-2-(naphthalen-2-yl)-2-(pyridin-2-yl)acetonitrile (2F)

Purification by PTLC (hexane/EtOAc = 4:1) and GPC afforded **2F** (28.3 mg, 54% yield) as a white solid. ^{1}H NMR (400 MHz, $CDCl_3$) δ 8.69 (d, J = 4.4 Hz, 1H), 8.13 (s, 1H), 7.93–7.80 (m, 4H), 7.67 (dd, J = 7.6, 1.2 Hz, 1H), 7.57–7.53 (m, 3H), 7.37 (dd, J = 7.6, 4.8 Hz, 1H); ^{13}C NMR (101 MHz, $CDCl_3$) δ 155.2 (d, $J_{C–F}$ = 29.0 Hz), 149.8, 137.6, 133.7, 132.9 (d, $J_{C–F}$ = 24.1 Hz), 132.6, 129.1, 128.7, 127.7, 127.6, 127.0, 125.8 (d, $J_{C–F}$ = 6.7 Hz), 124.6, 122.4 (d, $J_{C–F}$ = 4.8 Hz), 119.8 (d, $J_{C–F}$ = 5.9 Hz), 116.7 (d, $J_{C–F}$ = 32.4 Hz), 92.3 (d, $J_{C–F}$ = 185.8 Hz); ^{19}F NMR (376 MHz, $CDCl_3$) δ –141.9; HRMS (ESI) *m*/*z* calcd for $C_{17}H_{11}FN_2Na$ $[M+Na]^+$: 285.0799 found 285.0798.

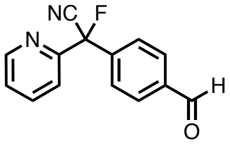

2-Fluoro-2-(4-formylphenyl)-2-(pyridin-2-yl)acetonitrile (2G)

Purification by PTLC (hexane/EtOAc = 4:1) afforded **2G** (38.4 mg, 80% yield) as a white solid. ^{1}H NMR (400 MHz, $CDCl_3$) δ 10.1 (s, 1H), 8.67 (dd, J = 4.0, 0.8 Hz, 1H), 7.96 (d, J = 8.0 Hz, 2H), 7.85 (td, J = 8.0, 1.6 Hz, 1H), 7.79 (d, J = 8.0 Hz, 2H), 7.65 (dd, J = 8.0, 0.8 Hz, 1H), 7.39 (dd, J = 8.0, 4.8 Hz, 1H); ^{13}C NMR (101 MHz, $CDCl_3$) δ 191.2, 154.4 (d, $J_{C–F}$ = 28.9 Hz), 149.9, 141.5 (d, $J_{C–F}$ = 24.0 Hz), 137.8, 137.3, 130.1, 126.4 (d, $J_{C–F}$ = 5.9 Hz), 124.9, 119.5 (d, $J_{C–F}$ = 5.8 Hz), 116.1 (d, $J_{C–F}$ = 32.7 Hz), 91.4 (d, $J_{C–F}$ = 187.9 Hz); ^{19}F NMR (376 MHz, $CDCl_3$) δ –146.2; HRMS (ESI) *m*/*z* calcd for $C_{14}H_{10}FN_2O$ $[M+H]^+$: 241.0772 found 241.0770.

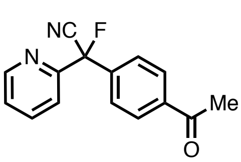

2-(4-Acetylphenyl)-2-fluoro-2-(pyridin-2-yl)acetonitrile (2H)

Purification by filtration with chloroform afforded **2H** (50.8 mg, quant.) as a yellow oil. ^{1}H NMR (400 MHz, $CDCl_3$) δ 8.67 (d, J = 4.4 Hz, 1H), 8.01 (d, J = 8.4 Hz, 2H), 7.85 (td, J = 7.6, 2.0 Hz, 1H), 7.70

(d, J = 8.4 Hz, 2H), 7.63 (d, J = 7.6 Hz, 1H), 7.38 (dd, J = 7.6, 4.4 Hz, 1H), 2.61 (s, 3H); ^{13}C NMR (101 MHz, $CDCl_3$) δ 197.0, 154.6 (d, $J_{C–F}$ = 28.9 Hz), 149.8, 140.2 (d, $J_{C–F}$ = 24.0 Hz), 138.2, 137.8, 128.8, 126.0 (d, $J_{C–F}$ = 5.8 Hz), 124.8, 119.5 (d, $J_{C–F}$ = 5.8 Hz), 116.2 (d, $J_{C–F}$ = 32.7 Hz), 91.5 (d, $J_{C–F}$ = 187.8 Hz), 26.7; ^{19}F NMR (376 MHz, $CDCl_3$) δ –145.7; HRMS (ESI) *m*/*z* calcd for $C_{15}H_{12}FN_2O$ $[M+H]^+$: 255.0928 found 255.0927.

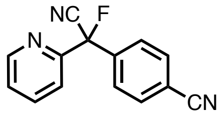

4-(Cyanofluoro(pyridin-2-yl)methyl)benzonitrile (2I)

Purification by filtration with chloroform afforded **2I** (45.6 mg, 96% yield) as a yellow solid. ^{1}H NMR (400 MHz, $CDCl_3$) δ 8.66 (dt, J = 4.8, 1.2 Hz, 1H), 7.86 (td, J = 8.0, 1.6 Hz, 1H), 7.76–7.73 (m, 4H), 7.65–7.63 (m, 1H), 7.40 (dd, J = 8.0, 1.2 Hz, 1H); ^{13}C NMR (101 MHz, $CDCl_3$) δ 154.0 (d, $J_{C–F}$ = 29.1 Hz), 149.9, 140.3 (d, $J_{C–F}$ = 24.2 Hz), 137.9, 132.6, 126.4 (d, $J_{C–F}$ = 6.8 Hz), 125.0, 119.4 (d, $J_{C–F}$ = 5.8 Hz), 117.7, 115.8 (d, $J_{C–F}$ = 33.0 Hz), 114.2, 91.0 (d, $J_{C–F}$ = 189.1 Hz); ^{19}F NMR (376 MHz, $CDCl_3$) δ –147.5; HRMS (ESI) *m*/*z* calcd for $C_{14}H_9FN_3$ $[M+H]^+$: 238.0775 found 238.0776.

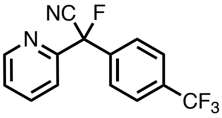

2-Fluoro-2-(pyridin-2-yl)-2-(4-(trifluoromethyl)phenyl)acetonitrile (2J)

Purification by PTLC (hexane/EtOAc = 4:1) afforded **2J** (46.0 mg, quant.) as a colorless oil. ^{1}H NMR (400 MHz, $CDCl_3$) δ 8.68 (d, J = 5.2 Hz, 1H), 7.85 (td, J = 7.6, 1.2 Hz, 1H), 7.74 (d, J = 8.8 Hz, 2H), 7.71 (d, J = 8.8 Hz, 2H), 7.64 (d, J = 7.6 Hz, 1H), 7.39 (dd, J = 8.0, 5.2 Hz, 1H); ^{13}C NMR (101 MHz, $CDCl_3$) δ 154.4 (d, $J_{C–F}$ = 28.2 Hz), 149.9, 139.5 (d, $J_{C–F}$ = 24.0 Hz), 137.8, 132.3 (q, $J_{C–F}$ = 32.8 Hz), 126.2 (d, $J_{C–F}$ = 5.8 Hz), 126.0 (q, $J_{C–F}$ = 3.8 Hz), 124.9, 123.5 (q, $J_{C–F}$ = 273.9 Hz), 119.5 (d, $J_{C–F}$ = 5.9 Hz), 116.1 (d, $J_{C–F}$ = 32.8 Hz), 91.3 (d, $J_{C–F}$ = 188.8 Hz); ^{19}F NMR (376 MHz, $CDCl_3$) δ –63.0, –146.1; HRMS (ESI) *m*/*z* calcd for $C_{14}H_9F_4N_2$ $[M+H]^+$: 281.0696 found 281.0695.

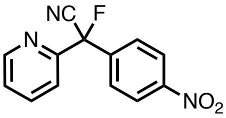

2-Fluoro-2-(4-nitrophenyl)-2-(pyridin-2-yl)acetonitrile (2K)

Purification by filtration with chloroform afforded **2K** (51.4 mg, quant.) as a yellow oil. ^{1}H NMR (400 MHz, $CDCl_3$) δ 8.67 (dd, J = 4.8, 1.2 Hz, 1H), 8.30 (d, J = 9.2 Hz, 2H), 7.87 (td, J = 7.6, 1.2 Hz, 1H), 7.83 (d, J = 9.2 Hz, 2H), 7.67 (dd, J = 7.6, 0.8 Hz, 1H), 7.40 (dd, J = 7.6, 4.8 Hz, 1H); ^{13}C NMR (101 MHz, $CDCl_3$) δ 154.0 (d, $J_{C–F}$ = 28.1 Hz), 150.0, 148.8, 142.0 (d, $J_{C–F}$ = 24.2 Hz), 138.0, 126.8 (d, $J_{C–F}$

= 6.9 Hz), 125.0, 124.1, 119.4 (d, $J_{C–F}$ = 5.8 Hz), 115.8 (d, $J_{C–F}$ = 32.9 Hz), 90.9 (d, $J_{C–F}$ = 189.1 Hz); ^{19}F NMR (376 MHz, $CDCl_3$) δ –147.5; HRMS (ESI) *m*/*z* calcd for $C_{13}H_9FN_3O_2$ [M+H]$^+$: 258.0673 found 258.0672.

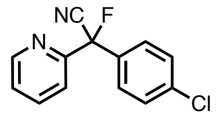

2-(4-Chlorophenyl)-2-fluoro-2-(pyridin-2-yl)acetonitrile (2L)

Purification by filtration with chloroform afforded **2L** (49.3 mg, quant.) as a yellow oil. ^{1}H NMR (400 MHz, $CDCl_3$) δ 8.67 (d, *J* = 4.8 Hz, 1H), 7.84 (td, *J* = 7.6, 1.2 Hz, 1H), 7.61 (d, *J* = 8.0 Hz, 1H), 7.51 (d, *J* = 8.8 Hz, 2H), 7.41 (d, *J* = 8.8 Hz, 2H), 7.38 (dd, *J* = 7.6, 4.8 Hz, 1H); ^{13}C NMR (101 MHz, $CDCl_3$) δ 154.7 (d, $J_{C–F}$ = 29.1 Hz), 149.8, 137.7, 136.5, 134.4 (d, $J_{C–F}$ = 24.2 Hz), 129.2, 127.3 (d, $J_{C–F}$ = 5.9 Hz), 124.7, 119.5 (d, $J_{C–F}$ = 5.9 Hz), 116.3 (d, $J_{C–F}$ = 32.9 Hz), 91.4 (d, $J_{C–F}$ = 188.2 Hz); ^{19}F NMR (376 MHz, $CDCl_3$) δ –143.0; HRMS (ESI) *m*/*z* calcd for $C_{13}H_9ClFN_2$ [M+H]$^+$: 247.0433 found 247.0432.

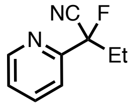

2-Fluoro-2-(pyridin-2-yl)butanenitrile (2M)

Purification by filtration with chloroform afforded **2M** (26.3 mg, 80% yield) as a colorless oil. ^{1}H NMR (400 MHz, $CDCl_3$) δ 8.67 (dd, *J* = 4.8, 0.8 Hz, 1H), 7.83 (td, *J* = 8.0, 0.8 Hz, 1H), 7.57 (d, *J* = 8.0 Hz, 1H), 7.36 (dd, *J* = 8.0, 4.8 Hz, 1H), 2.49–2.27 (m, 2H), 1.13 (t, *J* = 7.2 Hz, 3H); ^{13}C NMR (101 MHz, $CDCl_3$) δ 154.6 (d, $J_{C–F}$ = 27.0 Hz), 149.6, 137.4, 124.4, 119.4 (d, $J_{C–F}$ = 6.8 Hz), 116.8 (d, $J_{C–F}$ = 33.7 Hz), 92.7 (d, $J_{C–F}$ = 185.9 Hz), 33.2 (d, $J_{C–F}$ = 23.1 Hz), 7.7 (d, $J_{C–F}$ = 3.8 Hz); ^{19}F NMR (376 MHz, $CDCl_3$) δ –157.1 (t, $J_{F–H}$ = 20.9 Hz); HRMS (ESI) *m*/*z* calcd for $C_9H_{10}FN_2$ [M+H]$^+$: 165.0823 found 165.0823.

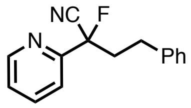

2-Fluoro-4-phenyl-2-(pyridin-2-yl)butanenitrile (2N)

Purification by PTLC (hexane/EtOAc = 4:1) afforded **2N** (44.7 mg, 93% yield) as a yellow oil. ^{1}H NMR (400 MHz, $CDCl_3$) δ 8.67 (d, J = 5.2 Hz, 1H), 7.83 (t, J = 8.0 Hz, 1H), 7.61 (d, J = 8.0 Hz, 1H), 7.37 (dd, J = 8.0, 5.2 Hz, 3H), 7.31–7.24 (m, 2H), 7.23–7.15 (m, 3H), 3.01–2.78 (m, 2H), 2.78–2.50 (m, 2H); ^{13}C NMR (101 MHz, $CDCl_3$) δ 154.4 (d, $J_{C–F}$ = 27.0 Hz), 149.7, 139.4, 137.5, 128.6, 128.3, 126.4, 124.6, 119.4 (d, $J_{C–F}$ = 6.8 Hz), 116.7 (d, $J_{C–F}$ = 33.6 Hz), 91.7 (d, $J_{C–F}$ = 185.9 Hz), 41.4 (d, $J_{C–F}$ = 23.0 Hz), 29.8 (d, $J_{C–F}$ = 2.8 Hz); ^{19}F NMR (376 MHz, $CDCl_3$) δ –156.0 (t, $J_{F–H}$ = 24.1 Hz); HRMS (ESI) *m*/*z* calcd for $C_{15}H_{14}FN_2$ $[M+H]^+$: 241.1136 found 241.1134.

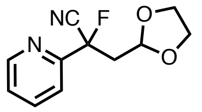

3-(1,3-Dioxolan-2-yl)-2-fluoro-2-(pyridin-2-yl)propanenitrile (2O)

Purification by PTLC (hexane/EtOAc = 4:1) afforded **2O** (20.4 mg, 46% yield) as a yellow oil. ^{1}H NMR (400 MHz, $CDCl_3$) δ 8.67 (dd, J = 4.8, 1.6 Hz, 1H), 7.84 (td, J = 7.6, 1.6 Hz, 1H), 7.64 (dd, J = 7.6, 1.2 Hz, 1H), 7.38 (dd, J = 7.6, 4.8 Hz, 1H), 5.20 (t, J = 4.8 Hz, 1H), 4.04–3.97 (m, 2H), 3.92–3.84 (m, 2H), 2.88–2.64 (m, 2H); ^{13}C NMR (101 MHz, $CDCl_3$) δ 154.2 (d, $J_{C–F}$ = 27.0 Hz), 149.6, 137.6, 124.6, 119.4 (d, $J_{C–F}$ = 6.8 Hz), 116.3 (d, $J_{C–F}$ = 33.6 Hz), 100.1 (d, $J_{C–F}$ = 2.9 Hz), 89.1 (d, $J_{C–F}$ = 185.8 Hz), 65.0 (d, $J_{C–F}$ = 3.9 Hz), 43.2 (d, $J_{C–F}$ = 22.1 Hz); ^{19}F NMR (376 MHz, $CDCl_3$) δ –153.7 (dd, $J_{F–H}$ = 29.0, 17.3 Hz); HRMS (ESI) *m*/*z* calcd for $C_{11}H_{12}FN_2O_2$ $[M+H]^+$: 223.0877 found 223.0878.

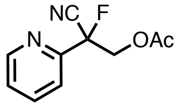

2-Cyano-2-fluoro-2-(pyridin-2-yl)ethyl acetate (2P)

Purification by PTLC (hexane/EtOAc = 4:1) afforded **2P** (37.5 mg, 90% yield) as a colorless oil. ^{1}H NMR (400 MHz, $CDCl_3$) δ 8.68 (dd, J = 4.8, 1.2 Hz, 1H), 7.87 (td, J = 7.6, 1.2 Hz, 1H), 7.65 (d, J = 7.6 Hz, 1H), 7.43 (dd, J = 7.6, 4.8 Hz, 1H), 4.79 (dd, $J_{H–F}$ = 44.8 Hz, J = 12.8 Hz, 1H), 4.74 (dd, $J_{H–F}$ = 34.4 Hz, J = 12.8 Hz, 1H), 2.16 (s, 3H); ^{13}C NMR (101 MHz, $CDCl_3$) δ 169.7, 151.2 (d, $J_{C–F}$ = 26.1 Hz), 149.8, 137.7, 125.2, 120.2 (d, $J_{C–F}$ = 5.8 Hz), 114.8 (d, $J_{C–F}$ = 33.7 Hz), 89.7 (d, $J_{C–F}$ = 190.7 Hz), 66.2 (d, $J_{C–F}$ = 23.0 Hz), 20.5; ^{19}F NMR (376 MHz, $CDCl_3$) δ –160.8 (dd, $J_{F–H}$ = 25.8, 14.5 Hz); HRMS (ESI) *m*/*z* calcd for $C_{10}H_9FN_2O_2$ $[M+H]^+$: 209.0721 found 209.0720.

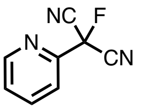

2-Fluoro-2-(pyridin-2-yl)malononitrile (2Q)

Purification by filtration afforded **2S** (27.4 mg, 85% yield) as a yellow oil. ^{1}H NMR (400 MHz, $CDCl_3$) δ 8.80 (d, J = 4.8 Hz, 1H), 7.99 (td, J = 7.6, 1.6 Hz, 1H), 7.79 (d, J = 7.6 Hz, 1H), 7.61–7.56 (m, 1H); ^{13}C NMR (101 MHz, $CDCl_3$) δ 150.7, 148.0 (d, $J_{C–F}$ = 25.0 Hz), 138.6, 127.0, 120.8 (d, $J_{C–F}$ = 2.9 Hz), 110.6 (d, $J_{C–F}$ = 35.6 Hz), 78.7 (d, $J_{C–F}$ = 196.4 Hz); ^{19}F NMR (376 MHz, $CDCl_3$) δ –138.0.

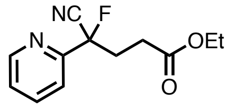

Ethyl 4-cyano-4-fluoro-4-(pyridin-2-yl)butanoate (2R)

Purification by filtration with chloroform afforded **2R** (41.6 mg, 88% yield) as a colorless oil. ^{1}H NMR (400 MHz, $CDCl_3$) δ 8.67 (d, J = 5.6 Hz, 1H), 7.84 (td, J = 7.6, 1.6 Hz, 1H), 7.58 (d, J = 7.6 Hz, 1H), 7.38 (dd, J = 7.6, 5.6 Hz, 1H), 4.14 (q, J = 7.2 Hz, 2H), 2.83–2.53 (m, 4H), 1.26 (t, J = 7.2 Hz, 3H); ^{13}C NMR (101 MHz, $CDCl_3$) δ 171.3, 153.9 (d, $J_{C–F}$ = 26.1 Hz), 149.7, 137.6, 124.7, 119.4 (d, $J_{C–F}$ = 6.8 Hz), 116.3 (d, $J_{C–F}$ = 33.7 Hz), 91.2 (d, $J_{C–F}$ = 186.9 Hz), 60.9, 34.8 (d, $J_{C–F}$ = 23.1 Hz), 28.5 (d, $J_{C–F}$ = 3.8 Hz), 14.1; ^{19}F NMR (376 MHz, $CDCl_3$) δ –156.3 (t, $J_{F–H}$ = 20.4 Hz); HRMS (ESI) *m*/*z* calcd for $C_{12}H_{13}FN_2O_2Na$ $[M+Na]^+$: 259.0853 found 259.0852.

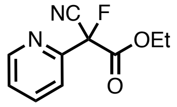

Ethyl 2-cyano-2-fluoro-2-(pyridin-2-yl)acetate (2S)

Purification by filtration with chloroform afforded **2S** (40.4 mg, 97% yield) as a colorless oil. ^{1}H NMR (400 MHz, $CDCl_3$) δ 8.68 (d, J = 5.2 Hz, 1H), 7.91 (td, J = 7.6, 1.6 Hz, 1H), 7.73 (d, J = 7.6 Hz, 1H), 7.48 (dd, J = 7.6, 5.2 Hz, 1H), 4.50–4.30 (m, 2H), 1.33 (t, J = 6.8 Hz, 3H); ^{13}C NMR (101 MHz, $CDCl_3$) δ 162.0 (d, $J_{C–F}$ = 28.0 Hz), 150.6 (d, $J_{C–F}$ = 26.2 Hz), 149.8, 137.9, 125.7, 120.8 (d, $J_{C–F}$ = 3.8 Hz), 113.5 (d, $J_{C–F}$ = 32.9 Hz), 87.5 (d, $J_{C–F}$ = 199.4 Hz), 64.4, 13.7; ^{19}F NMR (376 MHz, $CDCl_3$) δ –148.0; HRMS (ESI) *m*/*z* calcd for $C_{10}H_9FN_2O_2Na$ $[M+Na]^+$: 231.0540 found 231.0539.

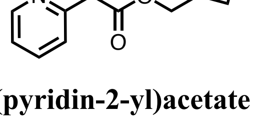

Cyclobutylmethyl 2-cyano-2-fluoro-2-(pyridin-2-yl)acetate (2T)

Purification by filtration with chloroform afforded **2T** (46.2 mg, 93% yield) as a yellow oil. ^{1}H NMR (400 MHz, $CDCl_3$) δ 8.68 (d, J = 4.4 Hz, 1H), 7.89 (td, J = 8.0, 1.2 Hz, 1H), 7.73 (d, J = 8.0 Hz, 1H),

7.51–7.40 (m, 1H), 4.31 (d, J = 6.4 Hz, 2H), 2.73–2.60 (m, 1H), 2.09–1.66 (m, 6H); ^{13}C NMR (101 MHz, $CDCl_3$) δ 162.2 (d, $J_{C–F}$ = 27.9 Hz), 150.8 (d, $J_{C–F}$ = 25.0 Hz), 149.9, 137.9, 125.7, 120.9 (d, $J_{C–F}$ = 3.8 Hz), 113.5 (d, $J_{C–F}$ = 33.6 Hz), 87.6 (d, $J_{C–F}$ = 197.3 Hz), 71.5, 33.6, 24.3, 18.2; ^{19}F NMR (376 MHz, $CDCl_3$) δ –148.0; HRMS (ESI) *m/z* calcd for $C_{13}H_{14}FN_2O_2$ $[M+H]^+$: 249.1034 found 249.1033.

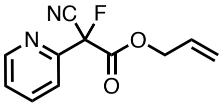

Allyl 2-cyano-2-fluoro-2-(pyridin-2-yl)acetate (2U)

Purification by PTLC (hexane/EtOAc = 8:1) afforded **2U** (39.6 mg, 90% yield) as a colorless oil. ^{1}H NMR (400 MHz, $CDCl_3$) δ 8.70 (d, J = 4.8 Hz, 1H), 7.89 (td, J = 7.6, 2.0 Hz, 1H), 7.27 (dt, J = 7.6, 1.2 Hz, 1H), 7.48–7.44 (m, 1H), 5.95–5.84 (m, 1H), 5.40–5.28 (m, 2H), 4.88–4.77 (m, 2H); ^{13}C NMR (101 MHz, $CDCl_3$) δ 161.8 (d, $J_{C–F}$ = 28.9 Hz), 150.6 (d, $J_{C–F}$ = 26.1 Hz), 149.9, 137.9, 129.9, 125.8, 120.9 (d, $J_{C–F}$ = 4.8 Hz), 120.1, 113.4 (d, $J_{C–F}$ = 32.8 Hz), 87.5 (d, $J_{C–F}$ = 197.4 Hz), 68.3; ^{19}F NMR (376 MHz, $CDCl_3$) δ –148.0; HRMS (ESI) *m/z* calcd for $C_{11}H_{10}FN_2O_2$ $[M+H]^+$: 221.0721 found 221.0720.

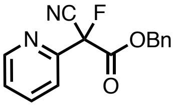

Benzyl 2-cyano-2-fluoro-2-(pyridin-2-yl)acetate (2V)

Purification by filtration with chloroform afforded **2V** (44.9 mg, 83% yield) as a yellow oil. ^{1}H NMR (400 MHz, $CDCl_3$) δ 8.66 (d, J = 4.8 Hz, 1H), 7.86 (td, J = 7.6, 1.2 Hz, 1H), 7.69 (d, J = 8.4 Hz, 1H), 7.46–7.42 (m, 1H), 7.36–7.29 (m, 5H), 5.36 (s, 2H); ^{13}C NMR (101 MHz, $CDCl_3$) δ 161.9 (d, $J_{C–F}$ = 27.9 Hz), 150.5 (d, $J_{C–F}$ = 25.0 Hz), 149.8, 137.9, 133.7, 128.8, 128.6, 128.2, 125.7, 121.0 (d, $J_{C–F}$ = 3.8 Hz), 113.3 (d, $J_{C–F}$ = 32.7 Hz), 87.6 (d, $J_{C–F}$ = 197.4 Hz), 69.5; ^{19}F NMR (376 MHz, $CDCl_3$) δ –148.2; HRMS (ESI) *m/z* calcd for $C_{15}H_{12}FN_2O_2$ $[M+H]^+$: 271.0877 found 271.0877.

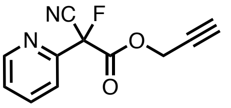

Prop-2-yn-1-yl 2-cyano-2-fluoro-2-(pyridin-2-yl)acetate (2W)

Purification by filtration with chloroform afforded **2W** (43.6 mg, quant.) as a yellow oil. ^{1}H NMR (400 MHz, $CDCl_3$) δ 8.69 (dd, J = 4.8, 1.2 Hz, 1H), 7.90 (td, J = 7.6, 1.2 Hz, 1H), 7.75 (dt, J = 7.6, 0.8 Hz, 1H), 7.50–7.44 (m, 1H), 4.91 (dd, J = 2.0, 0.8 Hz, 2H), 2.56 (t, J = 2.4 Hz, 1H); ^{13}C NMR (101 MHz, $CDCl_3$) δ 161.4 (d, $J_{C–F}$ = 28.9 Hz), 150.3 (d, $J_{C–F}$ = 25.0 Hz), 150.0, 138.0, 125.9, 121.1 (d, $J_{C–F}$ = 3.8 Hz), 113.1 (d, $J_{C–F}$ = 32.7 Hz), 87.4 (d, $J_{C–F}$ = 199.4 Hz), 76.9, 75.3, 55.2; ^{19}F NMR (376 MHz, $CDCl_3$) δ –147.5; HRMS (ESI) *m/z* calcd for $C_{11}H_8FN_2O_2$ $[M+H]^+$: 219.0564 found 219.0564.

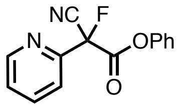

Phenyl 2-cyano-2-fluoro-2-(pyridin-2-yl)acetate (2X)

Purification by filtration with chloroform afforded **2X** (42.5 mg, 83% yield) as a yellow oil. ^{1}H NMR (400 MHz, $CDCl_3$) δ 8.75 (d, J = 4.0 Hz, 1H), 7.95 (td, J = 7.2, 1.6 Hz, 1H), 7.85 (dd, J = 7.2, 0.8 Hz, 1H), 7.53–7.49 (m, 1H), 7.41 (t, J = 8.0 Hz, 2H), 7.30 (t, J = 8.0 Hz, 1H), 7.19 (d, J = 8.0 Hz, 2H); ^{13}C NMR (101 MHz, $CDCl_3$) δ 160.6 (d, $J_{C–F}$ = 28.2 Hz), 150.6 (d, $J_{C–F}$ = 25.3 Hz), 150.1 (d, $J_{C–F}$ = 4.8 Hz), 149.9, 138.2, 129.7, 127.0, 126.0, 121.4 (d, $J_{C–F}$ = 2.8 Hz), 120.7 (d, $J_{C–F}$ = 11.6 Hz), 113.3 (d, $J_{C–F}$ = 33.9 Hz), 87.4 (d, $J_{C–F}$ = 198.8 Hz); ^{19}F NMR (376 MHz, $CDCl_3$) δ –146.5; HRMS (ESI) *m*/*z* calcd for $C_{14}H_{10}FN_2O_2$ $[M+H]^+$: 257.0721 found 257.0719.

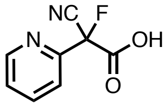

2-Cyano-2-fluoro-2-(pyridin-2-yl)acetic acid (2Y)

The reaction was conducted with Selectfluor® (5.0 equiv) at 50 °C. After the reaction, the solution was concentrated *in vacuo*. To the crude material containing **2Y** was added EtOH (1.0 mL). The mixture was stirred with refluxing for 24 h. After cooling the reaction mixture to room temperature, the reaction was quenched with $NaHCO_3$ aq. The mixture was extracted with EtOAc and washed with brine. The combined organic layer was dried over Na_2SO_4, filtered, and concentrated *in vacuo*. The residue was purified by Isolera® (hexane/EtOAc = 19:1 to 9:1) to afford **2S** (11.8 mg, 31% yield) as a colorless oil.

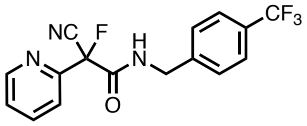

2-Cyano-2-fluoro-2-(pyridin-2-yl)-*N*-(4-(trifluoromethyl)benzyl)acetamide (2Z)

The reaction was performed at –30 °C. Purification by PTLC (chloroform/EtOAc = 10:1) afforded **2Z** (26.3 mg, 39% yield) as a white solid. ^{1}H NMR (400 MHz, $CDCl_3$) δ 8.68 (d, J = 4.4 Hz, 1H), 7.90 (td, J = 8.0, 1.6 Hz, 1H), 7.79 (d, J = 8.0 Hz, 1H), 7.60 (d, J = 8.0 Hz, 2H), 7.48 (ddd, J = 8.0, 4.4, 1.6 Hz, 1H), 7.40 (d, J = 8.0 Hz, 2H), 7.32 (brs, 1H), 4.72–4.53 (m, 2H); ^{13}C NMR (101 MHz, $CDCl_3$) δ 161.9 (d, $J_{C–F}$ = 24.2 Hz), 150.6 (d, $J_{C–F}$ = 24.2 Hz), 150.0, 140.6, 138.1, 130.1 (q, $J_{C–F}$ = 32.9 Hz), 127.8, 125.8, 123.9 (q, $J_{C–F}$ = 273.5 Hz), 114.0 (d, $J_{C–F}$ = 33.0 Hz), 88.1 (d, $J_{C–F}$ = 202.7 Hz), 43.5; ^{19}F NMR (376 MHz, $CDCl_3$) δ –62.7, –150.3; HRMS (ESI) *m*/*z* calcd for $C_{16}H_{12}F_4N_3O$ $[M+H]^+$: 338.0911 found 338.0911.

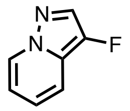

3-Fluoropyrazolo[1,5-*a*]pyridine (2Z')

Purification by PTLC (chloroform/EtOAc = 10:1) afforded **2Z'** as a white solid. ^{1}H NMR (400 MHz, $CDCl_3$) δ 8.28 (dd, *J* = 7.2, 1.2 Hz, 1H), 7.80 (d, $J_{H–F}$ = 3.6 Hz, 1H), 7.51 (dt, *J* = 8.8, 1.2 Hz, 1H), 7.07 (dd, *J* = 8.8, 6.8 Hz, 1H), 6.71 (td, *J* = 6.8, 1.2 Hz, 1H); ^{13}C NMR (101 MHz, $CDCl_3$) δ 140.5 (d, $J_{C–F}$ = 244.3 Hz), 128.3, 127.5, 127.4 (d, $J_{C–F}$ = 11.6 Hz), 122.2 (d, $J_{C–F}$ = 1.9 Hz), 115.2 (d, $J_{C–F}$ = 4.9 Hz), 111.8; ^{19}F NMR (376 MHz, $CDCl_3$) δ –183.9; HRMS (ESI) *m*/*z* calcd for $C_7H_6FN_2$ $[M+H]^+$: 137.0510 found 137.0509.

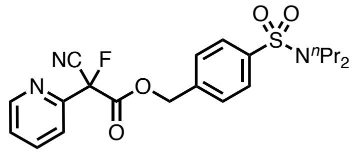

4-(*N*,*N*-Dipropylsulfamoyl)benzyl 2-cyano-2-fluoro-2-(pyridin-2-yl)acetate (2AA)

Purification by PTLC (hexane/EtOAc = 4:1) afforded **2AA** (85.8 mg, 99% yield) as a colorless oil. ^{1}H NMR (400 MHz, $CDCl_3$) δ 8.65 (dd, *J* = 4.8, 1.2 Hz, 1H), 7.90 (td, *J* = 8.0, 1.2 Hz, 1H), 7.78 (d, *J* = 8.8 Hz, 2H), 7.73 (d, *J* = 8.0 Hz, 1H), 7.49–7.43 (m, 3H), 5.42 (s, 2H), 3.07 (t, *J* = 7.6 Hz, 4H), 1.61–1.51 (m, 4H), 0.87 (t, *J* = 7.6 Hz, 6H); ^{13}C NMR (101 MHz, $CDCl_3$) δ 161.8 (d, $J_{C–F}$ = 28.9 Hz), 150.4 (d, $J_{C–F}$ = 26.1 Hz), 149.9, 140.6, 138.1, 128.2, 127.4, 125.9, 121.1 (d, $J_{C–F}$ = 3.8 Hz), 113.3 (d, $J_{C–F}$ = 33.7 Hz), 87.4 (d, $J_{C–F}$ = 198.4 Hz), 68.2, 50.1, 22.0, 11.1; ^{19}F NMR (376 MHz, $CDCl_3$) δ –147.9; HRMS (ESI) *m*/*z* calcd for $C_{21}H_{25}FN_3O_4S$ $[M+H]^+$: 434.1544 found 434.1545.

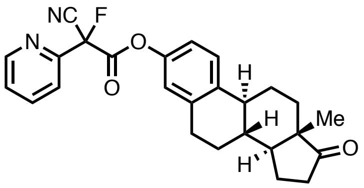

(8*R*,9*S*,13*S*,14*S*)-13-Methyl-17-oxo-7,8,9,11,12,13,14,15,16,17-decahydro-6*H*-cyclopenta[*a*]phenanthren-3-yl 2-cyano-2-fluoro-2-(pyridin-2-yl)acetate (2AB)

Purification by PTLC (hexane/EtOAc = 4:1) afforded **2AB** (63.1 mg, 73% yield) as a white solid. ^{1}H NMR (400 MHz, $CDCl_3$) δ 8.74 (d, *J* = 4.4 Hz, 1H), 7.94 (td, *J* = 7.6, 2.0 Hz, 1H), 7.83 (d, *J* = 7.6 Hz, 1H), 7.54–7.48 (m, 1H), 7.30 (d, *J* = 8.4 Hz, 1H), 6.94 (d, *J* = 8.4 Hz, 1H), 6.91 (s, 1H), 2.91 (dd, *J* = 8.8, 4.0 Hz, 2H), 2.56–2.46 (m, 1H), 2.43–2.36 (m, 1H), 2.33–2.23 (m, 1H), 2.20–1.90 (m, 4H), 1.75–1.48 (m, 6H), 0.91 (s, 3H); ^{13}C NMR (101 MHz, $CDCl_3$) δ 220.6, 160.8 (d, $J_{C–F}$ = 29.1 Hz), 150.6 (d, $J_{C–F}$ = 25.1 Hz), 150.0, 147.8, 138.6, 138.5, 138.1, 126.6, 125.9, 121.3 (d, $J_{C–F}$ = 3.9 Hz), 120.6, 117.7, 113.3 (d, $J_{C–F}$ = 33.9 Hz), 87.4 (d, $J_{C–F}$ = 198.8 Hz), 50.3, 47.8, 44.0, 37.8, 35.8, 31.4, 29.3, 26.1, 25.7,

21.5, 13.8; ^{19}F NMR (376 MHz, $CDCl_3$) δ –146.4; HRMS (ESI) *m/z* calcd for $C_{26}H_{26}FN_2O_3$ $[M+H]^+$: 433.1922 found 433.1920.

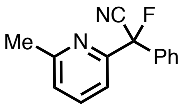

2-Fluoro-2-(6-methylpyridin-2-yl)-2-phenylacetonitrile (2AC)

The reaction was performed with $NaClO_4$ (1.0 equiv). Purification by PTLC (hexane/EtOAc = 4:1) afforded **2AC** (39.4 mg, 87% yield) as a colorless oil. ^{1}H NMR (400 MHz, $CDCl_3$) δ 7.67 (t, *J* = 8.0 Hz, 1H), 7.60–7.53 (m, 2H), 7.45–7.39 (m, 3H), 7.35 (d, *J* = 8.0 Hz, 1H), 7.19 (d, *J* = 8.0 Hz, 1H), 2.58 (s, 3H); ^{13}C NMR (101 MHz, $CDCl_3$) δ 159.1, 154.3 (d, $J_{C–F}$ = 28.1 Hz), 137.4, 136.1 (d, $J_{C–F}$ = 24.3 Hz), 130.1, 128.8, 125.9 (d, $J_{C–F}$ = 5.9 Hz), 124.1, 116.9 (d, $J_{C–F}$ = 33.9 Hz), 116.5 (d, $J_{C–F}$ = 5.8 Hz), 92.1 (d, $J_{C–F}$ = 186.2 Hz), 24.4; ^{19}F NMR (376 MHz, $CDCl_3$) δ –141.7; HRMS (ESI) *m/z* calcd for $C_{14}H_{12}FN_2$ $[M+H]^+$: 227.0979 found 227.0977.

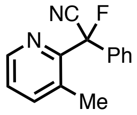

2-Fluoro-2-(3-methylpyridin-2-yl)-2-phenylacetonitrile (2AD)

Purification by PTLC (hexane/EtOAc = 4:1) afforded **2AD** (39.6 mg, 88% yield) as a colorless oil. ^{1}H NMR (400 MHz, $CDCl_3$) δ 8.57 (d, *J* = 5.2 Hz, 1H), 7.56 (dd, *J* = 7.6, 0.8 Hz, 1H), 7.46–7.39 (m, 5H), 7.33 (dd, *J* = 7.6, 5.2 Hz, 1H), 2.18 (d, $J_{H–F}$ = 3.6 Hz, 3H); ^{13}C NMR (101 MHz, $CDCl_3$) δ 151.9 (d, $J_{C–F}$ = 25.1 Hz), 146.1, 140.8, 135.7 (d, $J_{C–F}$ = 24.2 Hz), 132.5, 130.0, 128.9, 125.5 (d, $J_{C–F}$ = 5.9 Hz), 124.8, 116.9 (d, $J_{C–F}$ = 34.9 Hz), 94.7 (d, $J_{C–F}$ = 187.3 Hz), 18.5 (d, $J_{C–F}$ = 5.9 Hz); ^{19}F NMR (376 MHz, $CDCl_3$) δ –143.5; HRMS (ESI) *m/z* calcd for $C_{14}H_{12}FN_2$ $[M+H]^+$: 227.0979 found 227.0978.

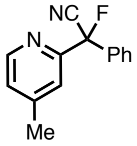

2-Fluoro-2-(4-methylpyridin-2-yl)-2-phenylacetonitrile (2AE)

The reaction was performed with $NaClO_4$ (1.0 equiv). Purification by PTLC (hexane/EtOAc = 4:1) afforded **2AE** (15.4 mg, 34% yield) as a colorless oil. ^{1}H NMR (400 MHz, $CDCl_3$) δ 8.51 (d, *J* = 4.8 Hz, 1H), 7.59–7.53 (m, 2H), 7.46–7.40 (m, 4H), 7.16 (d, *J* = 4.8 Hz, 1H), 2.42 (s, 3H); ^{13}C NMR (101 MHz, $CDCl_3$) δ 155.0 (d, $J_{C–F}$ = 28.1 Hz), 149.5, 149.1, 136.0 (d, $J_{C–F}$ = 24.2 Hz), 130.2, 128.9, 125.8 (d, $J_{C–F}$ = 5.9 Hz), 125.4, 120.4 (d, $J_{C–F}$ = 5.8 Hz), 116.8 (d, $J_{C–F}$ = 33.9 Hz), 92.0 (d, $J_{C–F}$ = 187.3 Hz), 21.3; ^{19}F NMR (376 MHz, $CDCl_3$) δ –142.2; HRMS (ESI) *m/z* calcd for $C_{14}H_{12}FN_2$ $[M+H]^+$: 227.0979 found 227.0977.

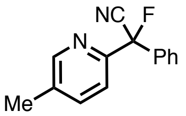

2-Fluoro-2-(5-methylpyridin-2-yl)-2-phenylacetonitrile (2AF)

Purification by PTLC (hexane/EtOAc = 4:1) afforded **2AF** (31.4 mg, 69% yield) as a colorless oil. ^{1}H NMR (400 MHz, $CDCl_3$) δ 8.49 (s, 1H), 7.61 (dd, *J* = 8.0, 1.6 Hz, 1H), 7.59–7.52 (m, 2H), 7.47 (d, *J* = 8.0 Hz, 1H), 7.45–7.38 (m, 3H), 2.38 (s, 3H); ^{13}C NMR (101 MHz, $CDCl_3$) δ 152.4 (d, $J_{C–F}$ = 29.1 Hz), 150.2, 137.9, 136.1 (d, $J_{C–F}$ = 24.2 Hz), 134.6, 130.1 (d, $J_{C–F}$ = 1.9 Hz), 128.9, 125.8 (d, $J_{C–F}$ = 5.8 Hz), 119.3 (d, $J_{C–F}$ = 5.8 Hz), 116.8 (d, $J_{C–F}$ = 33.9 Hz), 92.0 (d, $J_{C–F}$= 186.1 Hz), 18.2; ^{19}F NMR (376 MHz, $CDCl_3$) δ –141.0; HRMS (ESI) *m*/*z* calcd for $C_{14}H_{12}FN_2$ $[M+H]^+$: 227.0979 found 227.0977.

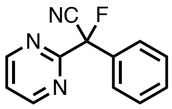

2-Fluoro-2-phenyl-2-(pyrimidin-2-yl)acetonitrile (2AG)

Purification by PTLC (chloroform/EtOAc = 19:1) afforded **2AG** (41.3 mg, 97% yield) as a white solid. ^{1}H NMR (400 MHz, $CDCl_3$) δ 8.86 (d, *J* = 4.8 Hz, 2H), 7.71–7.66 (m, 2H),7.47–7.39 (m, 3H), 7.37 (t, *J* = 4.8 Hz, 1H); ^{13}C NMR (101 MHz, $CDCl_3$) δ 163.4 (d, $J_{C–F}$ = 24.0 Hz), 158.0, 134.8 (d, $J_{C–F}$ = 24.1 Hz), 130.4, 128.9, 125.5 (d, $J_{C–F}$ = 5.8 Hz), 121.5, 116.2 (d, $J_{C–F}$ = 32.7 Hz), 91.3 (d, $J_{C–F}$ = 191.7 Hz); ^{19}F NMR (376 MHz, $CDCl_3$) δ –144.5; HRMS (ESI) *m*/*z* calcd for $C_{12}H_9FN_3$ $[M+H]^+$: 214.0775 found 214.0776.

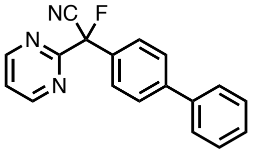

2-([1,1'-Biphenyl]-4-yl)-2-fluoro-2-(pyrimidin-2-yl)acetonitrile (2AH)

Purification by PTLC (chloroform/EtOAc = 19:1) afforded **2AH** (50.3 mg, 87% yield) as a white solid. ^{1}H NMR (400 MHz, $CDCl_3$) δ 8.88 (d, *J* = 5.2 Hz, 2H), 7.75 (d, *J* = 8.4 Hz, 2H), 7.65 (d, *J* = 8.4 Hz, 2H), 7.56 (d, *J* = 7.2 Hz, 2H), 7.45 (t, *J* = 7.2 Hz, 2H), 7.40–7.36 (m, 2H); ^{13}C NMR (101 MHz, $CDCl_3$) δ 163.5 (d, $J_{C–F}$ = 26.2 Hz), 158.1, 143.4, 139.8, 133.7 (d, $J_{C–F}$ = 24.2 Hz), 128.9, 128.0, 127.7, 127.2, 126.1 (d, $J_{C–F}$ = 5.9 Hz), 121.6, 116.2 (d, $J_{C–F}$ = 32.9 Hz), 91.2 (d, $J_{C–F}$ = 193.0 Hz); ^{19}F NMR (376 MHz, $CDCl_3$) δ –143.8; HRMS (ESI) *m*/*z* calcd for $C_{18}H_{13}FN_3$ $[M+H]^+$: 290.1088 found 290.1087.

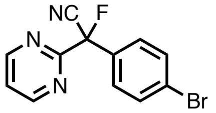

2-(4-Bromophenyl)-2-fluoro-2-(pyrimidin-2-yl)acetonitrile (2AI)

Purification by PTLC (chloroform/EtOAc = 19:1) afforded **2AI** (50.3 mg, 86% yield) as a white solid. ^{1}H NMR (400 MHz, $CDCl_3$) δ 8.85 (d, J = 4.8 Hz, 1H), 7.62–7.53 (m, 4H), 7.39 (t, J = 4.8 Hz, 1H); ^{13}C NMR (101 MHz, $CDCl_3$) δ 163.0 (d, $J_{C–F}$ = 25.3 Hz), 158.1, 133.9 (d, $J_{C–F}$ = 25.3 Hz), 132.2, 127.2 (d, $J_{C–F}$ = 5.9 Hz), 125.0, 121.7, 115.8 (d, $J_{C–F}$ = 32.0 Hz), 90.9 (d, $J_{C–F}$ = 194.0 Hz); ^{19}F NMR (376 MHz, $CDCl_3$) δ –144.8; HRMS (ESI) m/z calcd for $C_{12}H_7BrN_3$ $[M–F]^+$: 271.9818 found 271.9816.

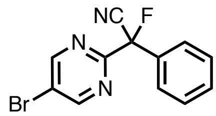

2-(5-Bromopyrimidin-2-yl)-2-fluoro-2-phenylacetonitrile (2AJ)

Purification by PTLC (chloroform/EtOAc = 19:1) afforded **2AJ** (58.4 mg, quant.) as a white solid. ^{1}H NMR (400 MHz, $CDCl_3$) δ 8.89 (s, 2H), 7.71–7.62 (m, 2H), 7.47–7.44 (m, 3H); ^{13}C NMR (101 MHz, $CDCl_3$) δ 161.5 (d, $J_{C–F}$ = 26.3 Hz), 158.8, 134.5 (d, $J_{C–F}$ = 25.3 Hz), 130.6, 129.0, 125.5 (d, $J_{C–F}$ = 5.9 Hz), 121.1, 115.8 (d, $J_{C–F}$ = 32.0 Hz), 90.9 (d, $J_{C–F}$ = 193.9 Hz); ^{19}F NMR (376 MHz, $CDCl_3$) δ –144.2; HRMS (ESI) m/z calcd for $C_{12}H_7BrN_3$ $[M–F]^+$: 271.9818 found 271.9817.

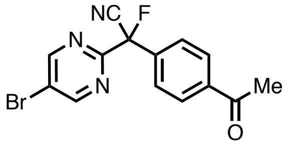

2-(4-Acetylphenyl)-2-(5-bromopyrimidin-2-yl)-2-fluoroacetonitrile (2AK)

Purification by PTLC (chloroform/EtOAc = 19:1) afforded **2AK** (59.5 mg, 89% yield) as a white solid. ^{1}H NMR (400 MHz, $CDCl_3$) δ 8.89 (s, 2H), 8.03 (d, J = 8.0 Hz, 2H), 8.03 (d, J = 8.0 Hz, 2H), 2.62 (s, 3H); ^{13}C NMR (101 MHz, $CDCl_3$) δ 196.9, 160.8 (d, $J_{C–F}$ = 25.0 Hz), 158.9, 138.6 (d, $J_{C–F}$ = 25.0 Hz), 138.5, 128.9, 125.7 (d, $J_{C–F}$ = 5.8 Hz), 121.4, 115.3 (d, $J_{C–F}$ = 31.8 Hz), 90.5 (d, $J_{C–F}$ = 194.5 Hz), 26.7; ^{19}F NMR (376 MHz, $CDCl_3$) δ –146.1; HRMS (ESI) m/z calcd for $C_{14}H_9BrN_3O$ $[M–F]^+$: 313.9924 found 313.9922.

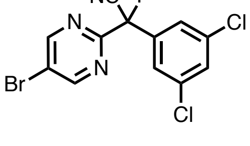

2-(5-Bromopyrimidin-2-yl)-2-(3,5-dichlorophenyl)-2-fluoroacetonitrile (2AL)

Purification by PTLC (chloroform/EtOAc = 19:1) afforded **2AL** (44.0 mg, 61% yield) as a pale yellow solid. ^{1}H NMR (400 MHz, $CDCl_3$) δ 8.91 (s, 2H), 7.57 (d, J = 1.6 Hz, 2H), 7.44 (t, J = 1.6 Hz, 1H); ^{13}C NMR (101 MHz, $CDCl_3$) δ 160.4 (d, $J_{C–F}$ = 25.1 Hz), 159.0, 137.4 (d, $J_{C–F}$ = 25.1 Hz), 135.9, 130.8,

124.0 (d, J_{C-F} = 6.8 Hz), 121.6, 114.9 (d, J_{C-F} = 32.0 Hz), 89.6 (d, J_{C-F} = 196.8 Hz); ^{19}F NMR (376 MHz, $CDCl_3$) δ –146.0; HRMS (DART) *m*/*z* calcd for $C_{12}H_6BrCl_2FN_3$ $[M+H]^+$: 359.9101 found 359.9099.

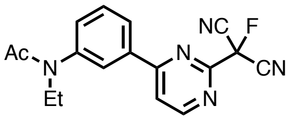

N-(3-(2-(Dicyanofluoromethyl)pyrimidin-4-yl)phenyl)-N-ethylacetamide (2AM)

Purification by Isolera® (hexane/EtOAc = 19:1 to 9:1) afforded **2AM** (31.7 mg, 49% yield) as a yellow solid. ^{1}H NMR (400 MHz, $CDCl_3$) δ 9.02 (d, *J* = 4.8 Hz, 1H), 8.20 (d, *J* = 7.6 Hz, 1H), 8.02 (s, 1H), 7.98 (d, *J* = 4.8 Hz, 1H), 7.66 (t, *J* = 7.6 Hz, 1H), 7.43 (d, *J* = 7.6 Hz, 1H), 3.83 (q, *J* = 7.2 Hz, 2H), 1.89 (s, 3H), 1.17 (t, *J* = 7.2 Hz, 3H); ^{13}C NMR (101 MHz, $CDCl_3$) δ 169.6, 164.9, 159.6, 157.7 (d, J_{C-F} = 22.1 Hz), 144.2, 136.0, 132.4, 130.8, 127.1, 126.9, 118.6, 110.0 (d, J_{C-F} = 35.6 Hz), 78.7 (d, J_{C-F} = 201.3 Hz), 44.0, 22.9, 13.1; ^{19}F NMR (376 MHz, $CDCl_3$) δ –137.8; HRMS (ESI) *m*/*z* calcd for $C_{17}H_{15}FN_5O$ $[M+H]^+$: 324.1255 found 324.1253.

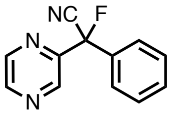

2-Fluoro-2-phenyl-2-(pyrazin-2-yl)acetonitrile (2AN)

The reaction was performed with $NaClO_4$ (1.0 equiv). Purification by PTLC (hexane/EtOAc = 2:1) afforded **2AN** (17.1 mg, 40% yield) as a white solid. ^{1}H NMR (400 MHz, $CDCl_3$) δ 8.91 (d, *J* = 1.2 Hz, 1H), 8.69 (d, *J* = 2.0 Hz, 1H), 8.61–8.55 (m, 1H), 7.61–7.55 (m, 2H), 7.49–7.44 (m, 3H); ^{13}C NMR (101 MHz, $CDCl_3$) δ 151.1 (d, J_{C-F} = 29.1 Hz), 145.8, 144.0, 141.3 (d, J_{C-F} = 6.8 Hz), 134.7 (d, J_{C-F} = 23.2 Hz), 130.7, 129.2, 125.6 (d, J_{C-F} = 5.8 Hz), 115.8 (d, J_{C-F} = 32.0 Hz), 91.2 (d, J_{C-F} = 187.2 Hz); ^{19}F NMR (376 MHz, $CDCl_3$) δ –145.6; HRMS (ESI) *m*/*z* calcd for $C_{12}H_9FN_3$ $[M+H]^+$: 214.0775 found 214.0775.

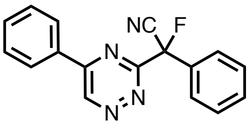

2-Fluoro-2-phenyl-2-(5-phenyl-1,2,4-triazin-3-yl)acetonitrile (2AO)

Purification by PTLC (hexane/EtOAc = 4:1) afforded **2AO** (18.0 mg, 31% yield) as a yellow solid. ^{1}H NMR (400 MHz, $CDCl_3$) δ 9.73 (s, 1H), 8.23 (d, *J* = 8.8 Hz, 2H), 7.83–7.75 (m, 2H), 7.69–7.63 (m, 1H), 7.62–7.56 (m, 2H), 7.50–7.44 (m, 3H); ^{13}C NMR (101 MHz, $CDCl_3$) δ 163.2 (d, J_{C-F} = 25.1 Hz), 156.1, 146.2, 134.2 (d, J_{C-F} = 25.3 Hz), 133.7, 132.0, 130.7, 129.7, 129.1, 128.0, 125.7 (d, J_{C-F} = 4.8 Hz), 115.6 (d, J_{C-F} = 32.0 Hz), 90.8 (d, J_{C-F} = 194.9 Hz); ^{19}F NMR (376 MHz, $CDCl_3$) δ –146.0; HRMS (ESI) *m*/*z* calcd for $C_{17}H_{11}FN_4Na$ $[M+Na]^+$: 313.0860 found 313.0858.

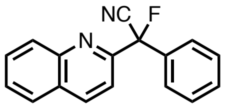

2-Fluoro-2-phenyl-2-(quinolin-2-yl)acetonitrile (2AP)

The reaction was performed with $NaClO_4$ (1.0 equiv). Purification by PTLC (hexane/EtOAc = 4:1) afforded **2AP** (45.6 mg, 87% yield) as a colorless oil. ^{1}H NMR (400 MHz, $CDCl_3$) δ 8.27 (d, J = 8.4 Hz, 1H), 8.20 (d, J = 8.4 Hz, 1H), 7.86 (d, J = 8.4 Hz, 1H), 7.81–7.77 (m, 1H), 7.64–7.60 (m, 4H), 7.44–7.41 (m, 3H); ^{13}C NMR (101 MHz, $CDCl_3$) δ 154.7 (d, $J_{C–F}$ = 29.1 Hz), 147.1, 138.0, 136.0 (d, $J_{C–F}$ = 24.3 Hz), 130.4, 130.1, 130.0, 128.9, 127.9, 127.6, 125.6 (d, $J_{C–F}$ = 5.9 Hz), 116.7 (d, $J_{C–F}$ = 32.9 Hz), 116.6 (d, $J_{C–F}$ = 4.8 Hz), 92.8 (d, $J_{C–F}$ = 187.3 Hz) (one peak is missing due to overlapping); ^{19}F NMR (376 MHz, $CDCl_3$) δ –143.3; HRMS (ESI) *m/z* calcd for $C_{17}H_{12}FN_2$ $[M+H]^+$: 263.0979 found 263.0980.

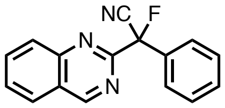

2-Fluoro-2-phenyl-2-(quinazolin-2-yl)acetonitrile (2AQ)

Purification by PTLC (hexane/EtOAc = 4:1) afforded **2AQ** (41.6 mg, 79% yield) as a white solid. ^{1}H NMR (400 MHz, $CDCl_3$) δ 9.45 (s, 1H), 8.20 (d, J = 8.8 Hz, 1H), 8.05–7.99 (m, 2H), 7.79–7.74 (m, 3H), 7.47–7.41 (m, 3H); ^{13}C NMR (101 MHz, $CDCl_3$) δ 161.8, 159.3 (d, $J_{C–F}$ = 24.2 Hz), 150.0, 135.4, 135.3 (d, $J_{C–F}$ = 24.2 Hz), 130.4, 129.4, 129.0, 127.4, 125.9 (d, $J_{C–F}$ = 5.9 Hz), 124.4, 116.6 (d, $J_{C–F}$ = 32.0 Hz), 91.7 (d, $J_{C–F}$ = 193.0 Hz) (one peak is missing due to overlapping); ^{19}F NMR (376 MHz, $CDCl_3$) δ –143.9; HRMS (ESI) *m/z* calcd for $C_{16}H_{11}FN_3$ $[M+H]^+$: 264.0932 found 264.0932.

5. Effect of Parameters

5-1. Screening of Fluorinating Reagents

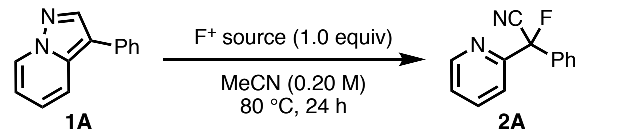

entry	F^+ source	yield of **2A**[a] / %	recovery of **1A**[a] / %
1	**F1**	27	43
2	**F2**	34	0
3	**F3**	39	37
4	**F4**	3	0
5	**F5**	5	3
6	NFSI	97	0
7	Selectfluor®	quant.	0

[a] Yields were determined by ^1H NMR analysis using CH_2Br_2 as an internal standard.

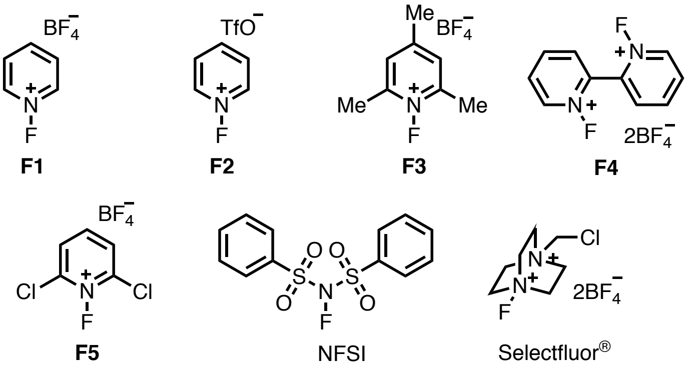

5-2. Effect of Solvent

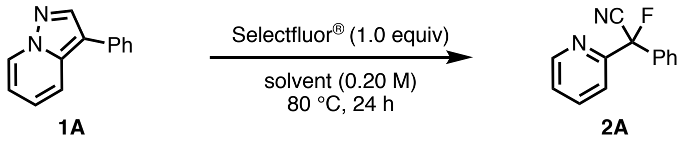

entry	solvent	yield of **2A**[a] / %	recovery of **1A**[a] / %
1	MeCN	quant.	0
2	acetone	65	0
3	DMF	64	0
4	MeOH	58	0
5	EtOAc	98	0

[a] Yields were determined by ^1H NMR analysis using CH_2Br_2 as an internal standard.

5-3. Effect of Temperature

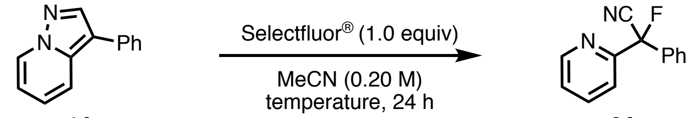

1A **2A**

entry	temperature / °C	yield of **2A**[a] / %	recovery of **1A**[a] / %
1	50	68	0
2	60	74	0
3	70	87	0
4	80	quant.	0

[a] Yields were determined by ^{1}H NMR analysis using CH_2Br_2 as an internal standard.

5-4. Effect of Temperature for 1Z

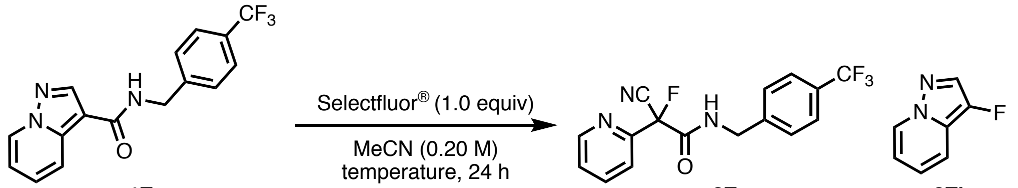

1Z **2Z** **2Z'**

entry	temperature / °C	yield of **2Z** / %	yield of **2Z'** / %
1	80	13	60
2	RT	24	42
3	–30	39	21

5-5. Effect of Additive

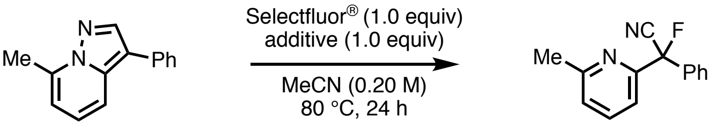

1AC
200 μmol

2AC

entry	additive	yield of **2AC**[a] / %	recovery of **1AC**[a] / %
1	none	53(51)[b]	0
2	$LiPF_6$	42	0
3	$NaPF_6$	0	0
4	KPF_6	0	0
5	$LiClO_4$	37	0
6	$NaClO_4$	88(87)[b]	0
7	LiOTf	18	0
8	NaOTf	39	0
9	KOTf	72	0
10	TBAC	42	12

[a] Yields were determined by ^{1}H NMR analysis using CH_2Br_2 as an internal standard.
[b] Numbers in parentheses are isolated yield.

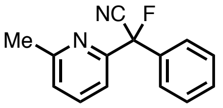

2AC
87%
51% (w/o $NaClO_4$)

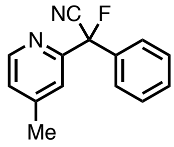

2AE
34%
27% (w/o $NaClO_4$)

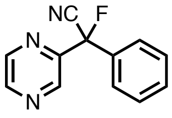

2AN
87%
56% (w/o $NaClO_4$)

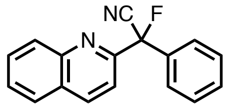

2AP
49%
29% (w/o $NaClO_4$)

5-6. Addition of Radical Scavengers

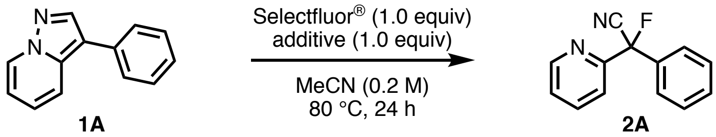

entry	additive	yield of **2A**[a] (%)	recovery of **1A**[a] (%)
1	TEMPO	36	60
2	Galvinoxyl	94	0
3	BHT	87	0

[a] Yields were determined by ^{1}H NMR analyis using CH_2Br_2 as an internal standard.

6. Fluorination Tracking by ^{1}H NMR

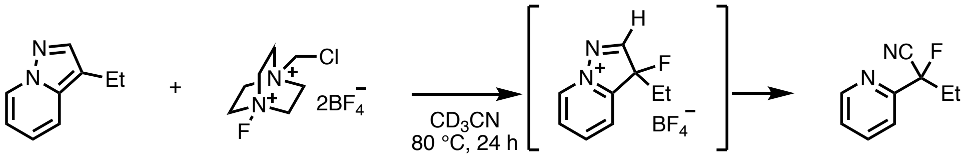

A Wilmad® screw-cap NMR tube was dried with a heat-gun *in vacuo* and filled with N_2 gas after cooling to room temperature. To this tube was added **1M** (7.3 mg, 50.0 μmol, 1.0 equiv) and Selectfluor® (17.8 mg, 50.0 μmol, 1.0 equiv). The tube was placed under vacuum and refilled with N_2 gas three times. To this tube was added acetonitrile-*d*$_3$ (0.5 mL). The tube was sealed with a screw cap and then heated at 80 °C for 24 h in an oil bath. The reaction progress was measured by ^{1}H NMR spectra at 1, 2, 4, 8, 16, and 24 h.

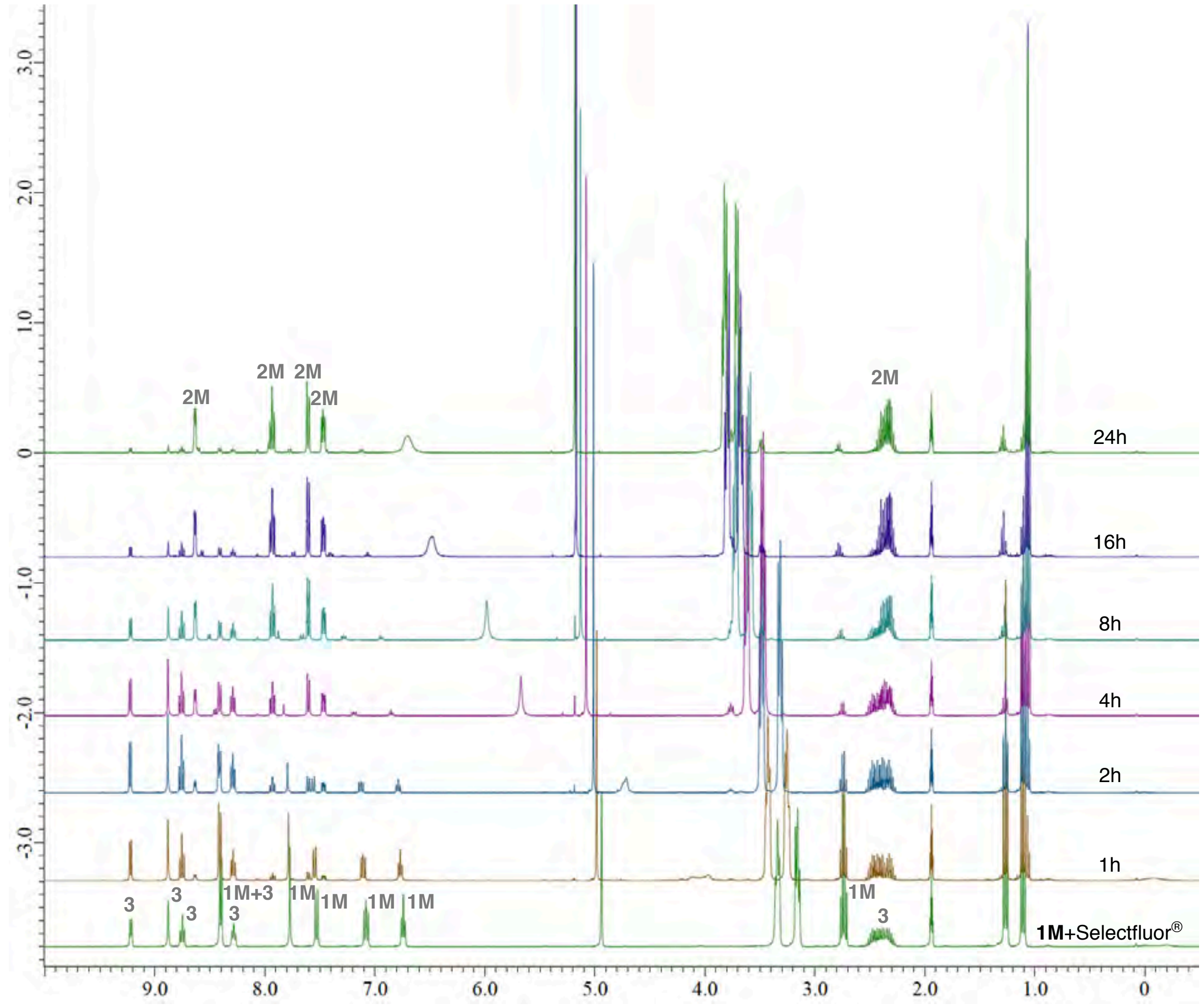

7. Another Role of Selectfluor®

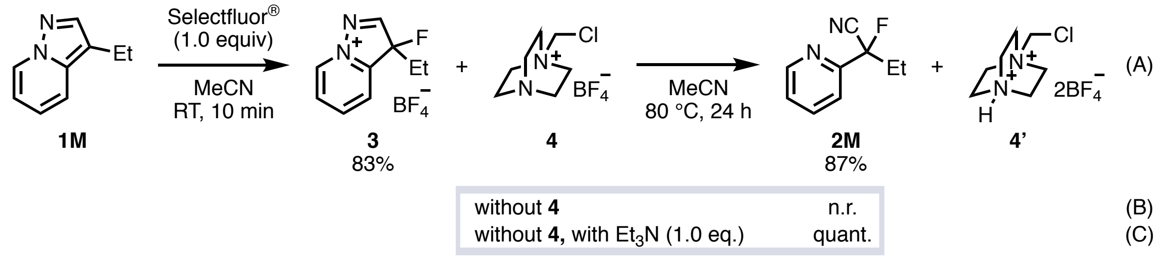

Preparation of 3

An 8-mL glass tube equipped with a screw cap containing a magnetic stirring bar was dried with a heat-gun *in vacuo* and filled with N_2 gas after cooling to room temperature. To this tube were added **1M** (29.2 mg, 0.200 mmol, 1.0 equiv) and Selectfluor® (70.8 mg, 0.200 mmol, 1.0 equiv). The tube was placed under vacuum and refilled with N_2 gas three times. To this tube was added MeCN (1.0 mL). After stirred at room temperature for 10 min, the mixture was concentrated *in vacuo* and washed with chloroform (to remove remaining **1M** and **2M**). The filtrate was concentrated *in vacuo* and extracted with acetone (to remove remaining Selectfluor® and **4'**). The solution was concentrated *in vacuo* to afford the crude material containing **3** (83% yield) and **4**. The yield of **3** was determined by ^{1}H NMR analysis using CH_2Br_2 as an internal standard.

Experiment A

An 8-mL glass tube equipped with a screw cap containing a magnetic stirring bar was dried with a heat-gun *in vacuo* and filled with N_2 gas after cooling to room temperature. To this tube was added a solution of the crude material containing **3** and **4** in MeCN (1.0 mL). The tube was sealed with a screw cap and then heated at 80 °C for 24 h. After cooling the reaction mixture to room temperature, the mixture was concentrated *in vacuo*. The residue was filtered with chloroform to afford **2M** (21.1 mg, 87% yield) as a colorless oil.

Experiment B

To the crude material containing **3** and **4** were added MeCN (1.0 mL) and 4.0 M HCl in 1,4-dioxane (100 µL, 0.400 mmol) dropwise at 0 °C. After stirred at room temperature for 3 h, the mixture was concentrated *in vacuo* and extracted acetone (to remove remaining **4'**). An 8-mL glass tube equipped with a screw cap containing a magnetic stirring bar was dried with a heat-gun *in vacuo* and filled with N_2 gas after cooling to room temperature. To this tube was added a solution of the obtained material containing **3** was added MeCN (1.0 mL). The tube was sealed with a screw cap and then heated at 80 °C for 24 h. However, no reaction progress was observed.

Experiment C

To the crude material containing **3** and **4** were added MeCN (1.0 mL) and 4.0 M HCl in 1,4-dioxane (100 µL, 0.400 mmol) dropwise at 0 °C. After stirred at room temperature for 3 h, the mixture was concentrated *in vacuo* and extracted acetone (to remove remaining **4'**). An 8-mL glass tube equipped with a screw cap containing a magnetic stirring bar was dried with a heat-gun *in vacuo* and filled with

N_2 gas after cooling to room temperature. To this tube was added a solution of the obtained material containing **3** were added MeCN (1.0 mL) and Et_3N (28 μL, 0.200 mmol). The tube was sealed with a screw cap and then heated at 80 °C for 24 h. After cooling the reaction mixture to room temperature, the mixture was concentrated *in vacuo*. The residue was filtered with chloroform to afford **2M** (24.3 mg, quant.) as a colorless oil.

8. Fluorination of 5 and 7

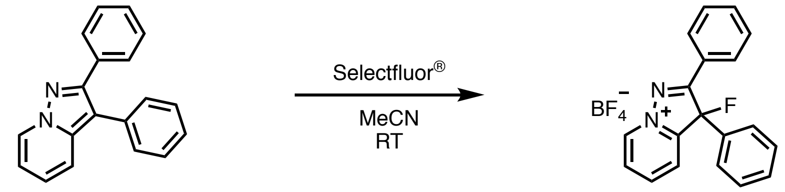

5 **6**

An 8-mL glass tube equipped with a screw cap containing a magnetic stirring bar was dried with a heat-gun *in vacuo* and filled with N_2 gas after cooling to room temperature. To this tube were added 2,3-diphenylpyrazolo[1,5-*a*]pyridine (**5**: 135 mg, 0.500 mmol, 1.0 equiv) and Selectfluor® (177 mg, 0.500 mmol, 1.0 equiv). The tube was placed under vacuum and refilled with N_2 gas three times. To this tube was added MeCN (2.5 mL). The tube was sealed with a screw cap and then the mixture was stirred at 80 °C for 24 h. After cooling the mixture to room temperature, 4.0 M HCl in 1,4-dioxane (150 μL, 0.600 mmol, 1.2 equiv) was added. After stirring the mixture for 1 h, the solution was concentrated *in vacuo*. The resulting material was extracted with acetone, and insoluble solids were removed by filtration. The filtrate was concentrated *in vacuo*, recrystallized from Et_2O/MeCN to afford 3-fluoro-2,3-diphenyl-3*H*-pyrazolo[1,5-*a*]pyridin-8-ium tetrafluoroborate (**6**: 160 mg, 85% yield) as a white solid. ^{1}H NMR (400 MHz, acetonitrile-d_3) δ 9.30 (d, J = 7.2 Hz, 1H), 8.63 (t, J = 7.2 Hz, 1H), 8.28 (t, J = 7.2 Hz, 1H), 8.22 (d, J = 7.2 Hz, 1H), 8.05 (d, J = 8.0 Hz, 2H), 7.77 (t, J = 8.0 Hz, 1H), 7.61–7.57 (m, 2H), 7.56–7.50 (m, 5H); ^{13}C NMR (101 MHz, acetonitrile-d_3) δ 173.4 (d, $J_{C–F}$ = 15.6 Hz), 148.8, 148.0 (d, $J_{C–F}$ = 27.2 Hz), 141.2, 136.8, 132.4, 132.2 (d, $J_{C–F}$ = 29.1 Hz), 131.6, 131.0 (d, $J_{C–F}$ = 2.9 Hz), 130.9, 125.5, 125.32, 125.25, 125.2 (d, $J_{C–F}$ = 5.8 Hz), 103.8 (d, $J_{C–F}$ = 203.7 Hz); ^{19}F NMR (376 MHz, $CDCl_3$) δ –150.1 (m, 4F), –161.6; HRMS (ESI) *m*/*z* calcd for $C_{19}H_{14}FN_2$ $[M–BF_4^-]^+$: 289.1136 found 289.1136.

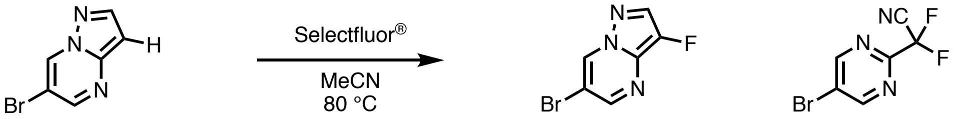

7 **8** **9**

An 8-mL glass tube equipped with a screw cap containing a magnetic stirring bar was dried with a heat-gun *in vacuo* and filled with N_2 gas after cooling to room temperature. To this tube were added 6-bromopyrazolo[1,5-*a*]pyrimidine (**7**: 39.6 mg, 0.200 mmol, 1.0 equiv) and Selectfluor® (70.9 mg, 0.200 mmol, 1.0 equiv). The tube was placed under vacuum and refilled with N_2 gas three times. To this tube

was added MeCN (1.0 mL). The tube was sealed with a screw cap and then heated at 80 °C for 24 h with stirring. After cooling the reaction mixture to room temperature, the mixture was concentrated *in vacuo*. The residue was purified by Isolera® (hexane/EtOAc = 19:1 to 9:1) to afford 6-bromo-3-fluoropyrazolo[1,5-*a*]pyrimidine (**8**: 21.6 mg, 50% yield) as a white solid and 2-(5-bromopyrimidin-2-yl)-2,2-difluoroacetonitrile (**9**: 4.7 mg, 10% yield) as a colorless oil.

When reaction was performed with Selectfluor® (2.0 equiv), only **9** was obtained (16.4 mg, 58% yield).

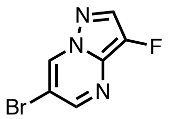

6-Bromo-3-fluoropyrazolo[1,5-*a*]pyrimidine (8)

^{1}H NMR (400 MHz, $CDCl_3$) δ 8.68 (dd, J = 2.4, 1.2 Hz, 1H), 8.43 (d, J = 1.6 Hz, 1H), 8.00 (d, $J_{H–F}$ = 3.6 Hz, 1H); ^{13}C NMR (101 MHz, $CDCl_3$) δ 149.4 (d, $J_{C–F}$ = 20.2 Hz), 138.7 (d, $J_{C–F}$ = 251.2 Hz), 134.6, 133.3 (d, $J_{C–F}$ = 23.2 Hz), 131.8 (d, $J_{C–F}$ = 11.6 Hz), 104.0; ^{19}F NMR (376 MHz, $CDCl_3$) δ –180.9; HRMS (DART) *m*/*z* calcd for $C_6H_4BrFN_3$ [M+H]$^+$: 215.9567 found 215.9566.

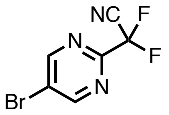

2-(5-Bromopyrimidin-2-yl)-2,2-difluoroacetonitrile (9)

^{1}H NMR (400 MHz, $CDCl_3$) δ 9.00 (s, 2H); ^{13}C NMR (101 MHz, $CDCl_3$) δ 159.2, 155.6 (t, $J_{C–F}$ = 27.6 Hz), 123.7, 111.2 (t, $J_{C–F}$ = 44.6 Hz), 105.9 (t, $J_{C–F}$ = 250.2 Hz); ^{19}F NMR (376 MHz, $CDCl_3$) δ –137.2; HRMS (DART) *m*/*z* calcd for $C_6H_5OBrF_2N_3$ [M+H_3O]$^+$: 251.9579 found 251.9574.

9. Studies Toward Asymmetric Fluorination

9-1. Using Chiral Phosphoric Acid[15]

9-1-1. Screening of Solvents

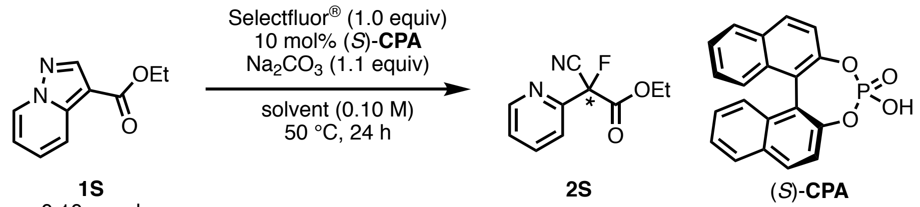

entry	solvent	yield of **2S**[a] / %	recovery of **1S**[a] / %
1	*n*-heptane	23	73
2	cyclohexane	15	78
3	*n*-hexane	20	83
4	PhMe	0	98
5	PhF	0	100
6	*m*-xylene	0	100
7	1,4-dioxane	0	100
8	DME	0	100
9	Et_2O	8	90
10	$^iPr_2O$	5	95
11	CPME	0	80
12	THF	0	100
13	$CHCl_3$	0	100
14	CH_2Cl_2	0	100
15	ZEORORA® H	0	100
16	PhMe/MeCN (1:1)	9	90
17	acetone	14	78

[a] Yields were determined by ^1H NMR analysis using CH_2Br_2 as an internal standard.

9-1-2. Screening of Chiral Phosphoric Acid

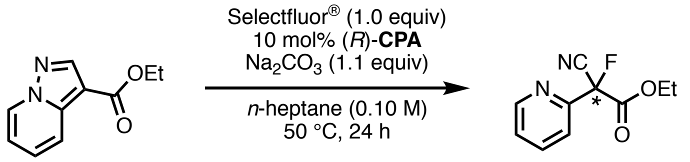

1S
0.20 mmol

2S

entry	CPA	yield of **2S**[a] / %	*ee* of **2S**[b] / %	recovery of **1S**[a] / %
1	(*R*)-**C1**	10	0	85
2	(*R*)-**C2**	15	0	87
3	(*R*)-**C3**	14	1	82
4	(*R*)-**C4**	3	–	77
5	(*R*)-**C5**	3	–	95
6	(*R*)-**C6**	8	2	87

[a] Yields were determined by ^1H NMR analysis using CH_2Br_2 as an internal standard.
[b] *Ee* values were determined by HPLC analysis.

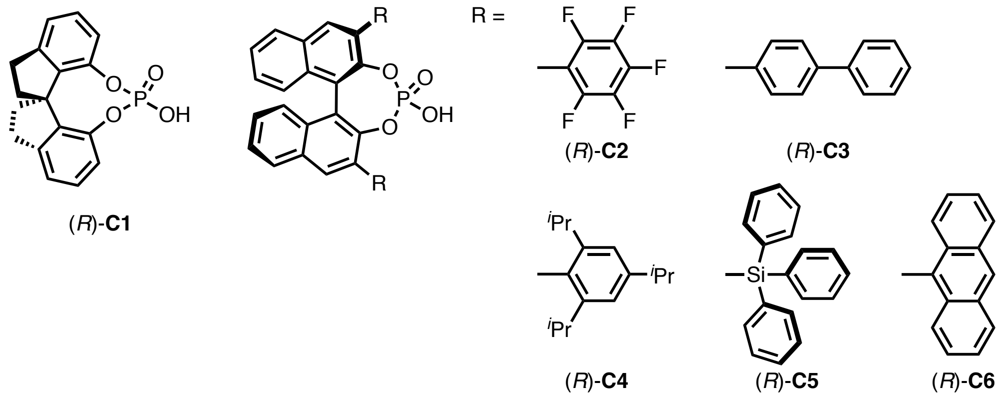

9-2. Decarboxylative Asymmetric Allylation[16]

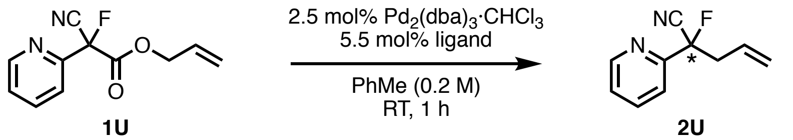

1U

2U

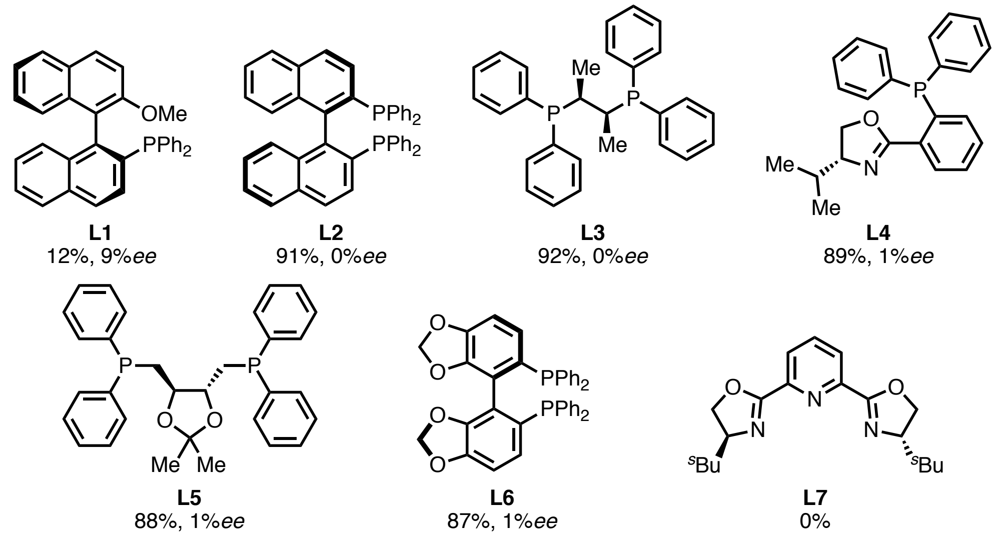

Yields were determined by ^{19}F NMR analysis using PhF as an internal standard.

9-3. Using Cinchona Alkaloids

9-3-1. Selection of Substrate

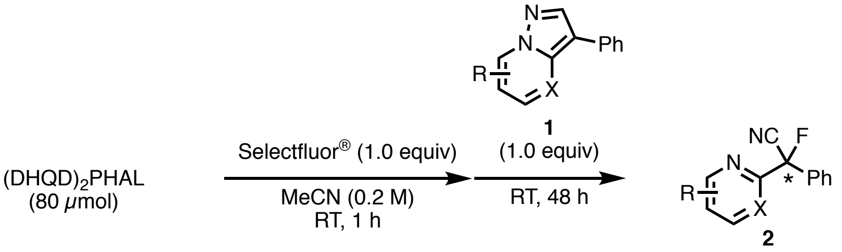

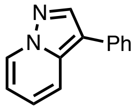

1A
71%, –10%*ee*

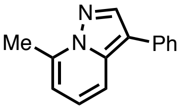

1AC
59%, 10%*ee*

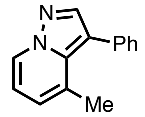

1AD
77%, 12%*ee*

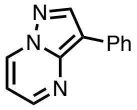

1AG
32%, 38%*ee*

44%, 29%*ee*
(50 °C)

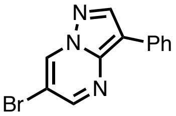

1AJ
0%

11%, 36%*ee*
(50 °C)

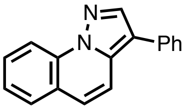

1AP
83%, 5%*ee*

Yields were determined by ^{1}H NMR using CH_2Br_2 as an internal standard.
Ee values were determined by HPLC analysis.

9-3-2. Screening of Cinchona Alkaloid

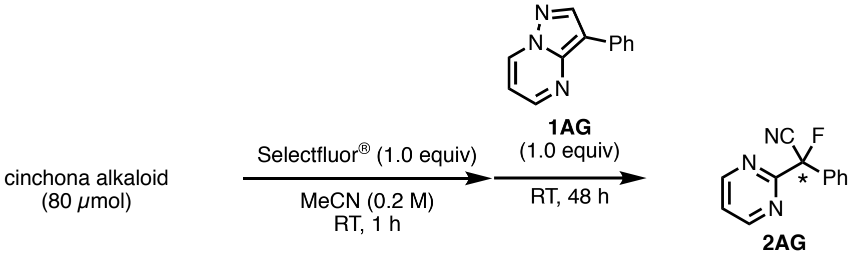

2AG

entry	cinchona alkaloid	yield of **2AG**[a] / %	*ee* of **2AG**[b] / %	recovery of **1AG**[a] / %
1	**N1**	32	38	70
2	**N2**	46	10	70
3	**N3**	quant.	–6	0
4	**N4**	76	4	34
5	**N5**	44	–9	70
6	**N6**	85	–20	17
7	**N7**	26	17	73
8	**N8**	72	24	17
9	**N9**	75	12	12

[a] Yields were determined by ^{1}H NMR using CH_2Br_2 as an internal standard.
[b] *Ee* values were determined by HPLC analysis.

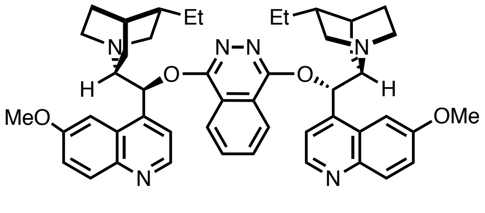

N1
(DHQD)$_2$PHAL

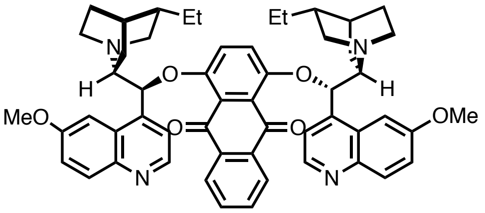

N2
(DHQD)$_2$AQN

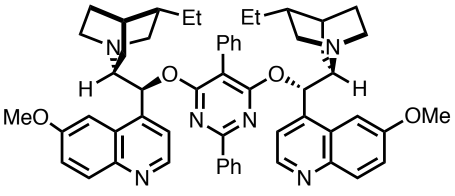

N3
(DHQD)$_2$Pyr

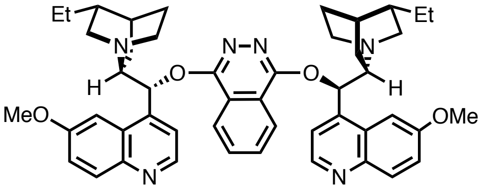

N4
(DHQ)$_2$PHAL

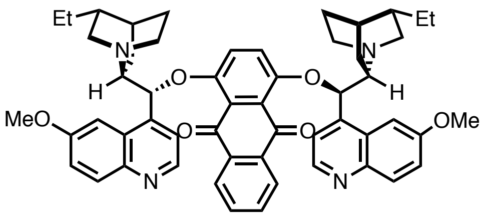

N5
(DHQ)$_2$AQN

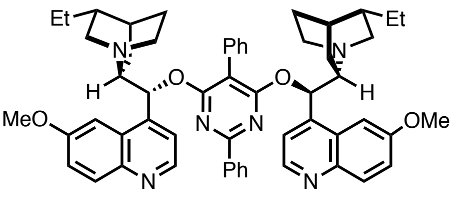

N6
(DHQ)$_2$Pyr

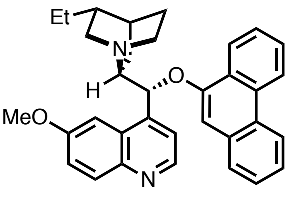

N7

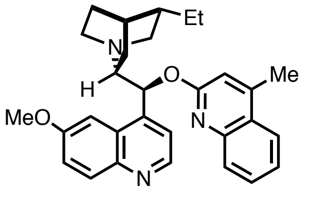

N8

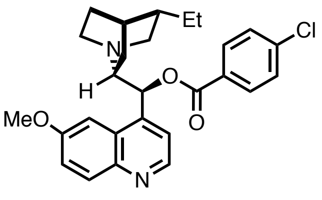

N9

9-3-3. Effect of Solvent

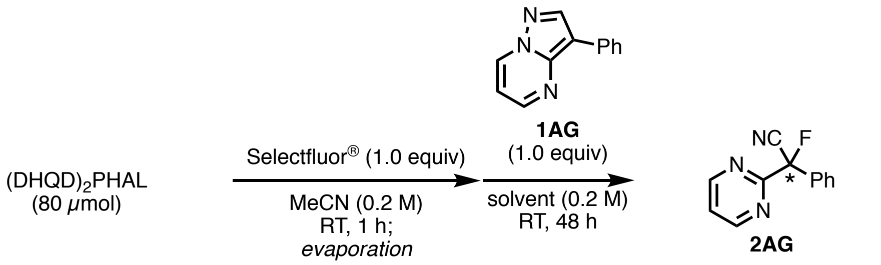

entry	solvent	yield of **2AG**[a] / %	*ee* of **2AG**[b] / %	recovery of **1AG**[a] / %
1	MeOH	trace	–	49
2	DMF	16	53	69
3	MeCN (no substitution)	32	38	70
4	acetone	11	40	43
5	CH_2Cl_2	17	11	57
6	$CHCl_3$	22	–8	74
7	EtOAc	12	40	76
8	CPME	16	41	65
9	THF	9	25	87
10	1,4-dioxane	13	37	50
11	Et_2O	20	42	60
12	PhF	7	49	93
13	*m*-xylene	24	41	66
14	PhMe	28	38	50

[a] Yields were determined by ^{1}H NMR analysis using CH_2Br_2 as an internal standard.
[b] *Ee* value were determined by HPLC analysis.

9-3-4. Effect of Fluorinating Agent

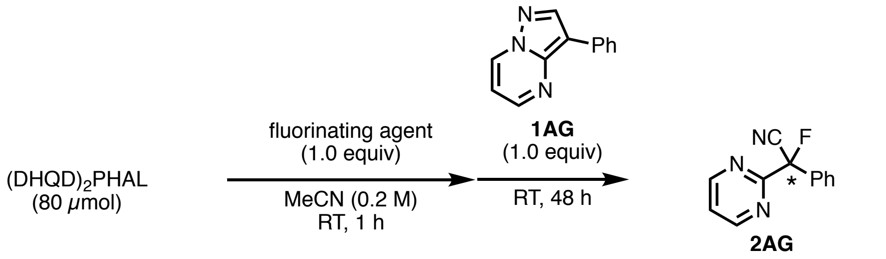

entry	fluorinationg agent	yield of **2AG**[a] / %	*ee* of **2AG**[b] / %	recovery of **1AG**[a] / %
1	Selectfluor®	32	38	70
2	NFSI	52	35	51
3[c]	NFSI	3	17	87

[a] Yields were determined by ^{1}H NMR analysis using CH_2Br_2 as an internal standard.
[b] *Ee* value was determined by HPLC analysis.
[c] 10 mol% of $(DHQD)_2PHAL$ was used.

9-3-5. Condition Optimization

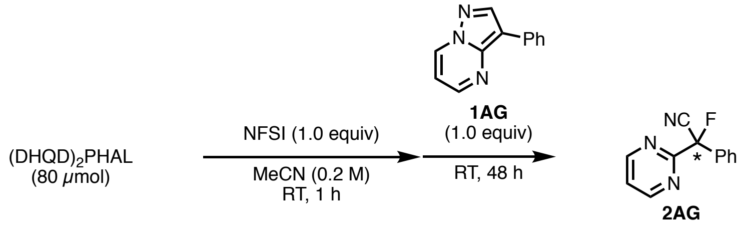

entry	variation from the standard conditions	yield of **2AG**[a] / %	*e.e.* of **2AG**[b] / %	recovery of **1AG**[a] / %
1	none	30	50	67
2	DMF (0.16 M)	38	49	61
3	0 °C for 48 h then RT for 48 h	11	70	64
4	0 °C for 48 h then 50 °C for 48 h	23	45	54
5	DMF (0.16 M), 0 °C for 48 h then RT for 48 h	41	68	59

[a] Yields were determined by ^1H NMR analysis using CH_2Br_2 as an internal standard.
[b] *Ee* values were determined by HPLC analysis.

9-3-6. Asymmetric Ring-Opening Fluorination

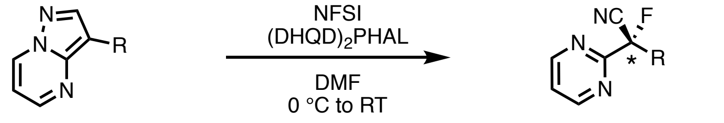

1 **2**

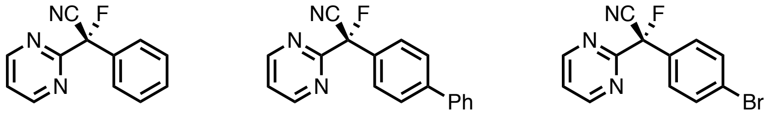

2AG 41%, *e.r.* = 84:16

2AH 24%, *e.r.* = 82:18

2AI 42%, *e.r.* = 70:30 (RT, 48 h)

E.r. values were determined by HPLC analysis.

General Procedure

An 8-mL glass tube equipped with a screw cap containing a magnetic stirring bar was dried with a heat-gun *in vacuo* and filled with N_2 gas after cooling to room temperature. To this tube were added NFSI (25.2 mg, 80.0 μmol, 1.0 equiv), $(DHQD)_2PHAL$ (62.3 mg, 80.0 μmol, 1.0 equiv), DMF (0.25 mL) at room temperature. After the mixture was stirred for 1 h, a solution pyrazolopyrimidizine **1** (80 μmol, 1.0 equiv) in DMF (0.25 mL) was added at 0 °C. The mixture was further stirred at 0 °C for 48 h, and then warmed to room temperature. After the mixture was stirred for another 48 h, H_2O was added. The mixture was extracted Et_2O and washed with brine. The combined organic layer was dried over Mg_2SO_4, filtered, and concentrated *in vacuo*. The residue was purified by PTLC (chloroform/EtOAc = 8:1) to afford the corresponding product **2**. The enantiomer ratio of **2** was determined by chiral HPLC analysis.

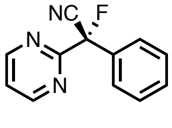

Purification by PTLC (chloroform/EtOAc = 19:1) afforded **2AG** (7.0 mg, 41% yield) as a colorless solid. *e.r.* = 84:16 [Daicel Chiralcel® OZ-H, *n*-hexane/2-propanol = 94:6, 40 °C, 0.7 mL/min, λ = 254 nm, *t* (minor) = 16.84 min, *t* (major) = 18.77 min].

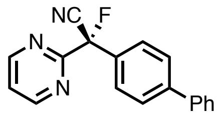

Purification by PTLC (chloroform/EtOAc = 19:1) afforded **2AH** (5.6 mg, 24% yield) as a colorless solid. *e.r.* = 82:18 [Daicel Chiralcel® OZ-H, *n*-hexane/2-propanol = 98:2, 30 °C, 0.7 mL/min, λ = 254 nm, *t* (minor) = 15.62 min, *t* (major) = 18.34 min]

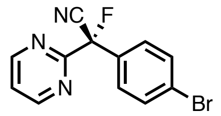

Reaction was performed at room temperature for 48 h. Purification by PTLC (chloroform/EtOAc = 19:1) afforded **2AI** (9.8 mg, 42% yield) as a colorless solid. *e.r.* = 70:30 [Daicel Chiralcel® OZ-H, *n*-hexane/2-propanol = 98:2, 30 °C, 1.2 mL/min, λ = 254 nm, *t* (minor) = 17.21 min, *t* (major) = 20.27 min]

Hydrolysis of 2AI for determining the absolute configuration

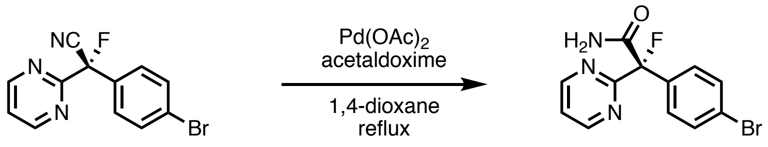

To an 8-mL glass tube containing a magnetic stirring bar and 2-(4-bromophenyl)-2-fluoro-2-(pyrimidin-2-yl)acetonitrile (**2AI**: 9.8 mg, 33.6 μmol, 1.0 equiv, *e.r.* = 70:30) were added $Pd(OAc)_2$ (0.30 mg, 1.34 μmol, 4.0 mol%), acetaldoxime (21 μL, 0.336 mmol, 10 equiv), and 1,4-dioxane (0.34 mL). After the mixture was stirred with refluxing for 1 h, the crude mixture was concentrated *in vacuo*, and the resulting residue was purified by PTLC (chloroform/EtOAc = 9:1) to afford 2-(4-bromophenyl)-2-fluoro-2-(pyrimidin-2-yl)acetamide (**10**: 10.4 mg, quant.) as a white solid. $[\alpha]_D^{20}$ = –158.0° (*c* = 1.0, acetone), *e.r.* = 70:30 [Daicel Chiralpak® OD-3, *n*-hexane/2-propanol = 65:35, 30 °C, 1.2 mL/min, λ = 230 nm, *t* (minor) = 7.94 min, *t* (major) = 12.37 min]; ^{1}H NMR (400 MHz, $CDCl_3$) δ 8.83 (d, *J* = 4.8 Hz, 2H), 7.58–7.53 (m, 4H), 7.33 (td, *J* = 4.8, 1.2 Hz, 1H), 6.79 (brs, 1H), 6.08 (brs, 1H); ^{13}C NMR (101 MHz, $CDCl_3$) δ 170.2 (d, $J_{C\text{–}F}$ = 24.2 Hz), 165.3 (d, $J_{C\text{–}F}$ = 22.3 Hz), 157.6, 134.8 (d, $J_{C\text{–}F}$ = 24.2 Hz), 131.2, 128.2 (d, $J_{C\text{–}F}$ = 9.7 Hz), 123.5, 121.0, 98.2 (d, $J_{C\text{–}F}$ = 194.9 Hz); ^{19}F NMR (376 MHz, $CDCl_3$) δ –146.9; HRMS (ESI) *m*/*z* calcd for $C_{12}H_{10}BrFN_2O$ $[M+H]^+$: 309.9986 found 309.9983.

Major enantiomer of **10** was isolated by HPLC using chiral stationary phase column [Daicel Chiralpak® OD-3, *n*-hexane/2-propanol = 65:35, 30 °C, 1.2 mL/min, λ = 254 nm, *t* (minor) = 7.94 min, *t* (major) = 12.37 min] to afford the major enantiomer. This enantiomer was recrystallized to determine the absolute configuration.

9-3-7. HPLC spectra

HPLC spectra of 2AG

Chromatogram Report

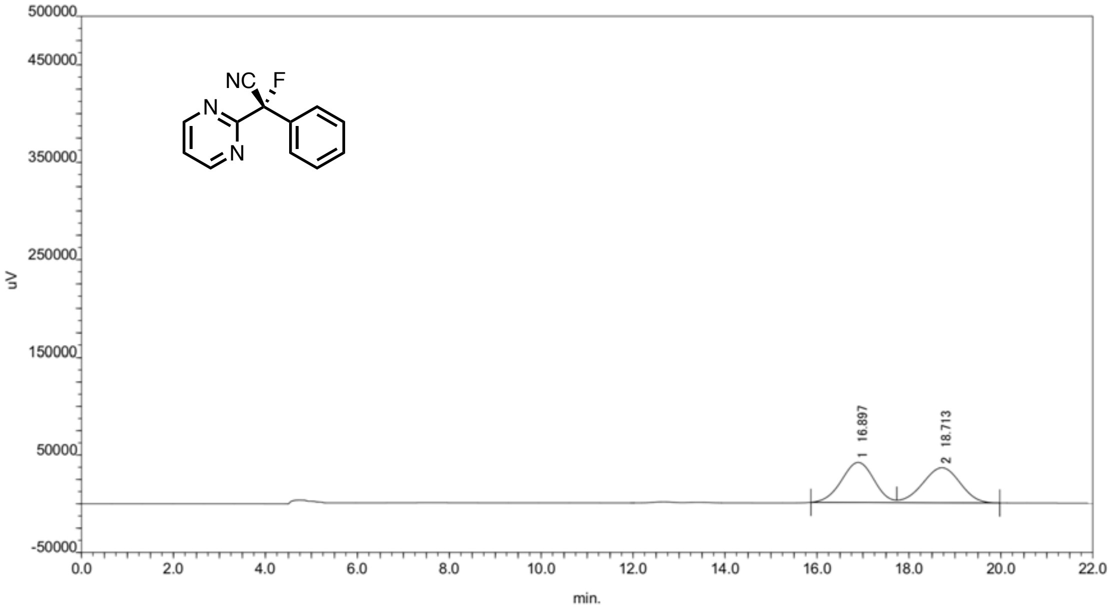

Result

No.	Rt (min)	Area	Area (%)	height
1	16.897	2022731.8	49.9	41175
2	18.713	2030747.1	50.1	35984
		4053478.9	100.0	77159

Chromatogram Report

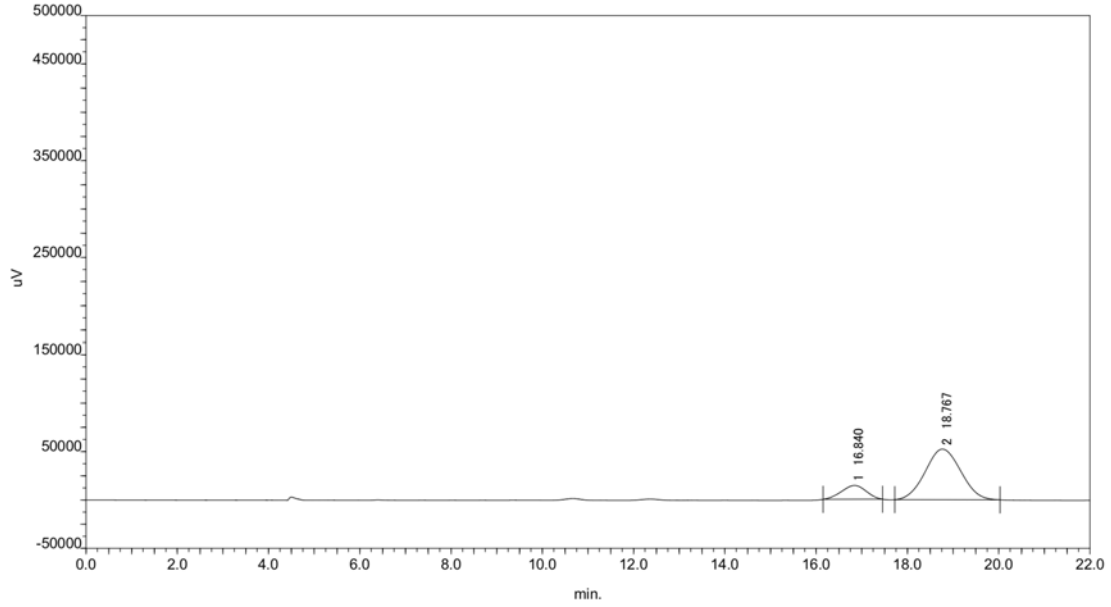

Result

No.	Rt (min)	Area	Area (%)	height
1	16.840	537769.4	16.0	14318
2	18.767	2827995.9	84.0	52327
		3365765.3	100.0	66645

HPLC spectra of 2AH

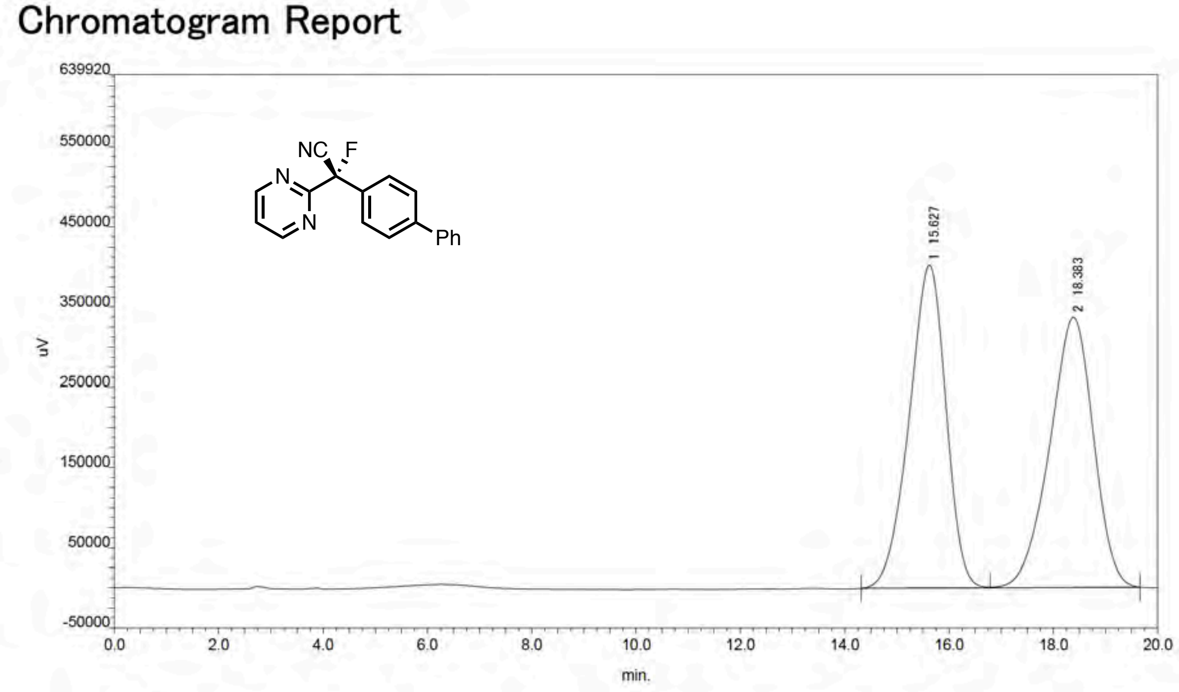

Result

No.	Rt(min)	Area	Area(%)	height
1	15.627	19706802.3	50.1	402559
2	18.383	19622431.1	49.9	337191
		39329233.4	100.0	739750

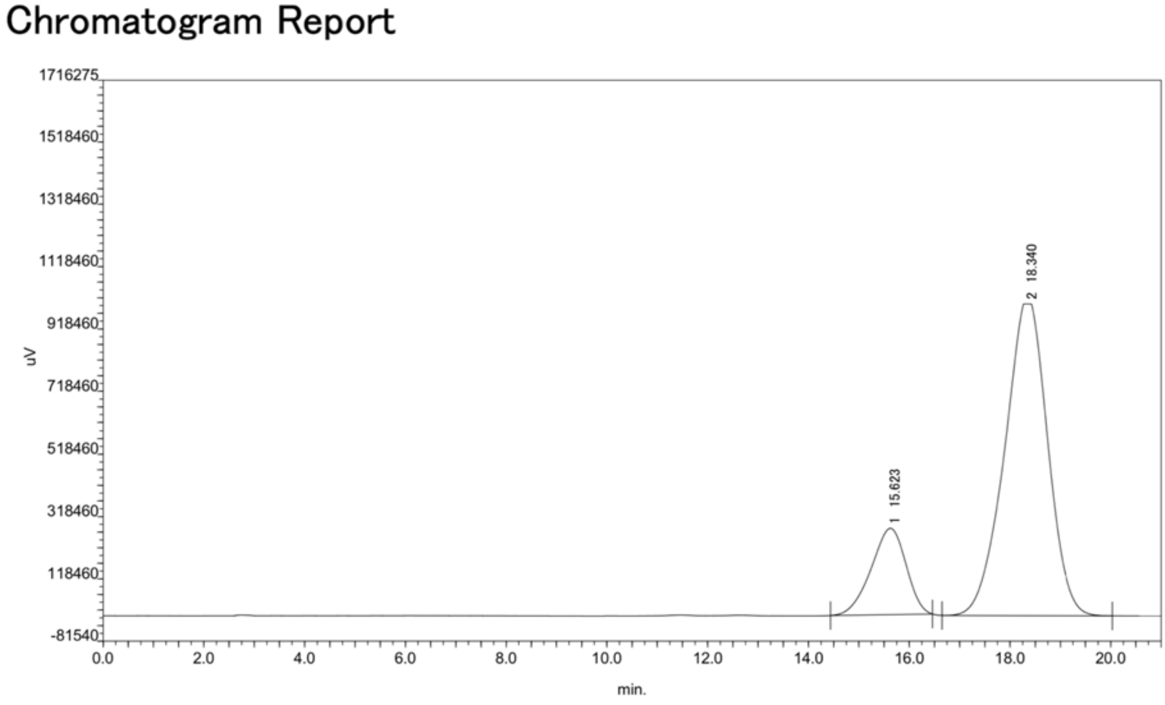

Result

No.	Rt(min)	Area	Area(%)	height
1	15.623	13342387.2	18.2	277046
2	18.340	59842551.2	81.8	998269
		73184938.4	100.0	1275315

HPLC spectra of 2AI

Chromatogram Report

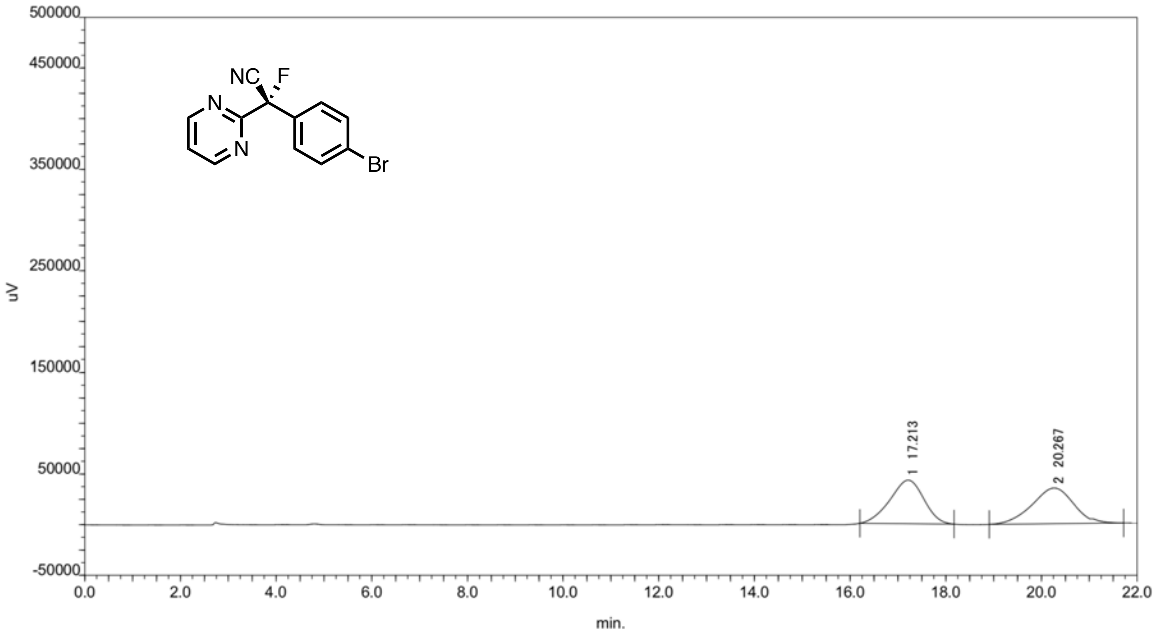

Result

No.	Rt(min)	Area	Area(%)	height
1	17.213	2123158.6	49.9	42980
2	20.267	2127784.0	50.1	35199
		4250942.6	100.0	78179

Chromatogram Report

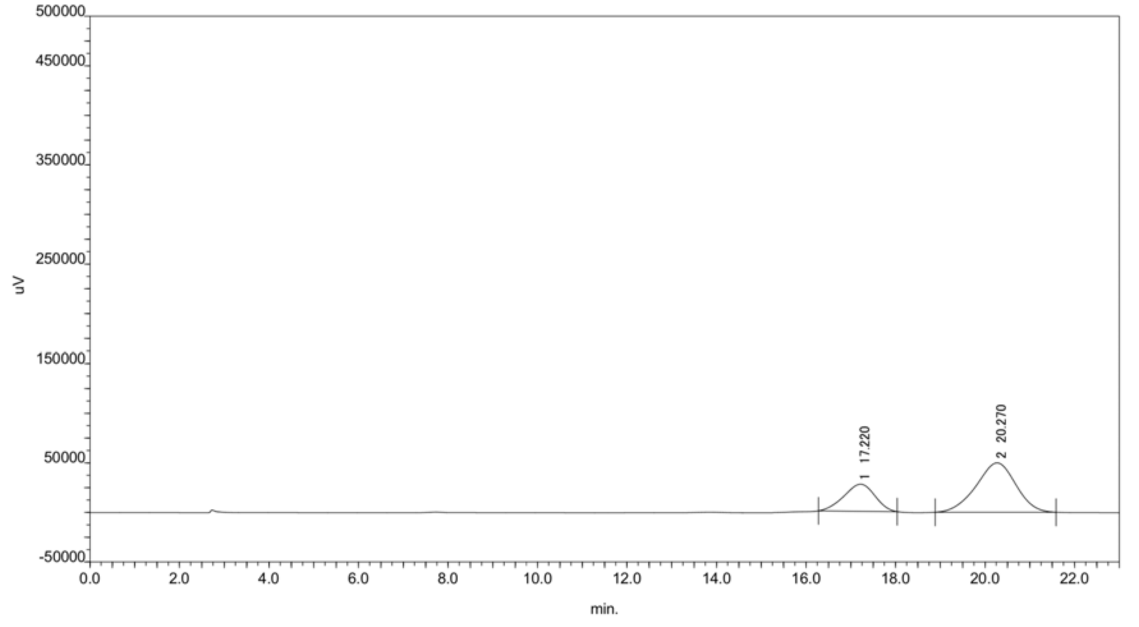

Result

No.	Rt(min)	Area	Area(%)	hight
1	17.220	1314304.2	29.9	27337
2	20.270	3083421.2	70.1	49855
		4397725.4	100.0	77192

HPLC spectra of 10

Chromatogram Report

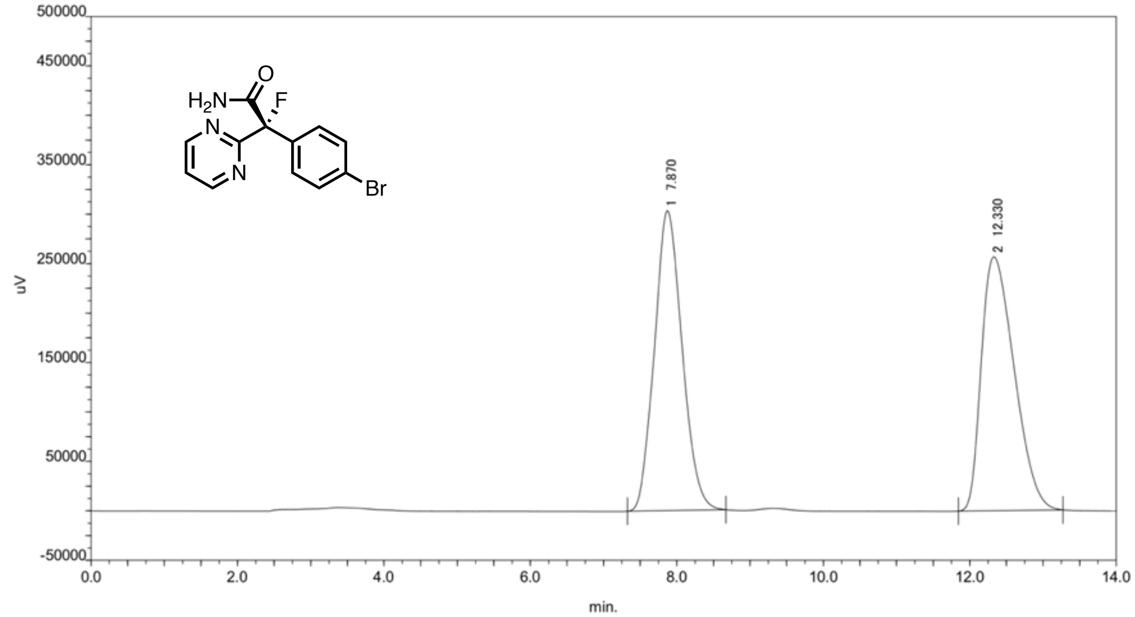

Result

No.	Rt(min)	Area	Area(%)	height
1	7.870	7970642.6	49.9	303290
2	12.330	8010456.2	50.1	256778
		15981098.8	100.0	560068

Chromatogram Report

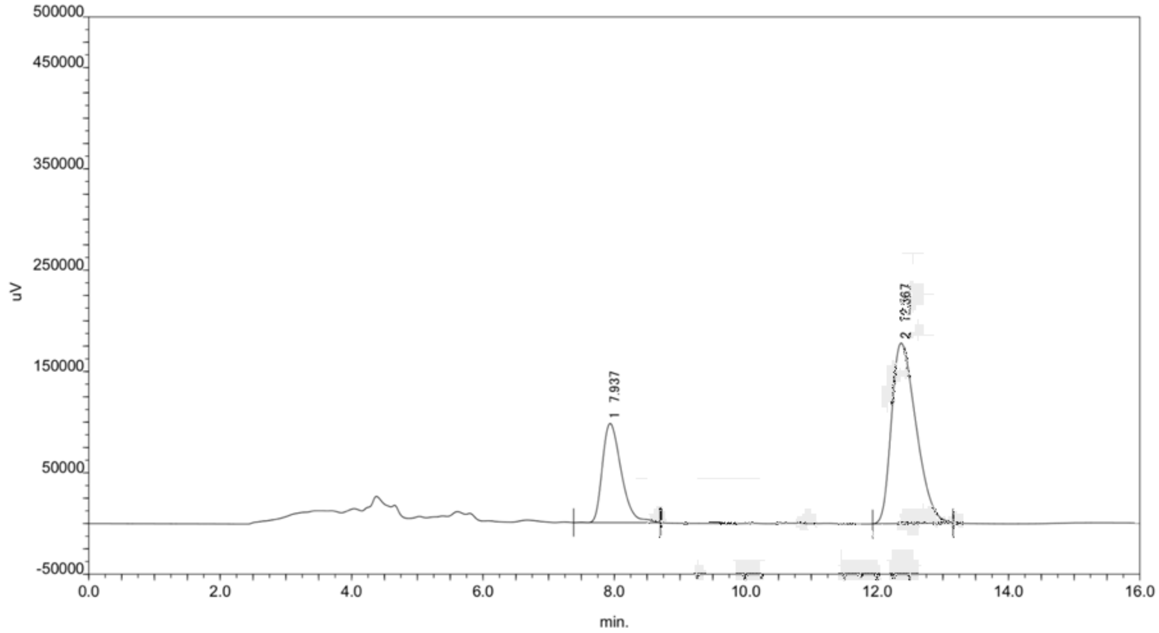

Result

No.	Rt(min)	Area	Area(%)	height
1	7.937	1948607.6	29.6	97824
2	12.367	4634092.8	70.4	178009
		6582700.4	100.0	275833

9-3-8. X-ray Crystal Structure Analysis of (*S*)-10

A crystal of (*S*)-**10** was grown from a 2-propanol/H_2O (vapor diffusion). A suitable crystal was mounted with Paratone oil on a MiTeGen MicroMounts and transferred to the 3-axis Eulerian Goniometer of a Rigaku R-AXIS RAPID II system with Ultrax 18 kW rotating anode X-ray generator using graphite-monochromated Cu-K_α radiation and imaging plate area detector. Cell parameters were determined and refined, and raw frame data were integrated using RAPID-AUTO (RIGAKU, 1998). The structures were solved by direct methods with (SHELXT)[17] and refined by full-matrix least-squares techniques against F^2 (SHELXL-2018/3)[18] by using Olex2 software package.[19] The intensities were corrected for Lorentz and polarization effects. The non-hydrogen atoms were refined anisotropically. Hydrogen atoms were placed using AFIX instructions.

Table S1. Crystallographic Data and Structure Refinement Details for (*S*)-**10**

**Compound**	(*S*)-**10**
CCDC number	2113745
Empirical formula	$C_{12}H_9BrFN_3O$
Formula weight	310.13
T / K	173(2)
Crystal system	monoclinic
Space group	$P2_1$
a / Å	8.0916(6)
b / Å	6.0738(5)
c / Å	12.9097(10)
α / °	90
β / °	105.671(7)
γ / °	90
V / Å^3	610.89(9)
Z	2
D_{calc}, /g cm^{-3}	1.686
μ / mm^{-1}	4.651
F(000)	308.0
Crystal size / mm	0.4 × 0.4 × 0.3
λ / Å	1.5418
2θ range / °	7.112 to 136.456
Reflns collected	7095
Indep reflns/R_{int}	2127/0.0427
Params	163
GOF on F^2	1.148
R_1, wR_2 [I>2σ(I)]	0.0323, 0.0846
R_1, wR_2 [all data]	0.0324, 0.0847
Max./Mini. Peak / e Å^{-3}	0.41/–1.05
Flack parameter	0.031(11)

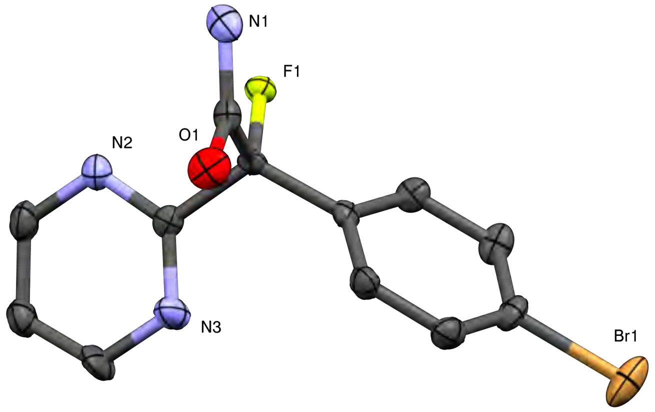

Figure S1. ORTEP drawing of (*S*)-**10** with 50% thermal ellipsoid. All hydrogen atoms are omitted for clarity.

10. Derivatization of Products

10-1. Derivatization of 2U

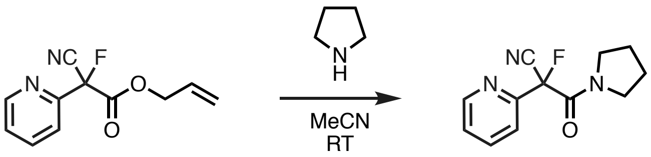

To an 8-mL glass tube equipped containing a magnetic stirring bar and **2U** (44.0 mg, 0.200 mmol, 1.0 equiv) were added pyrrolidine (71.8 mg, 1.00 mmol, 5.0 equiv) and MeCN (1.0 mL). The mixture was stirred at room temperature for 12 h. The mixture was concentrated *in vacuo*. The residue was purified by PTLC (chloroform /EtOAc = 9:1) to afford 2-fluoro-2-(pyridine-2-yl)-2-(pyrrolidin-1-yl)acetonitrile (**11**: 19.1 mg, 41% yield) as a white solid. ^{1}H NMR (400 MHz, $CDCl_3$) δ 8.68 (d, J = 4.8 Hz, 1H), 7.88 (td, J = 8.0, 2.0 Hz, 1H), 7.73 (d, J = 8.0 Hz, 1H), 7.45–7.42 (m, 1H), 3.68–3.53 (m, 4H), 1.97–1.81 (m, 4H); ^{13}C NMR (101 MHz, $CDCl_3$) δ 159.7 (d, $J_{C–F}$ = 23.3 Hz), 151.5 (d, $J_{C–F}$ = 23.2 Hz), 149.8, 137.8, 125.4, 121.5 (d, $J_{C–F}$ = 3.8 Hz), 114.3 (d, $J_{C–F}$ = 34.9 Hz), 90.0 (d, $J_{C–F}$ = 197.9 Hz), 48.2, 47.0 (d, $J_{C–F}$ = 6.9 Hz), 26.5, 23.2; ^{19}F NMR (376 MHz, $CDCl_3$) δ –143.4; HRMS (ESI) *m*/*z* calcd for $C_{12}H_{13}FN_3O$ $[M+H]^+$: 234.1037 found 234.1036.

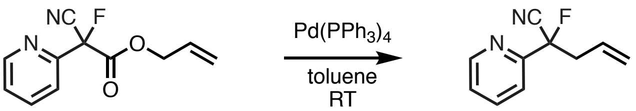

An 8-mL glass tube equipped with a screw cap containing a magnetic stirring bar was dried with a heat-gun *in vacuo* and filled with N_2 gas after cooling to room temperature. To this tube were added **2U**

(22.0 mg, 0.100 mmol, 1.0 equiv) and $Pd(PPh_3)_4$ (5.78 mg, 5.00 μmol, 5.0 mol%). The tube was placed under vacuum and refilled with N_2 gas three times. To this was added toluene (1.0 mL). The tube was sealed with a screw cap and then stirred at room temperature for 1 h. Then the mixture was passed through a short silica-gel pad with EtOAc as an eluent. The filtrate was concentrated *in vacuo*. The residue was purified by PTLC (chloroform/EtOAc = 9:1) to afford 2-fluoro-2-(pyridine-2-yl)pent-4-enenitrile (**12**: 35.2 mg, quant.) as a colorless oil. ^{1}H NMR (400 MHz, $CDCl_3$) δ 8.67 (d, J = 4.4 Hz, 1H), 7.83 (td, J = 8.0, 2.0 Hz, 1H), 7.58 (d, J = 8.0 Hz, 1H), 7.37 (dd, J = 8.0, 4.4 Hz, 1H), 5.86–5.76 (m, 1H), 5.30–5.26 (m, 2H), 3.22–2.99 (m, 2H); ^{13}C NMR (101 MHz, $CDCl_3$) δ 154.0 (d, J_{C-F} = 26.2 Hz), 149.6, 137.4, 128.4 (d, J_{C-F} = 2.9 Hz), 124.6, 122.1, 119.6 (d, J_{C-F} = 6.8 Hz), 116.5 (d, J_{C-F} = 33.9 Hz), 91.2 (d, J_{C-F} = 188.2 Hz), 43.8 (d, J_{C-F} = 24.2 Hz); ^{19}F NMR (376 MHz, $CDCl_3$) δ –155.1 (t, J_{H-F} = 23.5 Hz); HRMS (ESI) *m/z* calcd for $C_{10}H_{10}FN_2$ [M+H]$^+$: 177.0823 found 177.0823.

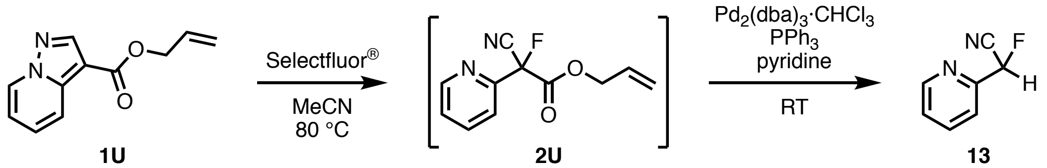

1U **2U** **13**

An 8-mL glass tube equipped with a screw cap containing a magnetic stirring bar was dried with a heat-gun *in vacuo* and filled with N_2 gas after cooling to room temperature. To this tube were added **1U** (37.6 mg, 0.200 mmol, 1.0 equiv) and Selectfluor® (70.8 mg, 0.200 mmol, 1.0 equiv). The tube was placed under vacuum and refilled N_2 gas three times. To this was added MeCN (1.0 mL). The tube was sealed with a screw cap and then heated at 80 °C for 24 h with stirring. After cooling the reaction mixture to room temperature, pyridine (48.3 μL, 0.600 mmol, 3.0 equiv) was added. After this mixture was stirred for 10 min, $Pd_2(dba)_3{\cdot}CHCl_3$ (10.4 mg, 10.0 μmol, 5.0 mol%), PPh_3 (10.5 mg, 40.0 μmol, 20 mol%) were added. The mixture was further stirred at room temperature for 1 h, passed through a short silica-gel pad with EtOAc as an eluent, and then concentrated *in vacuo*. The residue was purified by PTLC (hexane/EtOAc = 8:1) to afford 2-fluoro-2-(pyridine-2-yl)acetonitrile (**13**: 25.0 mg, 92% yield) as a colorless oil. ^{1}H NMR (400 MHz, $CDCl_3$) δ 8.69 (d, J = 4.0 Hz, 1H), 7.88 (td, J = 7.6, 1.6 Hz, 1H), 7.63 (d, J = 7.6 Hz, 1H), 7.47–7.40 (m, 1H), 6.18 (d, J_{H-F} = 46.4 Hz, 1H); ^{13}C NMR (101 MHz, $CDCl_3$) δ 150.7 (d, J_{C-F} = 23.1 Hz), 150.0, 137.7, 125.3, 121.1 (d, J_{C-F} = 3.8 Hz), 114.6 (d, J_{C-F} = 31.8 Hz), 80.5 (d, J_{C-F} = 184.8 Hz); ^{19}F NMR (376 MHz, $CDCl_3$) δ –182.0 (d, J_{F-H} = 46.2 Hz); HRMS (ESI) *m/z* calcd for $C_7H_6FN_2$ [M+H]$^+$: 137.0510 found 137.0511.

10-2. Derivatization of 2A

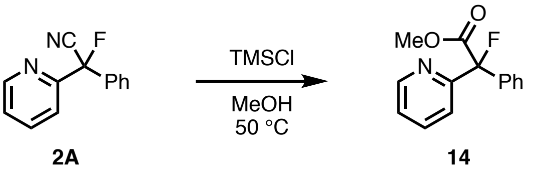

2A **14**

An 8-mL glass tube equipped with a screw cap containing a magnetic stirring bar was dried with a heat-gun *in vacuo* and filled with N_2 after cooling to room temperature. To this tube were added **2A** (42.5 mg, 0.200 mmol, 1.0 equiv), chlorotrimethylsilane (130 µL, 1.00 mmol, 5.0 equiv), and MeOH (1.0 mL). The tube was sealed with a screw cap and then heated at 50 °C for 6 h with stirring. The reaction was quenched with $NaHCO_3$ aq. and the mixture was extracted with EtOAc and washed with brine. The combined organic layer was dried over Na_2SO_4, filtered, and concentrated *in vacuo*. The residue was purified by PTLC (hexane/EtOAc = 5:1) to afford methyl 2-fluoro-2-phenyl-2-(pyridine-2-yl)acetate (**14**: 40.1 mg, 82% yield) as a colorless oil. ^{1}H NMR (400 MHz, $CDCl_3$) δ 8.66 (dd, J = 5.2, 1.2 Hz, 1H), 7.72 (td, J = 8.0, 1.2 Hz, 1H), 7.55–7.51 (m, 2H), 7.41–7.37 (m, 4H), 7.31 (ddd, J = 8.0, 5.2, 0.8 Hz, 1H), 3.88 (s, 3H); ^{13}C NMR (101 MHz, $CDCl_3$) δ 169.3 (d, $J_{C–F}$ = 27.1 Hz), 157.7 (d, $J_{C–F}$ = 24.2 Hz), 149.0, 137.0, 136.9 (d, $J_{C–F}$ = 23.2 Hz), 128.9, 128.1, 126.5 (d, $J_{C–F}$ = 7.8 Hz), 123.8, 121.9 (d, $J_{C–F}$ = 4.8 Hz), 97.8 (d, $J_{C–F}$ = 190.1 Hz), 53.2; ^{19}F NMR (376 MHz, $CDCl_3$) δ –147.3; HRMS (ESI) *m*/*z* calcd for $C_{14}H_{13}FNO_2$ $[M+H]^+$: 246.0925 found 246.0924.

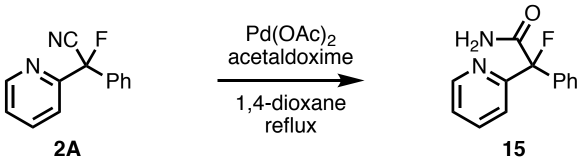

To an 8-mL glass tube containing a magnetic stirring bar and **2A** (41.9 mg, 0.200 mmol, 1.0 equiv) were added $Pd(OAc)_2$ (1.80 mg, 8.00 µmol, 4.0 mol%), acetaldoxime (120 µL, 2.00 mmol, 10 equiv), and 1,4-dioxane (2.0 mL). After the mixture was stirred with refluxing for 1 h, the crude mixture was concentrated *in vacuo*, and the resulting residue was purified by Isolera® (hexane/EtOAc = 4:1 to 1:1) to afford 2-fluoro-2-phenyl-2-(pyridine-2-yl)acetamide (**15**: 42.4 mg, 92% yield) as a white solid. ^{1}H NMR (400 MHz, $CDCl_3$) δ 8.68 (dd, J = 4.8, 0.8 Hz, 1H), 7.74 (t, J = 7.6 Hz, 1H), 7.60–7.58 (m, 2H), 7.48 (dd, J = 7.6, 0.8 Hz, 1H), 7.42–7.36 (m, 3H), 7.32 (ddd, J = 7.6, 4.8, 0.8 Hz, 1H), 7.26 (brs, 1H), 5.77 (brs, 1H); ^{13}C NMR (101 MHz, $CDCl_3$) δ 171.0 (d, $J_{C–F}$ = 24.0 Hz), 157.4 (d, $J_{C–F}$ = 24.0 Hz), 149.0, 137.4 (d, $J_{C–F}$ = 22.1 Hz), 137.0, 129.0, 128.3, 126.5 (d, $J_{C–F}$ = 7.7 Hz), 123.7, 122.1 (d, $J_{C–F}$ = 6.8 Hz), 97.8 (d, $J_{C–F}$ = 188.7 Hz); ^{19}F NMR (376 MHz, $CDCl_3$) δ –144.8; HRMS (ESI) *m*/*z* calcd for $C_{13}H_{11}FN_2O$ $[M+H]^+$: 231.0928 found 231.0927.

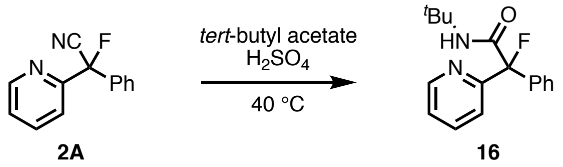

To an 8-mL glass tube containing a magnetic stirring bar and **2A** (42.4 mg, 0.200 mmol, 1.0 equiv) were added *tert*-butyl acetate (161 µL, 1.20 mmol, 6.0 equiv) and conc. H_2SO_4 (10 µL, 0.200 mmol, 1.0 equiv). The mixture was stirred at 40 °C for 2 h. After cooling to room temperature, the mixture was

poured into cold $NaHCO_3$ aq. to neutralize and extracted with EtOAc. The combined organic layer was dried over Na_2SO_4, filtered, and concentrated *in vacuo*. The residue was purified by PTLC (hexane/EtOAc = 4:1) to afford *N*-(*tert*-butyl)-2-fluoro-2-phenyl-2-(pyridine-2-yl)acetamide (**16**: 53.8 mg, 94% yield) as a white solid. ^{1}H NMR (400 MHz, $CDCl_3$) δ 8.65 (d, J = 4.4 Hz, 1H), 7.69 (td, J = 7.6, 1.6 Hz, 1H), 7.59–7.51 (m, 2H), 7.44 (dd, J = 7.6, 1.2 Hz, 1H), 7.40–7.36 (m, 3H), 7.29–7.25 (m, 1H), 7.05 (brs, 1H), 1.40 (s, 9H); ^{13}C NMR (101 MHz, $CDCl_3$) δ 167.3 (d, $J_{C–F}$ = 22.1 Hz), 157.9 (d, $J_{C–F}$ = 23.1 Hz), 148.9, 138.2 (d, $J_{C–F}$ = 22.2 Hz), 136.7, 128.8, 128.2, 126.7 (d, $J_{C–F}$ = 7.7 Hz), 123.4, 122.4 (d, $J_{C–F}$ = 4.8 Hz), 97.6 (d, $J_{C–F}$ = 190.7 Hz), 51.6, 28.6; ^{19}F NMR (376 MHz, $CDCl_3$) δ –144.0; HRMS (ESI) *m*/*z* calcd for $C_{17}H_{20}FN_2O$ [M+H]$^+$: 287.1554 found 287.1552.

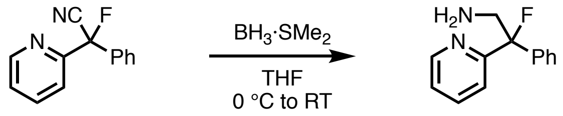

2A **17**

To a solution of **2A** (42.4 mg, 0.200 mmol, 1.0 equiv) in THF (1.0 mL) was added $BH_3·SMe_2$ (57.0 μL, 0.600 mmol, 3.0 equiv) dropwise at 0 °C. After stirring the mixture at room temperature for 19 h the reaction was quenched with MeOH carefully at 0 °C and concentrated *in vacuo*. To this residue were added MeOH (1.0 mL) and adjusted to pH = 2 with conc. HCl aq. at 0 °C. After stirring the mixture at room temperature for 12 h, solvent was removed *in vacuo*. The residue was adjusted to pH = 8 with $NaHCO_3$ aq., extracted with EtOAc, and washed with brine. The combined organic layer was dried over Na_2SO_4, filtered, and concentrated *in vacuo*. The residue was purified by PTLC (chloroform/EtOAc = 8:1) to afford 2-fluoro-2-phenyl-2-(pyridine-2-yl)ethan-1-amine (**17**: 17.7 mg, 41% yield) as a colorless oil. ^{1}H NMR (400 MHz, $CDCl_3$) δ 8.62 (dd, J = 4.8, 1.2 Hz, 1H), 7.72–7.67 (m, 1H), 7.56 (dd, J = 7.6, 1.2 Hz, 1H), 7.51–7.48 (m, 2H), 7.34 (t, J = 7.6 Hz, 2H), 7.29–7.25 (m, 1H), 7.22–7.19 (m, 1H), 3.75 (dd, $J_{H–F}$ = 26.8 Hz, J = 14.4 Hz, 1H), 3.47 (dd, $J_{H–F}$ = 20.0 Hz, J = 14.4 Hz, 1H), 1.46 (brs, 2H); ^{13}C NMR (101 MHz, $CDCl_3$) δ 160.5 (d, $J_{C–F}$ = 27.9 Hz), 148.8, 141.0 (d, $J_{C–F}$ = 23.1 Hz), 137.0, 128.4, 127.9, 125.0 (d, $J_{C–F}$ = 9.6 Hz), 122.7, 121.1 (d, $J_{C–F}$ = 8.7 Hz), 100.6 (d, $J_{C–F}$ = 176.2 Hz), 50.9 (d, $J_{C–F}$ = 23.1 Hz); ^{19}F NMR (376 MHz, $CDCl_3$) δ –166.0 (t, $J_{F–H}$ = 22.6 Hz); HRMS (ESI) *m*/*z* calcd for $C_{13}H_{14}FN_2$ [M+H]$^+$: 217.1136 found 217.1134.

11. References

[1] Alam, K.; Kim, S. M.; Kim, D. J.; Park, J. K. Development of Structurally Diverse *N*-Heterocyclic Carbene Ligands via Palladium-Copper-Catalyzed Decarboxylative Arylation of Pyrazolo[1,5-*a*]pyridine-3-carboxylic Acid. *Adv. Synth. Catal.* **2016**, *358*, 2661–2670.

[2] Wu, H. C.; Chu, J. H.; Li, C. W.; Hwang, L. C.; Wu, M. J. Palladium-Catalyzed Regioselective Arylation of Pyrazolo[1,5-*a*]pyridines via C–H Activation and Synthetic Applications on P38 Kinase Inhibitors. *Organometallics* **2016**, *35*, 288–300.

[3] Ravi, C.; Samanta, S.; Mohan, D. C.; Reddy, N. N. K.; Adimurthy, S. Synthesis of Functionalized Pyrazolo[1,5-*a*]pyridines: [3+2] Cycloaddition of *N*-Aminopyridines and α,β-Unsaturated Carbonyl Compounds/Alkenes at Room Temperature. *Synthesis* **2017**, *49*, 2513–2522.

[4] Balkenhohl, M.; Salgues, B.; Hirai, T.; Karaghiosoff, K.; Knochel, P. Regioselective Metalation and Functionalization of the Pyrazolo[1,5-*a*]pyridine Scaffold Using Mg- and Zn-TMP Bases. *Org. Lett.* **2018**, *20*, 3114–3118.

[5] Bedford, R. B.; Durrant, S. J.; Montgomery, M. Catalyst-Switchable Regiocontrol in the Direct Arylation of Remote C–H Groups in Pyrazolo[1,5-*a*]pyrimidines. *Angew. Chem., Int. Ed.* **2015**, *127*, 8911–8914.

[6] Benchikh, E.; Fitzgerald, S. P.; Lowry, P. A.; Connell, I. R. Immunodetection and Quantification of Pyrazolopyrimidine Sedatives. Patent WO2011007180A1, January 20, **2011**.

[7] Bach Taña, J.; Pages Santacana, L. M.; Taltavull Moll, J.; Eastwood, P. R.; Gonzalez Rodrigues, J.; Giulio Matassa, V. Pyrazole Derivatives as JAK Inhibitors. Patent WO2011101161A1, August 25, **2011**.

[8] Bethel, P. A.; Campbell, A. D.; Goldberg, F. W.; Kemmitt, P. D.; Lamont, G. M.; Suleman, A. Optimized Scale up of 3-Pyrimidinylpyrazolo[1,5-*a*]pyridine via Suzuki Coupling; A General Method of Accessing a Range of 3-(Hetero)arylpyrazolo[1,5-*a*]pyridines. *Tetrahedron* **2012**, *68*, 5434–5444.

[9] Ripka, A.; Shapiro, G.; Mcriner, A. J.; Bursavich, M. G. Imidazotriazinone Compounds as PDE9 Inhibitors and Their Preparation. Patent WO2013142269A1, September 26, **2013**.

[10] Li, J.; Zhang, P.; Jiang, M.; Yang, H.; Zhao, Y.; Fu, H. Visible Light as a Sole Requirement for Intramolecular C(sp^3)-H Imination. *Org. Lett.* **2017**, *19*, 1994–1997.

[11] Stanton, M. G.; Noucti, N.; Sloman, D. J.; Munoz, B.; Lim, J. Pyrazolo[1,5-*a*]pyrimidine Derivatives. Patent WO2009014620A1, January 29, **2009**.

[12] Lindsay-Scott, P. J.; Charlesworth, N. G.; Grozavu, A. A Flexible Strategy for the Regiocontrolled Synthesis of Pyrazolo[1,5-*a*]pyrazines. *J. Org. Chem.* **2017**, *82*, 11295–11303.

[13] Velankar, A. D.; Quintini, G.; Prabhu, A.; Weber, A.; Hunaeus, G.; Voland, B.; Wuest, M.; Orjeda, C.; Harel, D.; Varghese, S.; Gore, V.; Patil, M.; Gayke, D.; Herdemann, M.; Heit, I.; Zaliani, A. Synthesis and Biological Evaluation of Novel (4 or 5-Aryl)pyrazolyl-indoles as Inhibitors of Interleukin-2 Inducible T-Cell Kinase (ITK). *Bioorg. Med. Chem.* **2010**, *18*, 4547–4559.

[14] Luo, Y.; Zhang, H.; Wang, S.; Zhou, Y.; Dong, S.; Feng, X. Asymmetric Catalytic Diverse Ring Opening/Cycloadditions of Cyclobutenones with (*E*)-Alkenyloxindoles and (*E*)-Dioxopyrrolidines. *Org. Lett.* **2020**, *22*, 2645–2650.
[15] Rauniyar, V.; Lackner, A. D.; Hamilton, G. L.; Toste, F. D. Asymmetric Electrophilic Fluorination Using an Anionic Chiral Phase-Transfer Catalyst. *Science* **2011**, *334*, 1681–1684.
[16] Nakamura, M.; Hajra, A.; Endo, K.; Nakamura, E. Synthesis of Chiral α-Fluoroketones Through Catalytic Enantioselective Decarboxylation. *Angew. Chem., Int. Ed.* **2005**, *44*, 7248–7251.
[17] Sheldrick, G. M. SHTLXT – Integrated Space-Group and Crystal-Structure Determination. *Acta Cryst.* **2015**, *A71*, 3–8.
[18] Sheldrick, G. M. Crystal Structure Refinement with SHELXL. *Acta Cryst.* **2015**, C*71,* 3–8.
[19] Dolomanov, O. V.; Bourhis, L. J.; Gildea, R. J.; Howard, J. A. K.; Puschmann, H. OLEX2: A Complete Structure Solution, Refinement and Analysis Program. *J. Appl. Crystallogr.* **2009**, *42,* 339–341.

12. ^{1}H, ^{13}C, and ^{19}F NMR Spectra

^{1}H NMR of **1D** (400 MHz, $CDCl_3$)

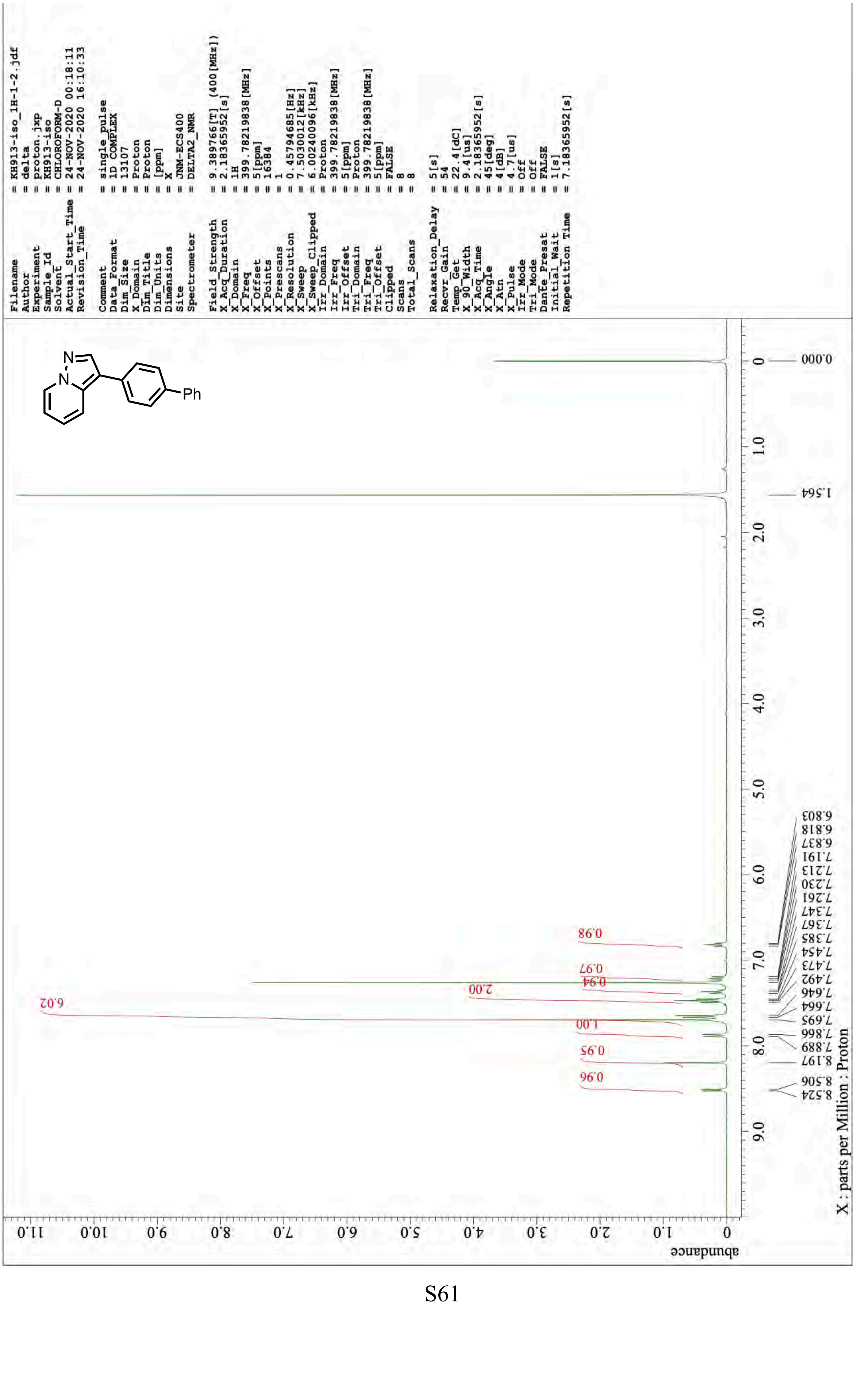

^{13}C NMR of **1D** (101 MHz, $CDCl_3$)

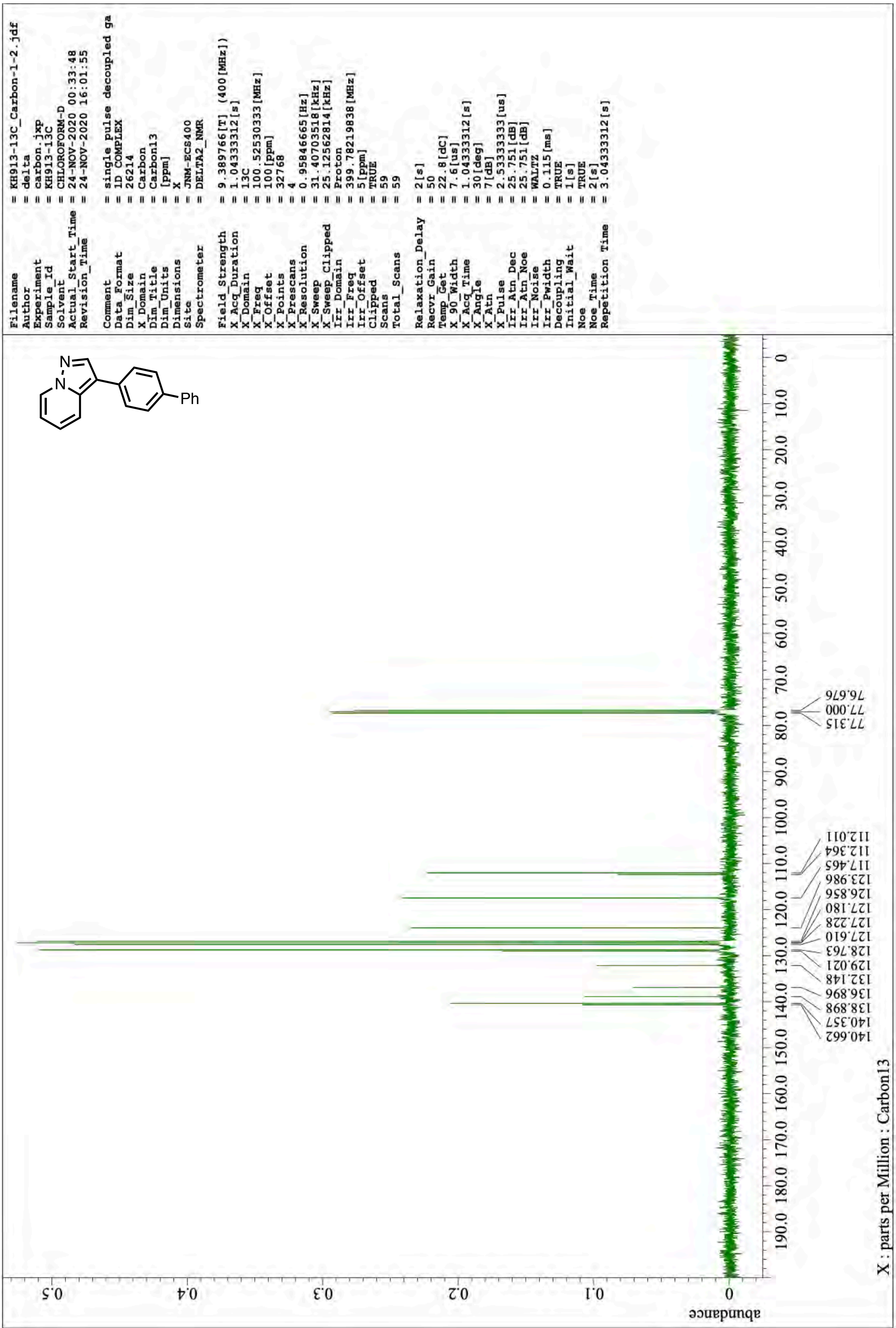

^{1}H NMR of **1G** (400 MHz, $CDCl_3$)

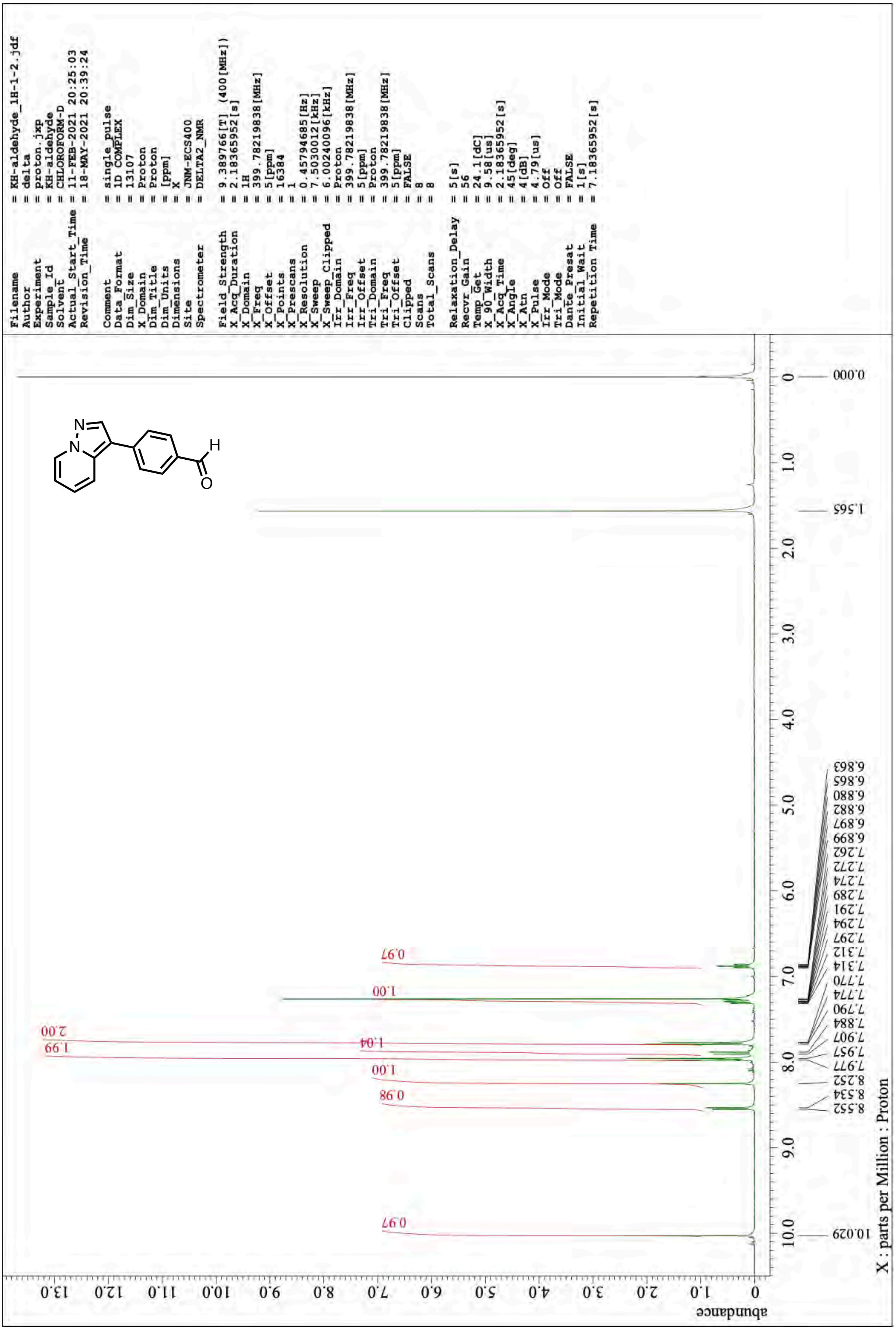

^{13}C NMR of **1G** (101 MHz, $CDCl_3$)

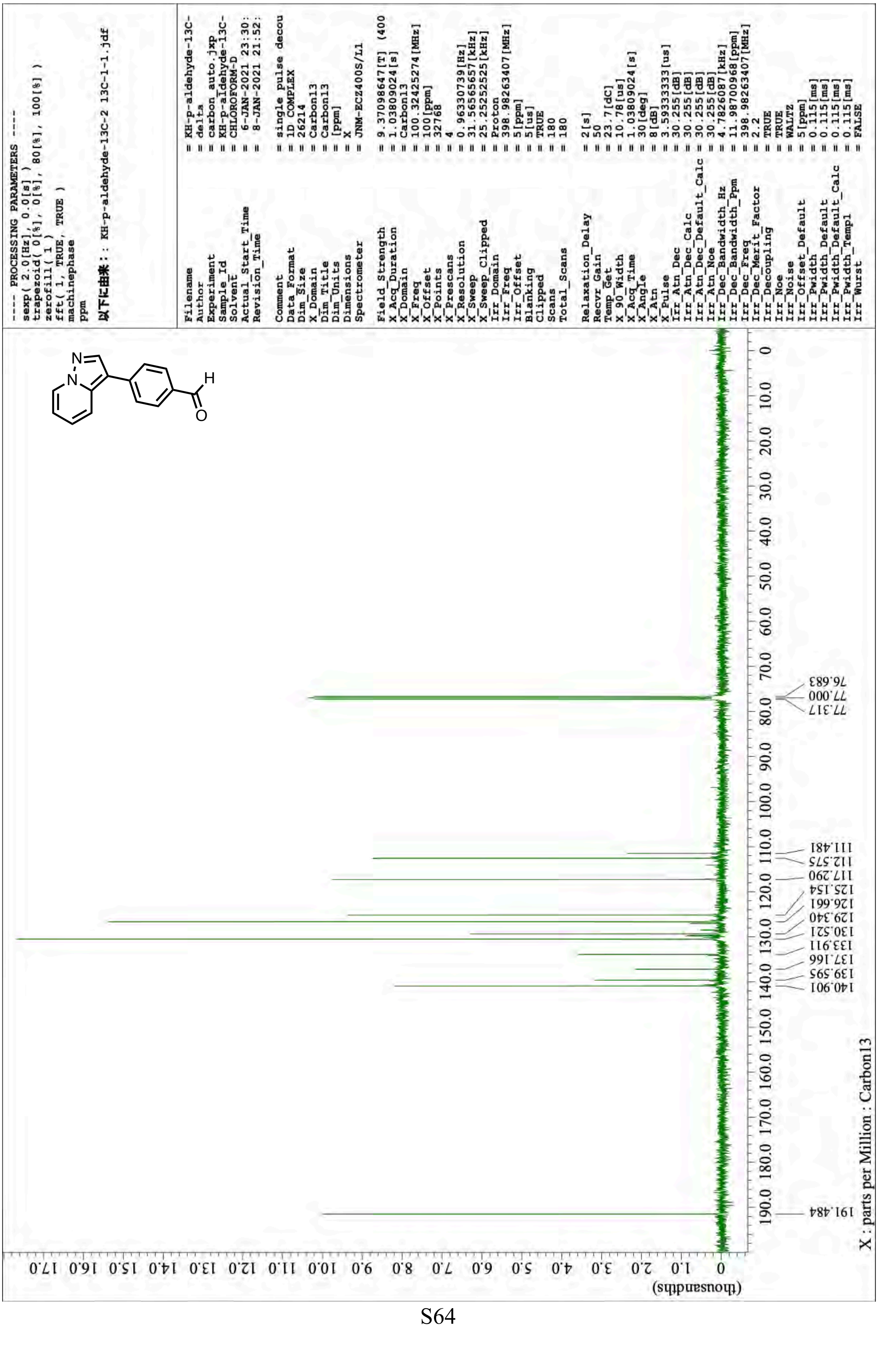

^{1}H NMR of **1H** (400 MHz, $CDCl_3$)

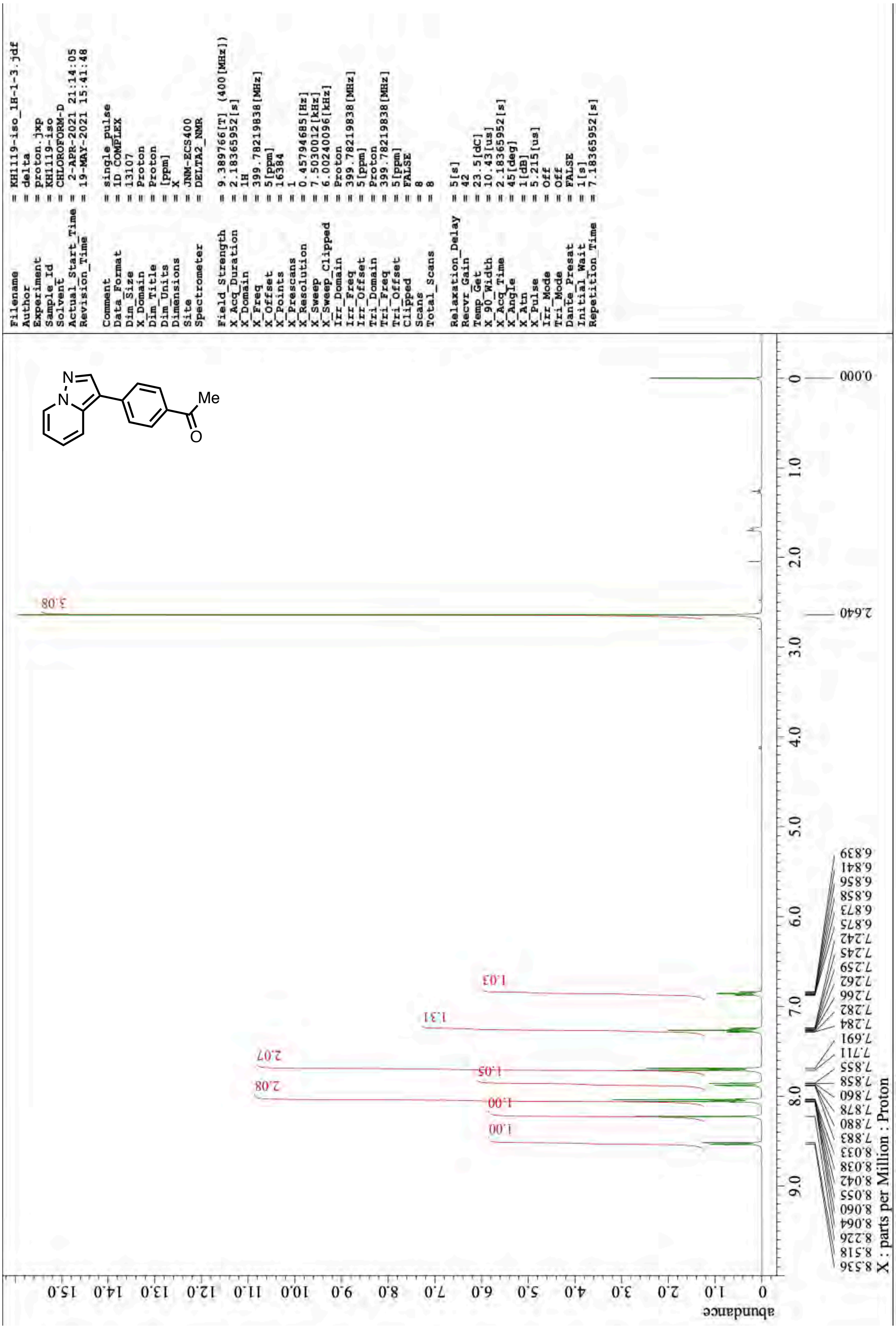

^{13}C NMR of **1H** (101 MHz, $CDCl_3$)

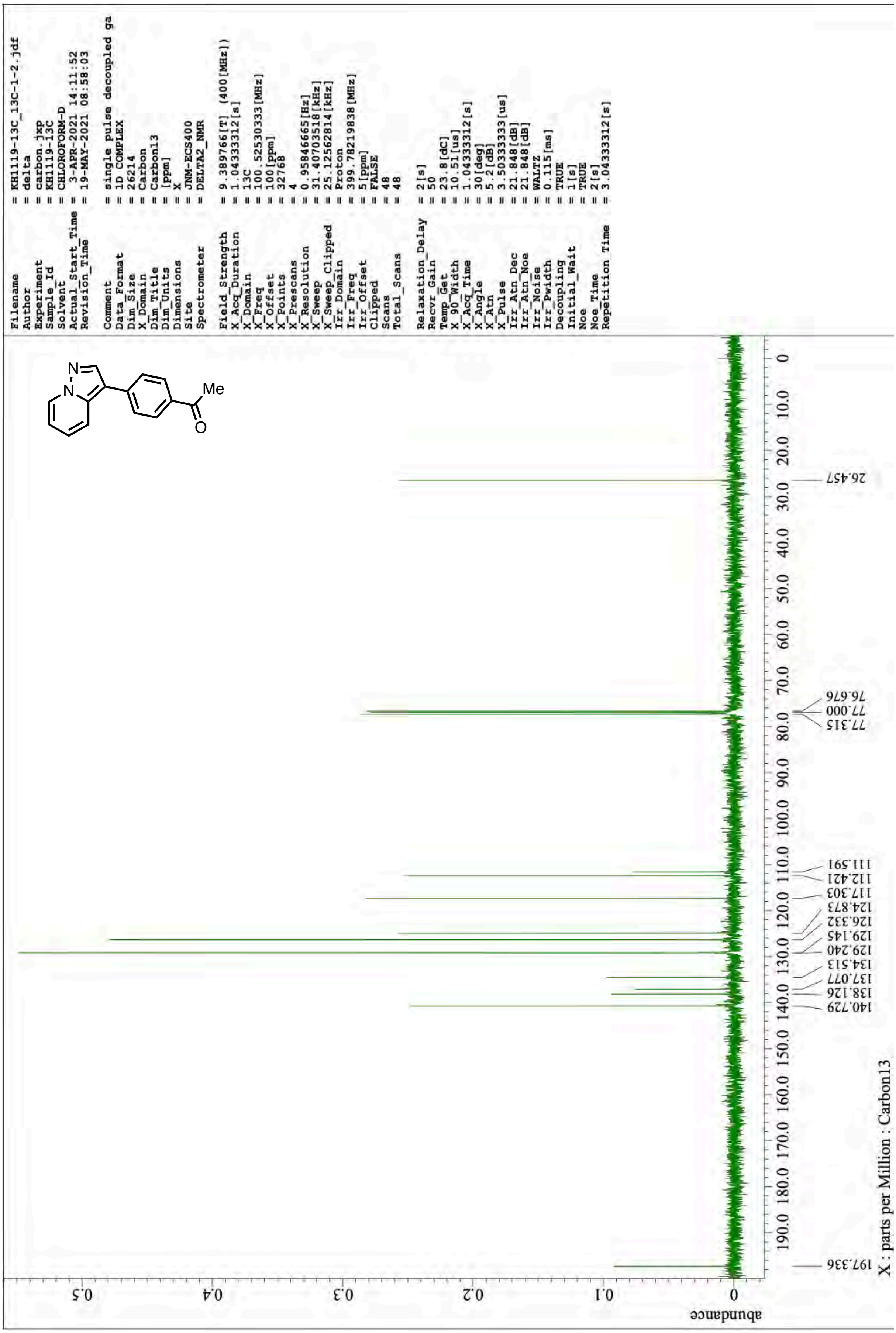

^{1}H NMR of **1K** (400 MHz, $CDCl_3$)

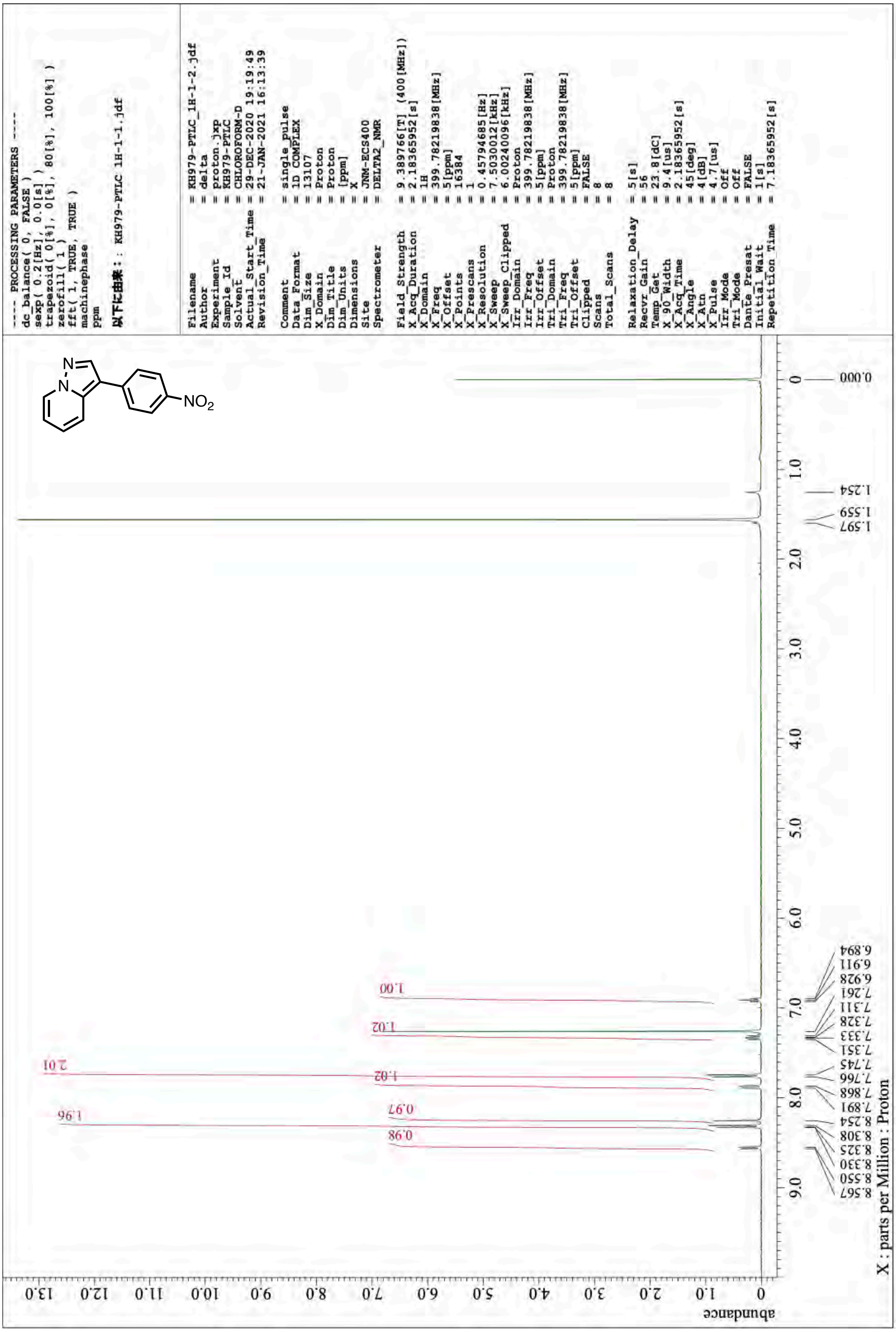

^{13}C NMR of **1K** (101 MHz, $CDCl_3$)

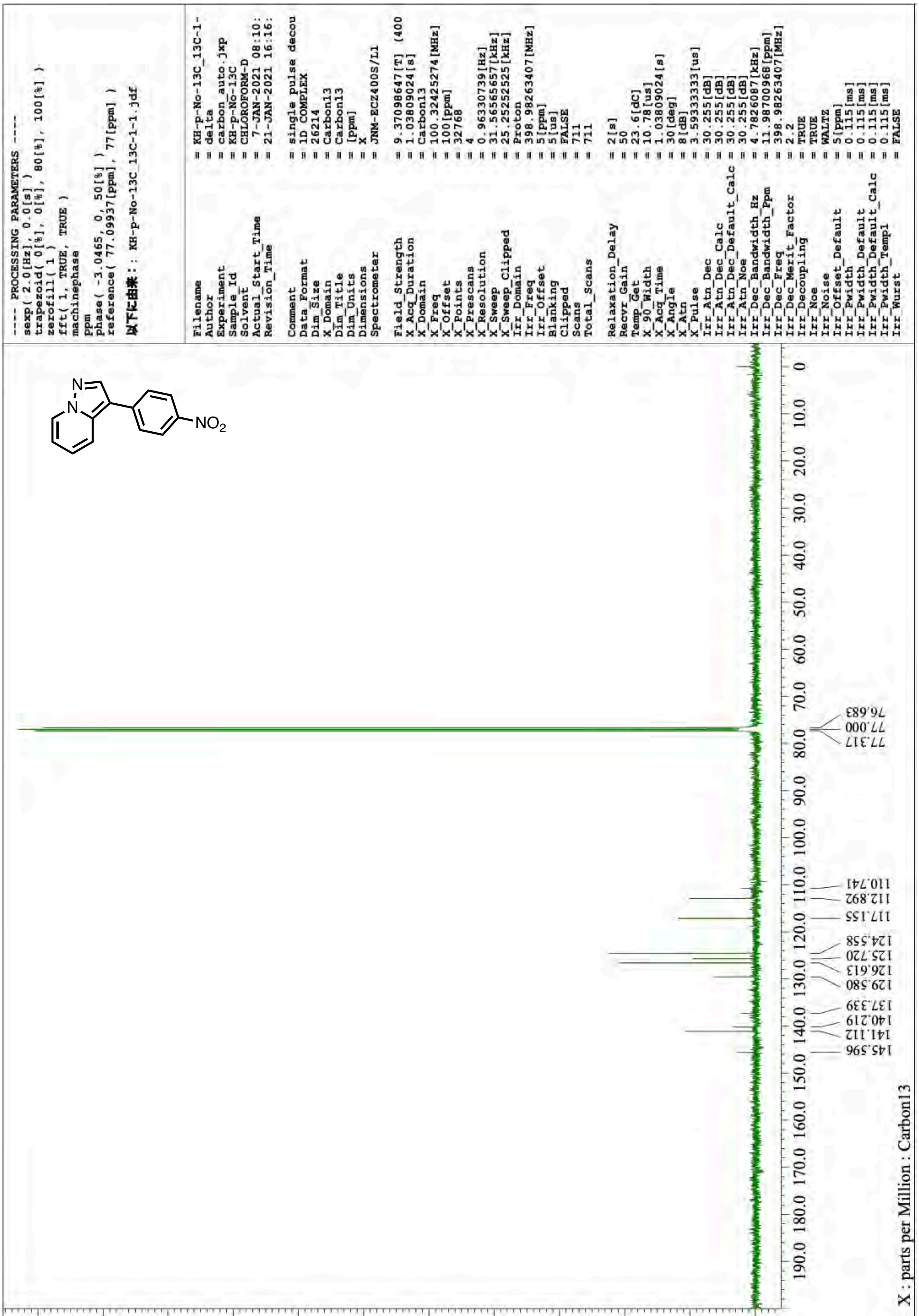

^{1}H NMR of **1M** (400 MHz, $CDCl_3$)

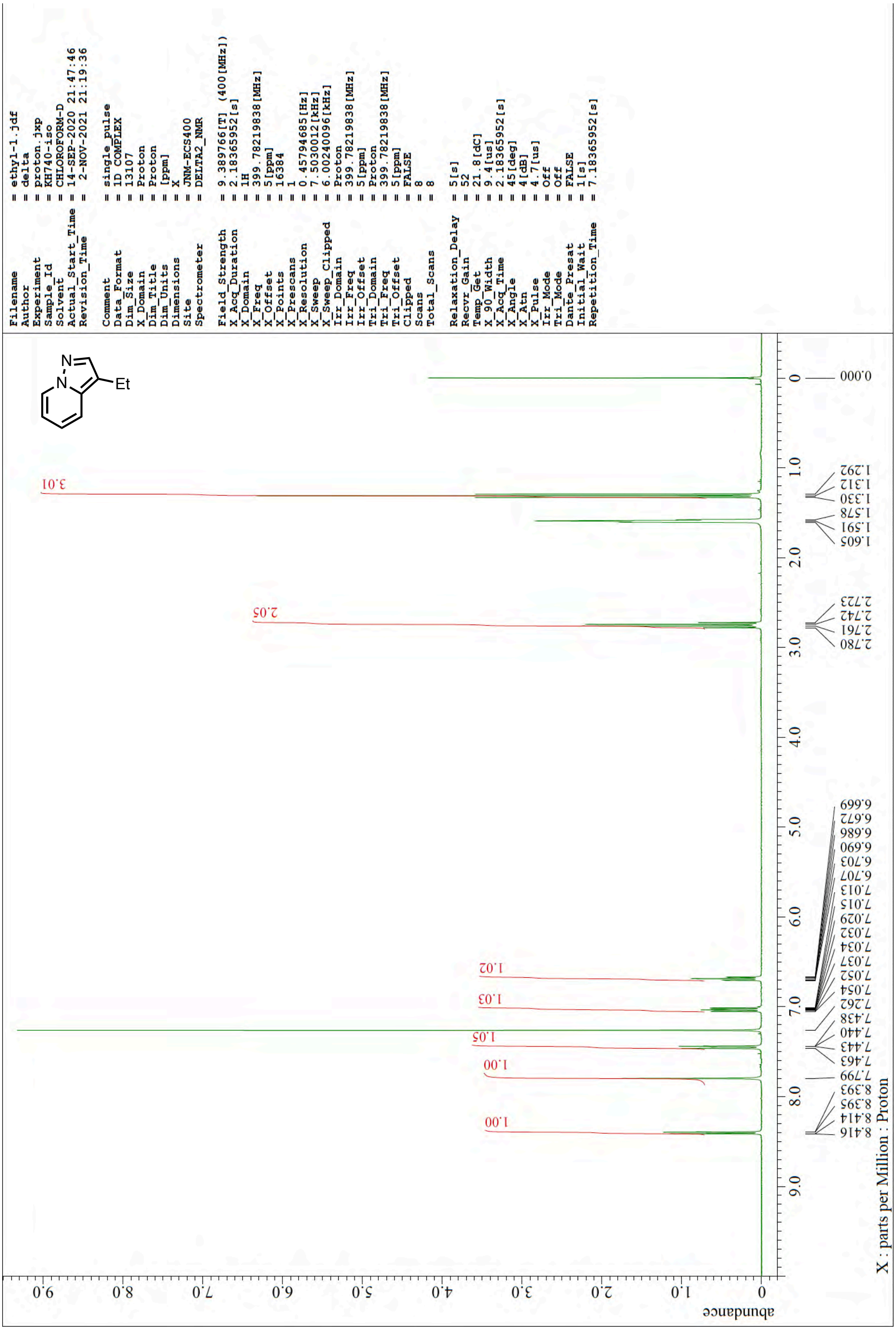

^{13}C NMR of **1M** (101 MHz, $CDCl_3$)

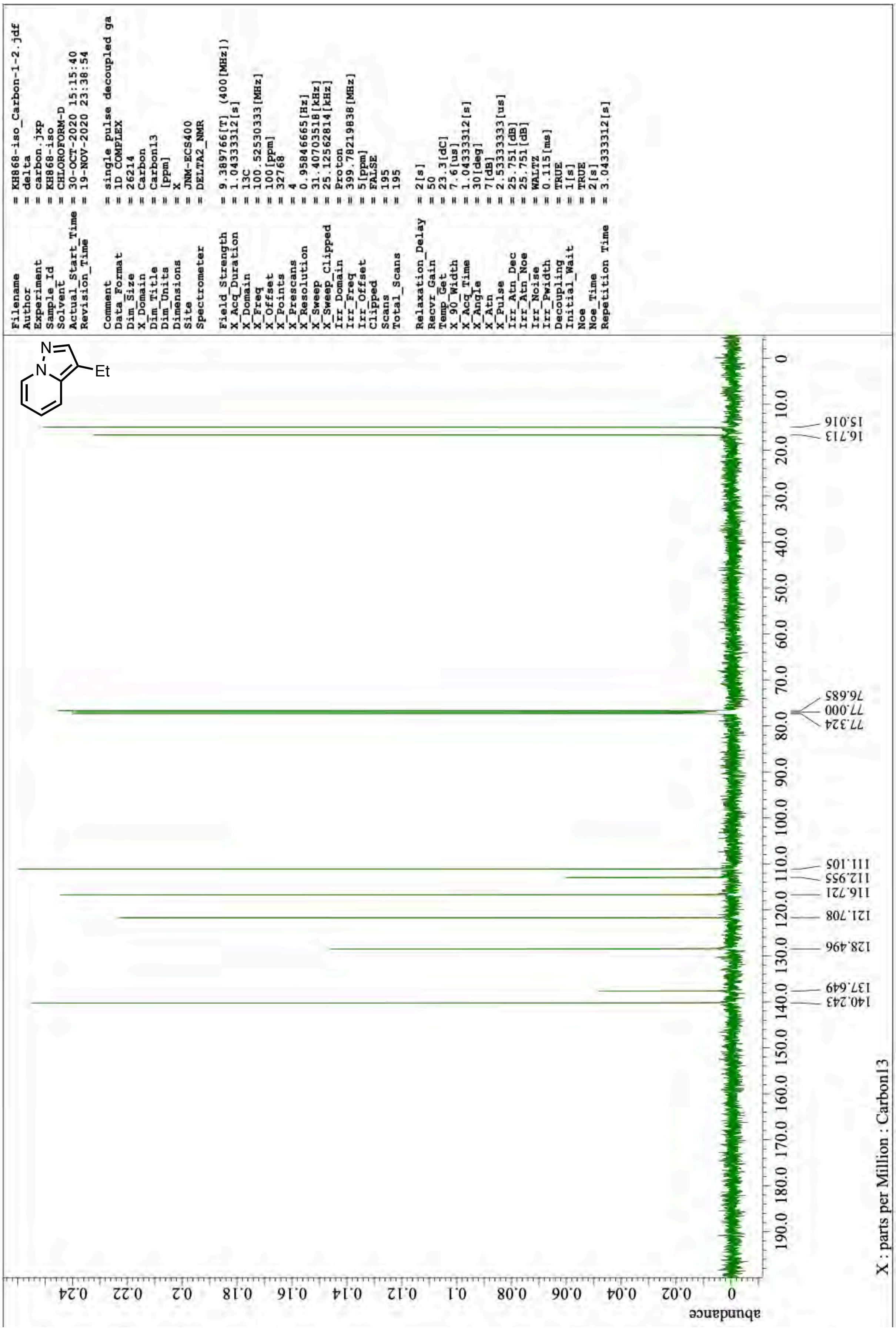

^{1}H NMR of **1N** (400 MHz, $CDCl_3$)

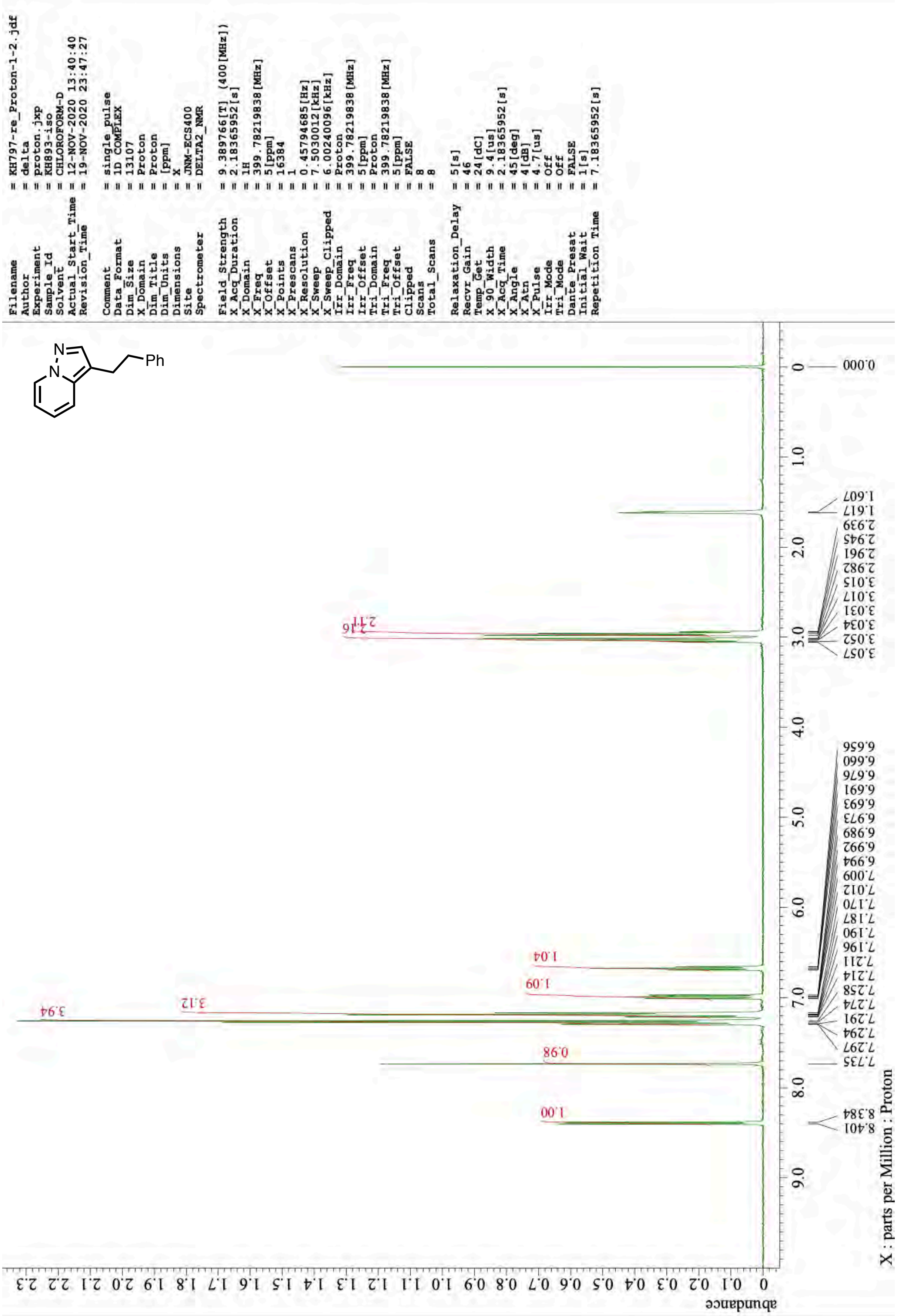

^{13}C NMR of **1N** (101 MHz, $CDCl_3$)

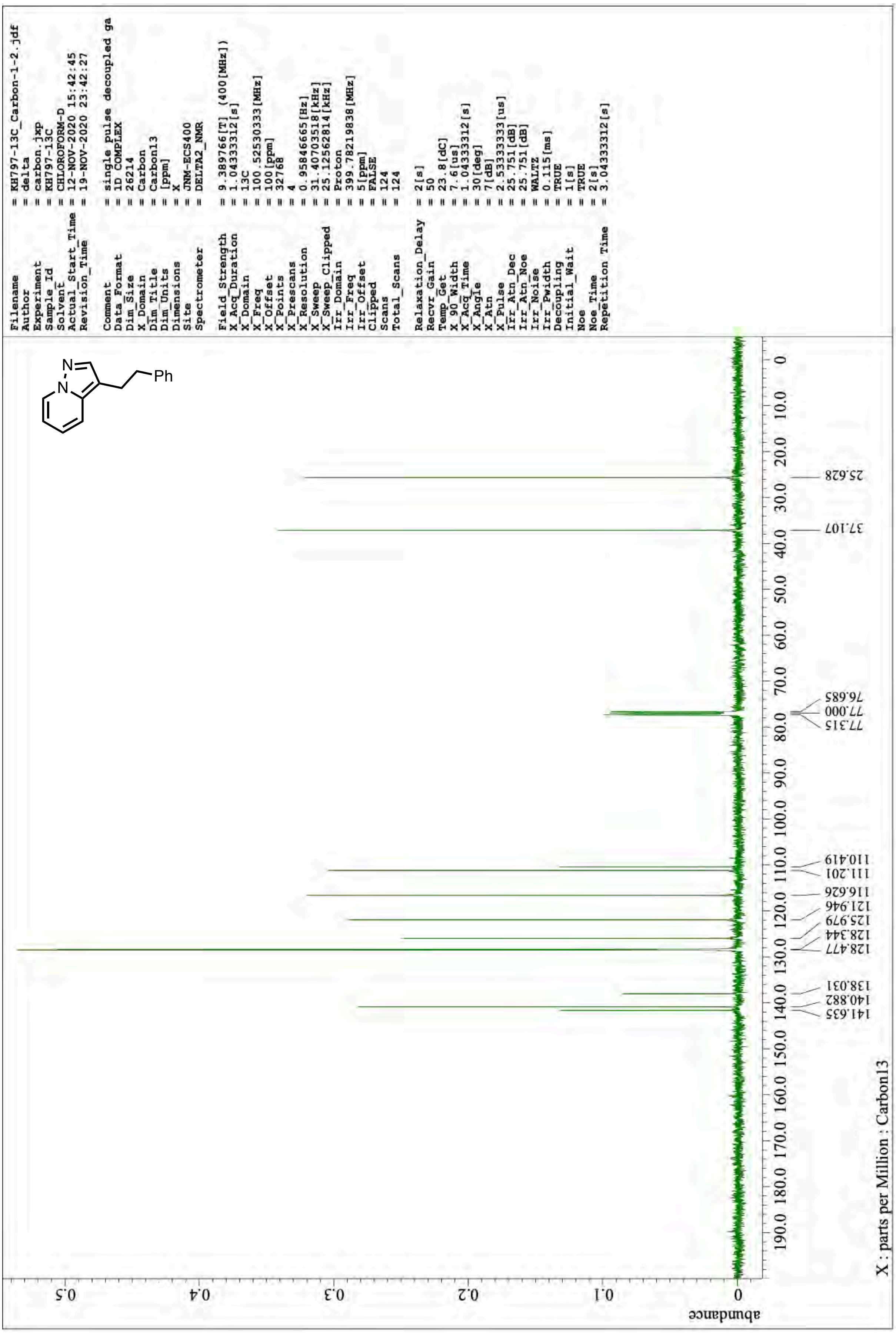

^{1}H NMR of **1O** (400 MHz, $CDCl_3$)

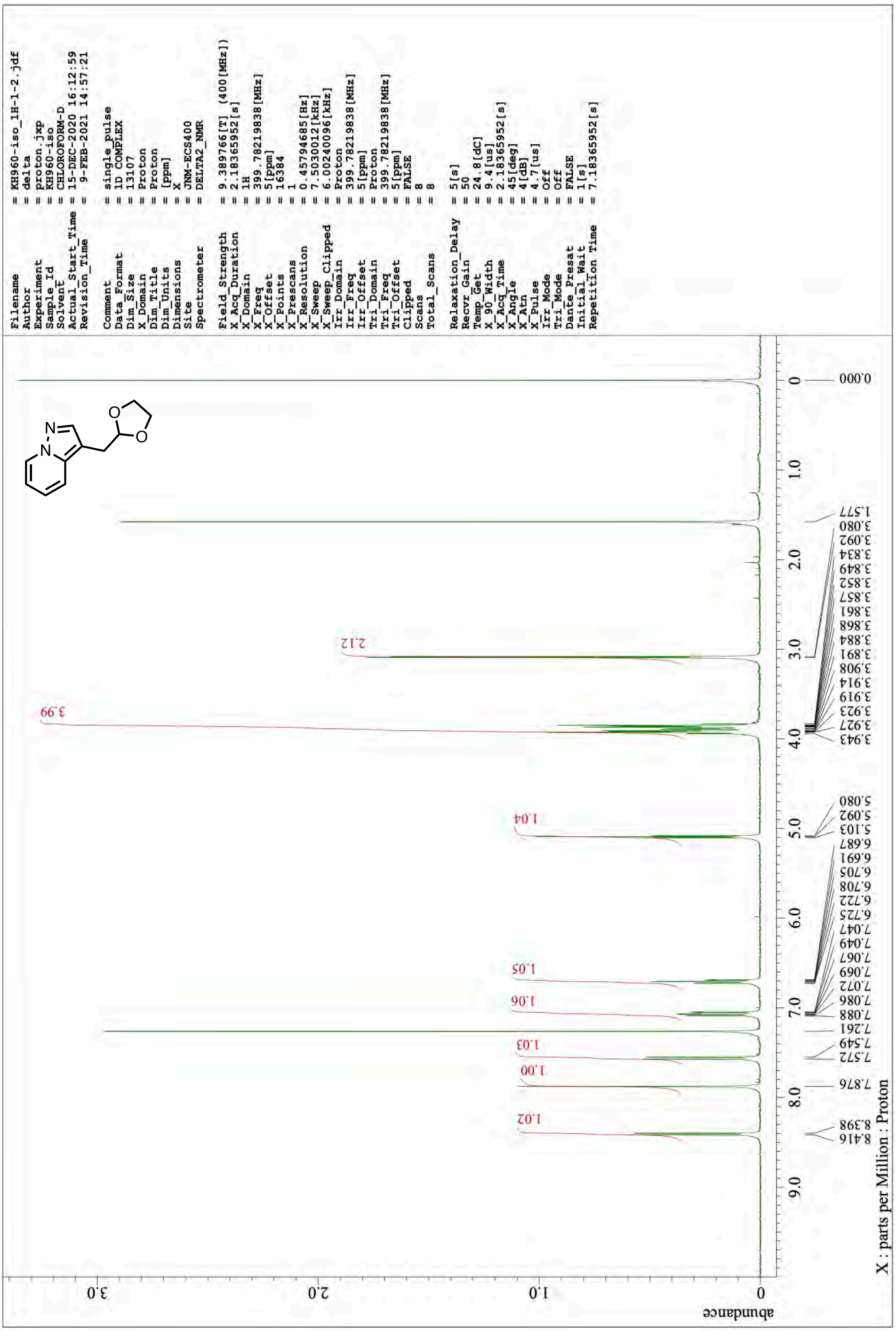

^{13}C NMR of **1O** (101 MHz, $CDCl_3$)

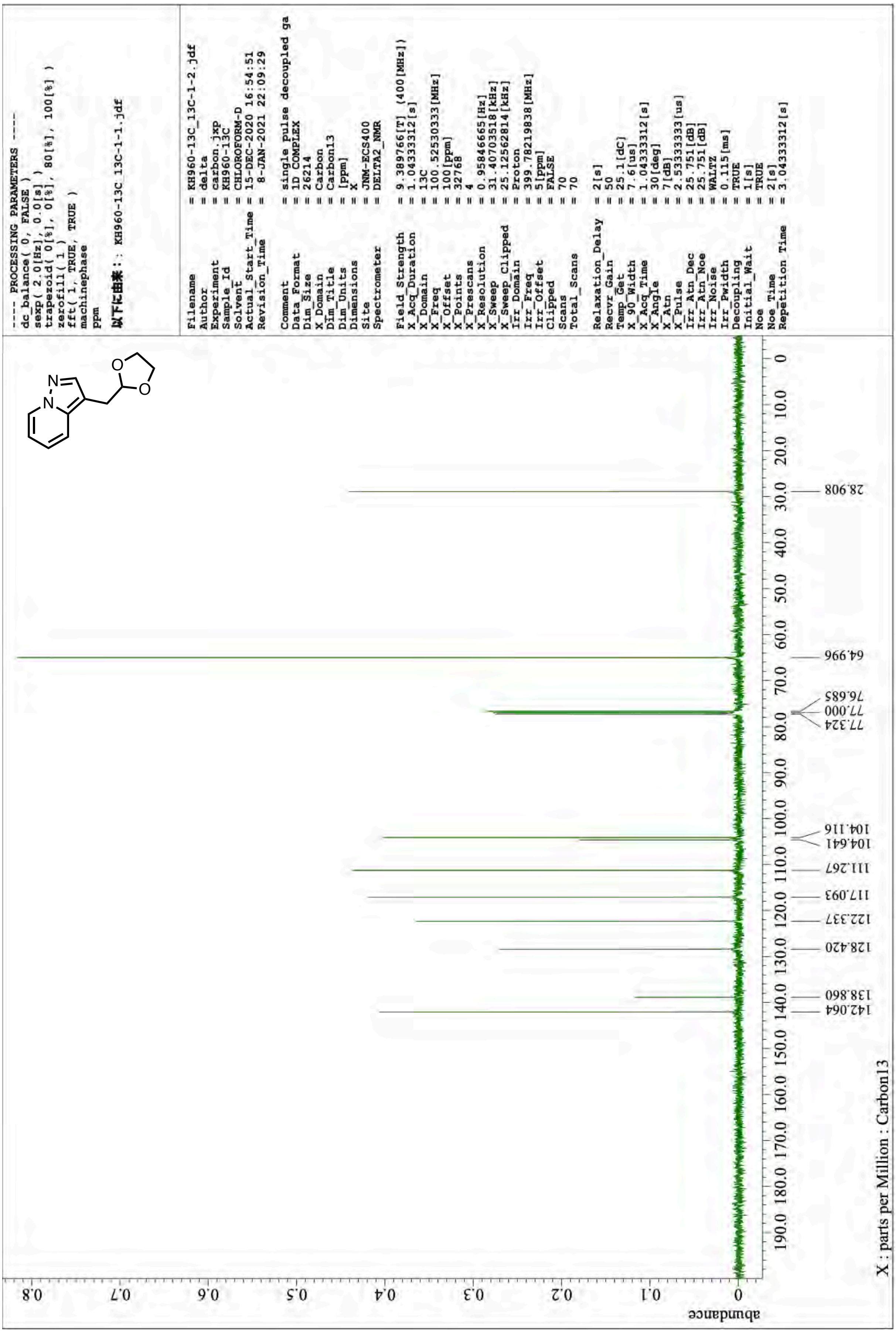

^{1}H NMR of **1P** (400 MHz, $CDCl_3$)

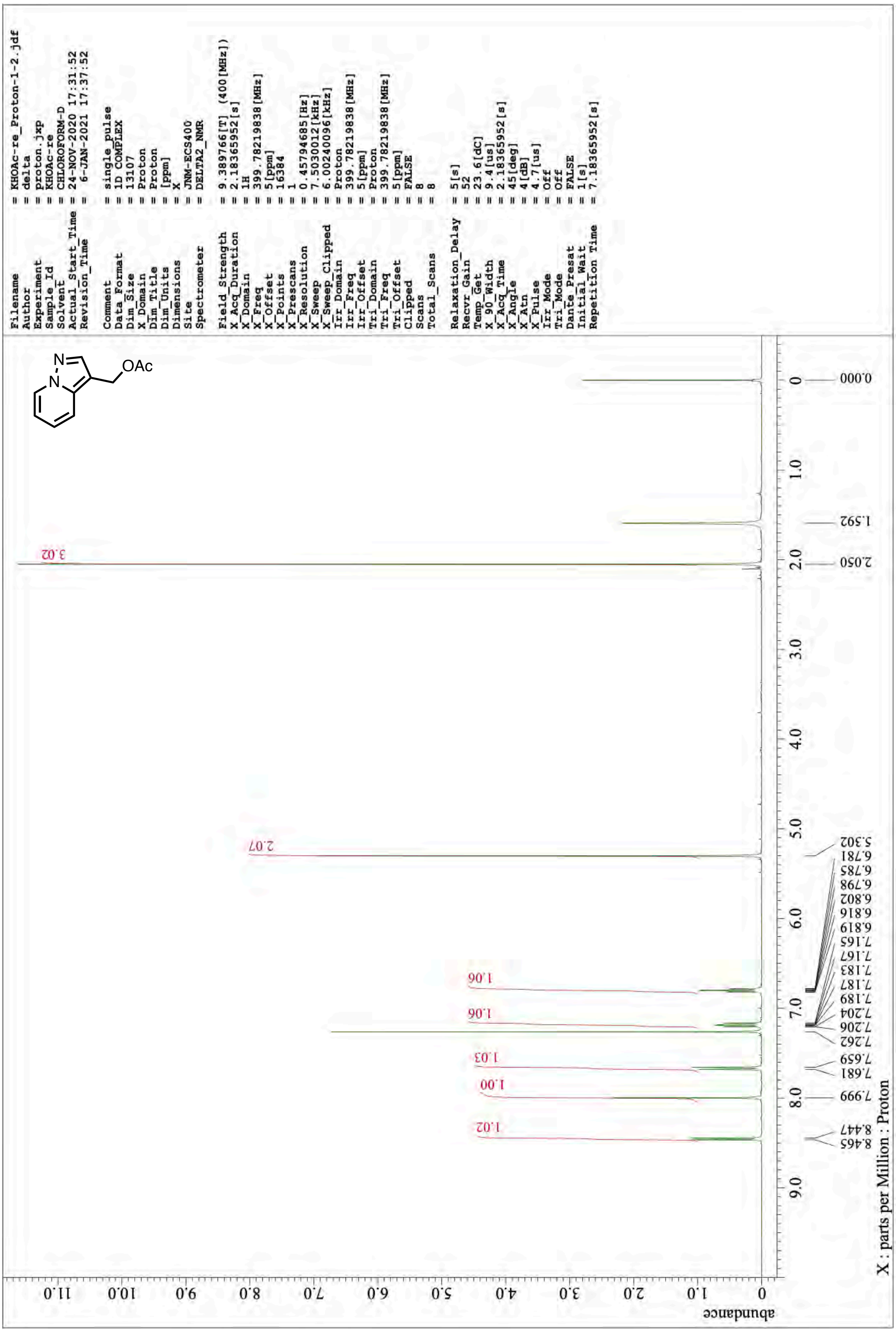

^{13}C NMR of **1P** (101 MHz, $CDCl_3$)

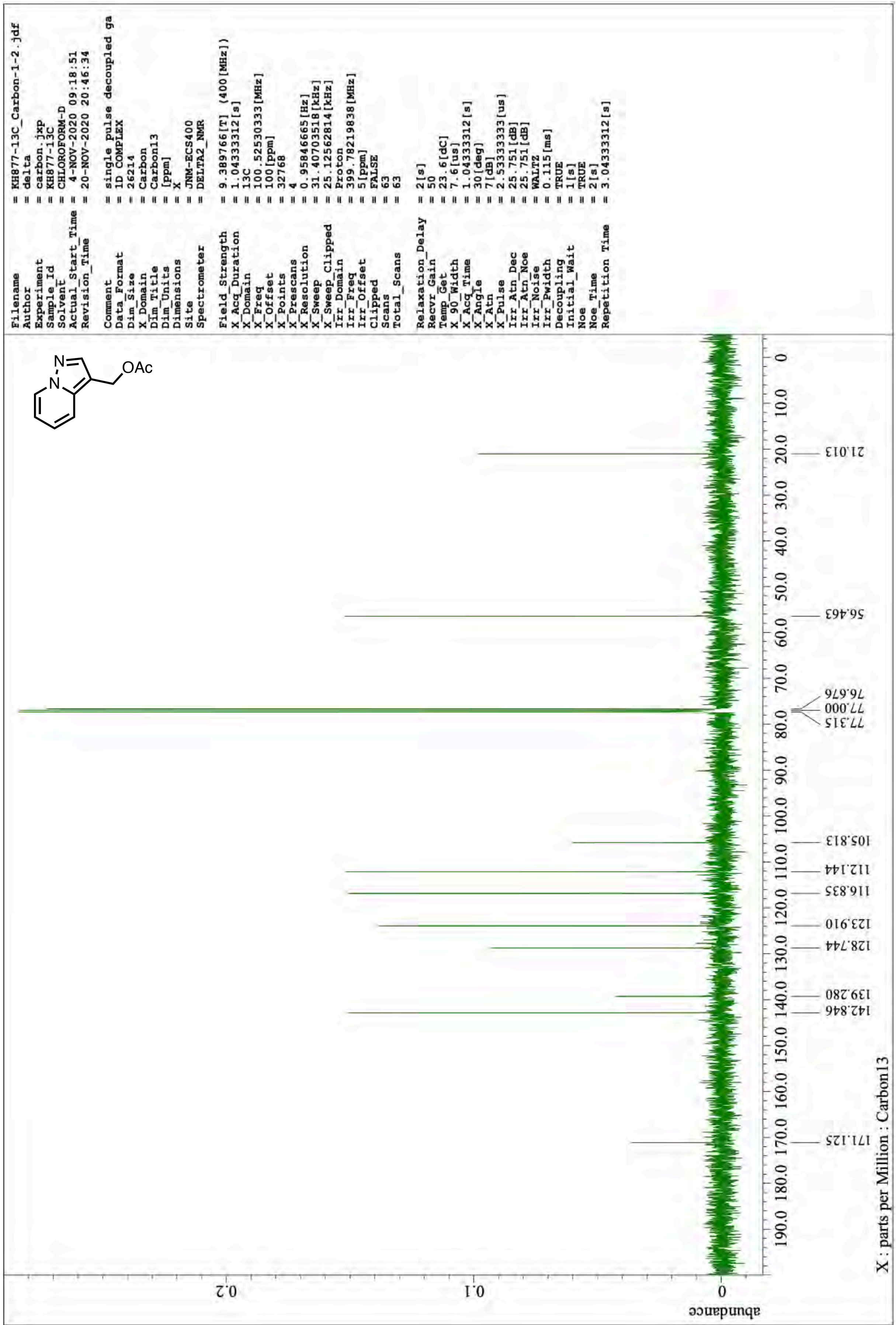

^{1}H NMR of **1R** (400 MHz, $CDCl_3$)

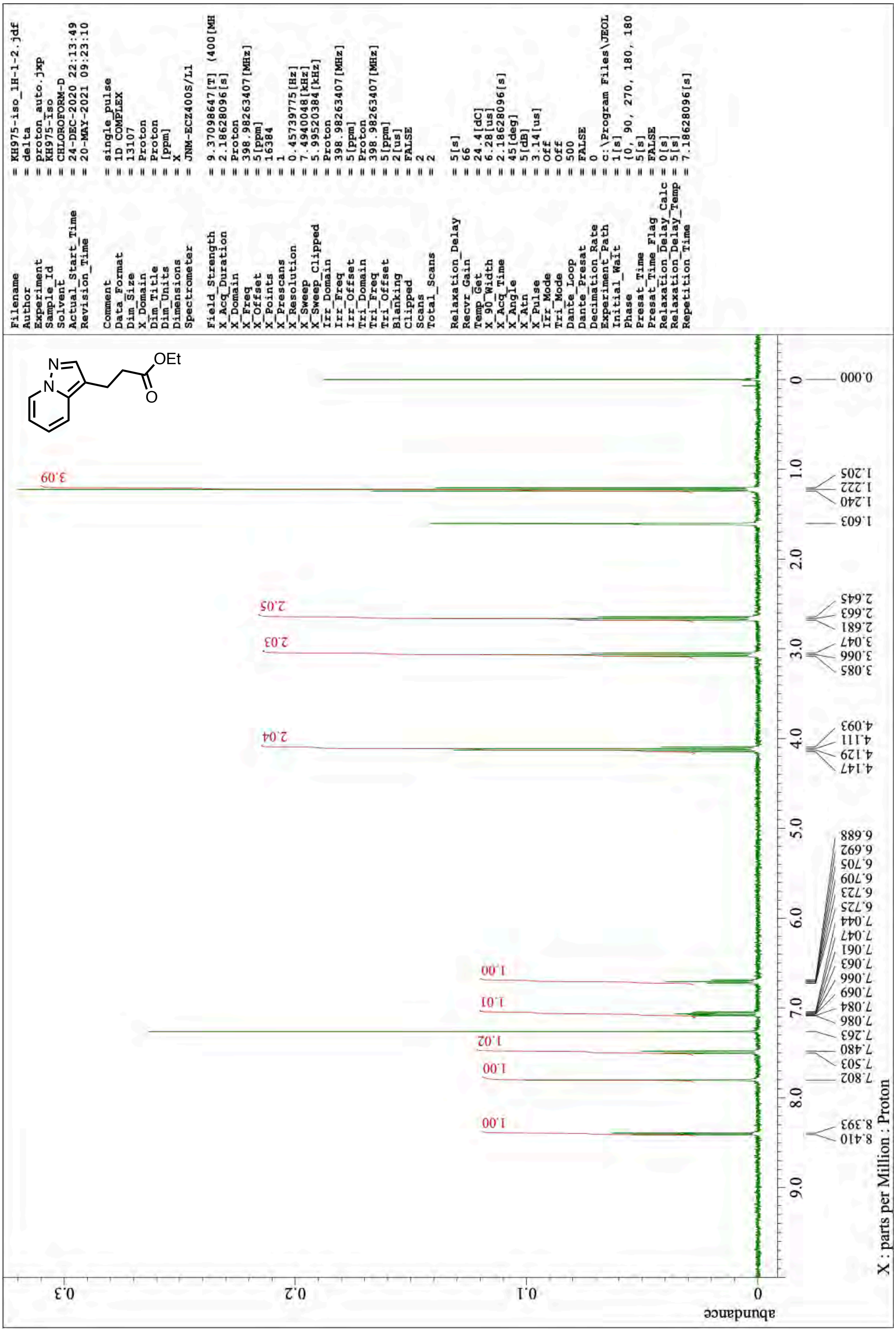

^{13}C NMR of **1R** (101 MHz, $CDCl_3$)

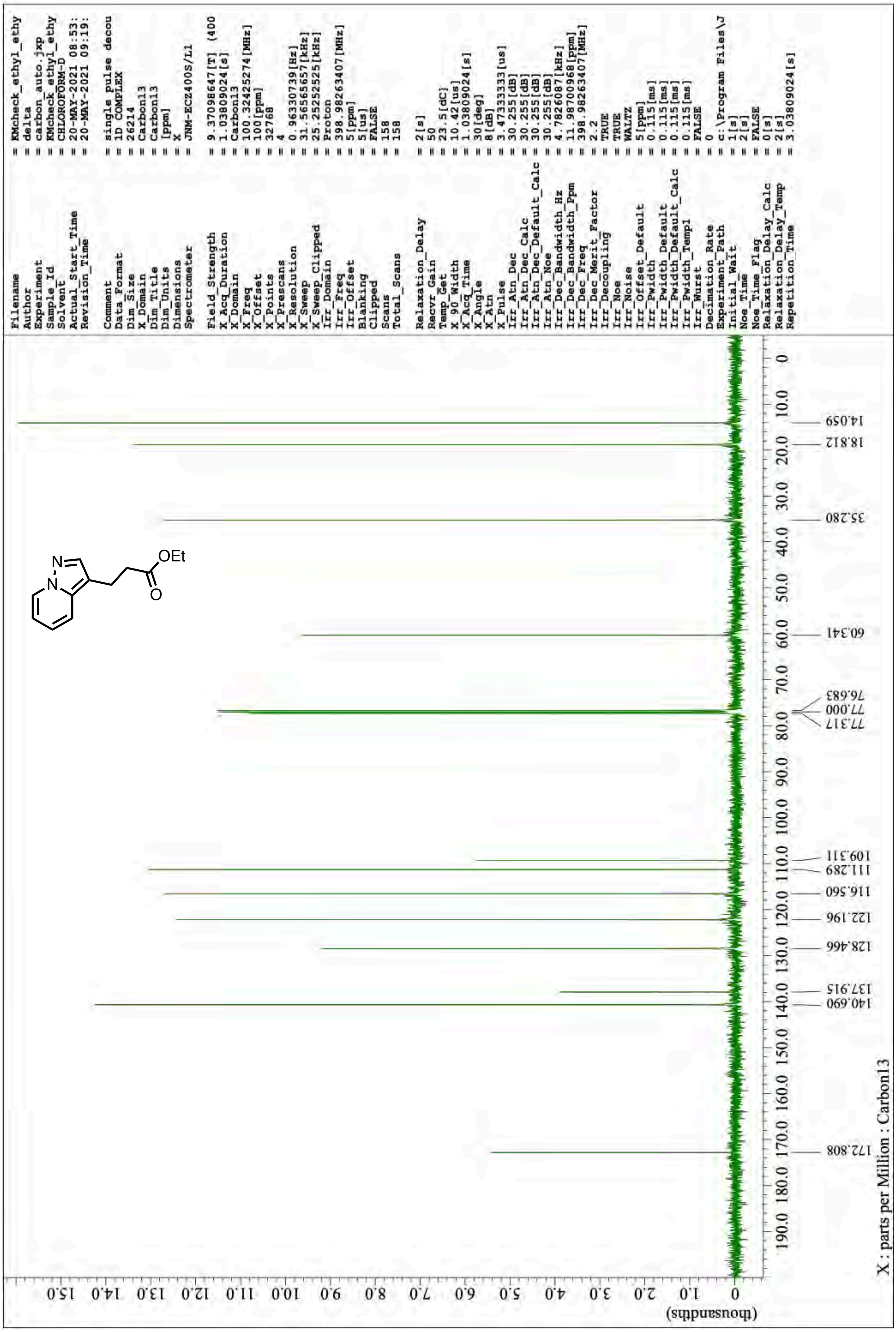

^{1}H NMR of **1T** (400 MHz, $CDCl_3$)

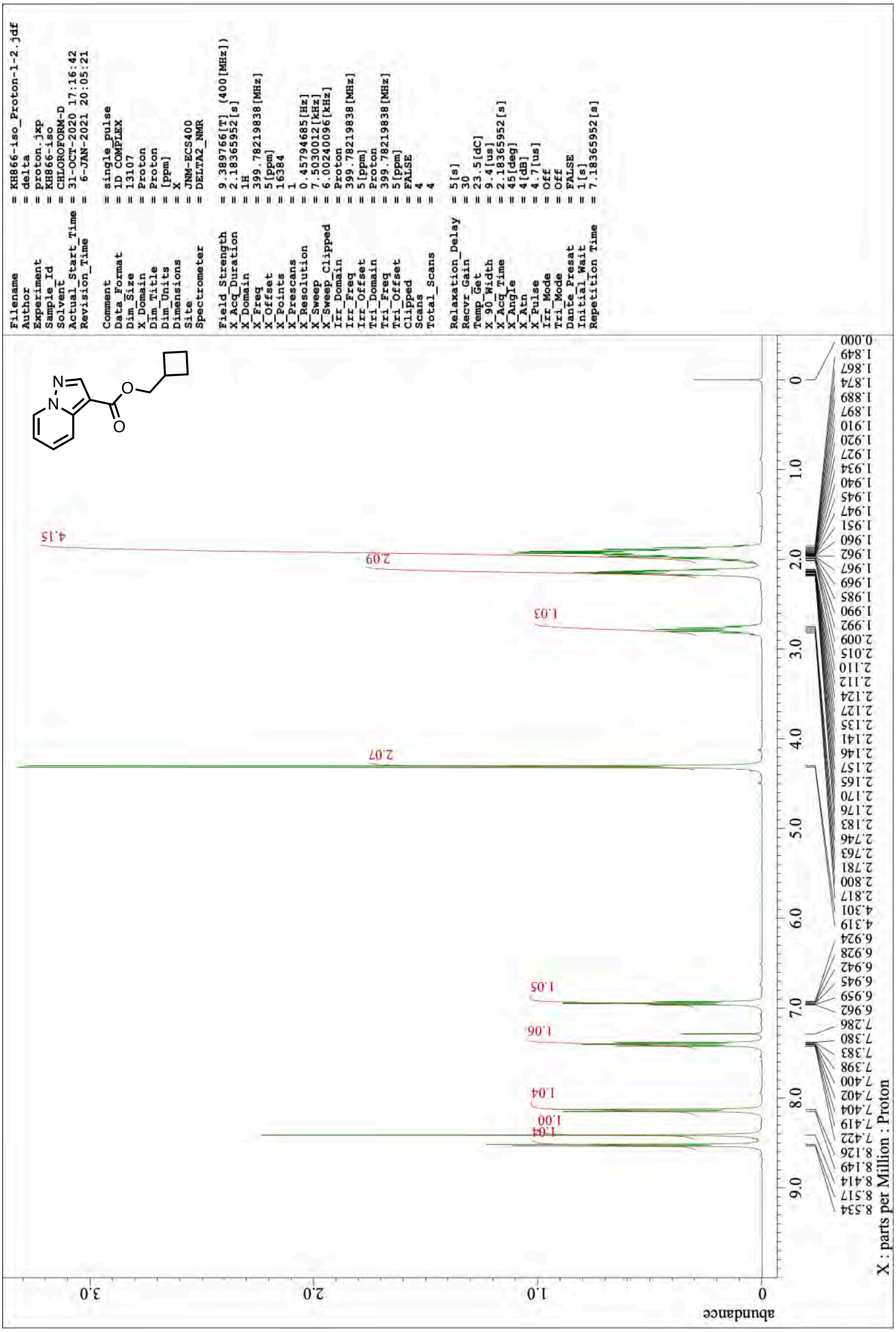

^{13}C NMR of **1T** (101 MHz, $CDCl_3$)

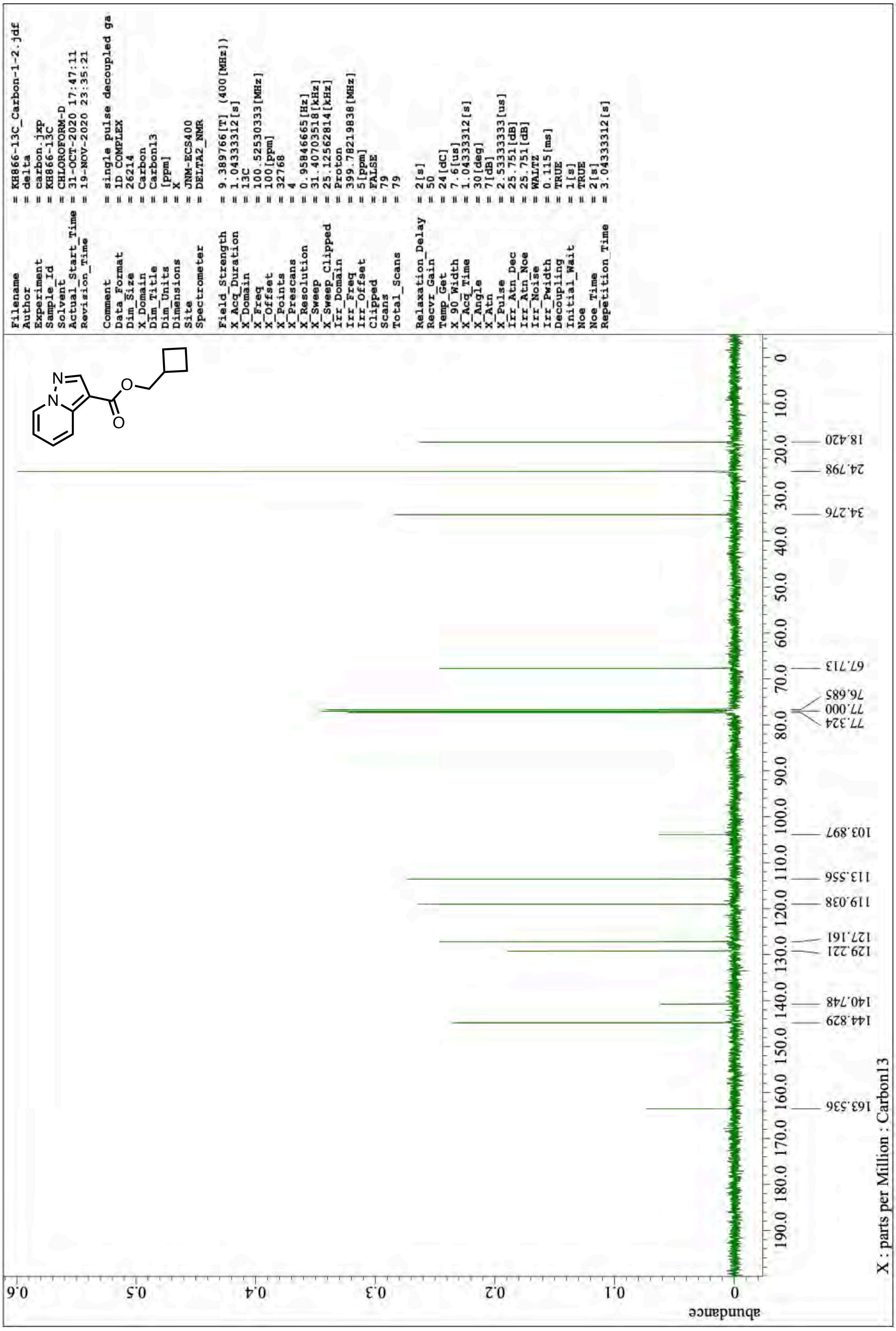

^{1}H NMR of **1U** (400 MHz, $CDCl_3$)

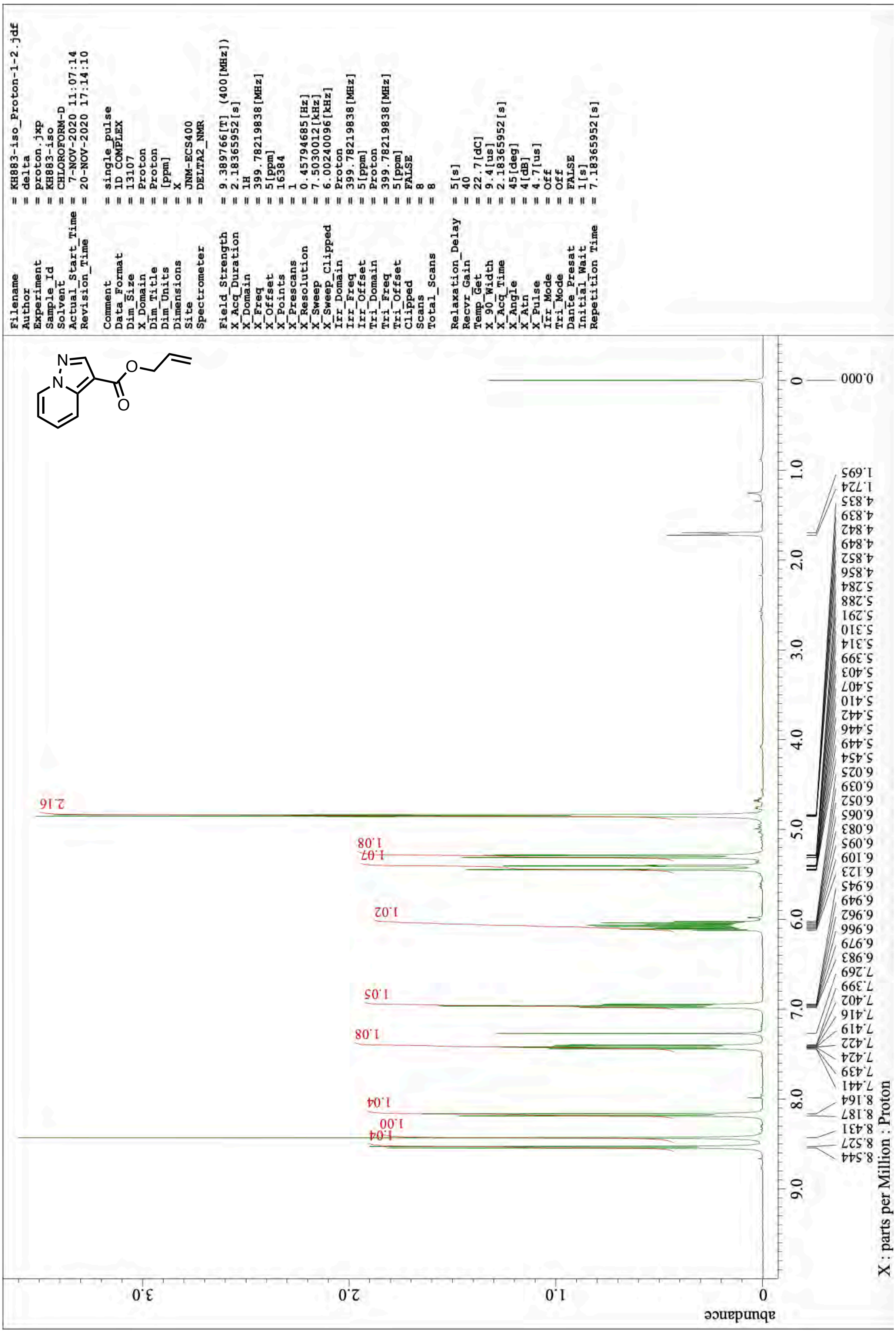

^{13}C NMR of **1U** (101 MHz, $CDCl_3$)

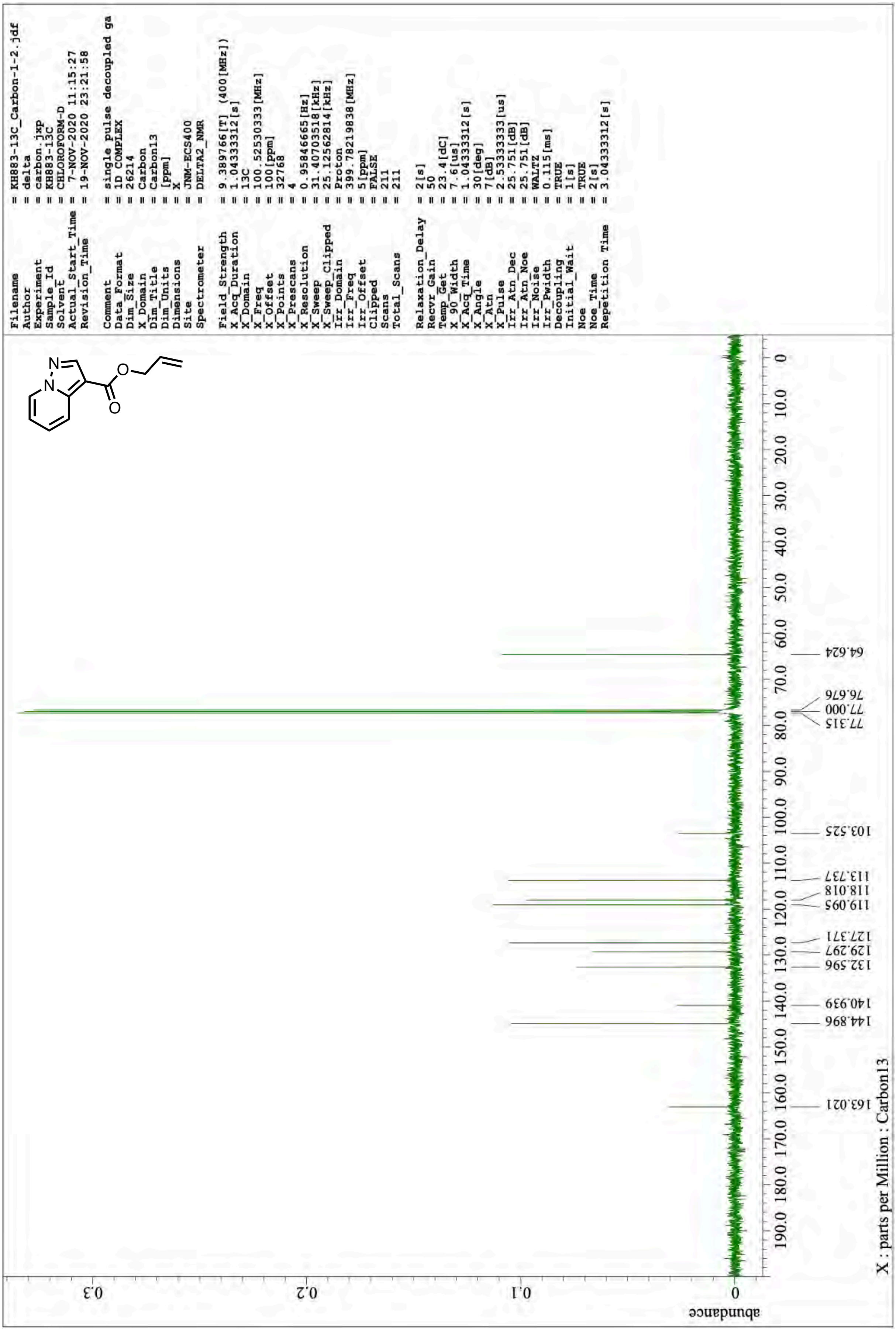

^{1}H NMR of **1V** (400 MHz, $CDCl_3$)

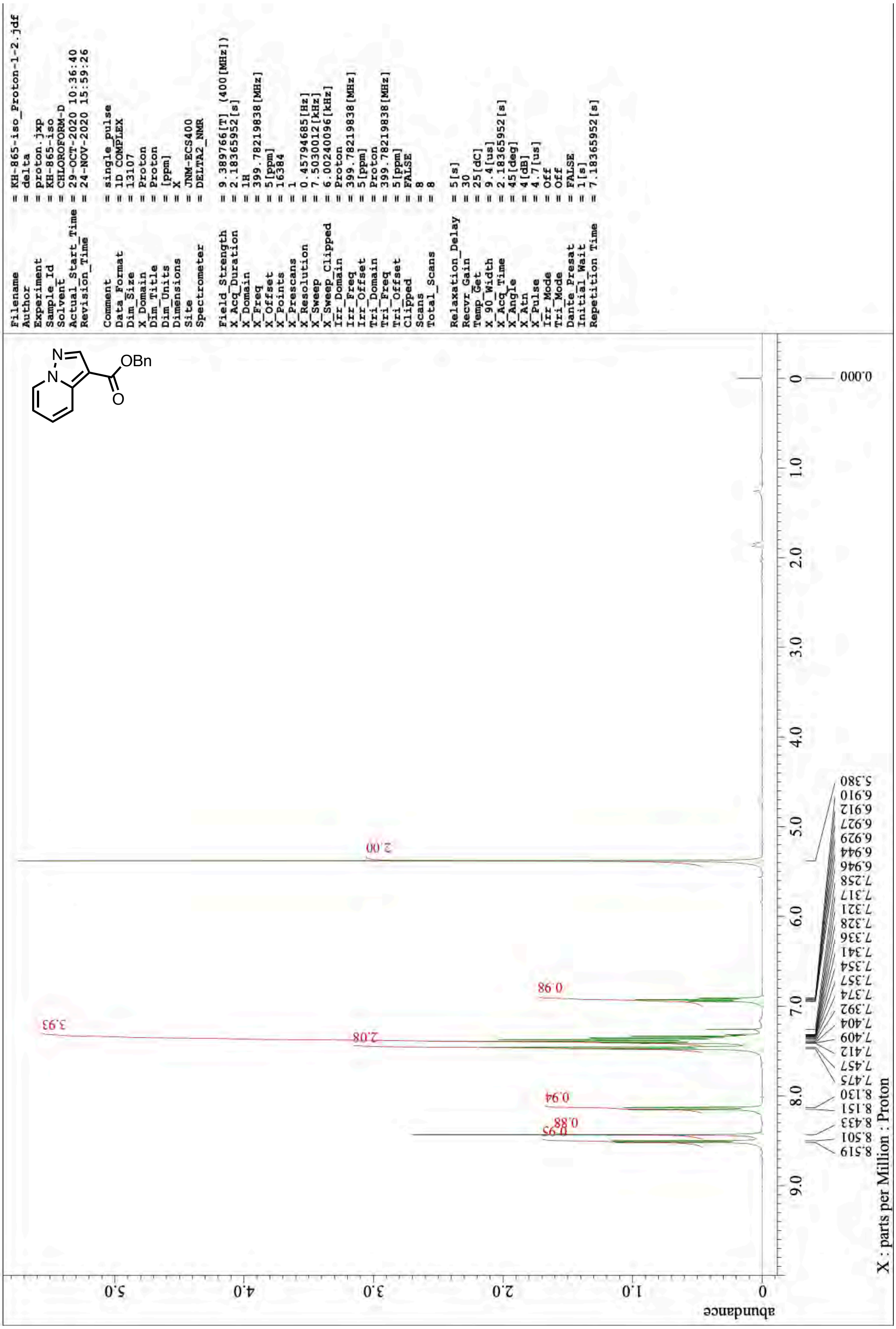

^{13}C NMR of **1V** (101 MHz, $CDCl_3$)

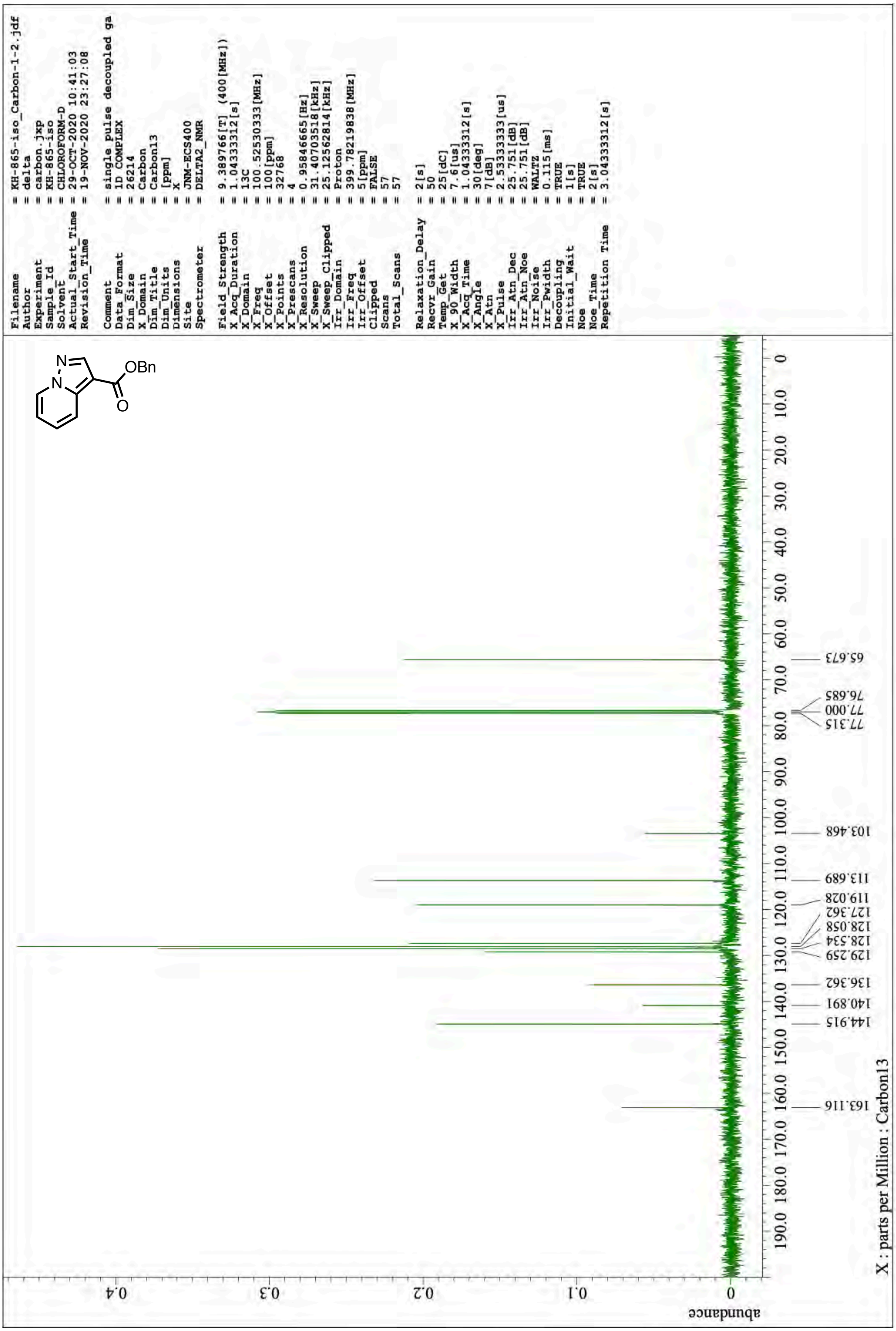

^{1}H NMR of **1W** (400 MHz, $CDCl_3$)

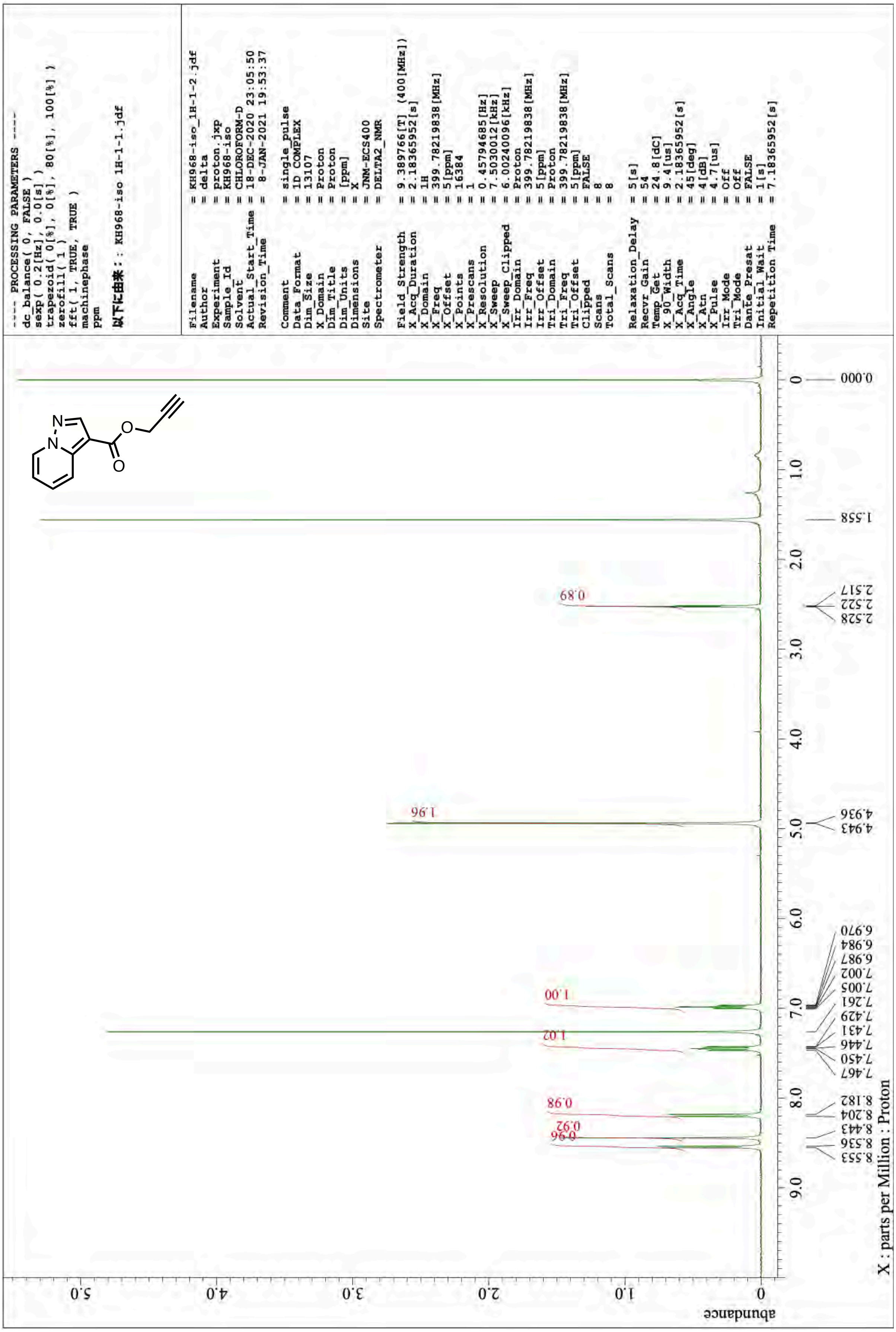

^{13}C NMR of **1W** (101 MHz, $CDCl_3$)

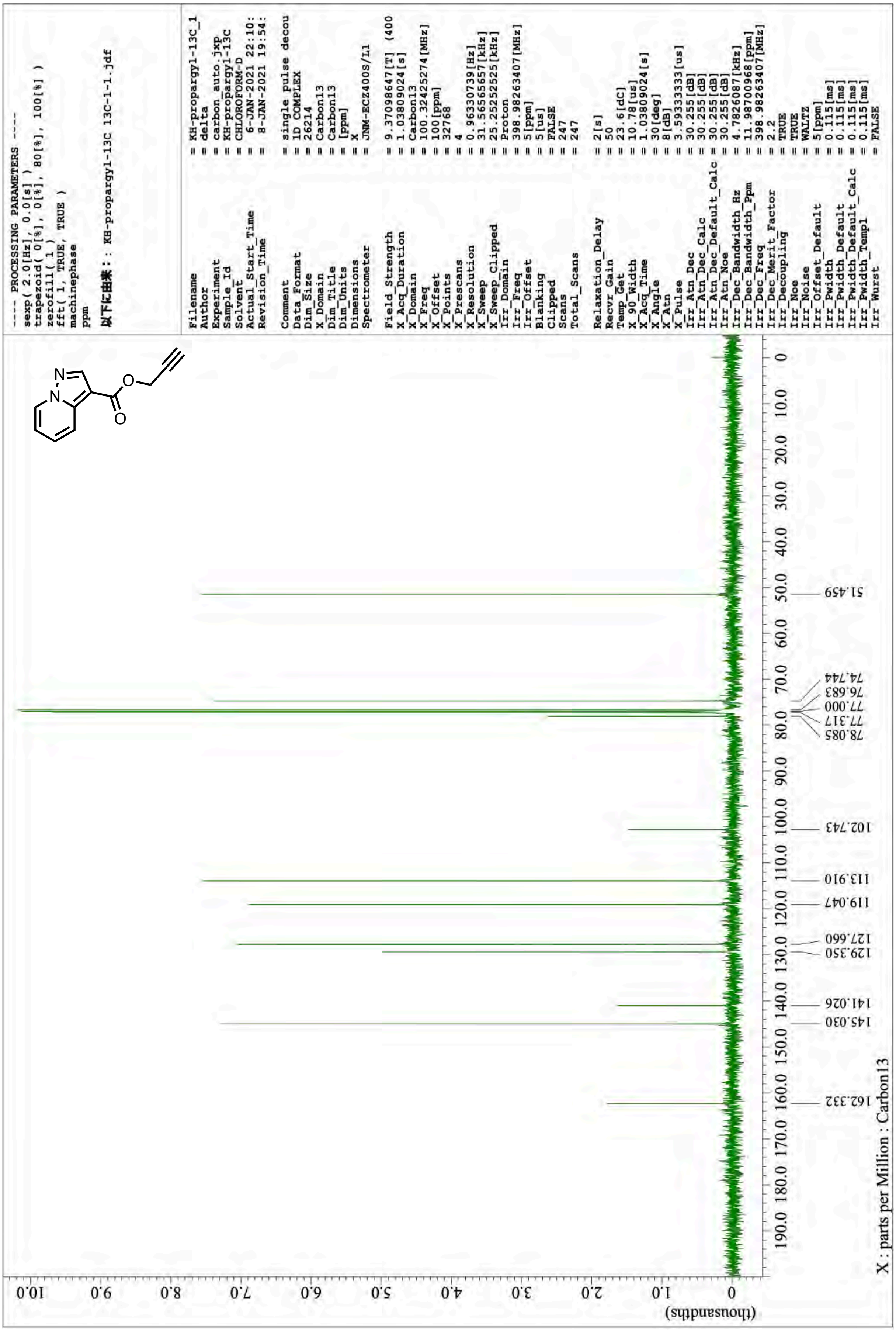

^{1}H NMR of **1X** (400 MHz, CDCl$_3$)

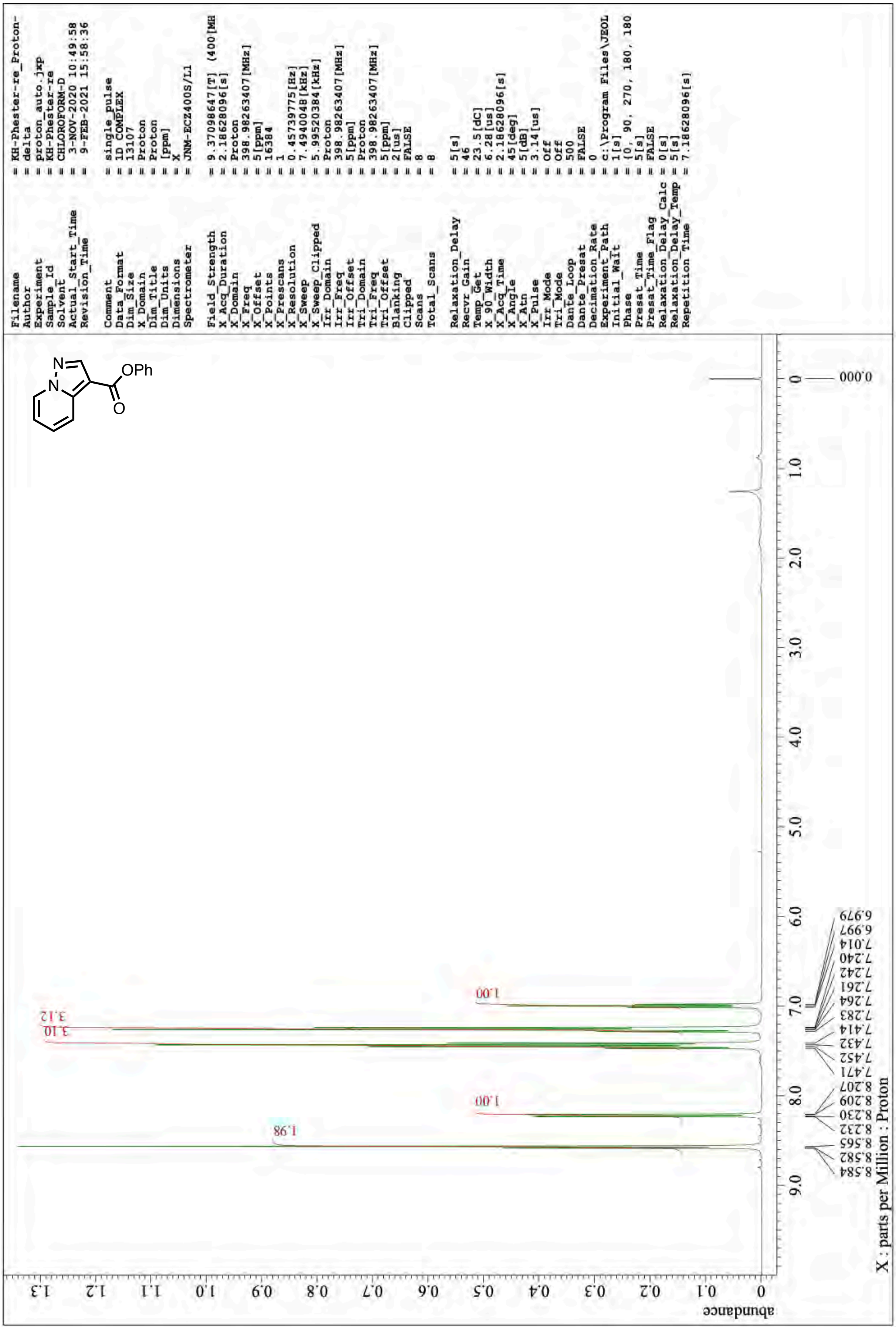

^{13}C NMR of **1X** (101 MHz, $CDCl_3$)

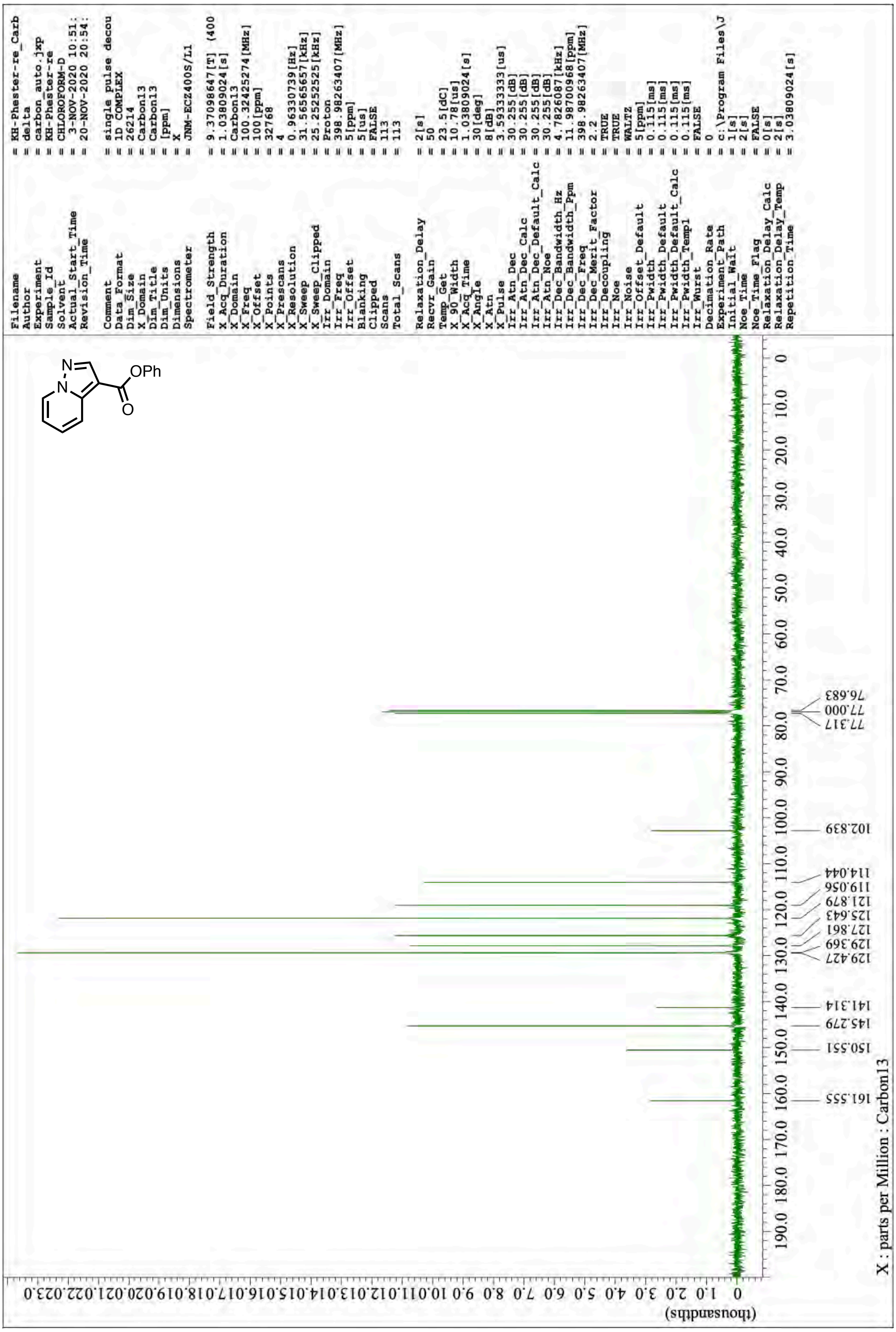

^{1}H NMR of **1Z** (400 MHz, $CDCl_3$)

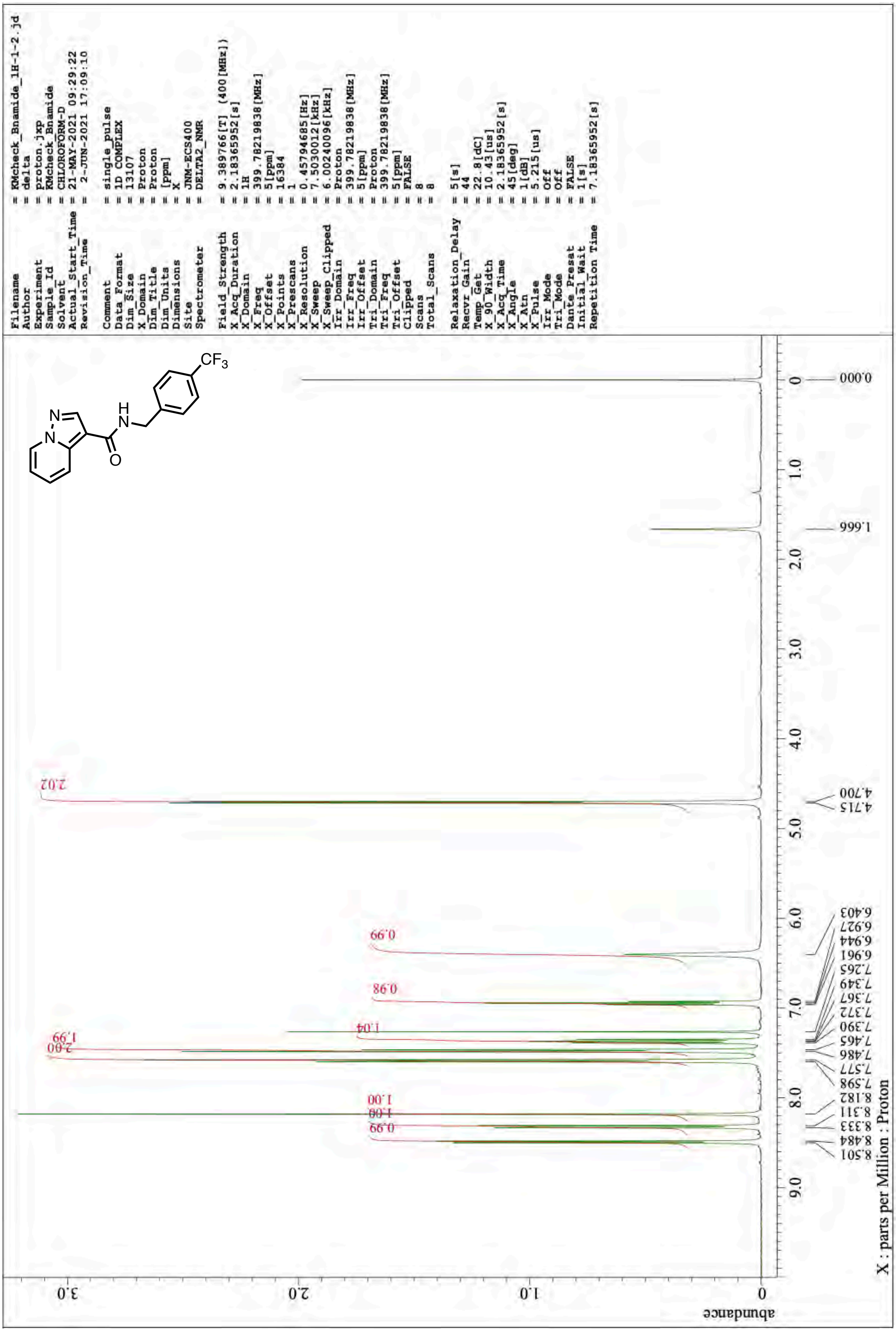

^{13}C NMR of **1Z** (101 MHz, $CDCl_3$)

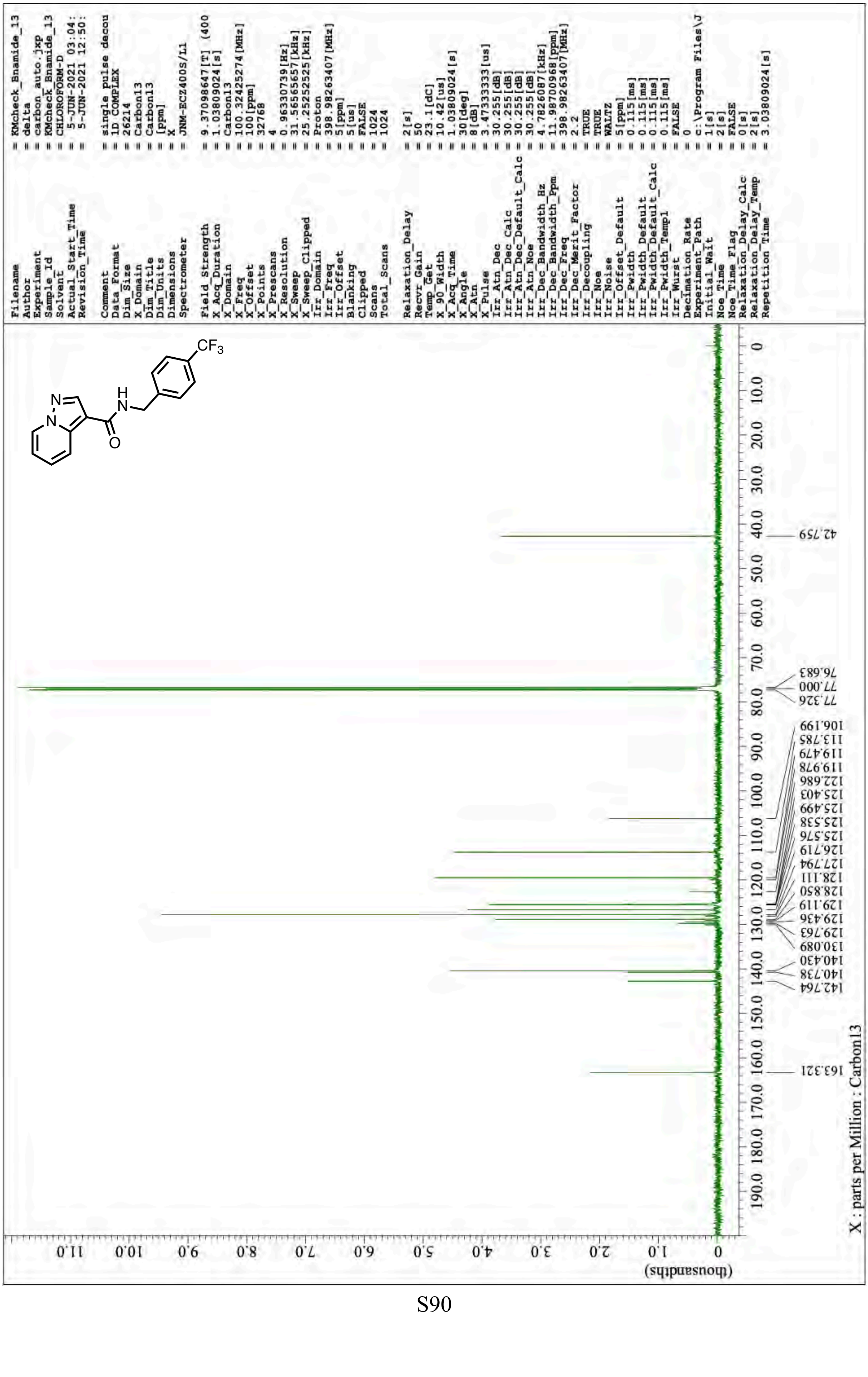

^{19}F NMR of **1Z** (376 MHz, $CDCl_3$)

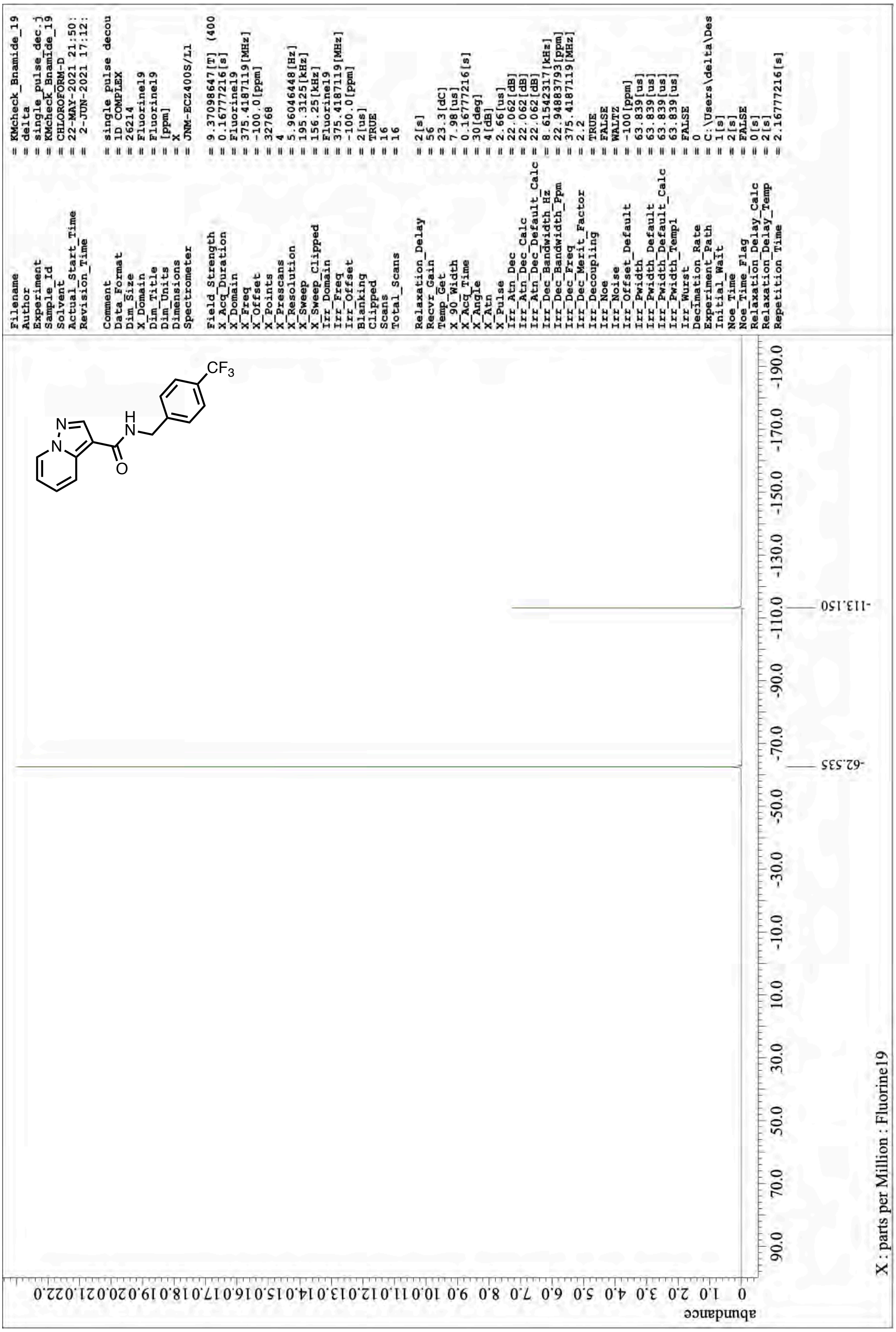

^{1}H NMR of **1AA** (400 MHz, $CDCl_3$)

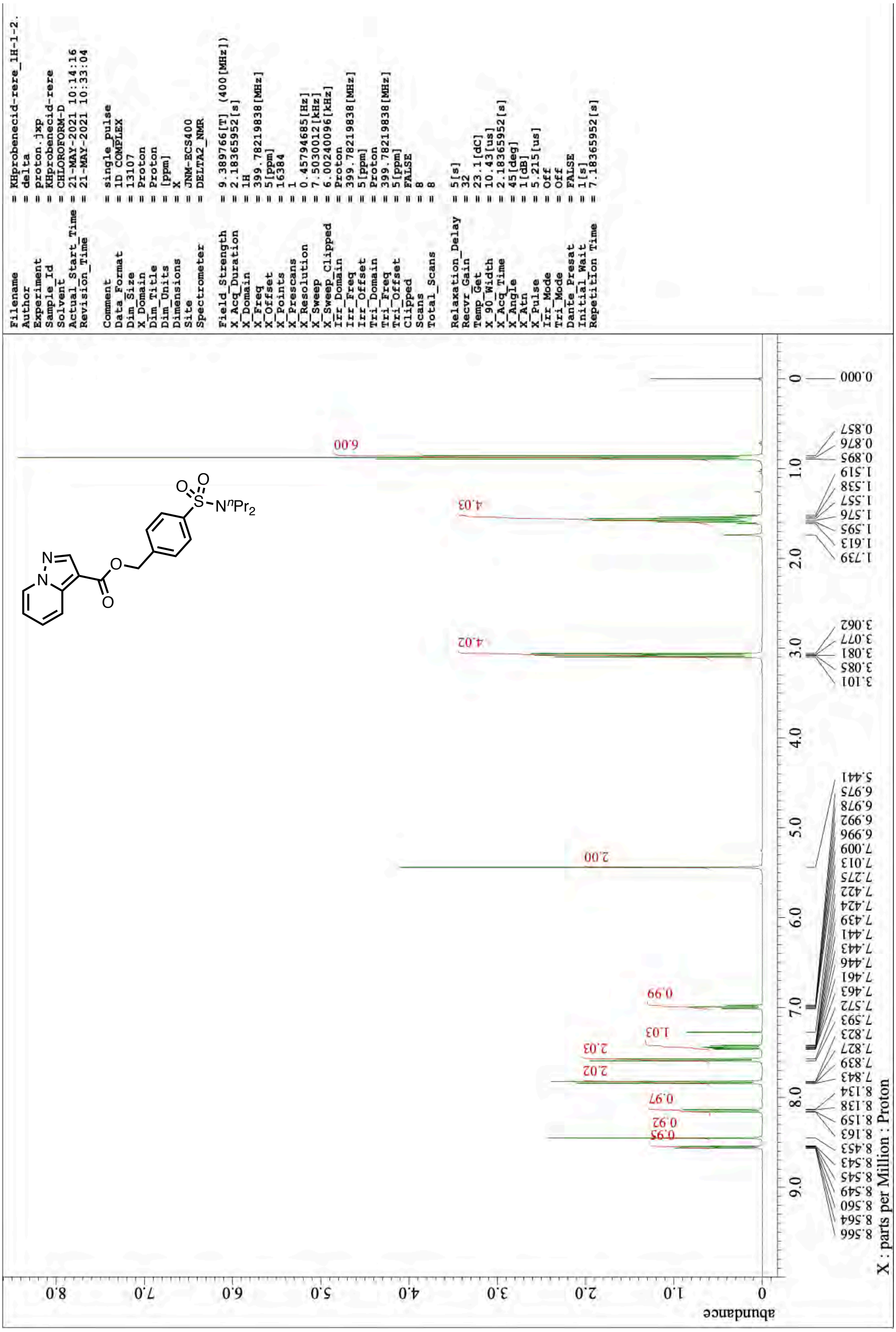

^{13}C NMR of **1AA** (101 MHz, $CDCl_3$)

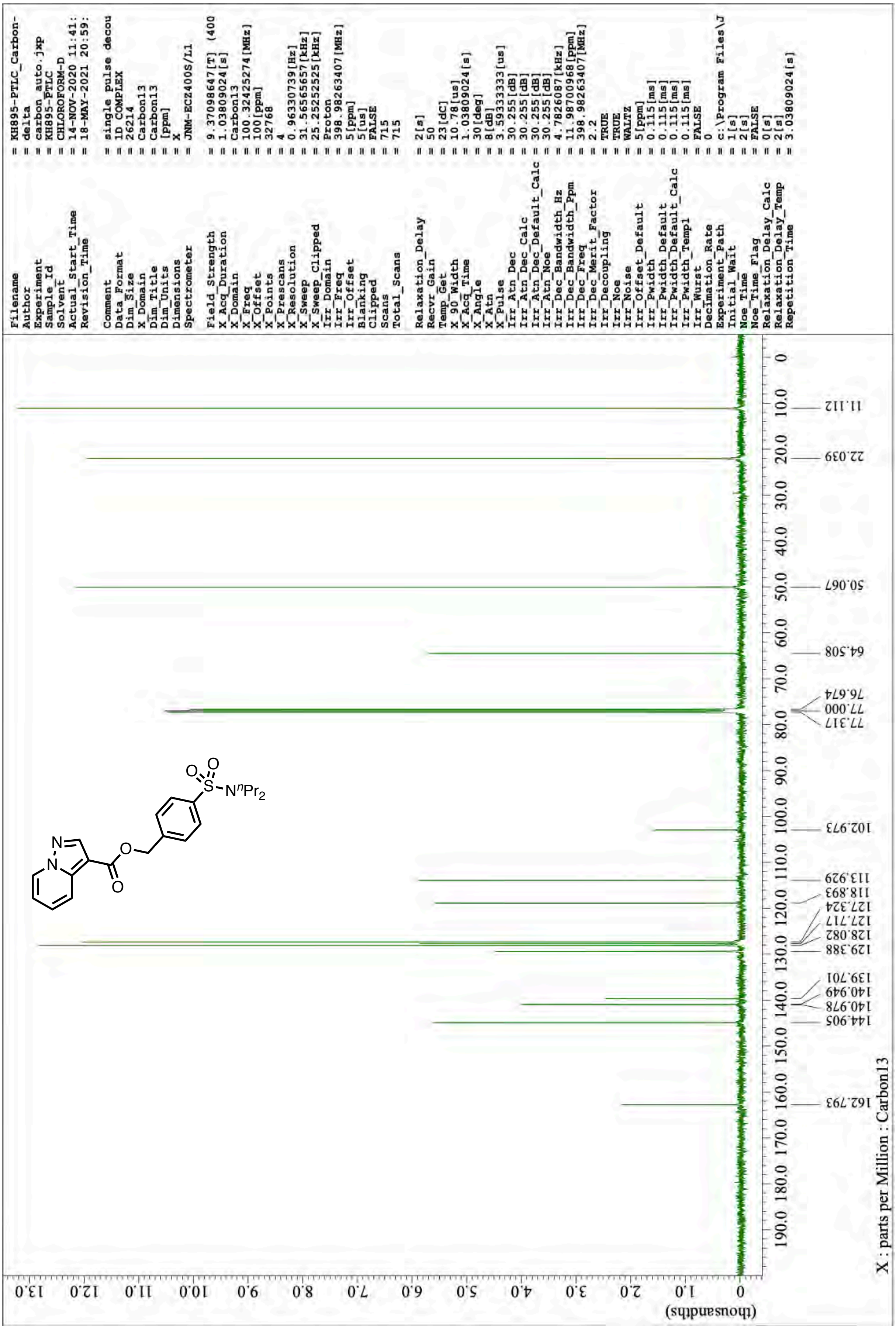

^{1}H NMR of **1AB** (400 MHz, $CDCl_3$)

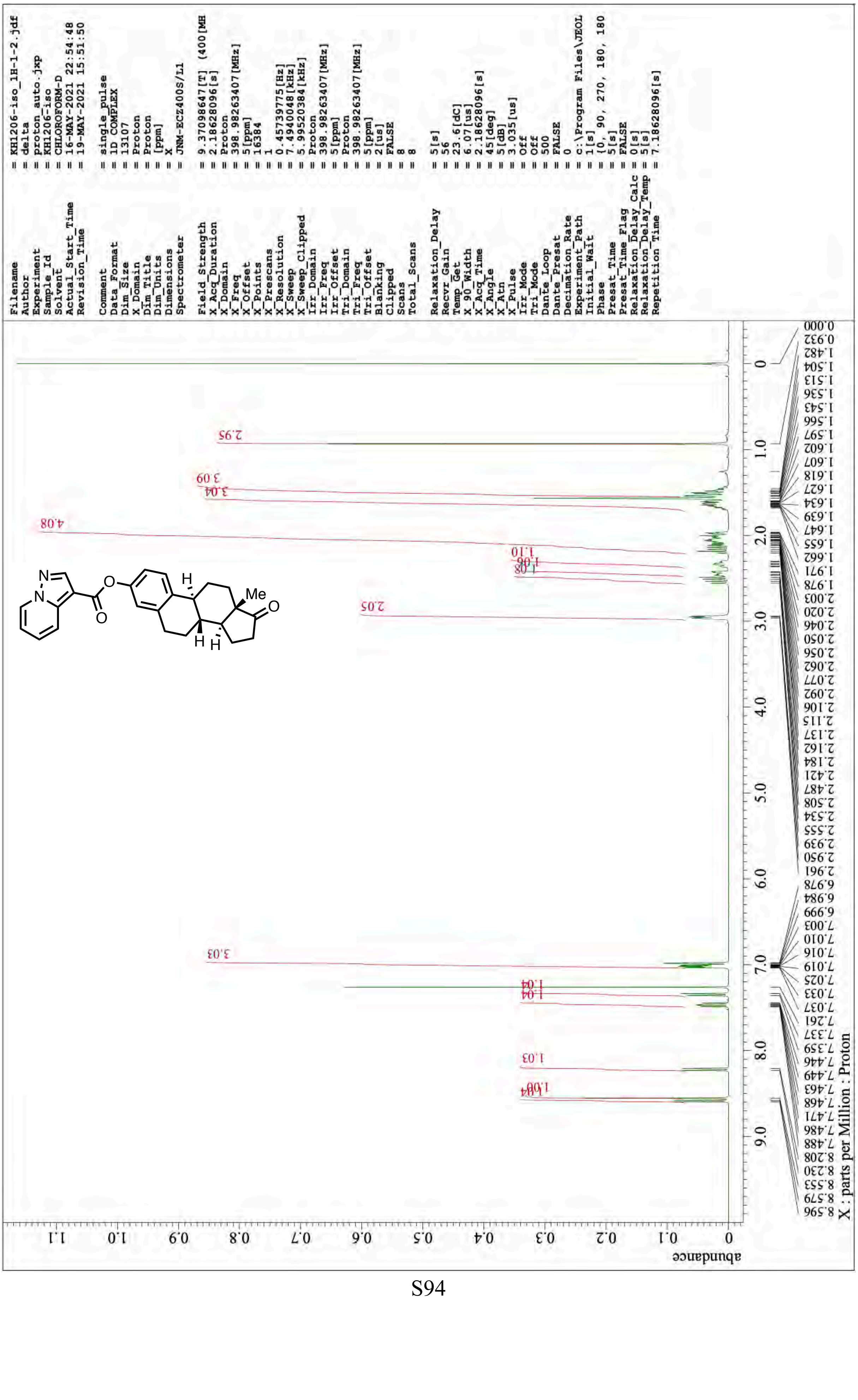

^{13}C NMR of **1AB** (101 MHz, $CDCl_3$)

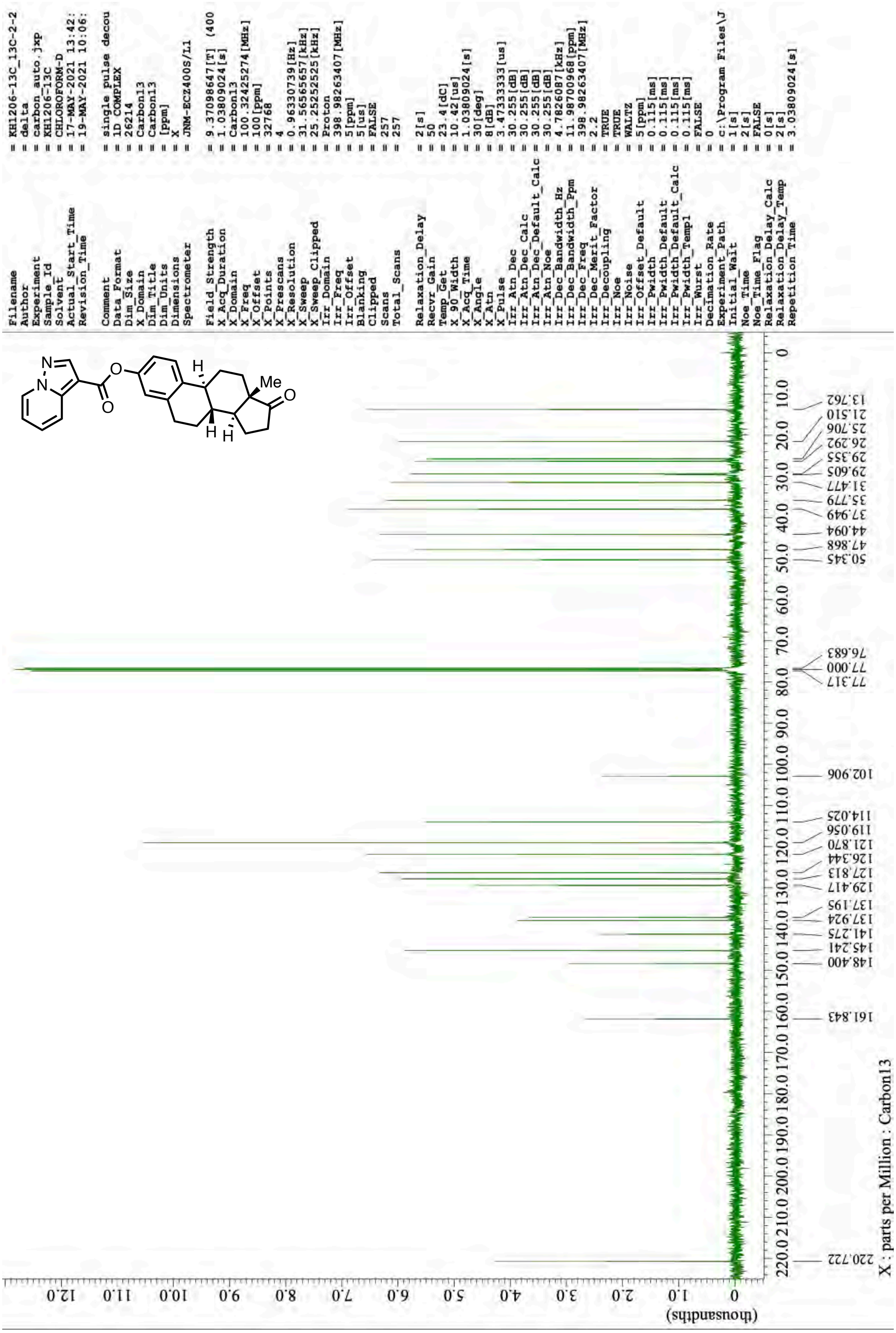

^{1}H NMR of **1AC** (400 MHz, $CDCl_3$)

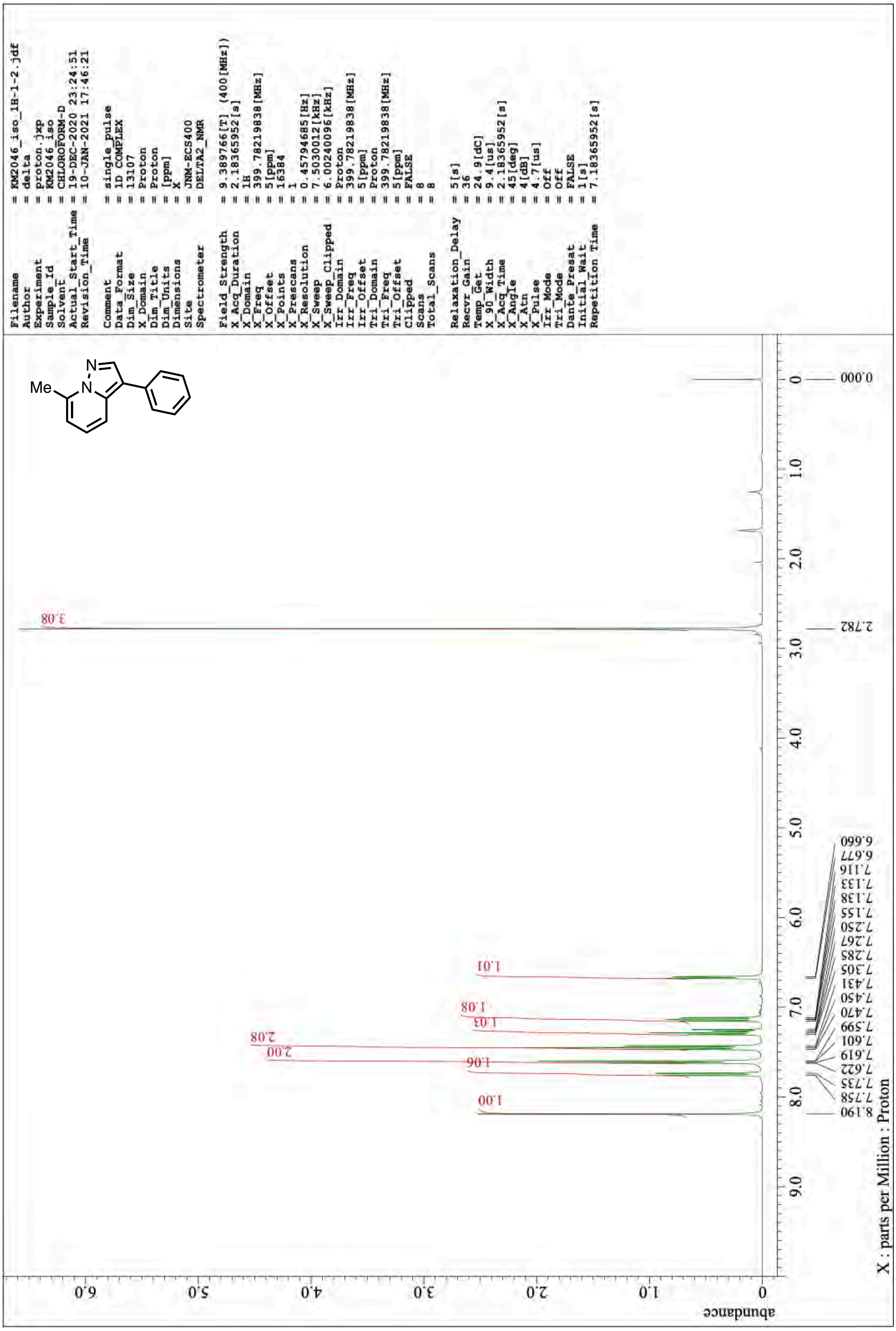

^{13}C NMR of **1AC** (101 MHz, $CDCl_3$)

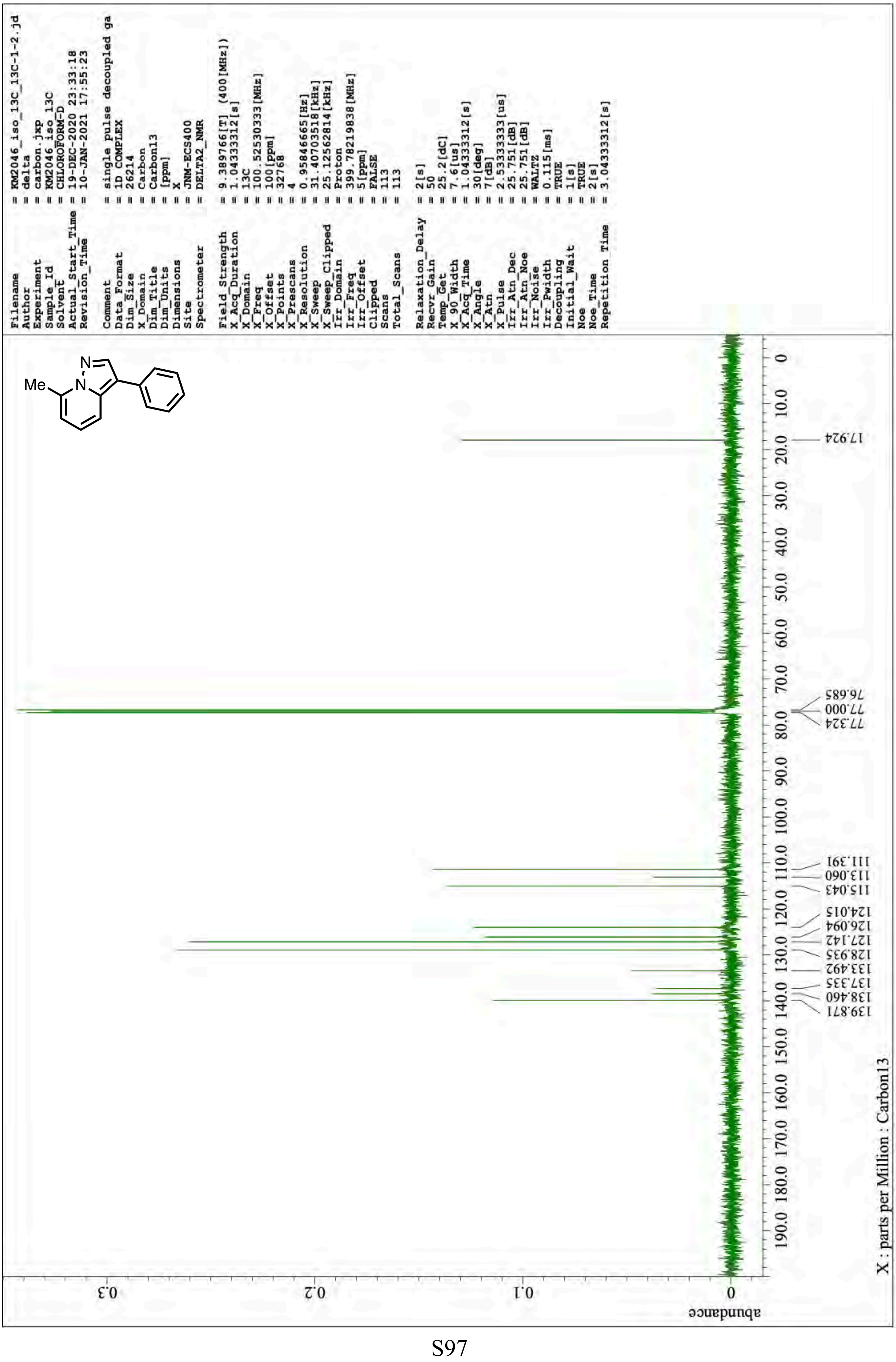

^{1}H NMR of **1AD** (400 MHz, $CDCl_3$)

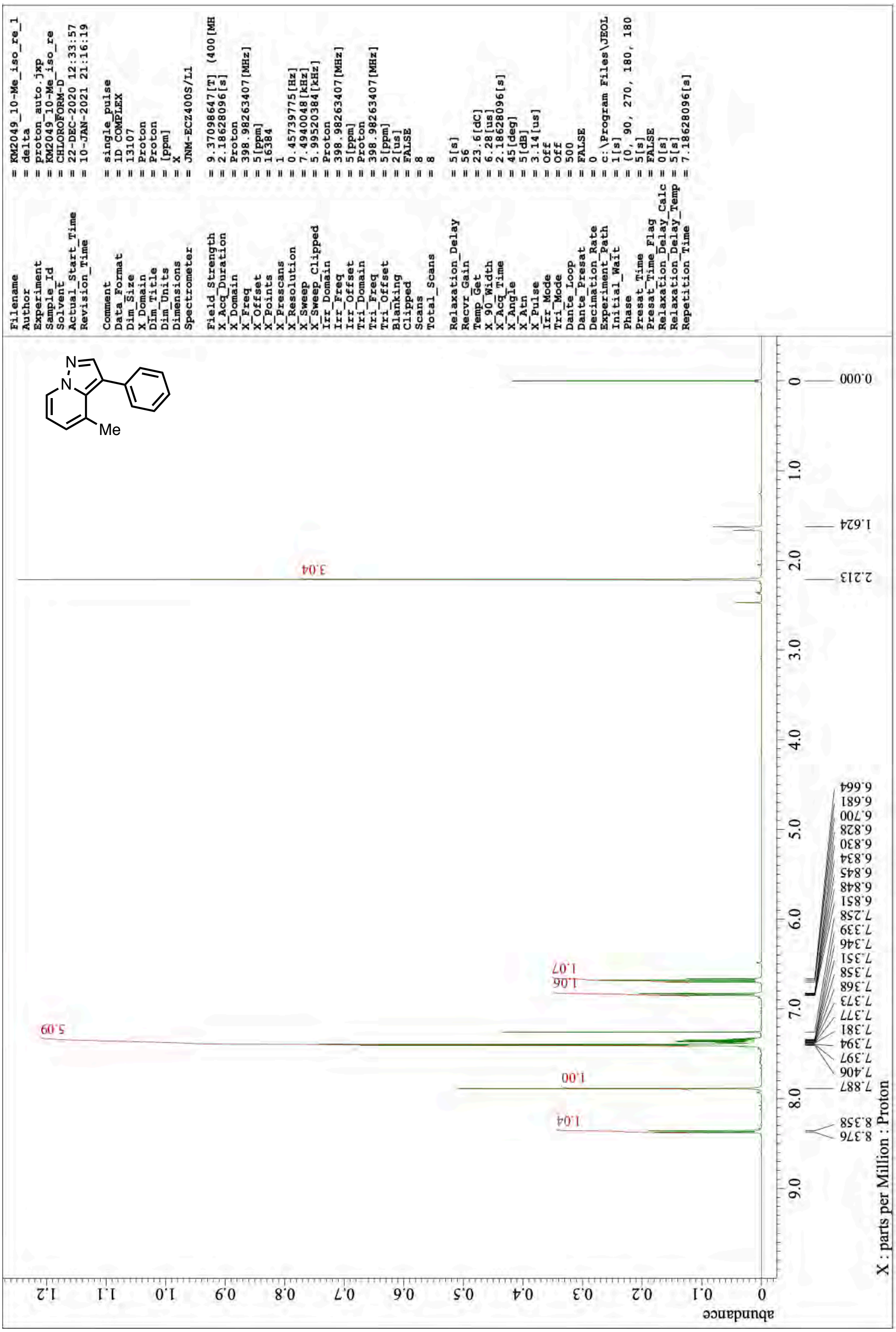

^{13}C NMR of **1AD** (101 MHz, $CDCl_3$)

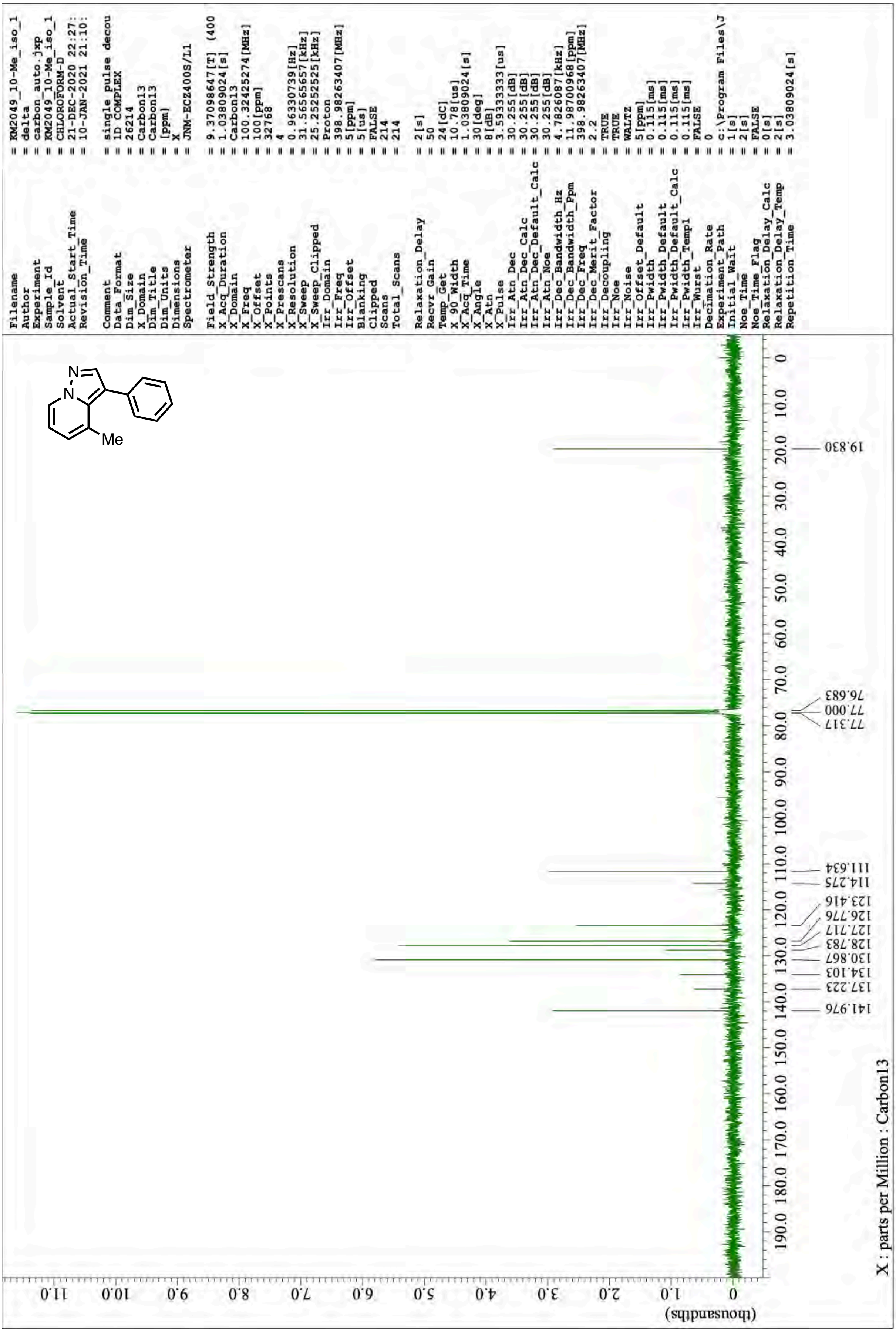

^{1}H NMR of **1AE** (400 MHz, $CDCl_3$)

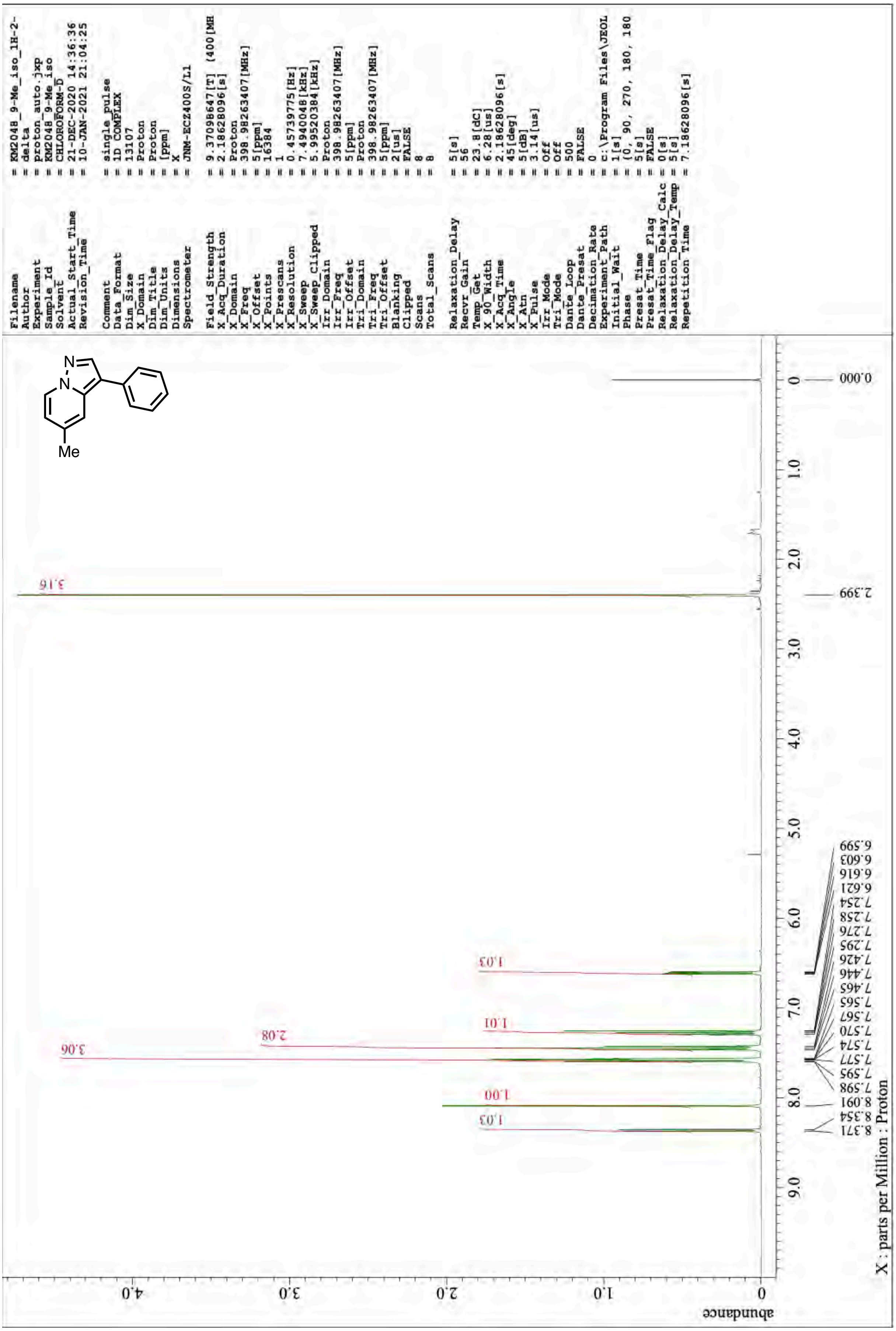

^{13}C NMR of **1AE** (101 MHz, $CDCl_3$)

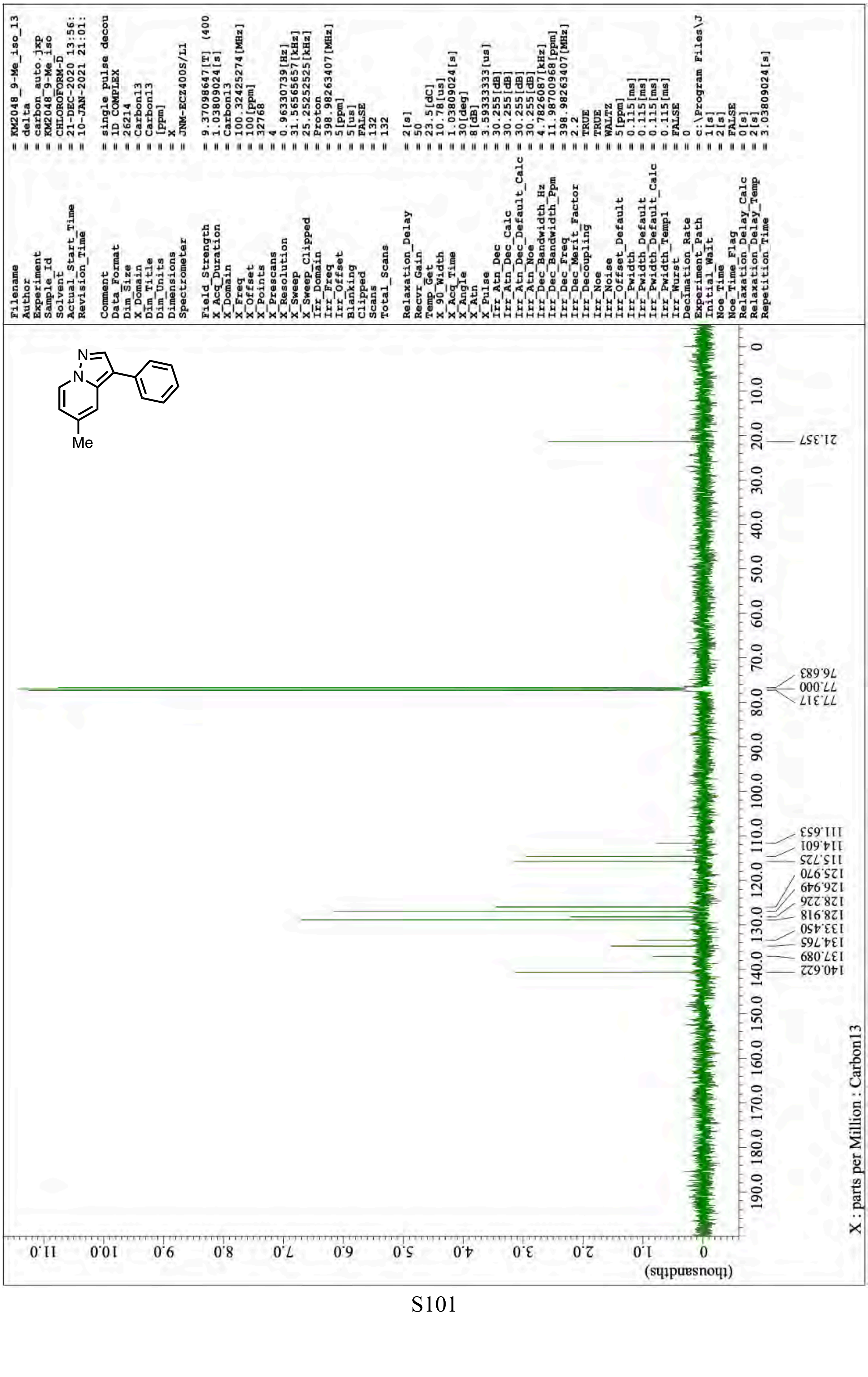

^{1}H NMR of **1AF** (400 MHz, $CDCl_3$)

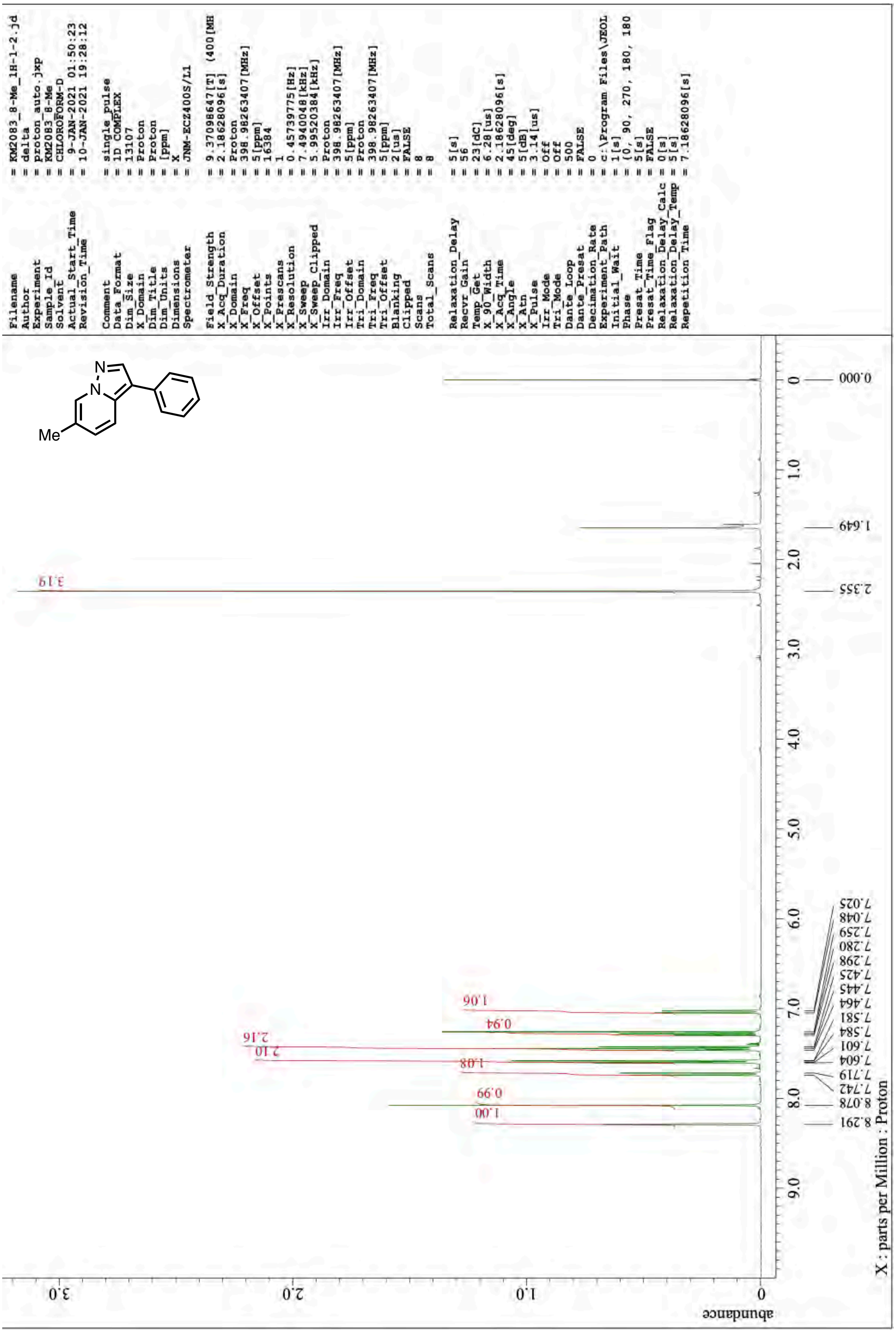

^{13}C NMR of **1AF** (101 MHz, $CDCl_3$)

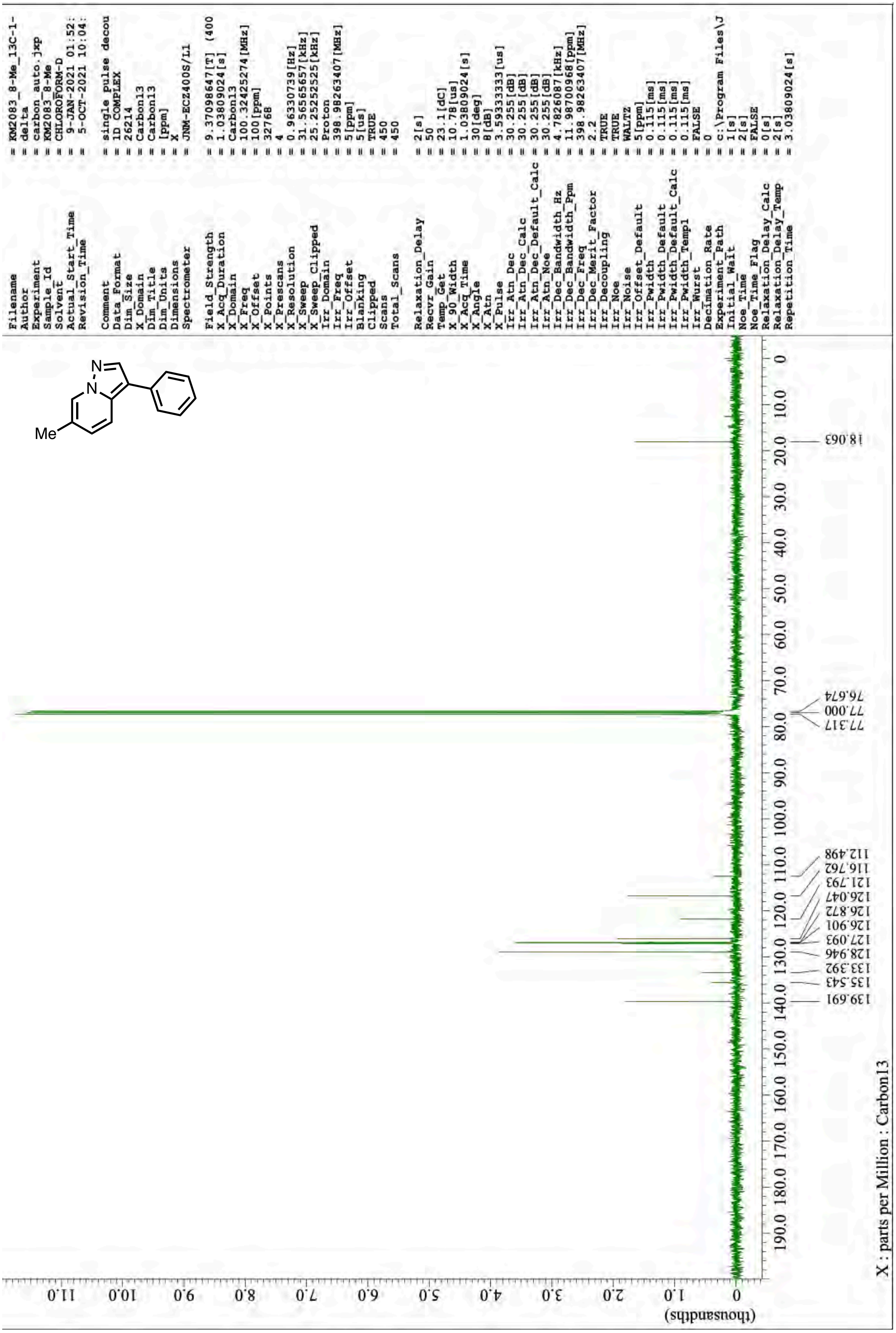

^{1}H NMR of **1AH** (400 MHz, $CDCl_3$)

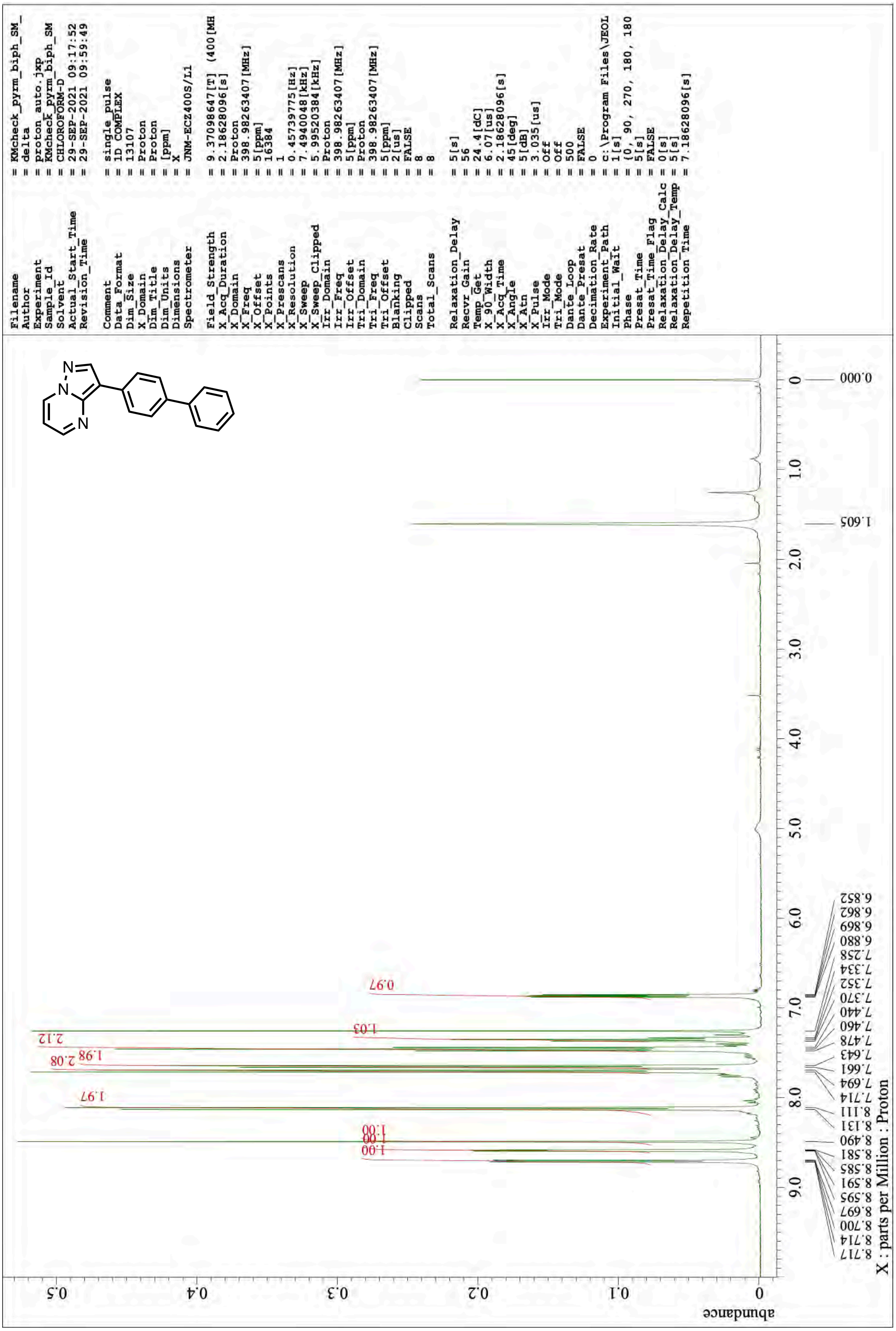

^{13}C NMR of **1AH** (101 MHz, $CDCl_3$)

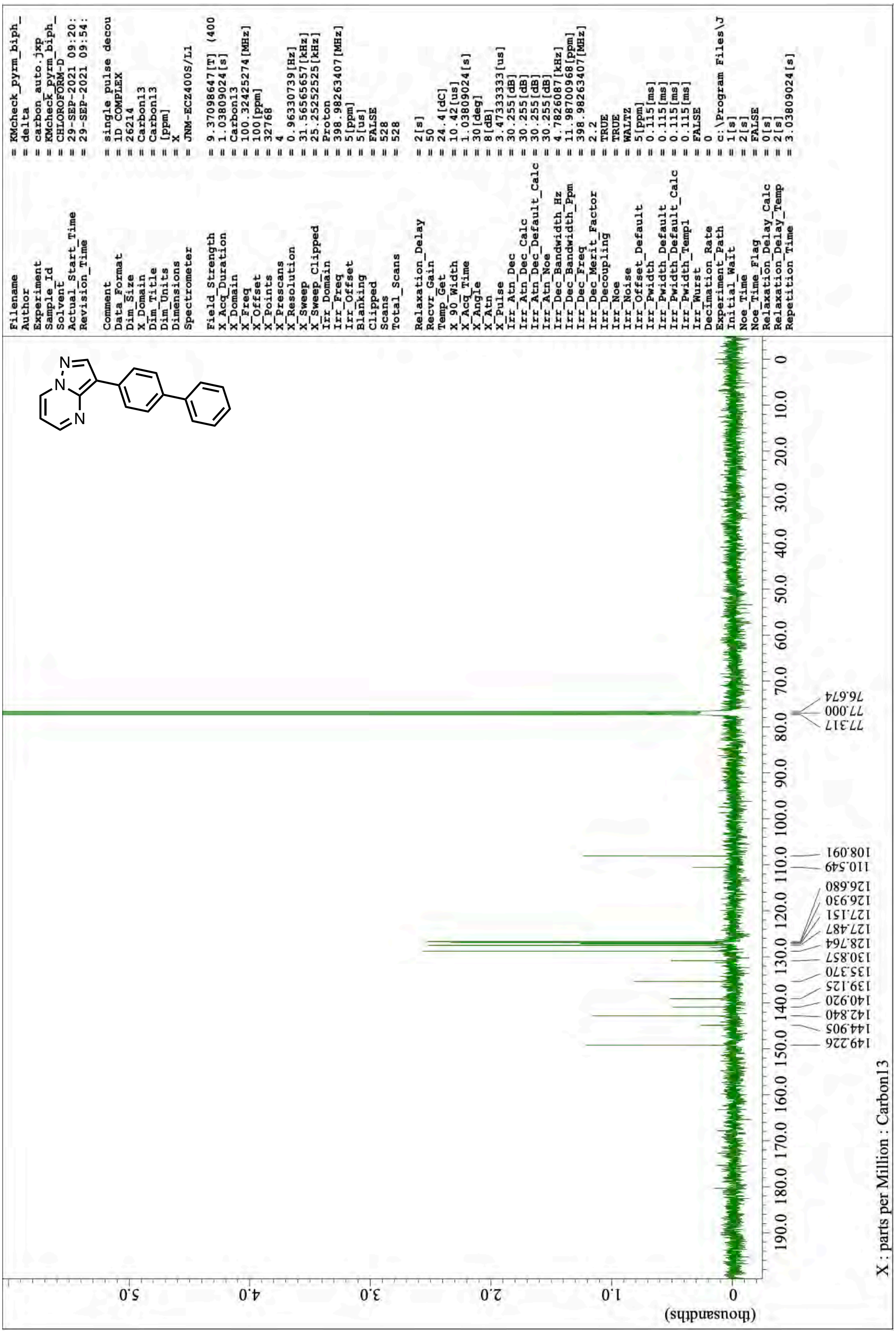

^{1}H NMR of **1AI** (400 MHz, $CDCl_3$)

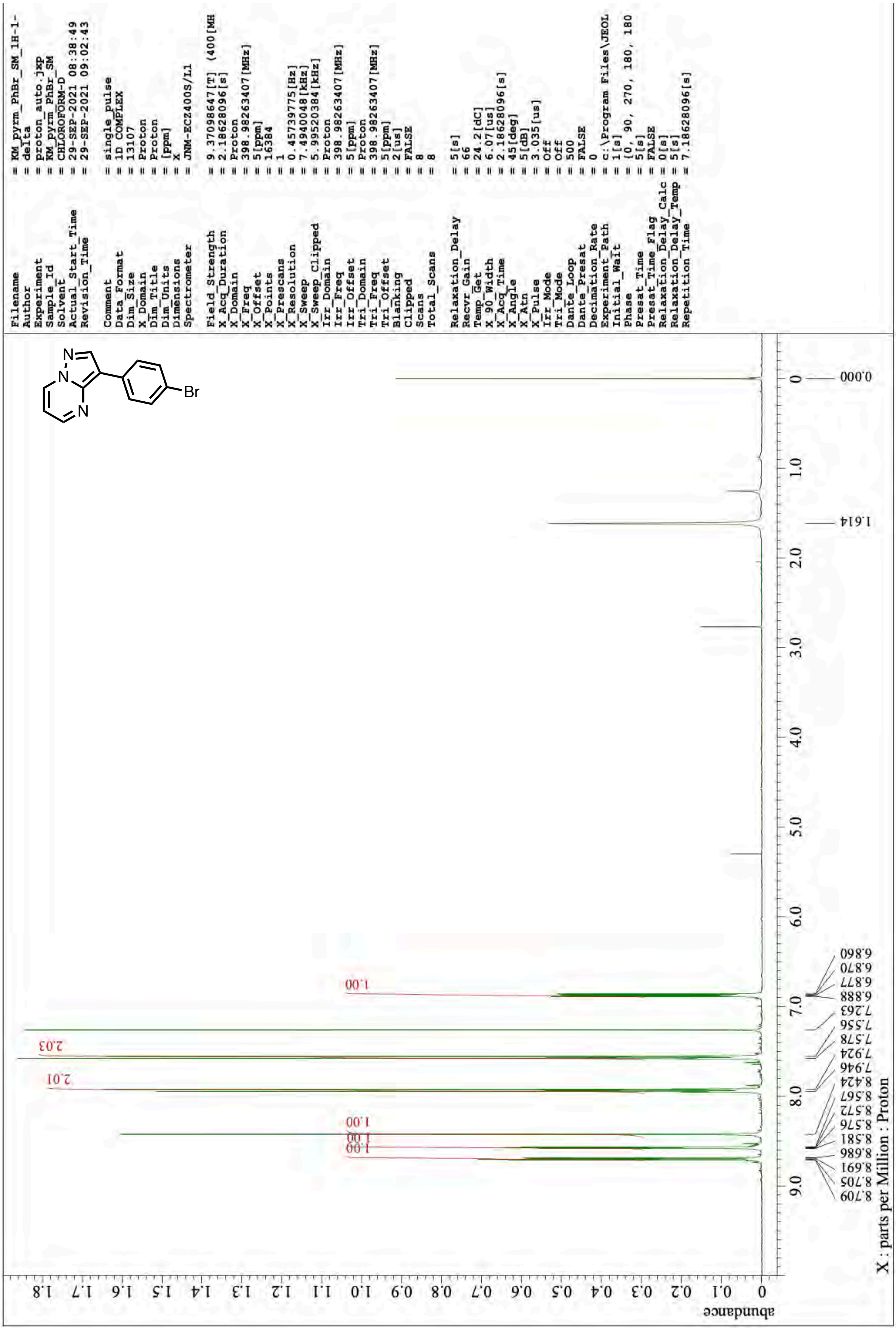

^{13}C NMR of **1AI** (101 MHz, $CDCl_3$)

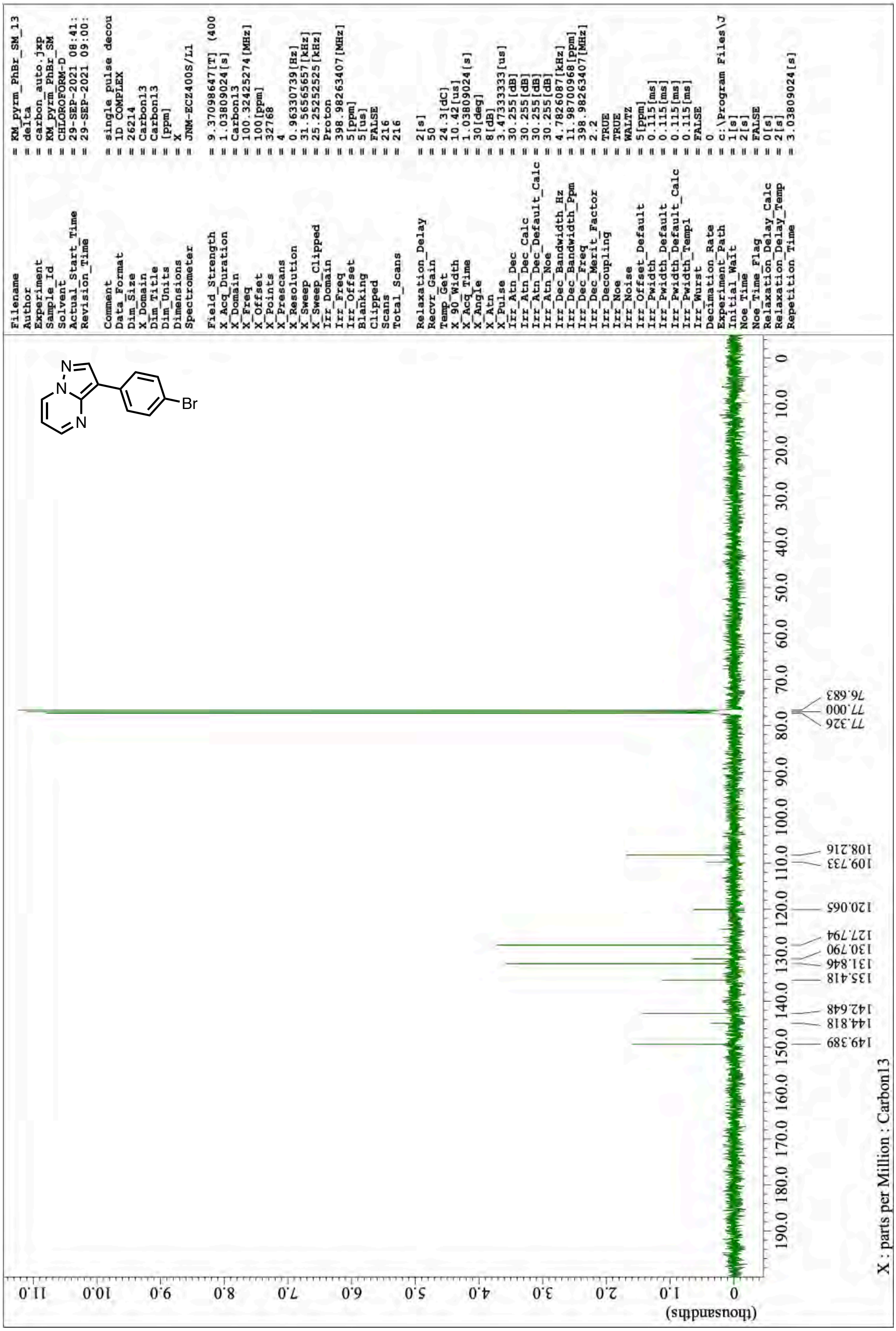

^{1}H NMR of **1AJ** (400 MHz, $CDCl_3$)

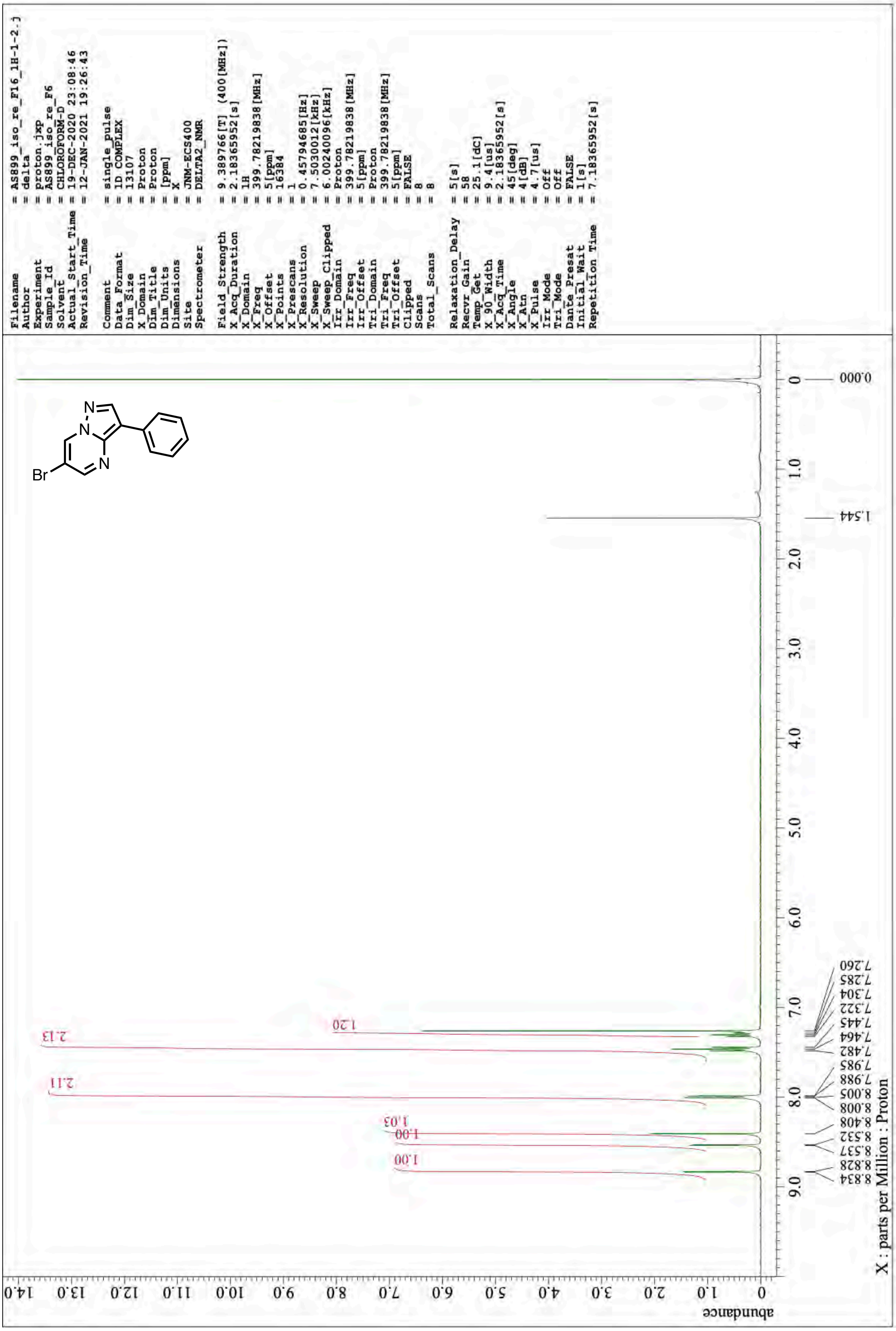

^{13}C NMR of **1AJ** (101 MHz, $CDCl_3$)

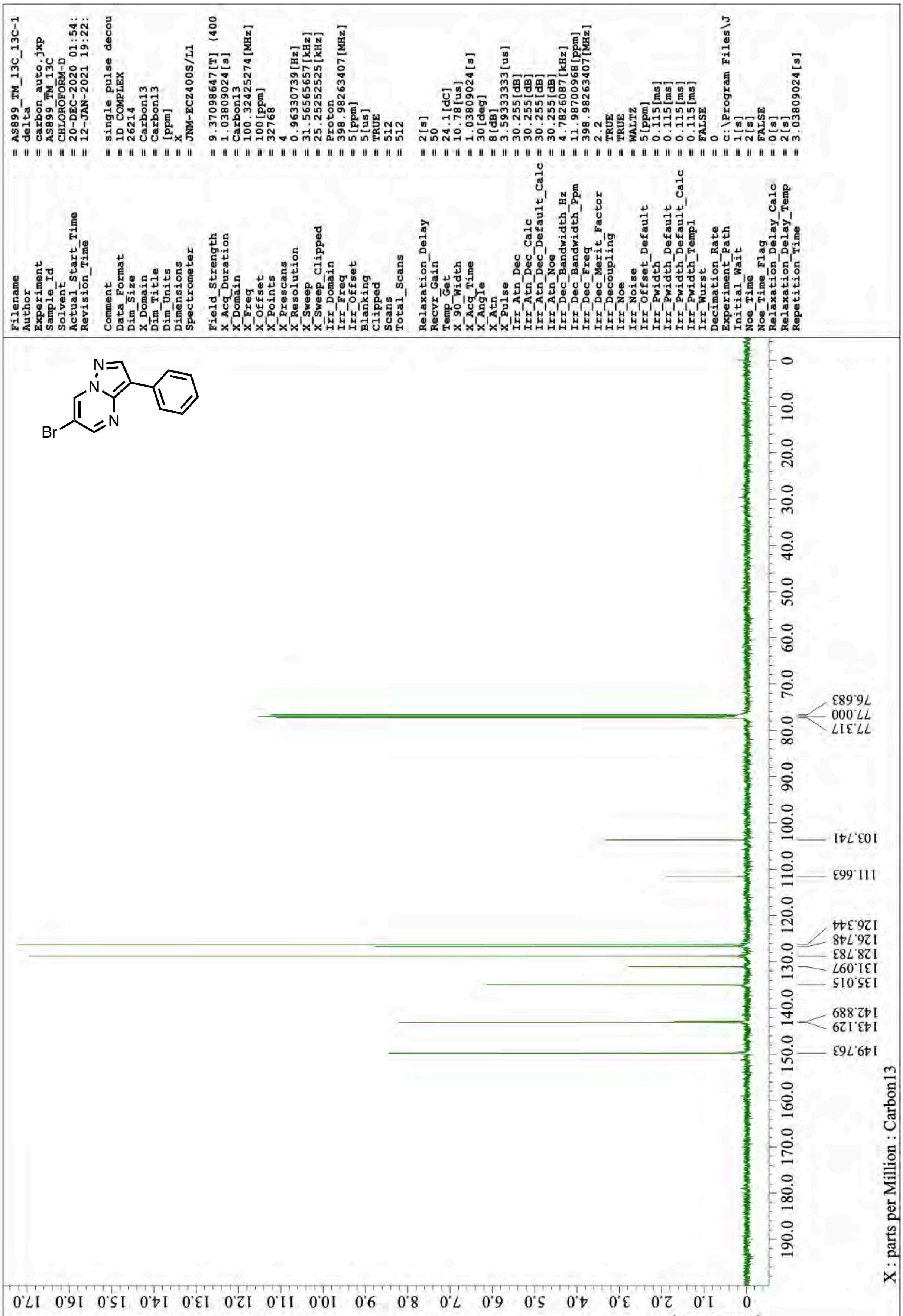

^{1}H NMR of **1AK** (400 MHz, $CDCl_3$)

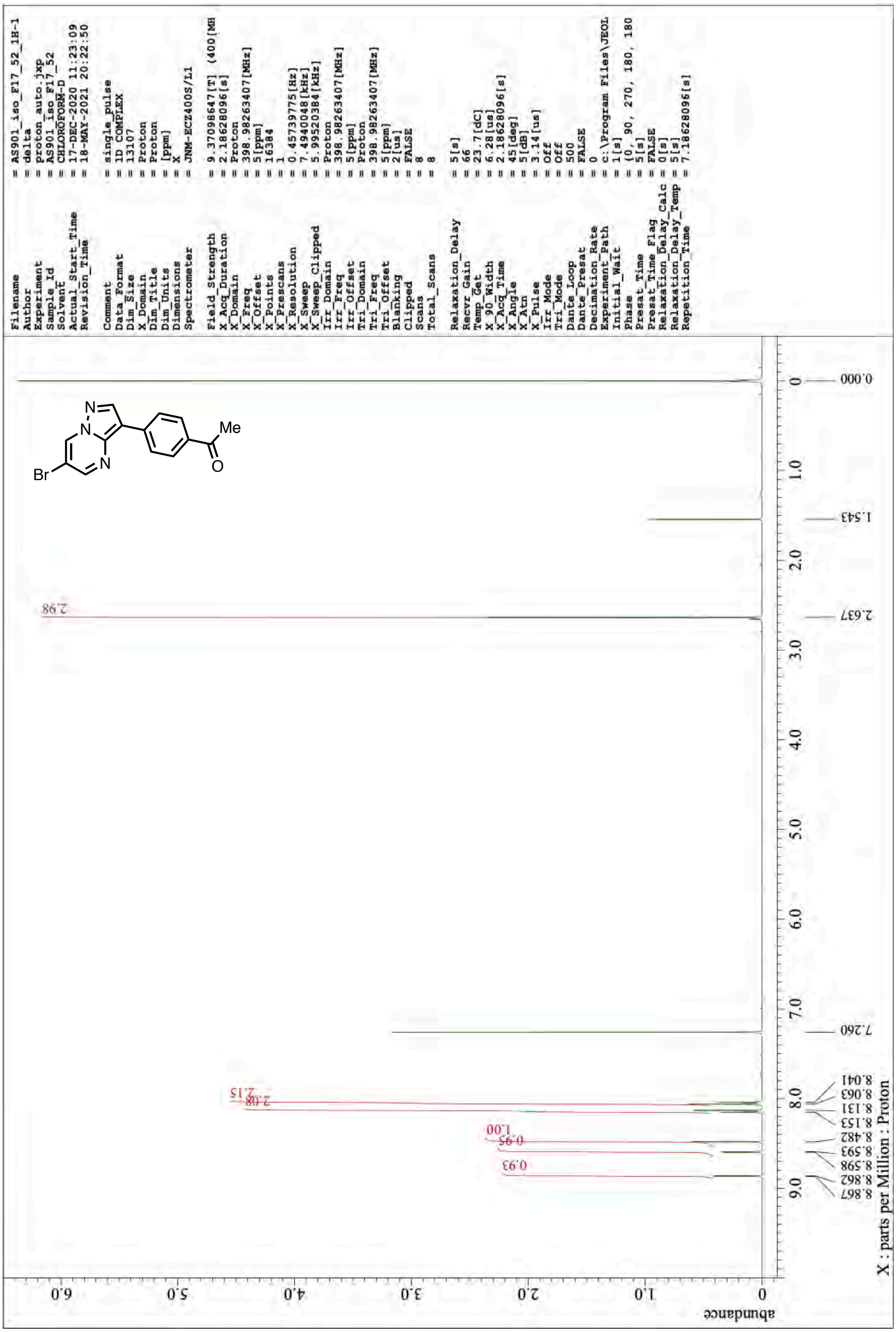

^{13}C NMR of **1AK** (101 MHz, $CDCl_3$)

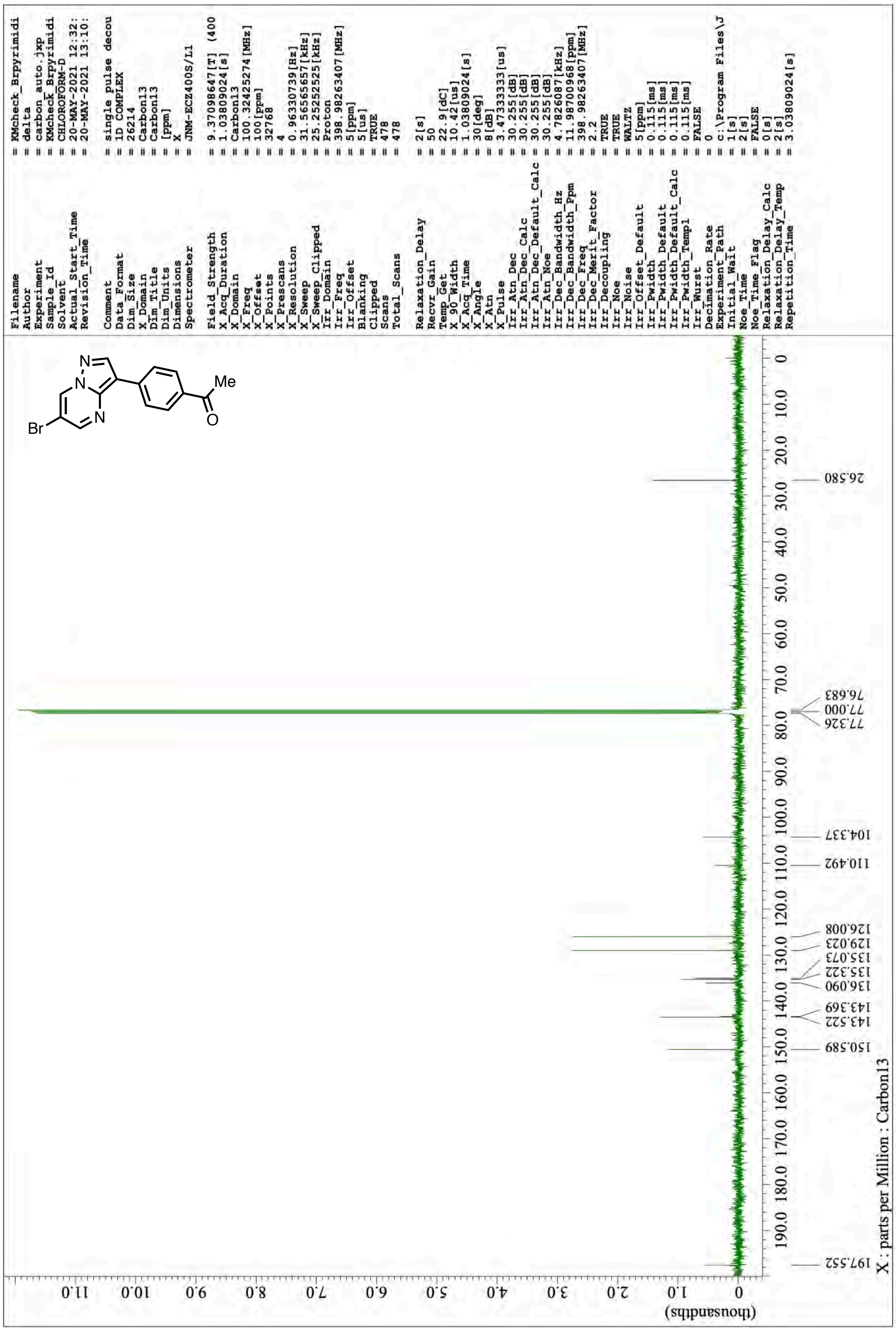

^{1}H NMR of **1AL** (400 MHz, $CDCl_3$)

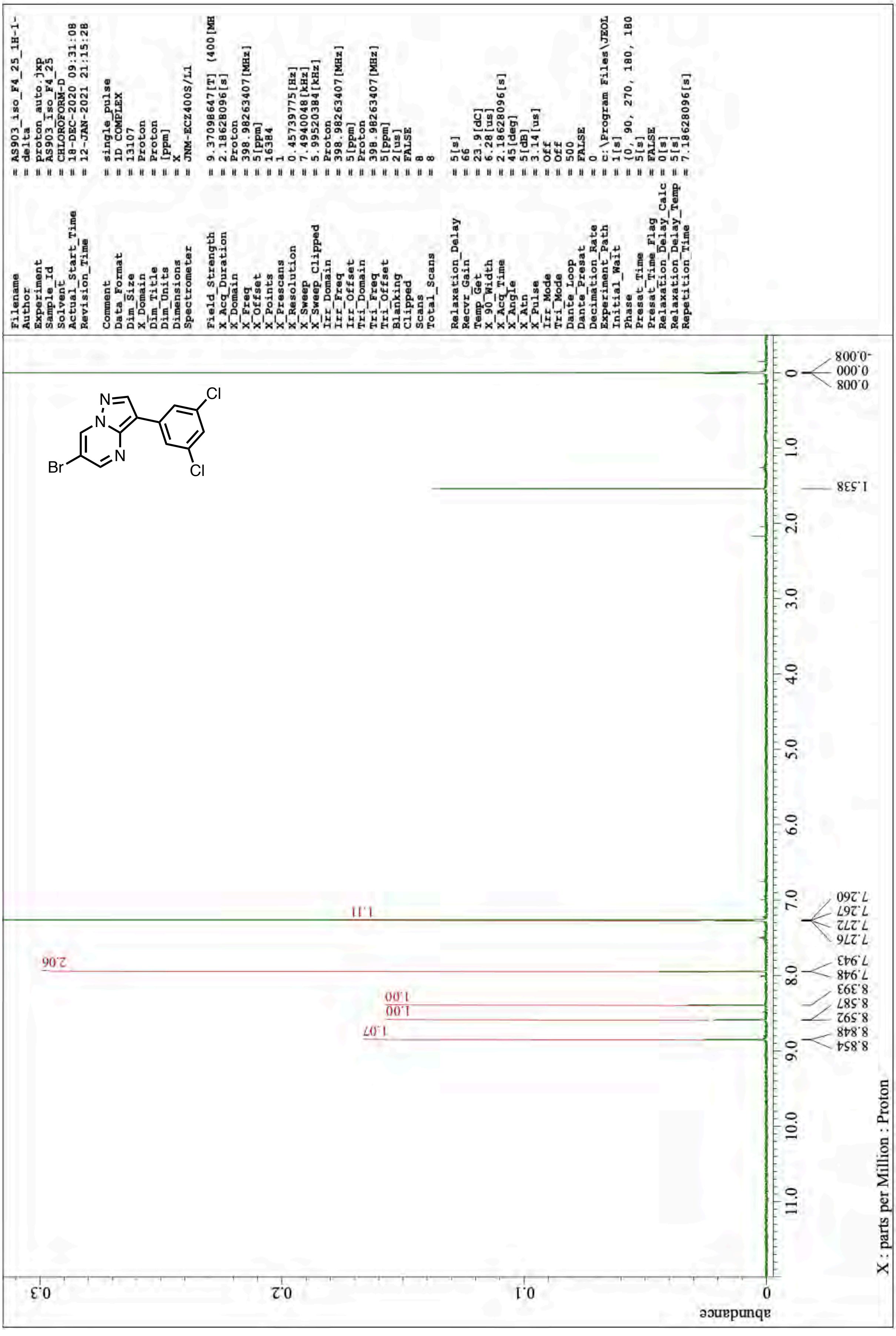

^{13}C NMR of **1AL** (101 MHz, $CDCl_3$)

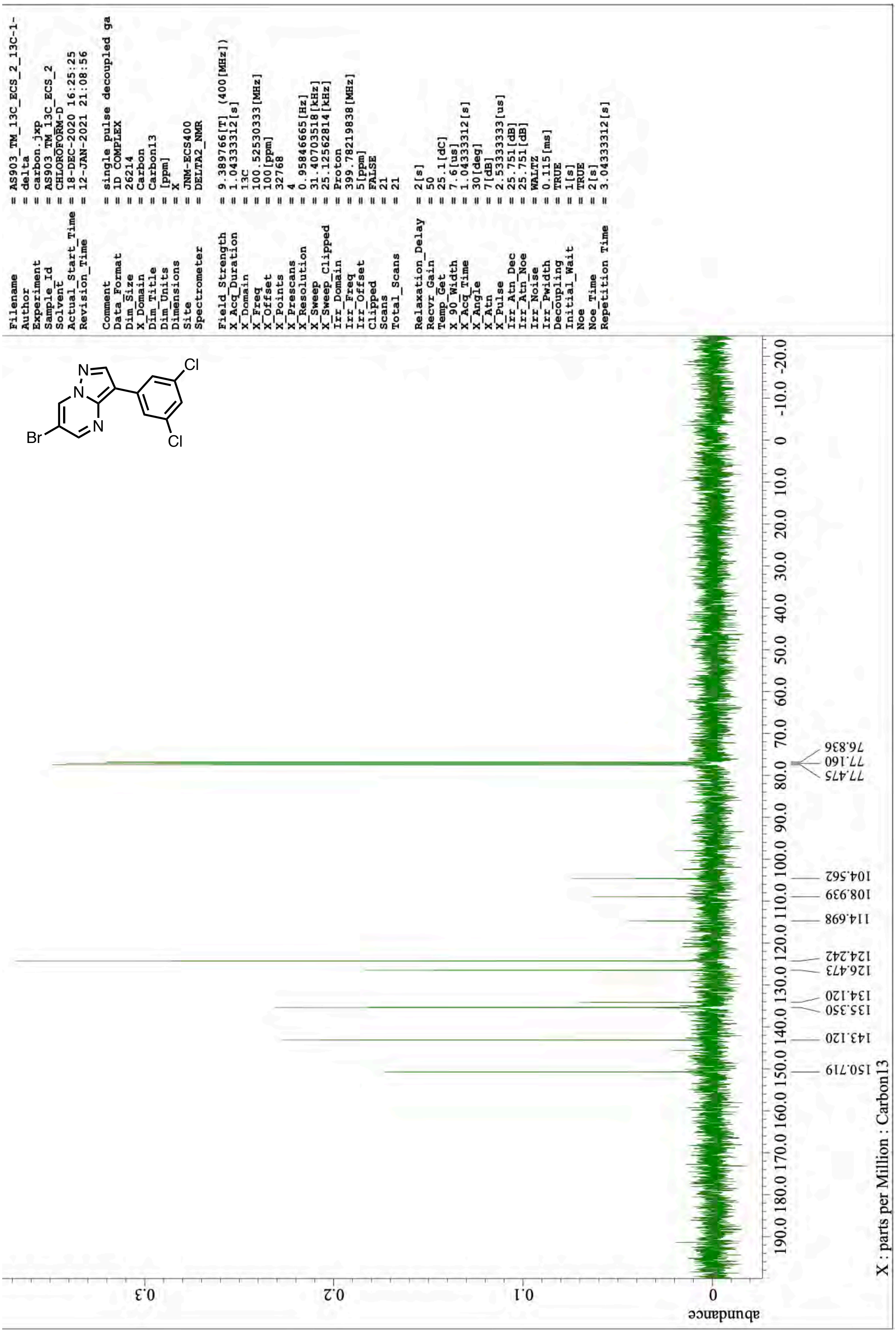

^{1}H NMR of **1AN** (400 MHz, $CDCl_3$)

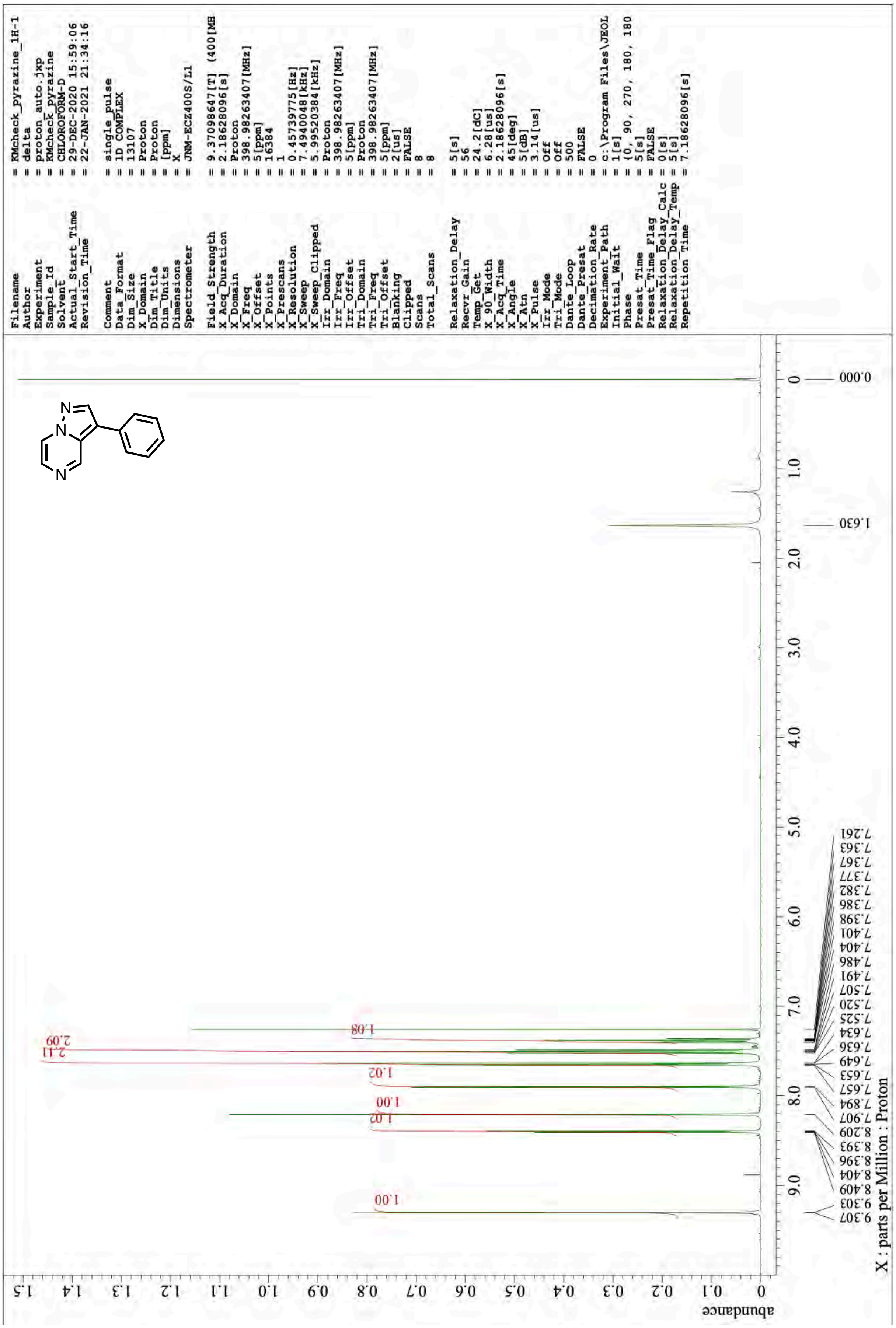

^{13}C NMR of **1AN** (101 MHz, $CDCl_3$)

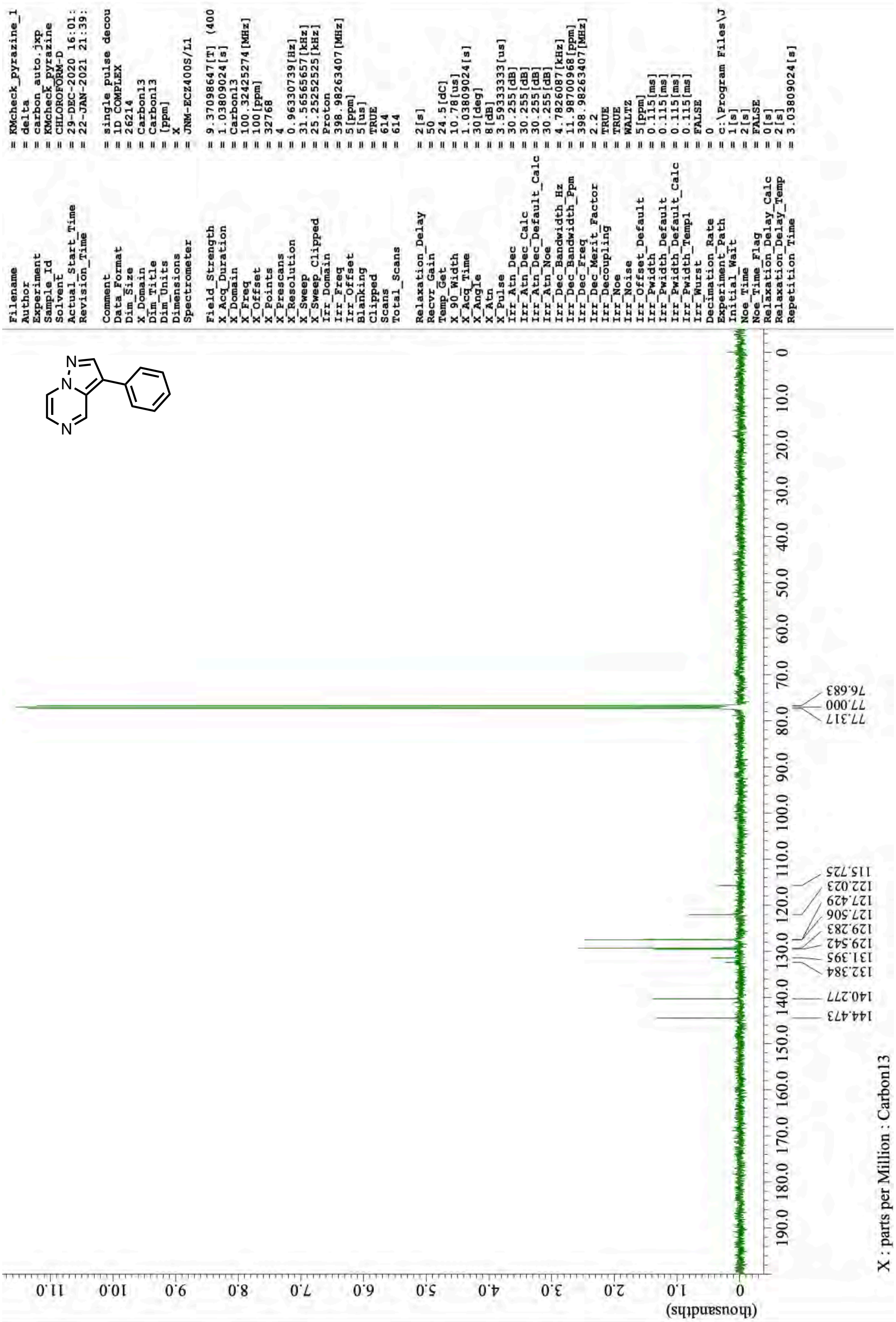

^{1}H NMR of **1AO** (400 MHz, $CDCl_3$)

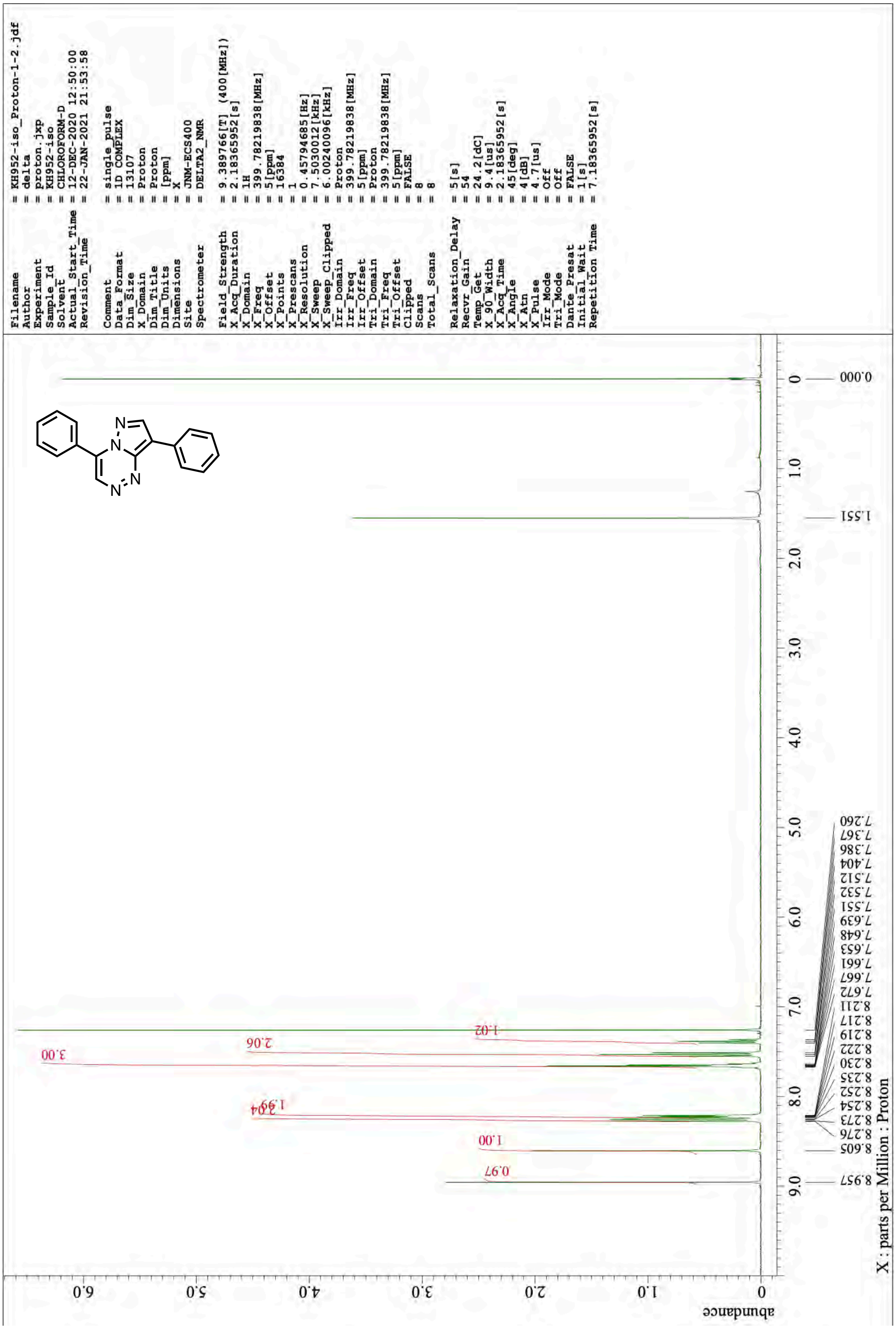

^{13}C NMR of **1AO** (101 MHz, $CDCl_3$)

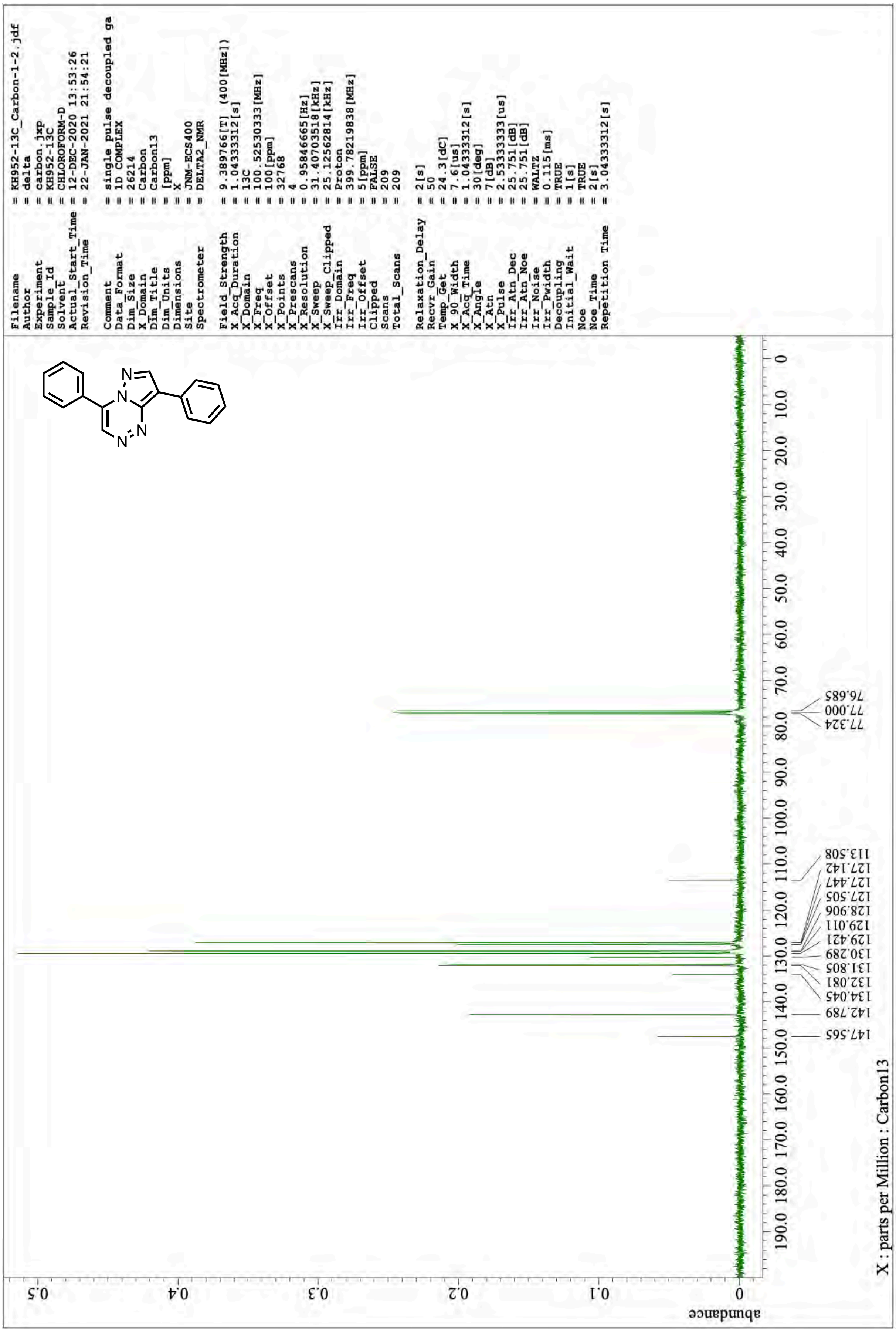

^{1}H NMR of **1AP** (400 MHz, $CDCl_3$)

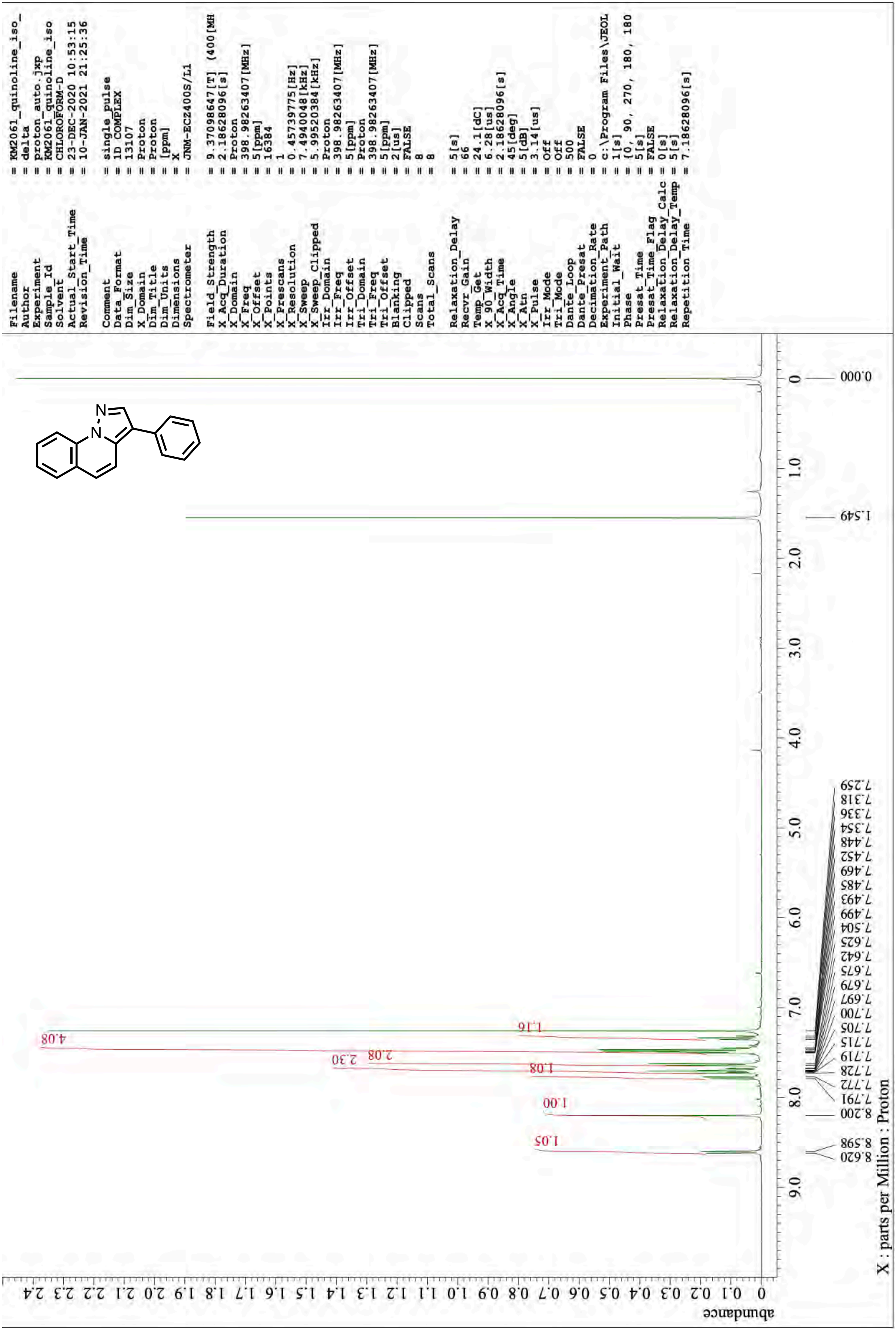

^{13}C NMR of **1AP** (101 MHz, $CDCl_3$)

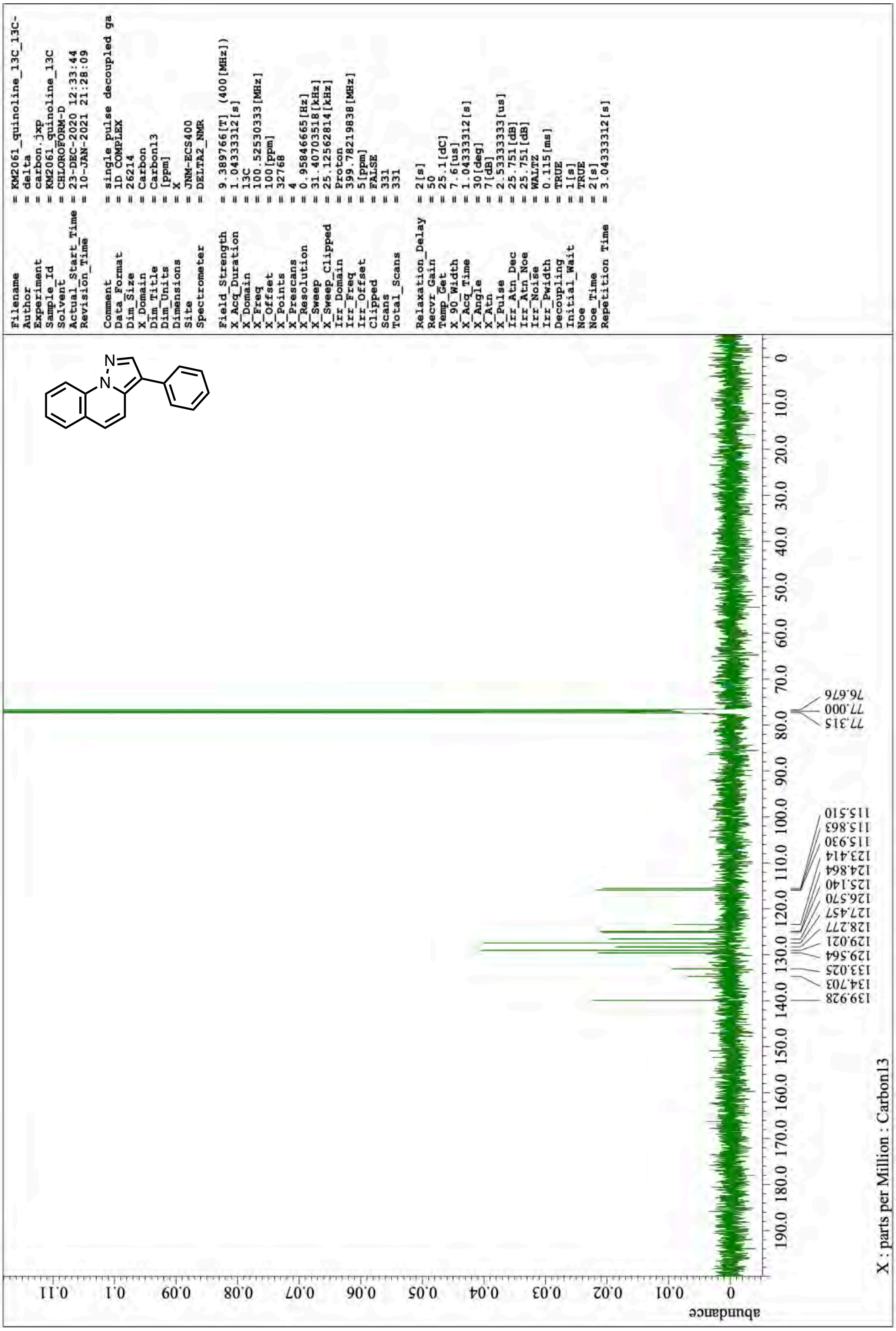

^{1}H NMR of **1AQ** (400 MHz, $CDCl_3$)

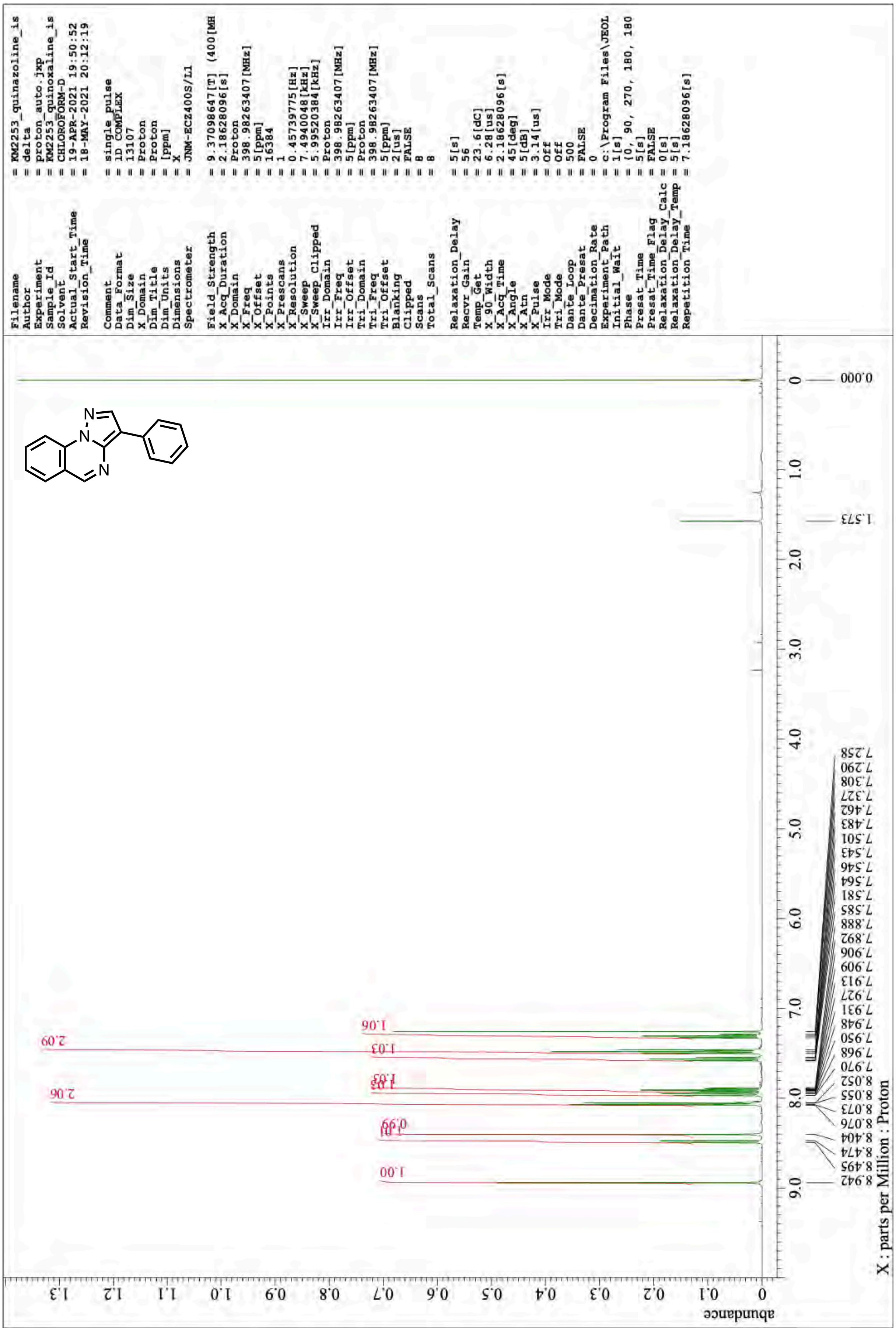

^{13}C NMR of **1AQ** (101 MHz, $CDCl_3$)

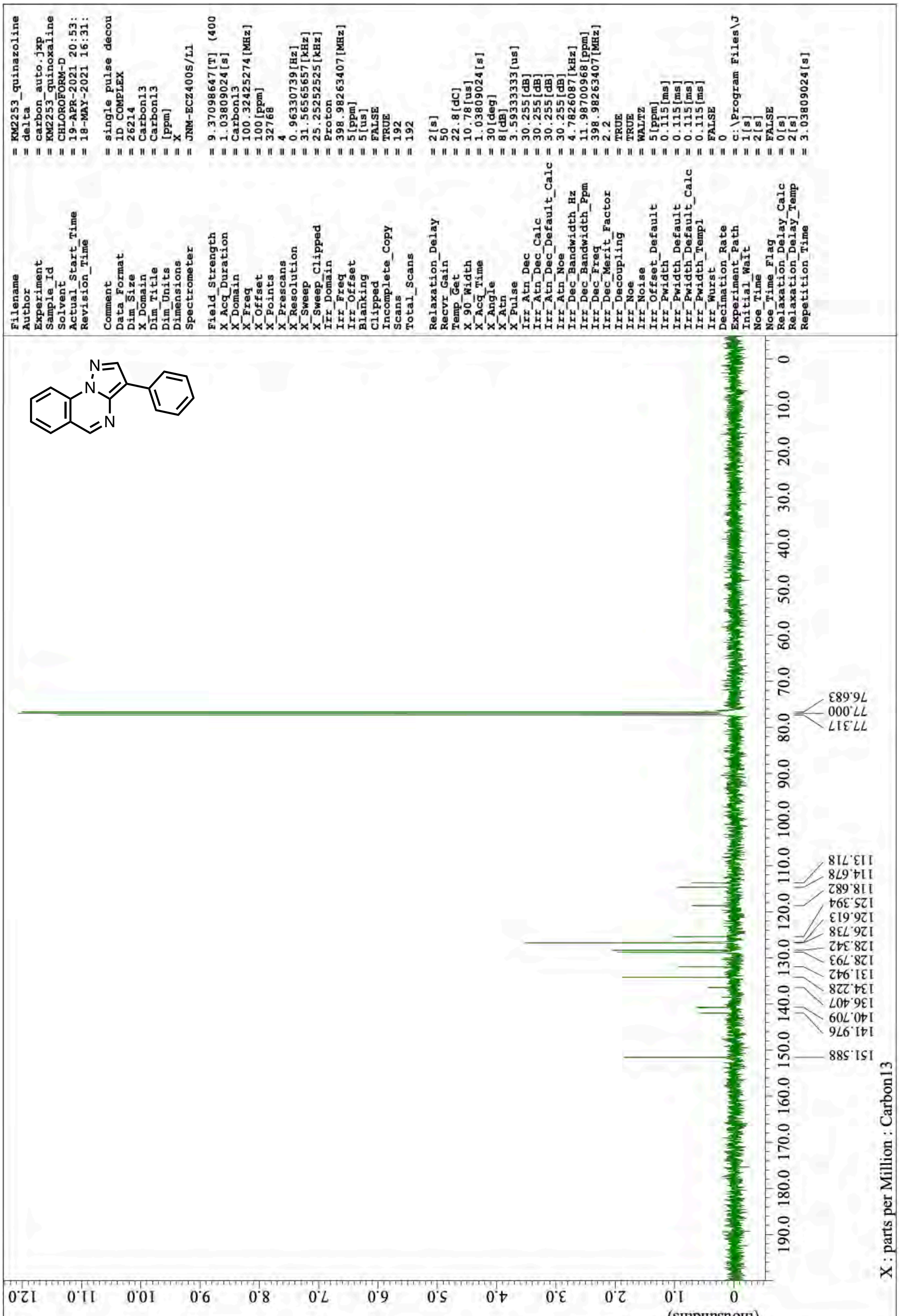

^{1}H NMR of **2A** (400 MHz, $CDCl_3$)

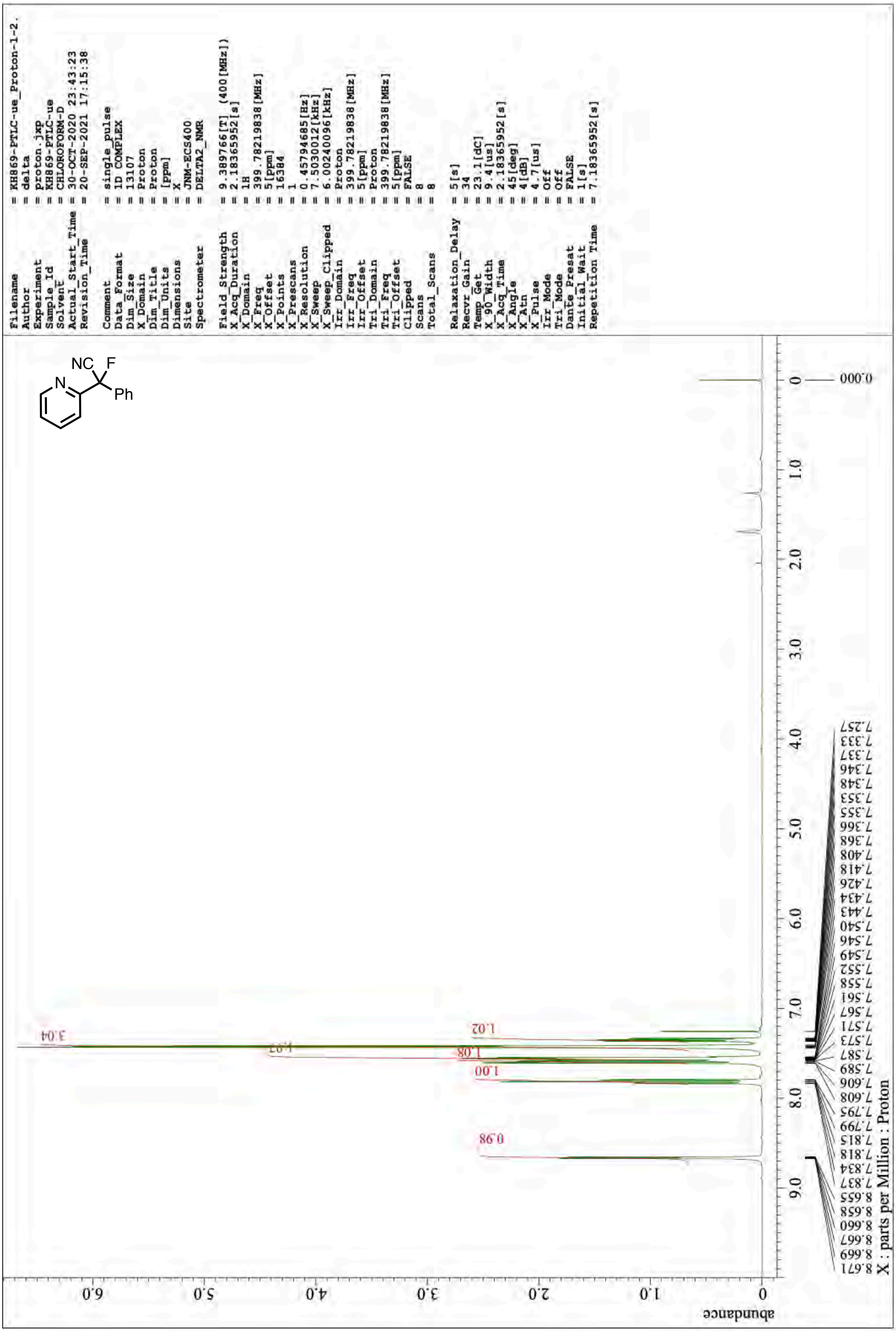

^{13}C NMR of **2A** (101 MHz, $CDCl_3$)

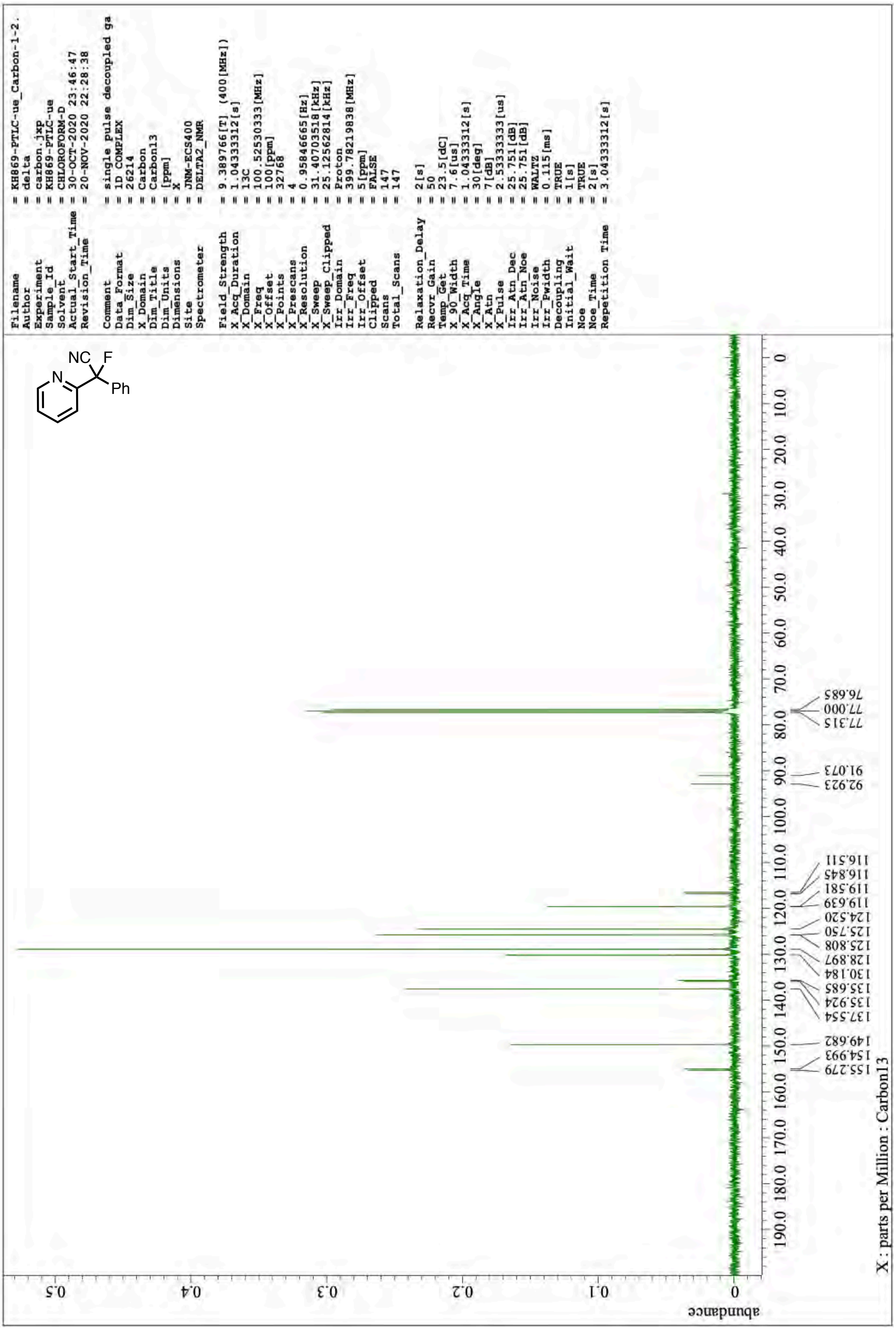

^{19}F NMR of **2A** (376 MHz, $CDCl_3$)

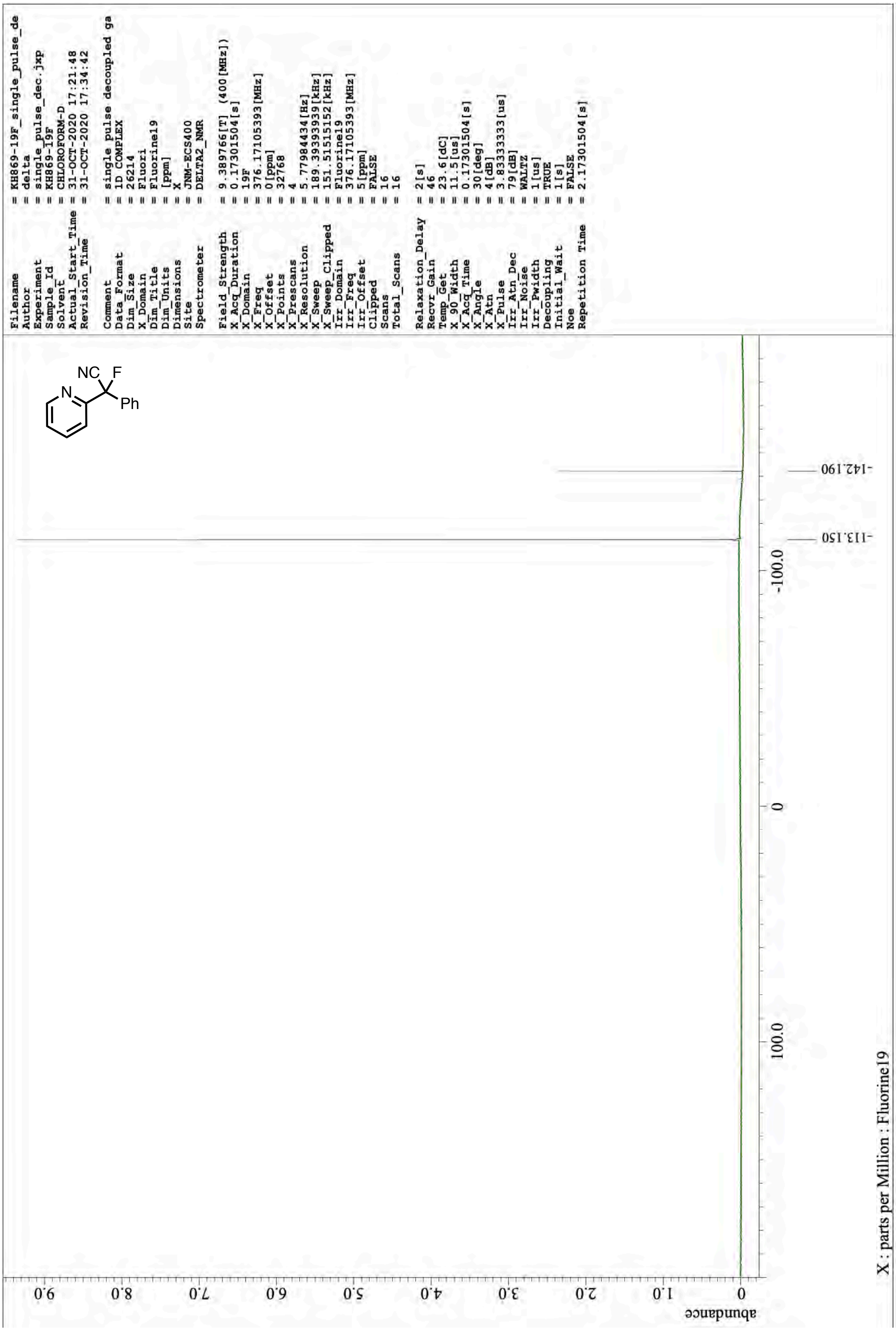

^{1}H NMR of **2B** (400 MHz, $CDCl_3$)

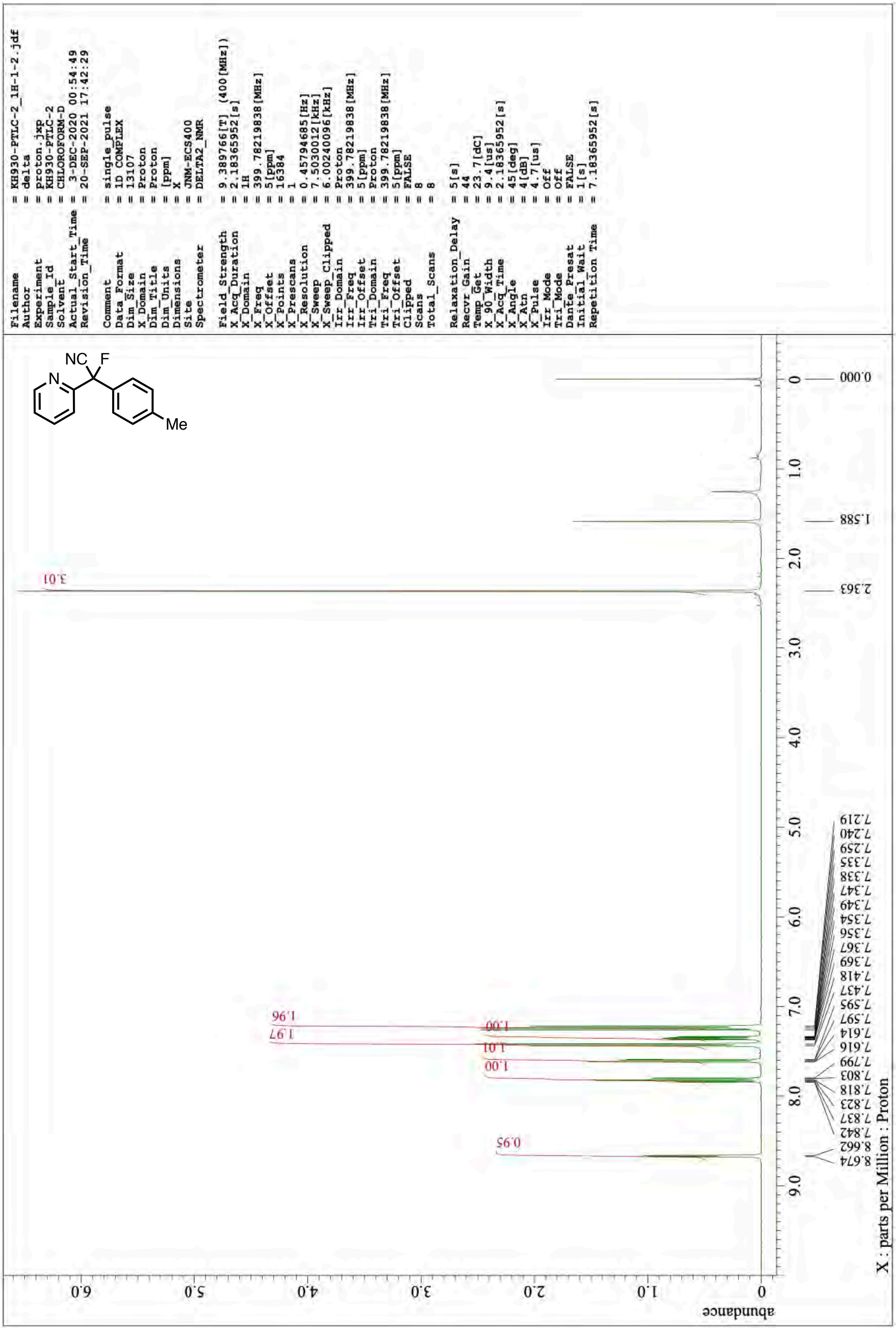

^{13}C NMR of **2B** (101 MHz, $CDCl_3$)

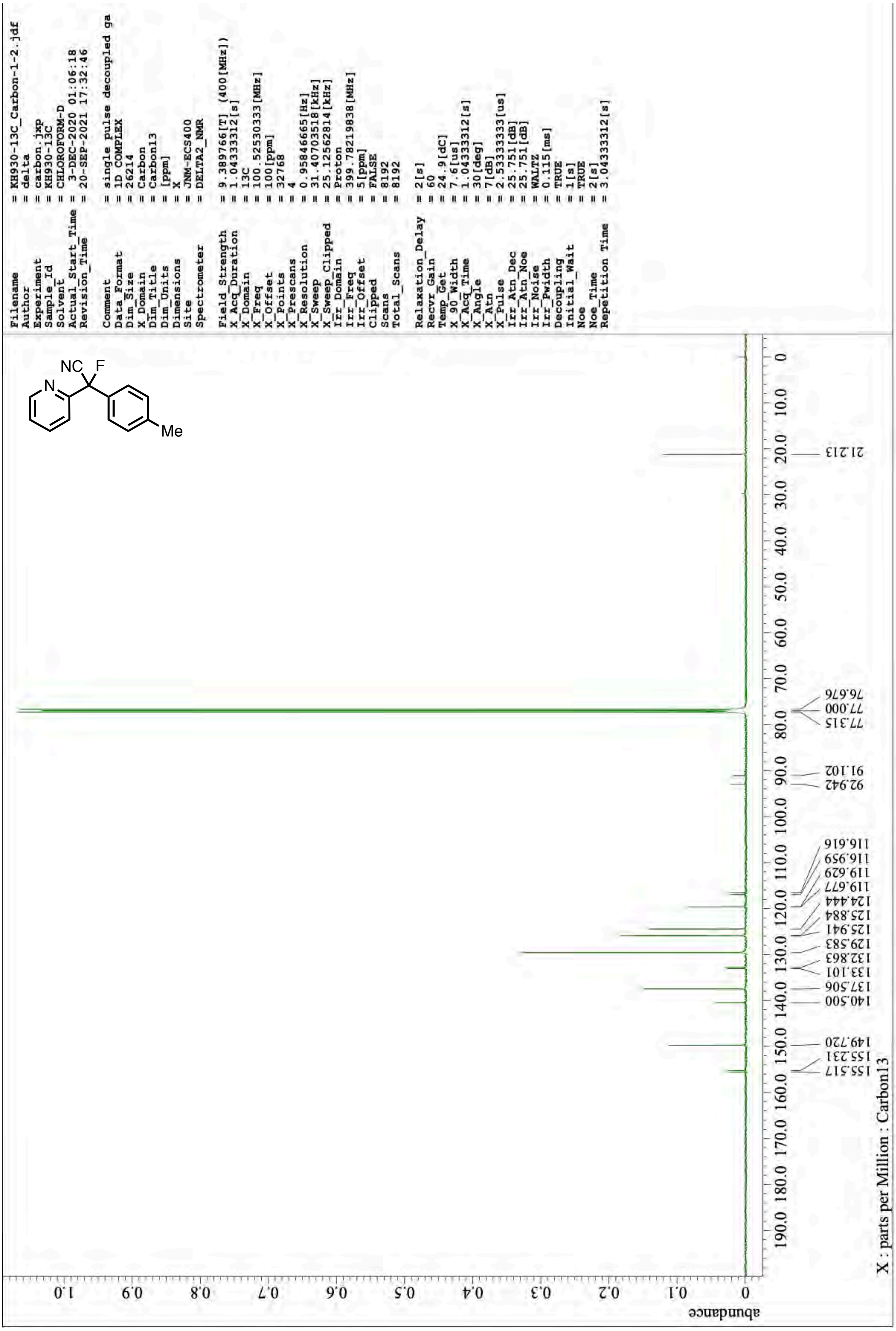

^{19}F NMR of **2B** (376 MHz, $CDCl_3$)

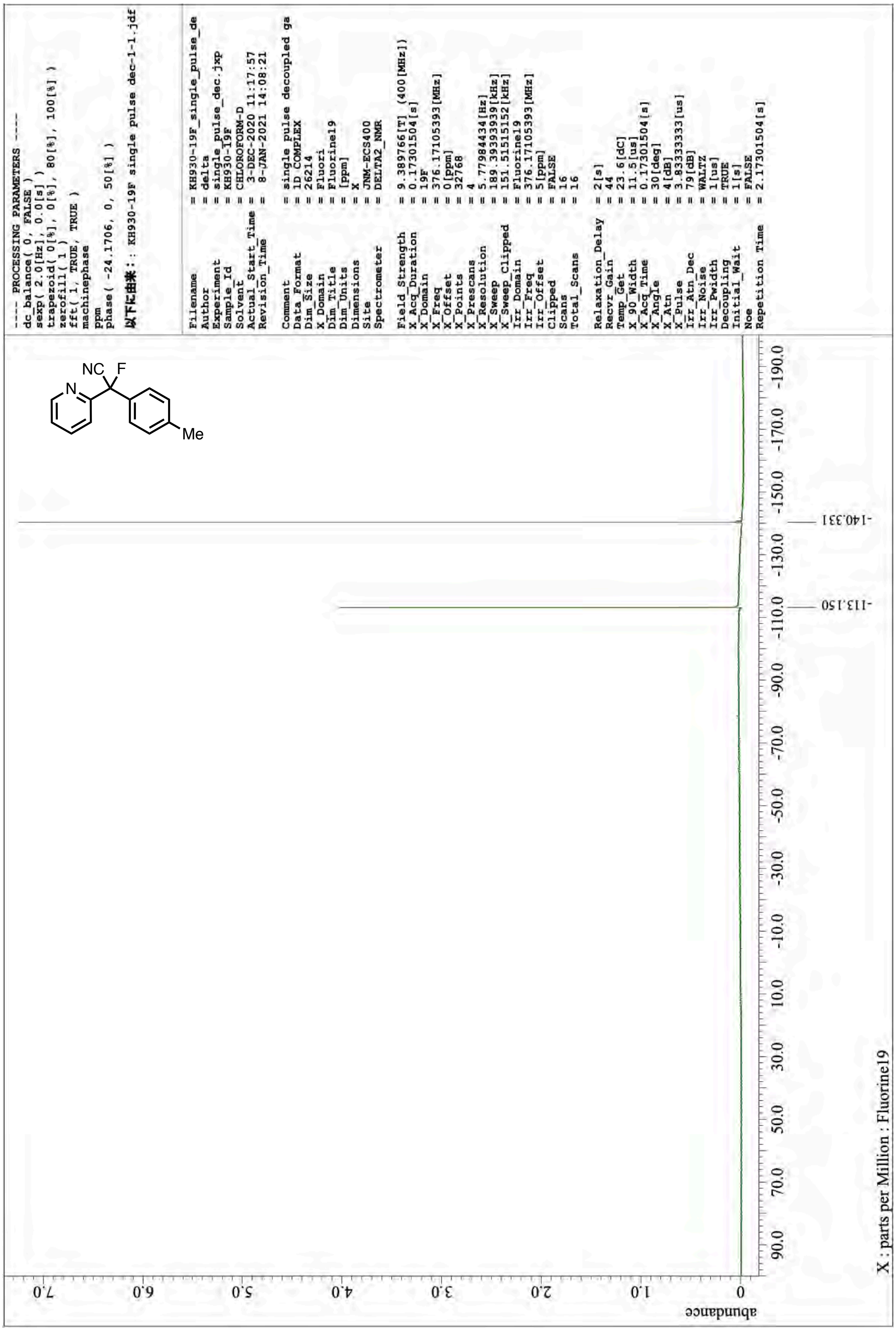

^{1}H NMR of **2C** (400 MHz, $CDCl_3$)

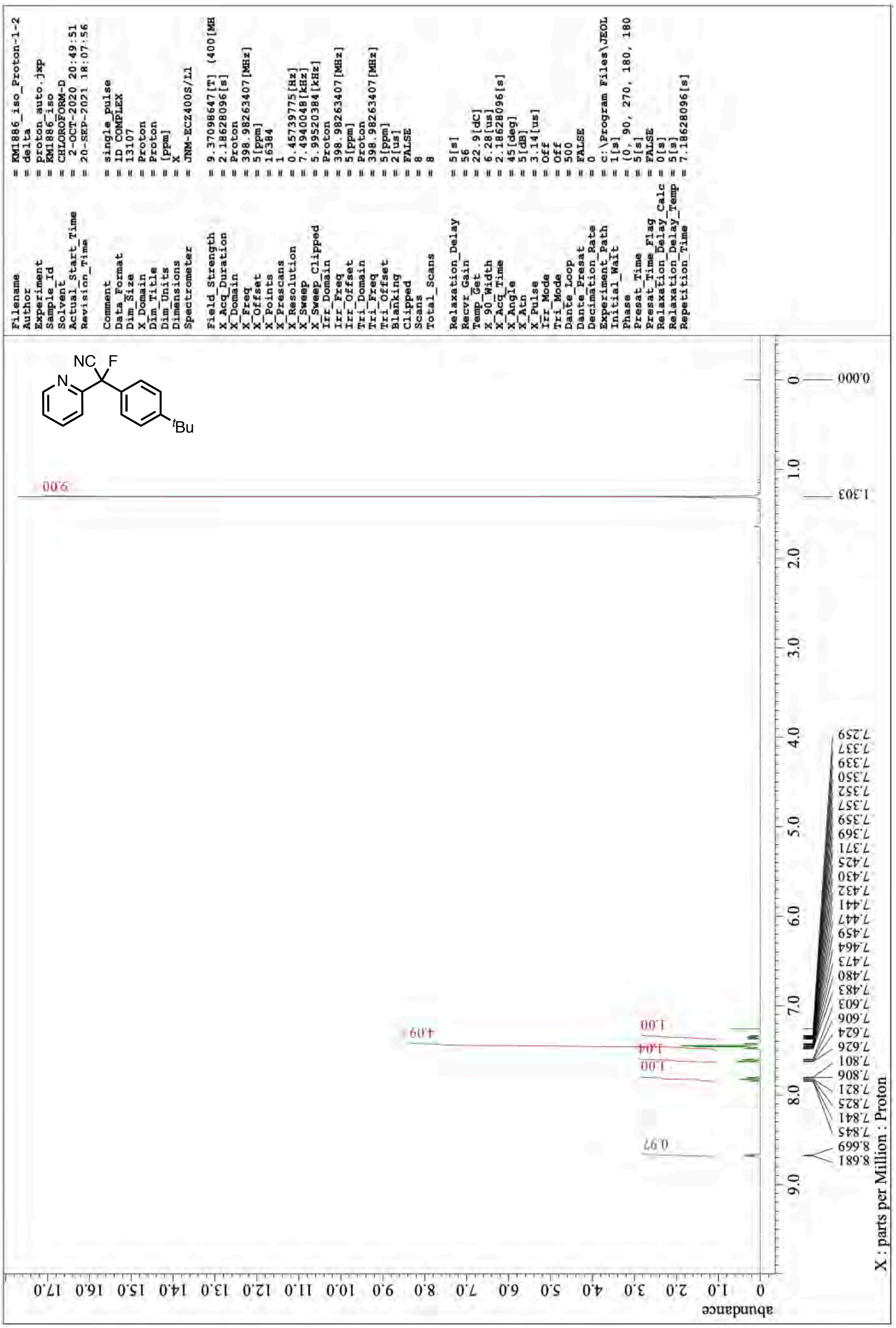

^{13}C NMR of **2C** (101 MHz, $CDCl_3$)

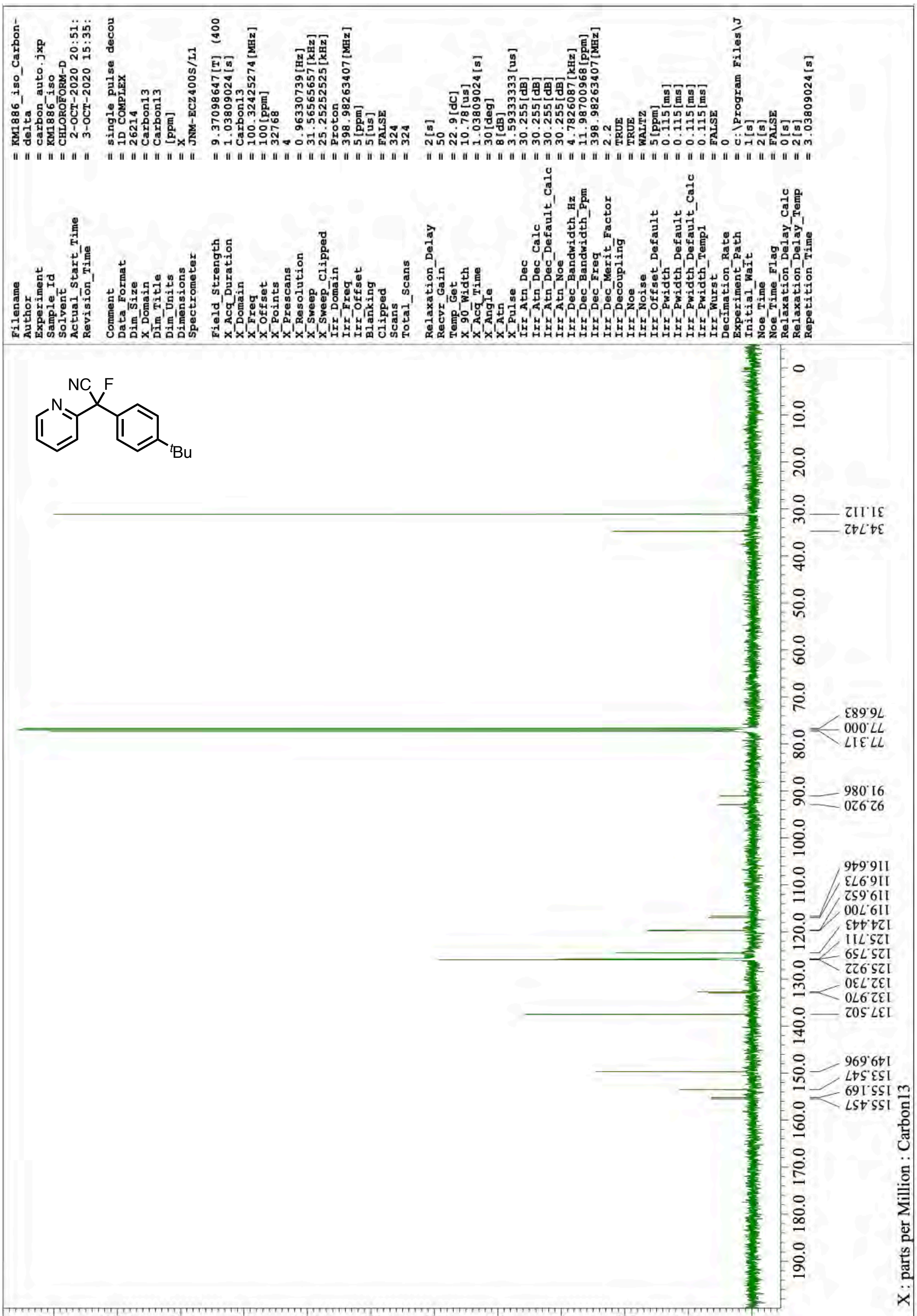

^{19}F NMR of **2C** (376 MHz, $CDCl_3$)

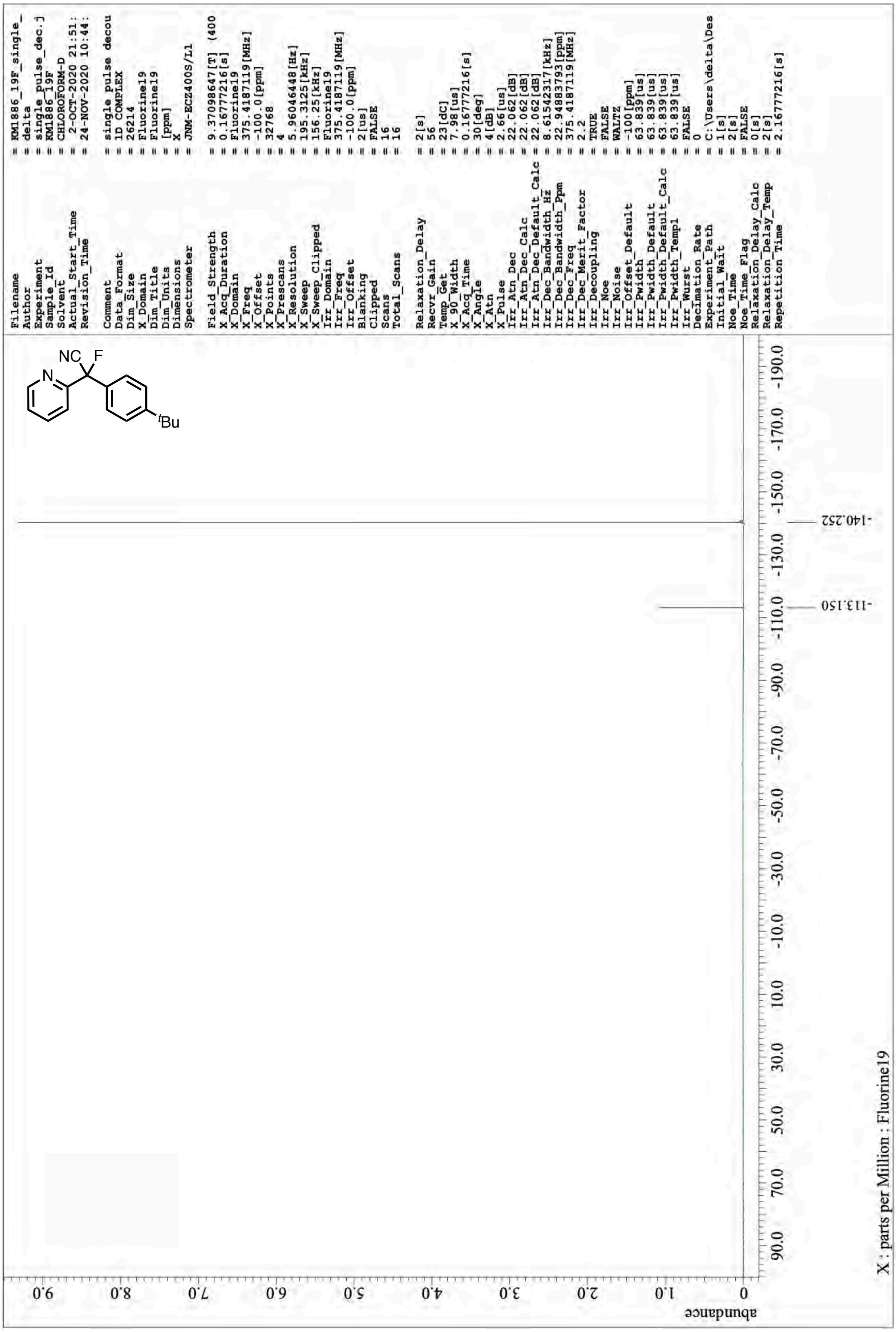

^{1}H NMR of **2D** (400 MHz, $CDCl_3$)

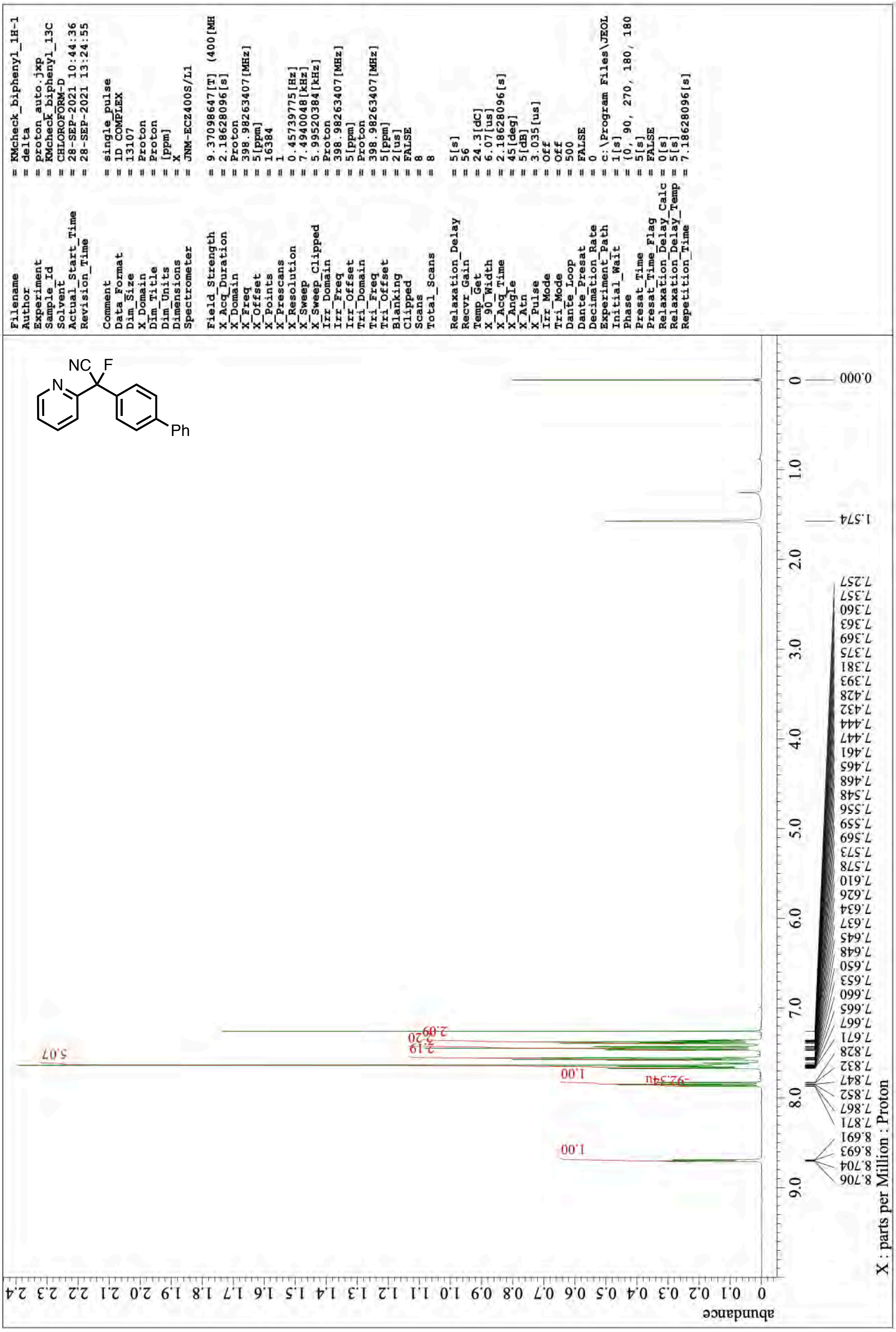

^{13}C NMR of **2D** (101 MHz, $CDCl_3$)

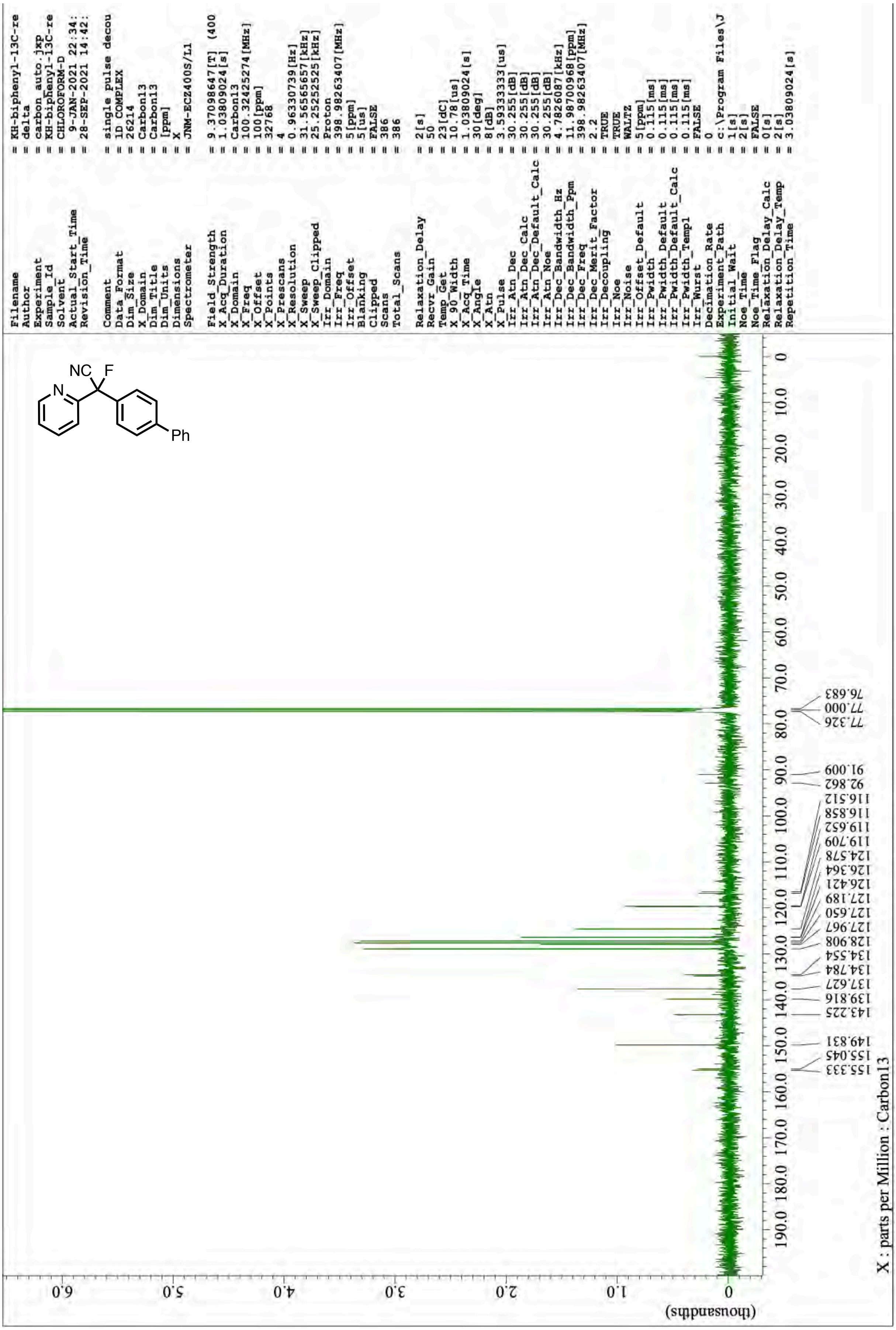

^{19}F NMR of **2D** (376 MHz, $CDCl_3$)

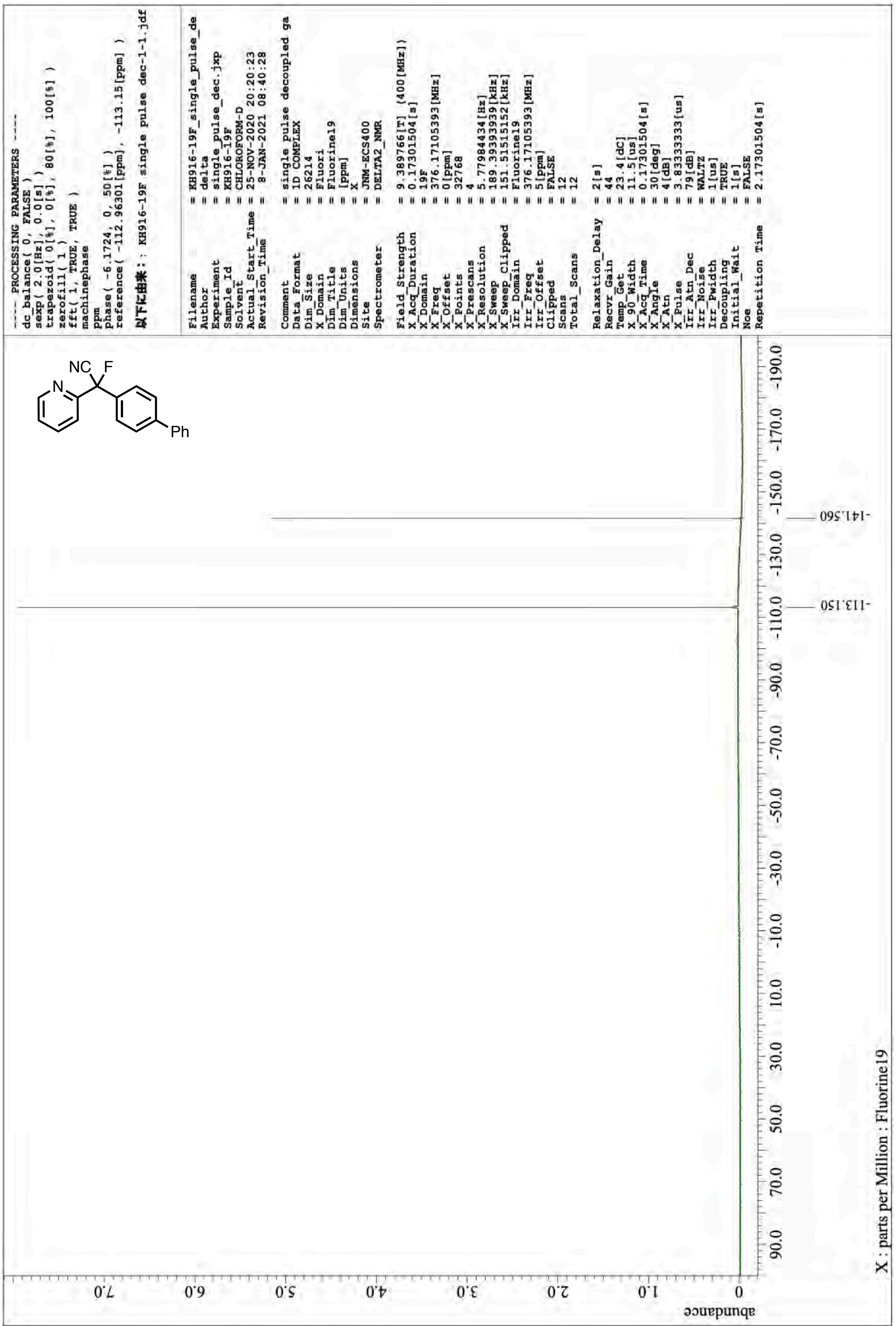

^{1}H NMR of **2E** (400 MHz, $CDCl_3$)

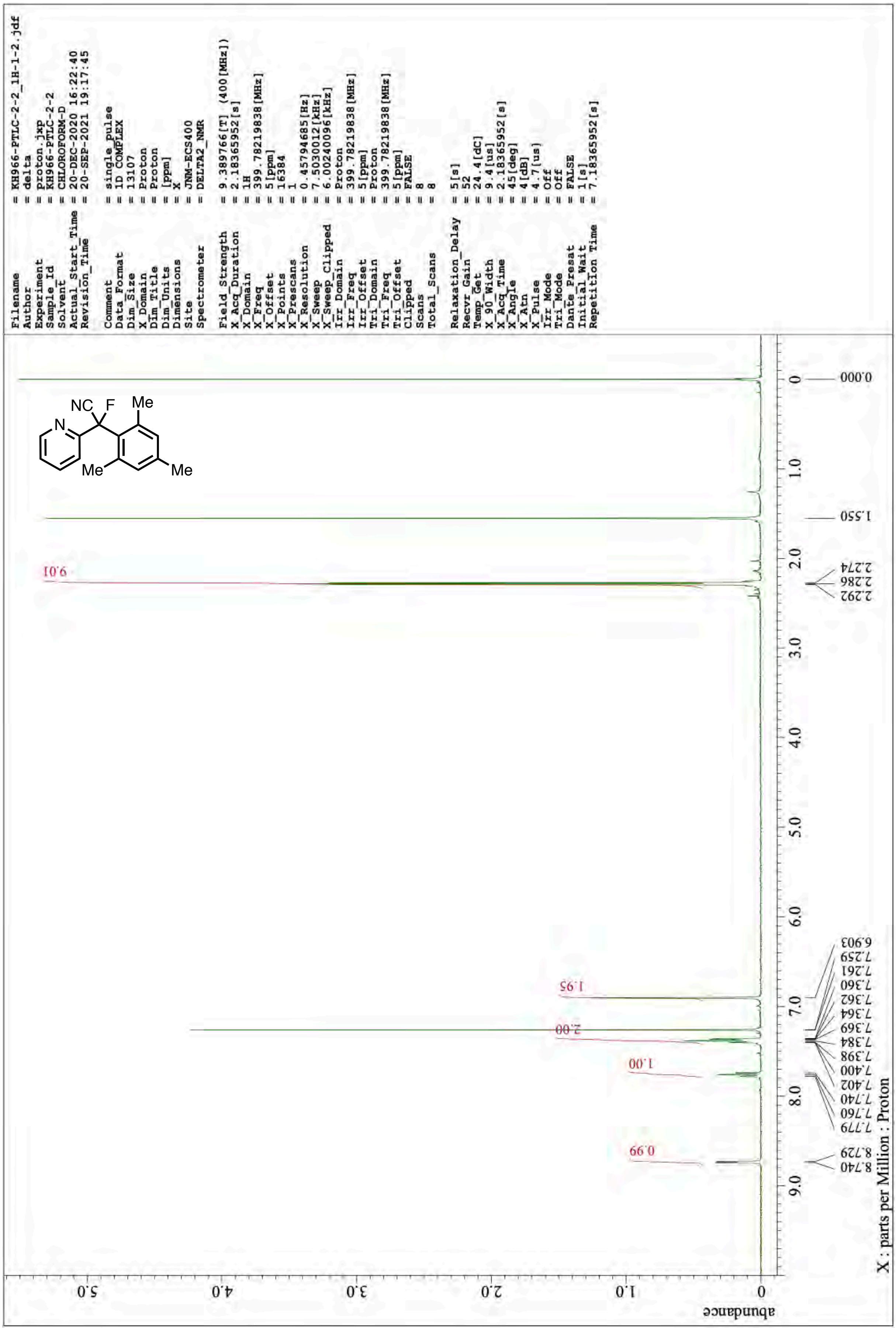

^{13}C NMR of **2E** (101 MHz, $CDCl_3$)

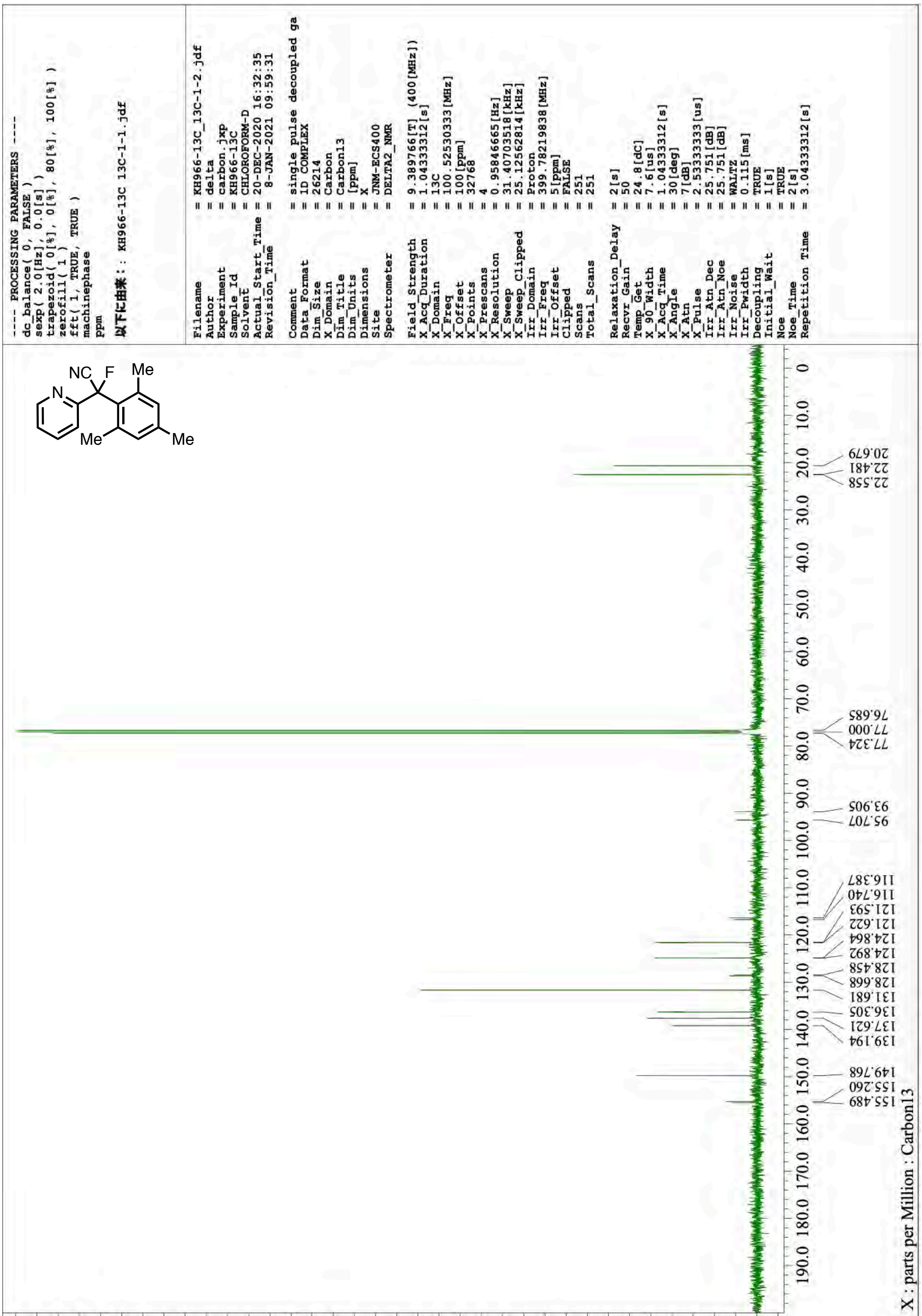

^{19}F NMR of **2E** (376 MHz, $CDCl_3$)

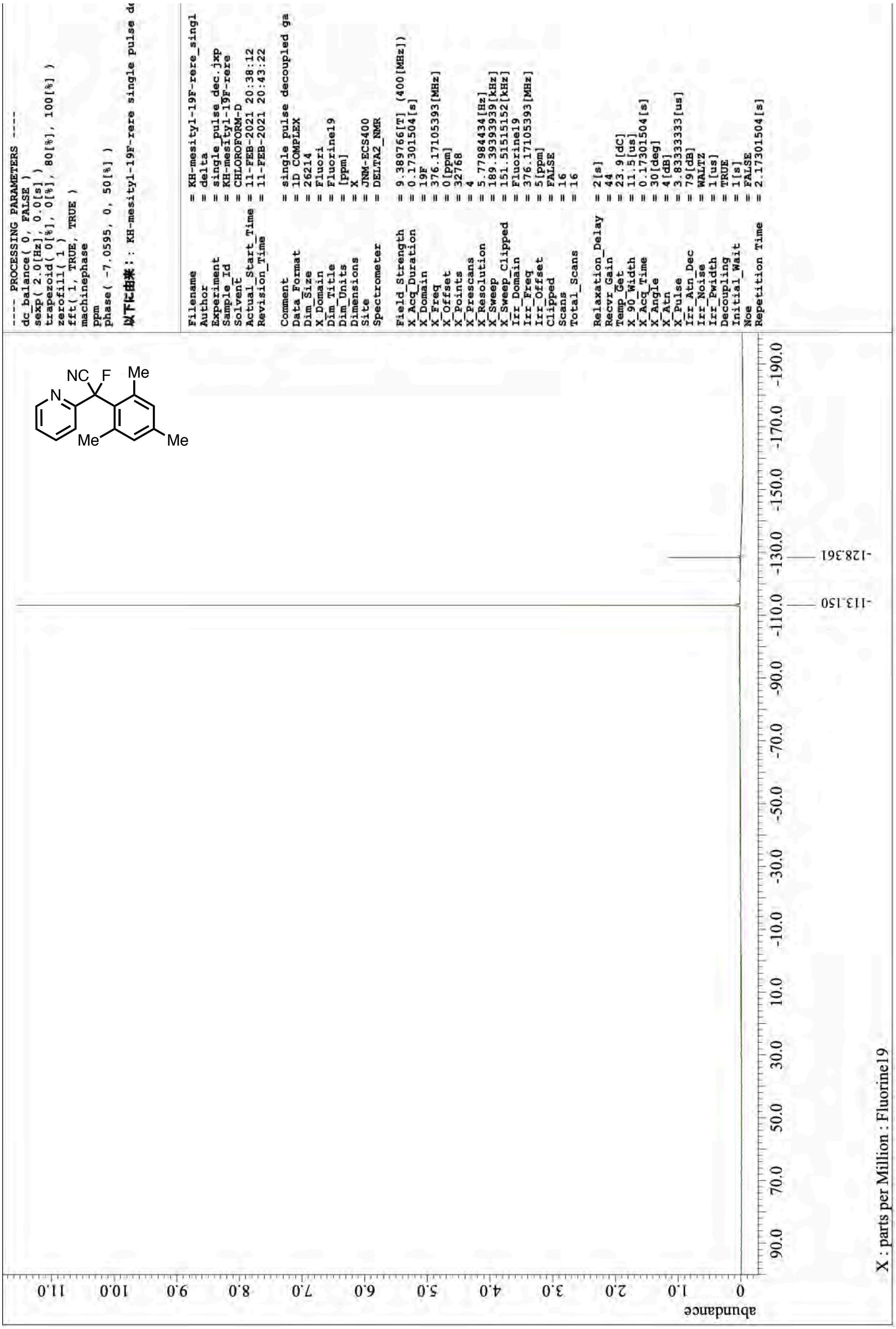

^{1}H NMR of **2F** (400 MHz, $CDCl_3$)

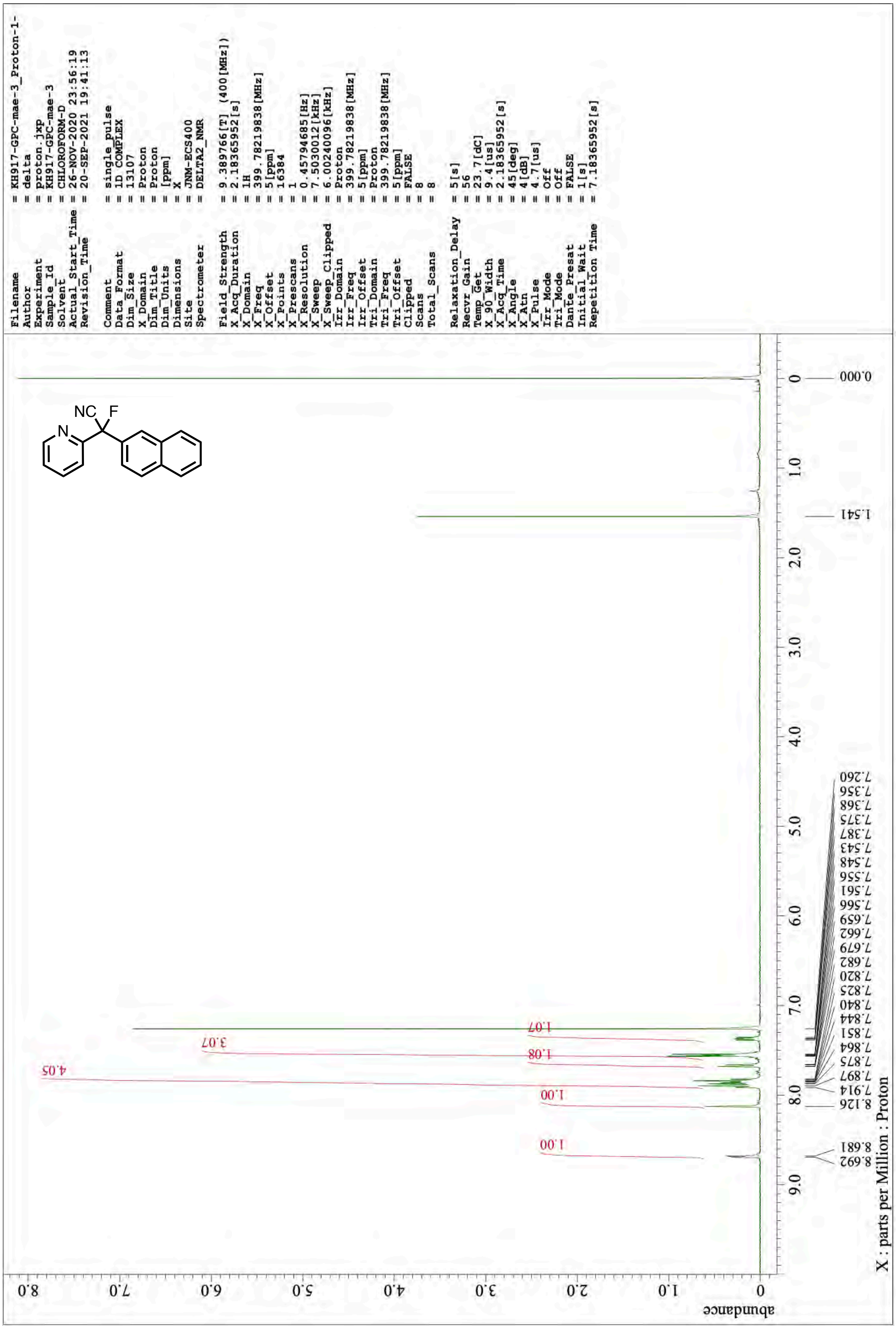

^{13}C NMR of **2F** (101 MHz, $CDCl_3$)

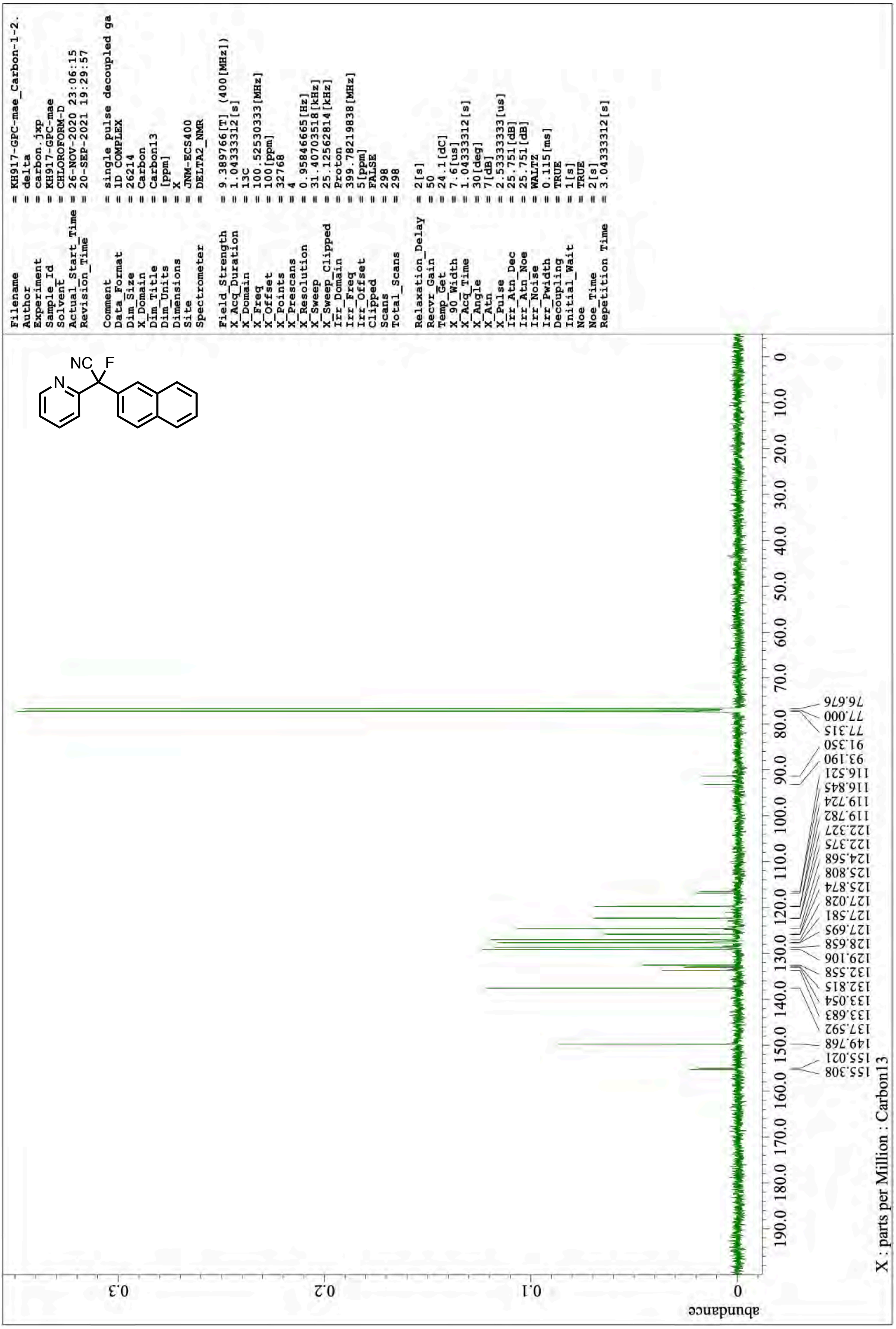

^{19}F NMR of **2F** (376 MHz, $CDCl_3$)

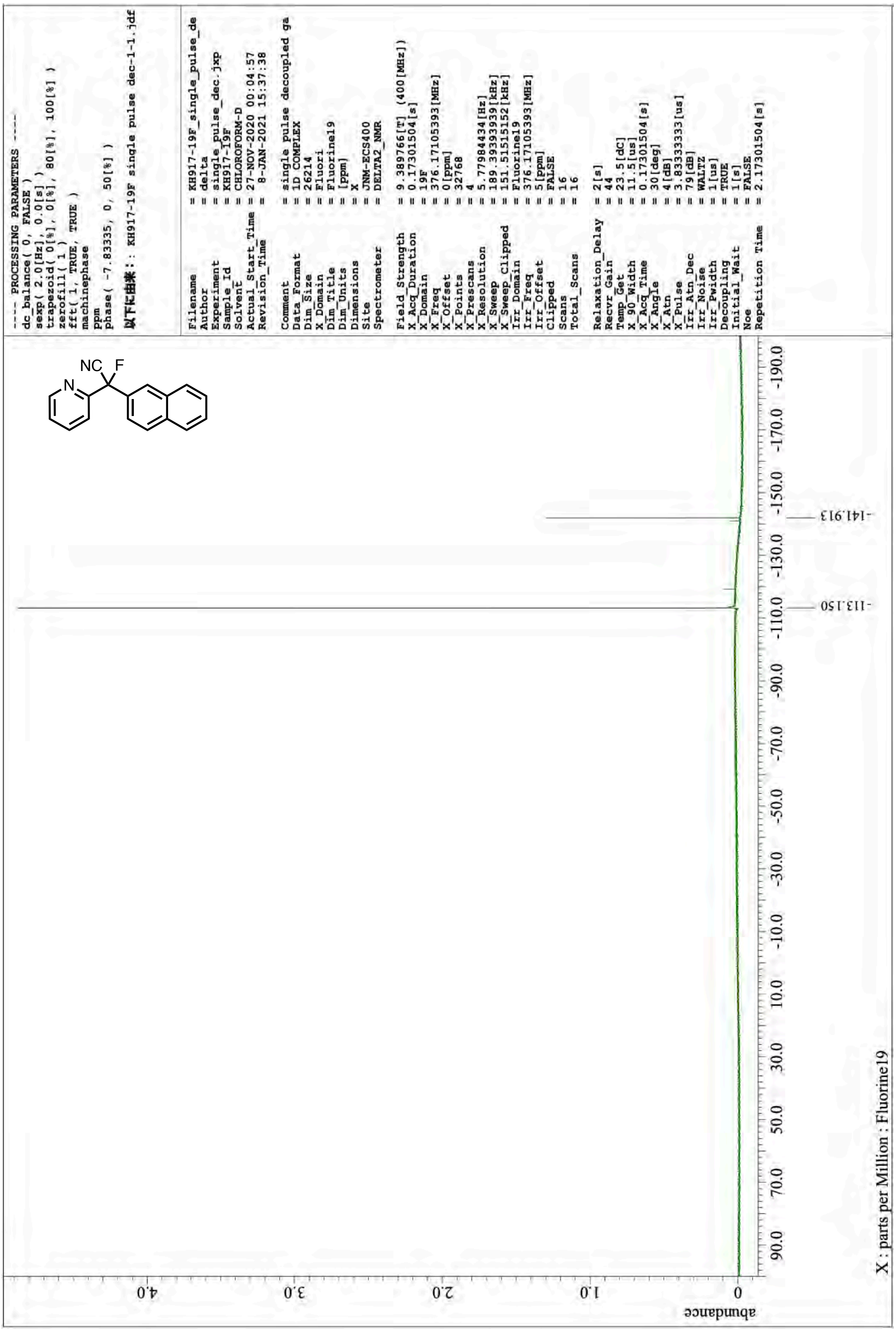

^{1}H NMR of **2G** (400 MHz, $CDCl_3$)

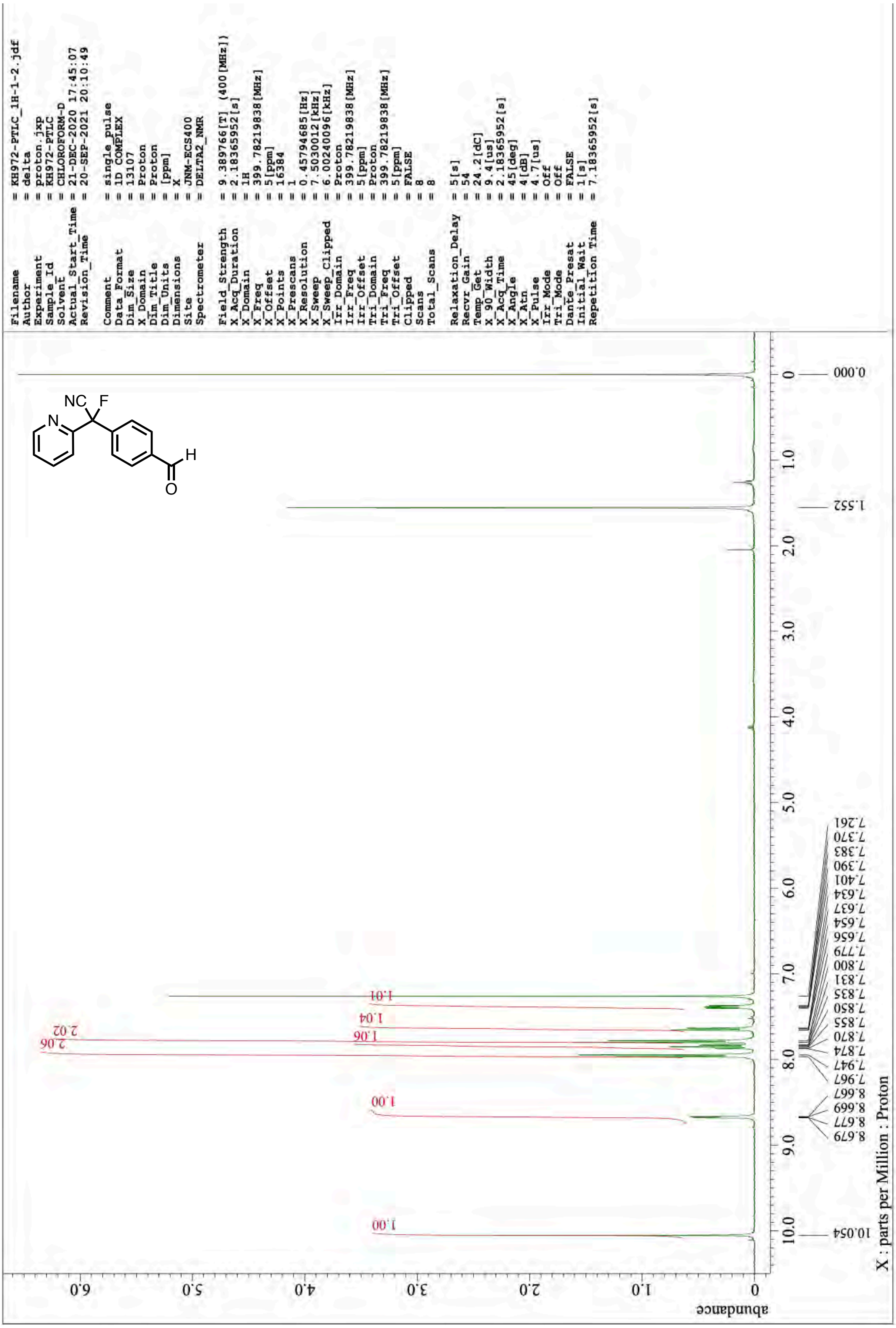

^{13}C NMR of **2G** (101 MHz, $CDCl_3$)

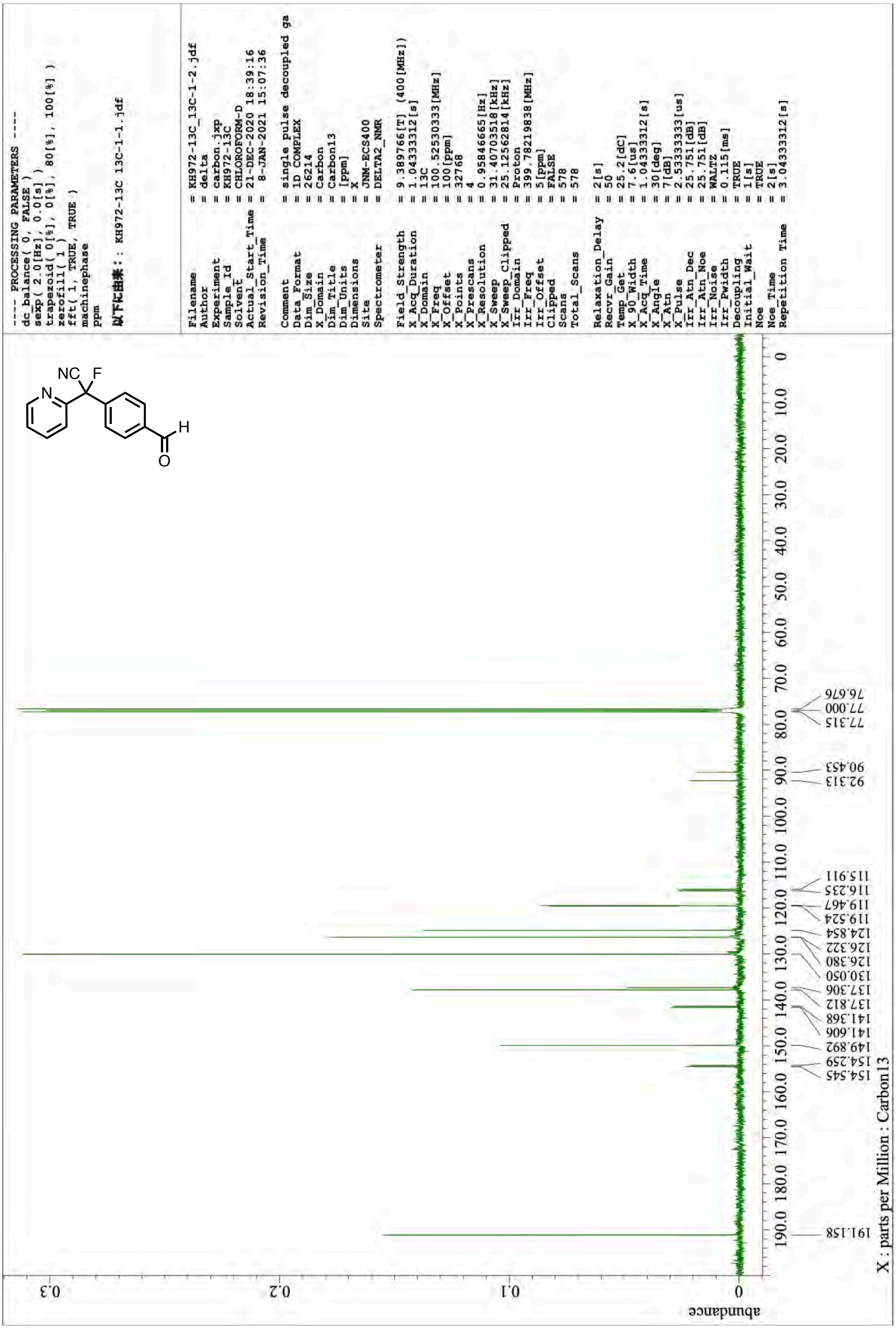

^{19}F NMR of **2G** (376 MHz, $CDCl_3$)

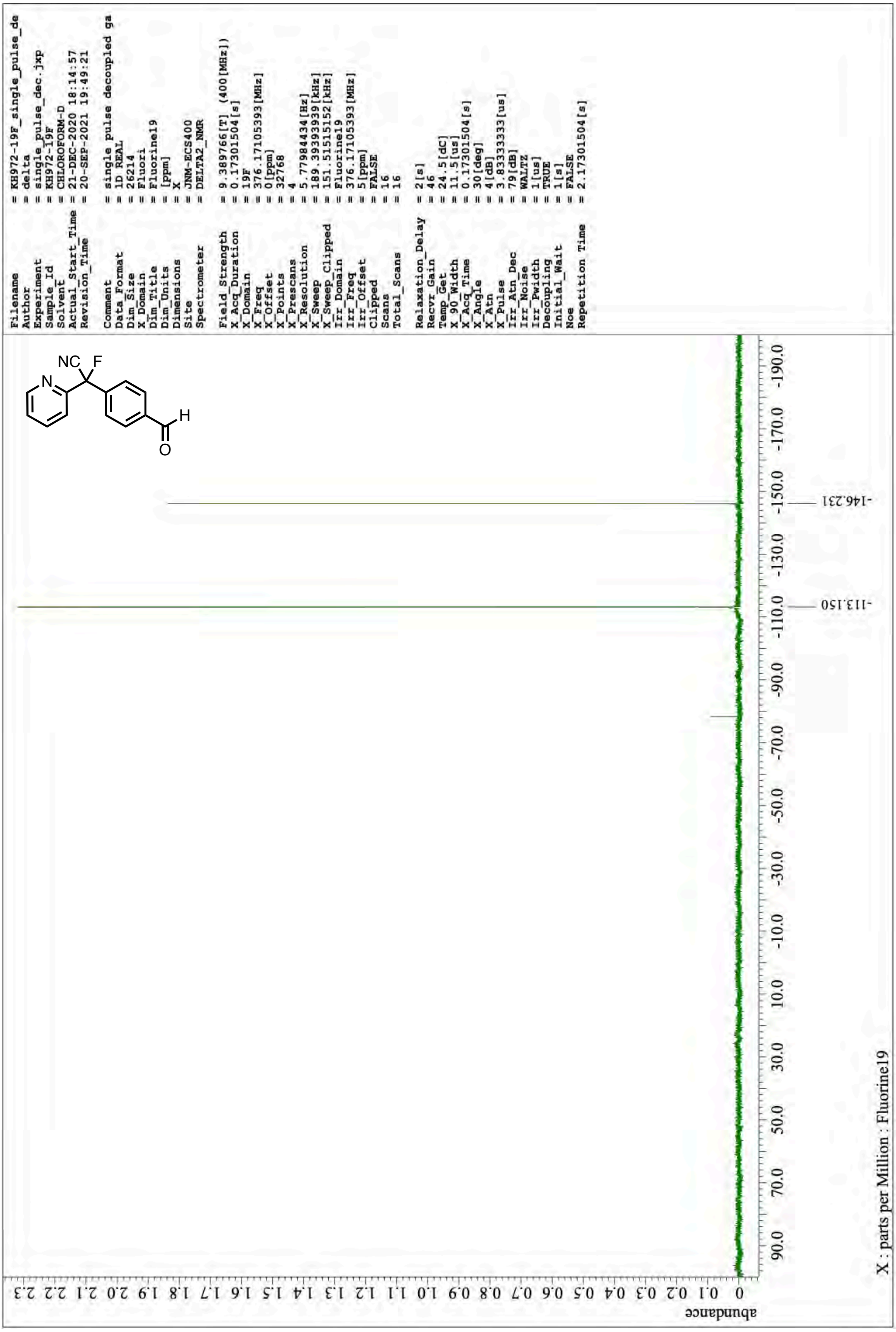

^{1}H NMR of **2H** (400 MHz, $CDCl_3$)

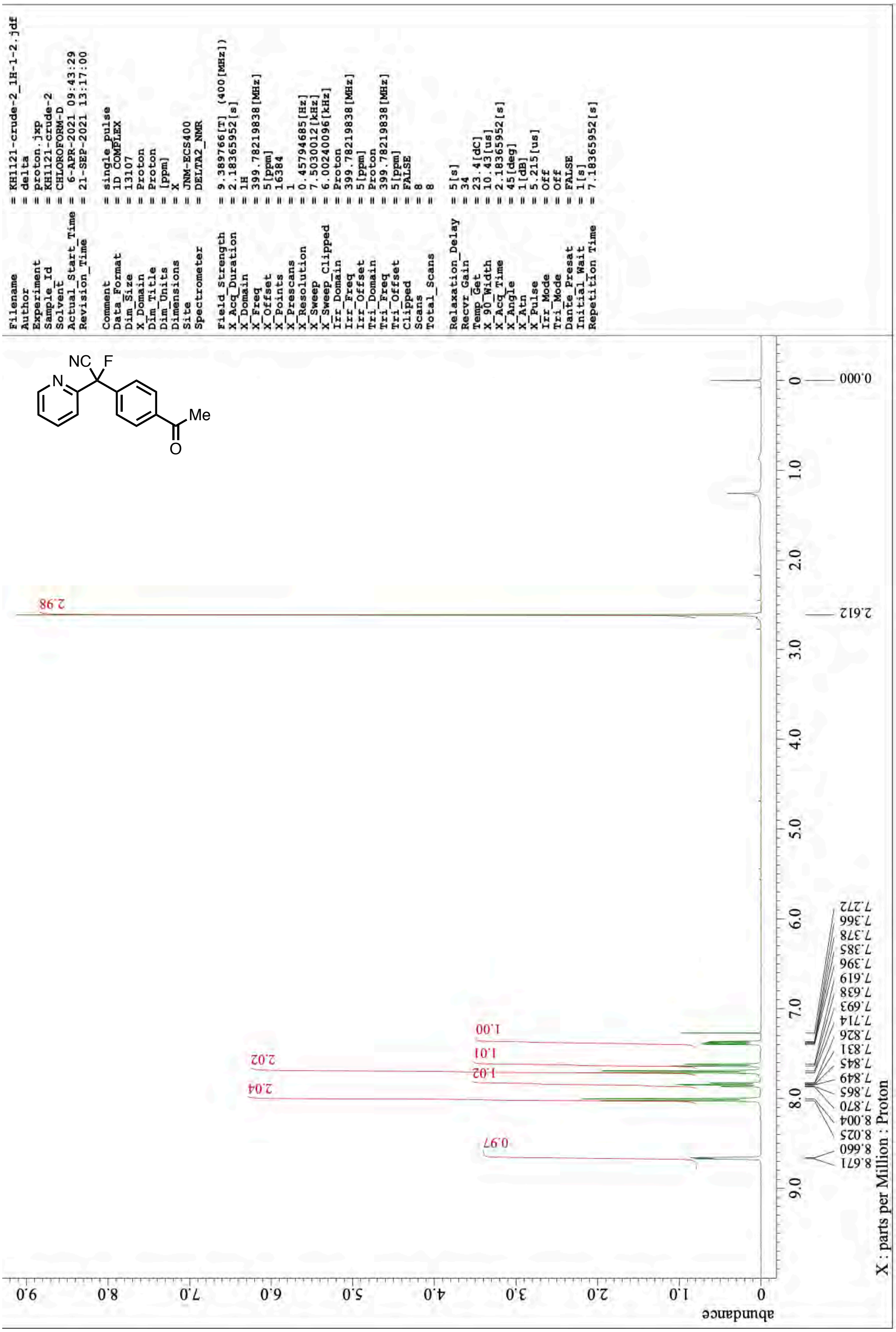

^{13}C NMR of **2H** (101 MHz, $CDCl_3$)

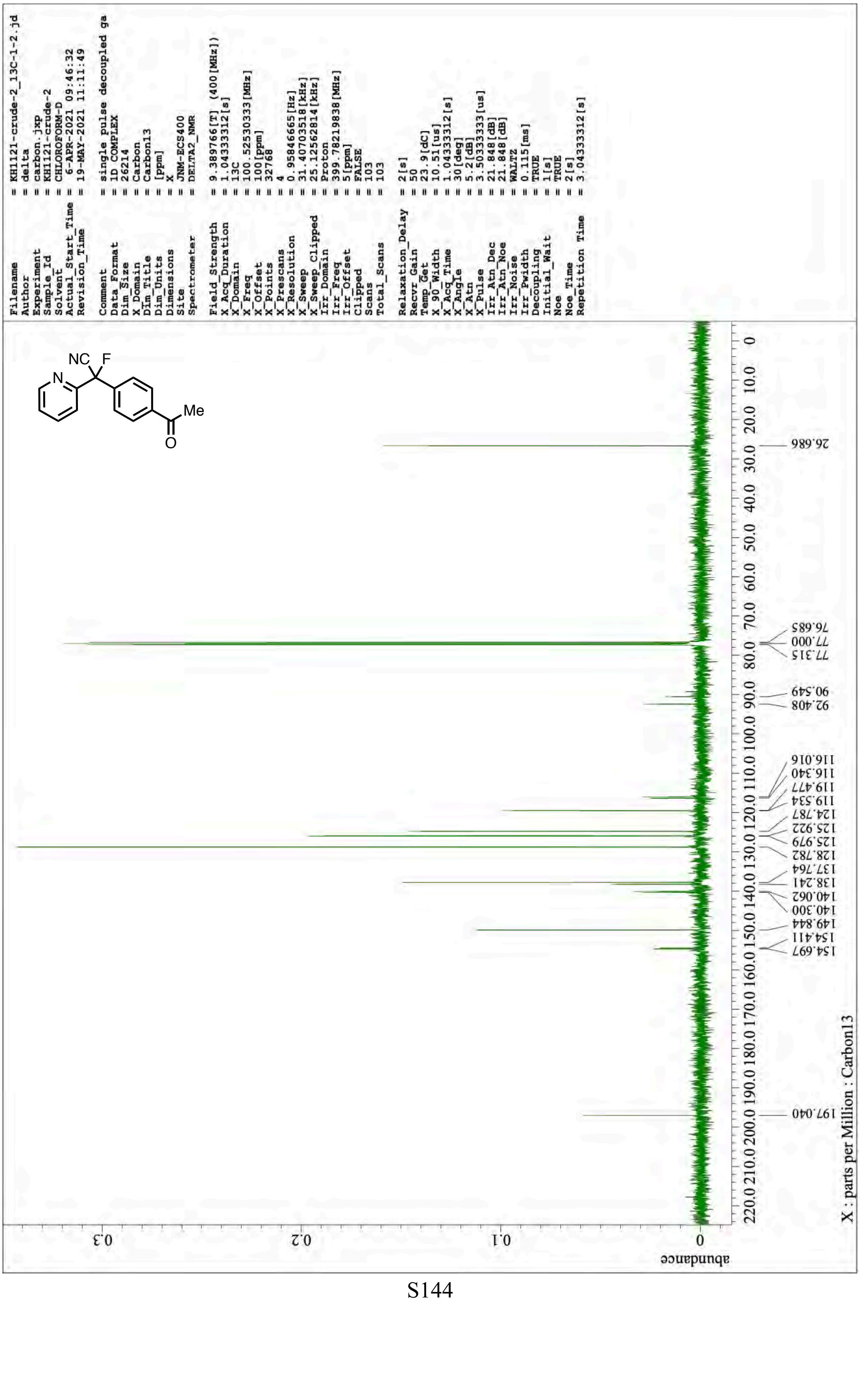

^{19}F NMR of **2H** (376 MHz, $CDCl_3$)

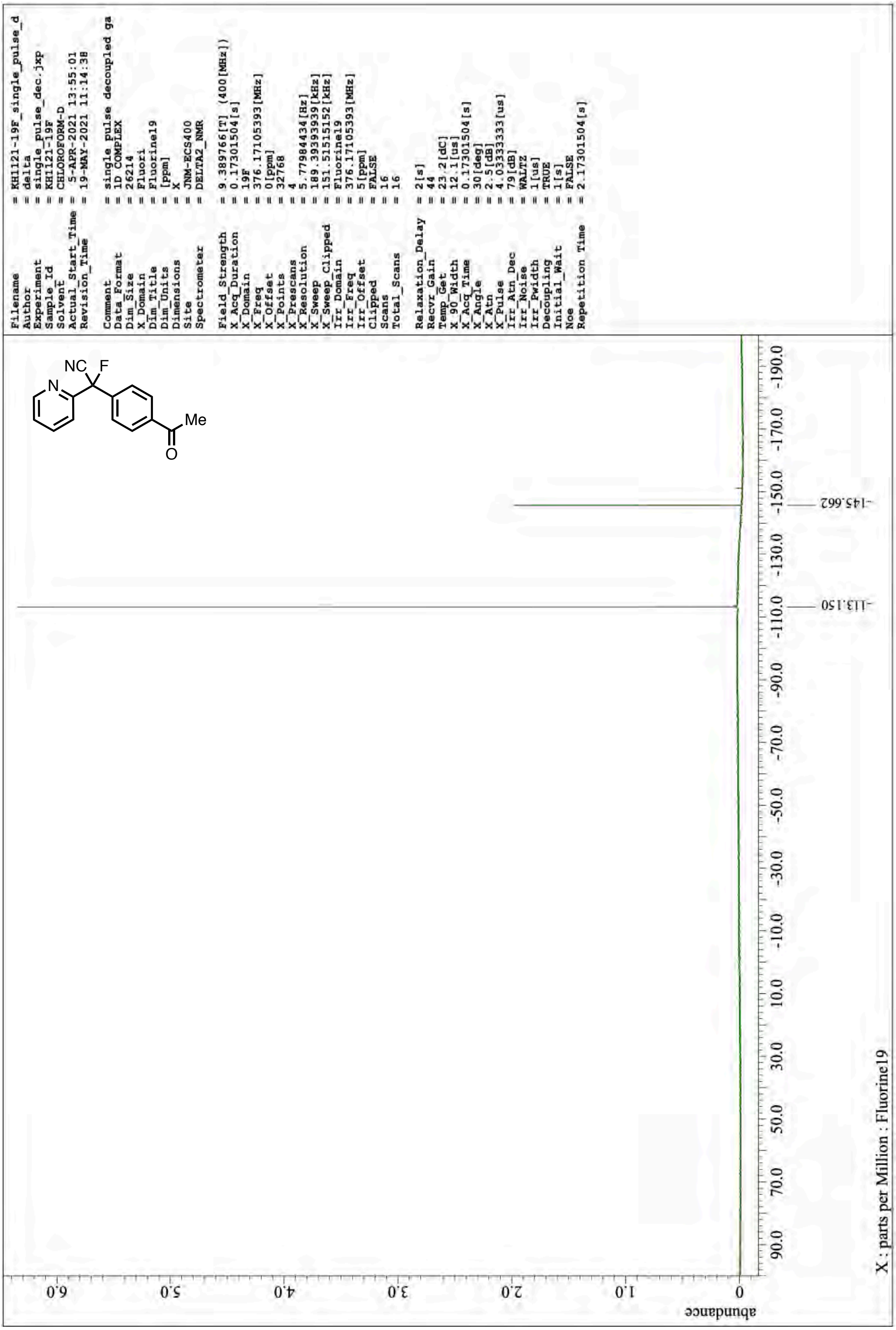

^{1}H NMR of **2l** (400 MHz, $CDCl_3$)

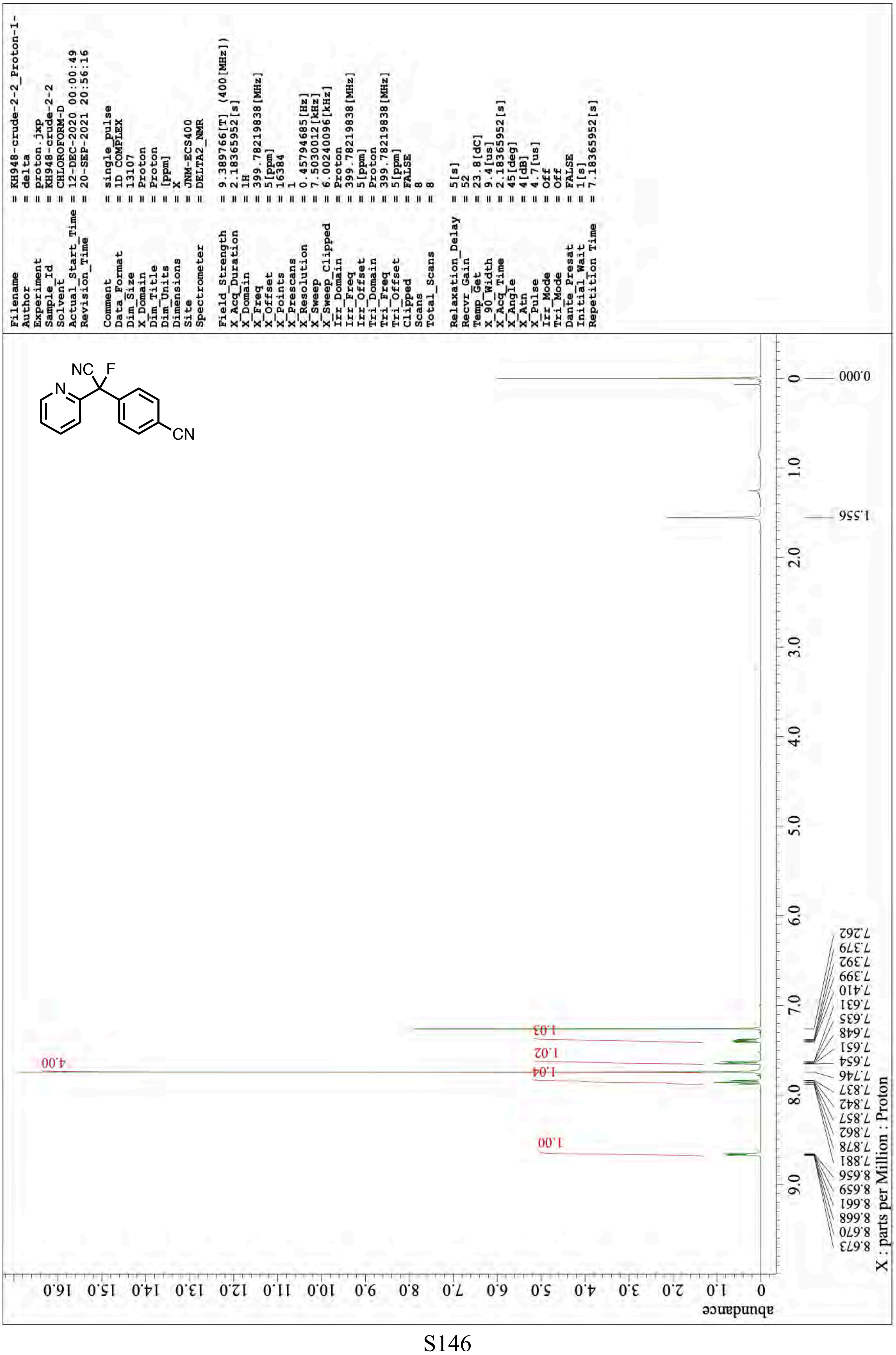

^{13}C NMR of **2l** (101 MHz, $CDCl_3$)

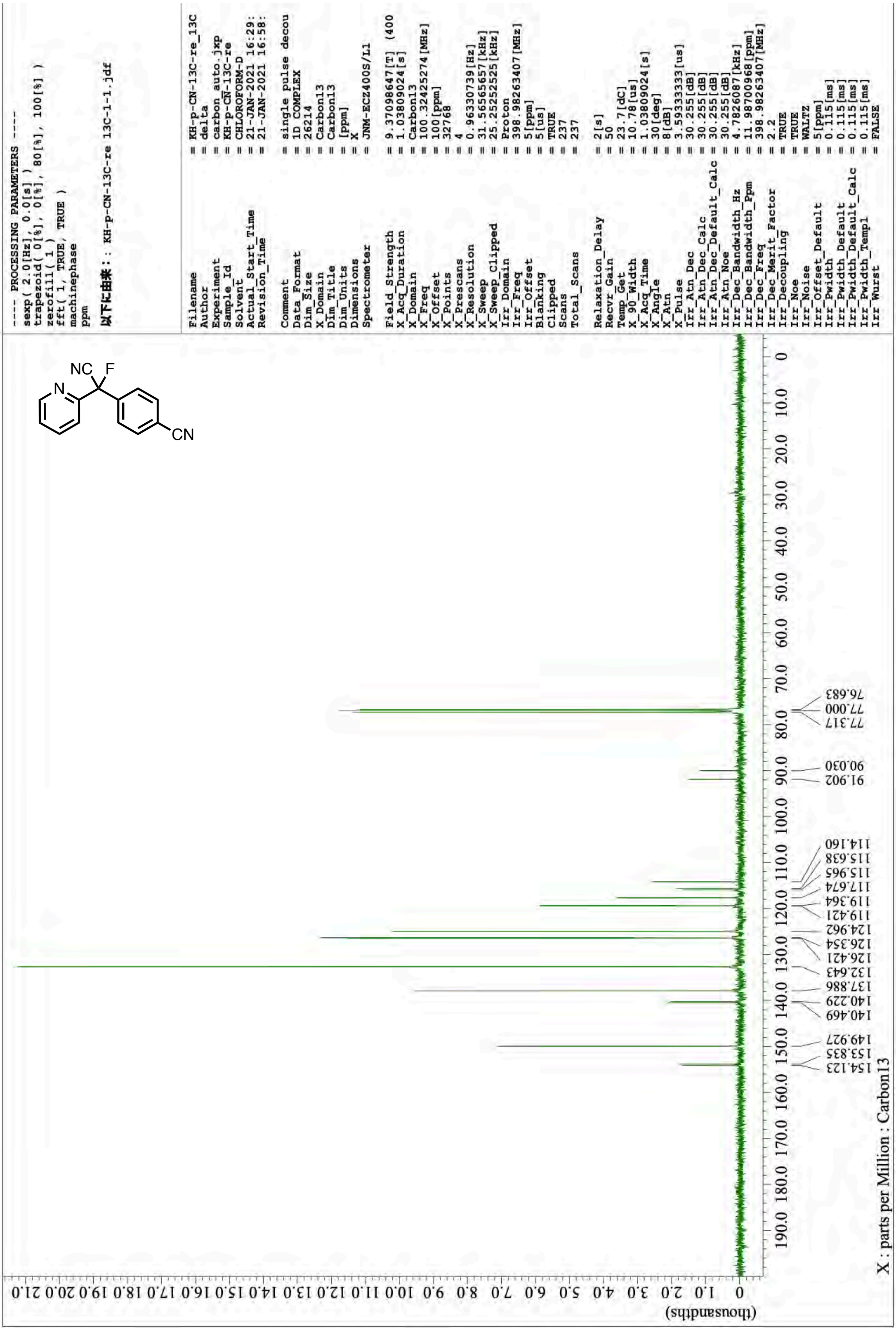

^{19}F NMR of **2l** (376 MHz, $CDCl_3$)

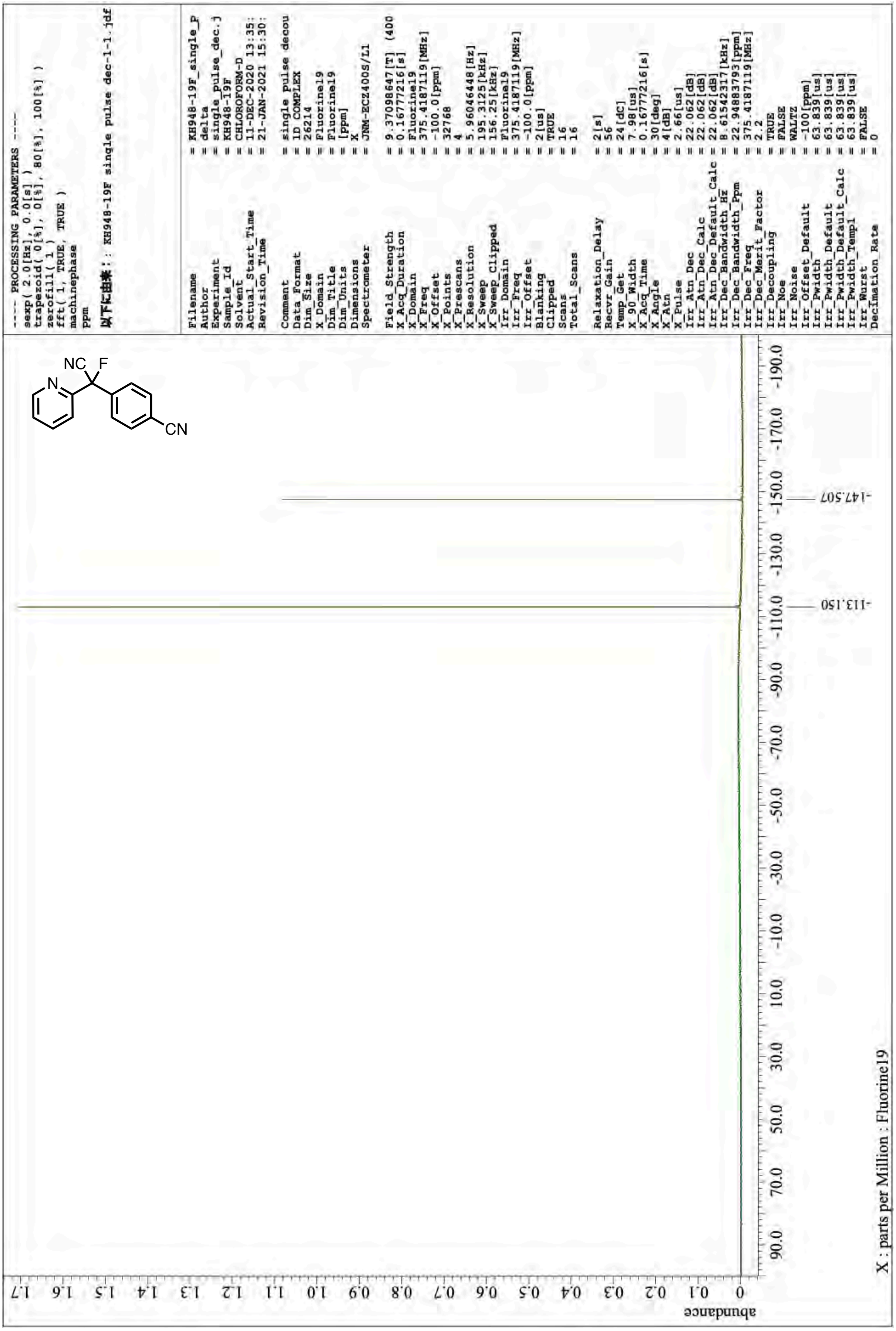

^{1}H NMR of **2J** (400 MHz, $CDCl_3$)

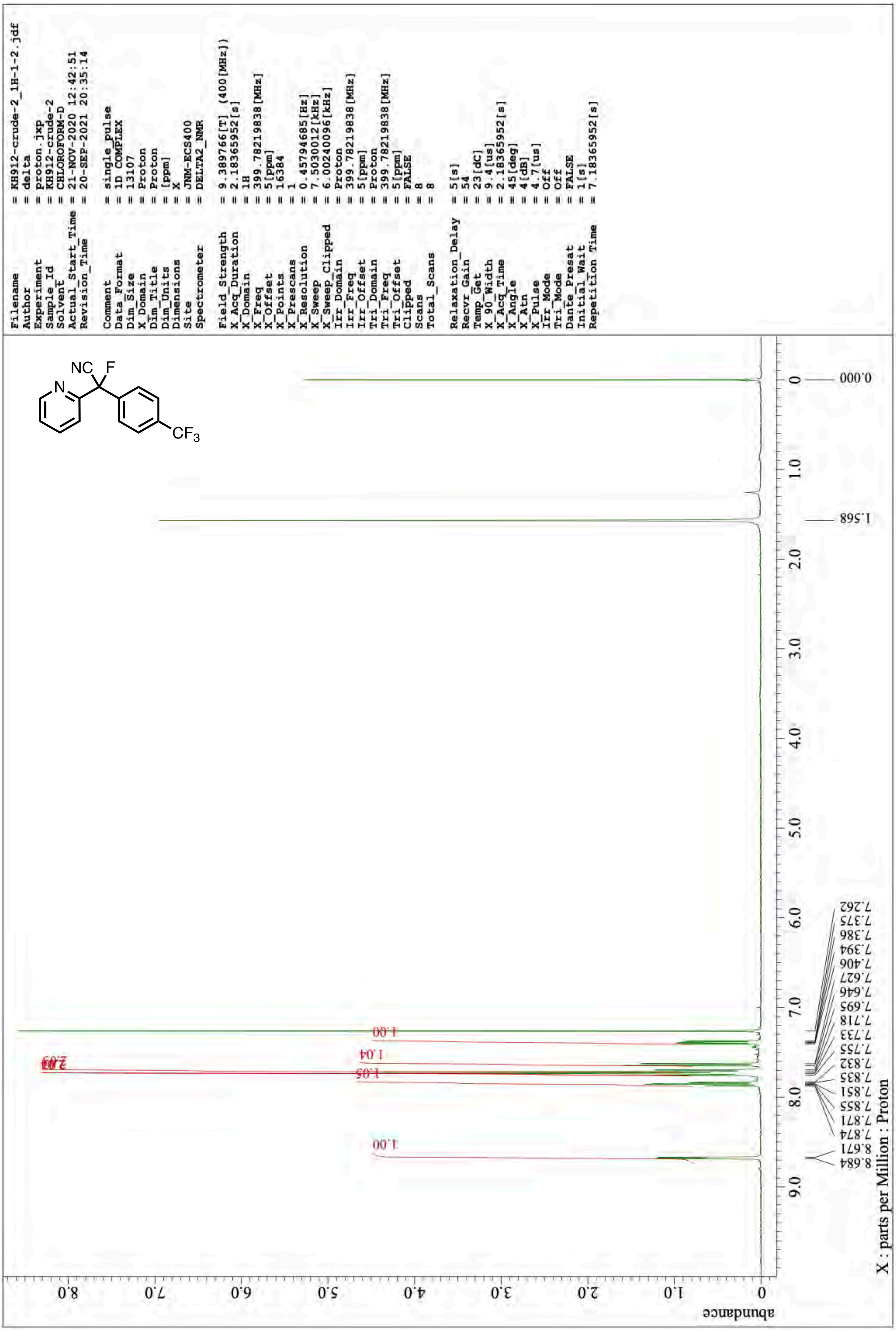

^{13}C NMR of **2J** (101 MHz, $CDCl_3$)

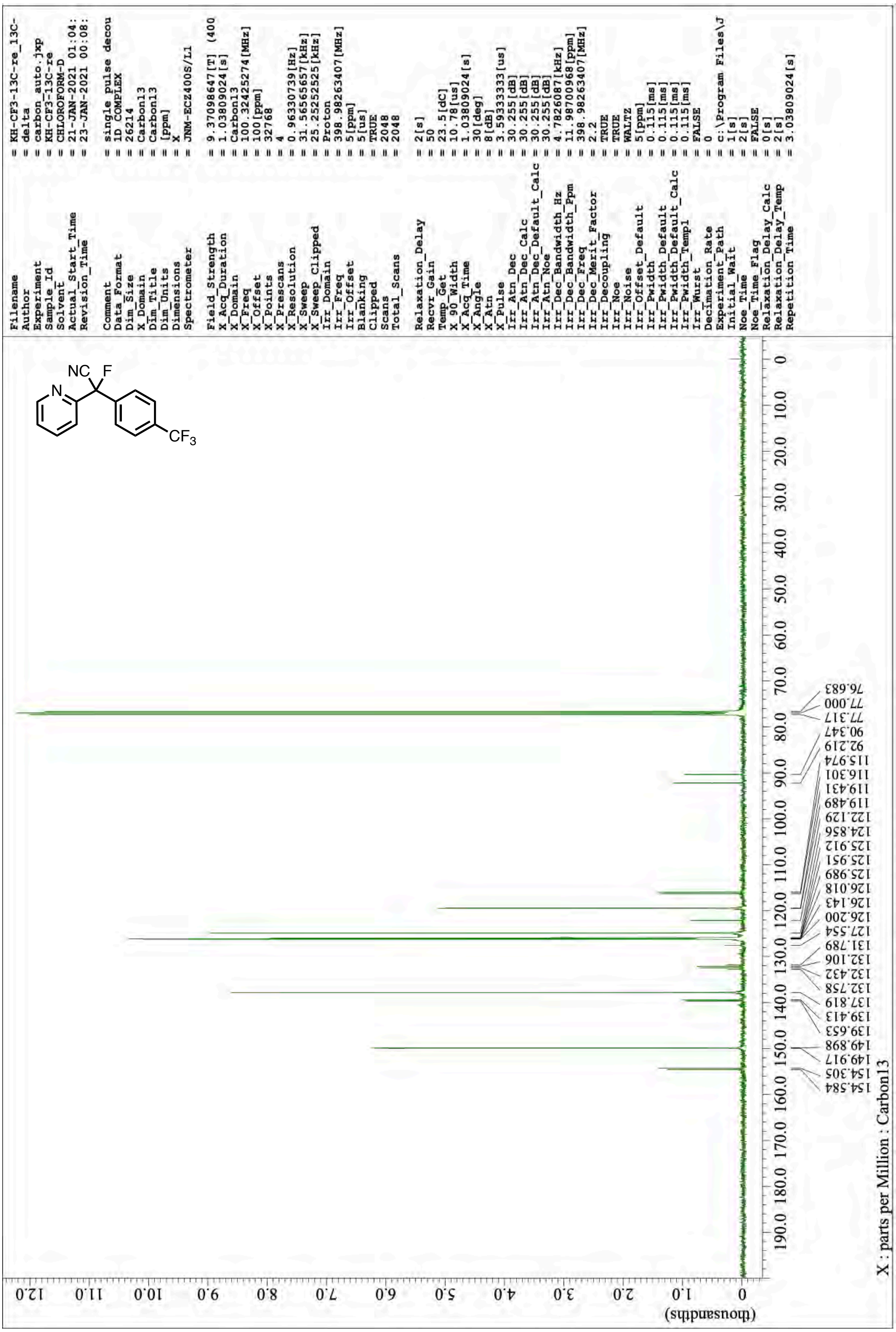

^{19}F NMR of **2J** (376 MHz, $CDCl_3$)

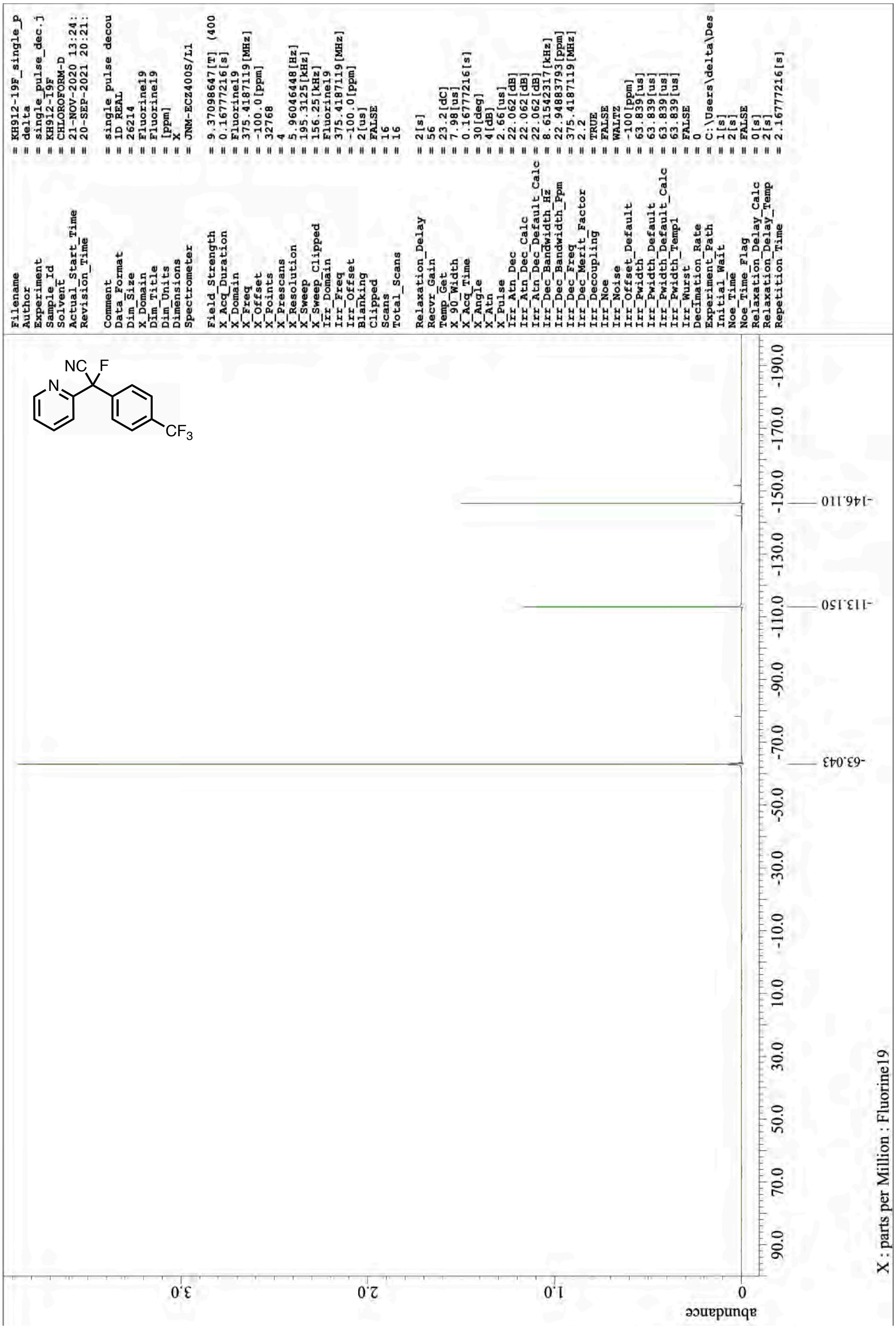

^{1}H NMR of **2K** (400 MHz, $CDCl_3$)

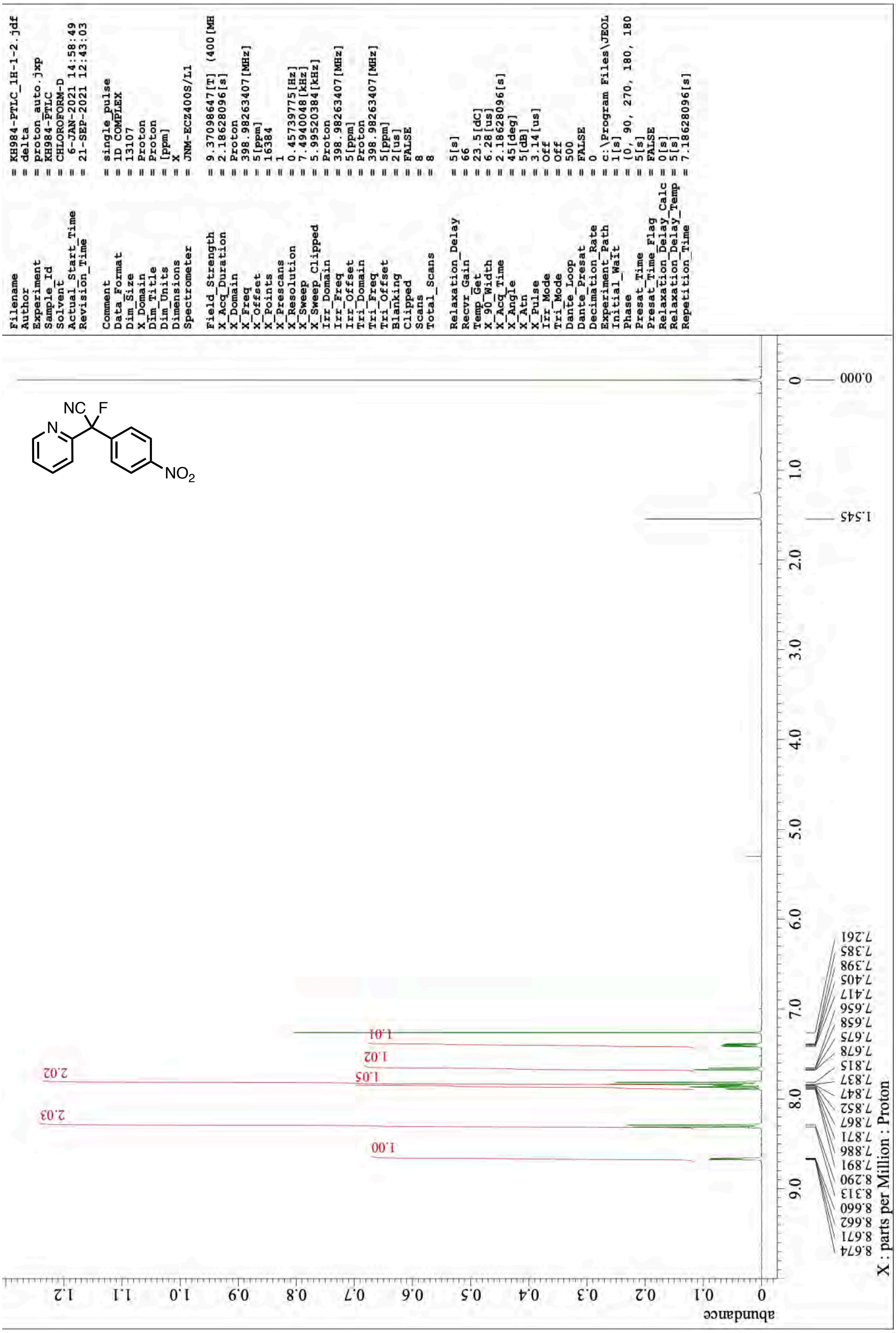

^{13}C NMR of **2K** (101 MHz, $CDCl_3$)

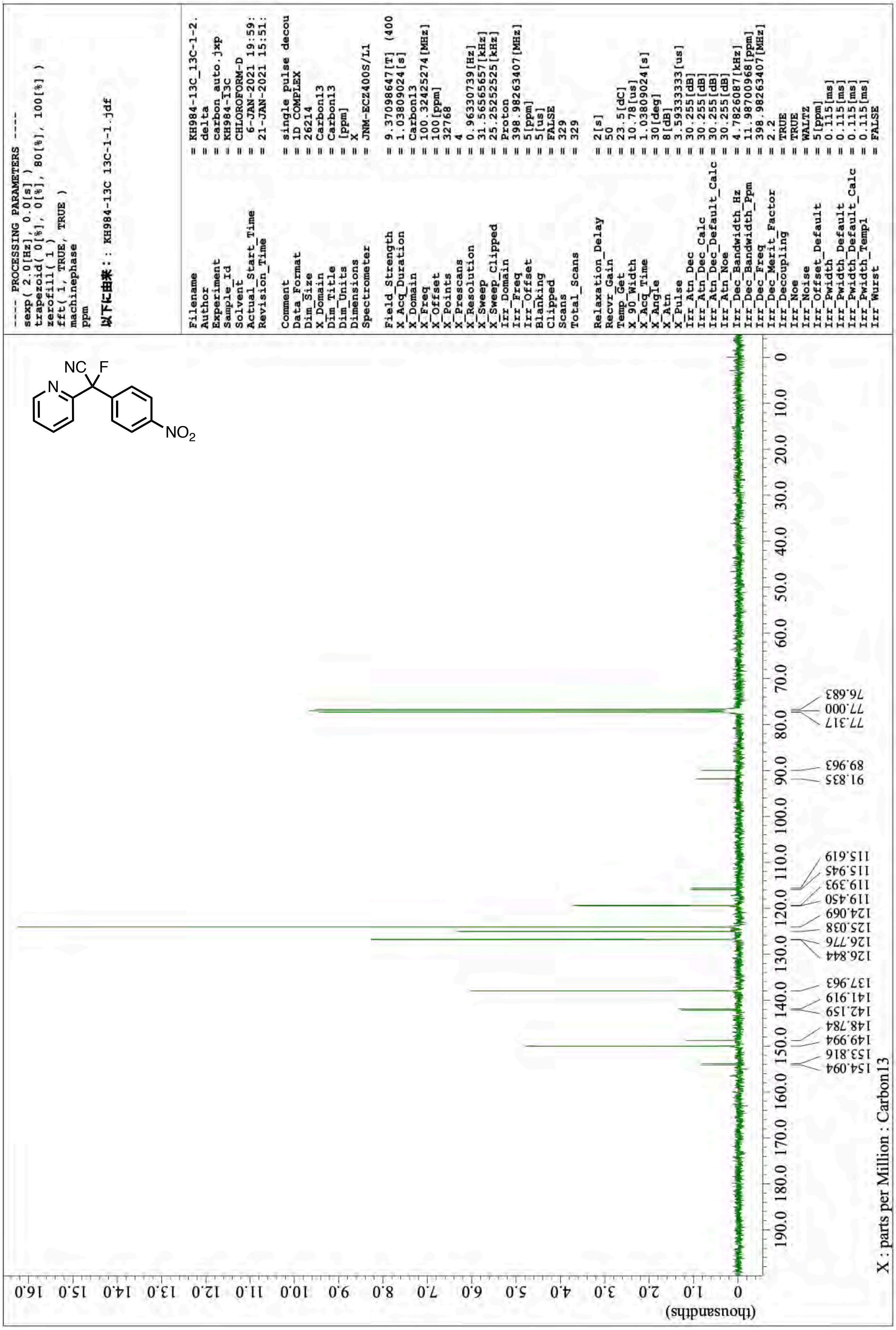

^{19}F NMR of **2K** (376 MHz, $CDCl_3$)

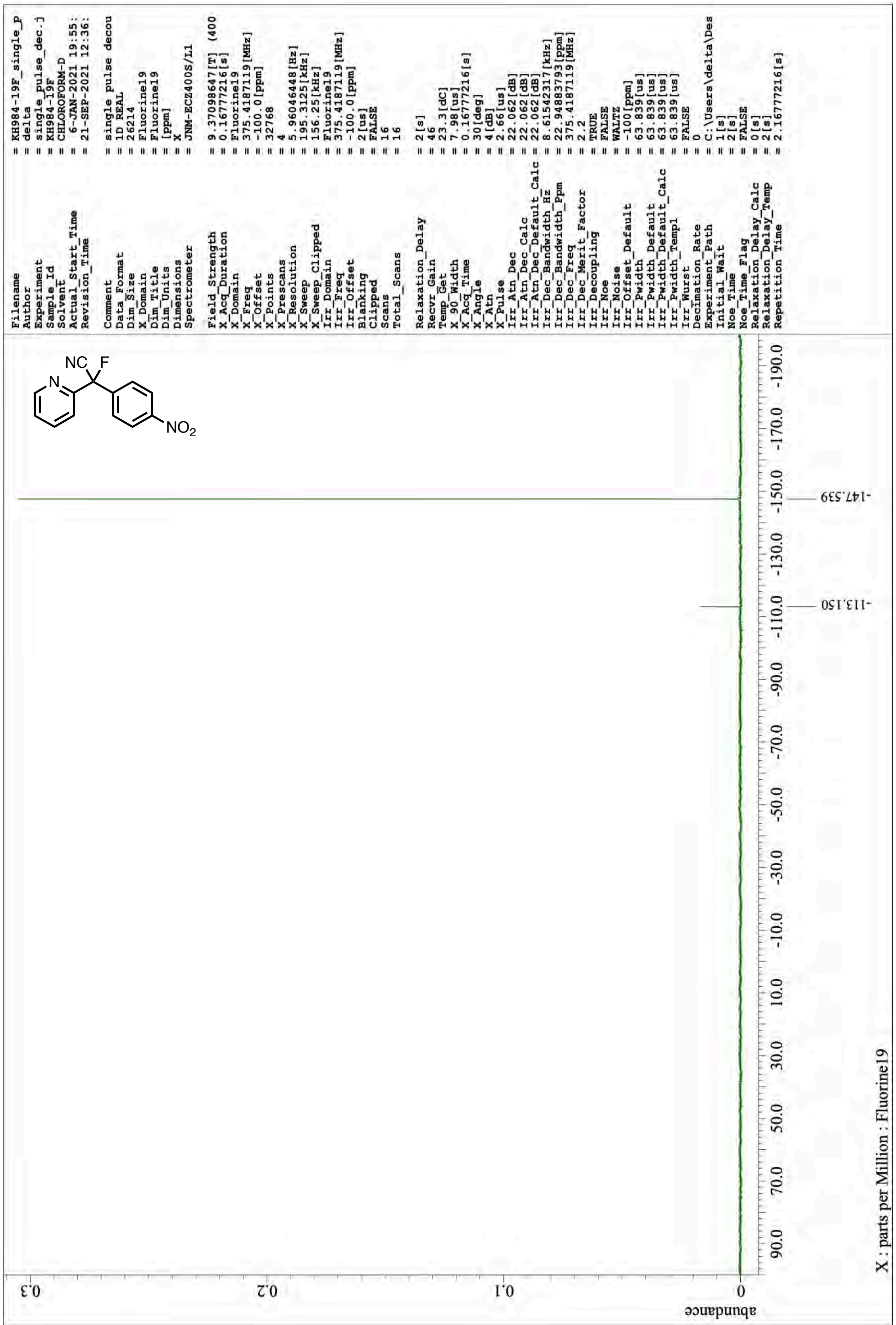

^{1}H NMR of **2L** (400 MHz, $CDCl_3$)

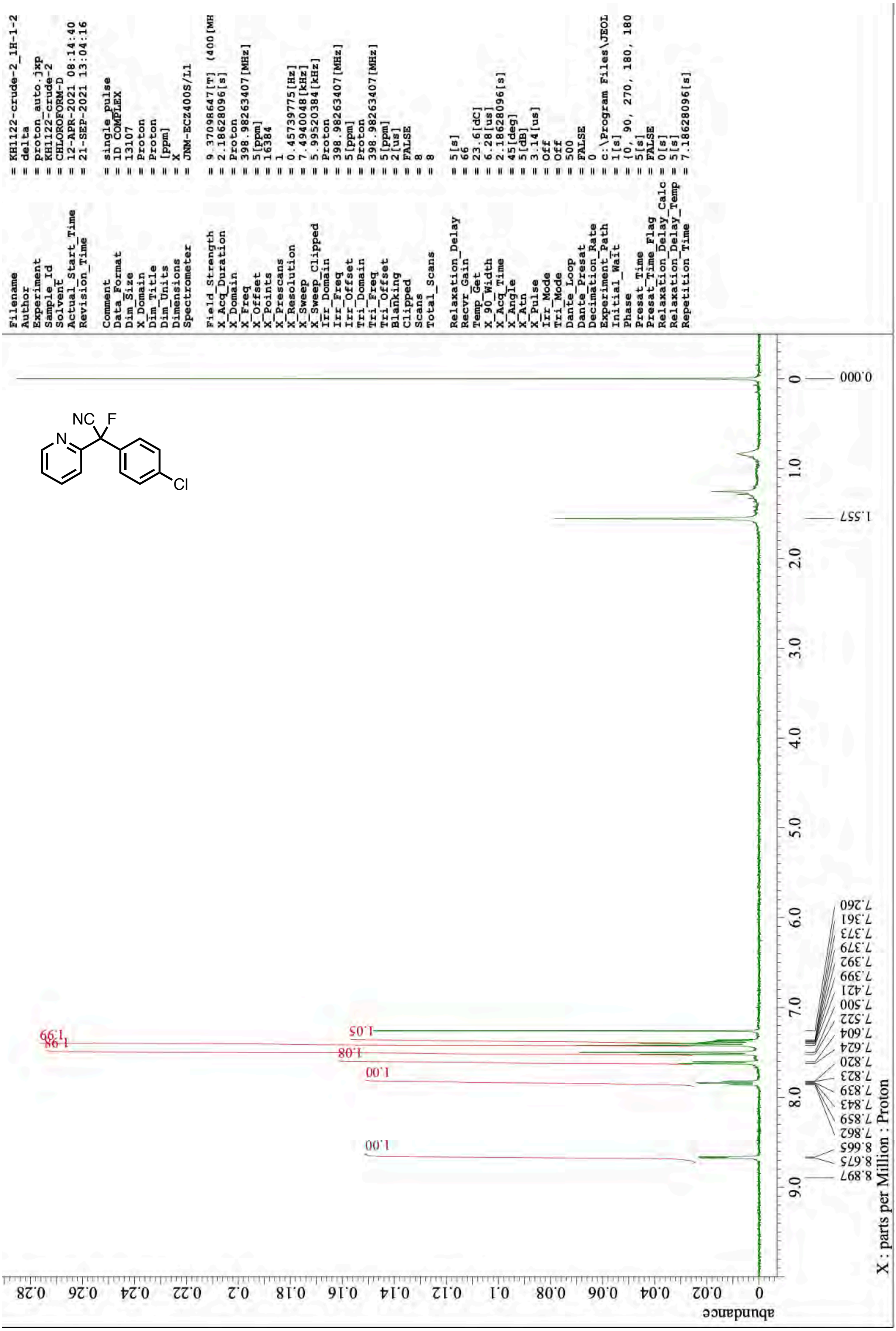

^{13}C NMR of **2L** (101 MHz, $CDCl_3$)

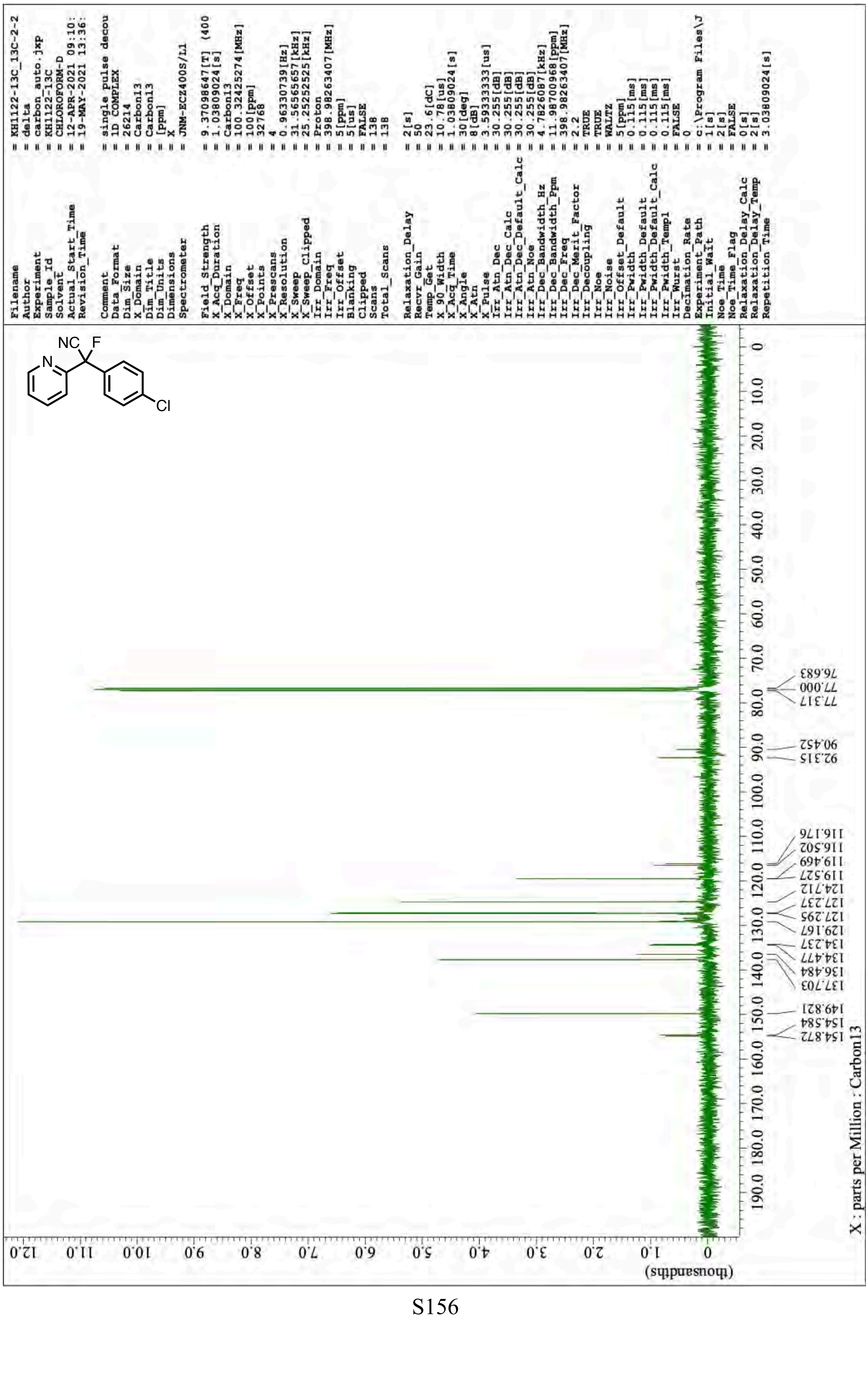

^{19}F NMR of **2L** (376 MHz, $CDCl_3$)

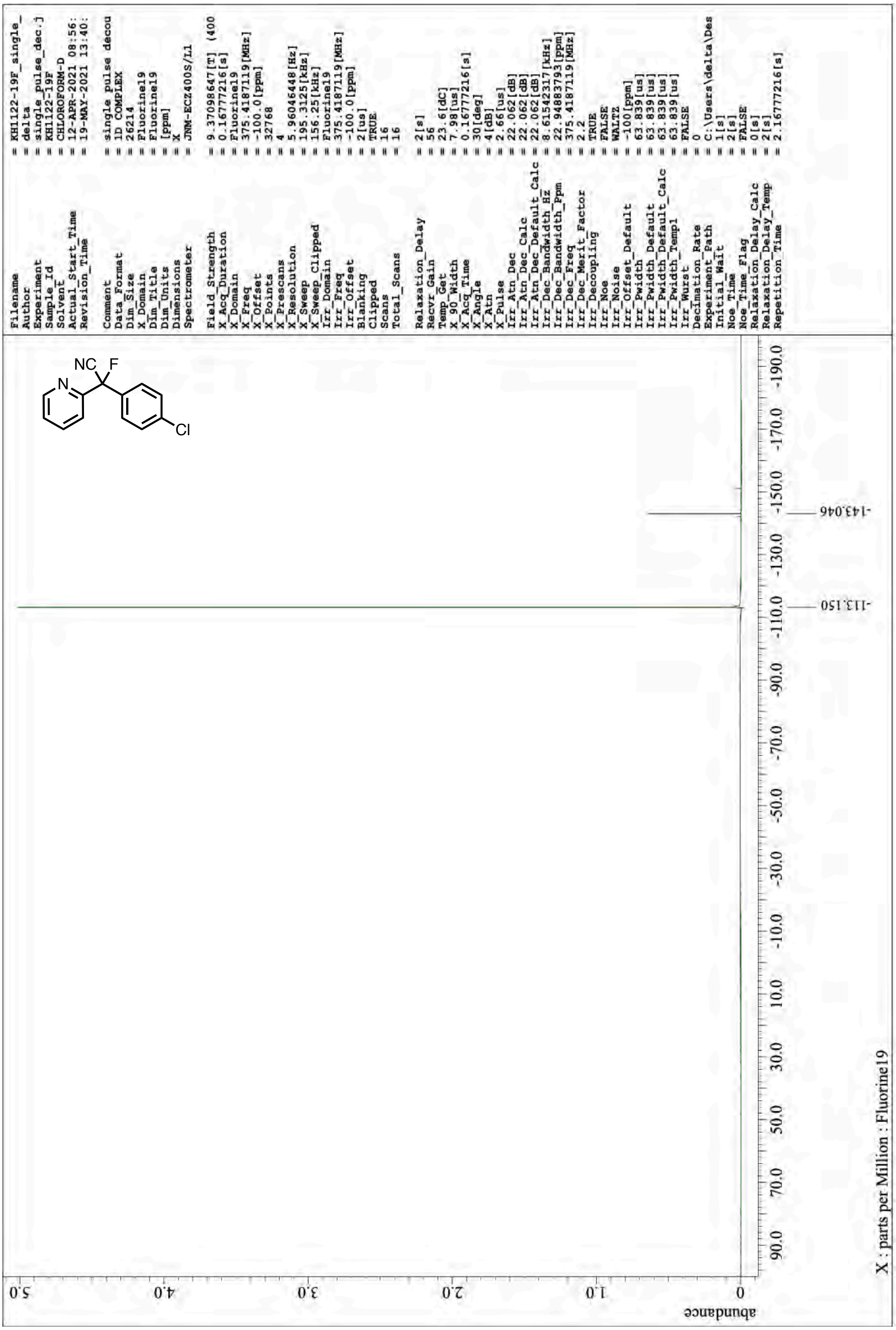

^{1}H NMR of **2M** (400 MHz, $CDCl_3$)

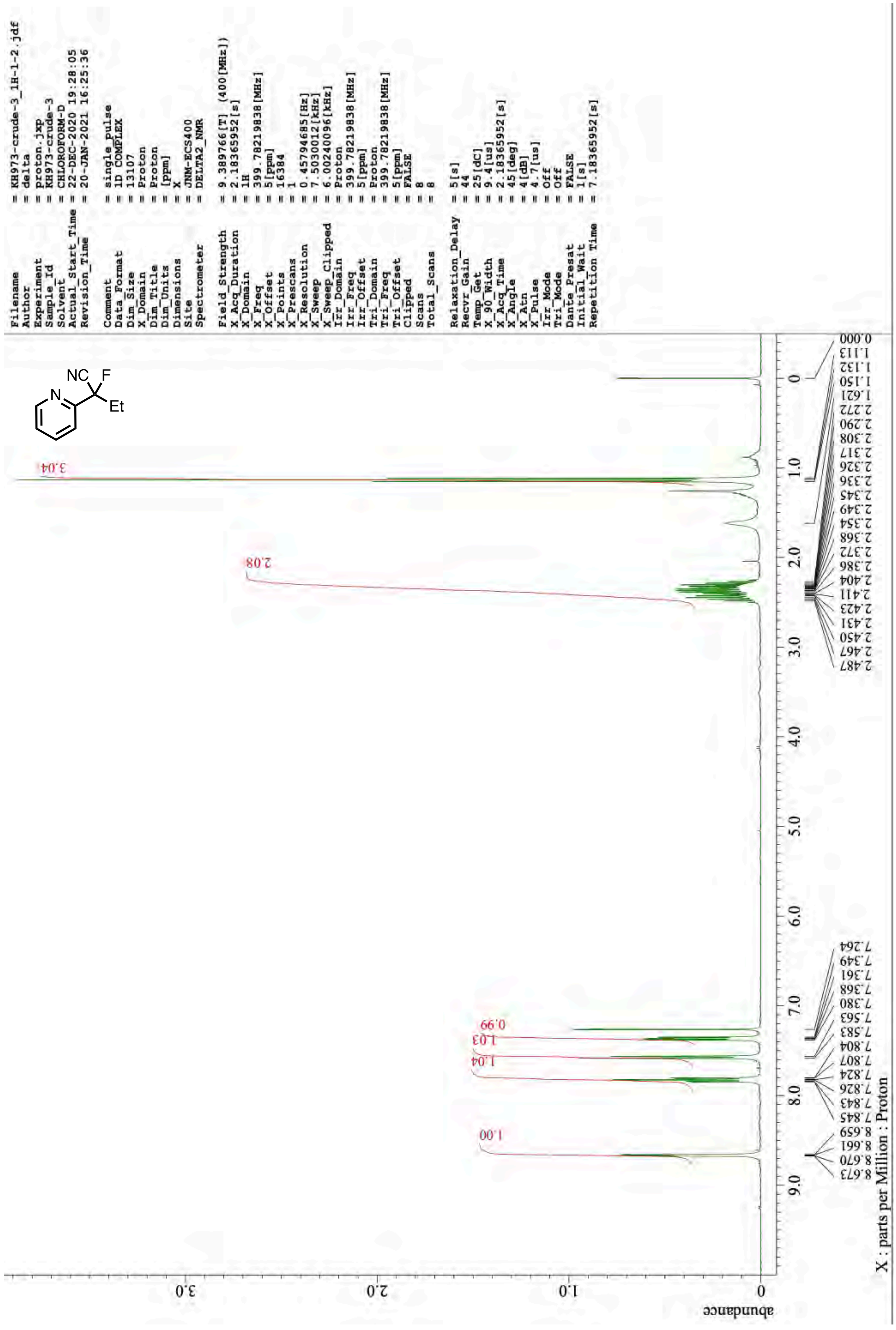

^{13}C NMR of **2M** (101 MHz, $CDCl_3$)

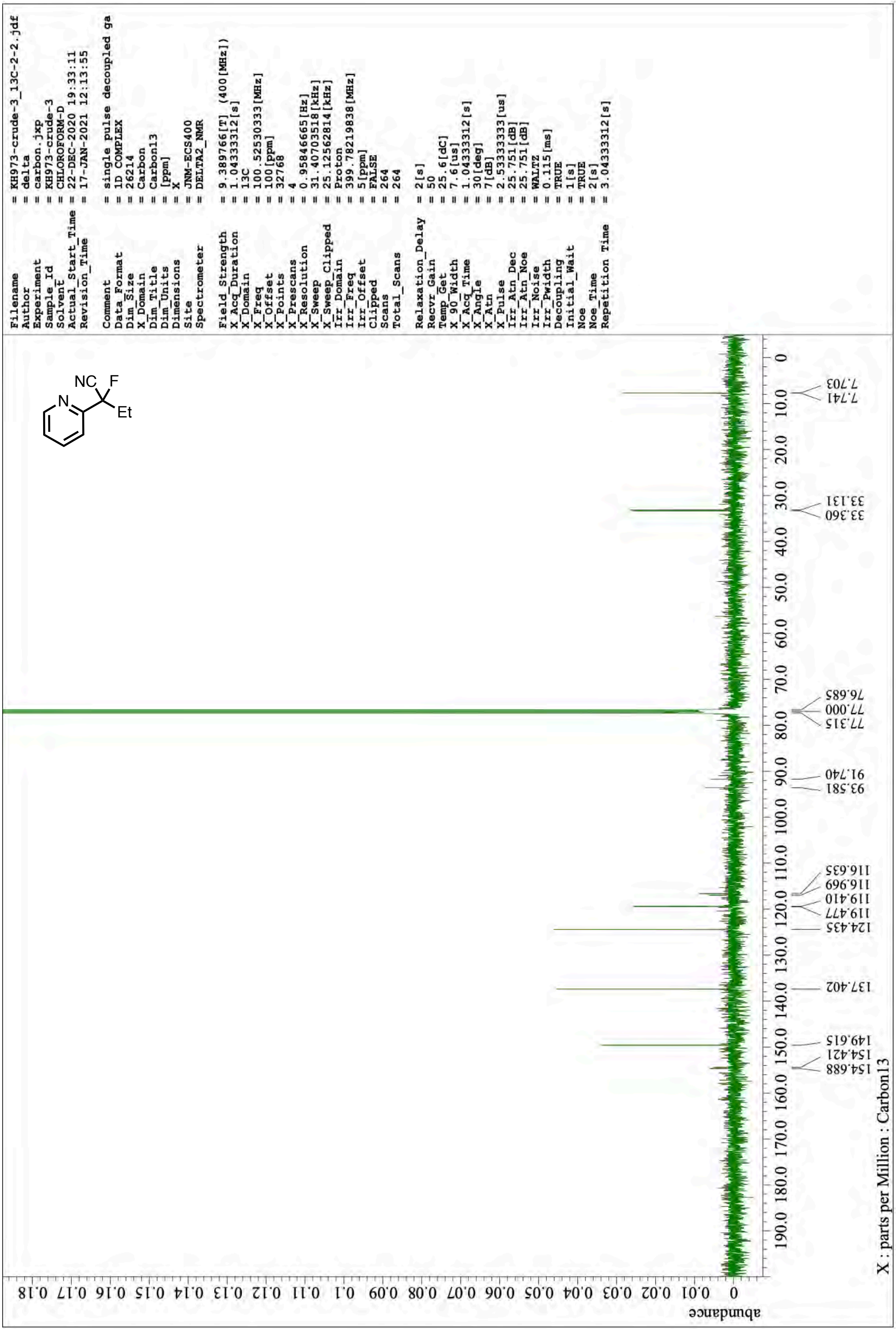

^{19}F NMR of **2M** (376 MHz, $CDCl_3$)

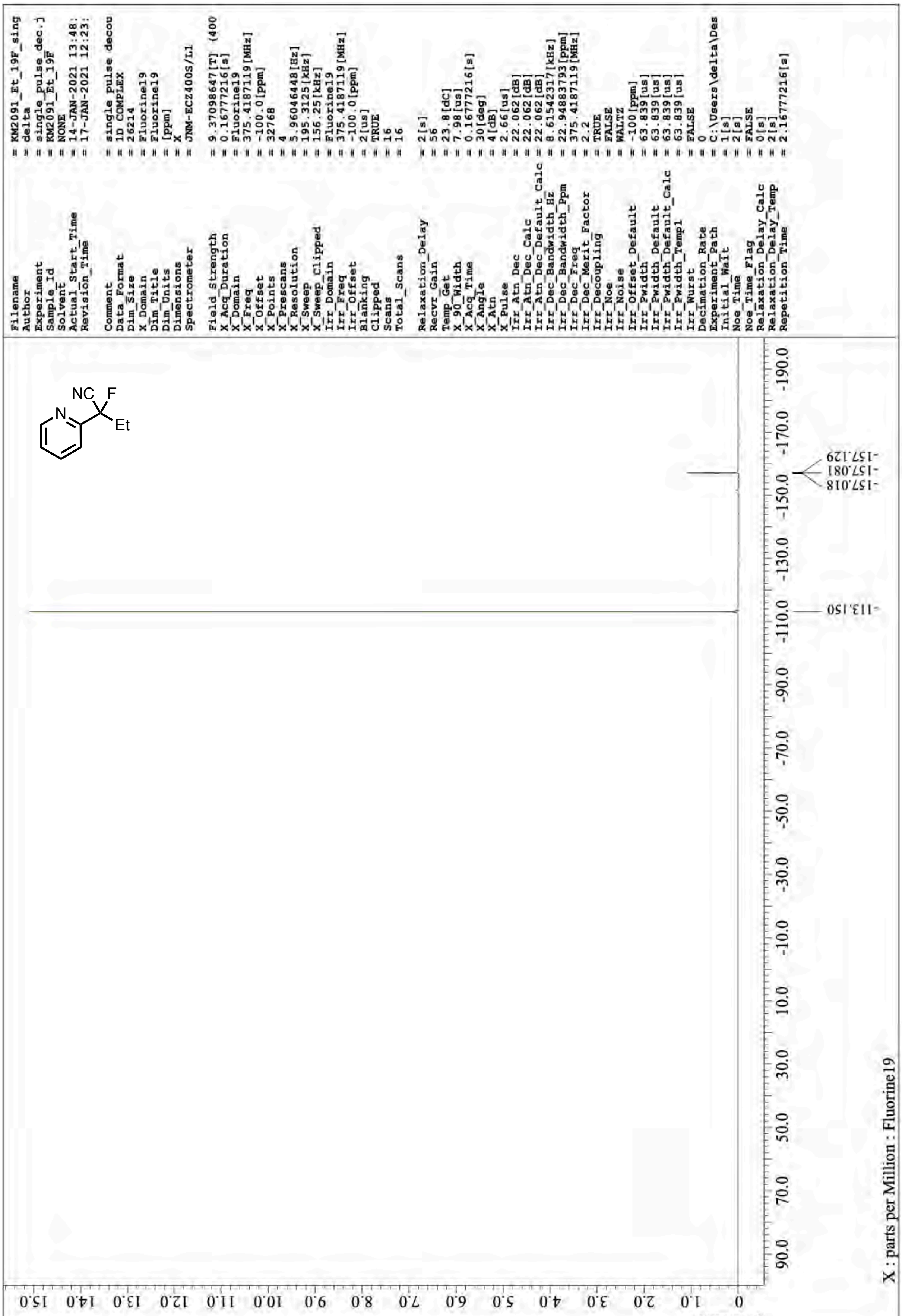

^{1}H NMR of **2N** (400 MHz, $CDCl_3$)

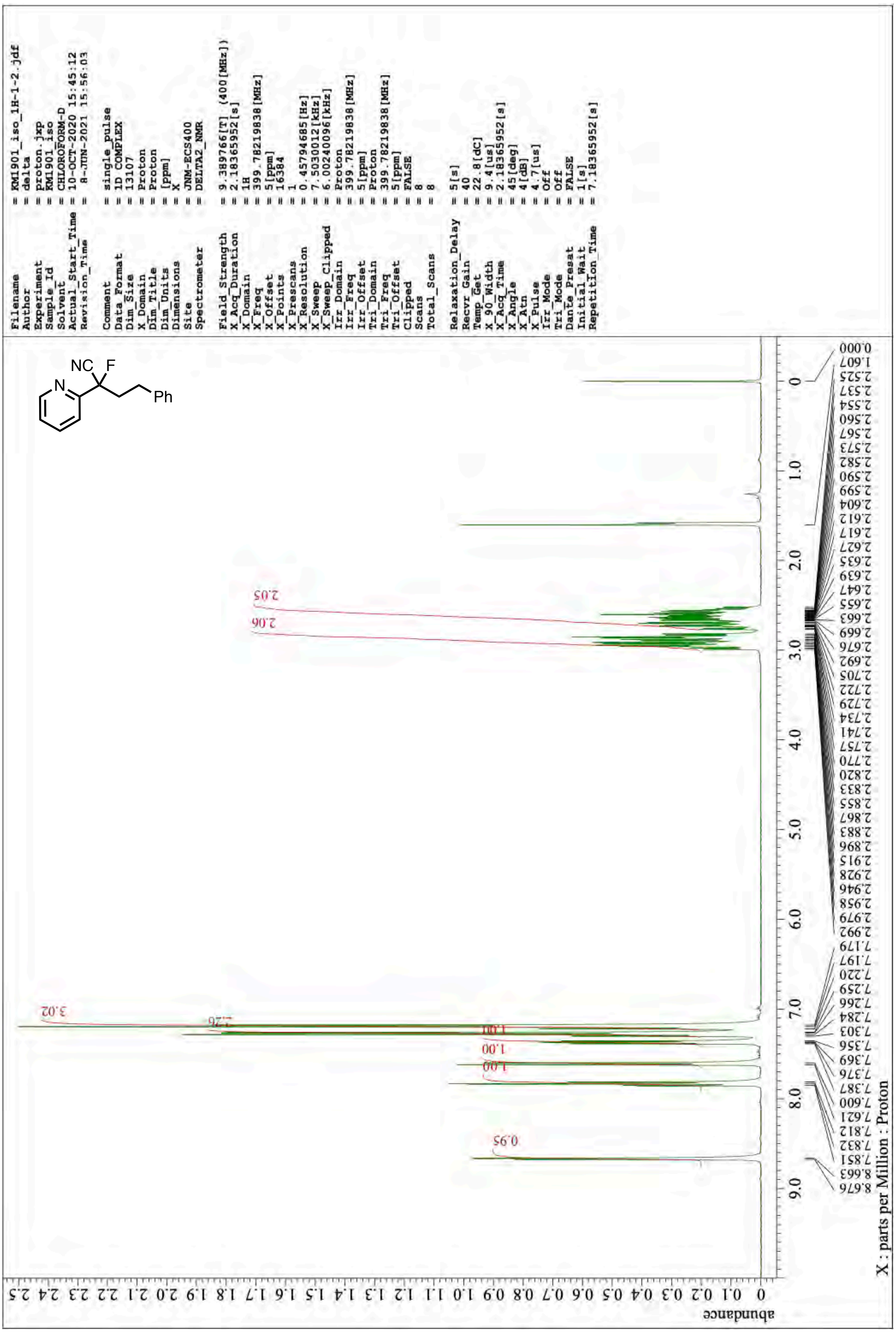

^{13}C NMR of **2N** (101 MHz, $CDCl_3$)

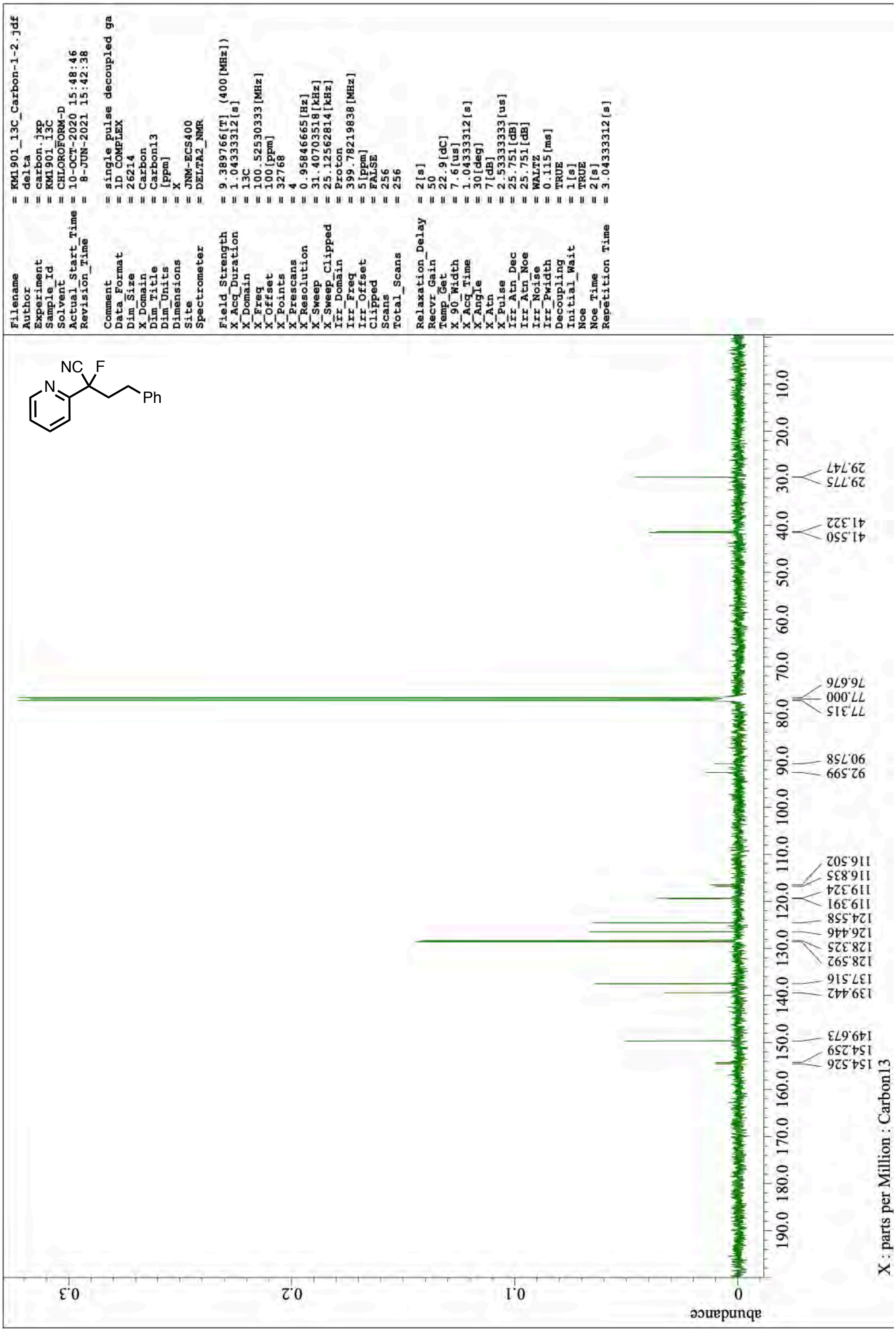

^{19}F NMR of **2N** (376 MHz, $CDCl_3$)

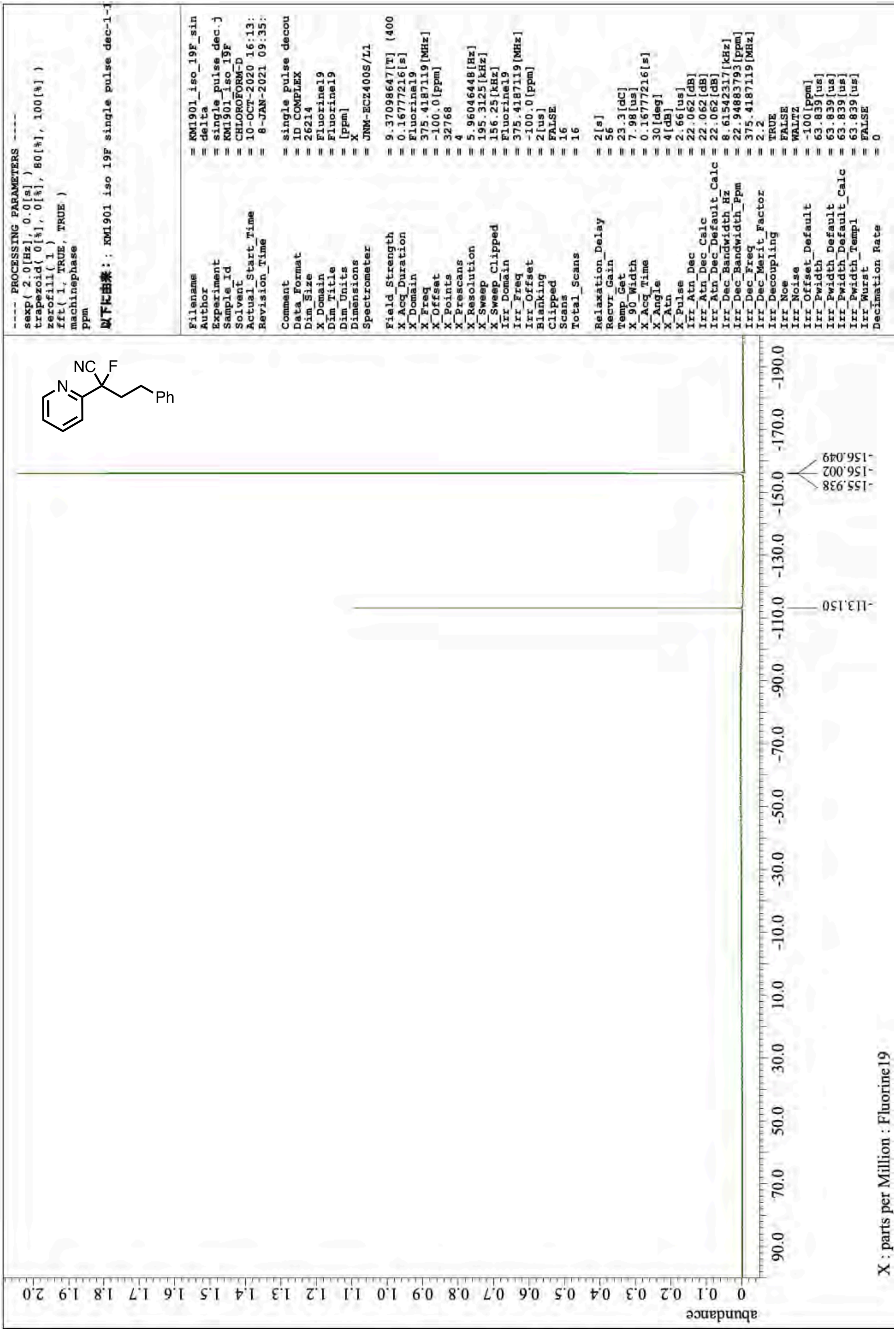

^{1}H NMR of **2O** (400 MHz, $CDCl_3$)

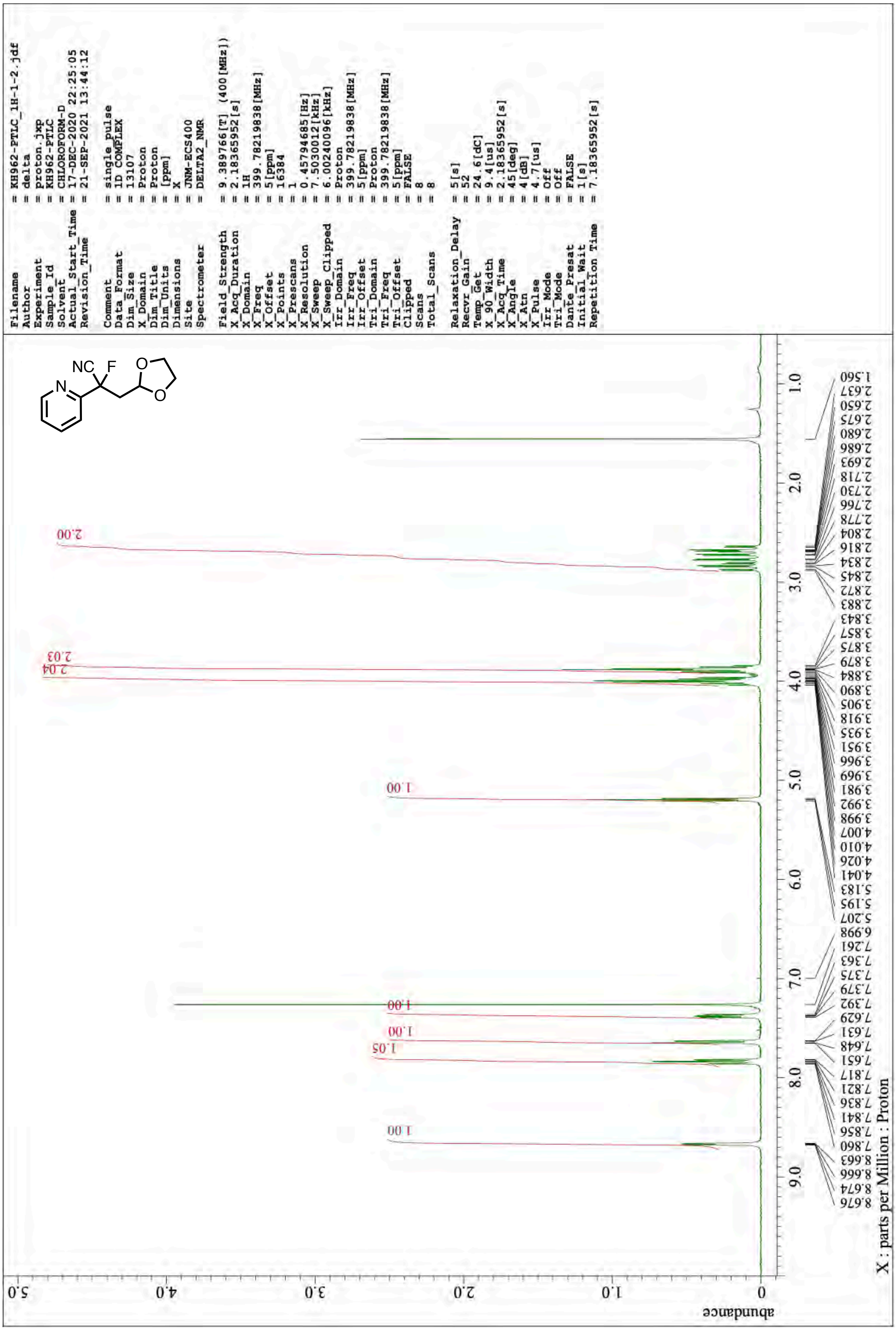

^{13}C NMR of **2O** (101 MHz, $CDCl_3$)

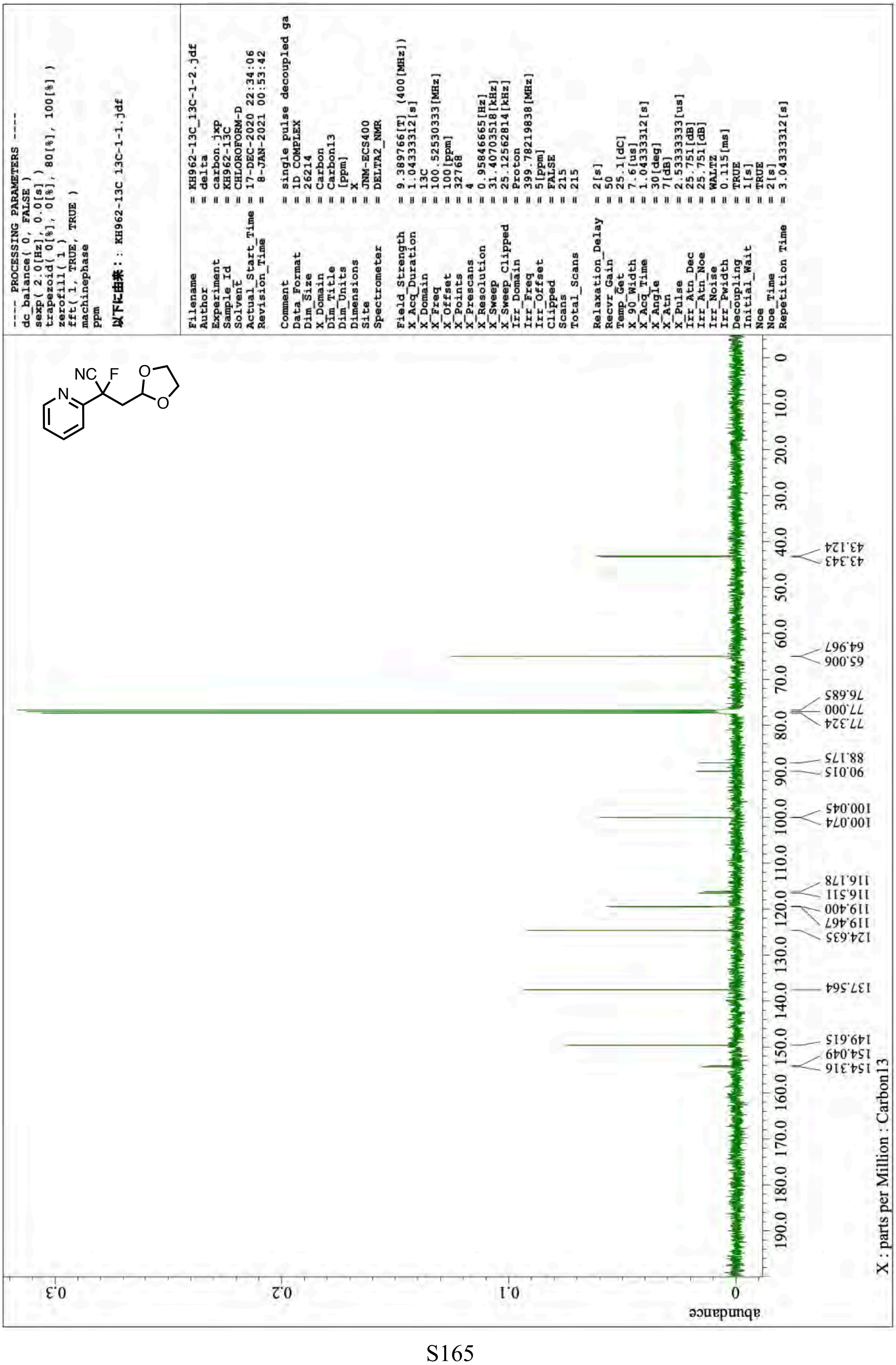

^{19}F NMR of **2O** (376 MHz, $CDCl_3$)

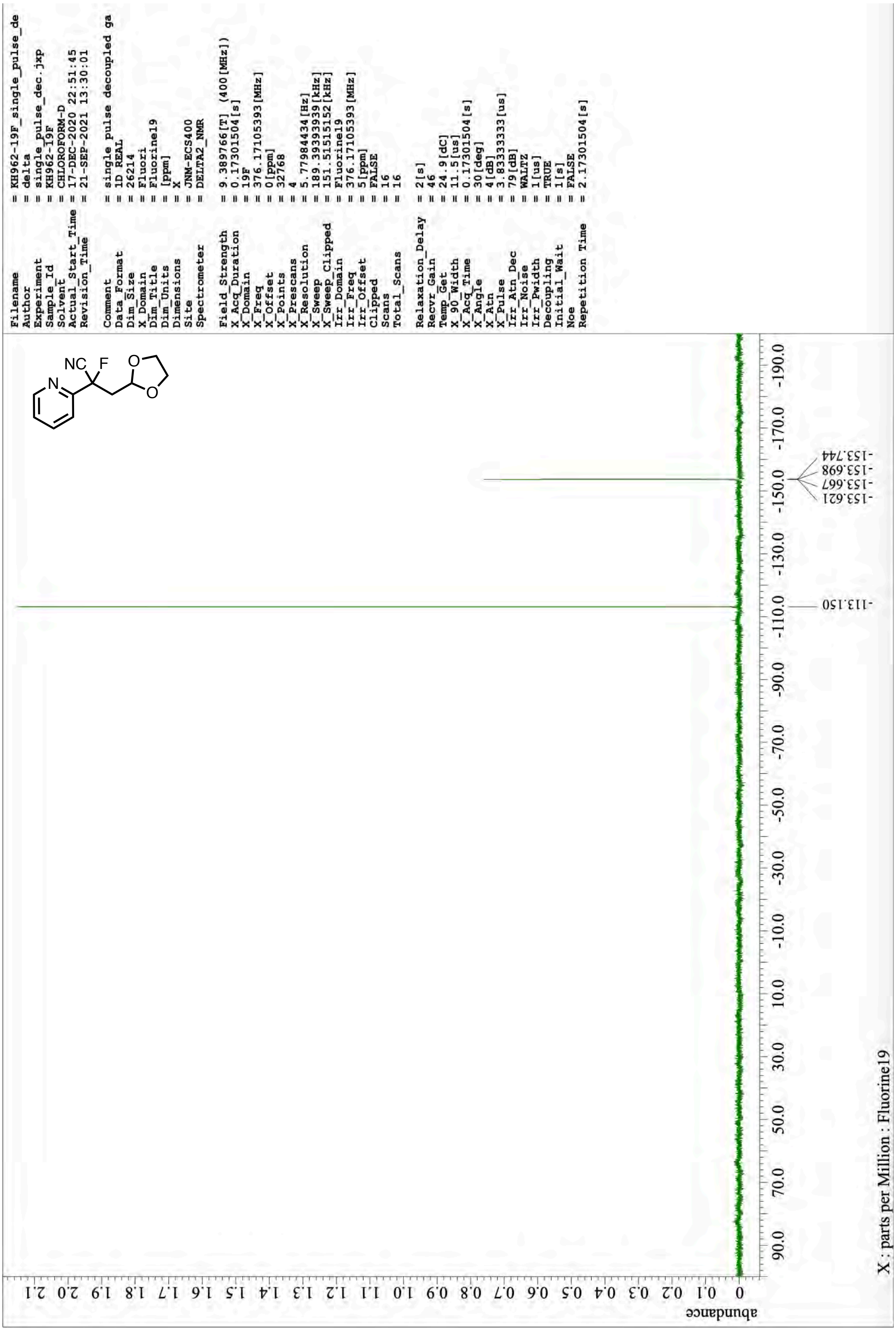

^{1}H NMR of **2P** (400 MHz, $CDCl_3$)

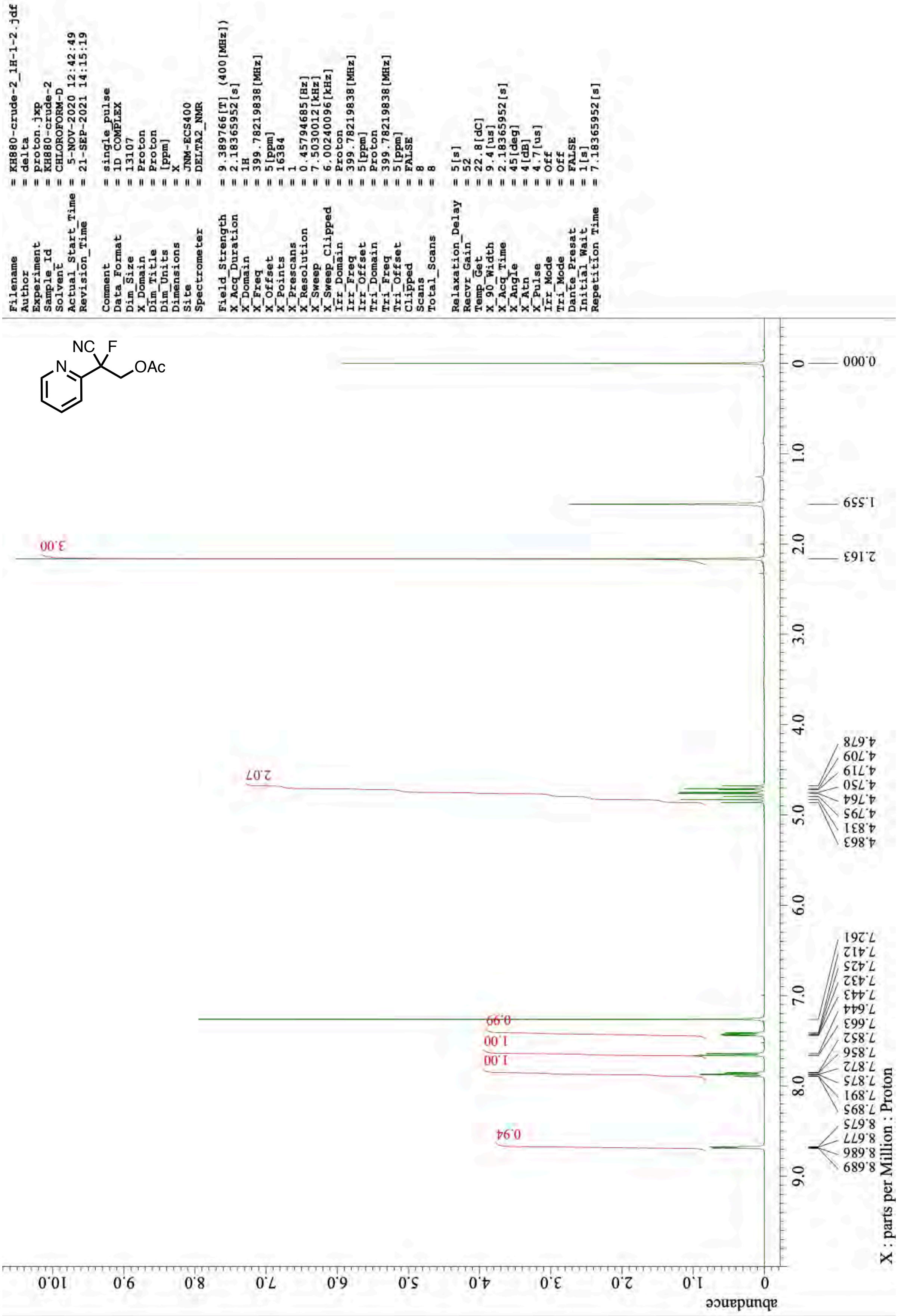

^{13}C NMR of **2P** (101 MHz, $CDCl_3$)

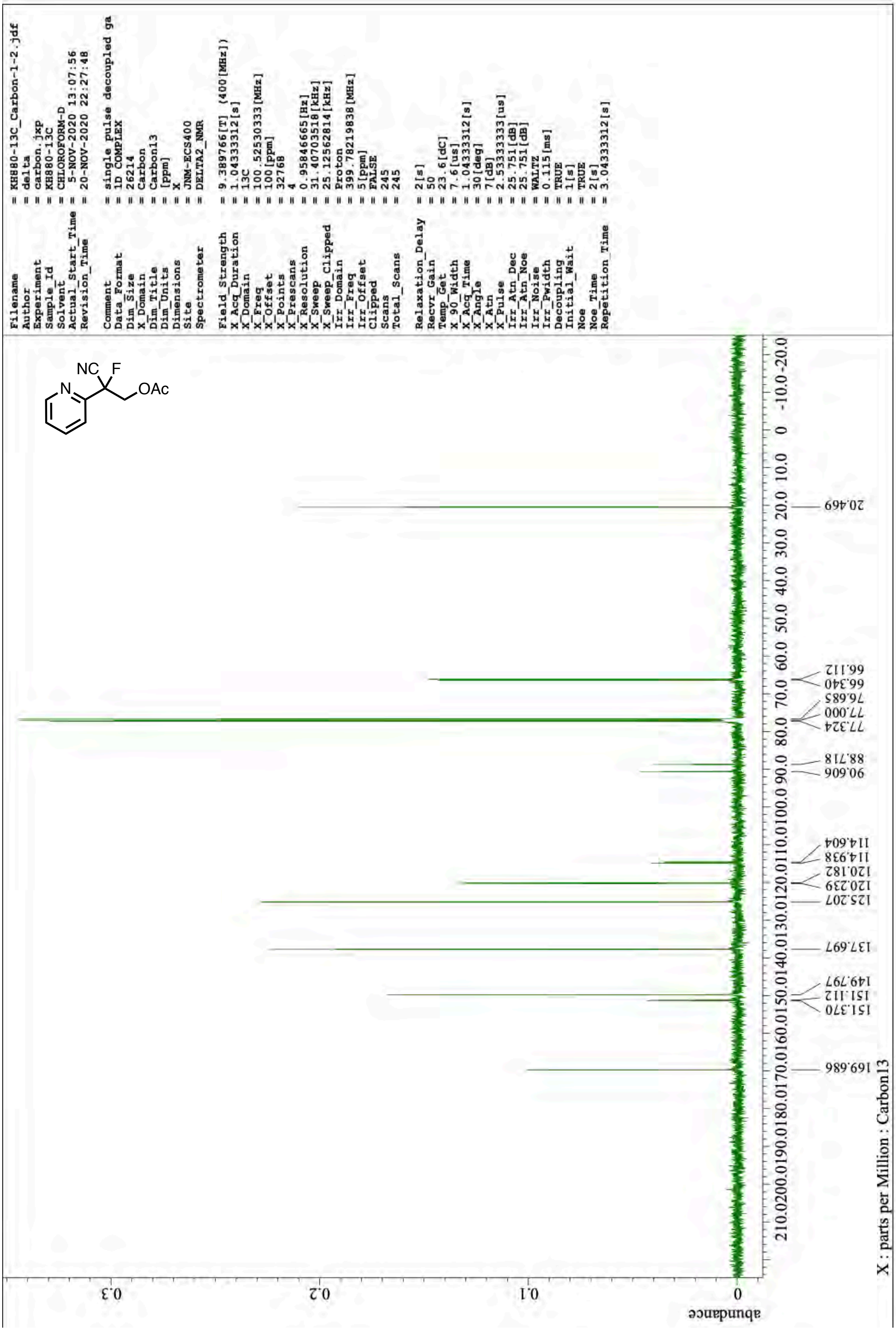

^{19}F NMR of **2P** (376 MHz, $CDCl_3$)

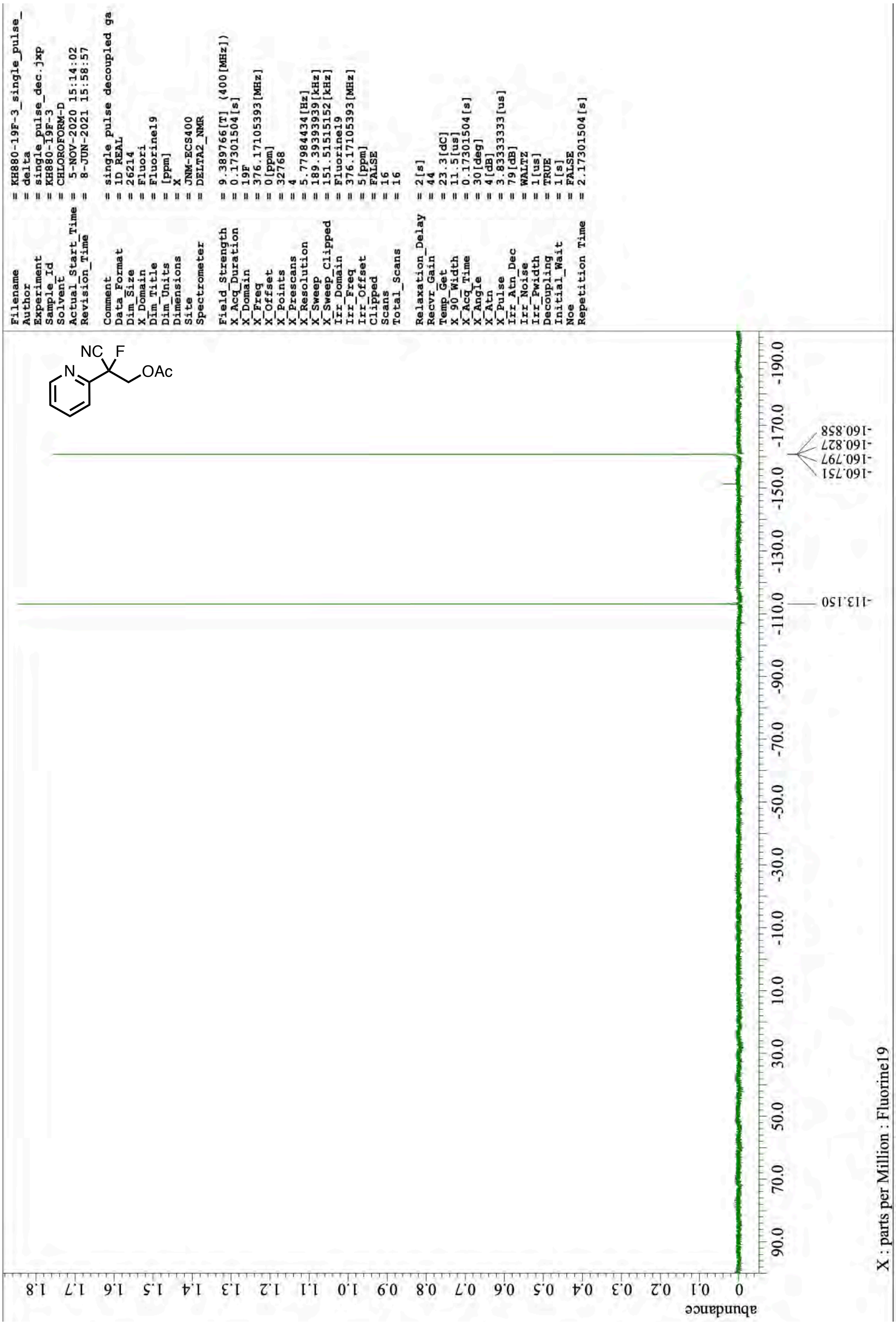

^{1}H NMR of **2Q** (400 MHz, $CDCl_3$)

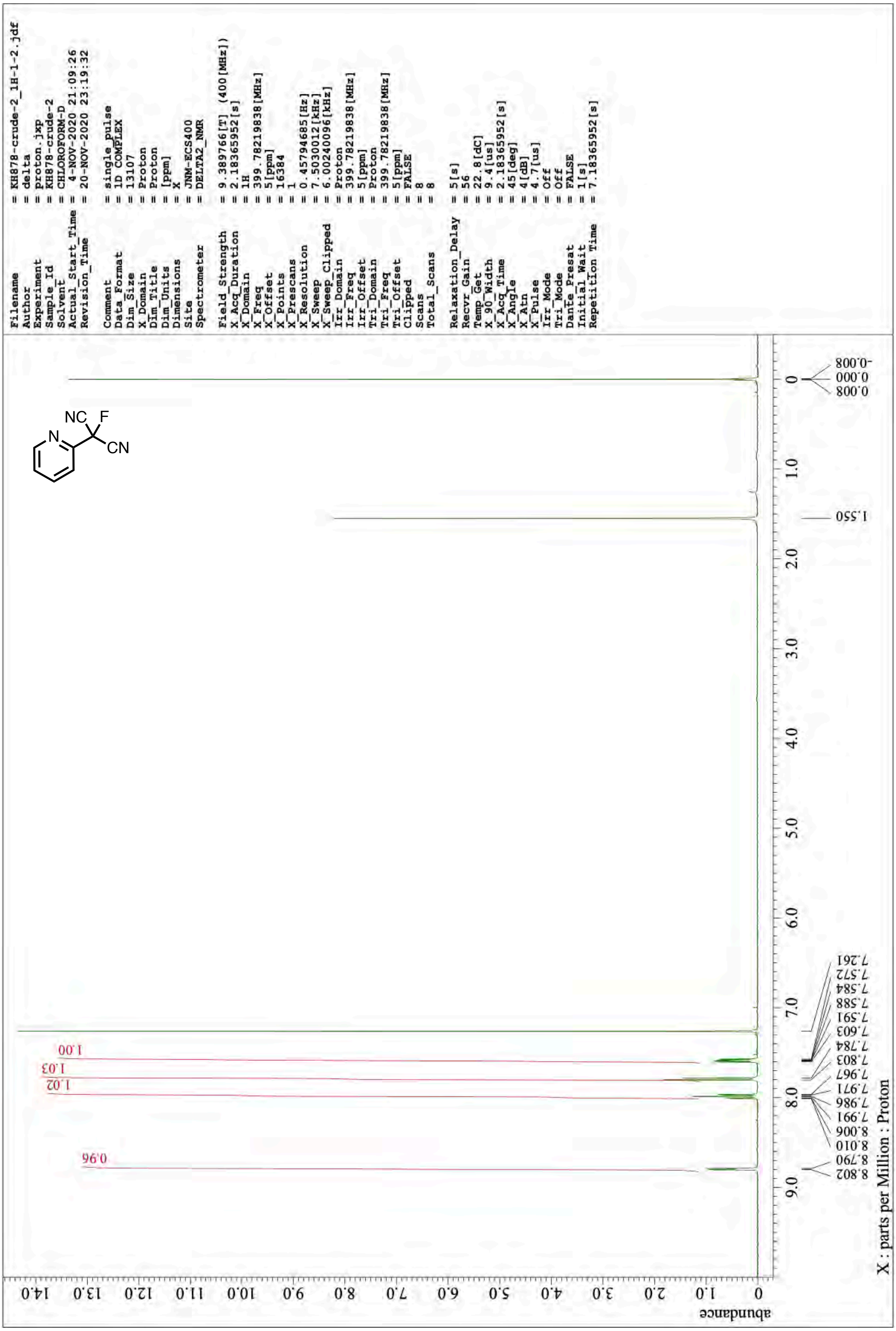

^{13}C NMR of **2Q** (101 MHz, $CDCl_3$)

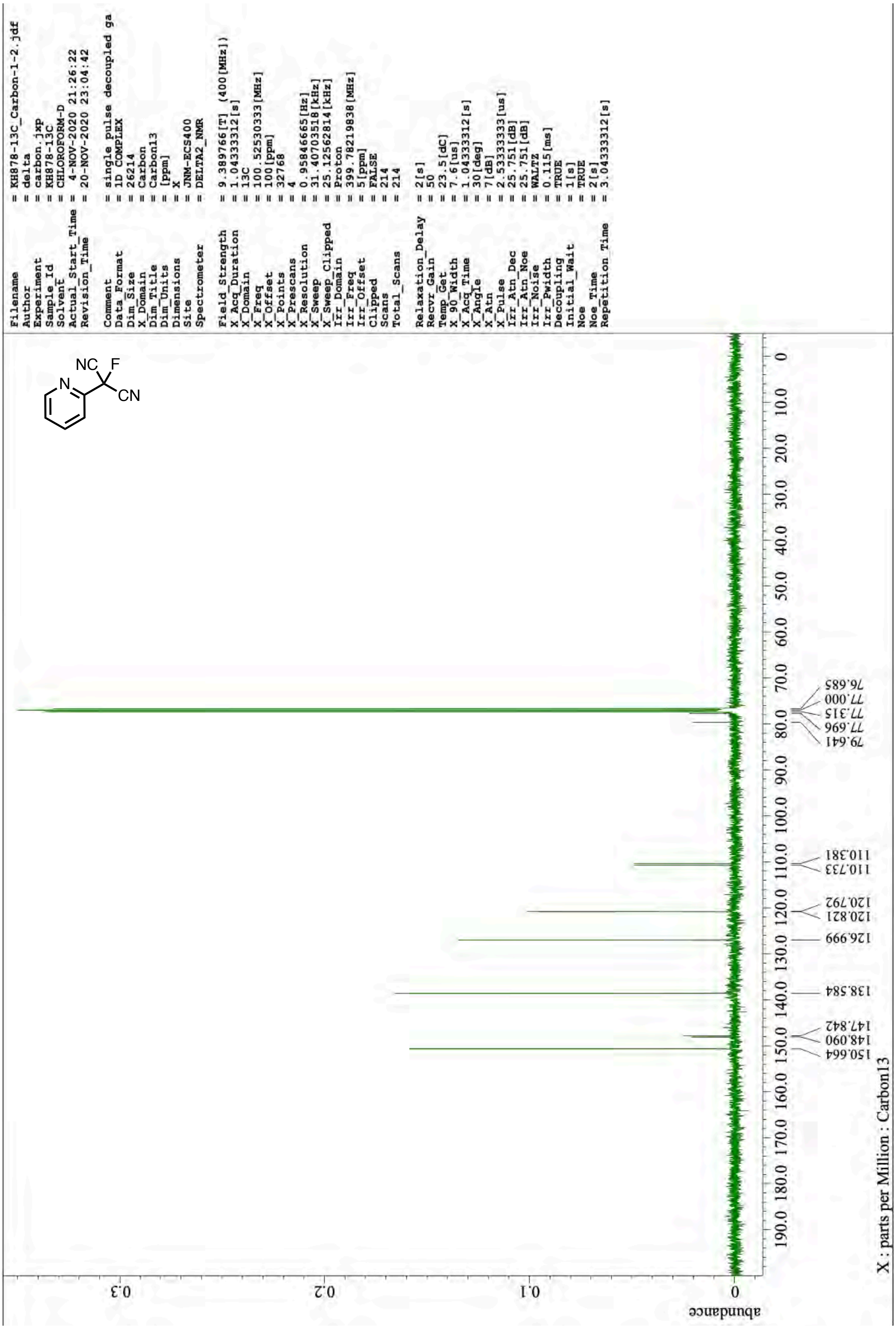

^{19}F NMR of **2Q** (376 MHz, $CDCl_3$)

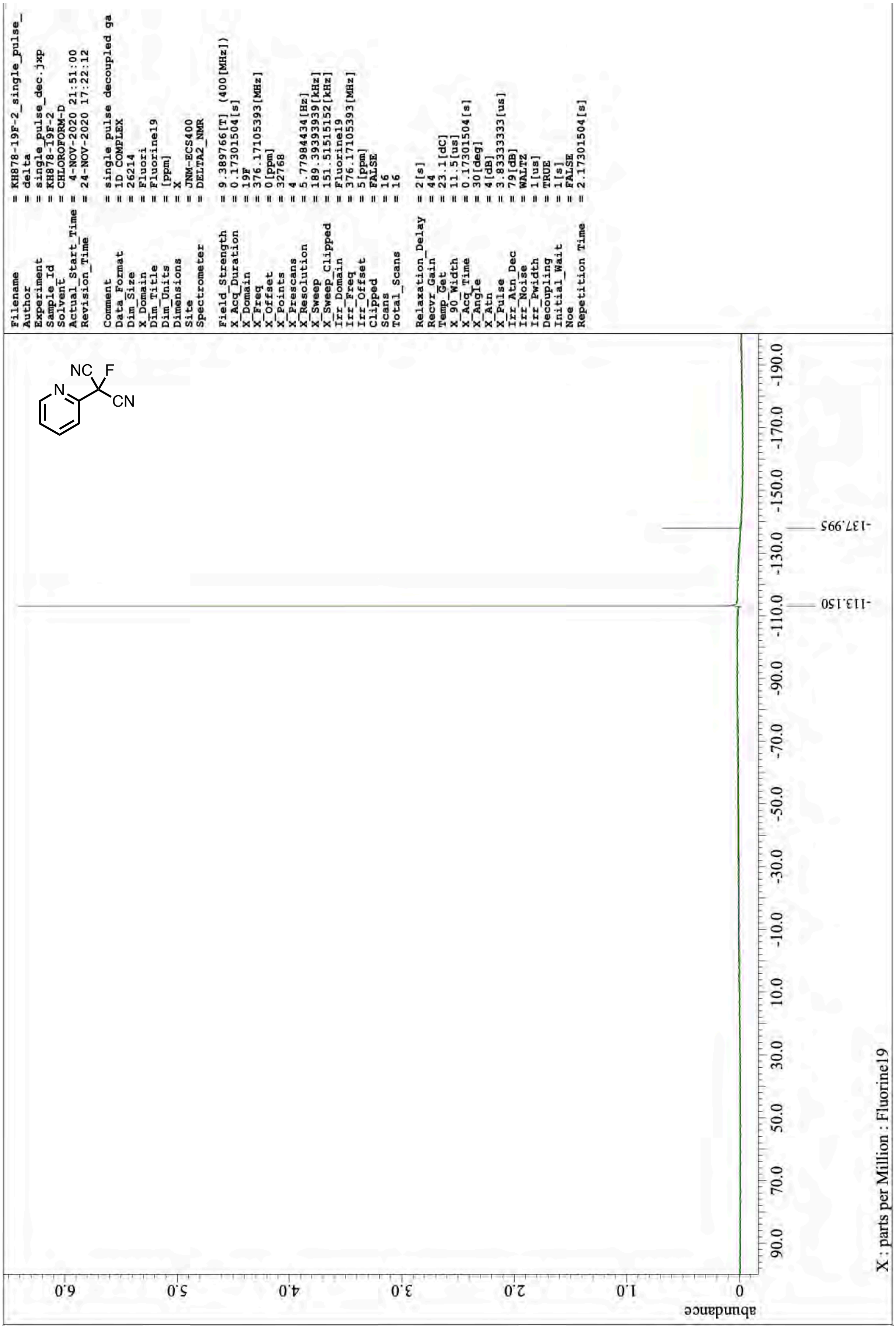

^{1}H NMR of **2R** (400 MHz, $CDCl_3$)

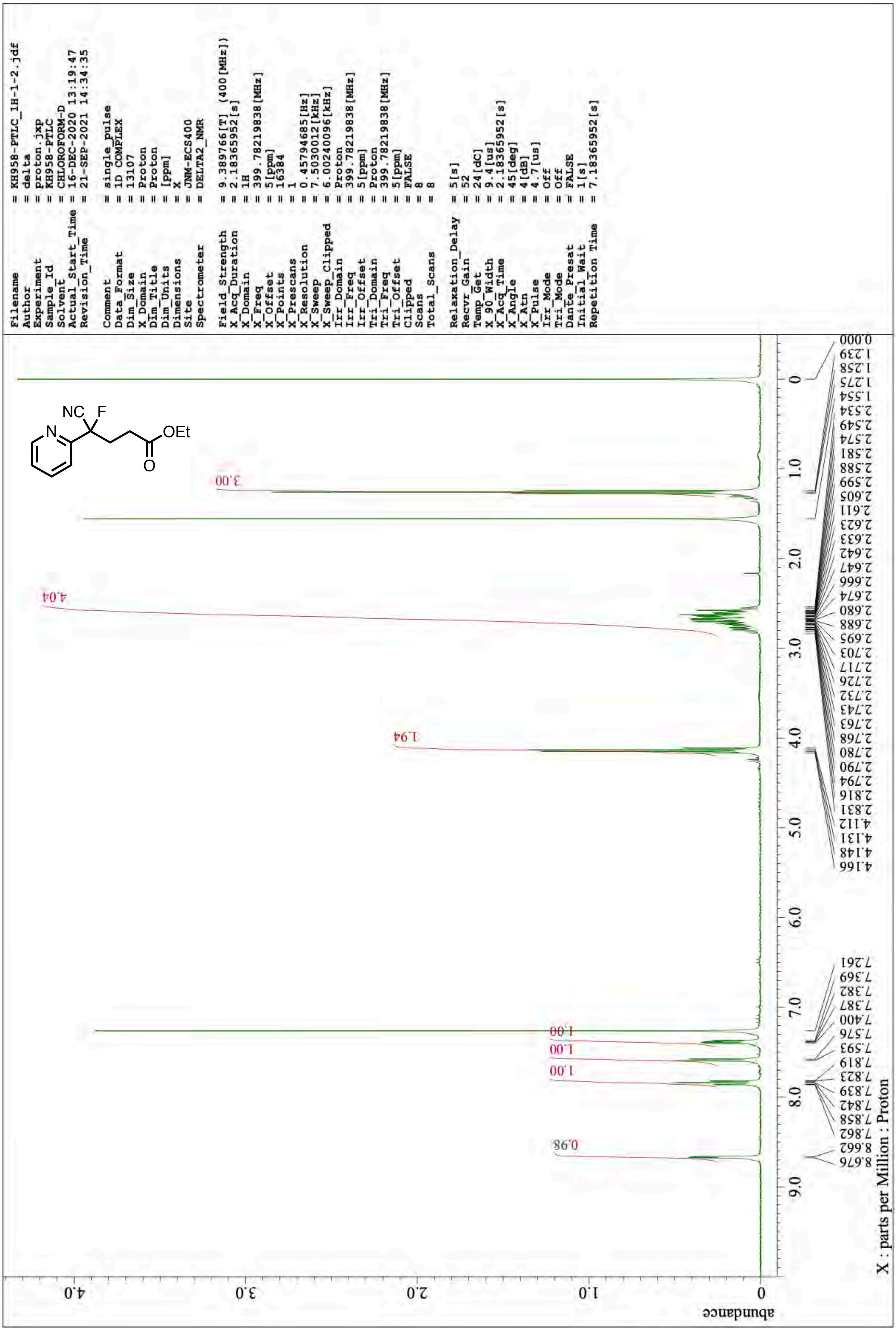

^{13}C NMR of **2R** (101 MHz, $CDCl_3$)

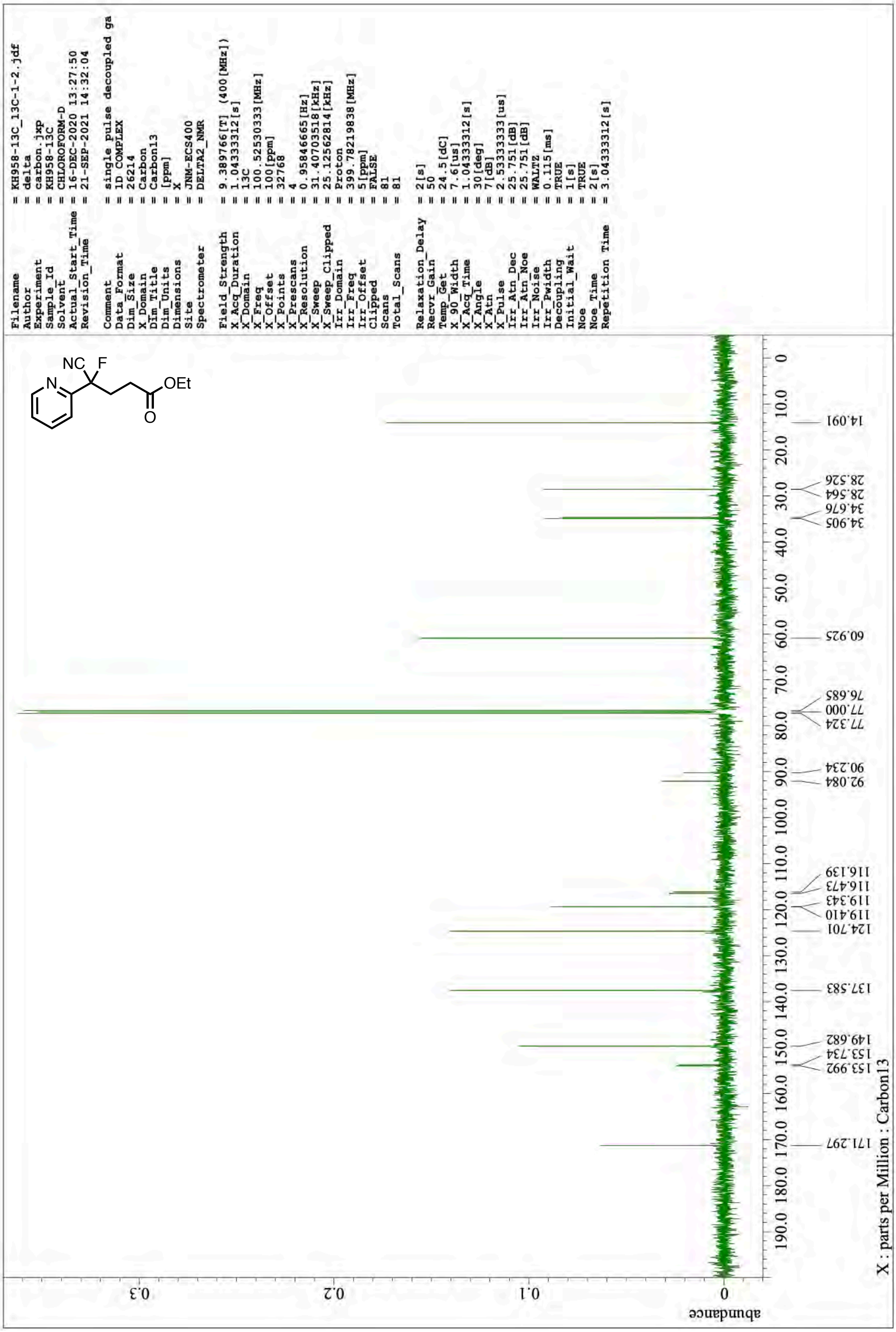

^{19}F NMR of **2R** (376 MHz, $CDCl_3$)

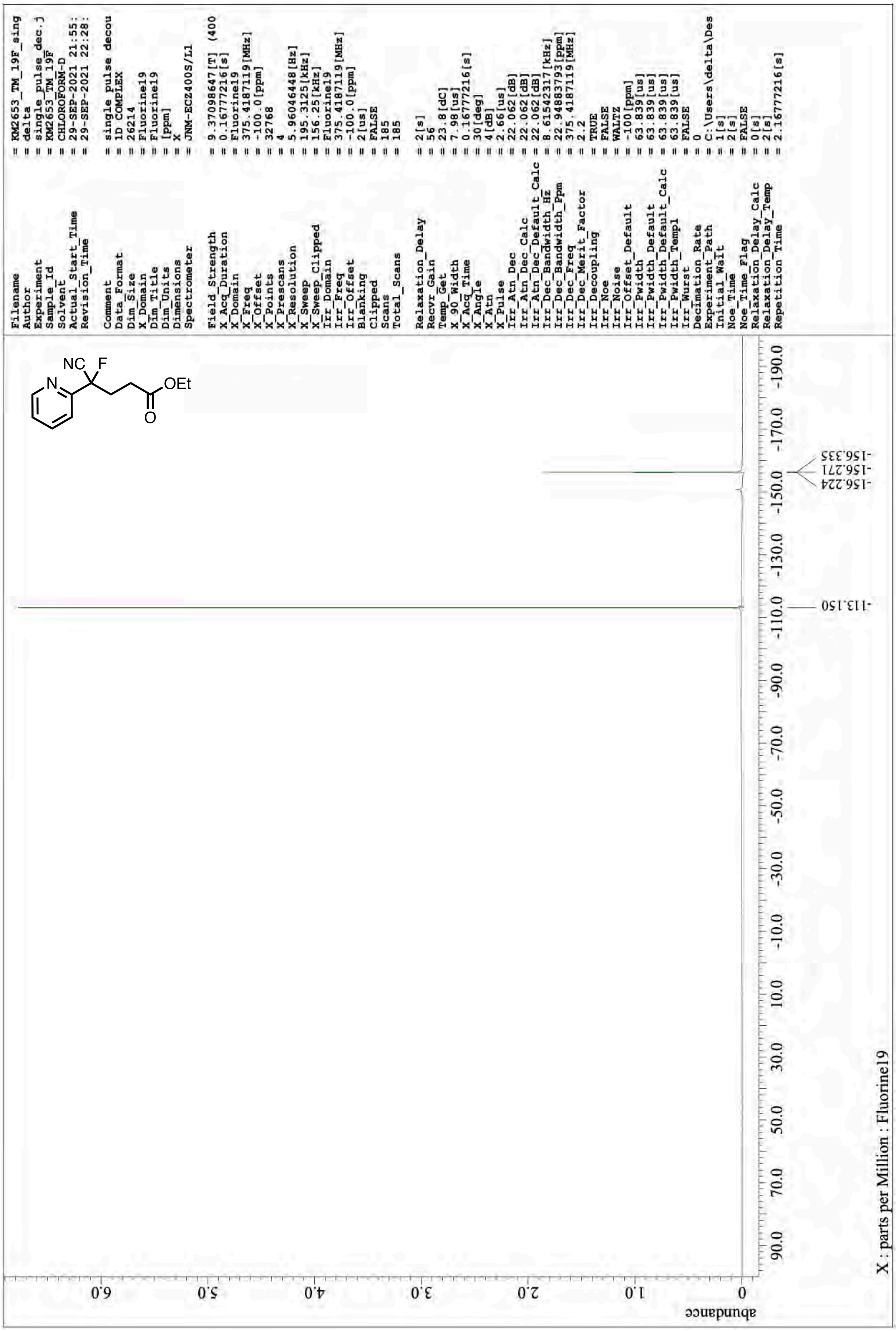

^{1}H NMR of **2S** (400 MHz, $CDCl_3$)

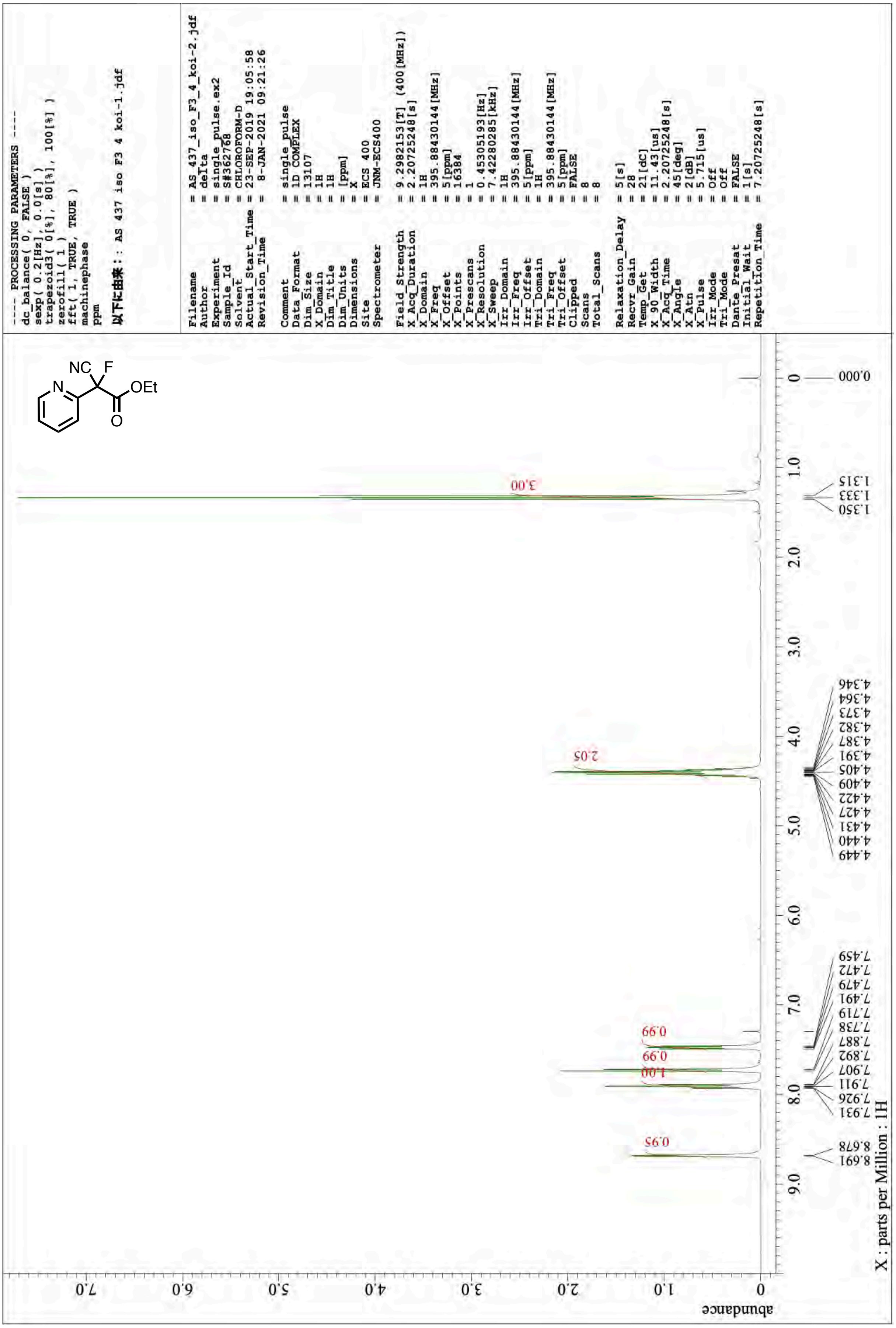

^{13}C NMR of **2S** (101 MHz, $CDCl_3$)

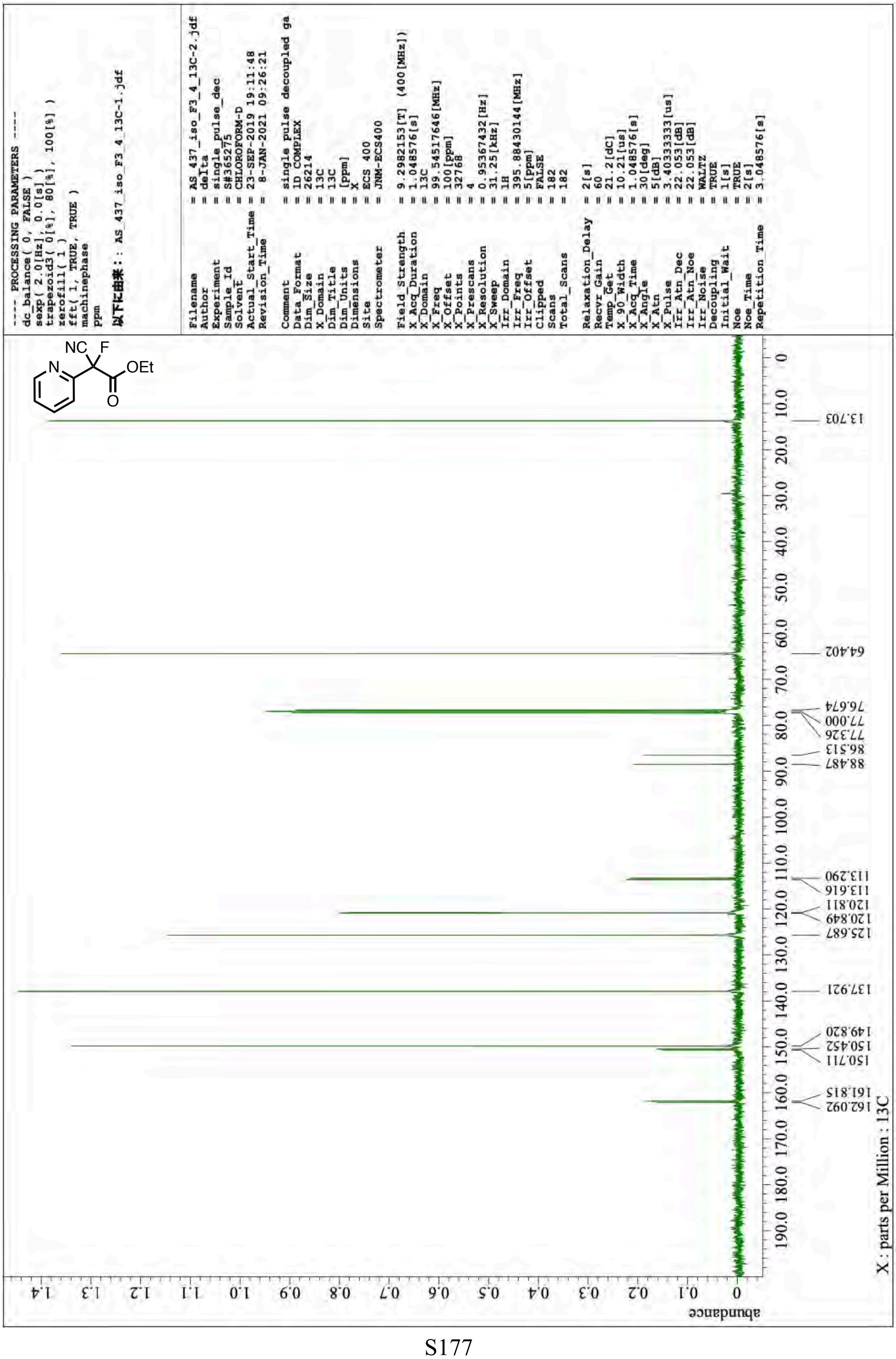

^{19}F NMR of **2S** (376 MHz, $CDCl_3$)

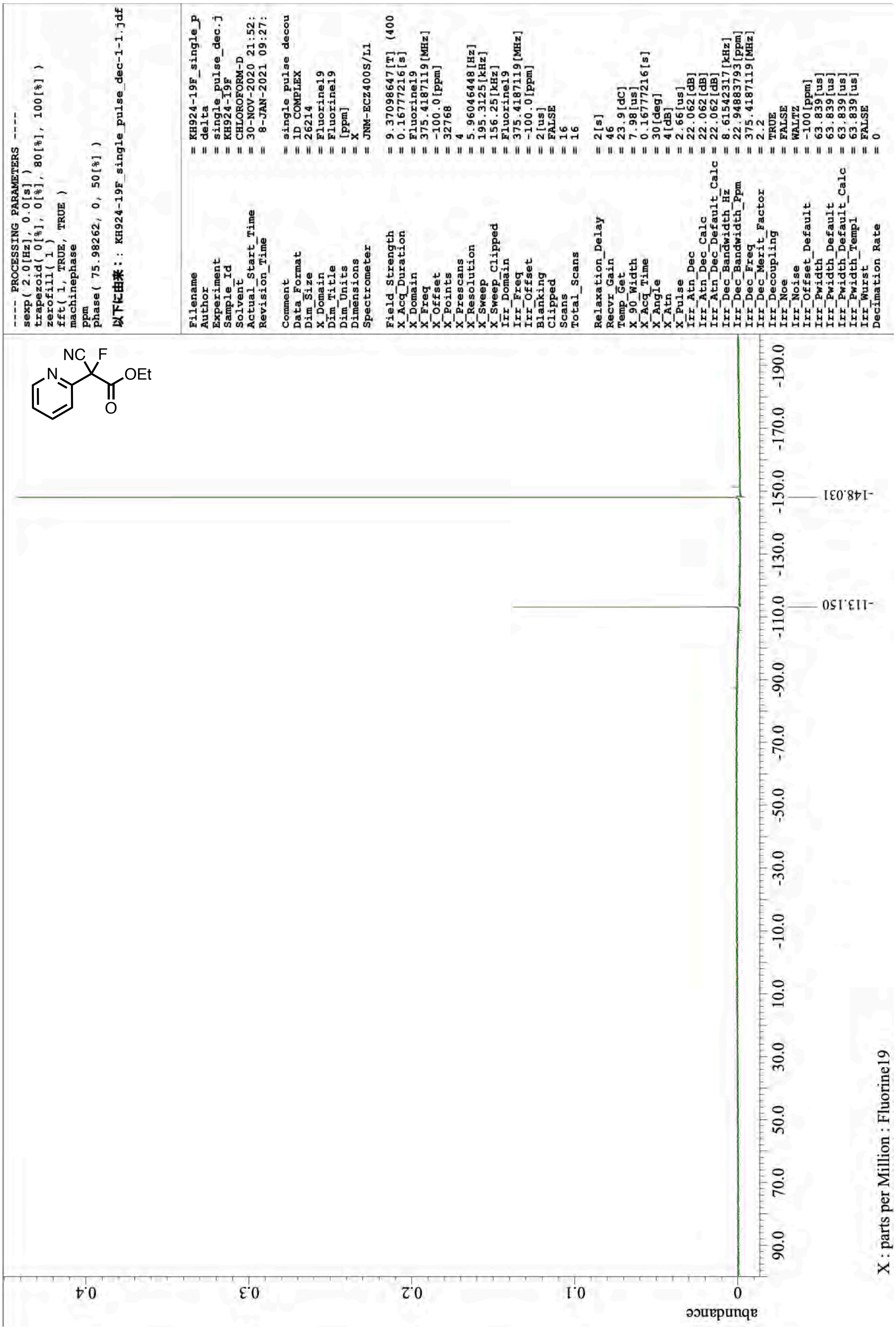

^{1}H NMR of **2T** (400 MHz, $CDCl_3$)

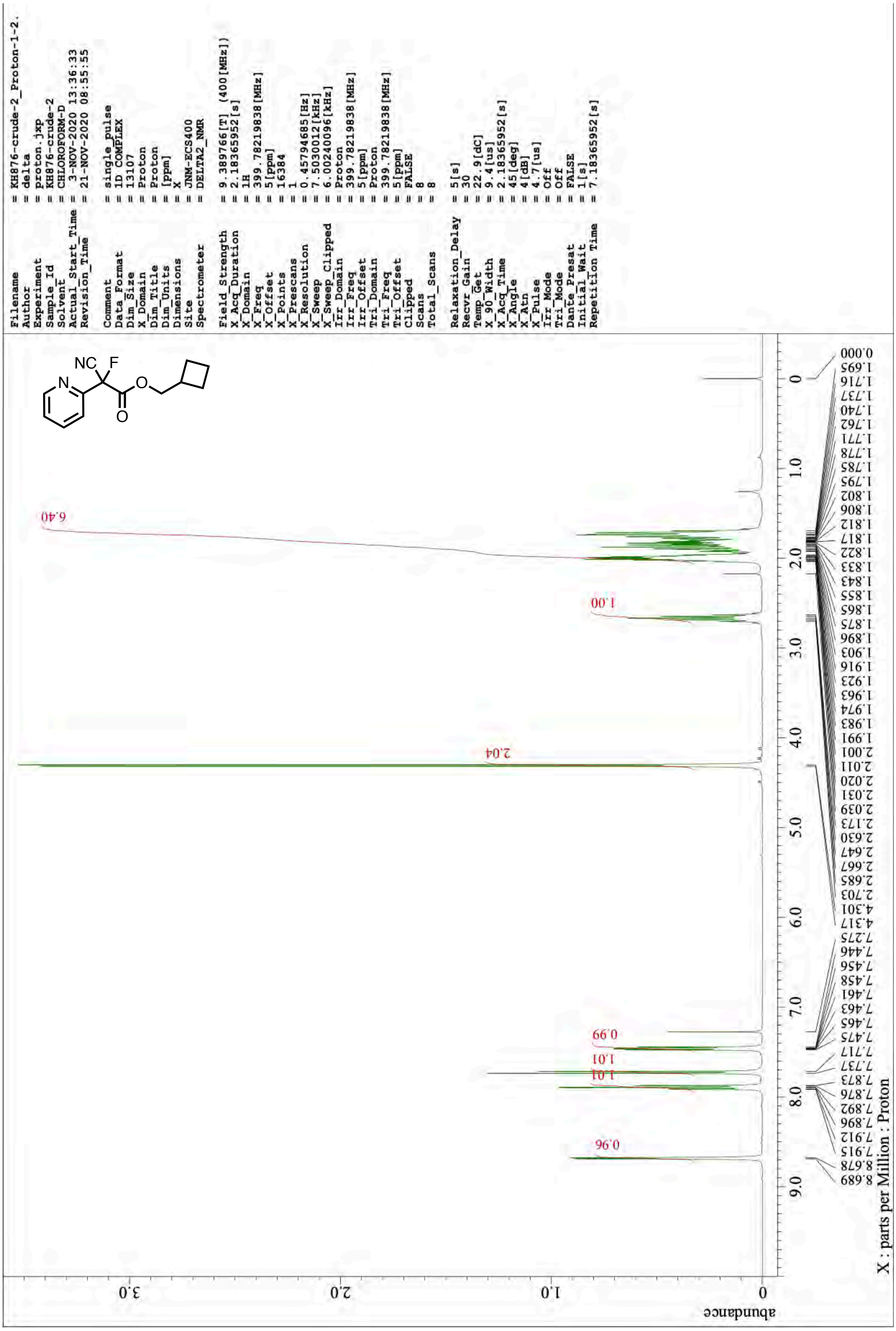

^{13}C NMR of **2T** (101 MHz, $CDCl_3$)

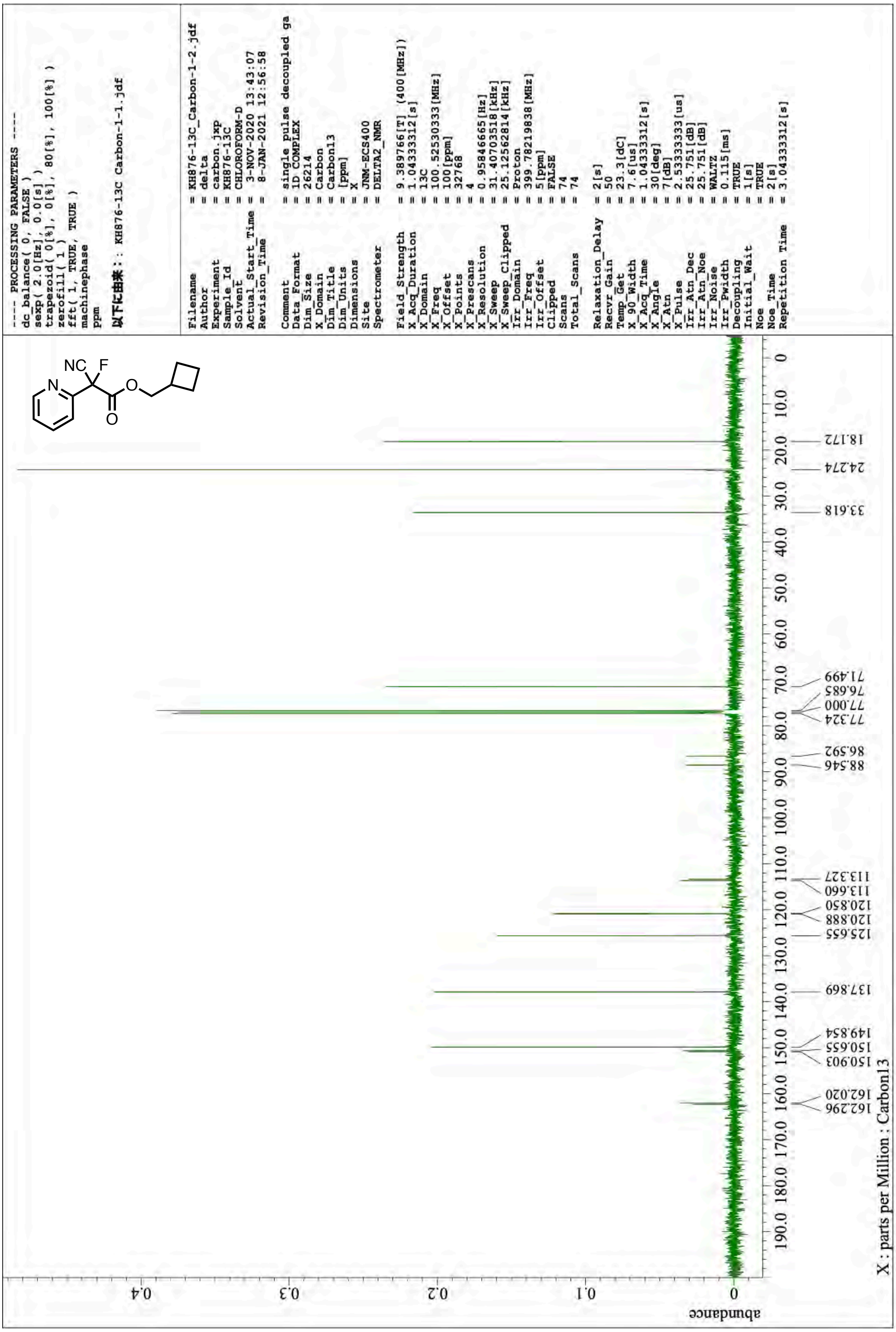

^{19}F NMR of **2T** (376 MHz, $CDCl_3$)

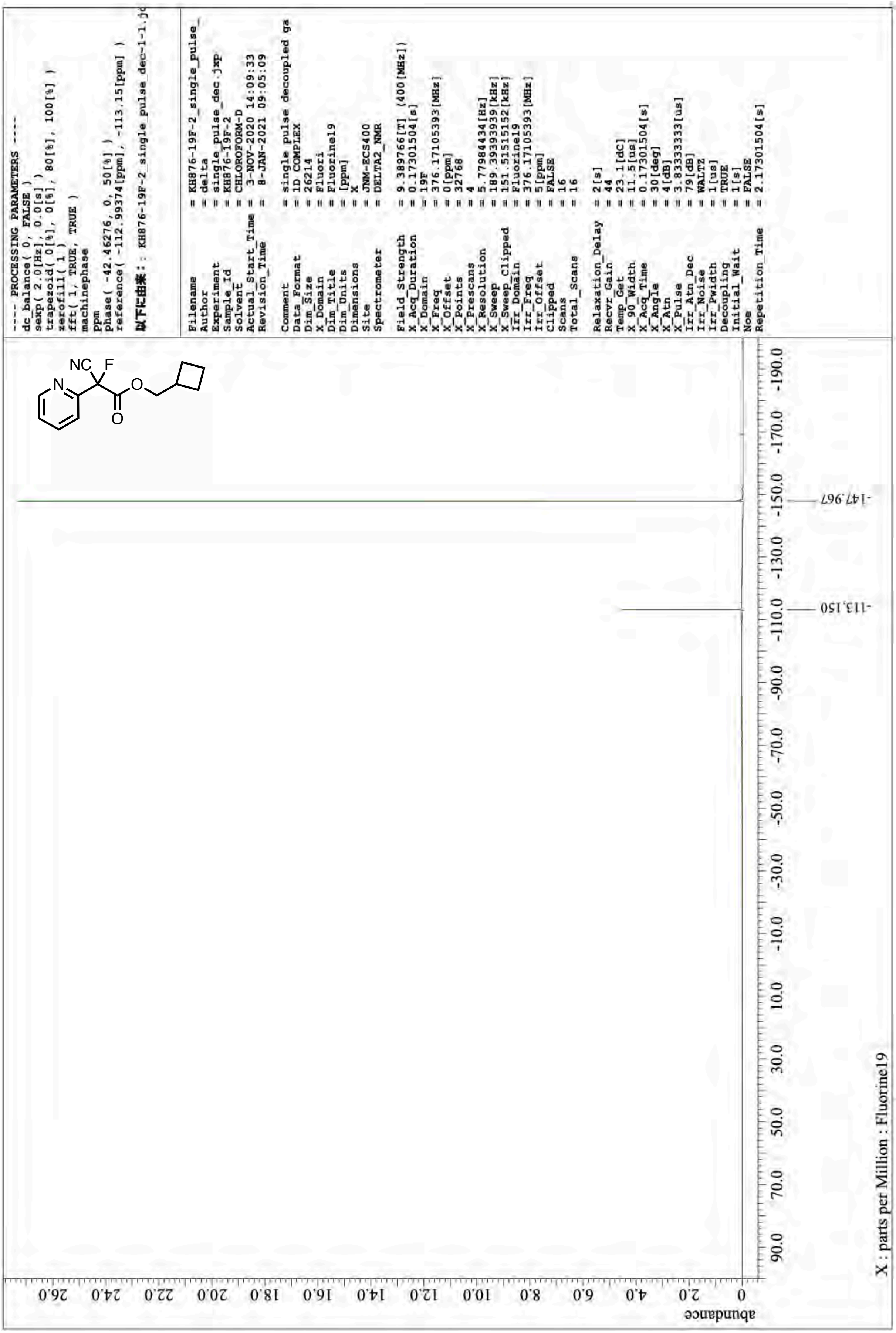

^{1}H NMR of **2U** (400 MHz, $CDCl_3$)

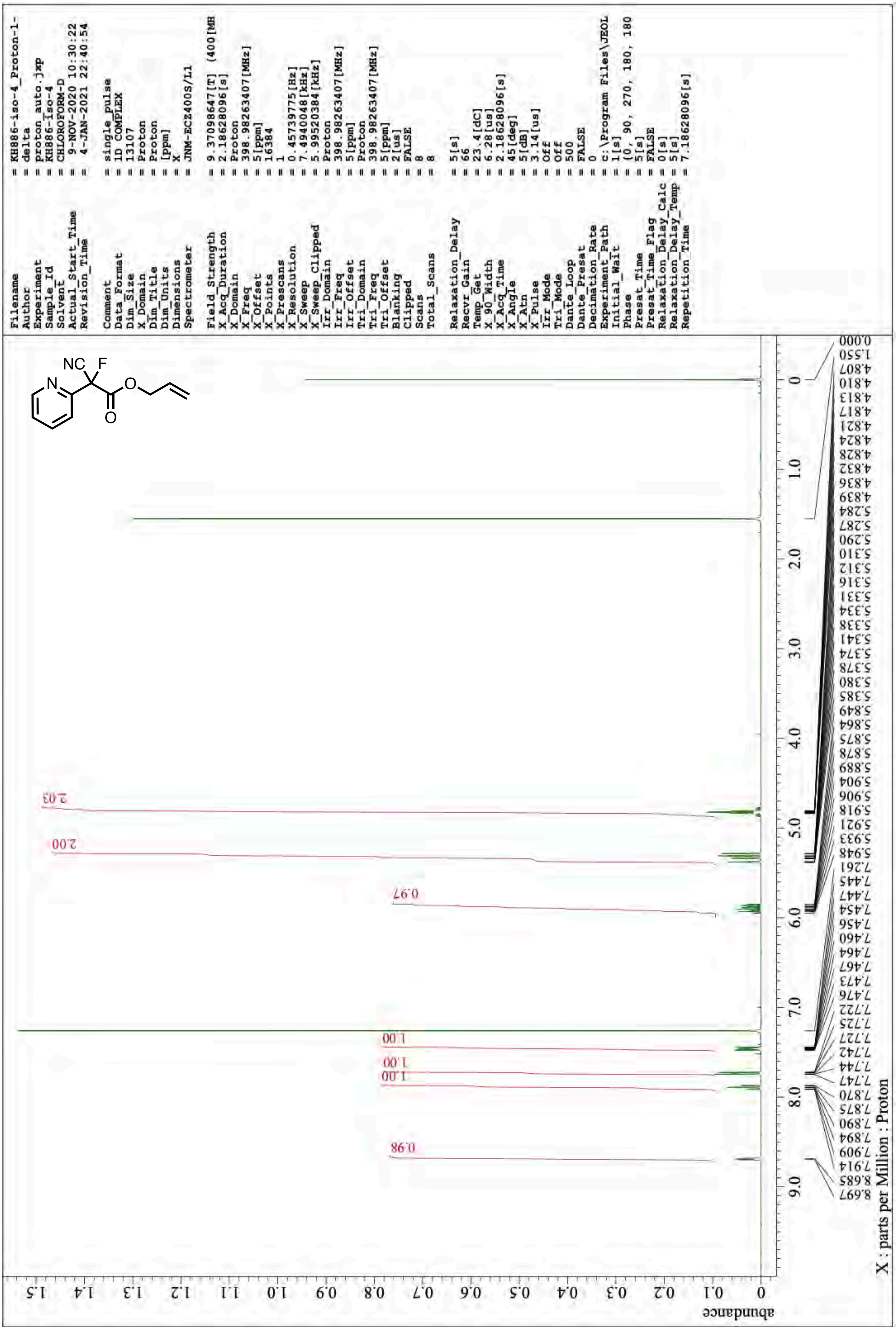

^{13}C NMR of **2U** (101 MHz, $CDCl_3$)

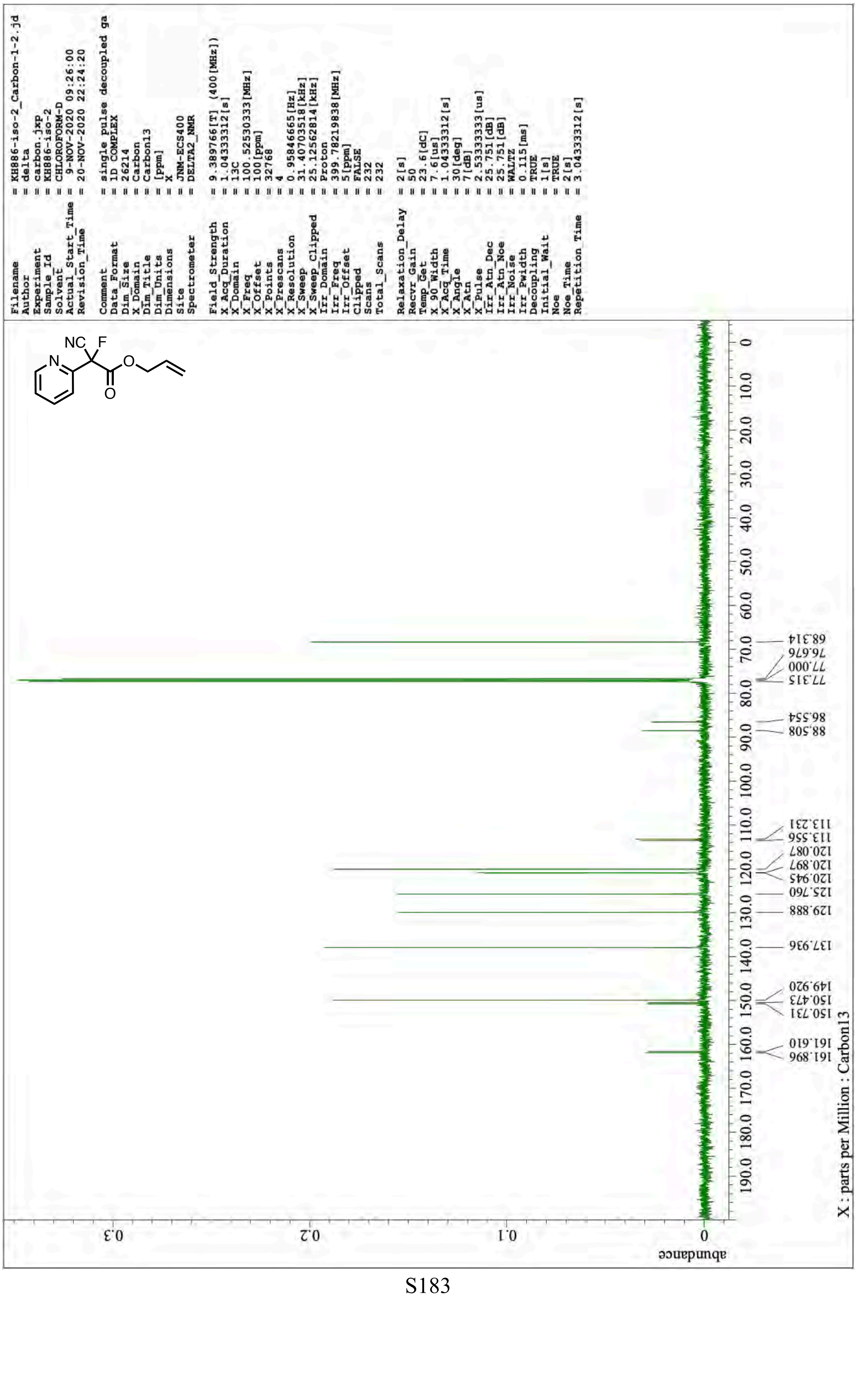

^{19}F NMR of **2U** (376 MHz, $CDCl_3$)

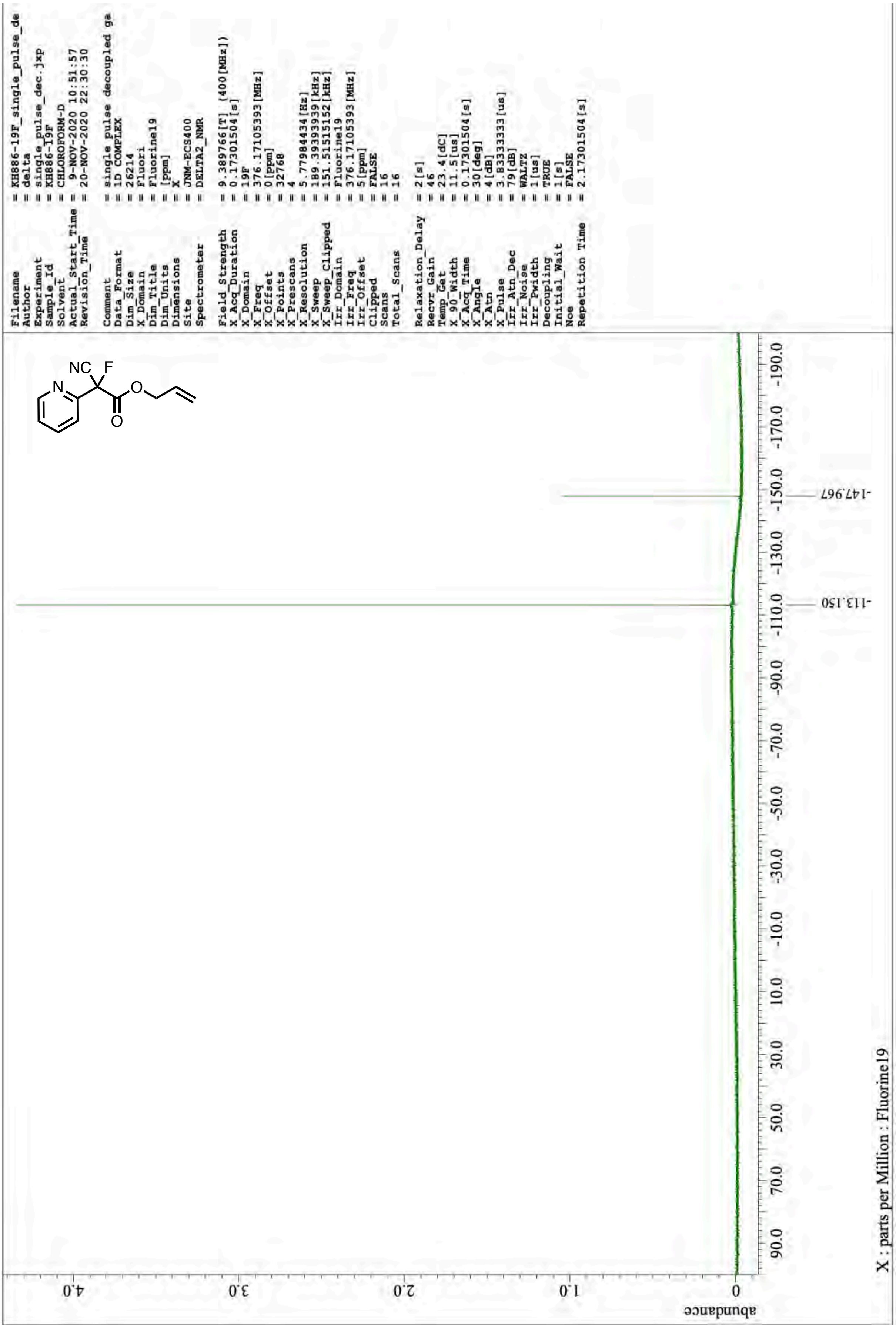

^{1}H NMR of **2V** (400 MHz, $CDCl_3$)

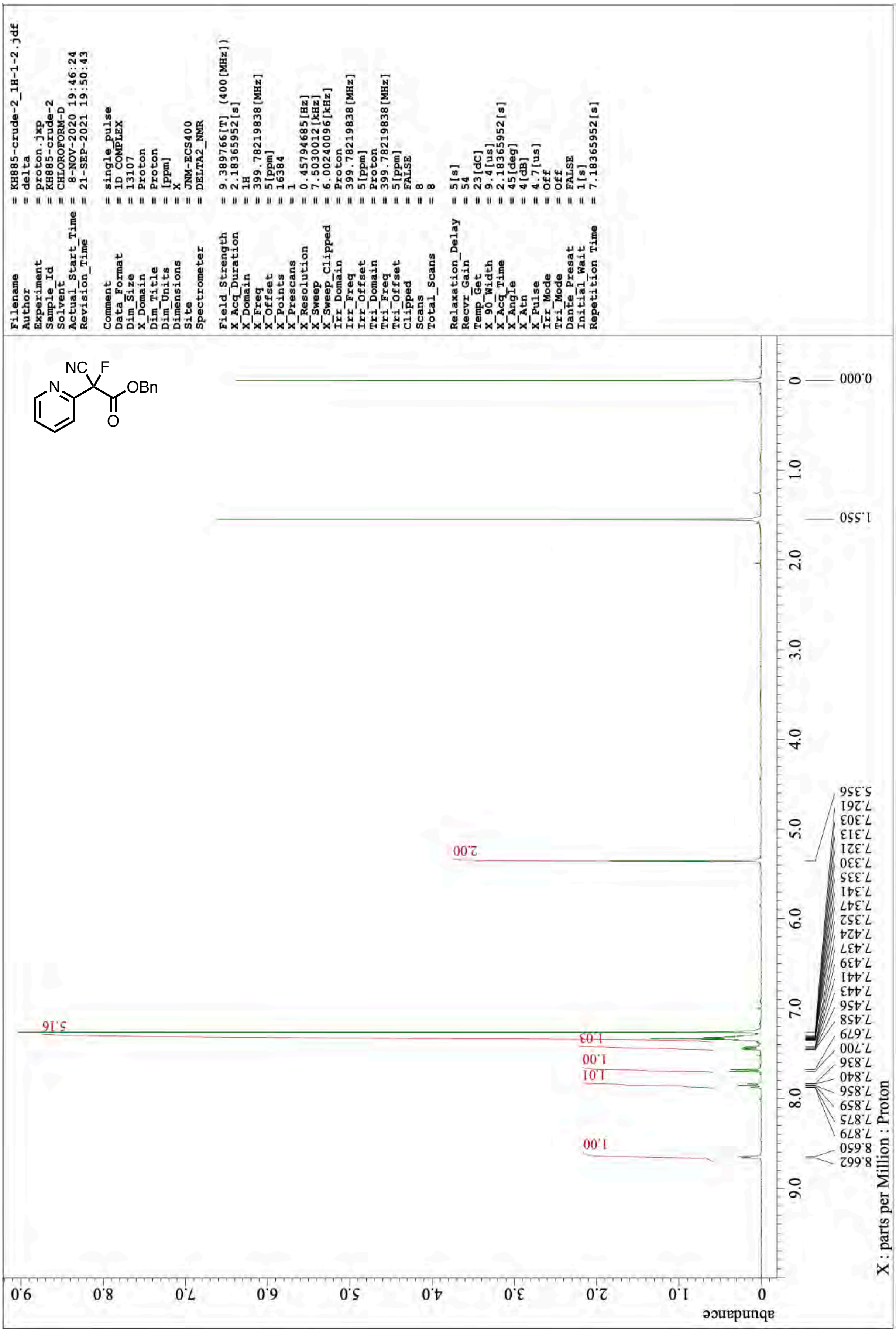

^{13}C NMR of **2V** (101 MHz, $CDCl_3$)

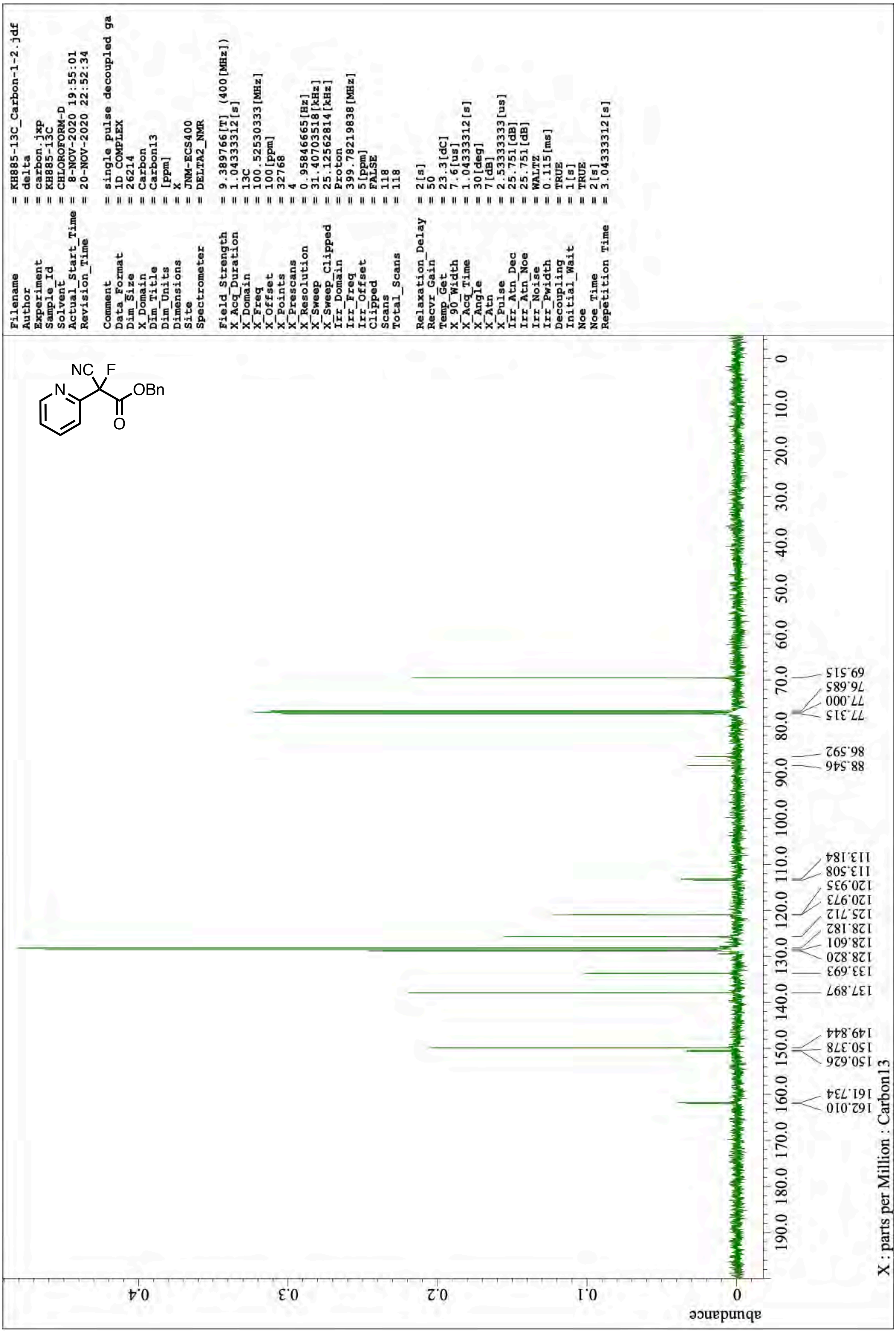

^{19}F NMR of **2V** (376 MHz, $CDCl_3$)

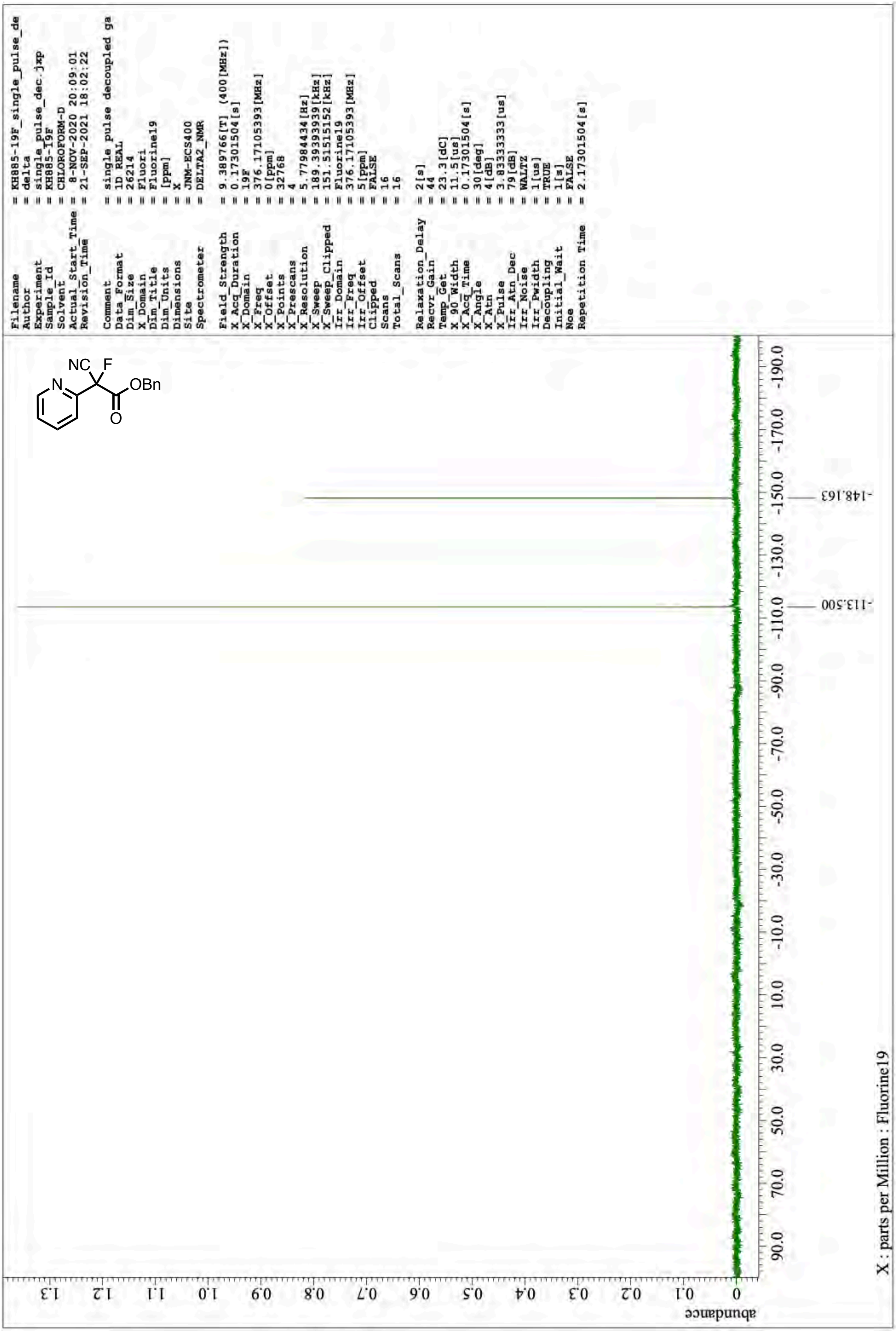

^{1}H NMR of **2W** (400 MHz, $CDCl_3$)

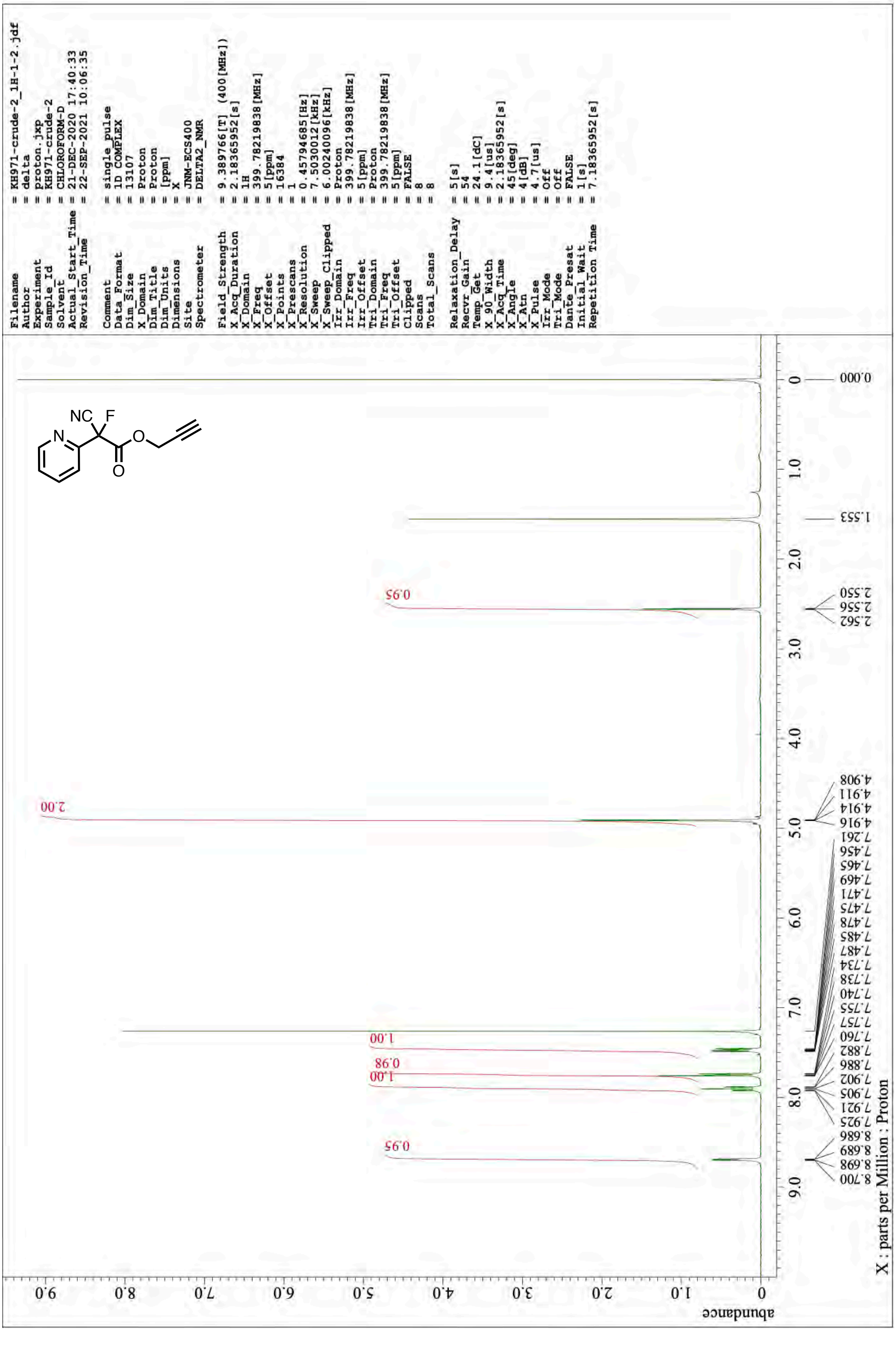

^{13}C NMR of **2W** (101 MHz, $CDCl_3$)

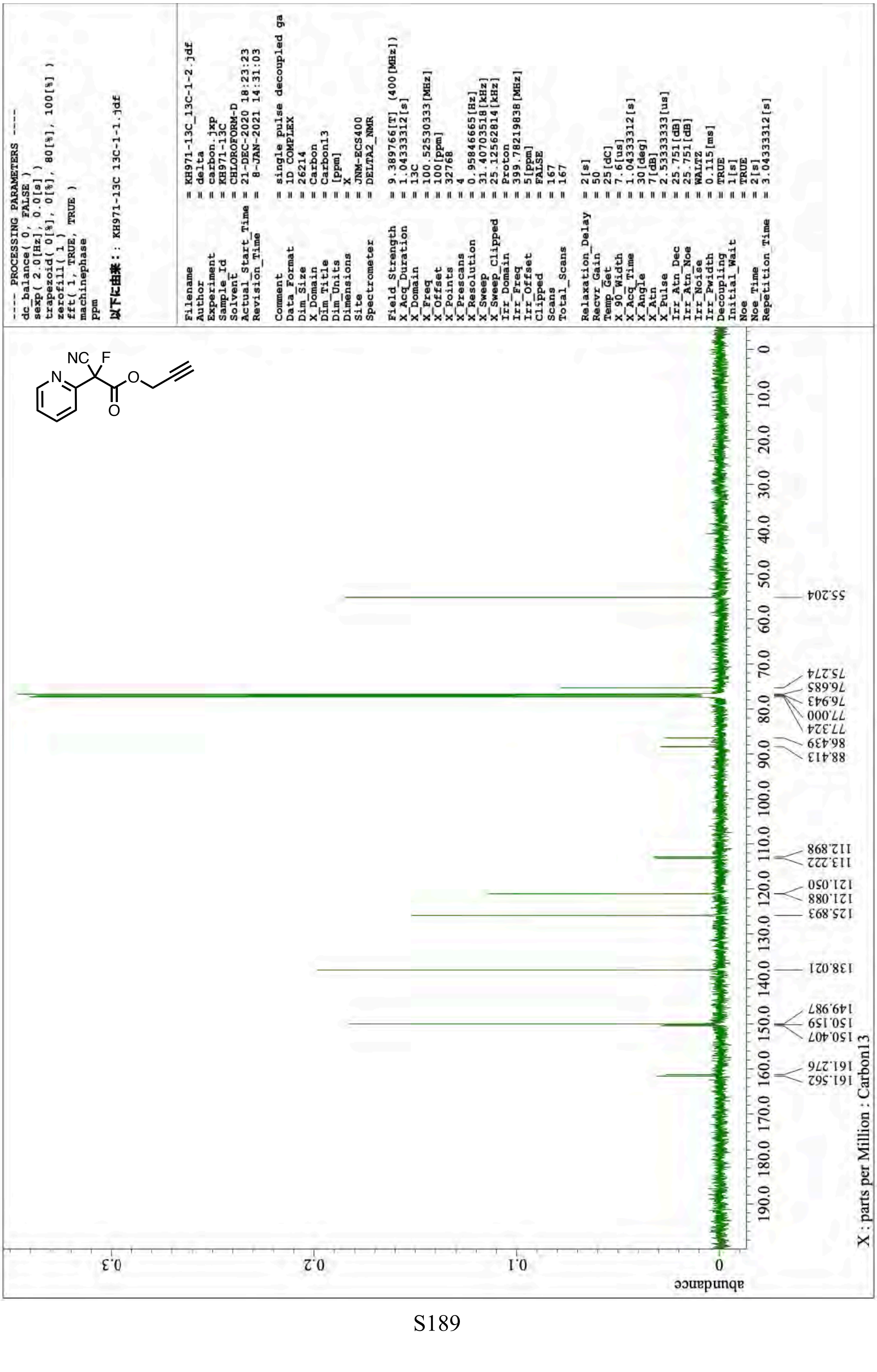

^{19}F NMR of **2W** (376 MHz, $CDCl_3$)

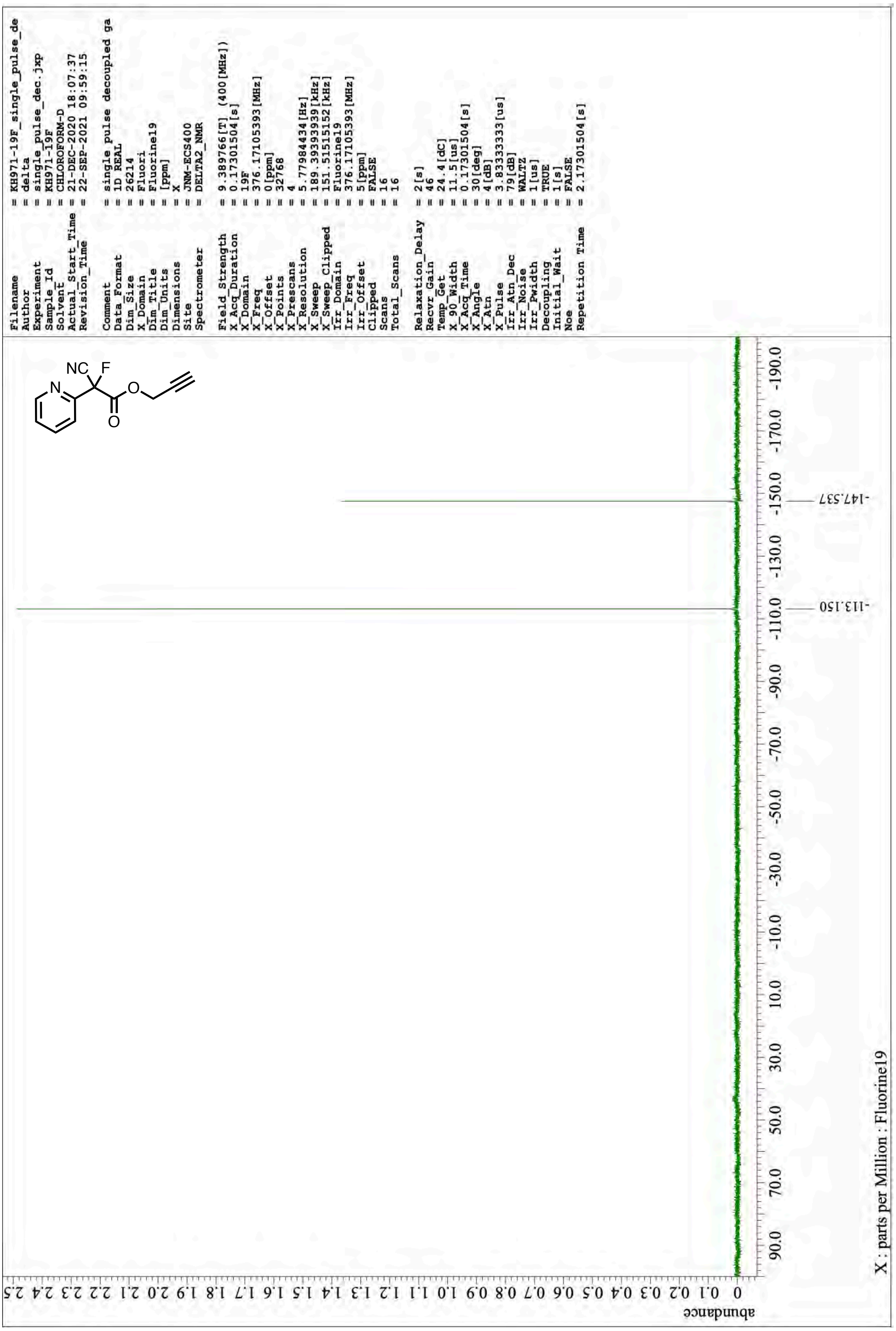

^{1}H NMR of **2X** (400 MHz, $CDCl_3$)

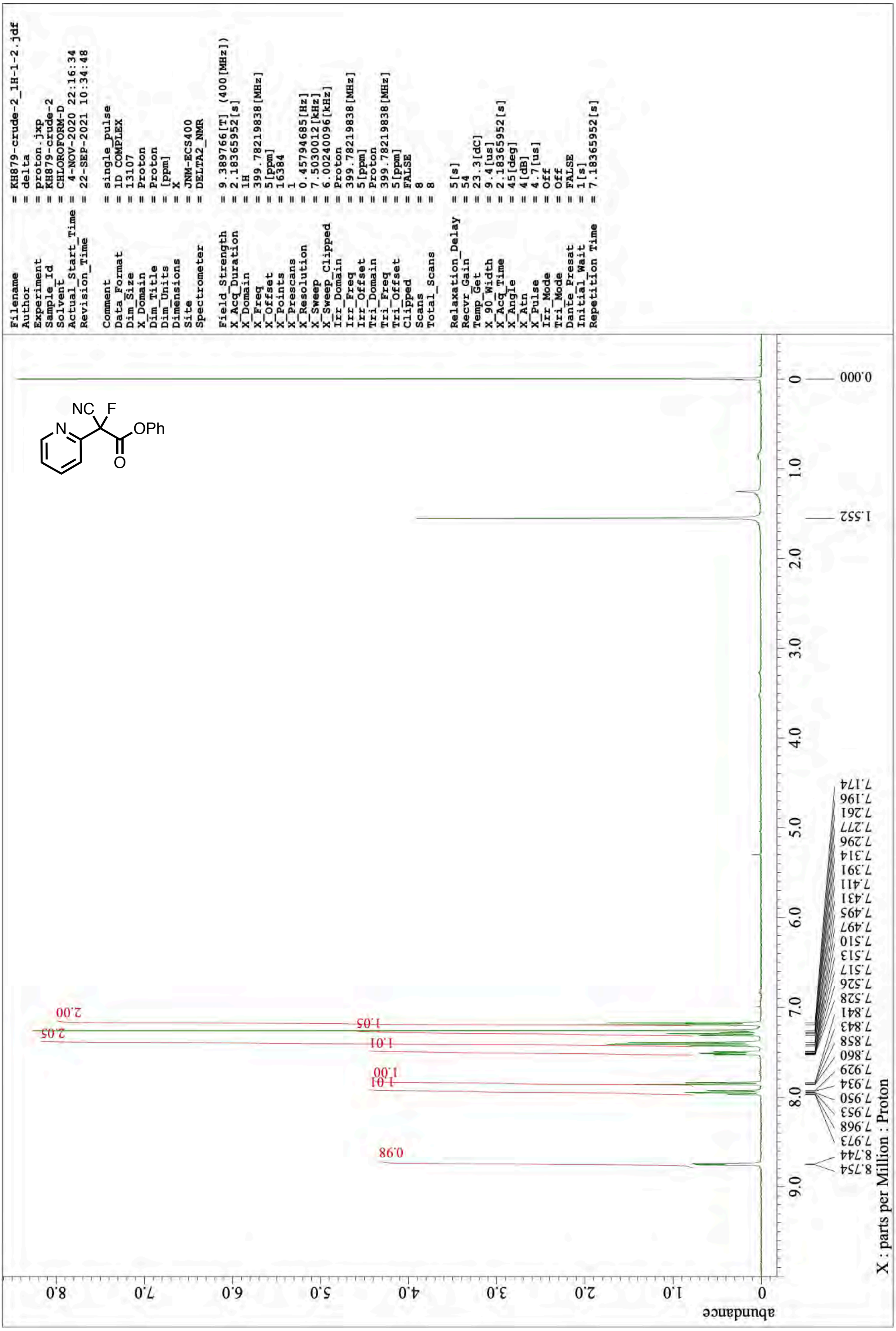

^{13}C NMR of **2X** (101 MHz, $CDCl_3$)

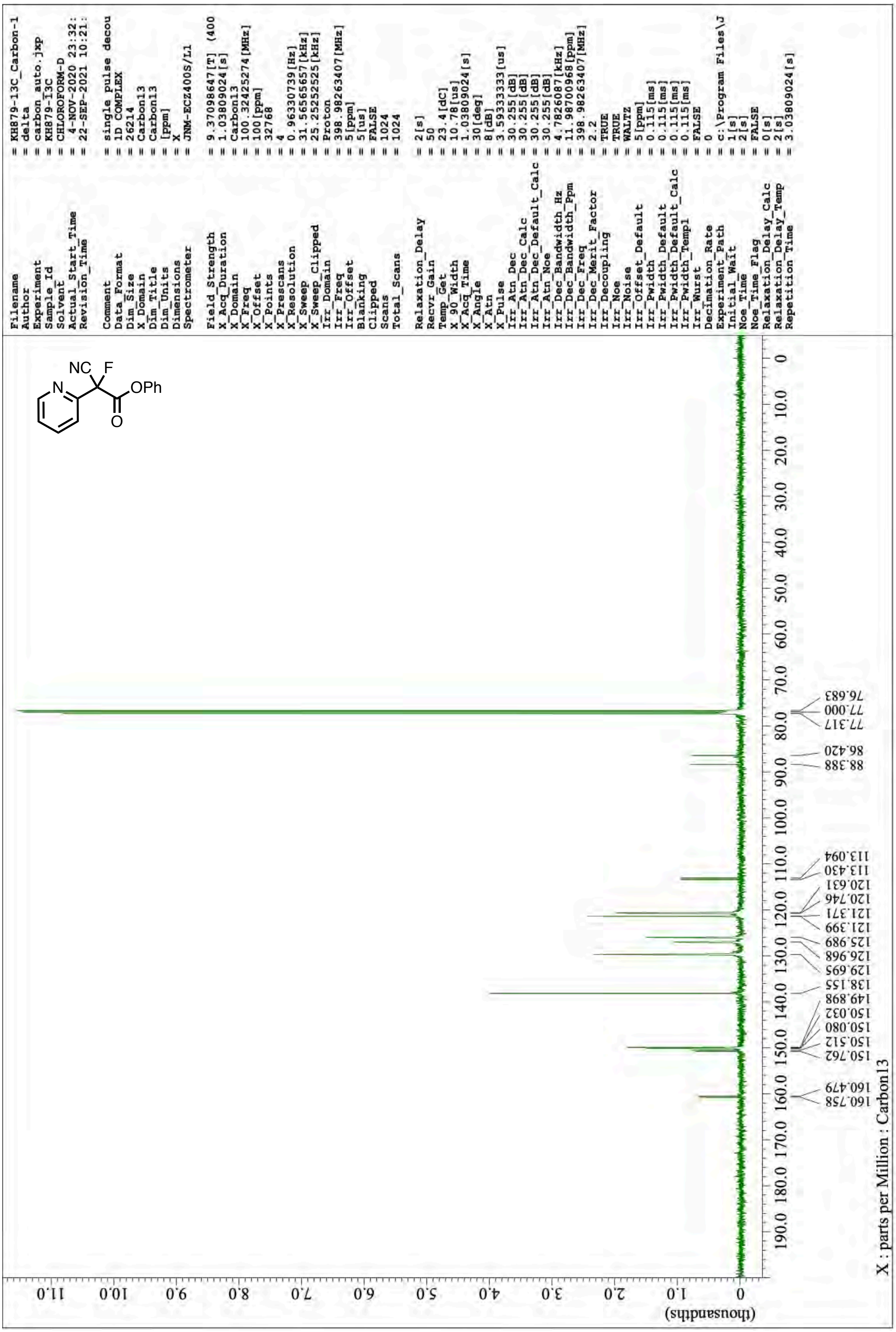

^{19}F NMR of **2X** (376 MHz, $CDCl_3$)

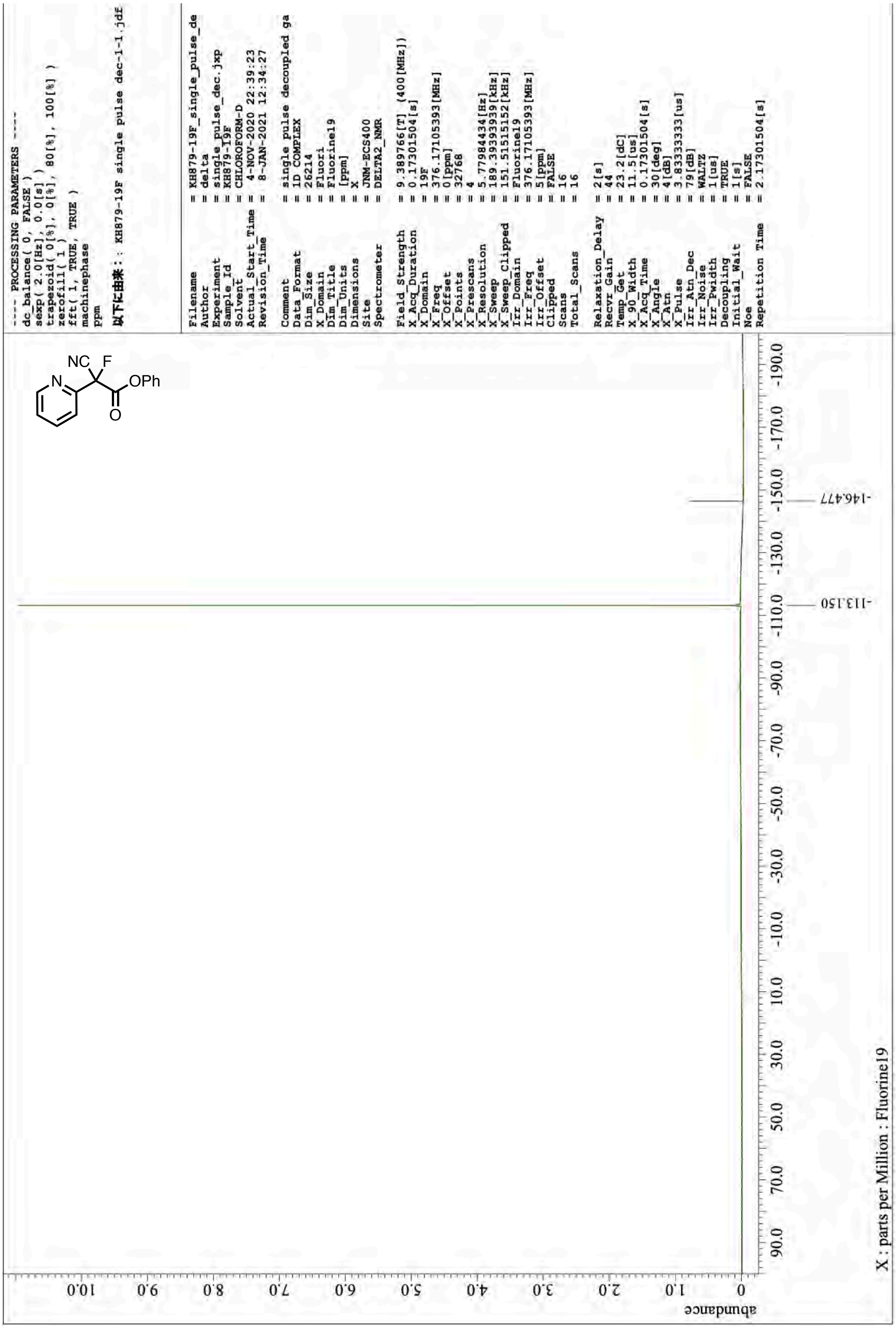

^{1}H NMR of **2Z** (400 MHz, $CDCl_3$)

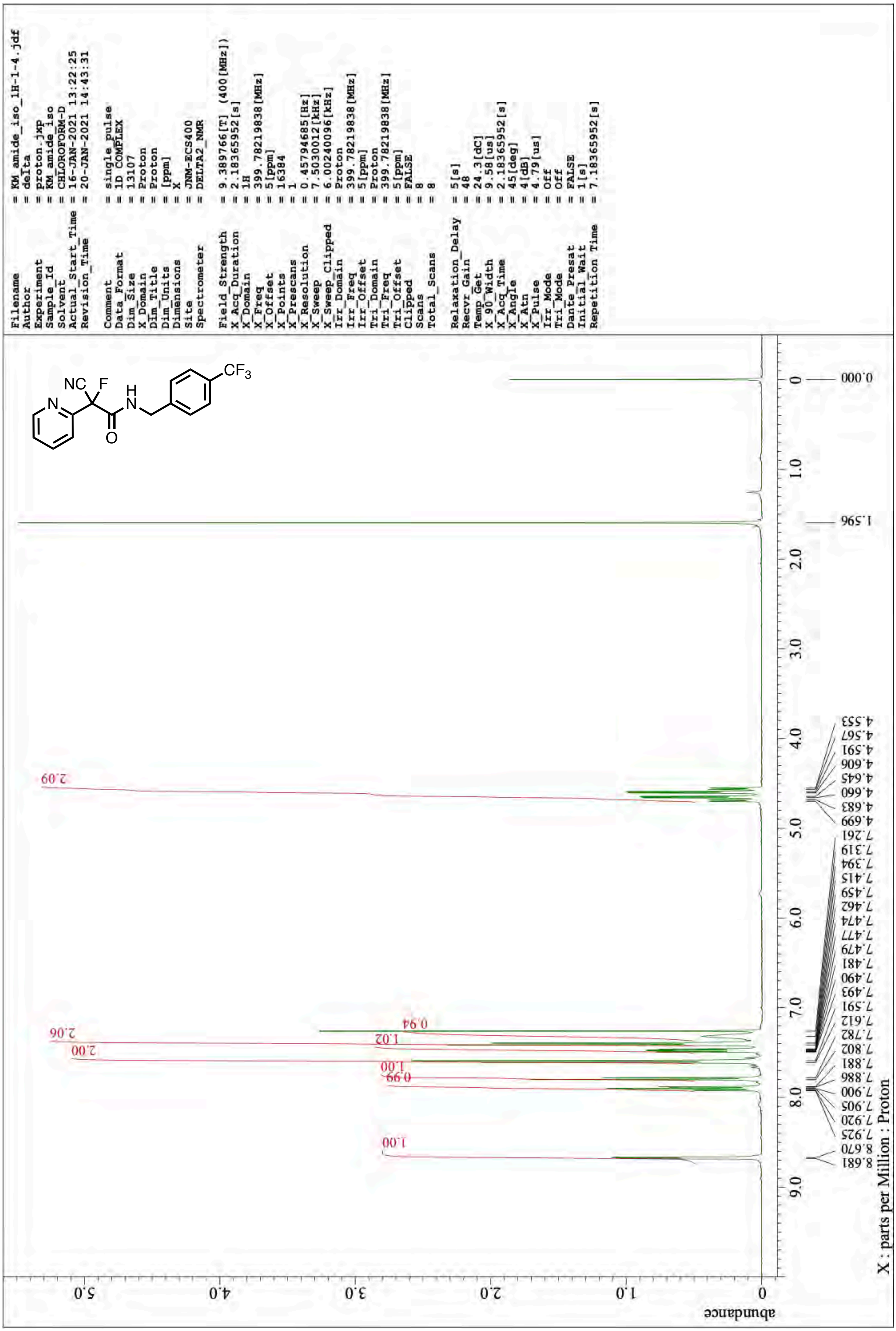

^{13}C NMR of **2Z** (101 MHz, $CDCl_3$)

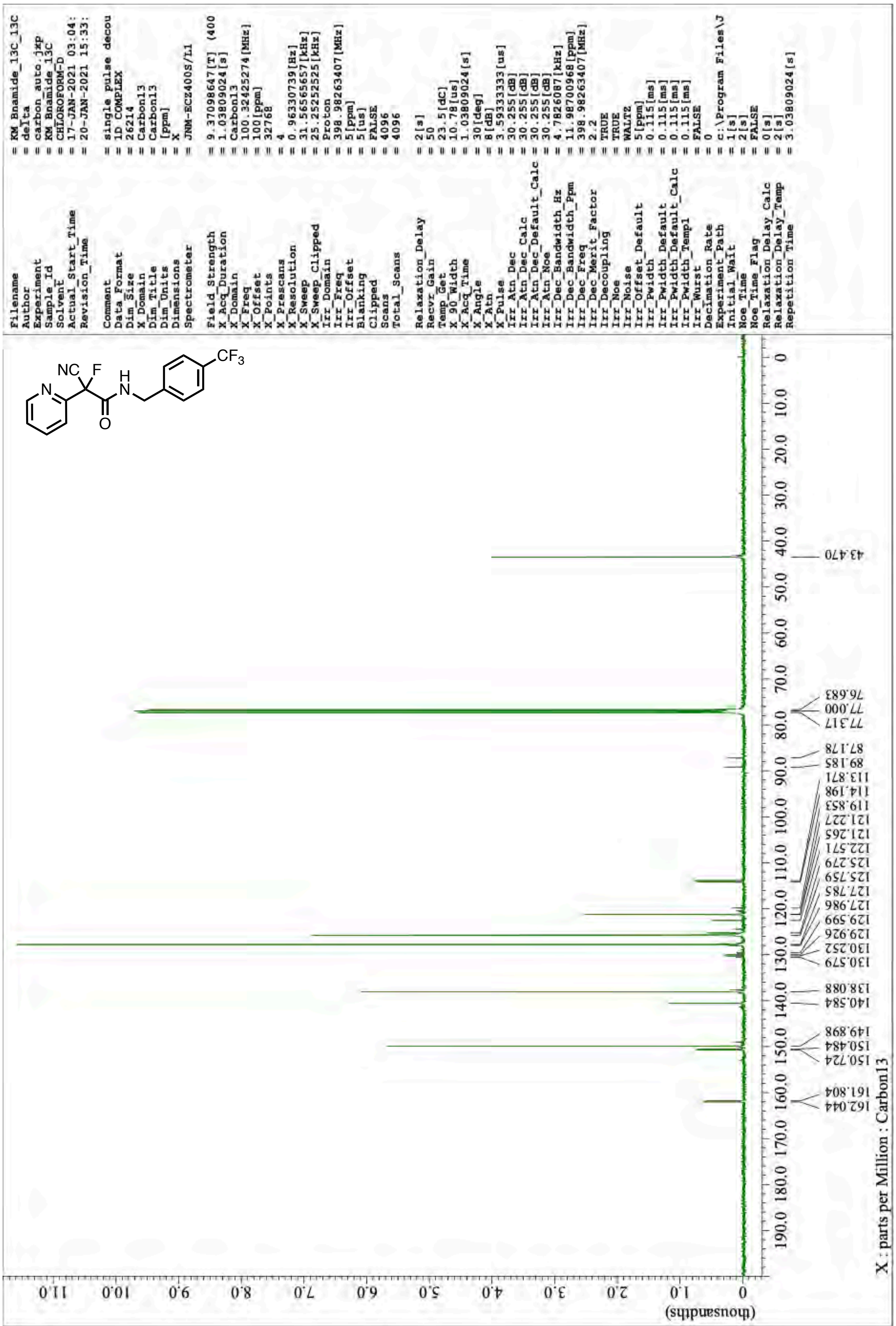

^{19}F NMR of **2Z** (376 MHz, $CDCl_3$)

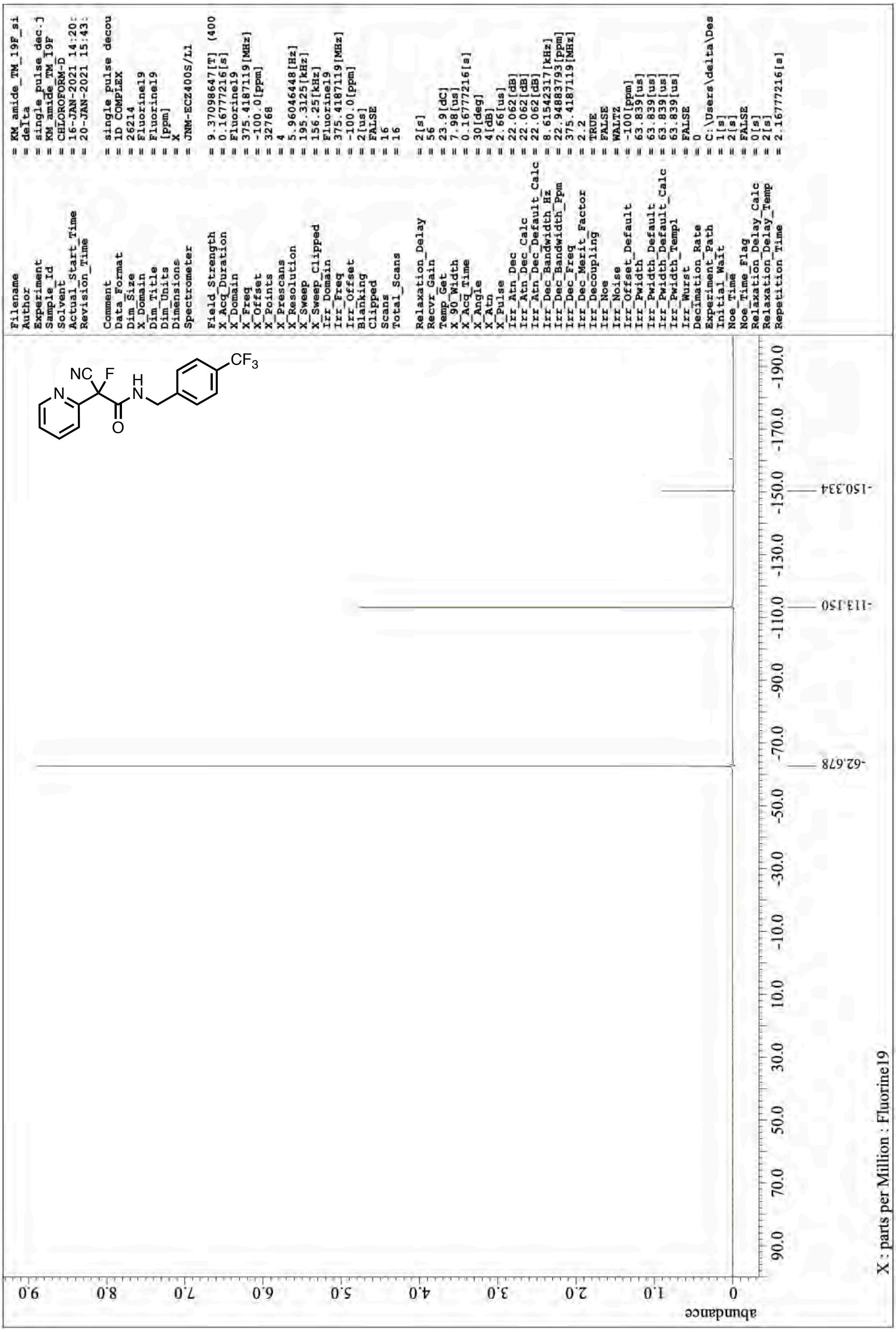

^{1}H NMR of **2Z’** (400 MHz, $CDCl_3$)

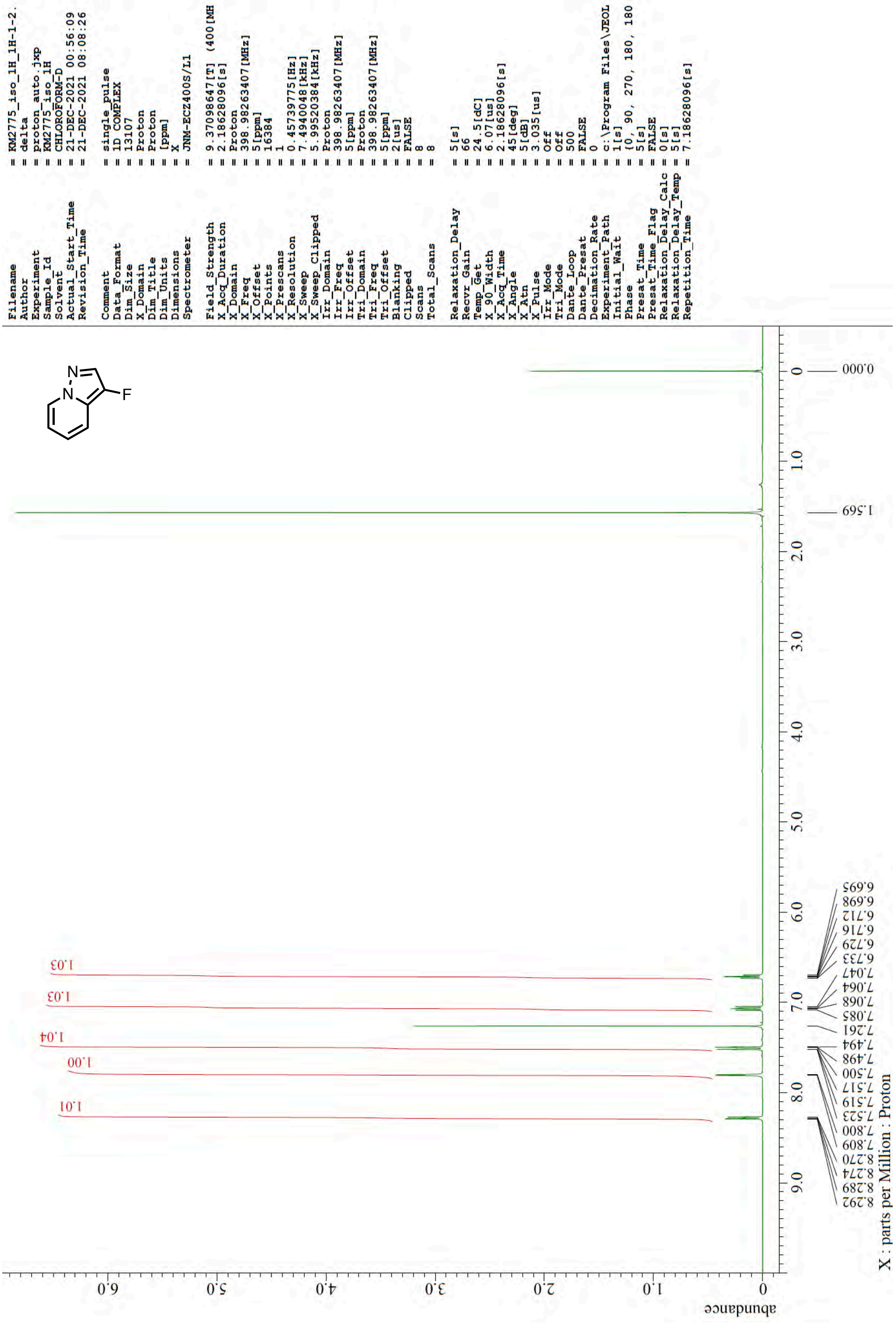

^{13}C NMR of **2Z’** (101 MHz, $CDCl_3$)

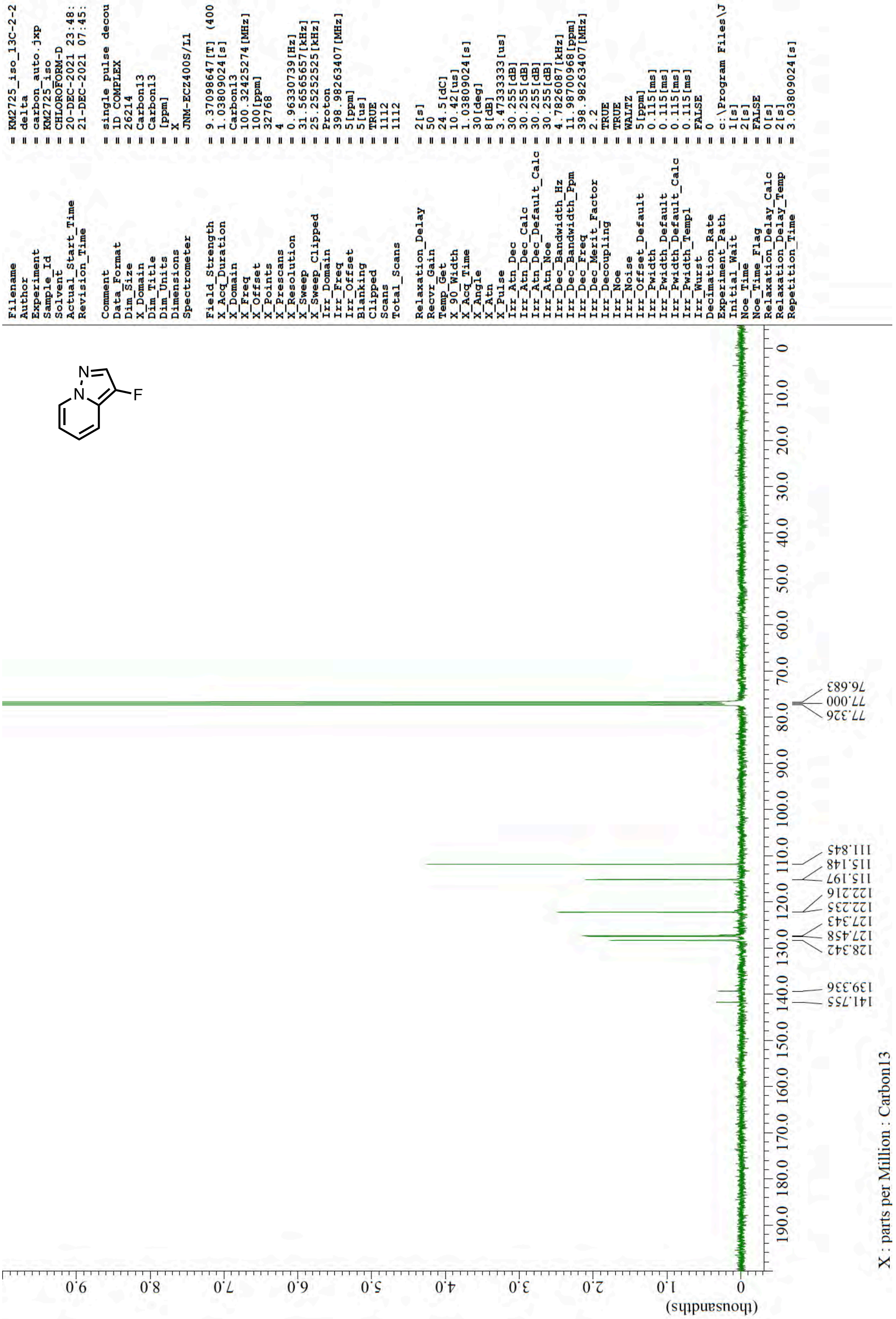

^{19}F NMR of **2Z’** (376 MHz, $CDCl_3$)

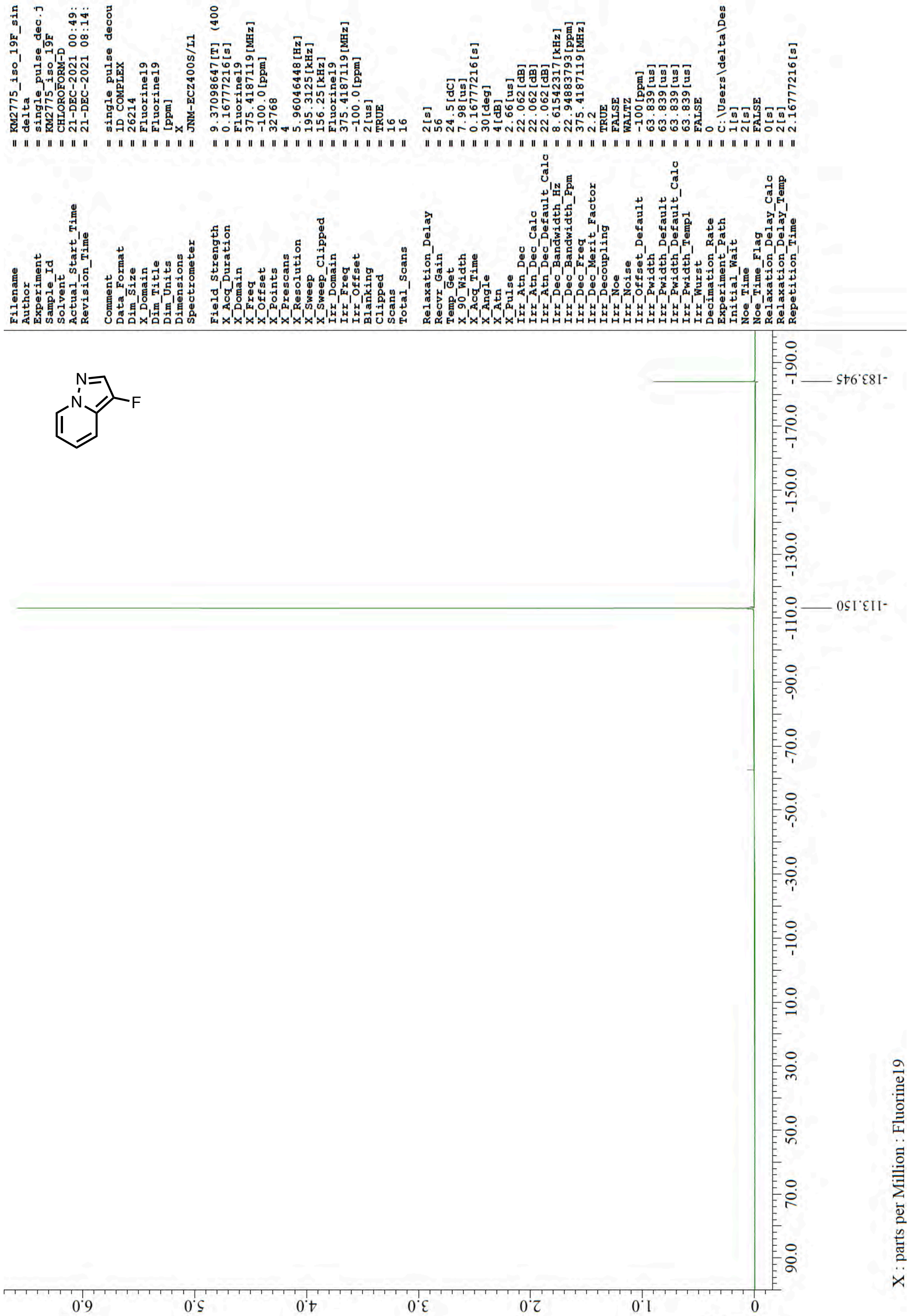

^{1}H NMR of **2AA** (400 MHz, $CDCl_3$)

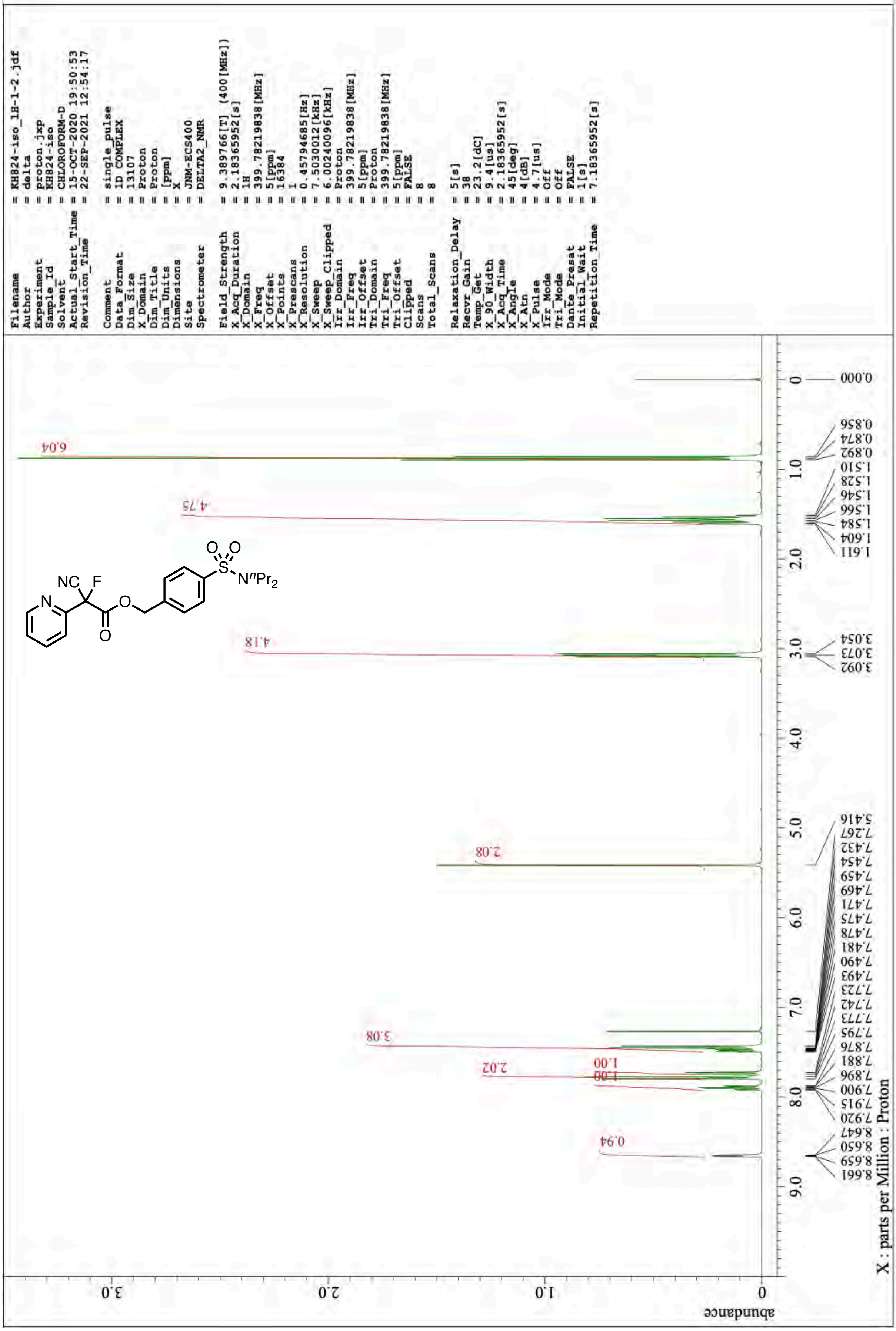

^{13}C NMR of **2AA** (101 MHz, $CDCl_3$)

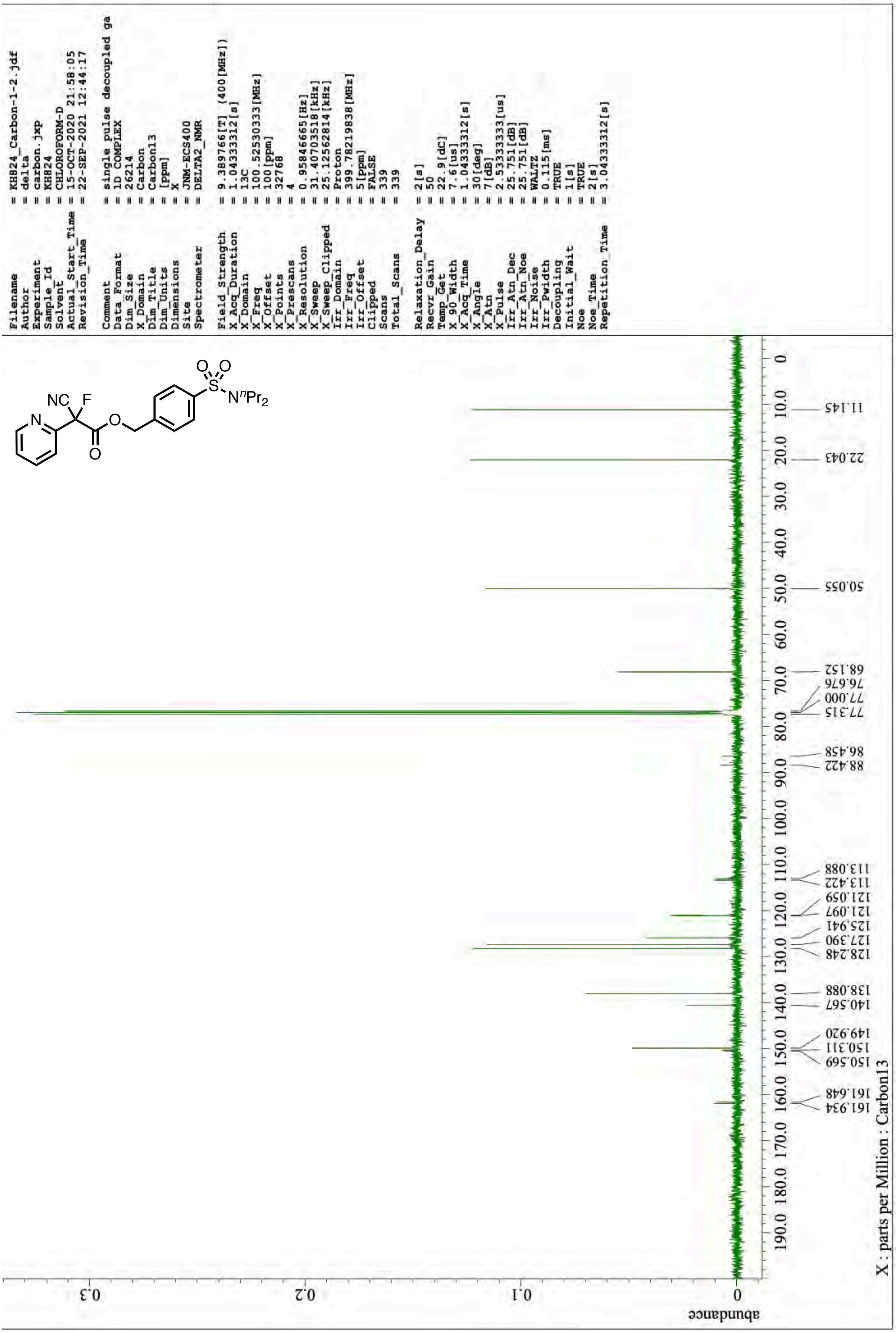

^{19}F NMR of **2AA** (376 MHz, $CDCl_3$)

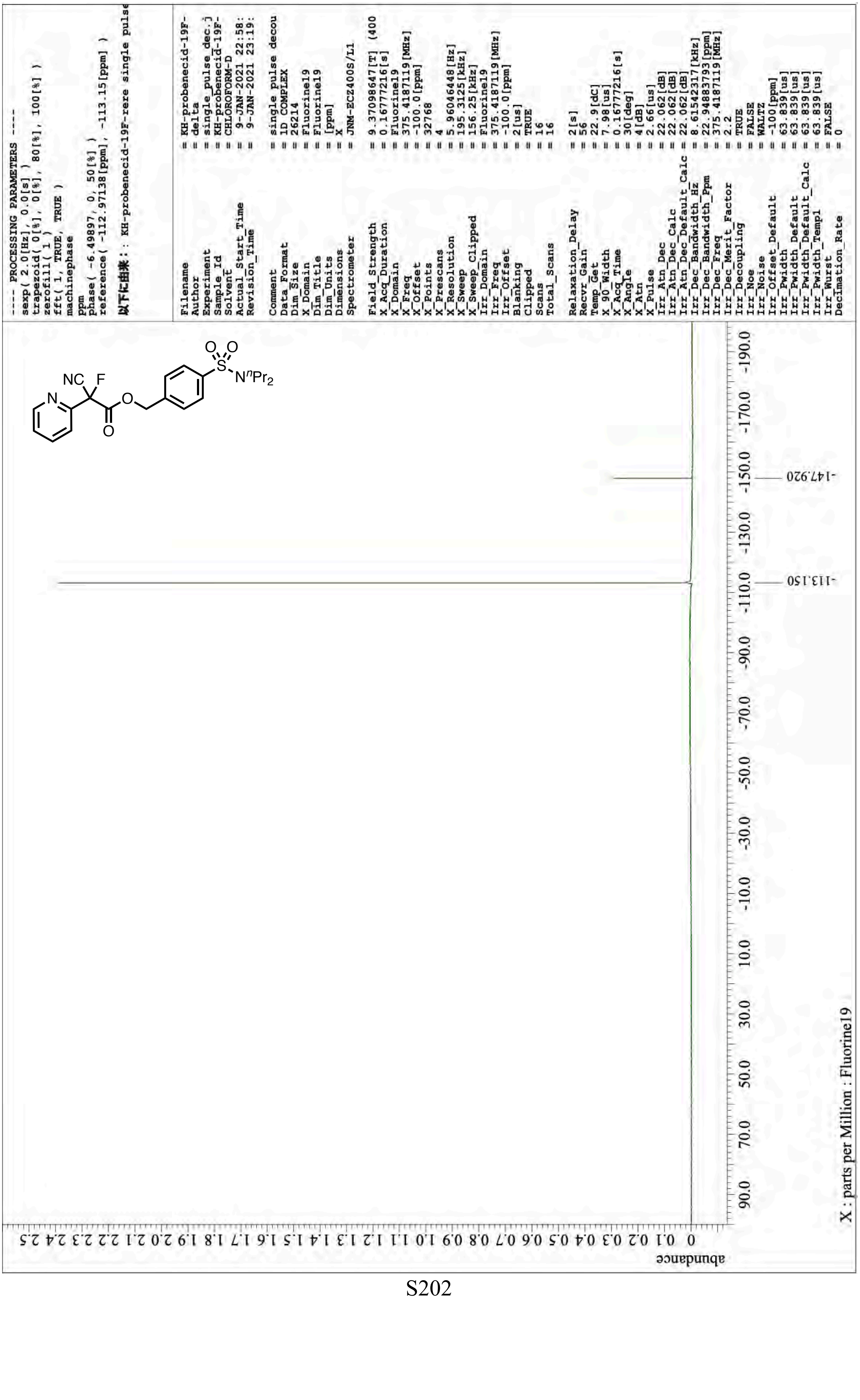

^{1}H NMR of **2AB** (400 MHz, $CDCl_3$)

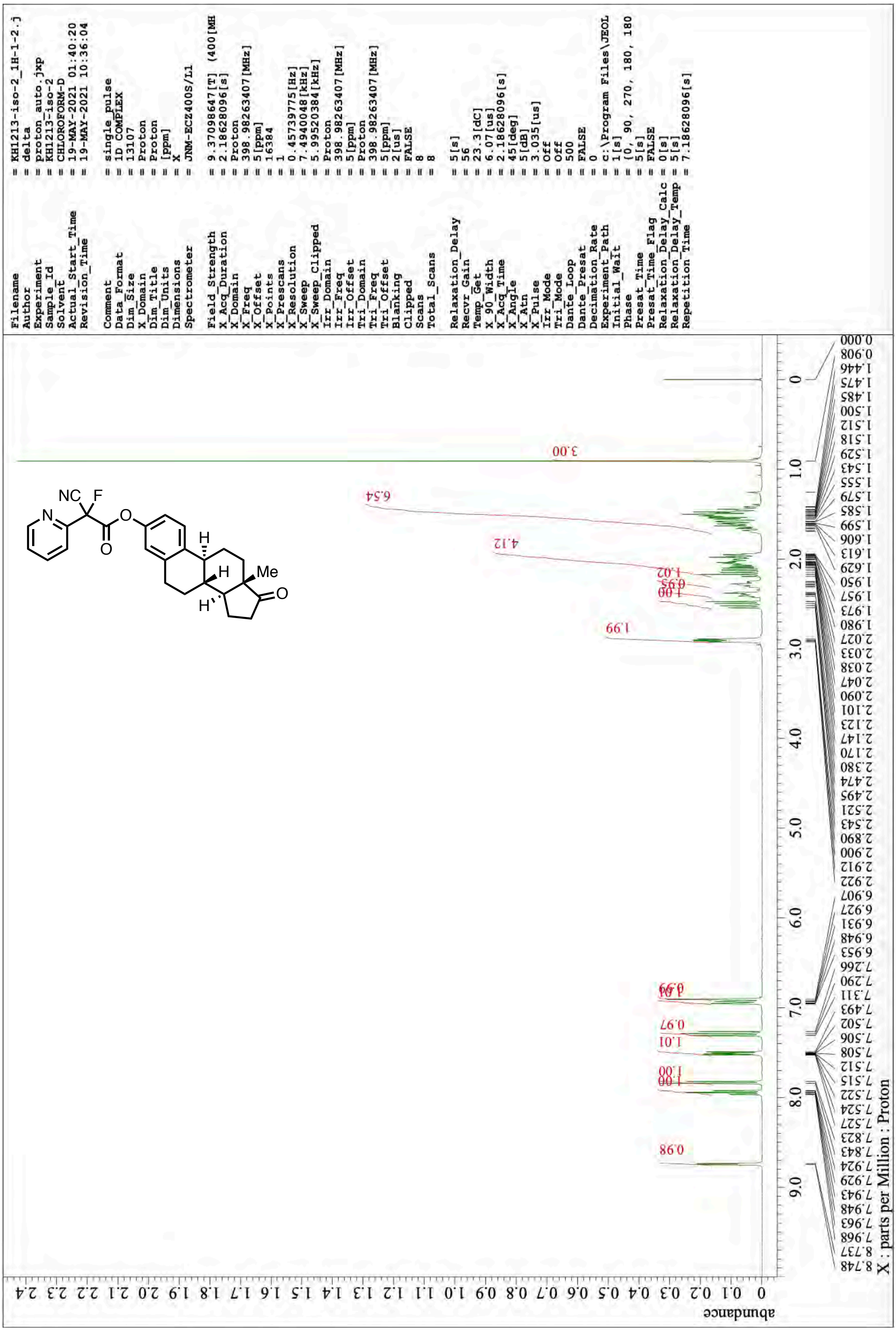

^{13}C NMR of **2AB** (101 MHz, $CDCl_3$)

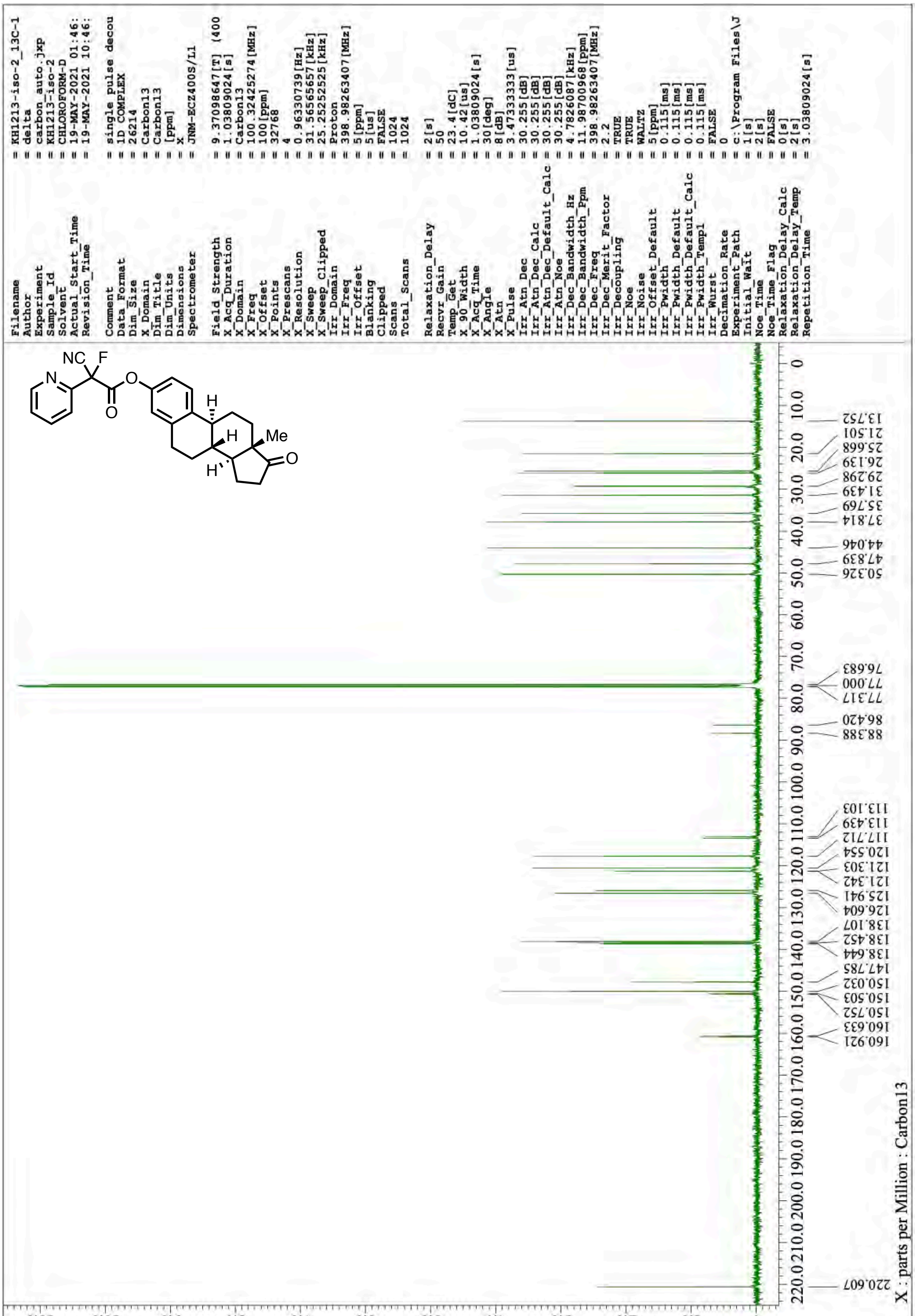

^{19}F NMR of **2AB** (376 MHz, $CDCl_3$)

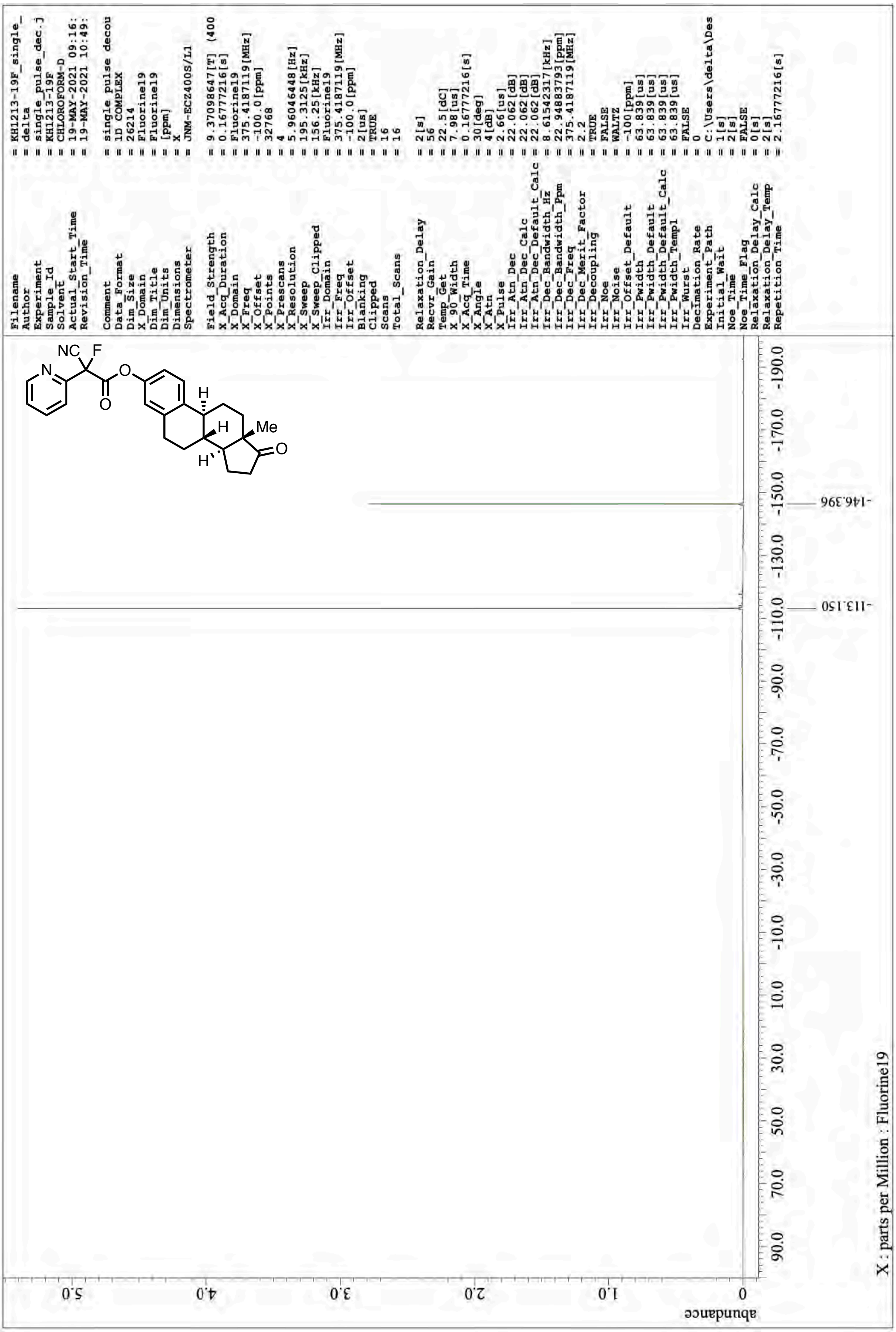

^{1}H NMR of **2AC** (400 MHz, $CDCl_3$)

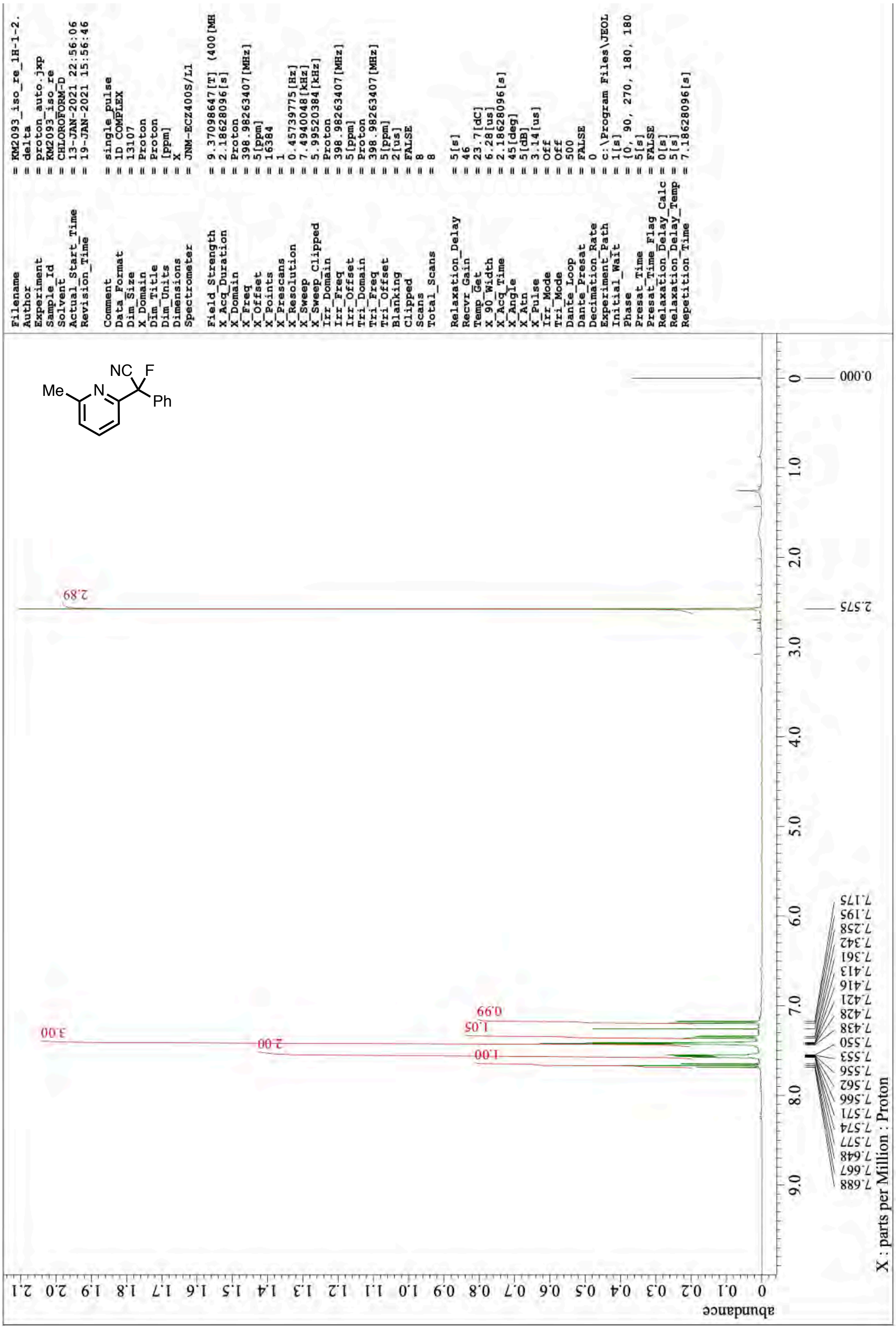

^{13}C NMR of **2AC** (101 MHz, $CDCl_3$)

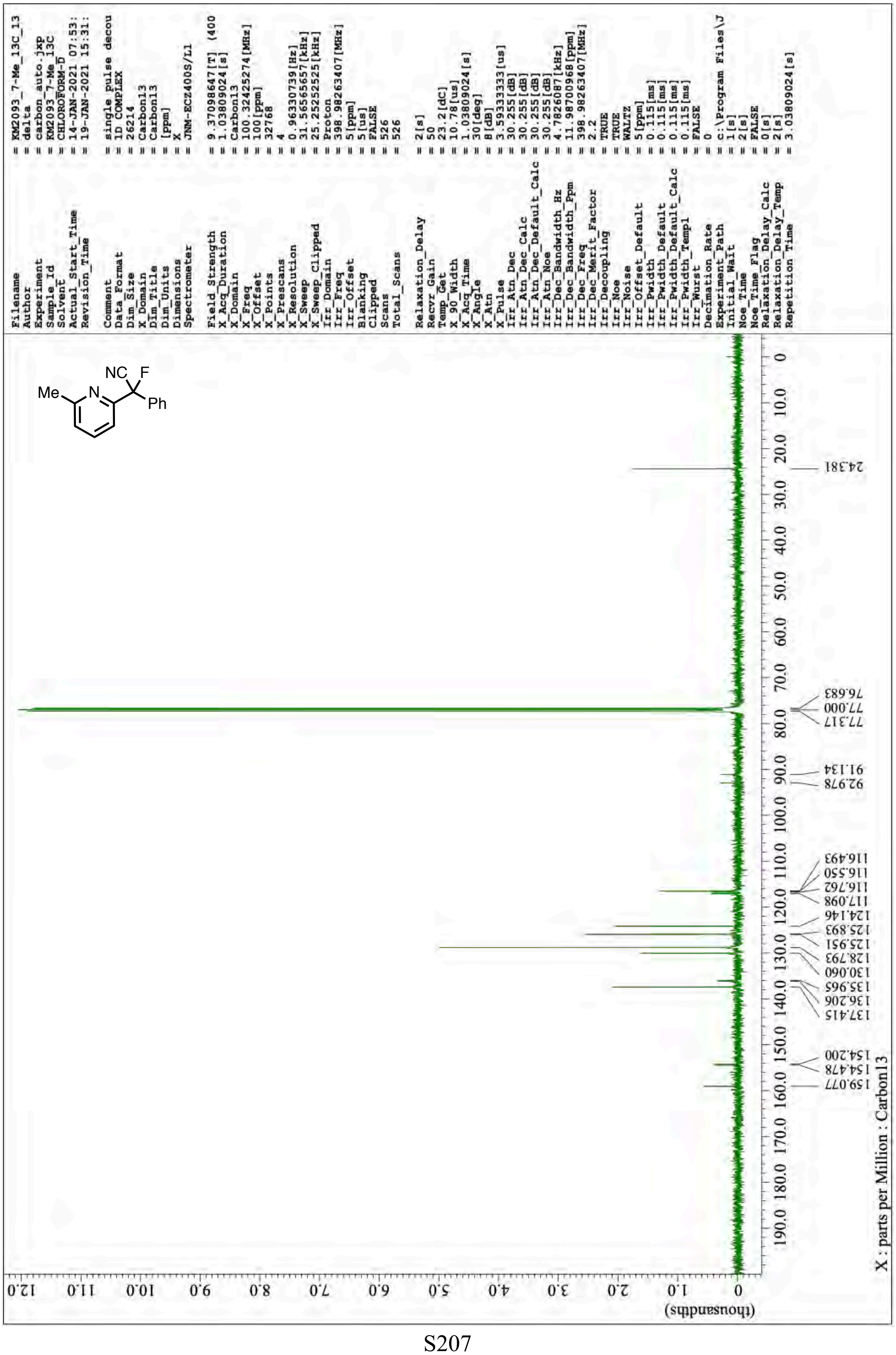

^{19}F NMR of **2AC** (376 MHz, $CDCl_3$)

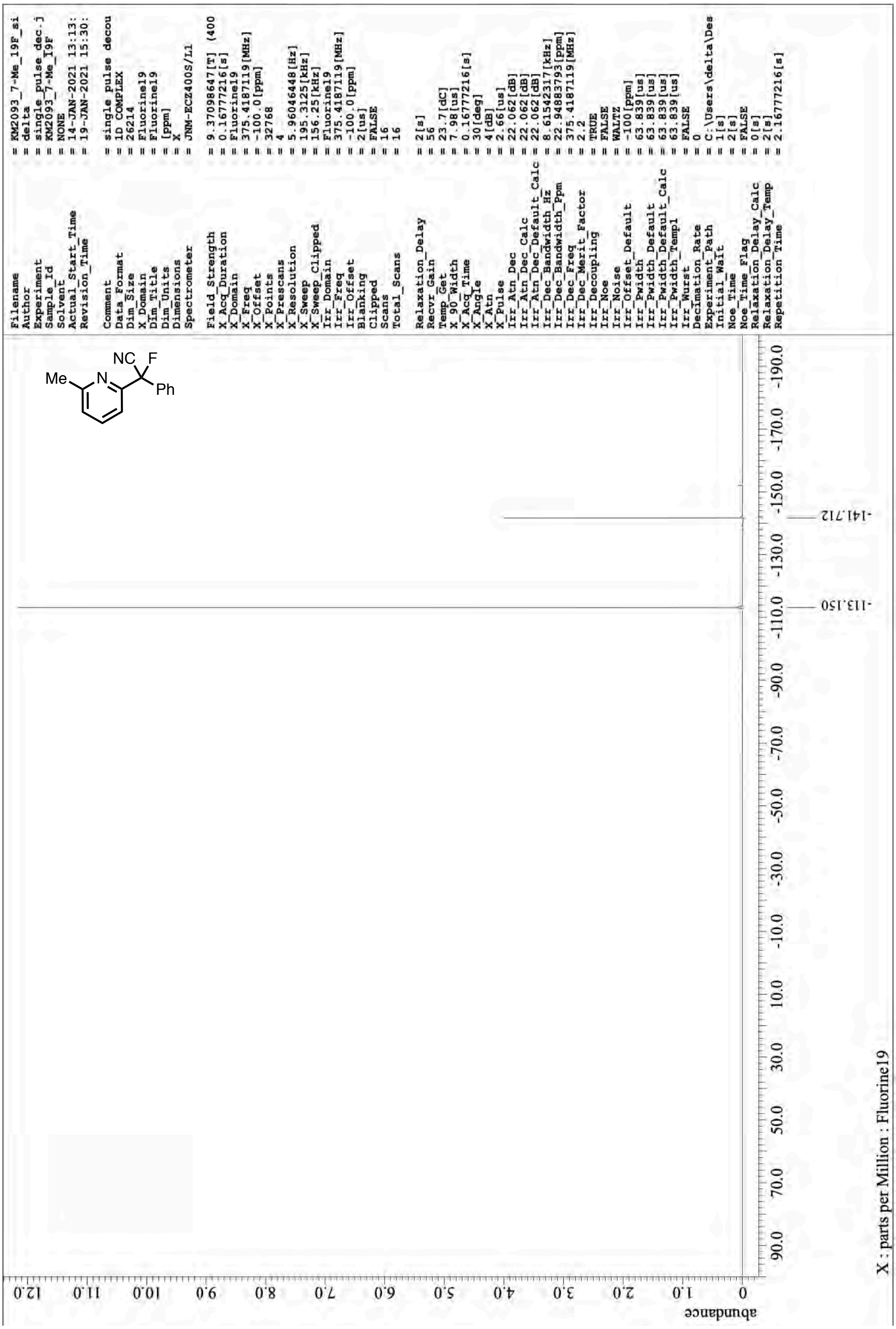

^{1}H NMR of **2AD** (400 MHz, $CDCl_3$)

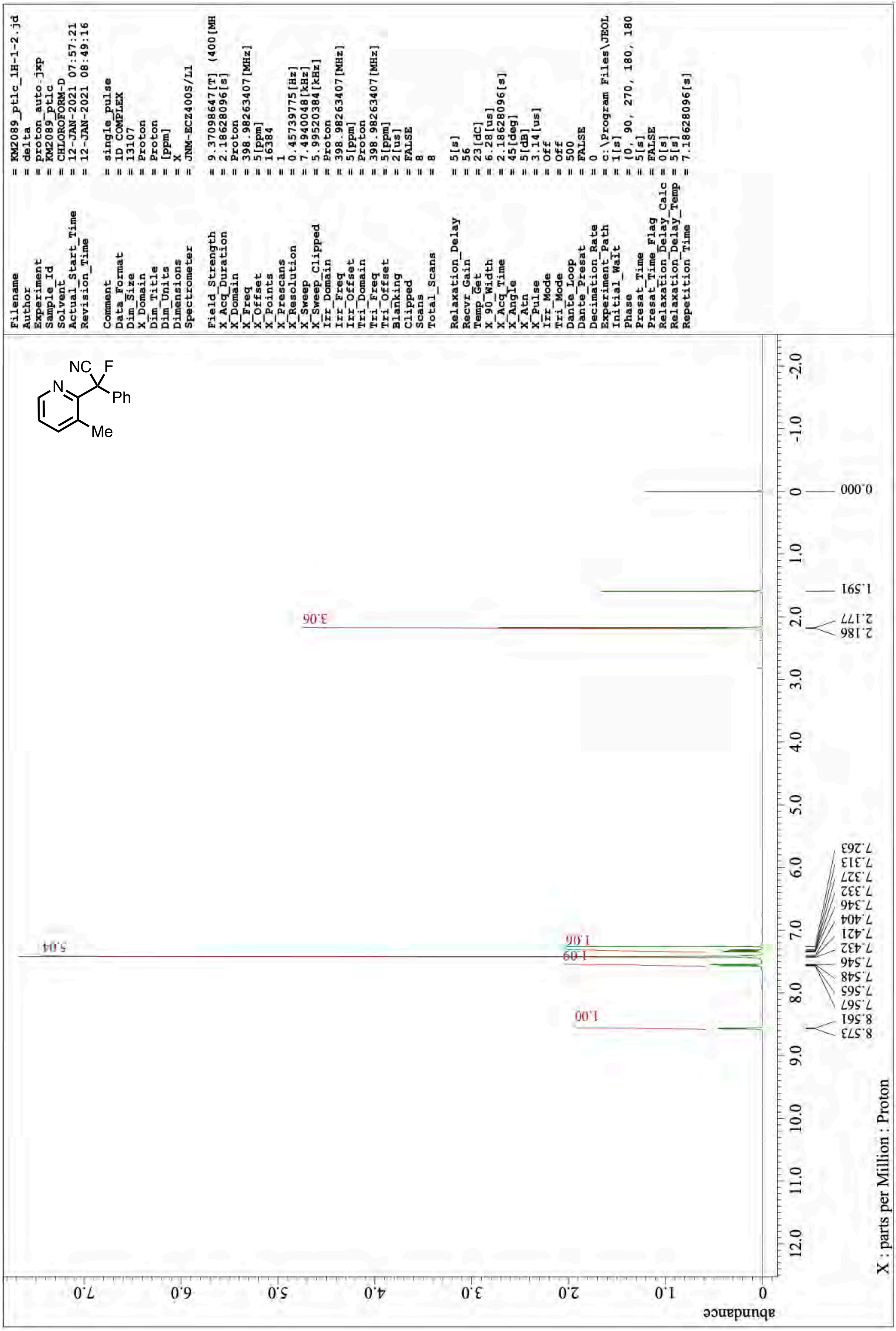

^{13}C NMR of **2AD** (101 MHz, $CDCl_3$)

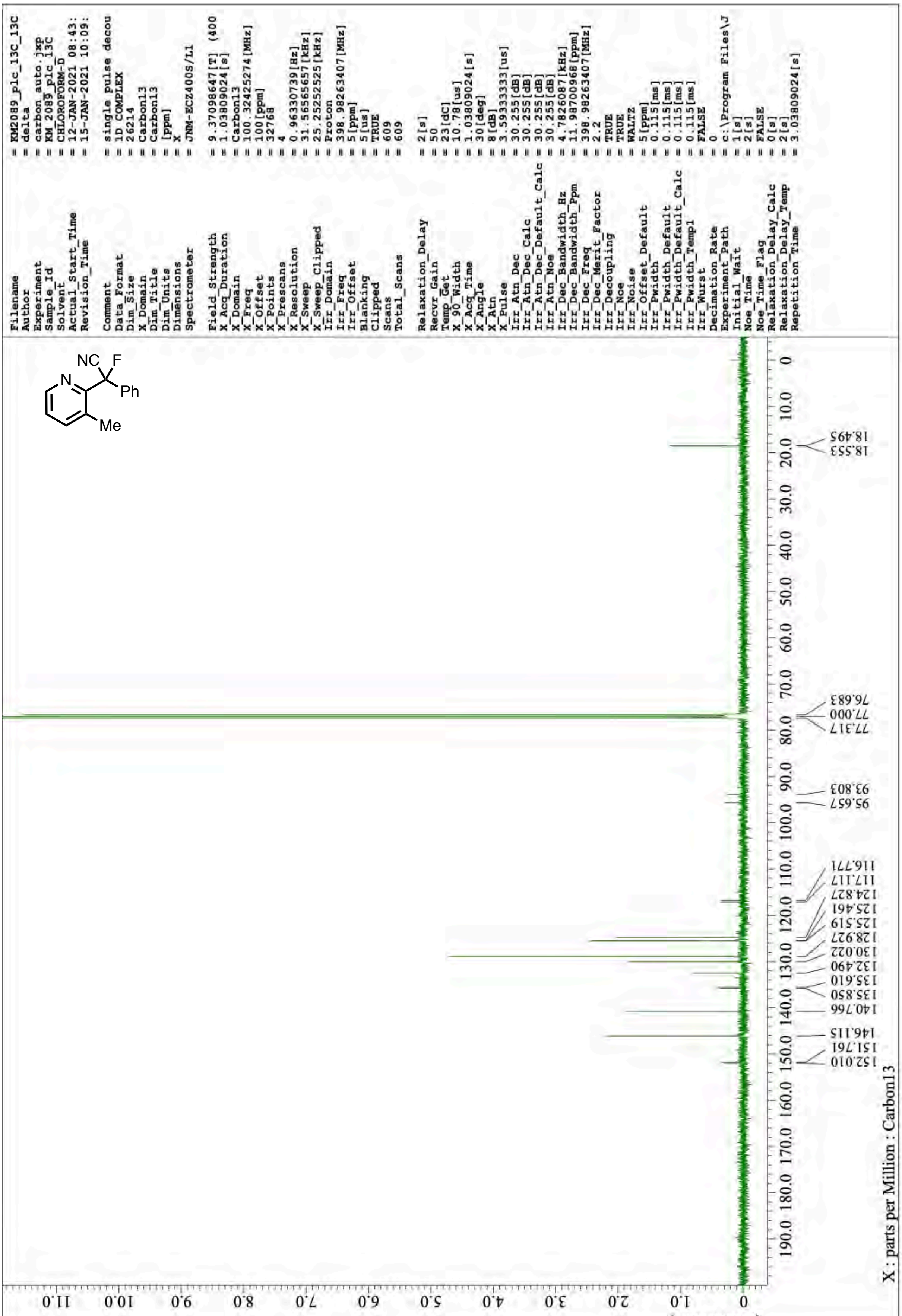

^{19}F NMR of **2AD** (376 MHz, $CDCl_3$)

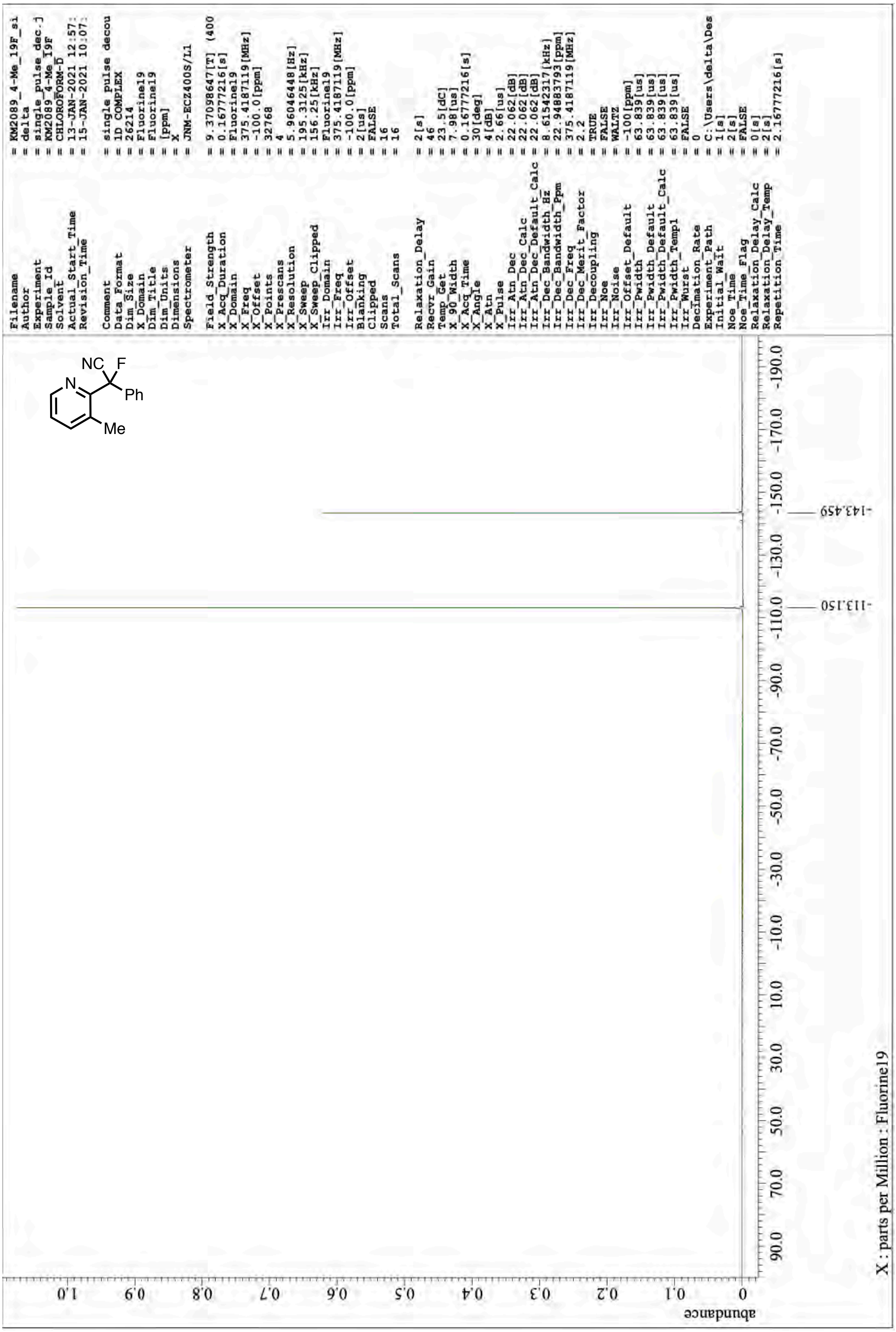

^{1}H NMR of **2AE** (400 MHz, $CDCl_3$)

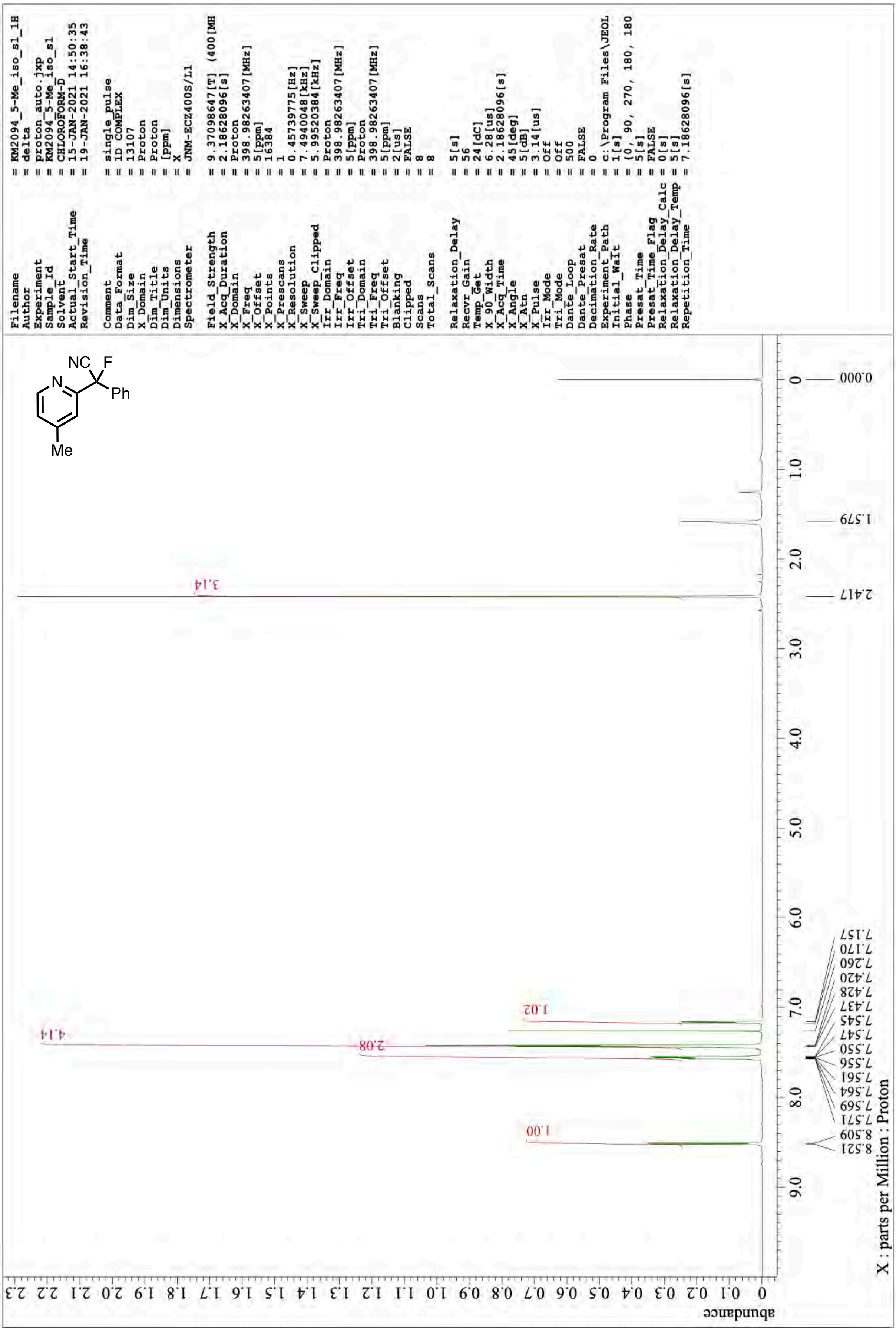

^{13}C NMR of **2AE** (101 MHz, $CDCl_3$)

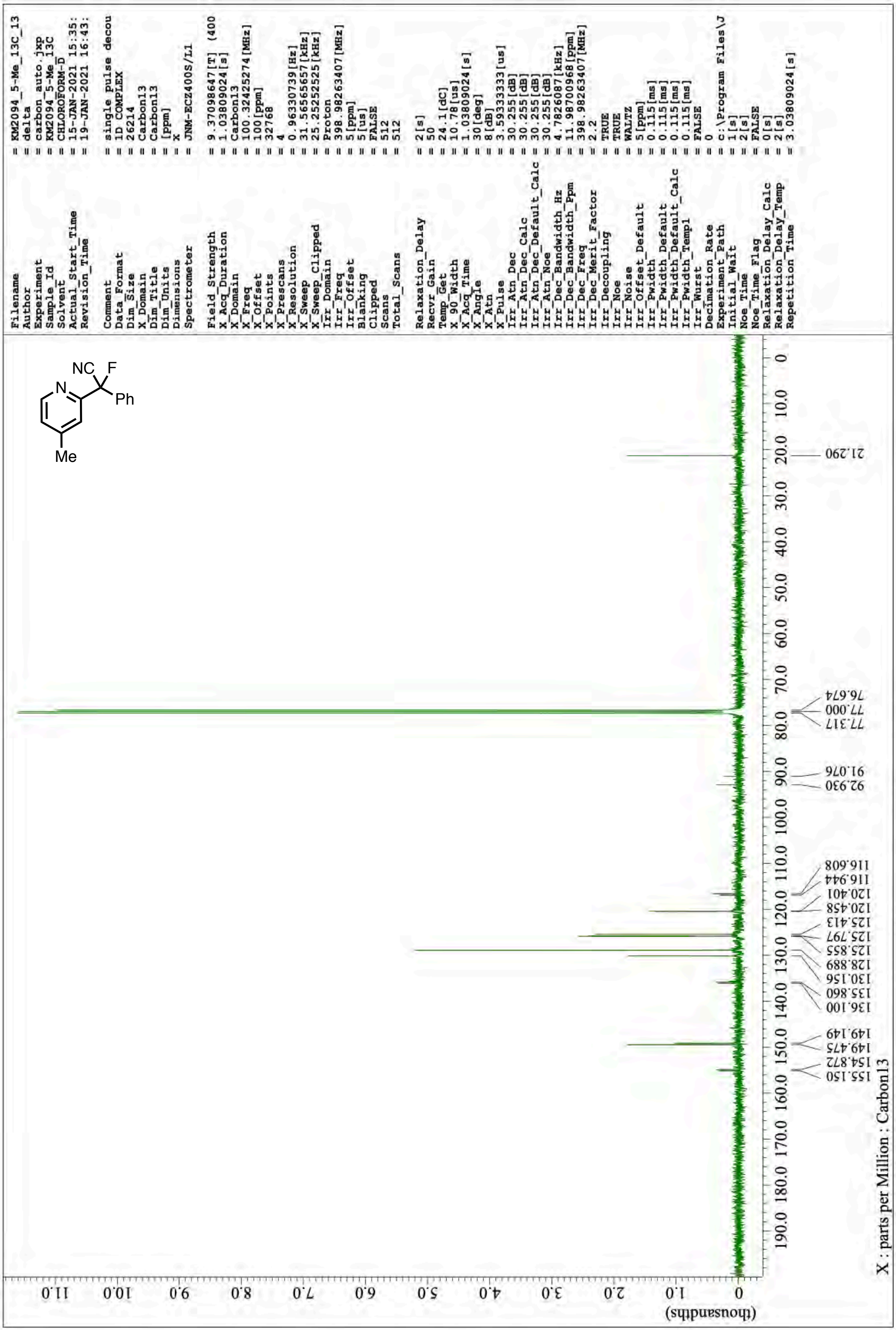

^{19}F NMR of **2AE** (376 MHz, $CDCl_3$)

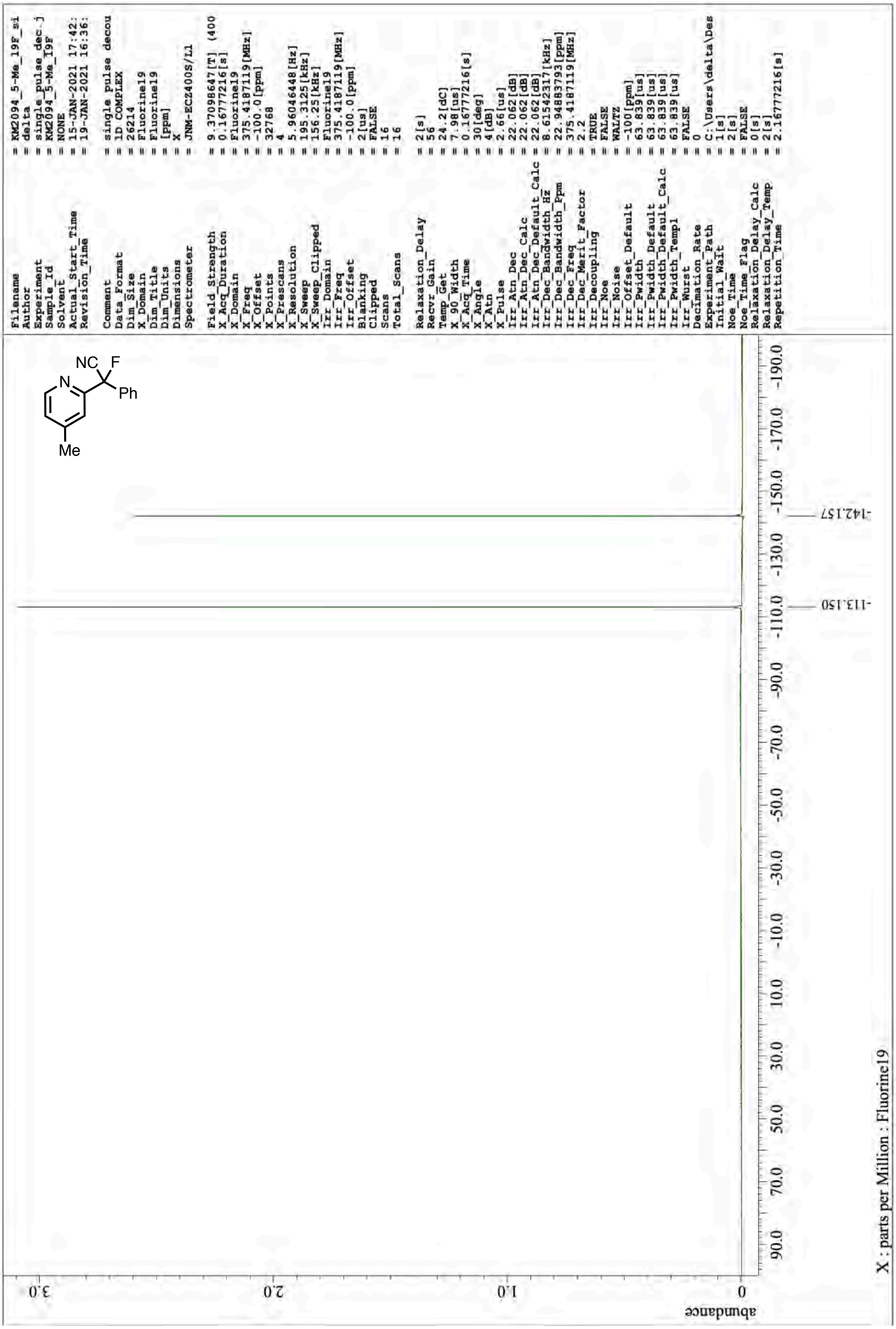

^{1}H NMR of **2AF** (400 MHz, $CDCl_3$)

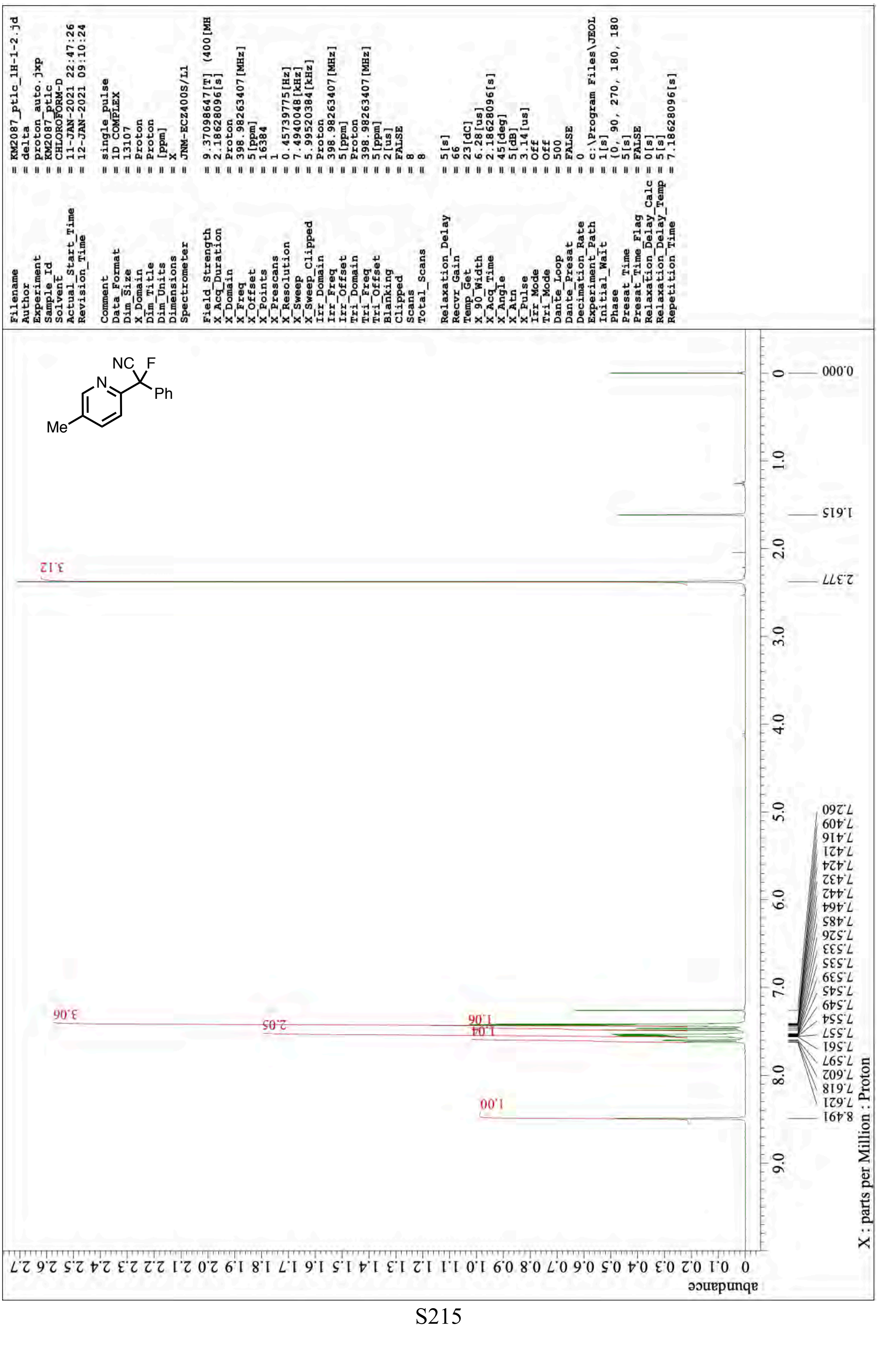

^{13}C NMR of **2AF** (101 MHz, $CDCl_3$)

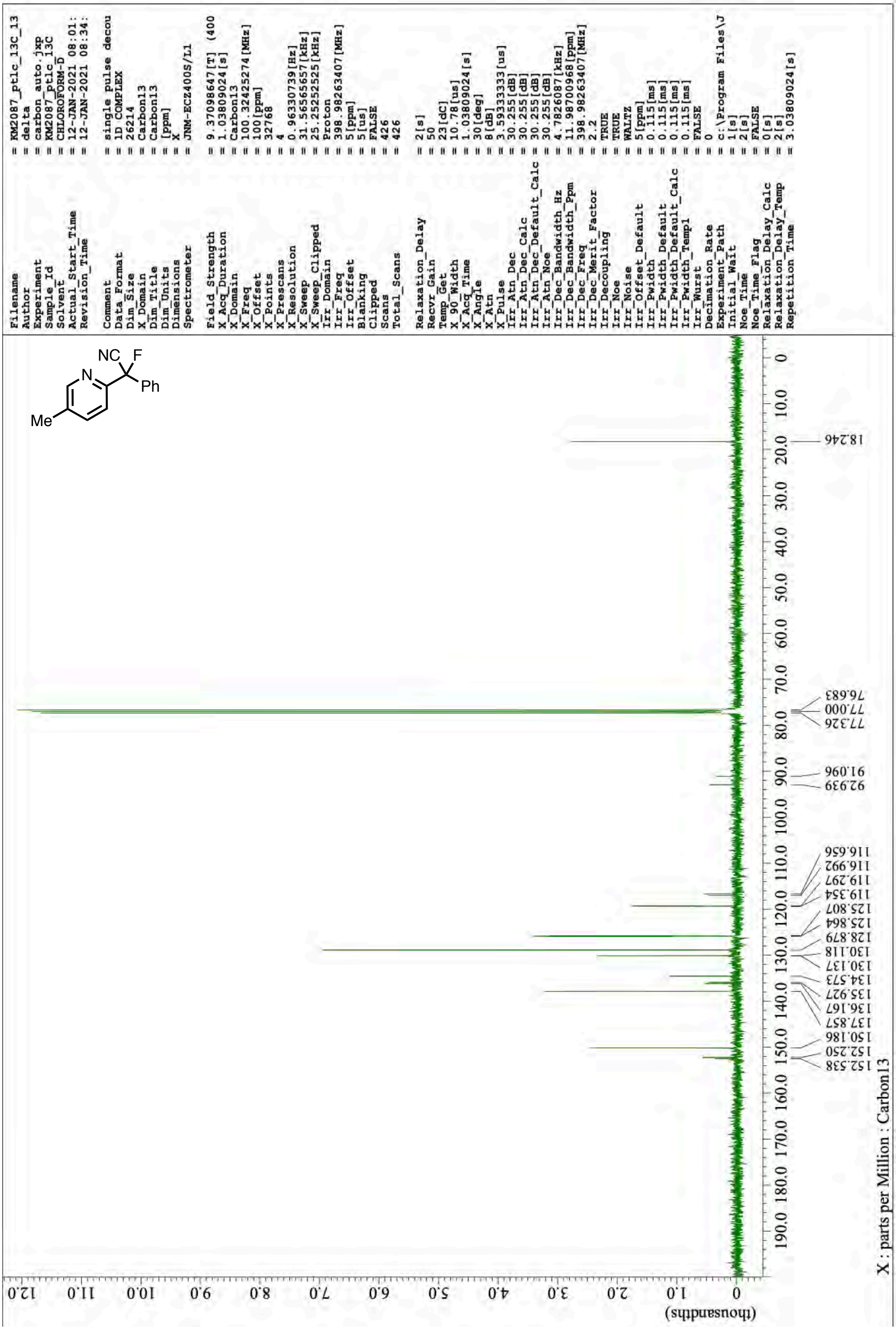

^{19}F NMR of **2AF** (376 MHz, $CDCl_3$)

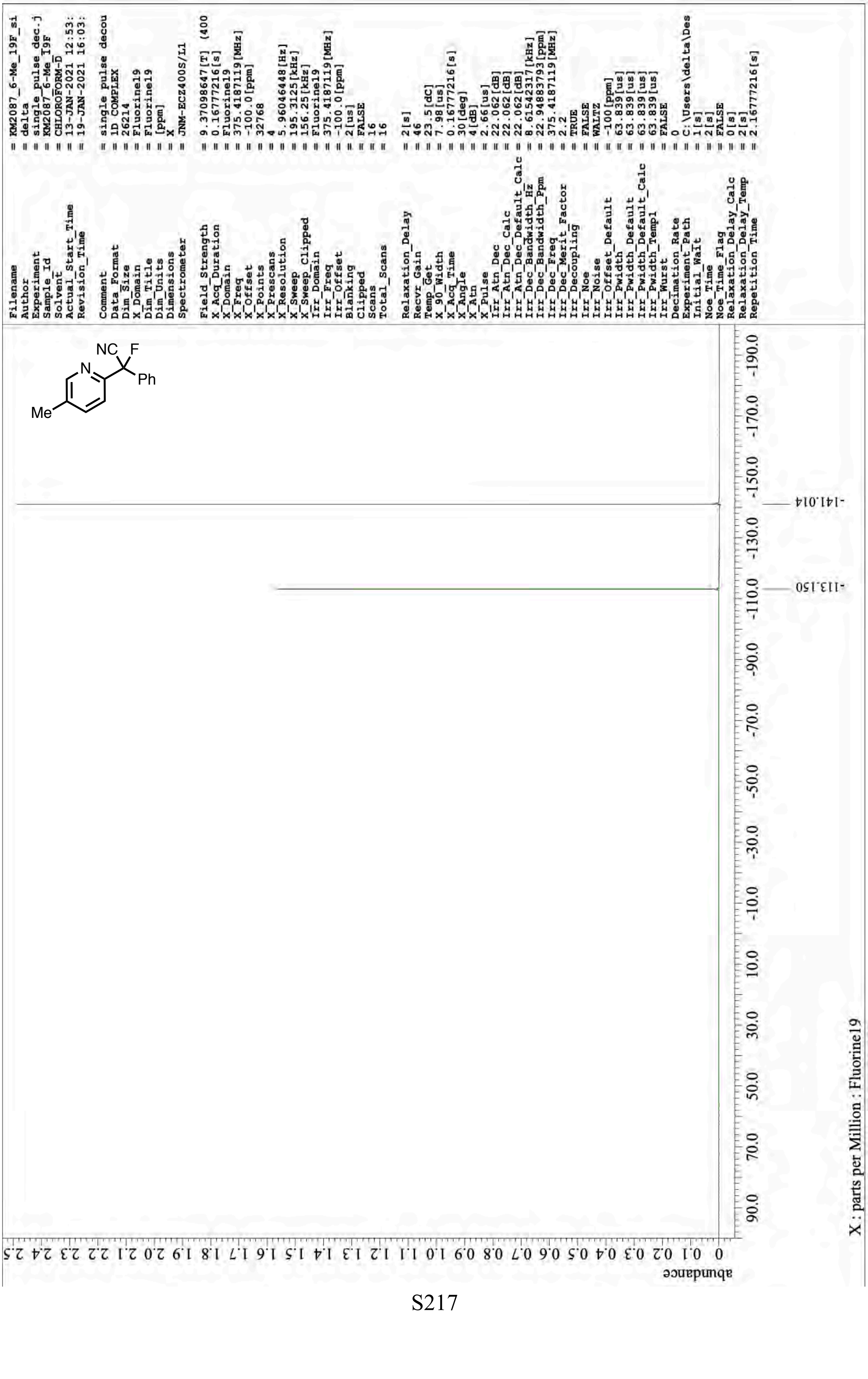

^{1}H NMR of **2AG** (400 MHz, $CDCl_3$)

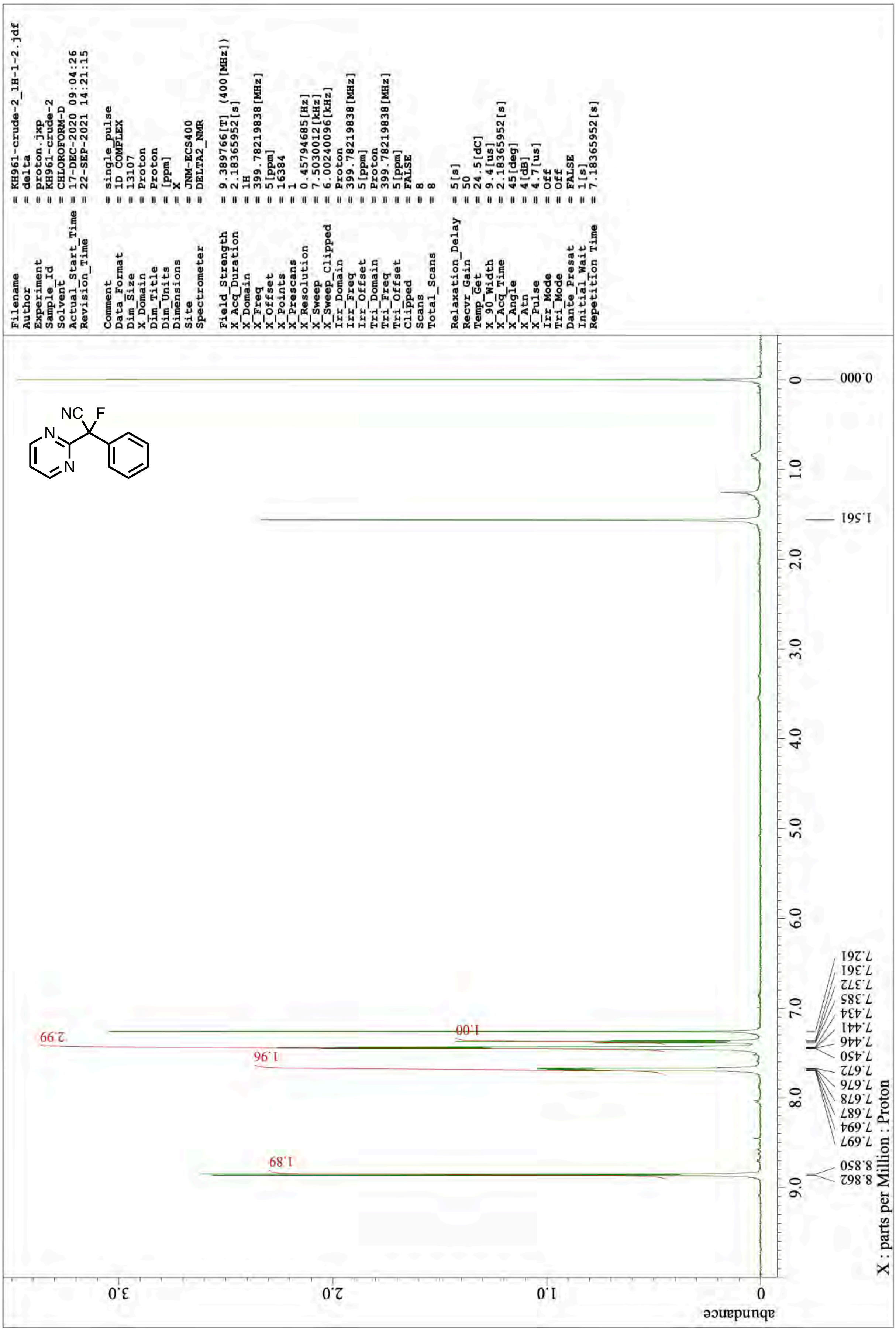

^{13}C NMR of **2AG** (101 MHz, $CDCl_3$)

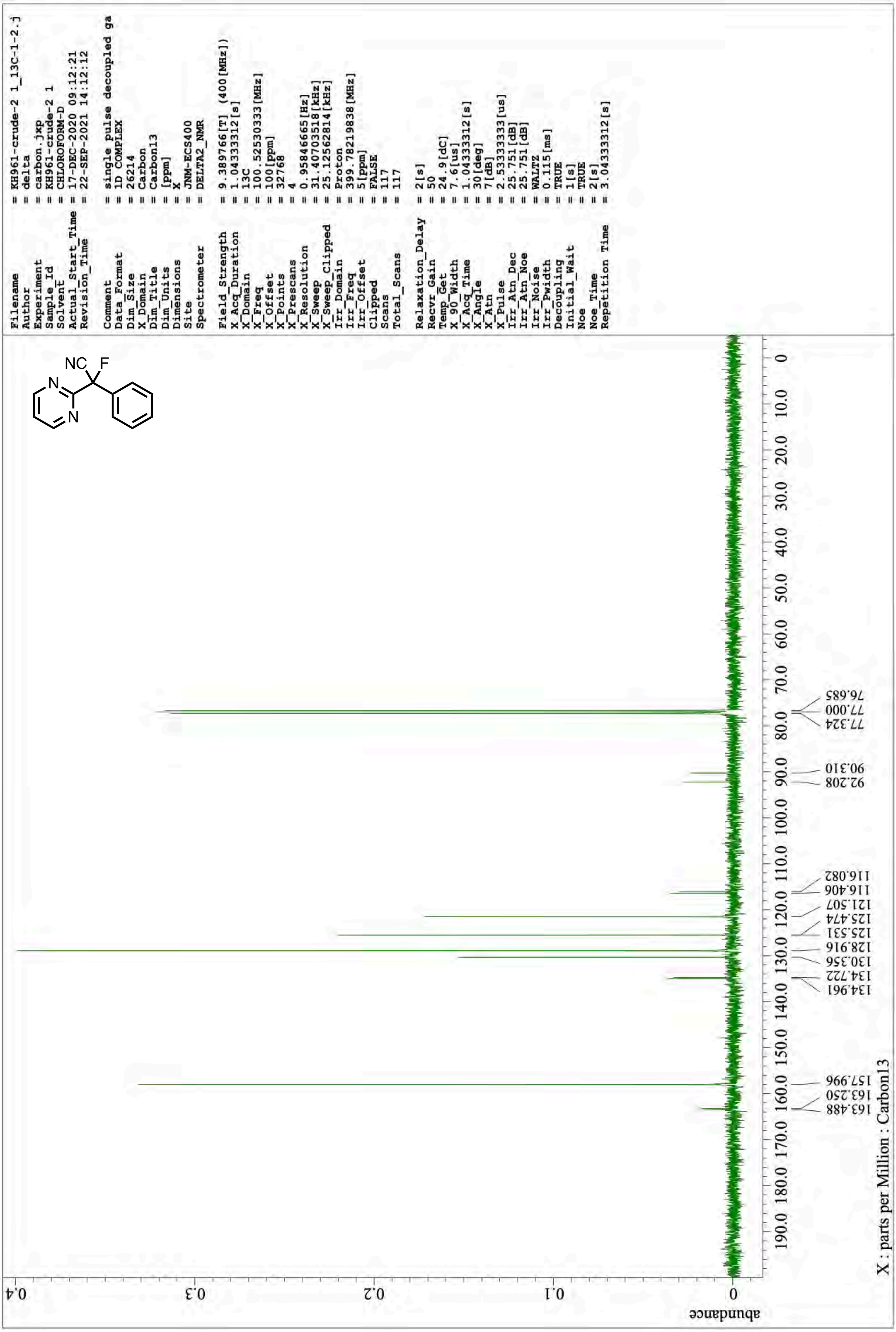

^{19}F NMR of **2AG** (376 MHz, $CDCl_3$)

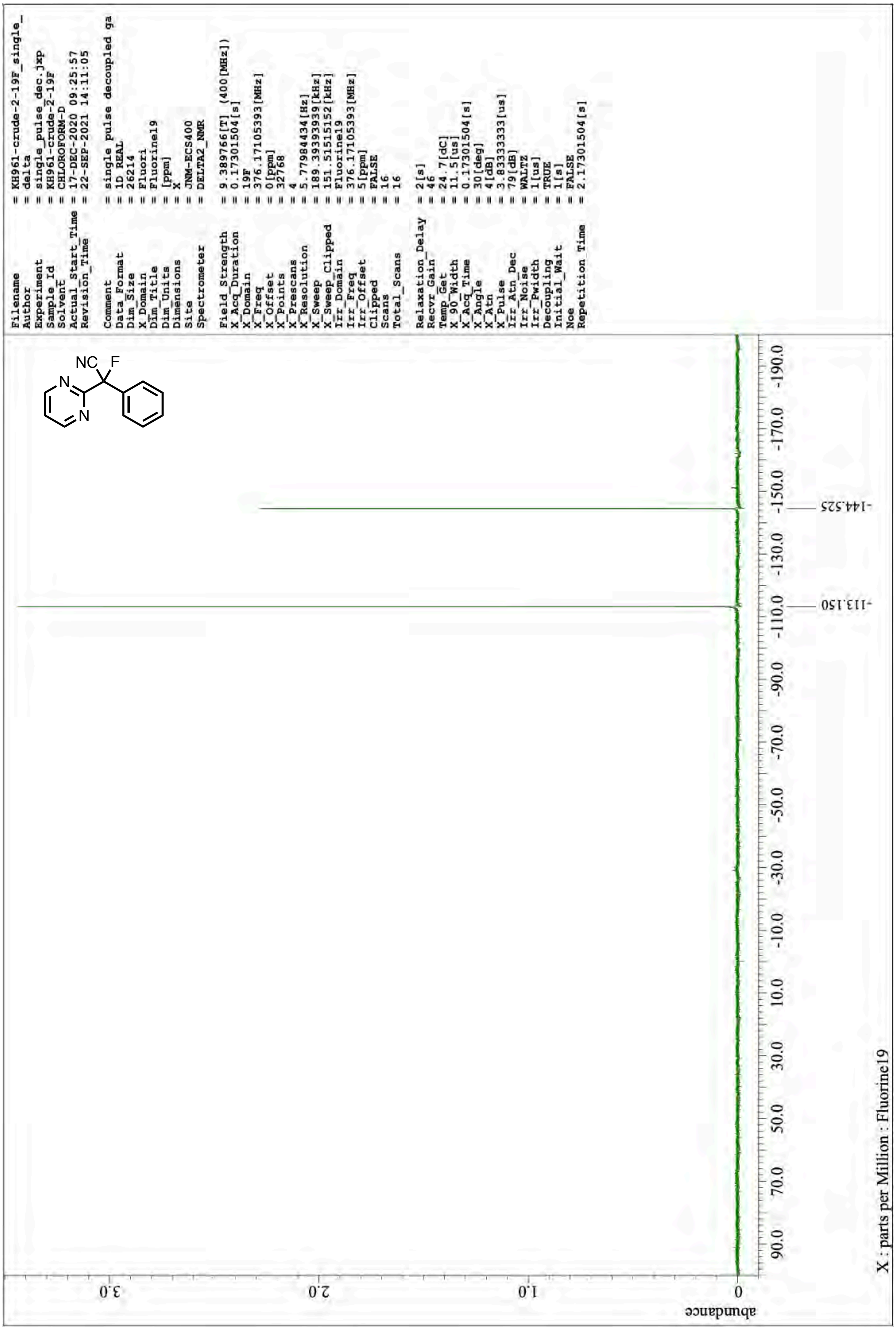

^{1}H NMR of **2AH** (400 MHz, $CDCl_3$)

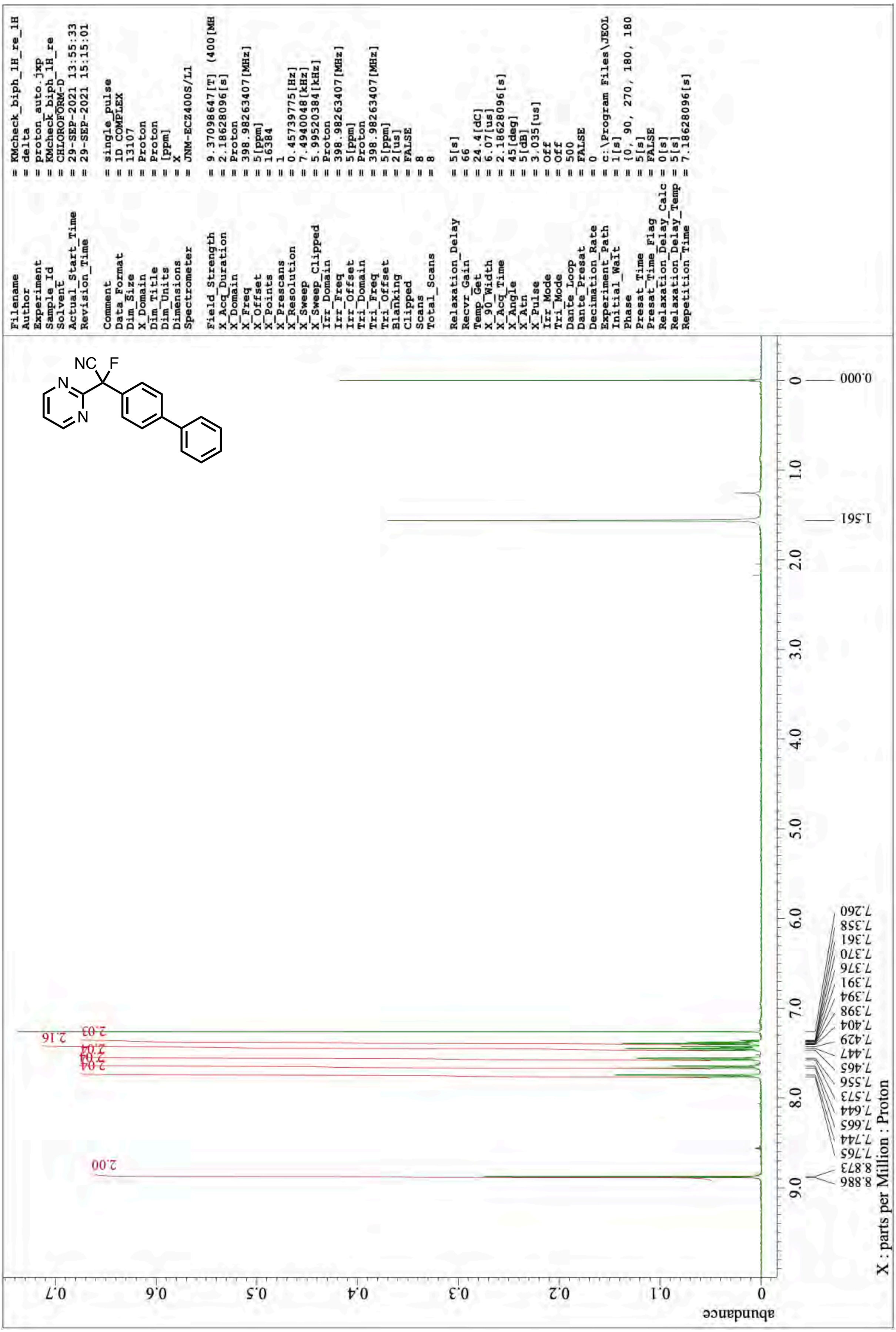

^{13}C NMR of **2AH** (101 MHz, $CDCl_3$)

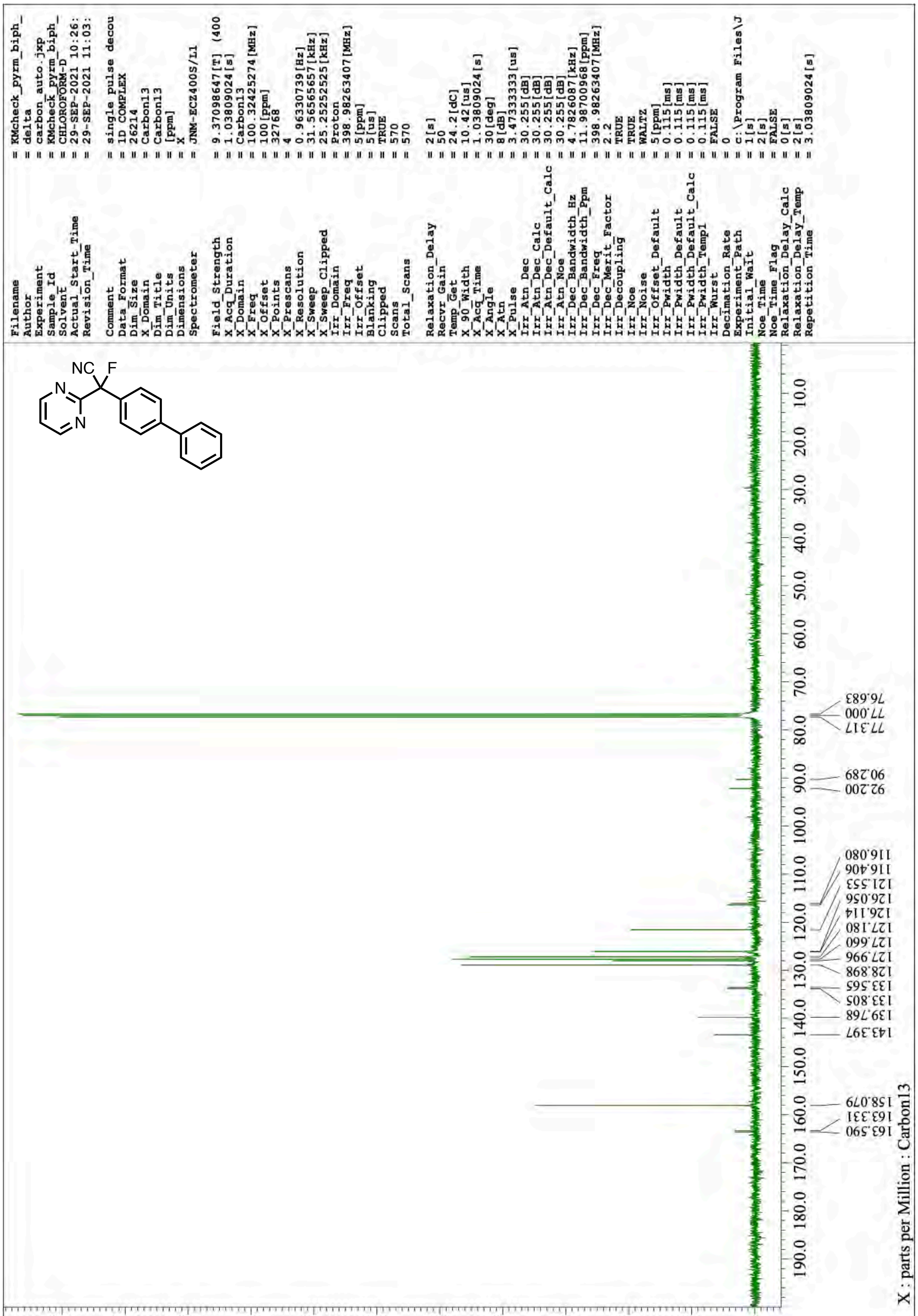

^{19}F NMR of **2AH** (376 MHz, $CDCl_3$)

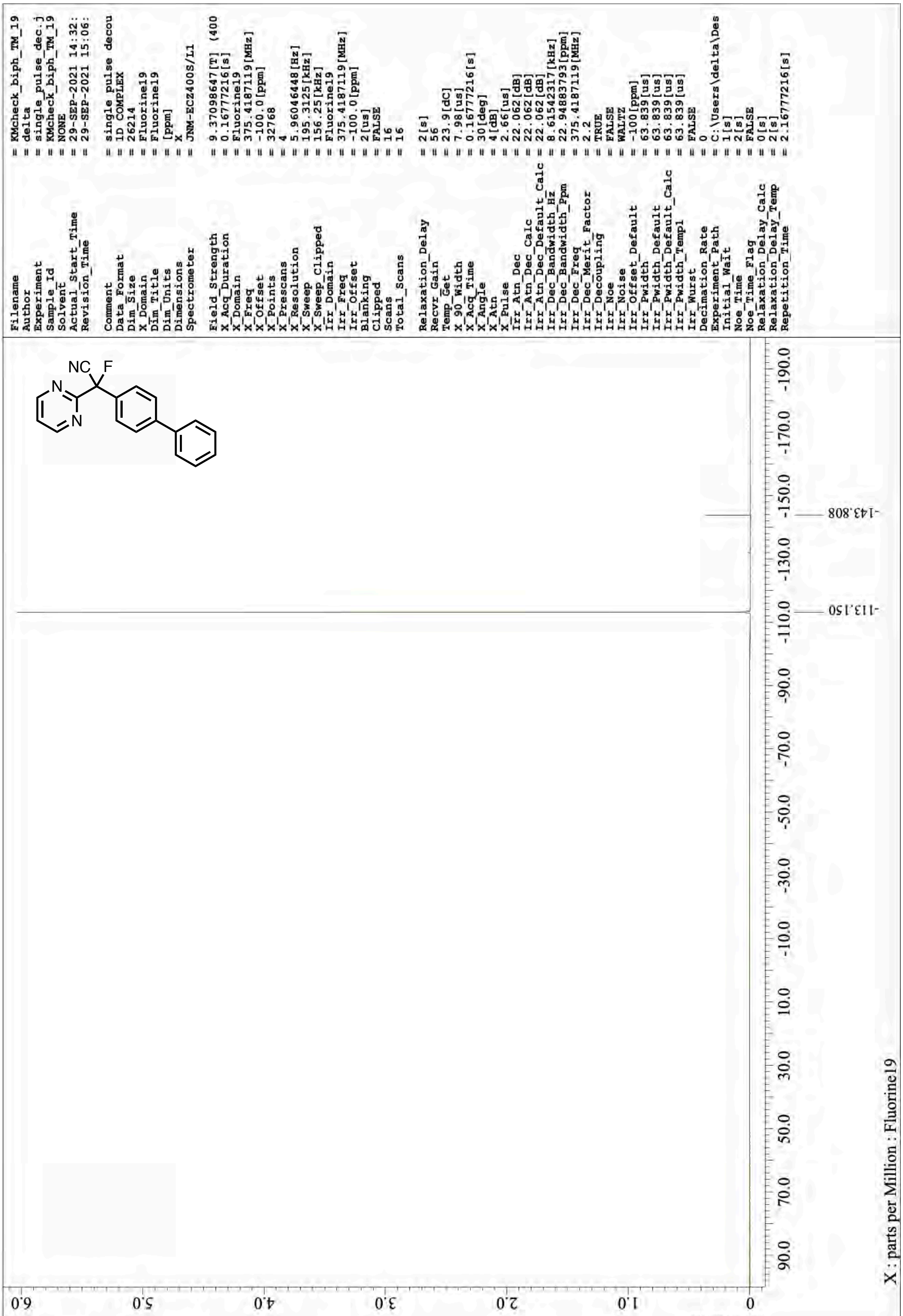

^1H NMR of **2AI** (400 MHz, $CDCl_3$)

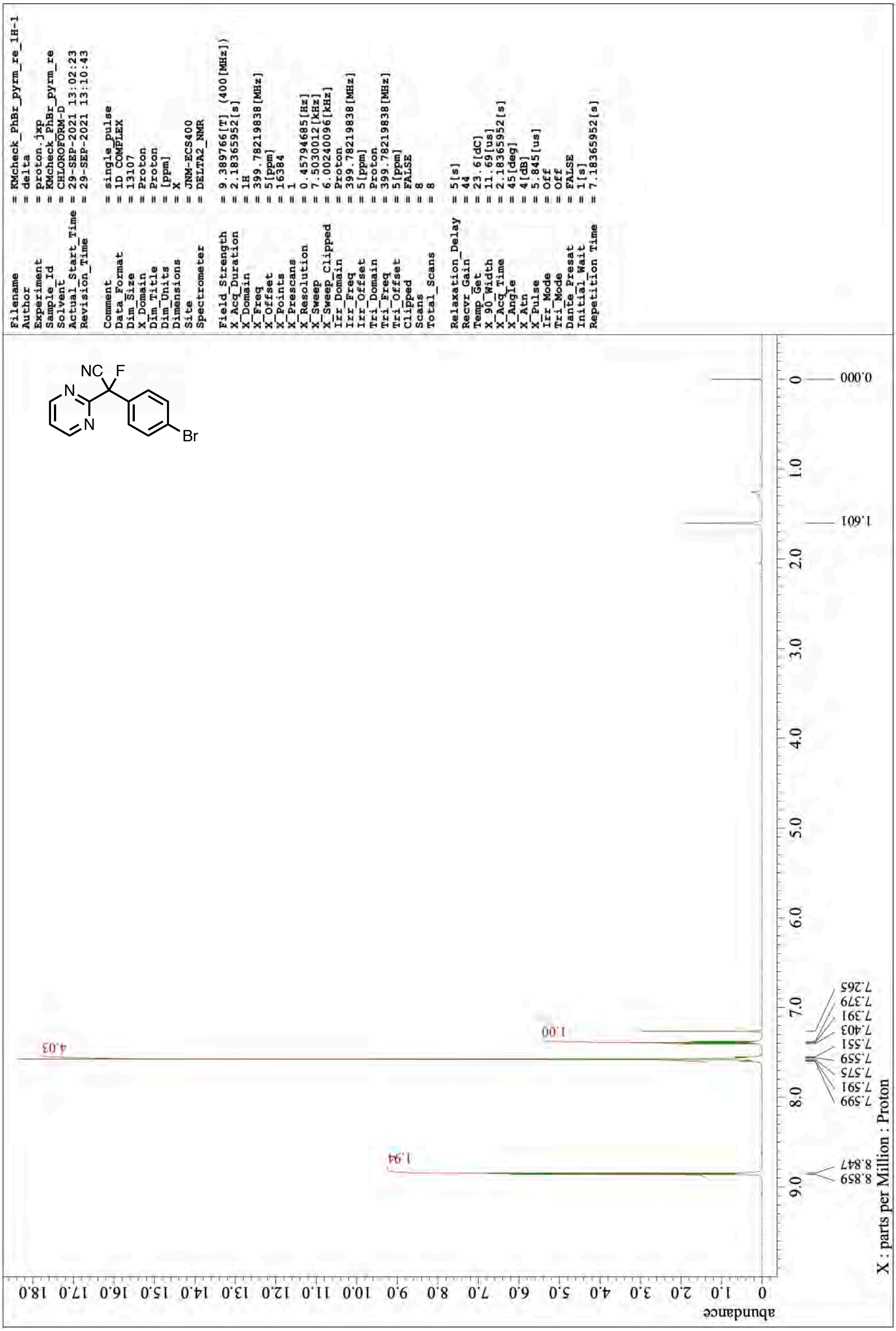

^{13}C NMR of **2AI** (101 MHz, $CDCl_3$)

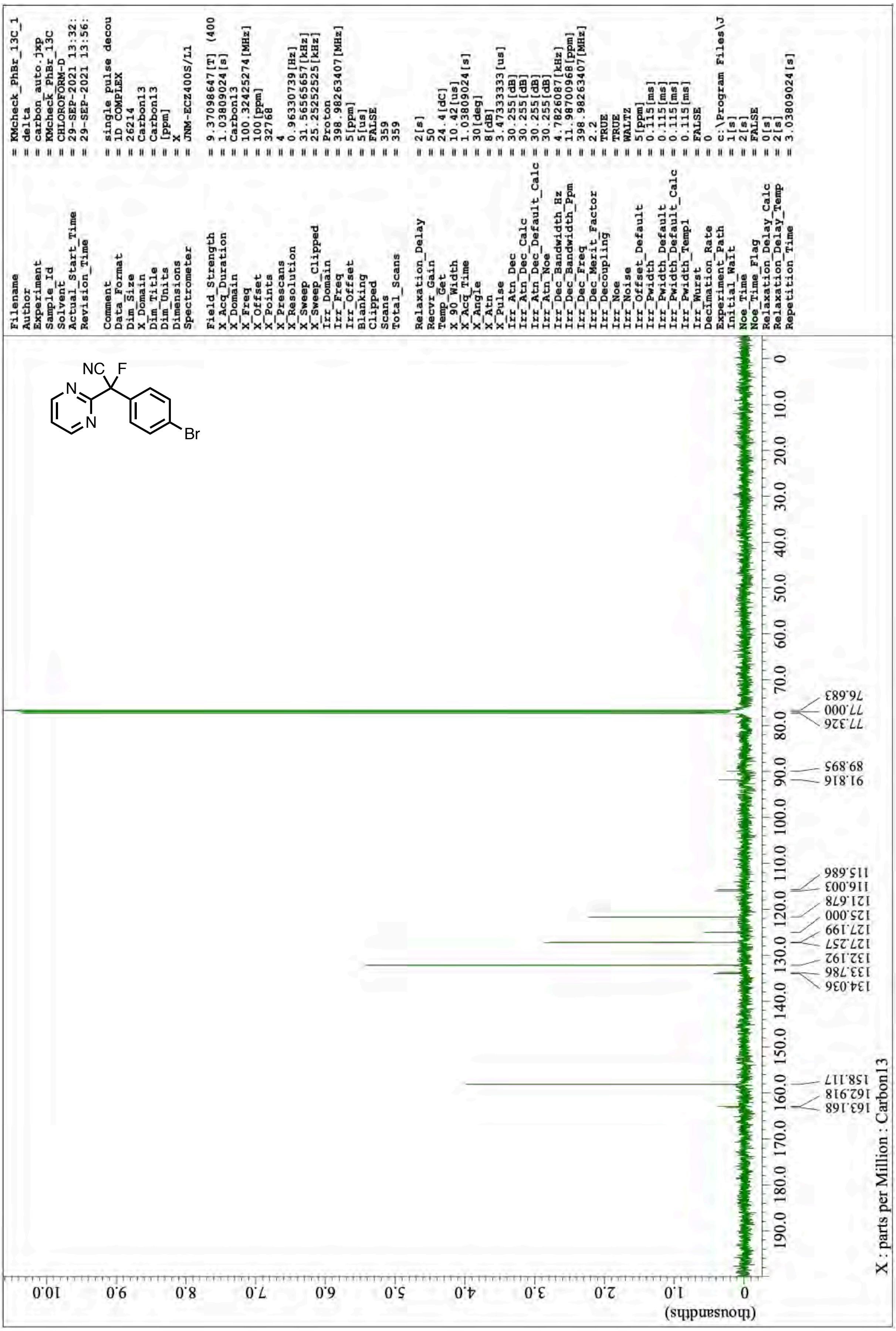

^{19}F NMR of **2AI** (376 MHz, $CDCl_3$)

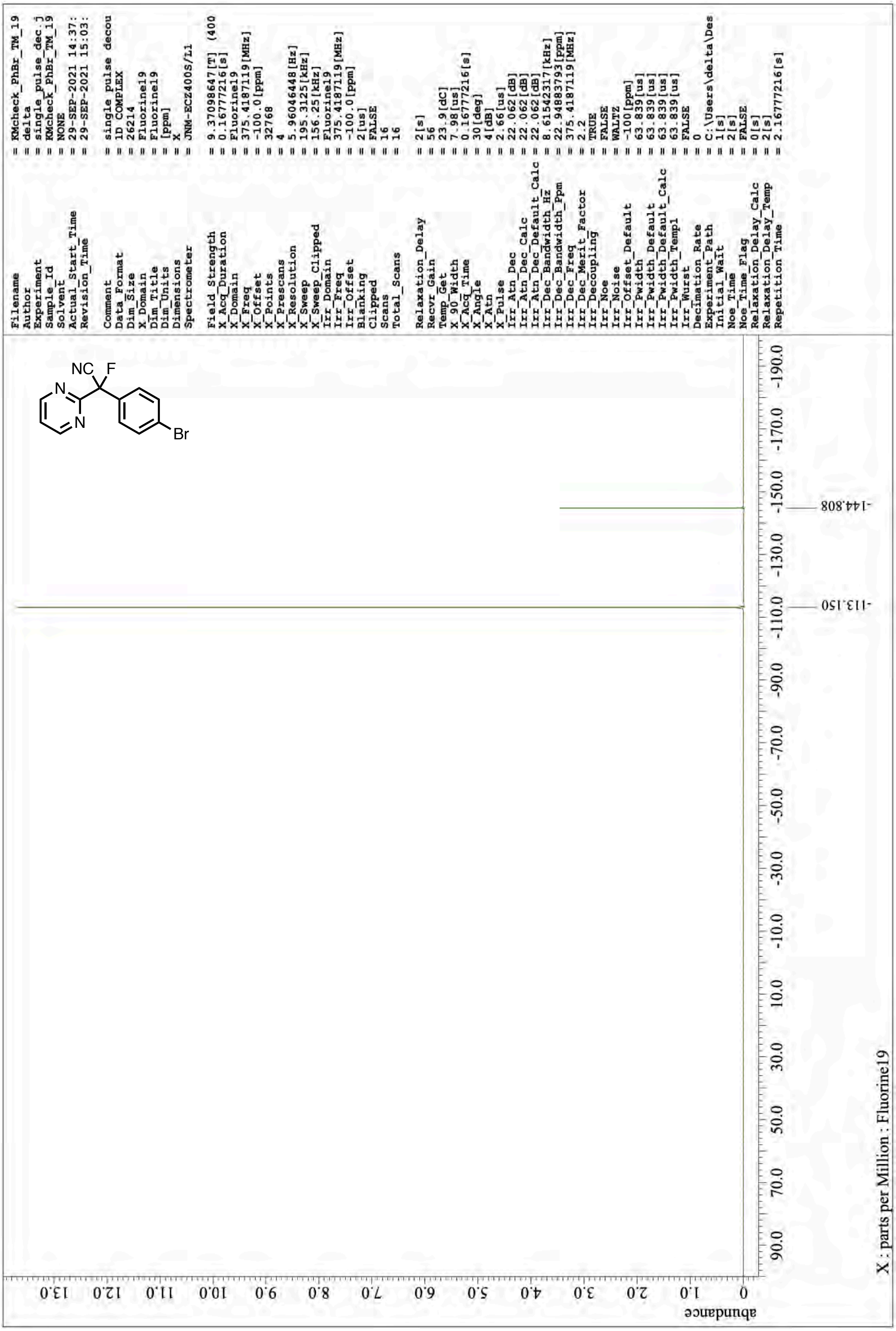

^{1}H NMR of **2AJ** (400 MHz, $CDCl_3$)

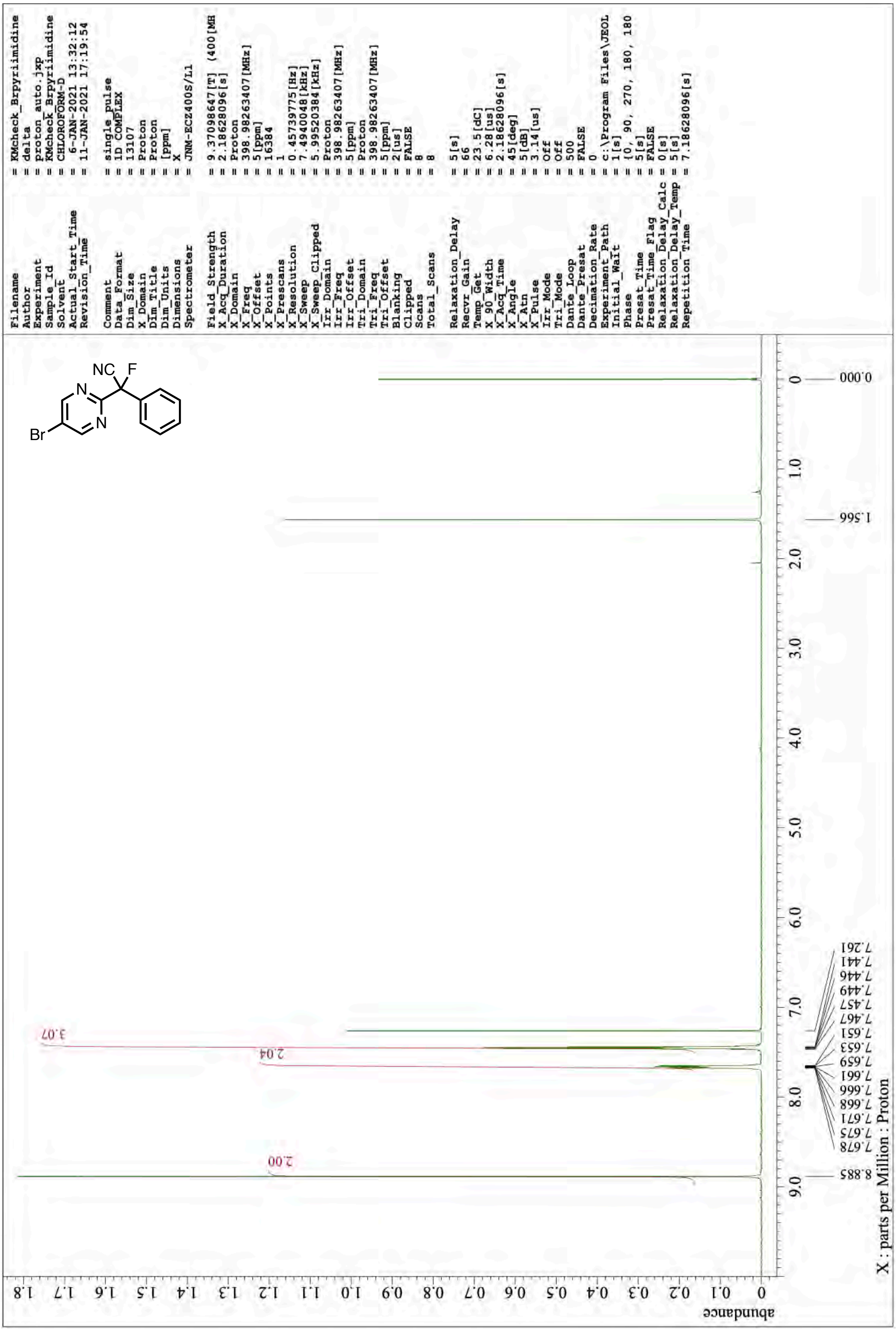

^{13}C NMR of **2AJ** (101 MHz, $CDCl_3$)

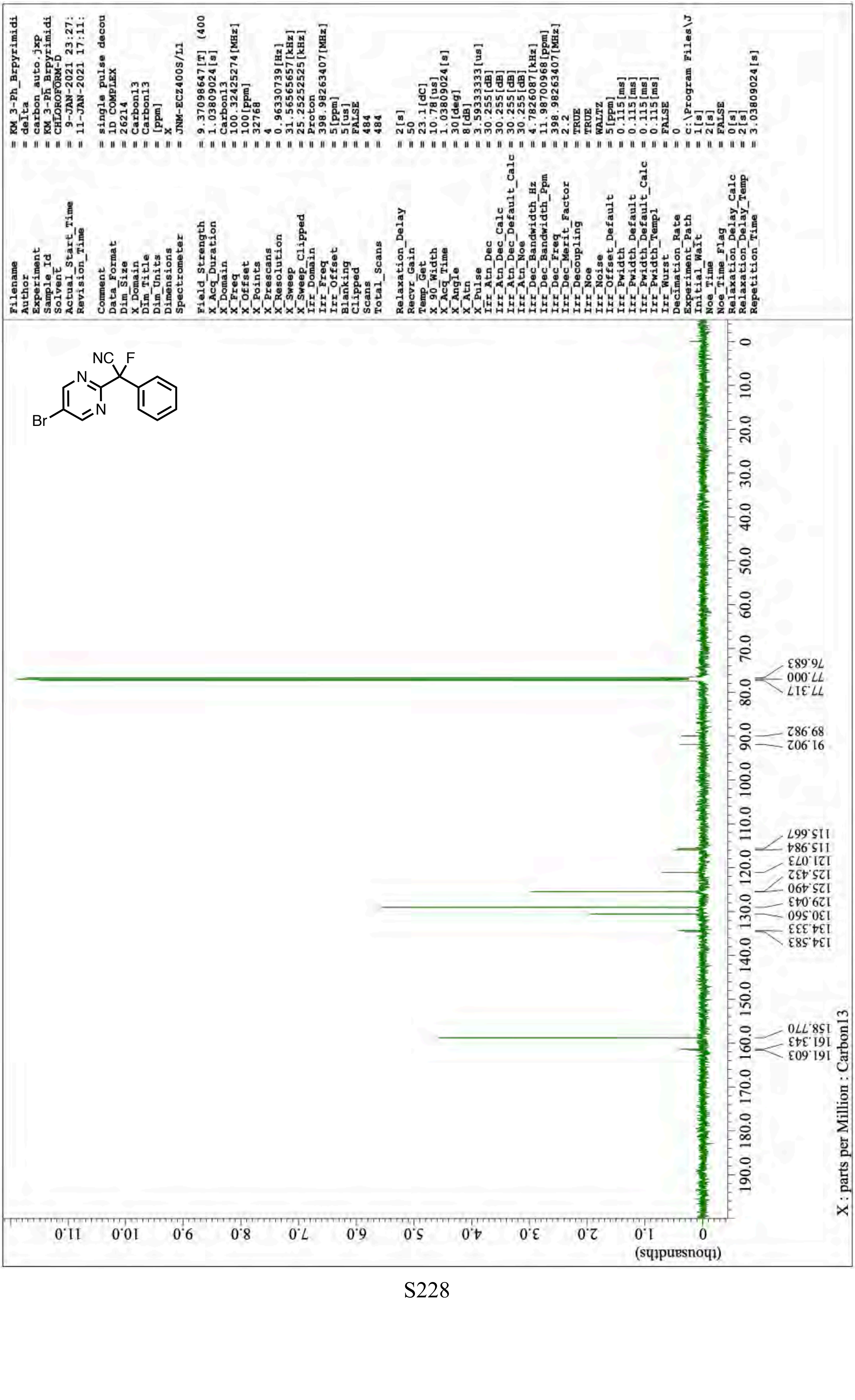

^{19}F NMR of **2AJ** (376 MHz, $CDCl_3$)

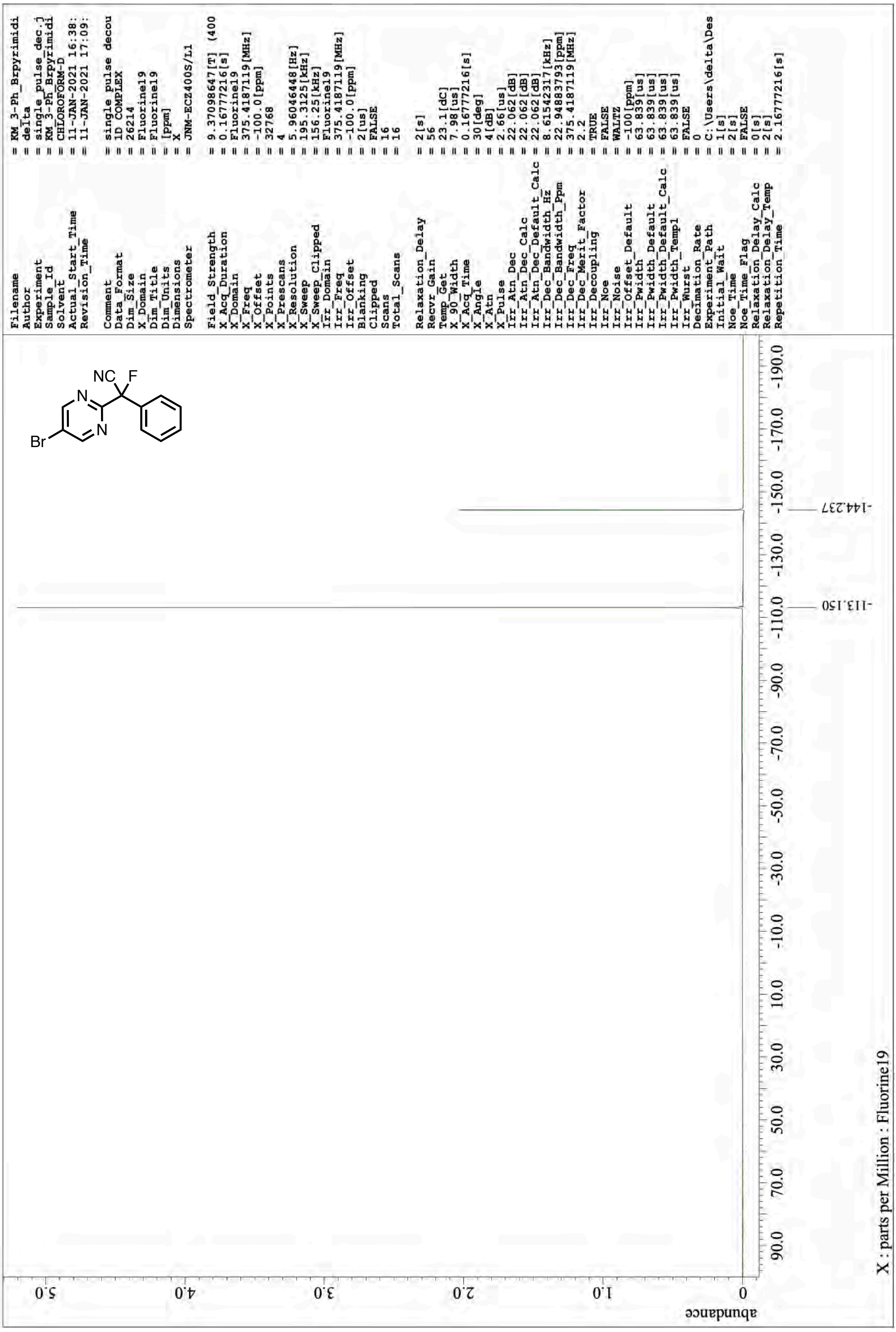

^{1}H NMR of **2AK** (400 MHz, $CDCl_3$)

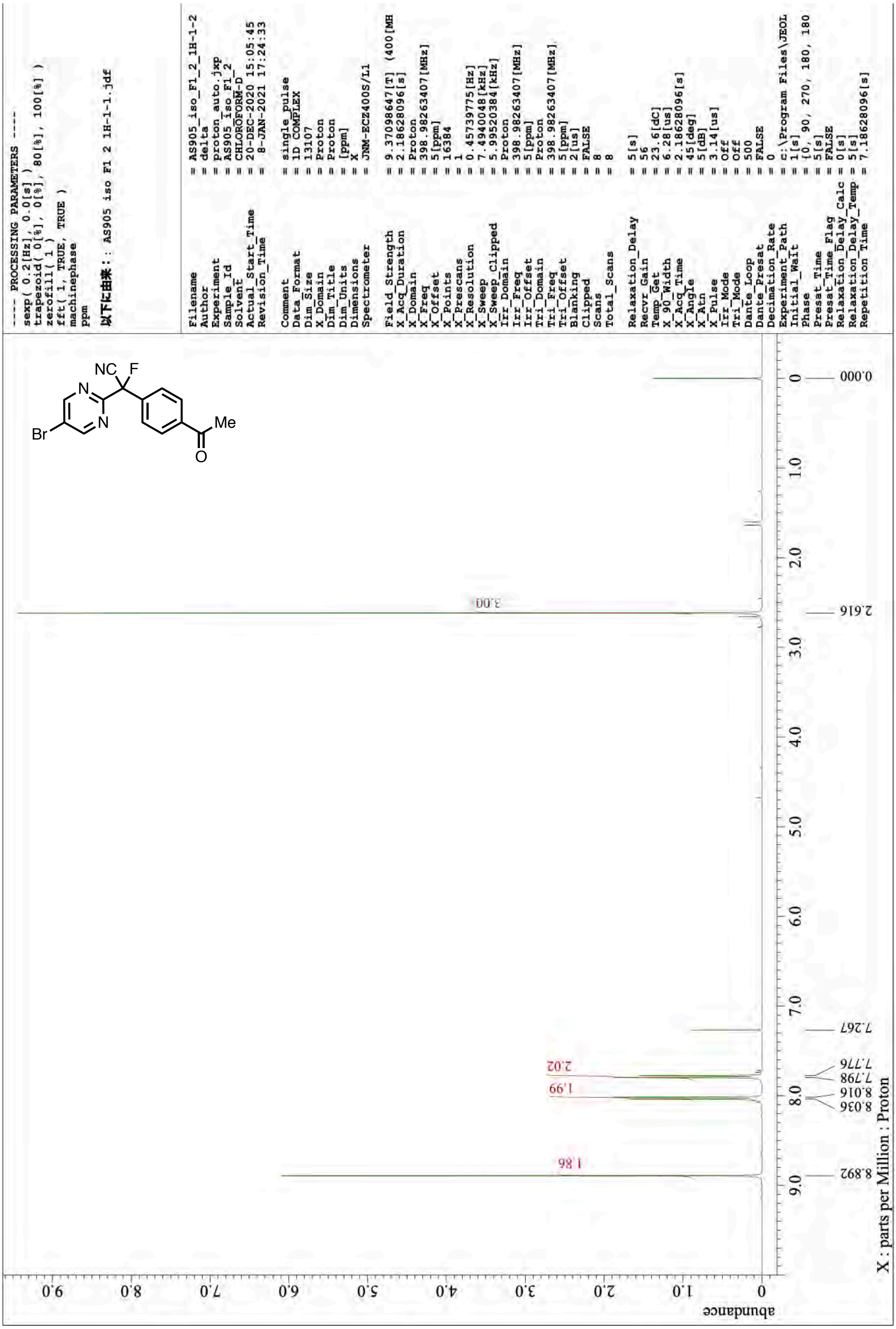

^{13}C NMR of **2AK** (101 MHz, $CDCl_3$)

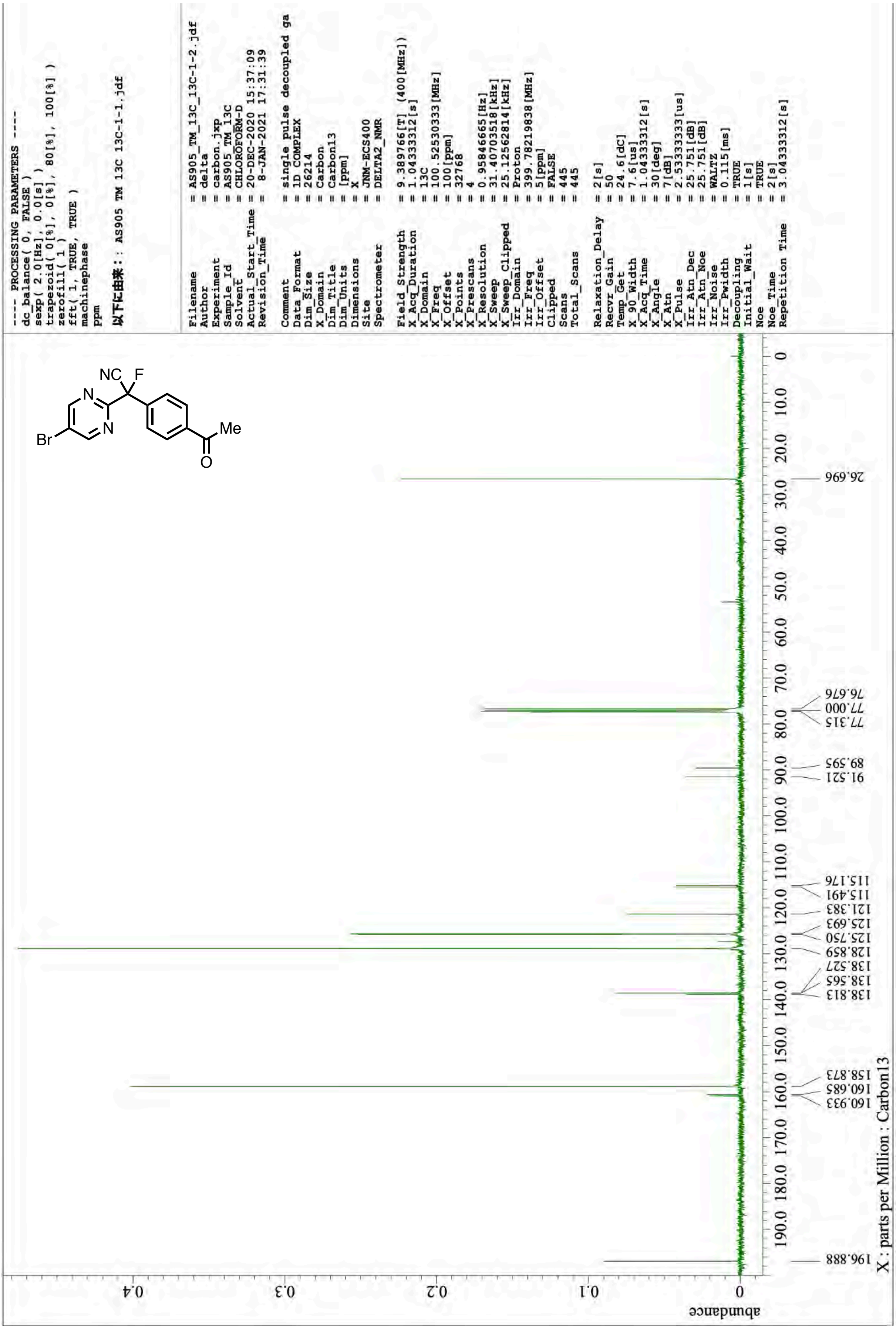

^{19}F NMR of **2AK** (376 MHz, $CDCl_3$)

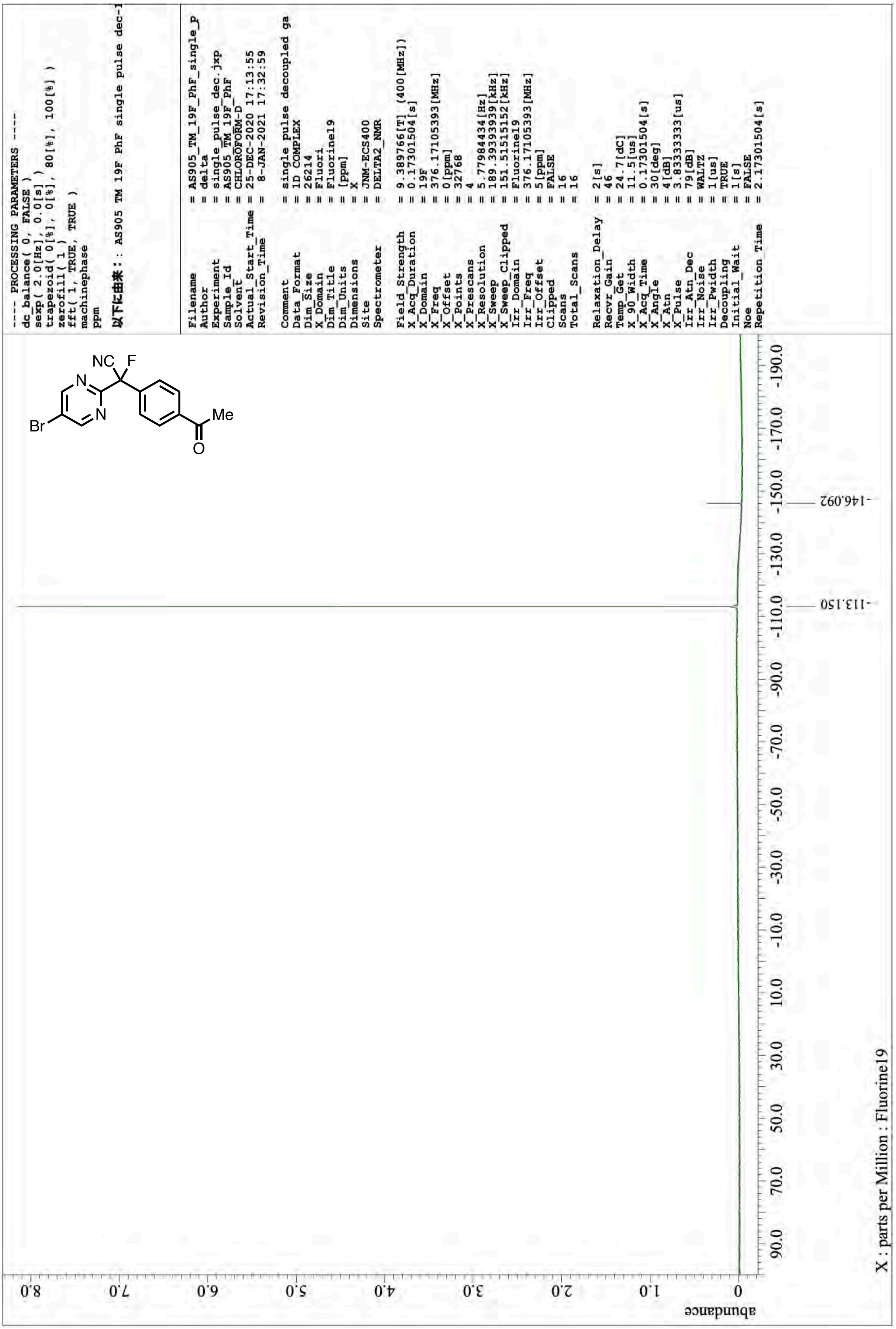

^{1}H NMR of **2AL** (400 MHz, $CDCl_3$)

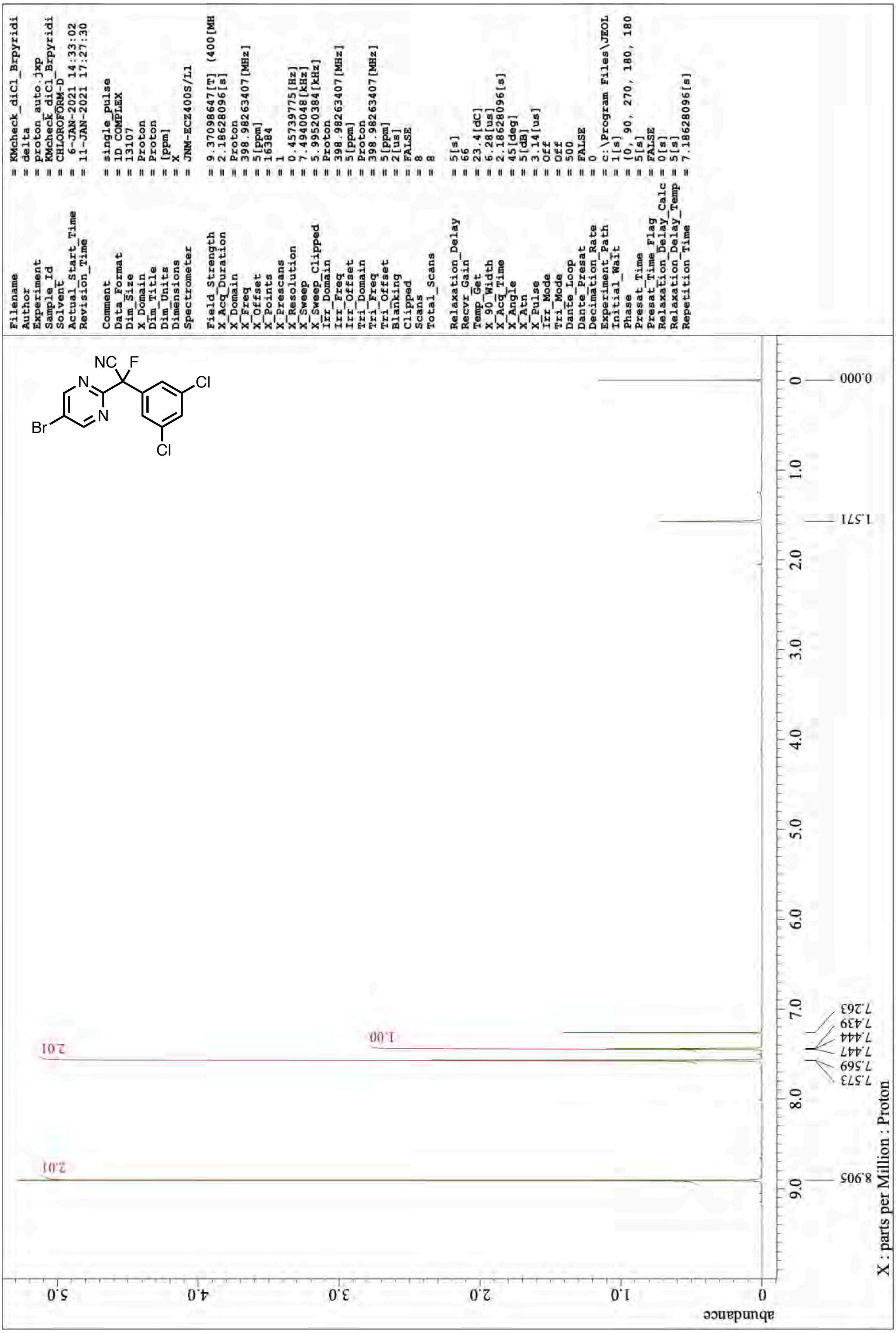

^{13}C NMR of **2AL** (101 MHz, $CDCl_3$)

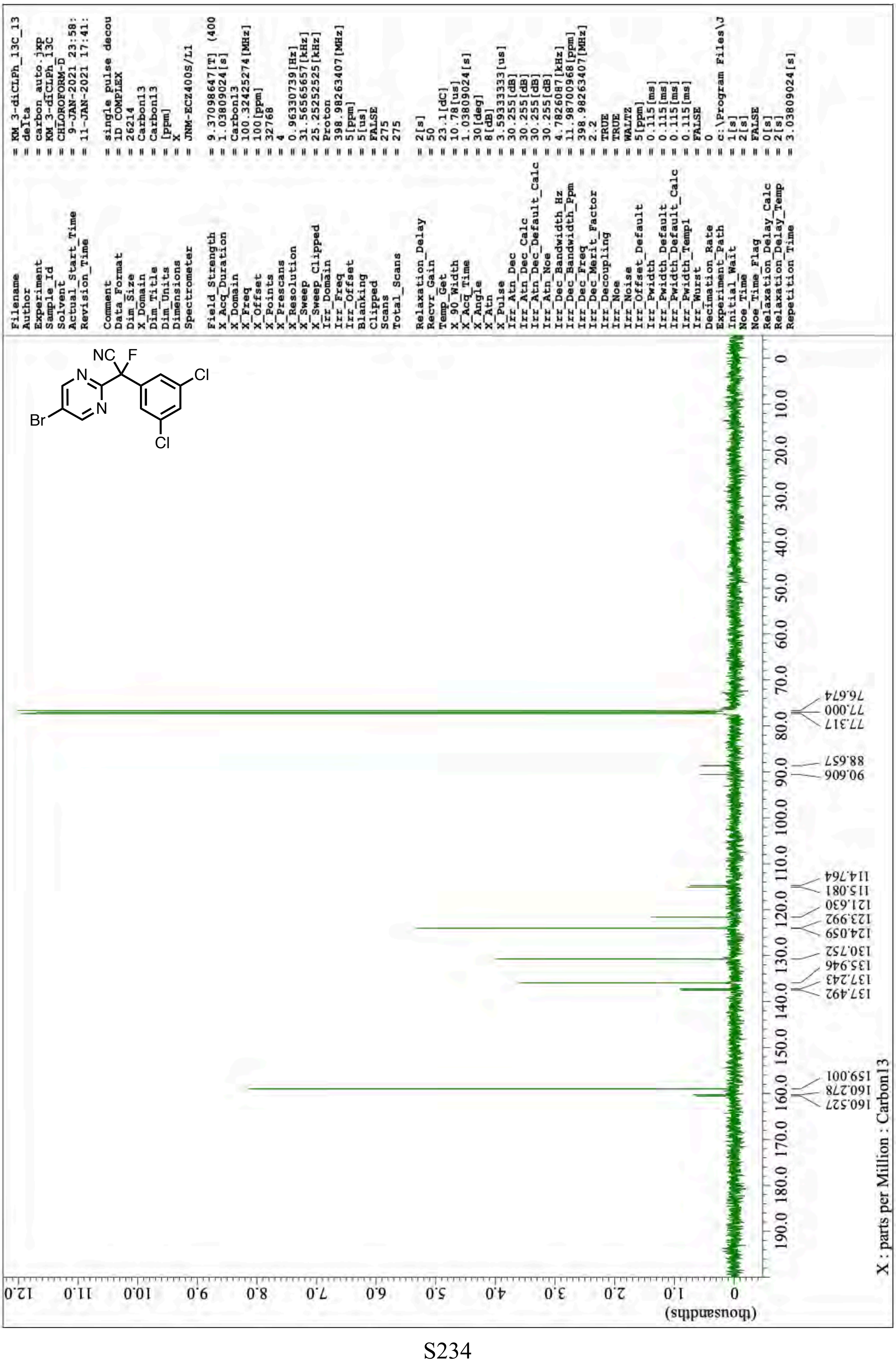

^{19}F NMR of **2AL** (376 MHz, $CDCl_3$)

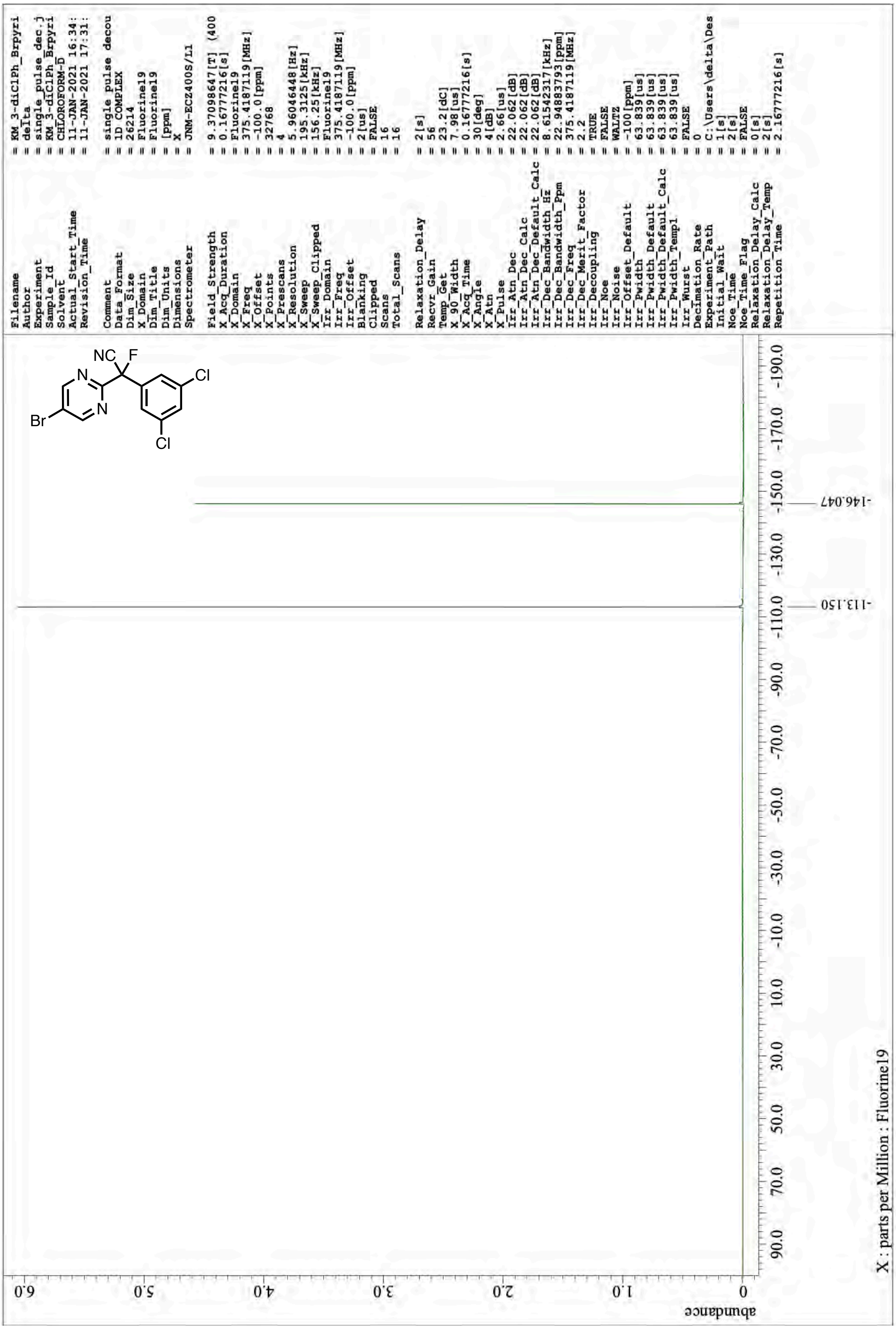

^{1}H NMR of **2AM** (400 MHz, $CDCl_3$)

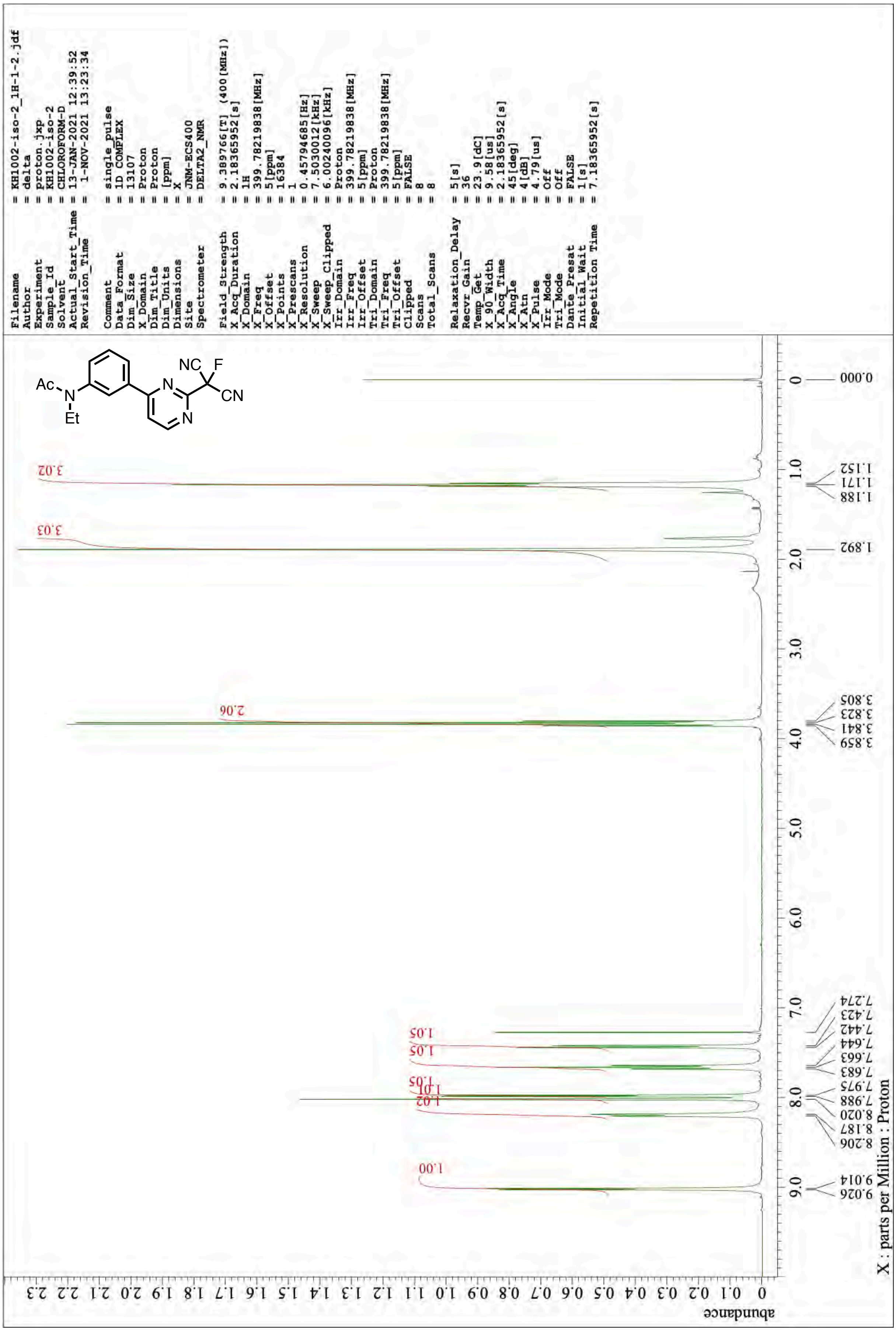

^{13}C NMR of **2AM** (101 MHz, $CDCl_3$)

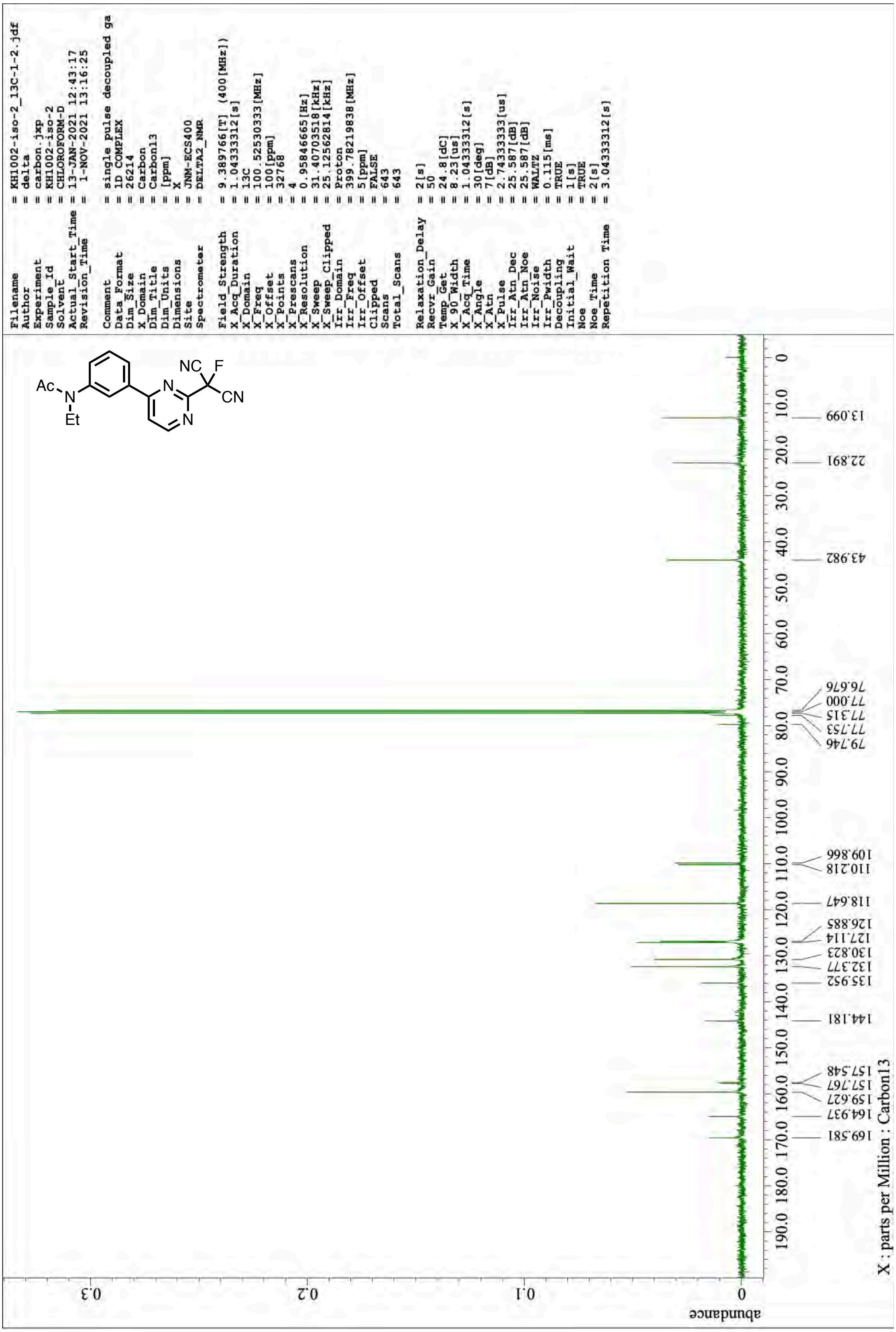

^{19}F NMR of **2AM** (376 MHz, $CDCl_3$)

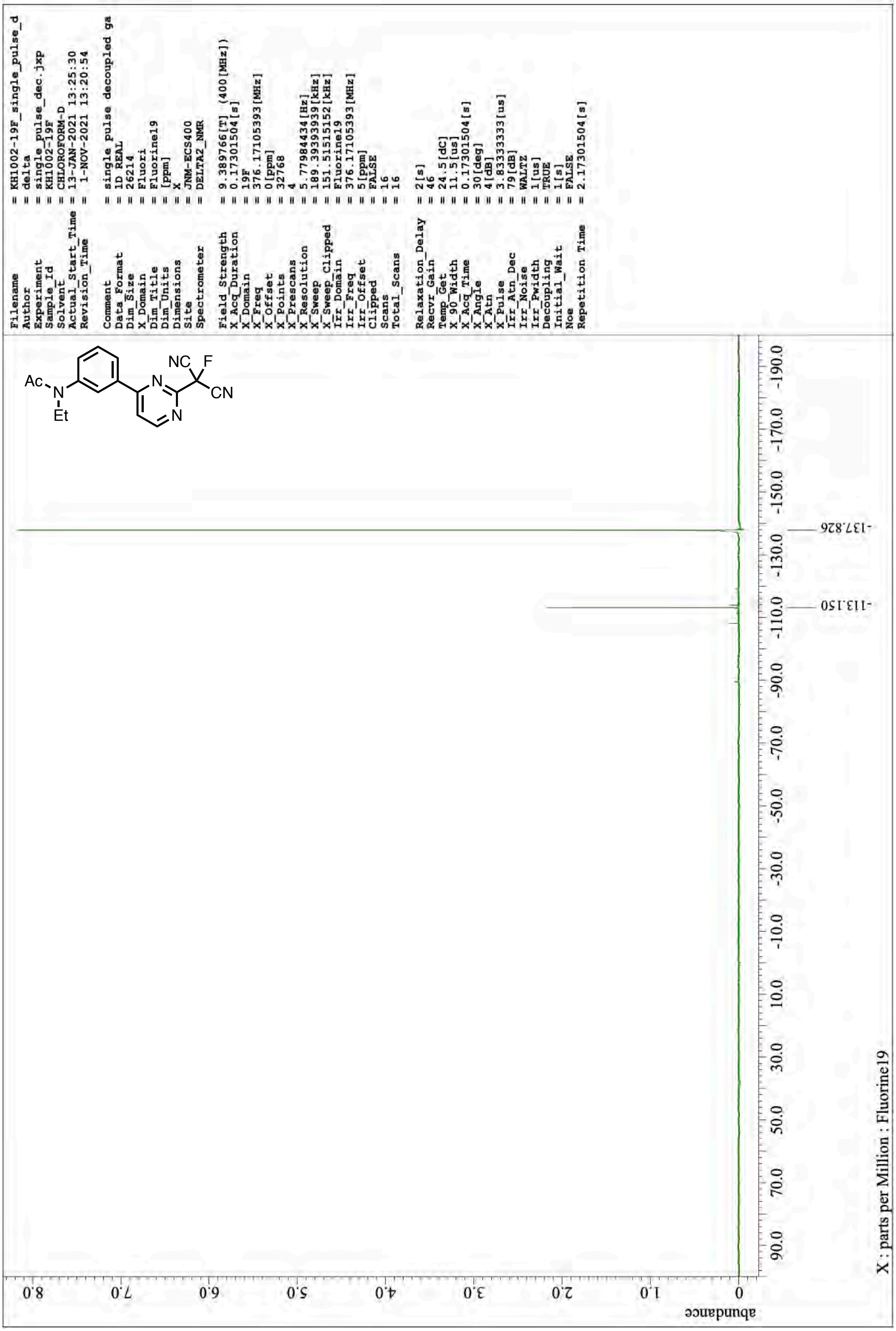

^{1}H NMR of **2AN** (400 MHz, $CDCl_3$)

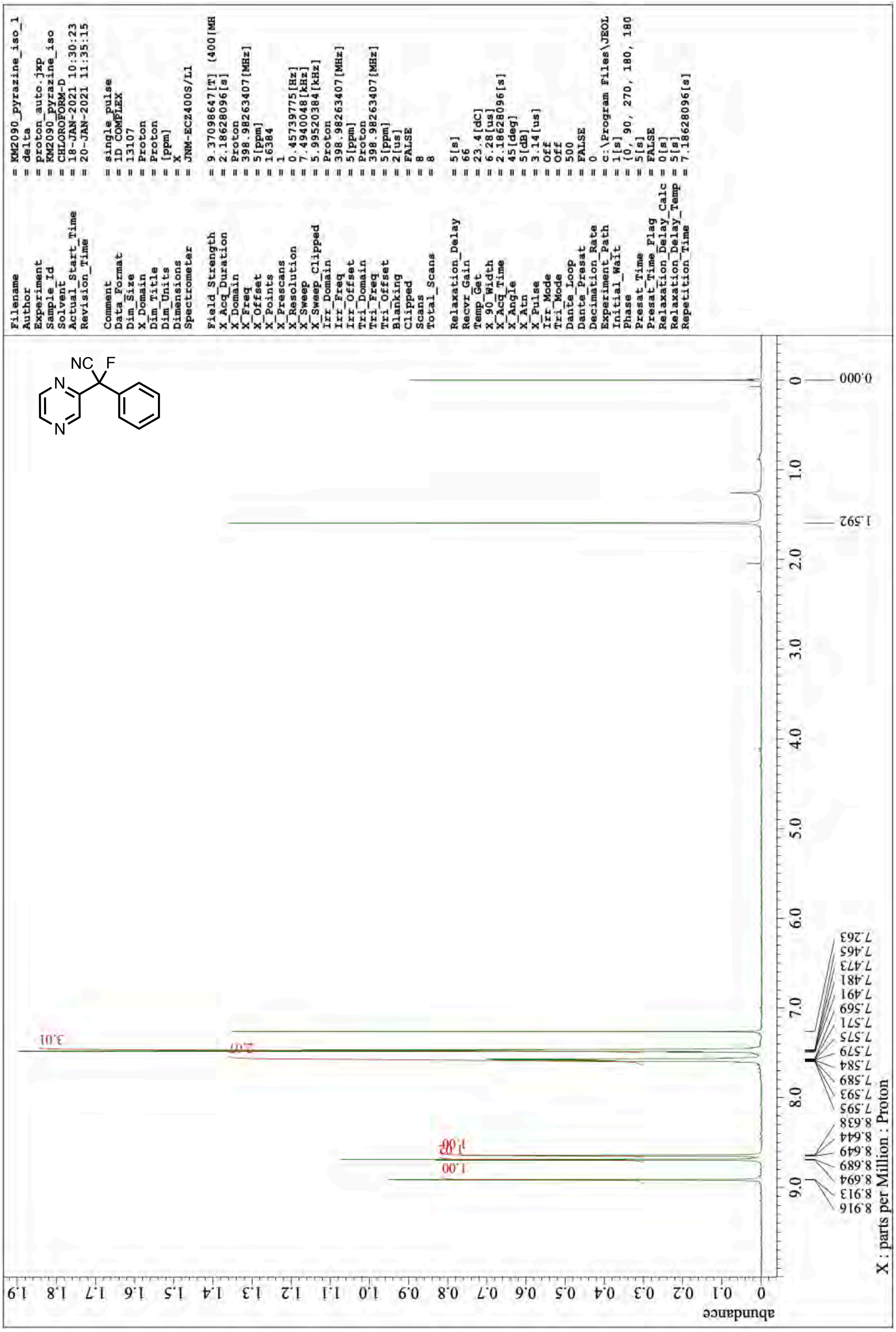

^{13}C NMR of **2AN** (101 MHz, $CDCl_3$)

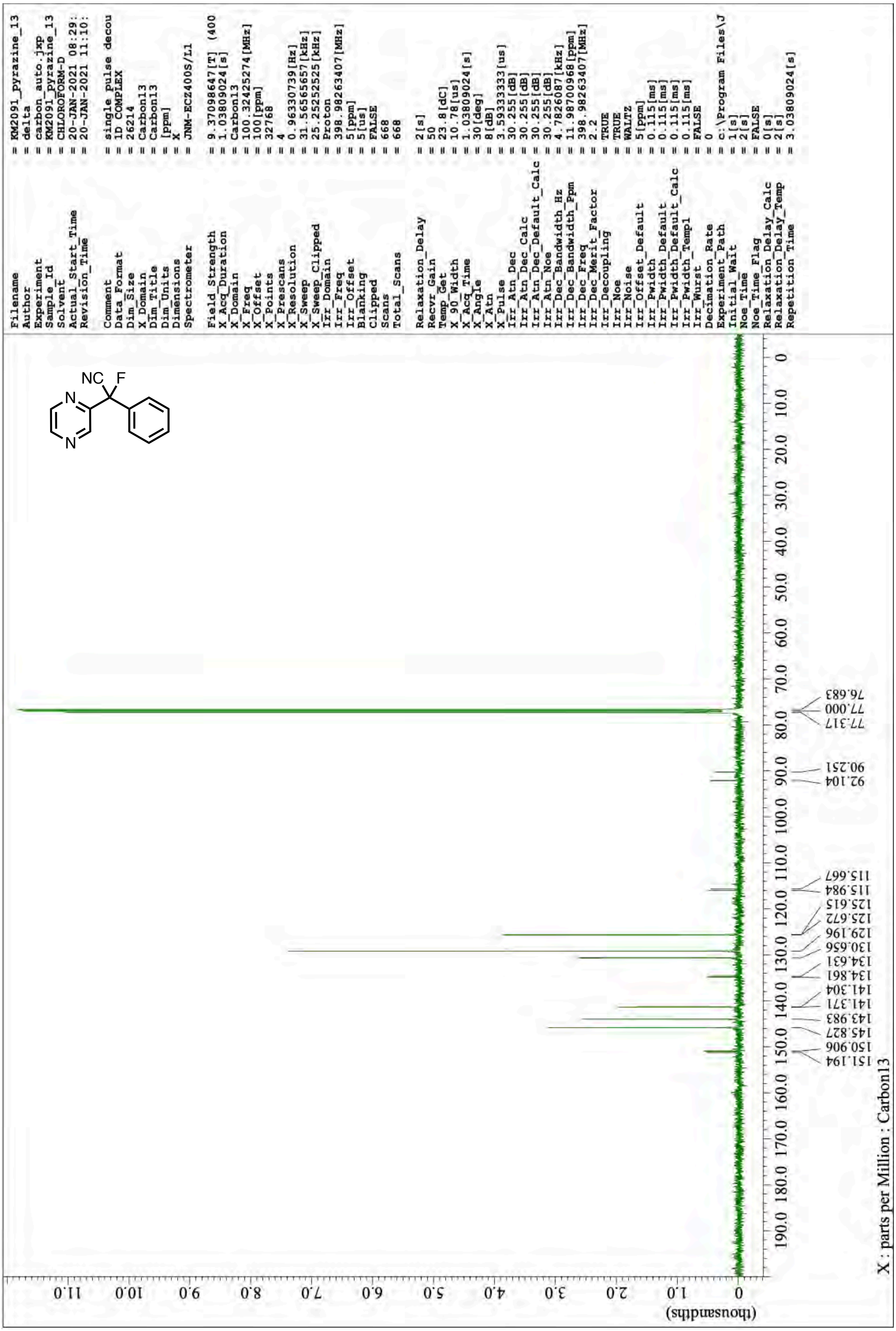

^{19}F NMR of **2AN** (376 MHz, $CDCl_3$)

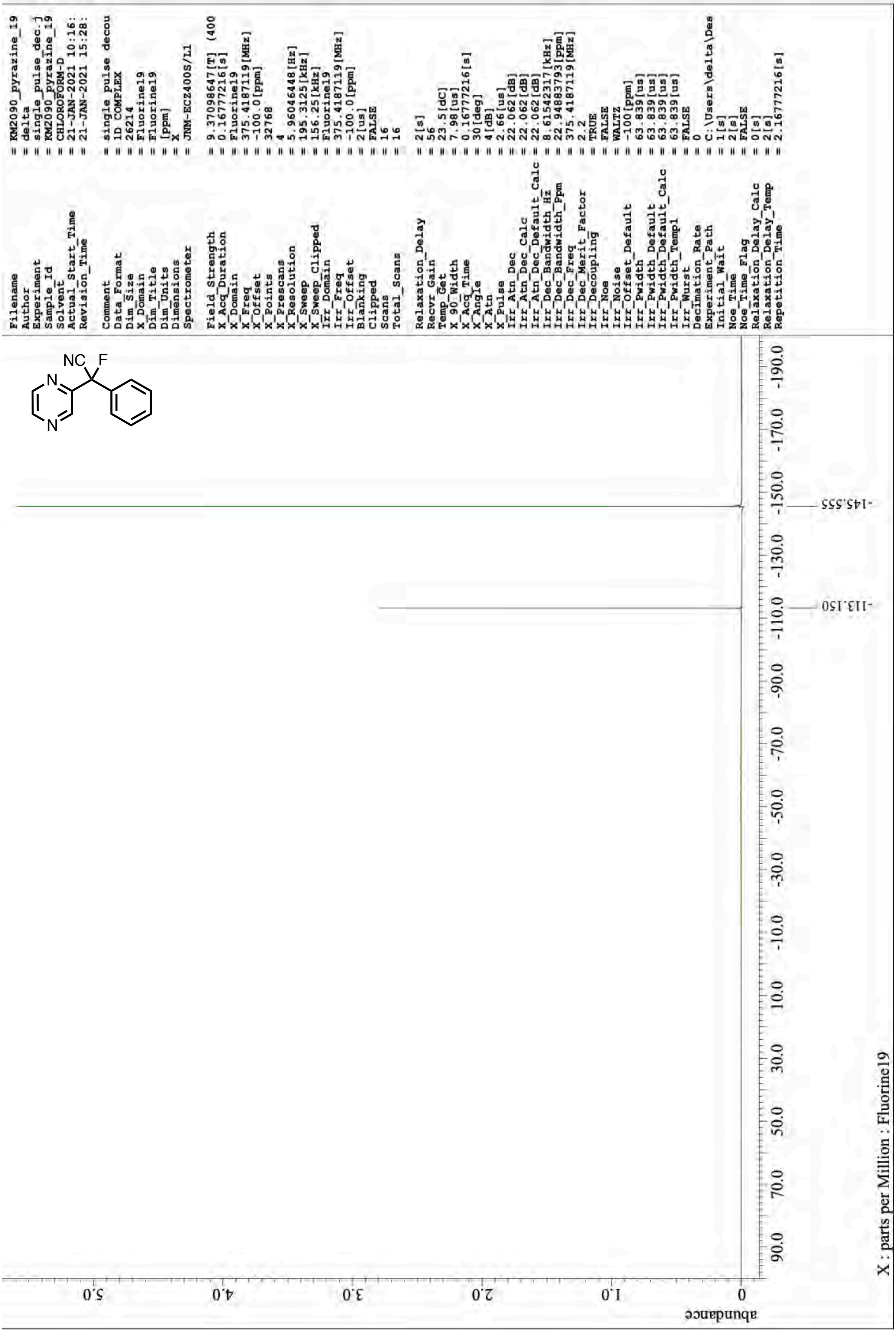

^{1}H NMR of **2AO** (400 MHz, $CDCl_3$)

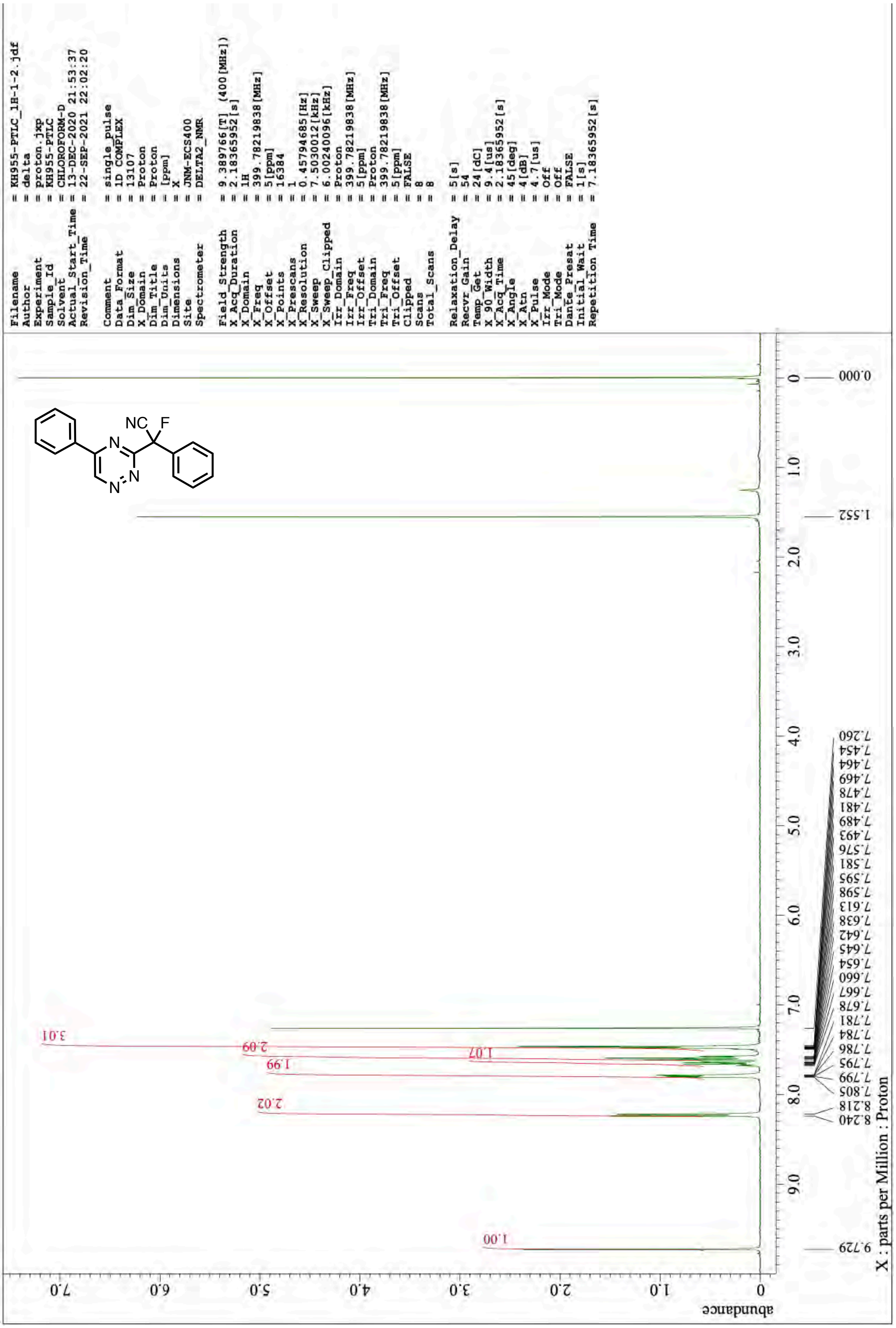

^{13}C NMR of **2AO** (101 MHz, $CDCl_3$)

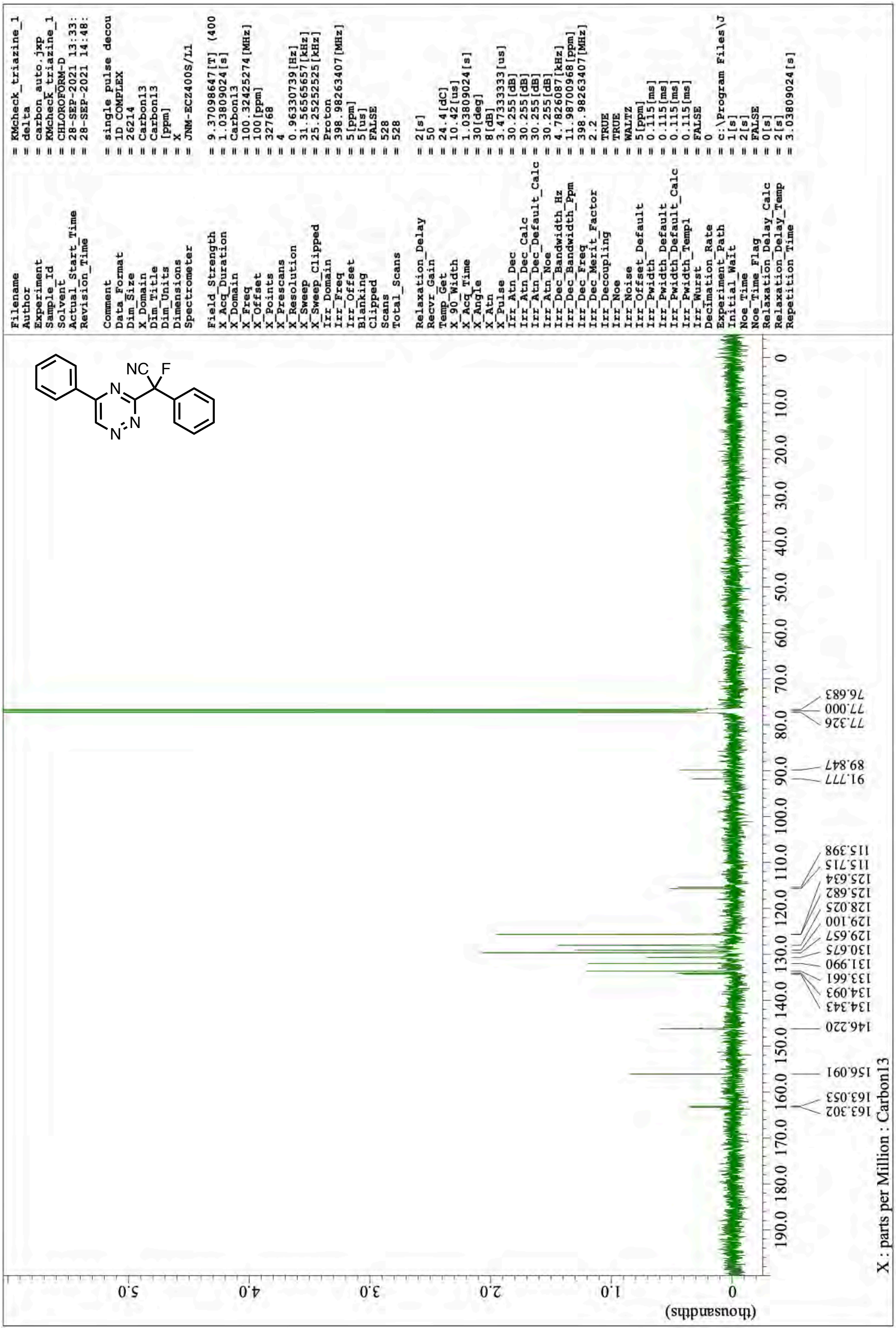

^{19}F NMR of **2AO** (376 MHz, $CDCl_3$)

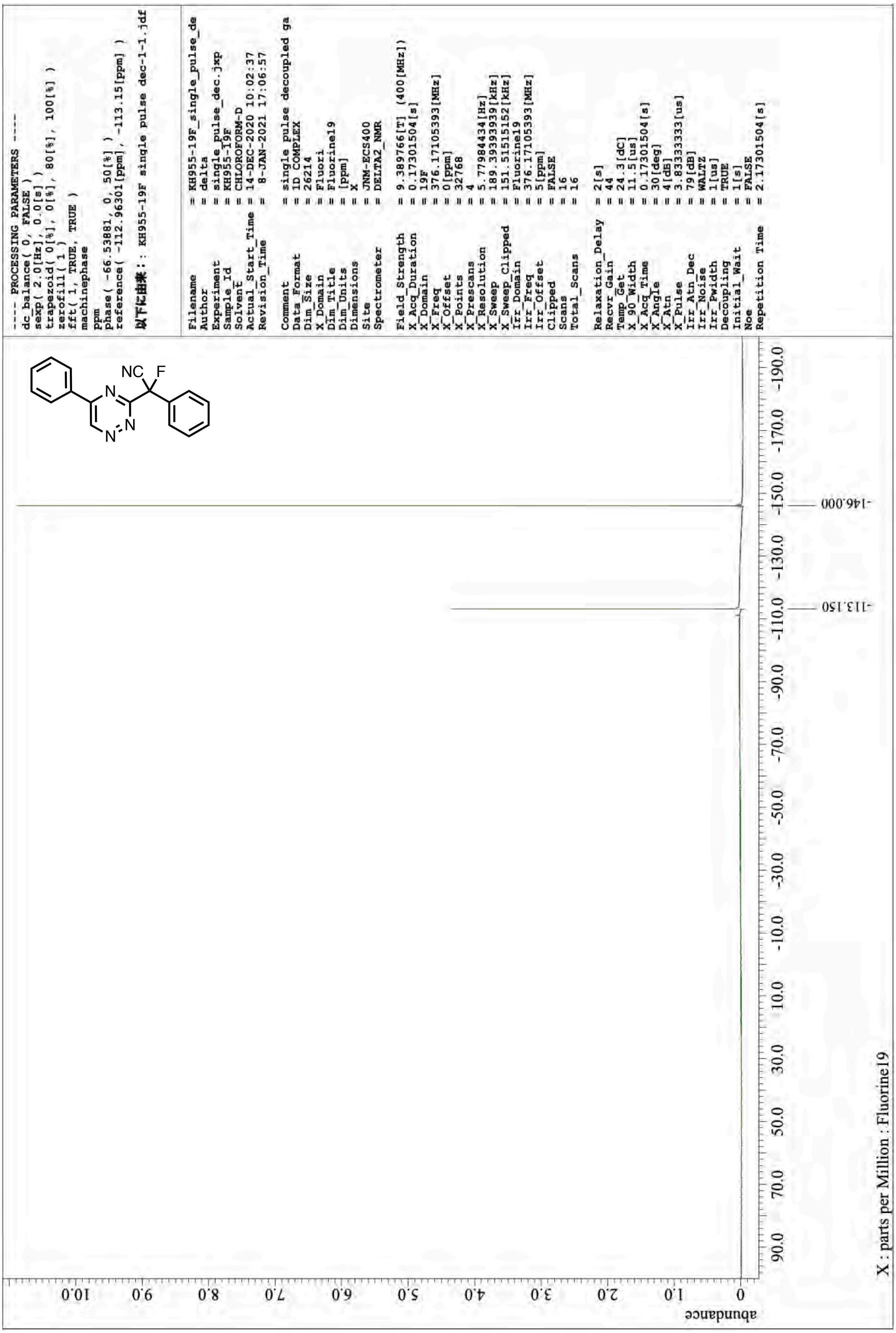

^{1}H NMR of **2AP** (400 MHz, $CDCl_3$)

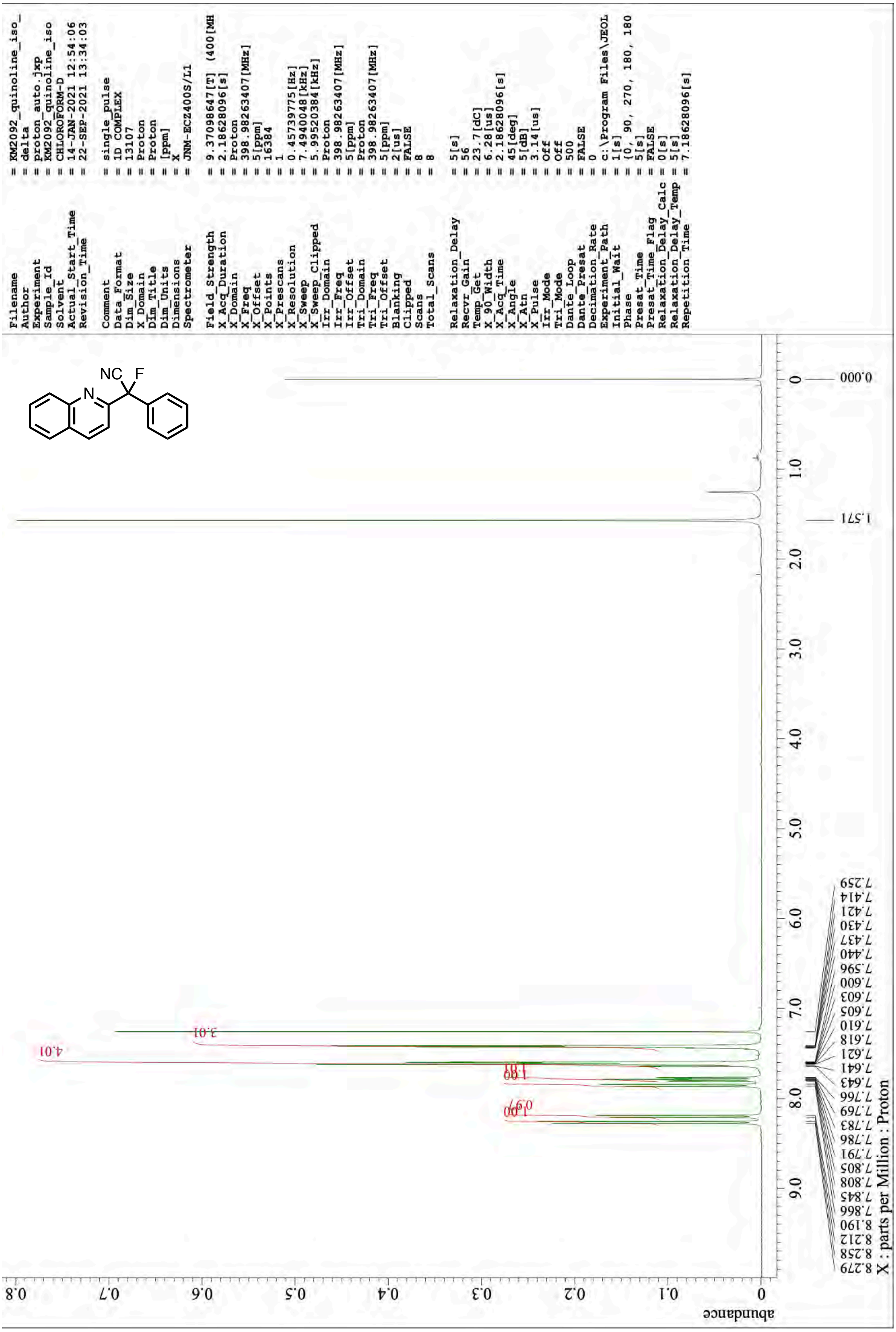

^{13}C NMR of **2AP** (101 MHz, $CDCl_3$)

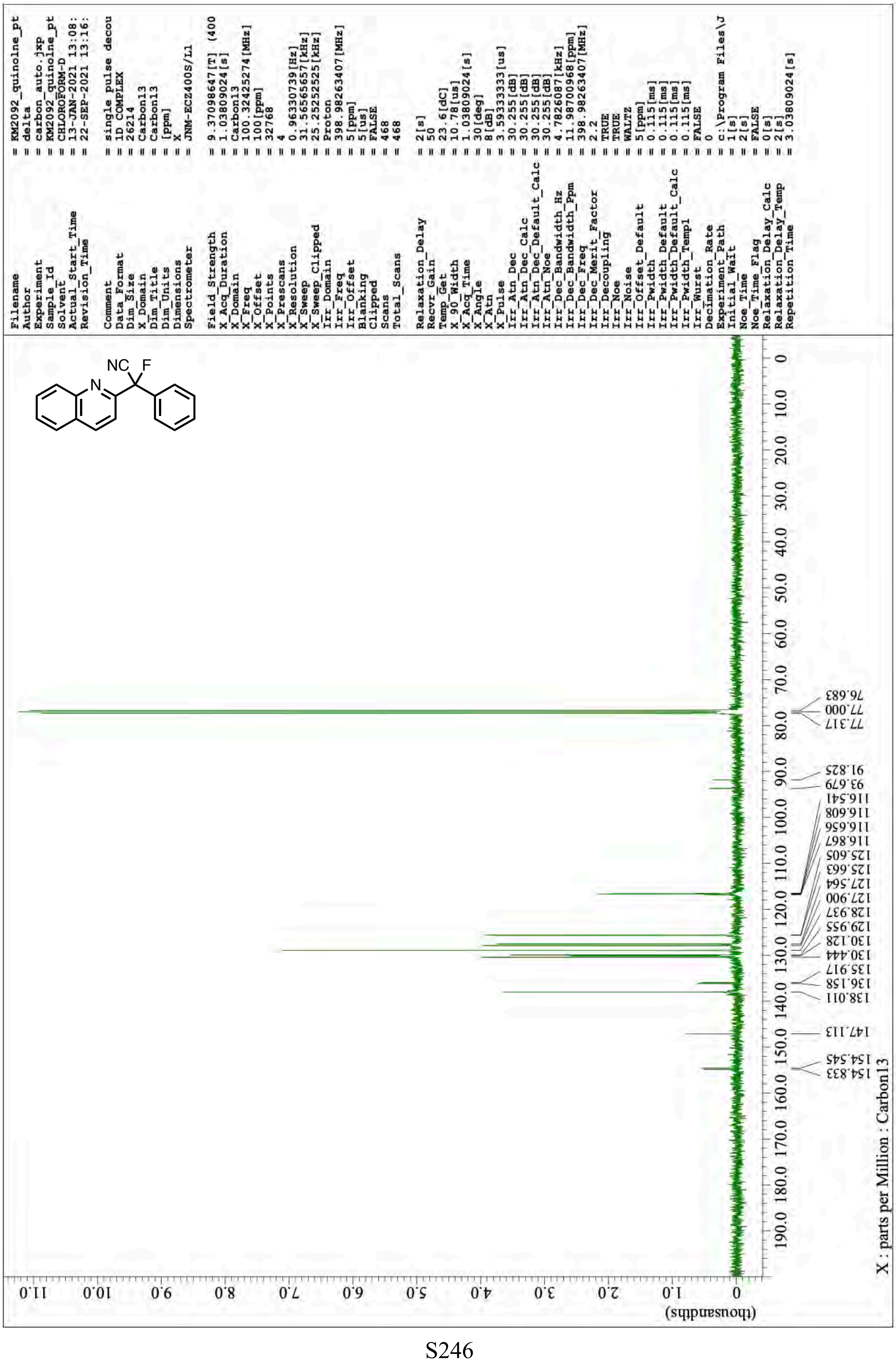

^{19}F NMR of **2AP** (376 MHz, $CDCl_3$)

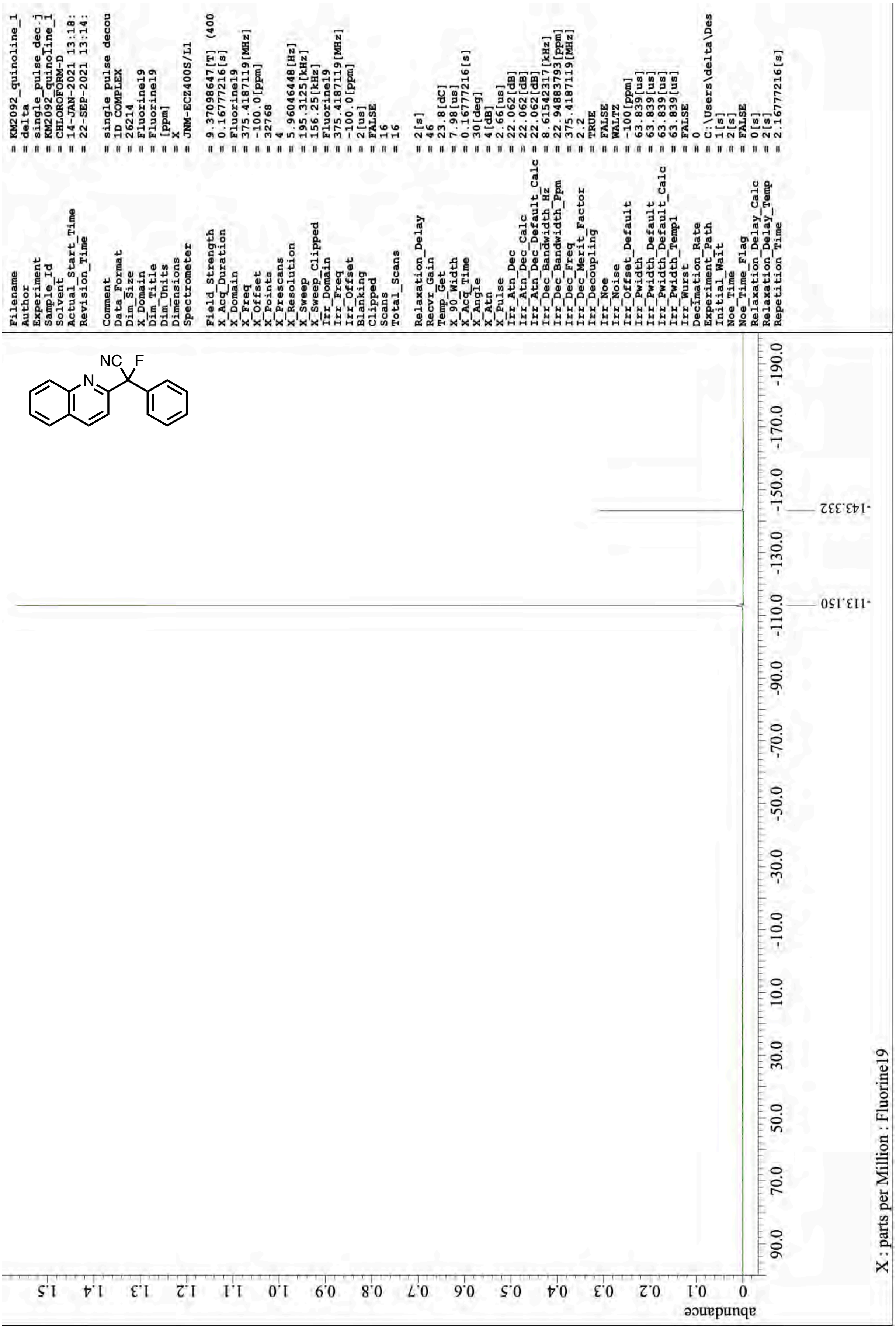

^{1}H NMR of **2AQ** (400 MHz, $CDCl_3$)

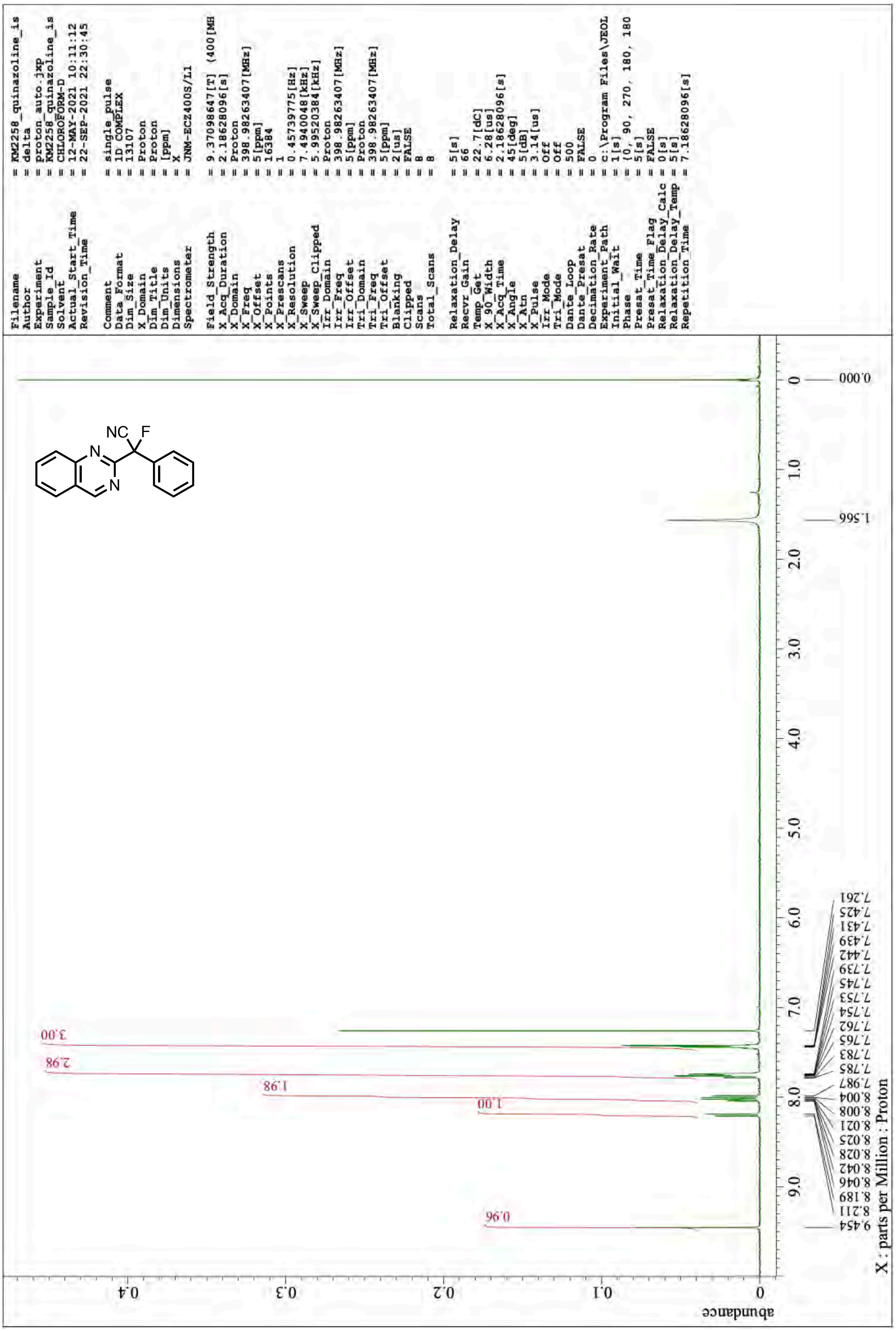

^{13}C NMR of **2AQ** (101 MHz, $CDCl_3$)

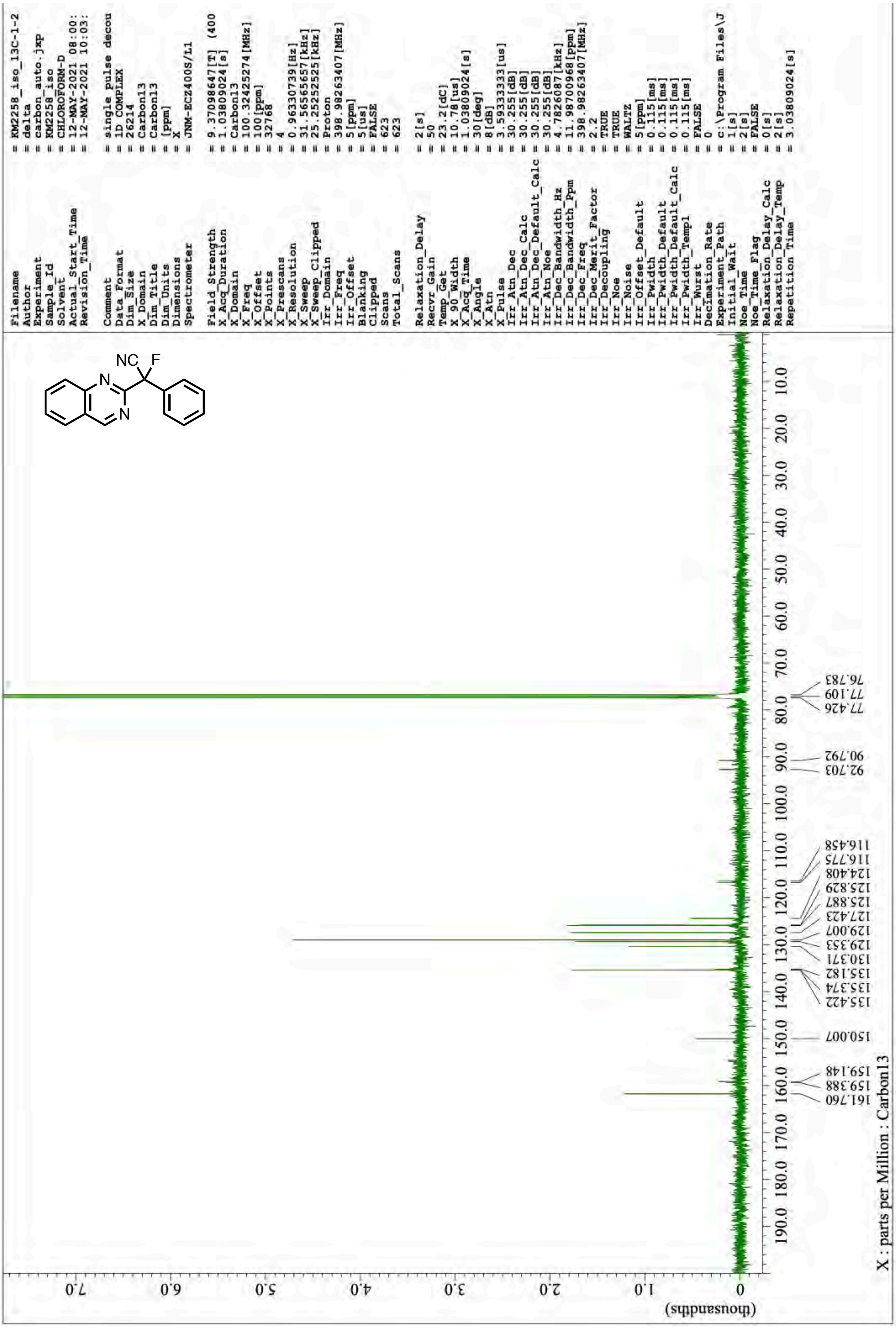

^{19}F NMR of **2AQ** (376 MHz, $CDCl_3$)

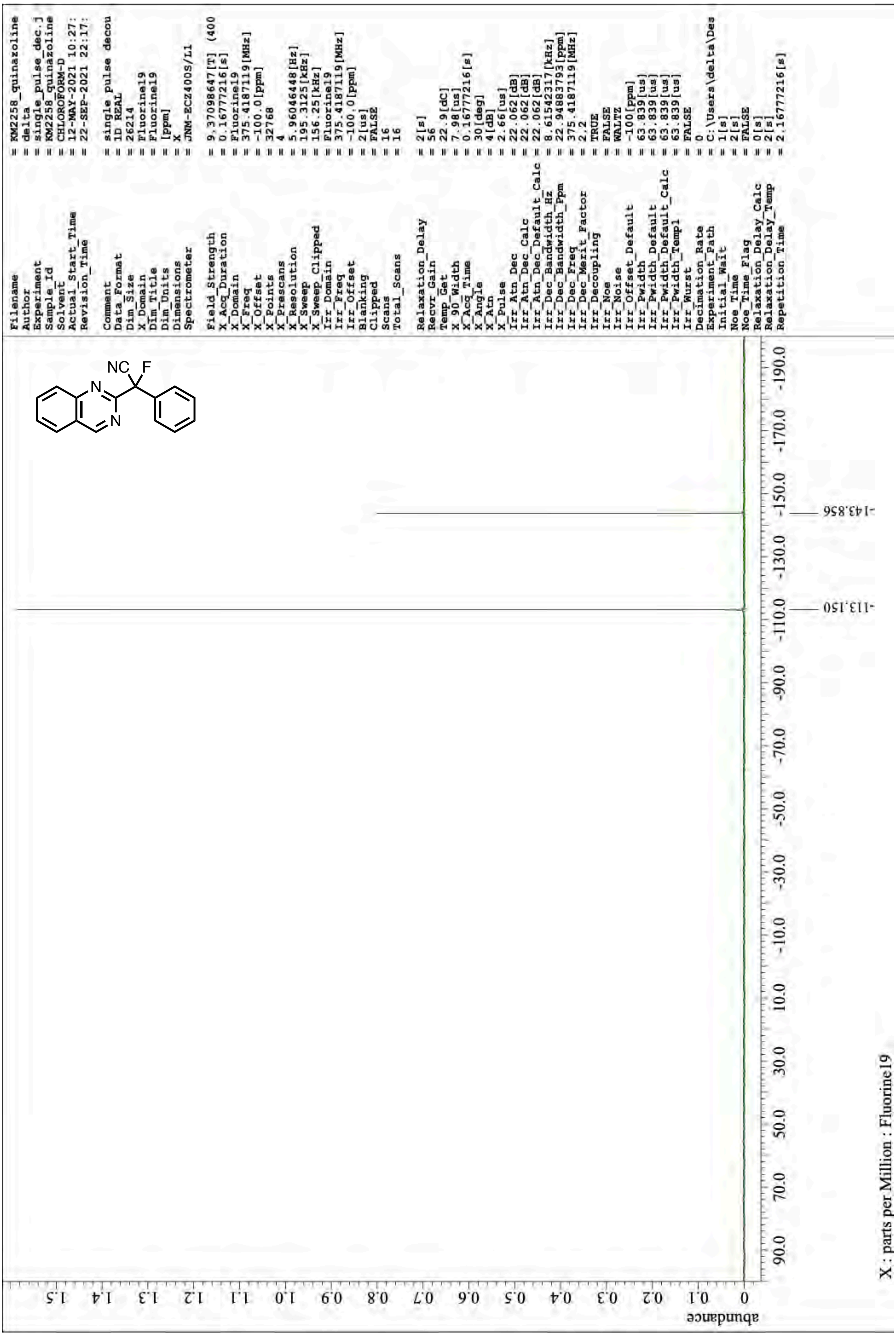

^{1}H NMR of **6** (400 MHz, acetonitrile-d_3)

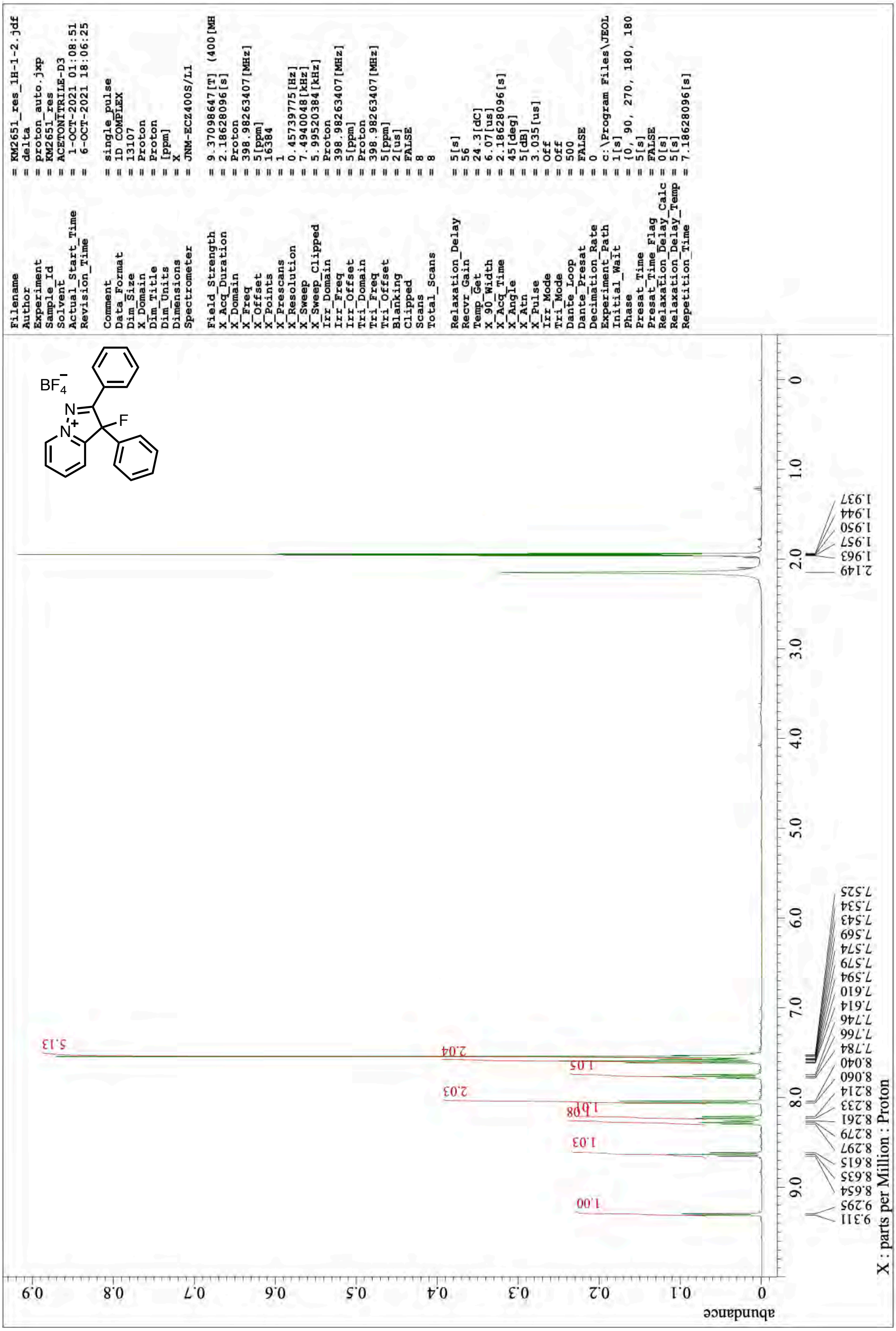

^{13}C NMR of **6** (101 MHz, acetonitrile-d_3)

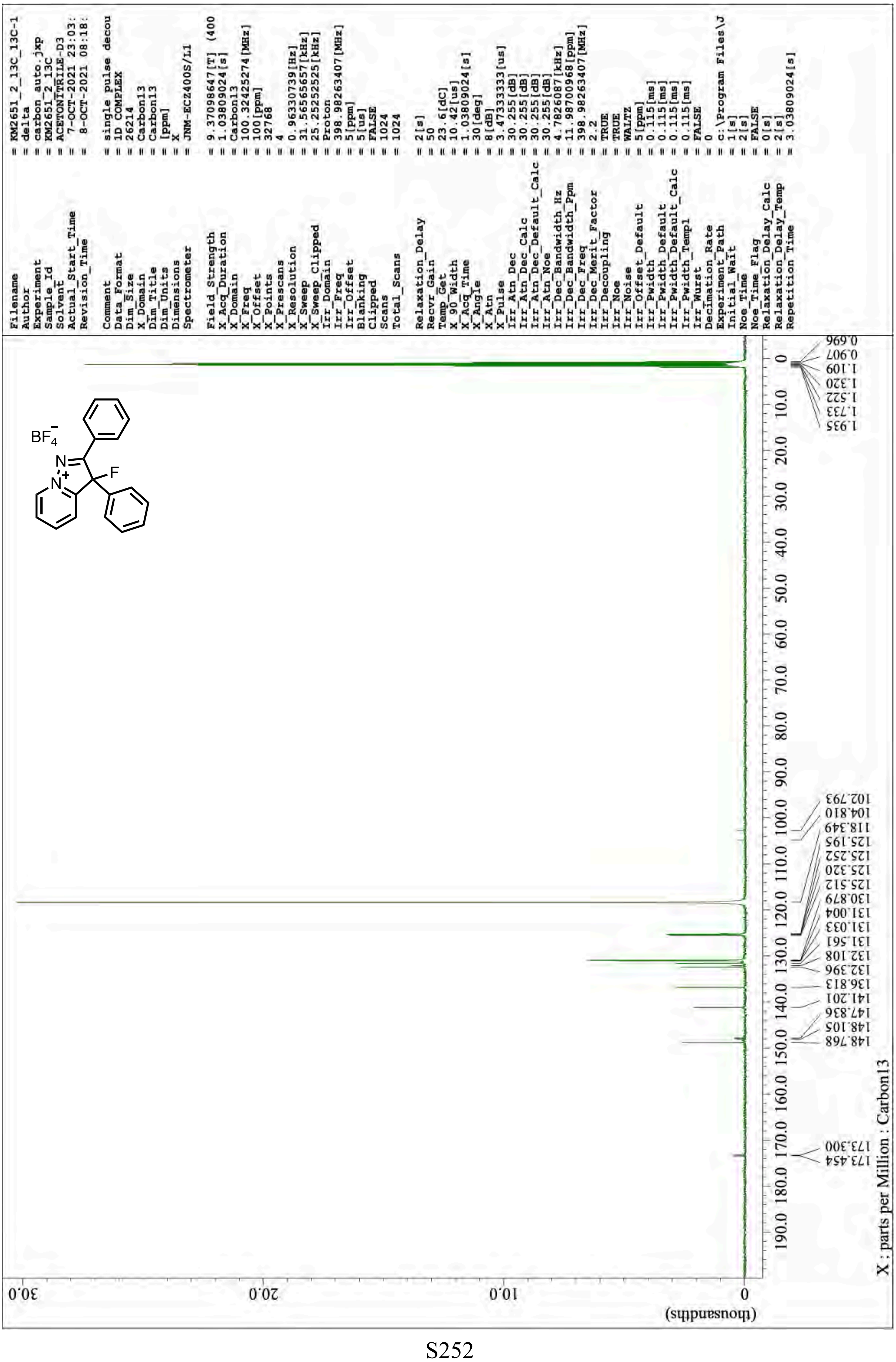

^{19}F NMR of **6** (376 MHz, acetonitrile-d_3)

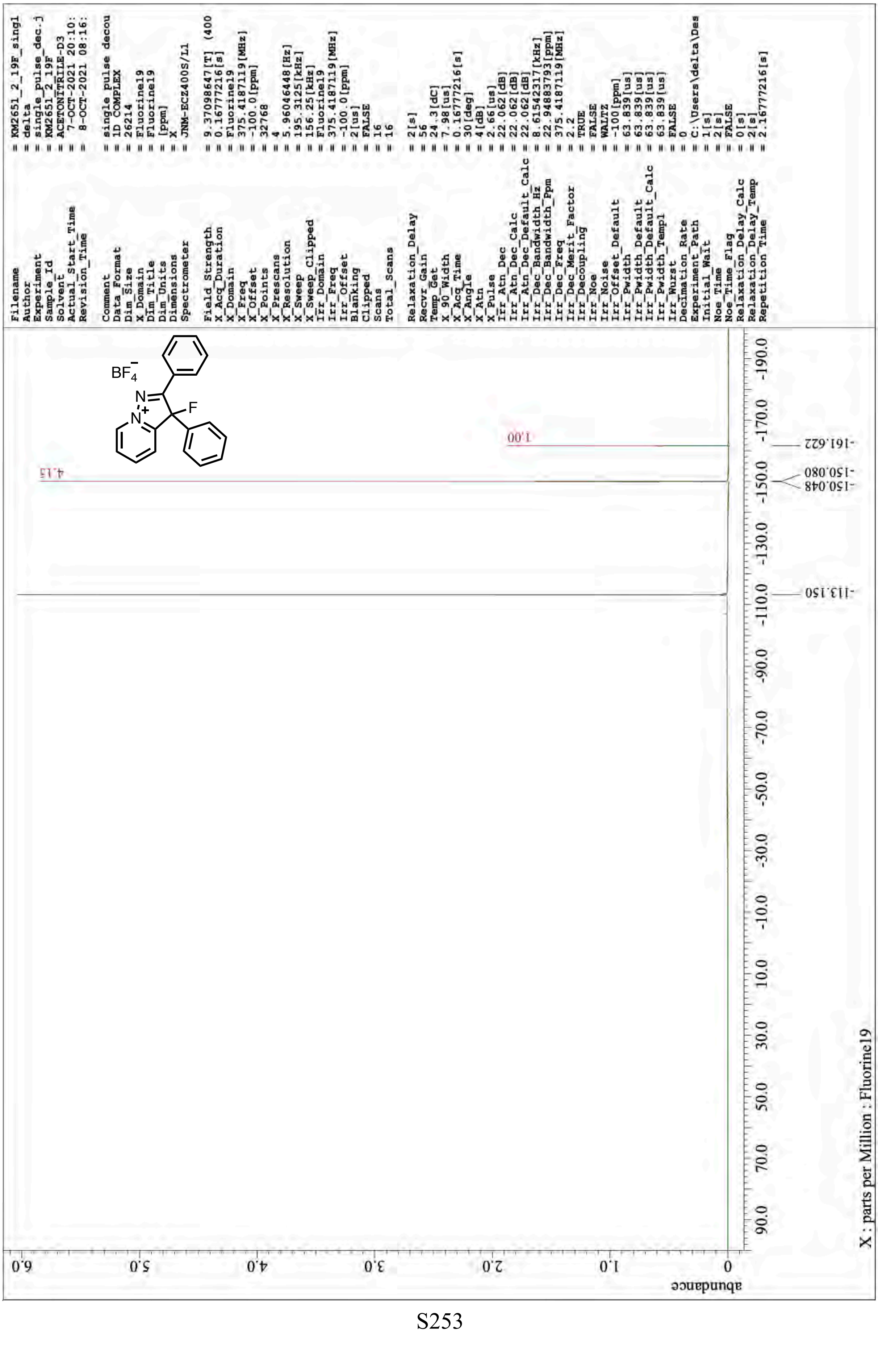

^1H NMR of **8** (400 MHz, $CDCl_3$)

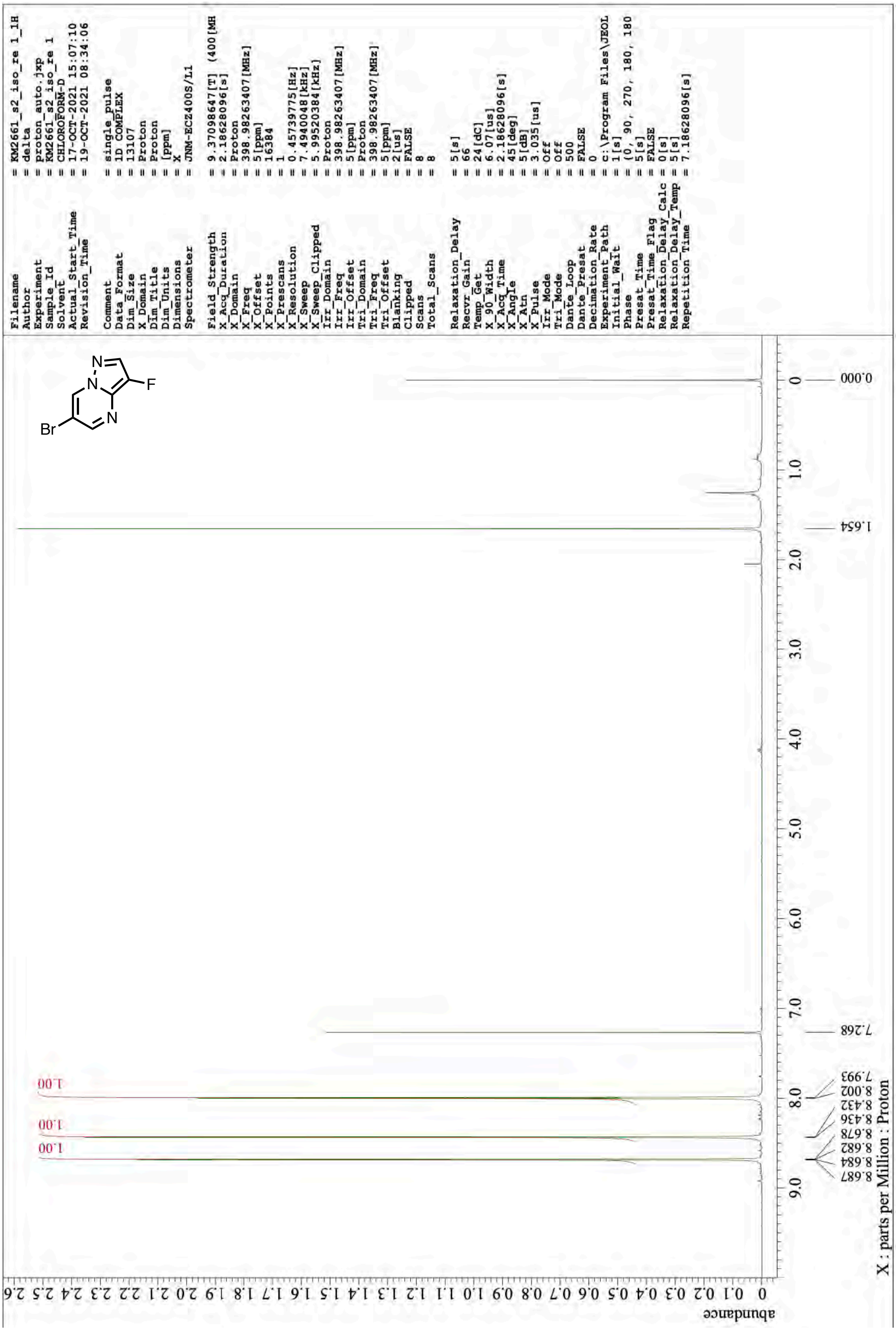

^{13}C NMR of **8** (101 MHz, $CDCl_3$)

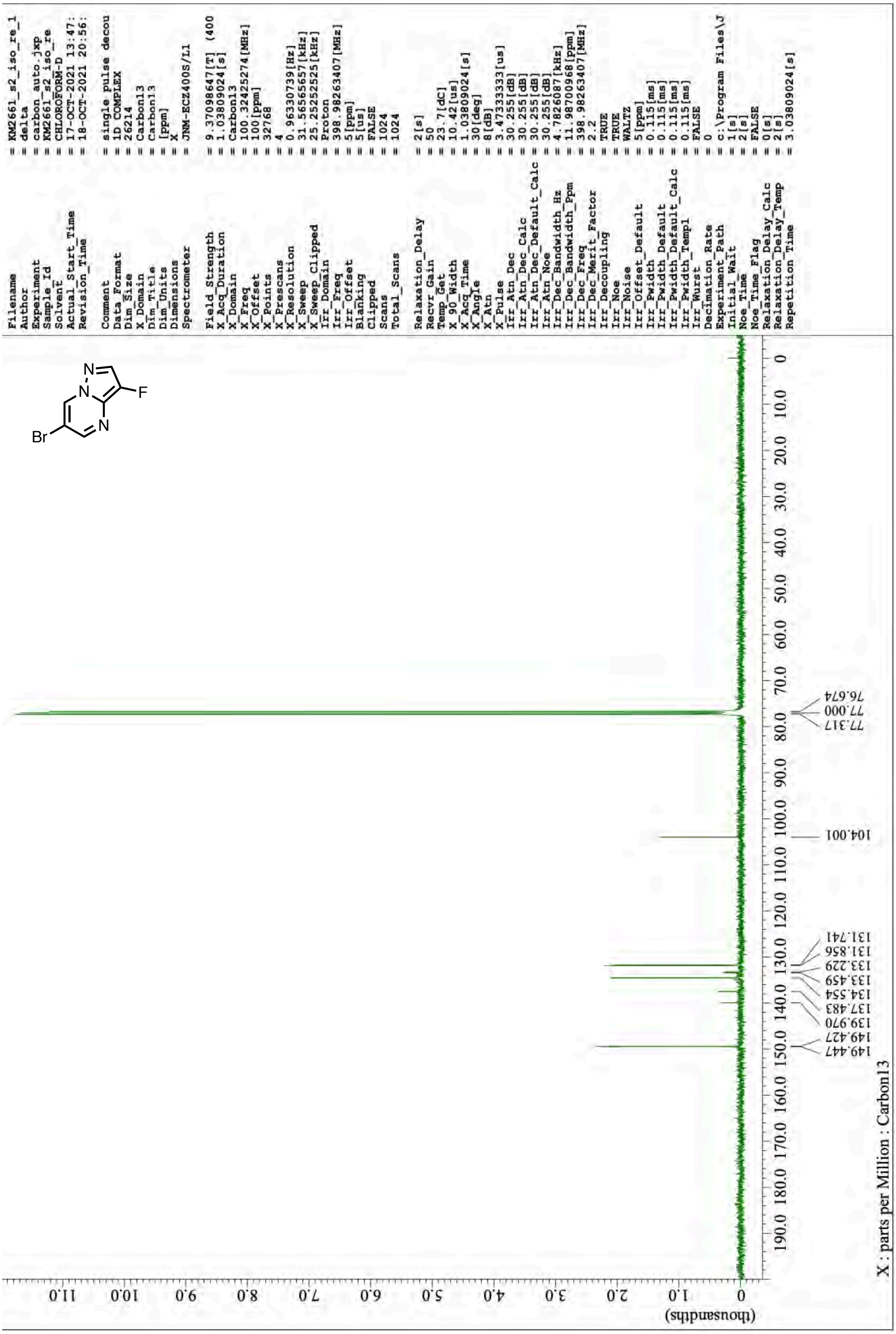

^{19}F NMR of **8** (376 MHz, $CDCl_3$)

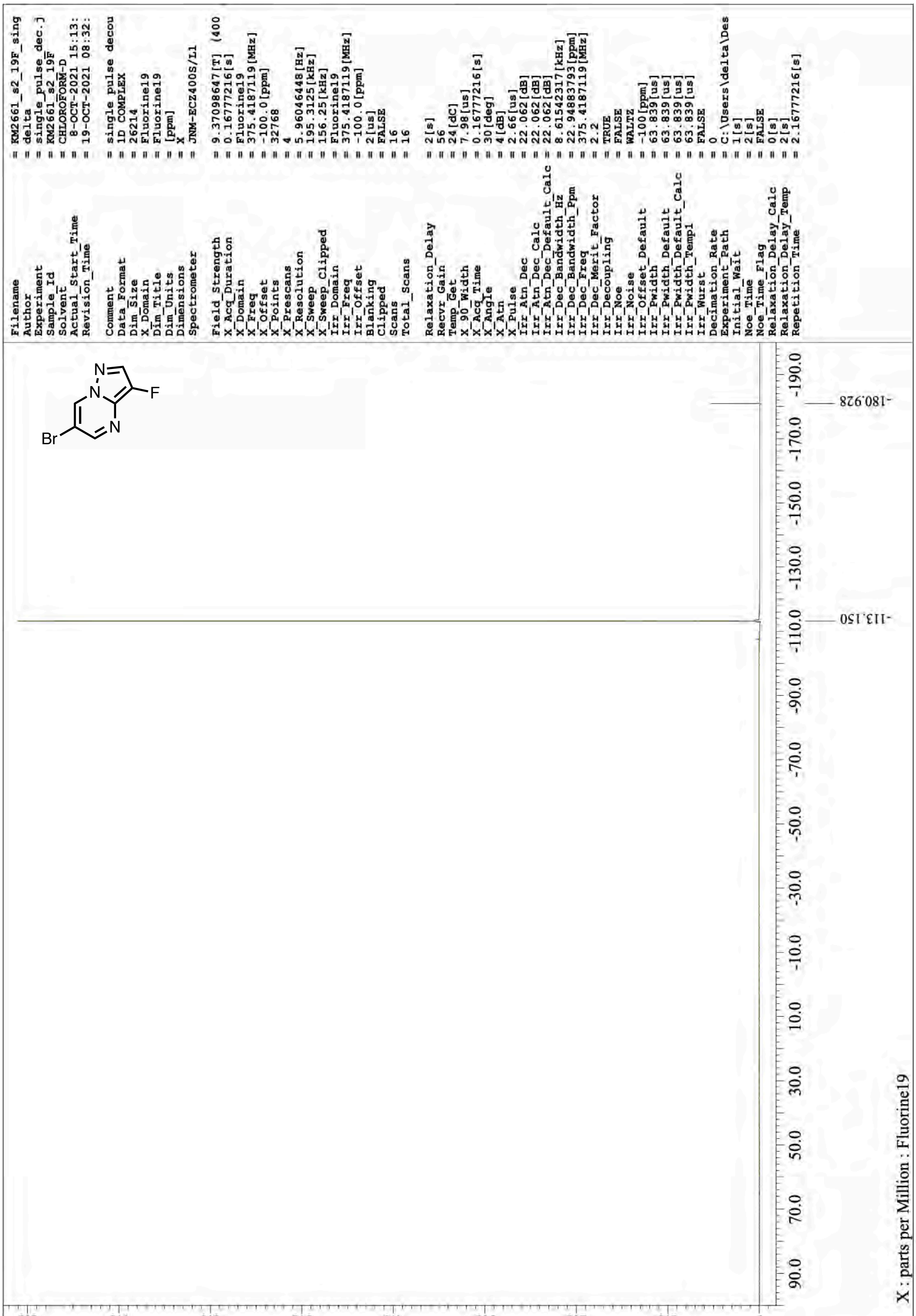

^{1}H NMR of **9** (400 MHz, $CDCl_3$)

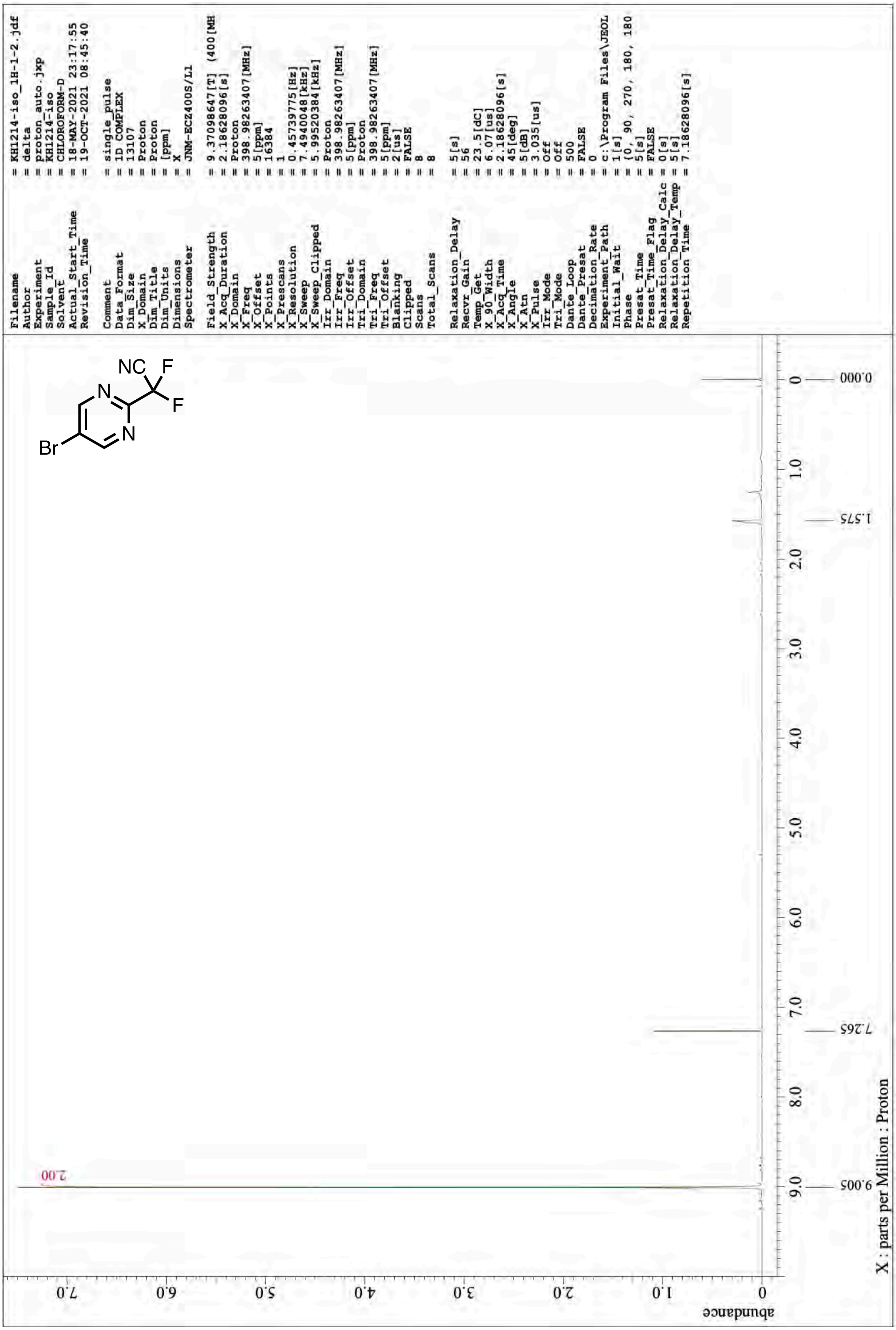

^{13}C NMR of **9** (101 MHz, $CDCl_3$)

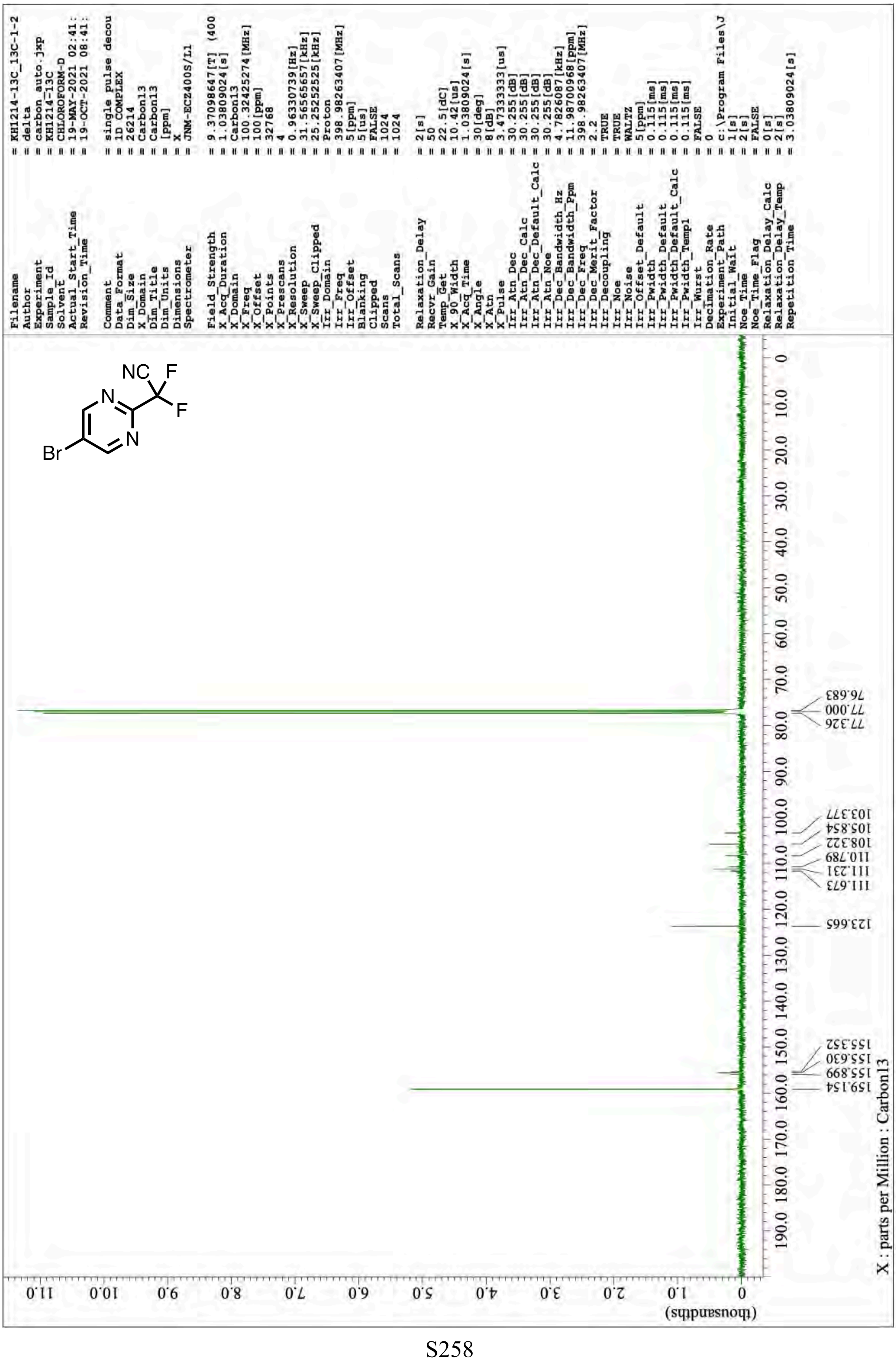

^{19}F NMR of **9** (376 MHz, $CDCl_3$)

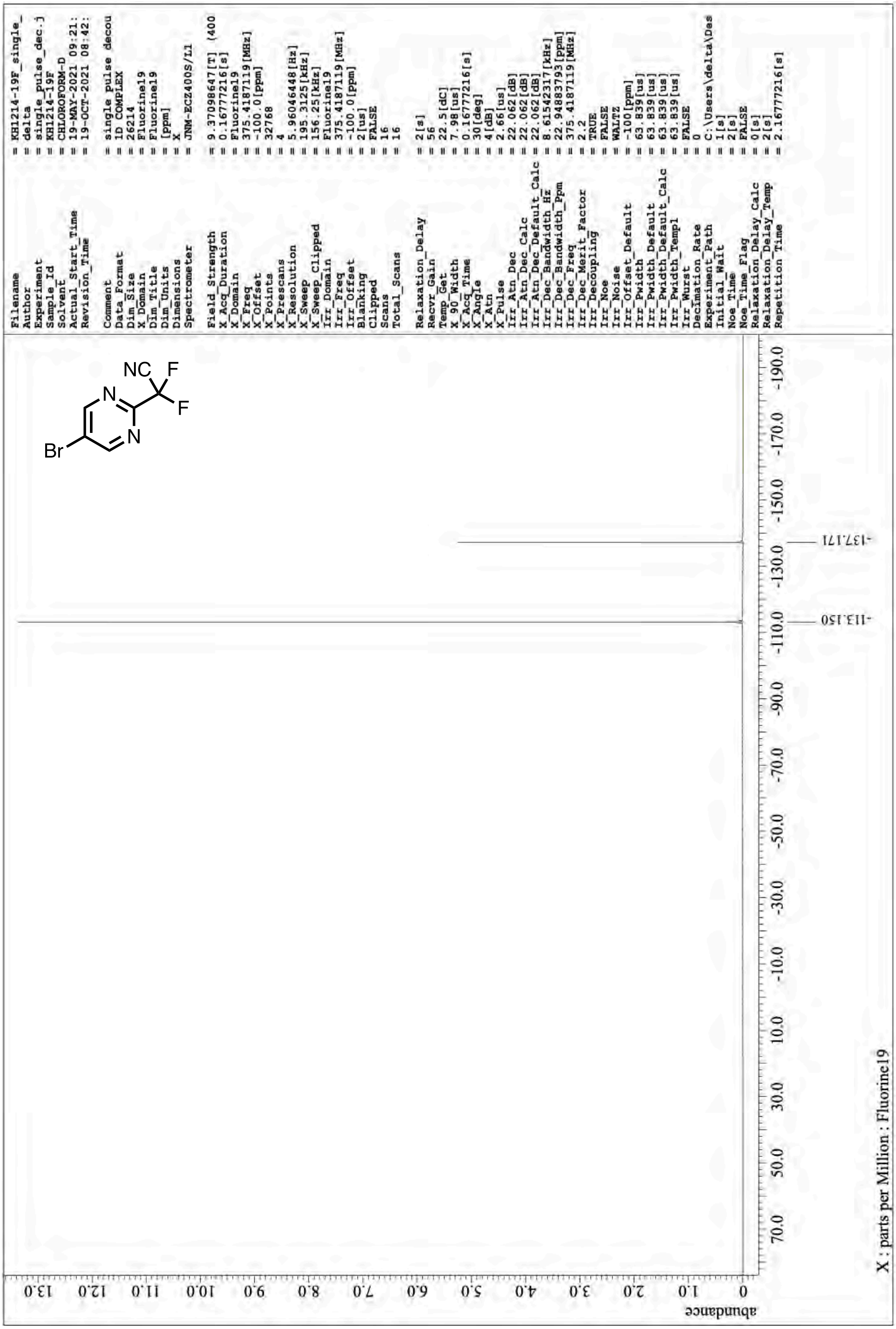

^{1}H NMR of **10** (400 MHz, $CDCl_3$)

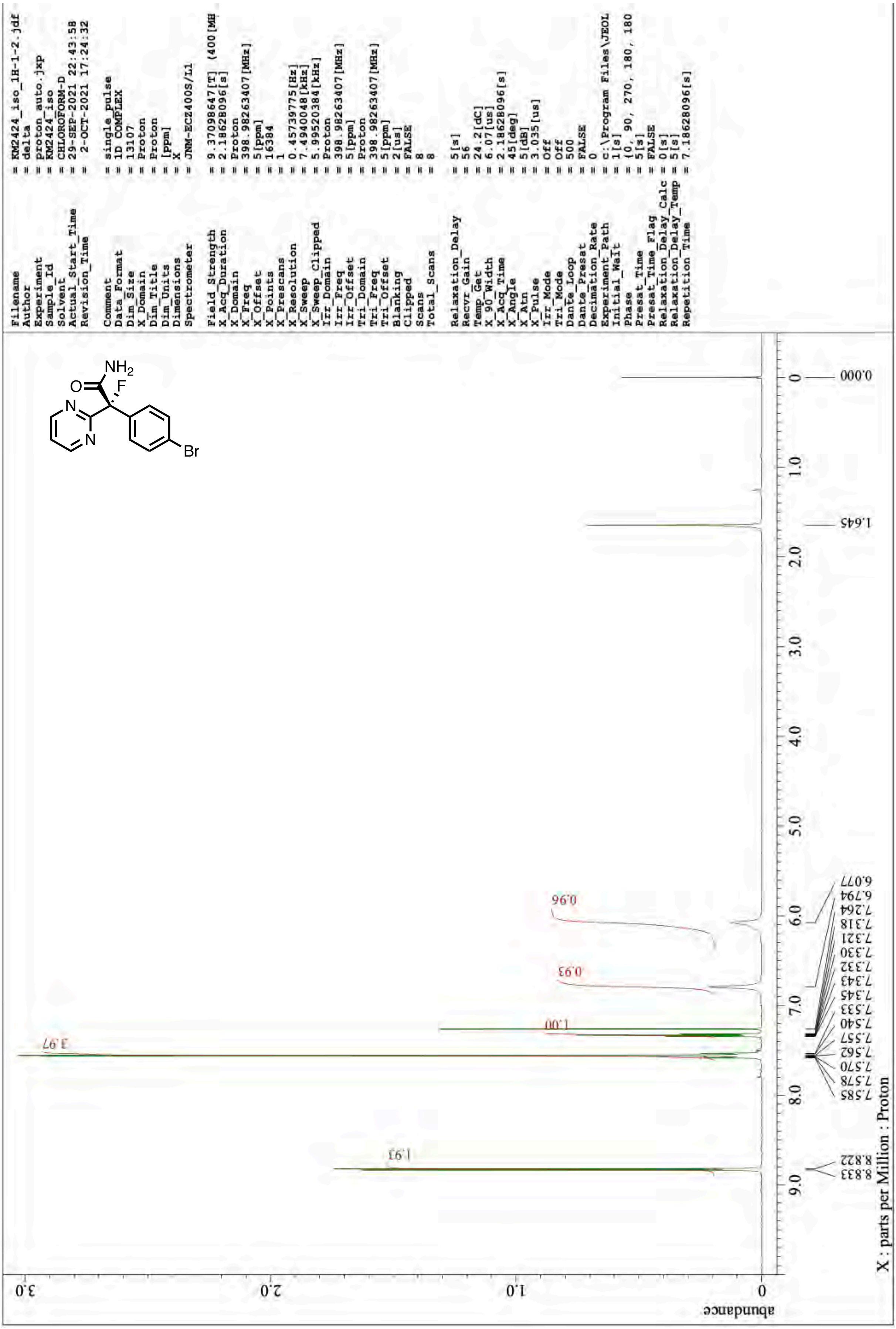

^{13}C NMR of **10** (101 MHz, $CDCl_3$)

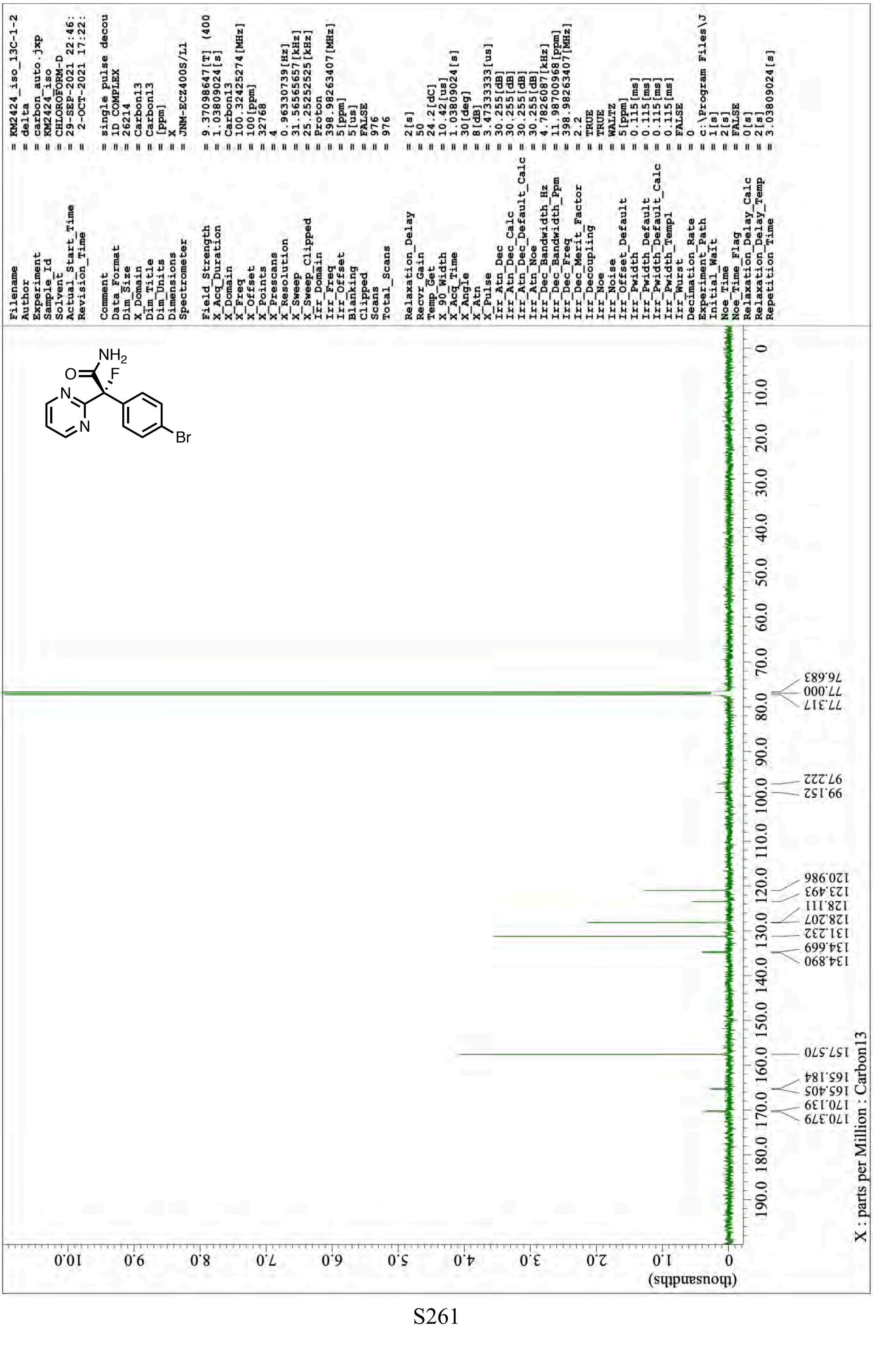

^{19}F NMR of **10** (376 MHz, $CDCl_3$)

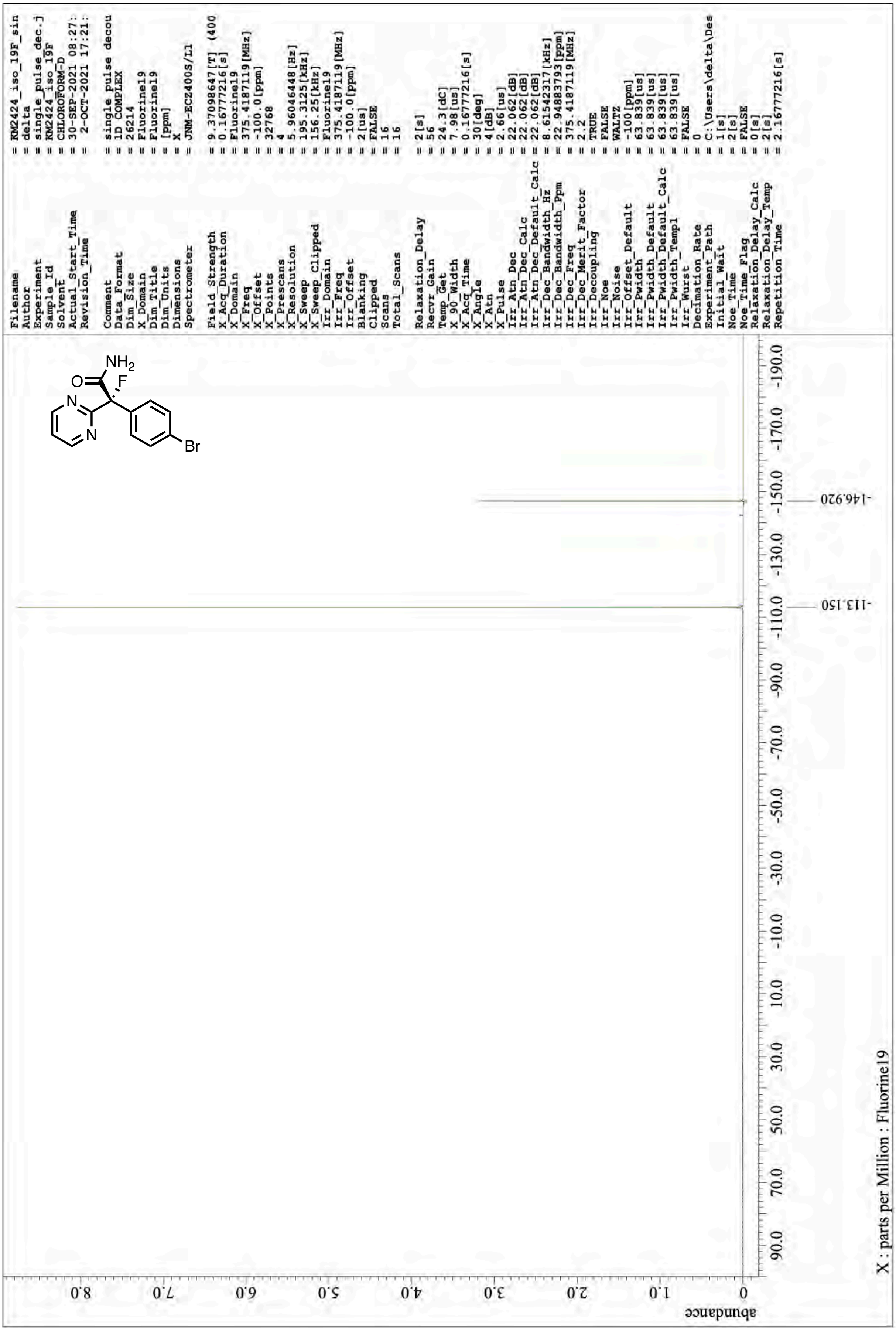

^{1}H NMR of **11** (400 MHz, $CDCl_3$)

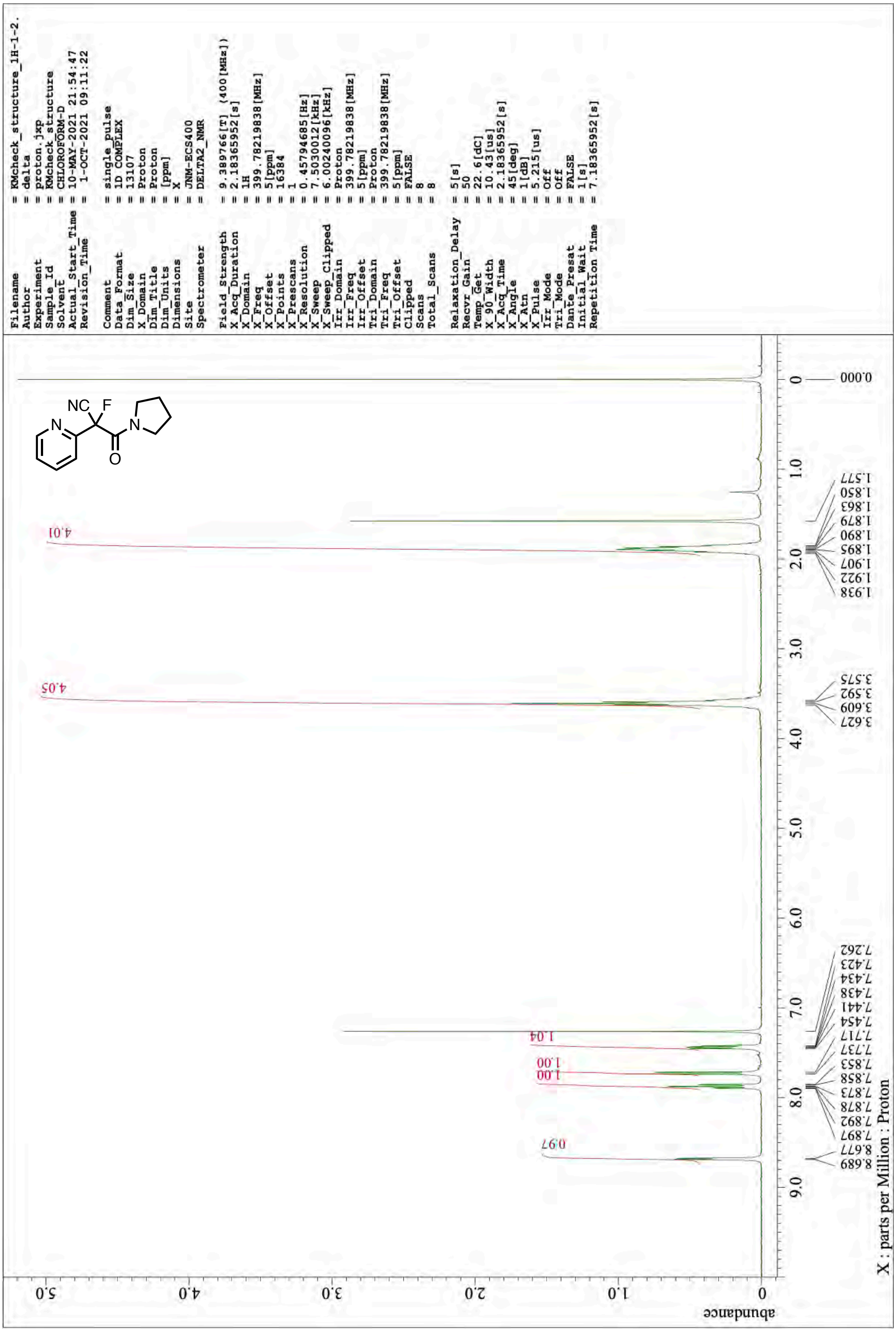

^{13}C NMR of **11** (101 MHz, $CDCl_3$)

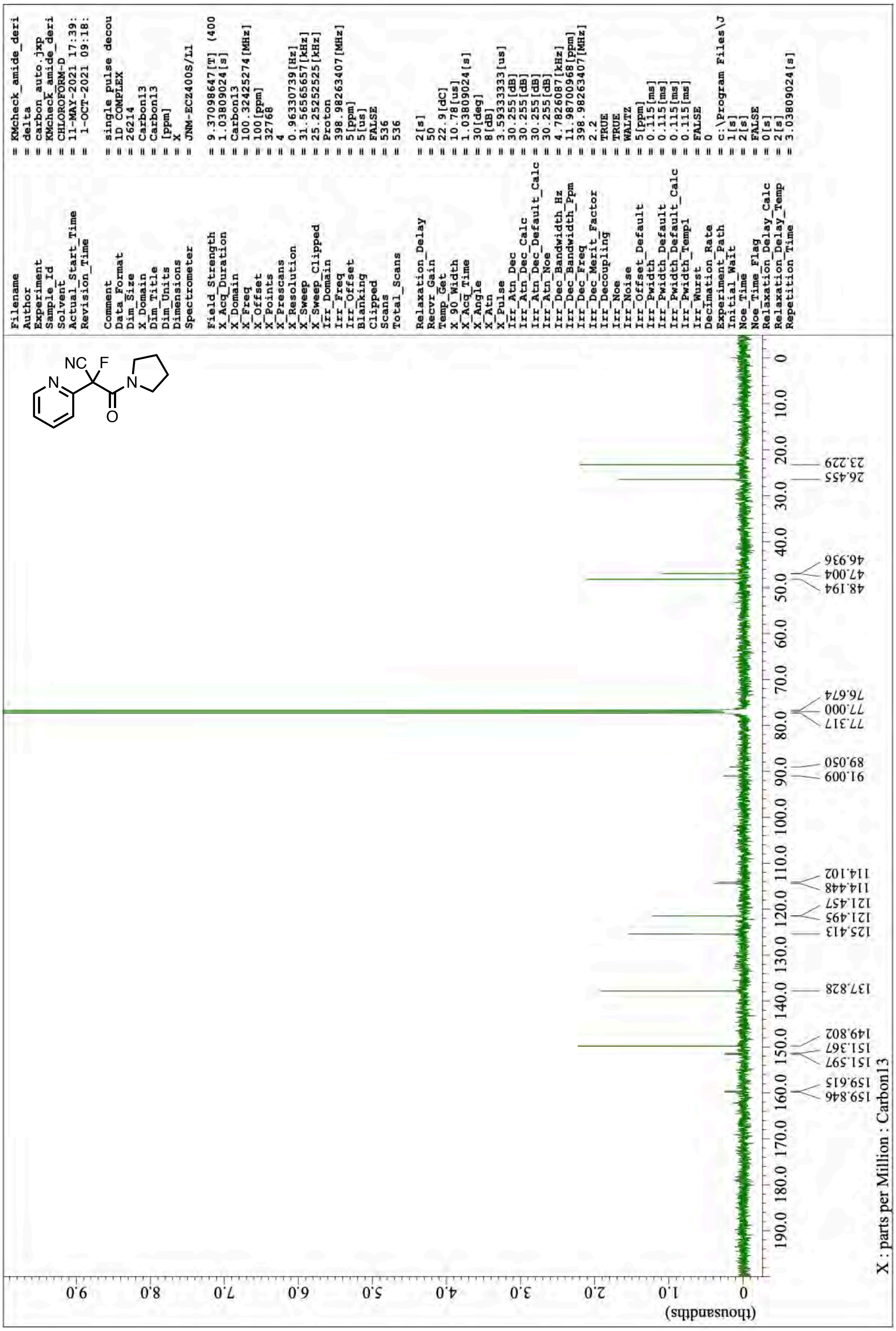

^{19}F NMR of **11** (376 MHz, $CDCl_3$)

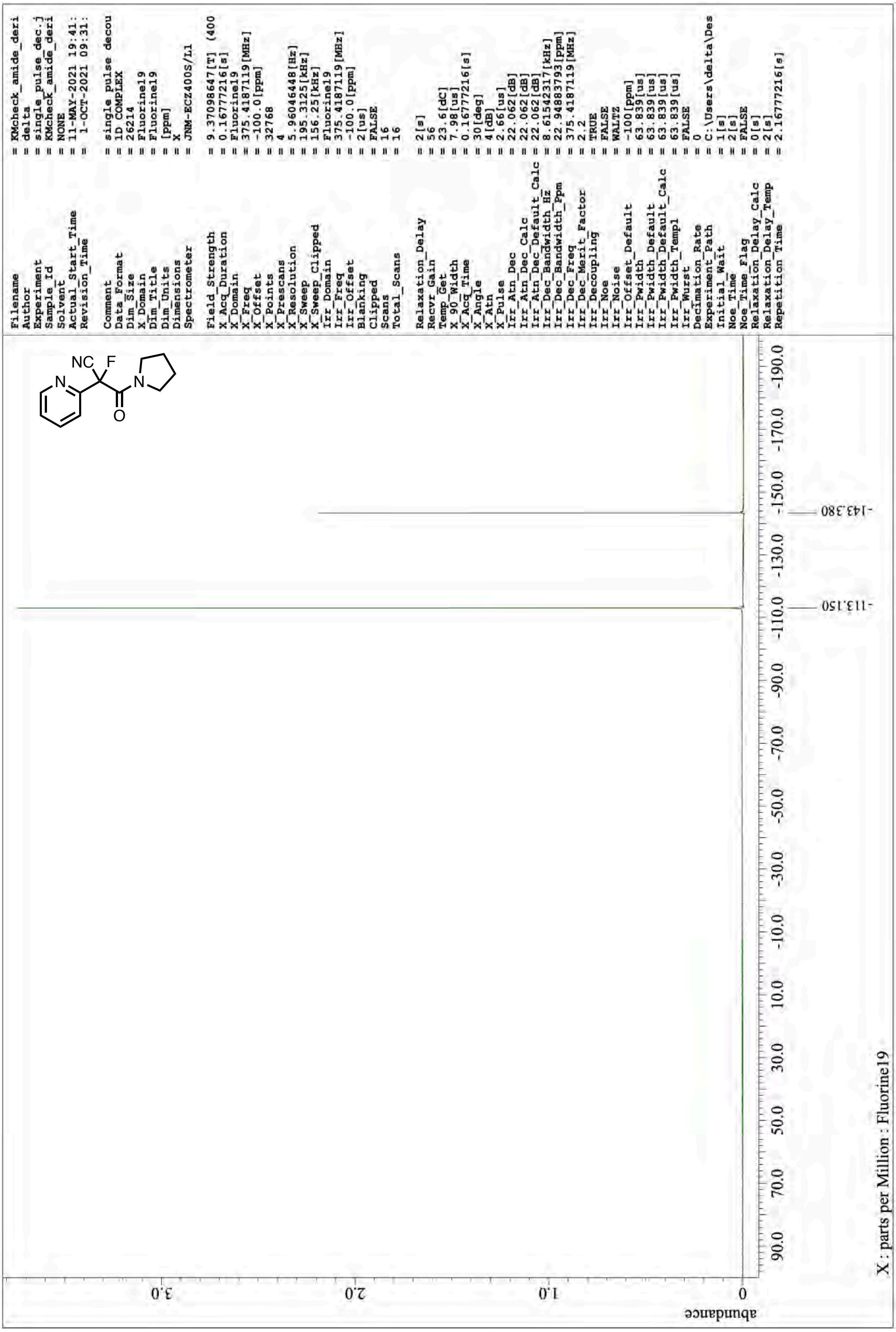

^{1}H NMR of **12** (400 MHz, $CDCl_3$)

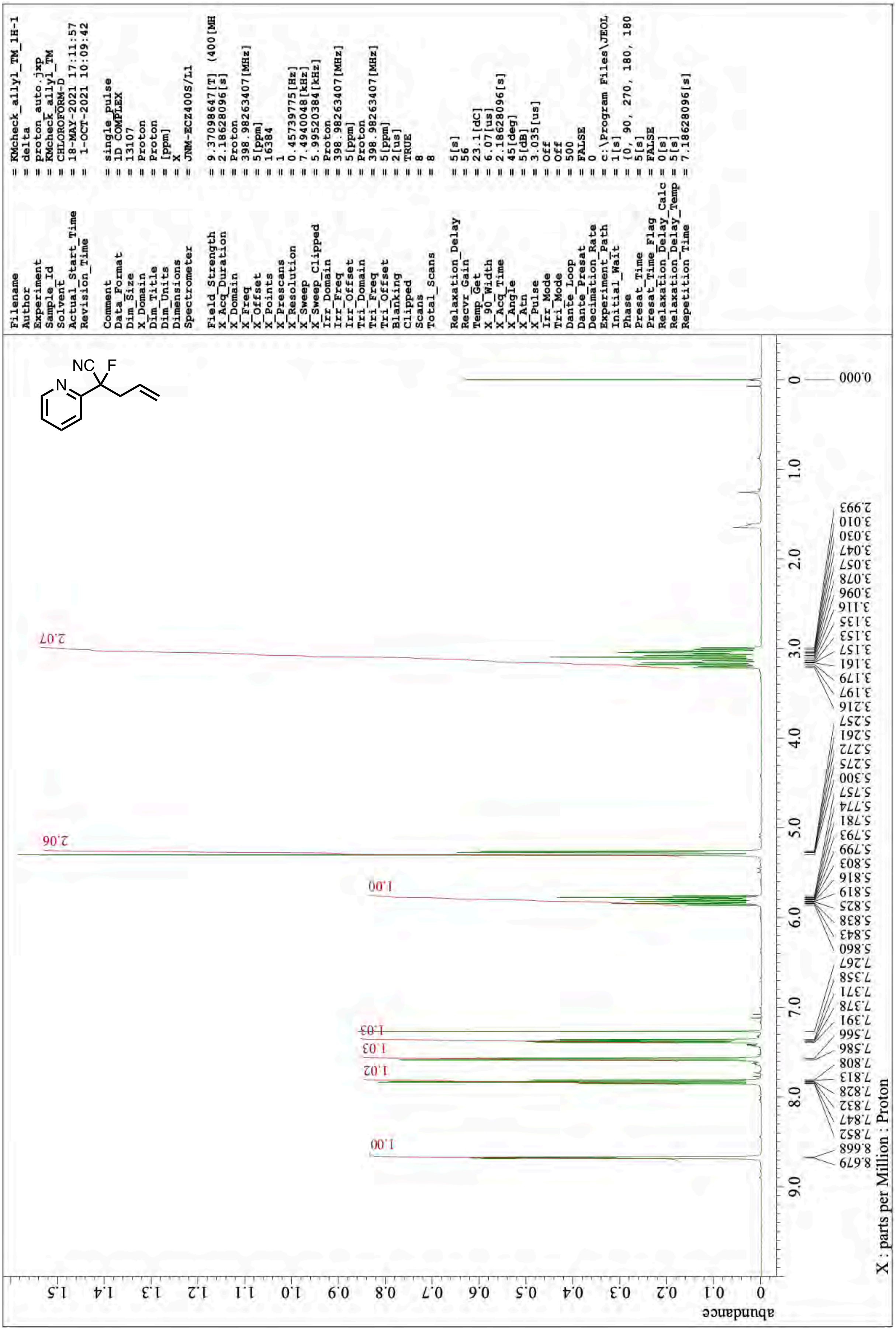

^{13}C NMR of **12** (101 MHz, $CDCl_3$)

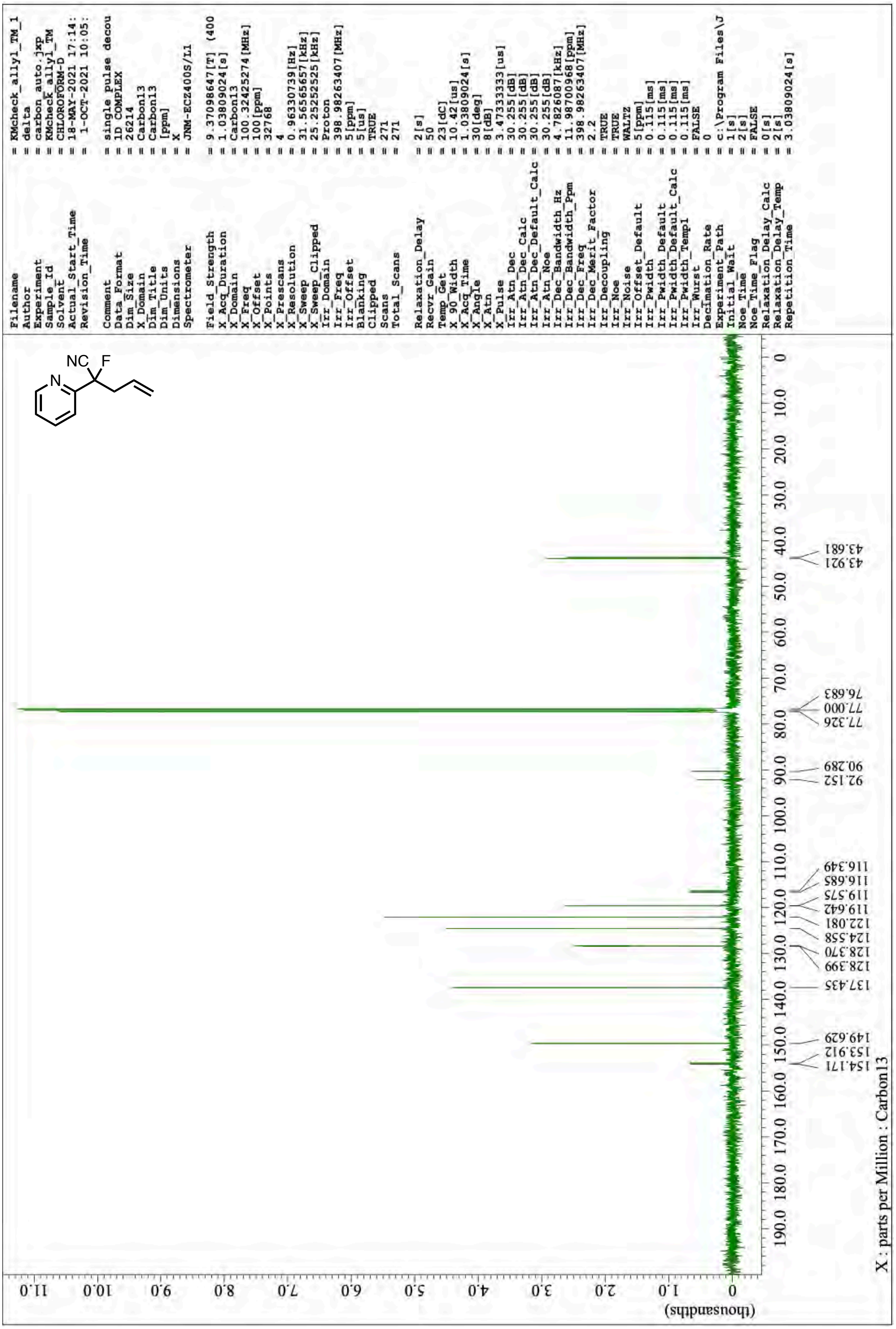

^{19}F NMR of **12** (376 MHz, $CDCl_3$)

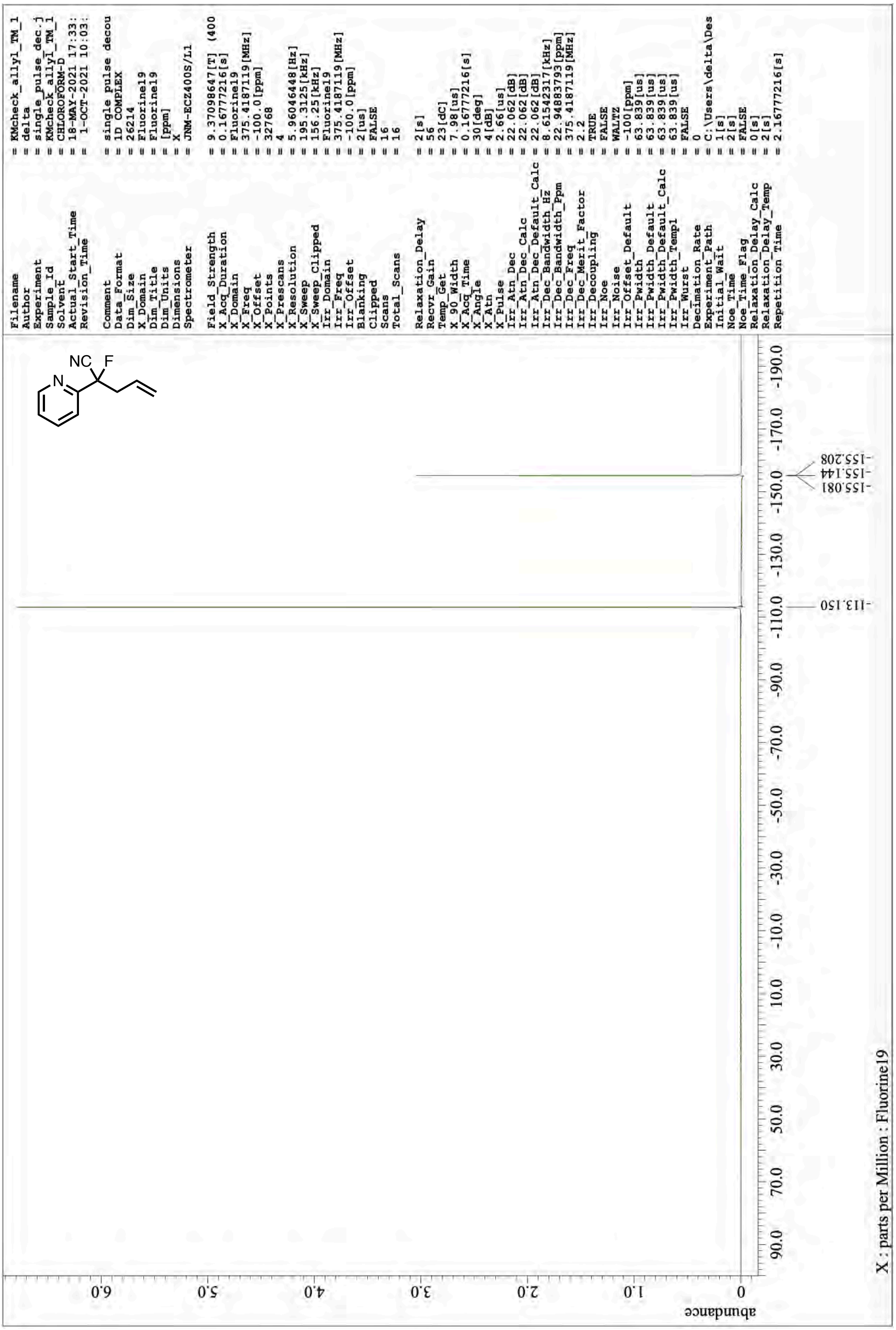

^{1}H NMR of **13** (400 MHz, $CDCl_3$)

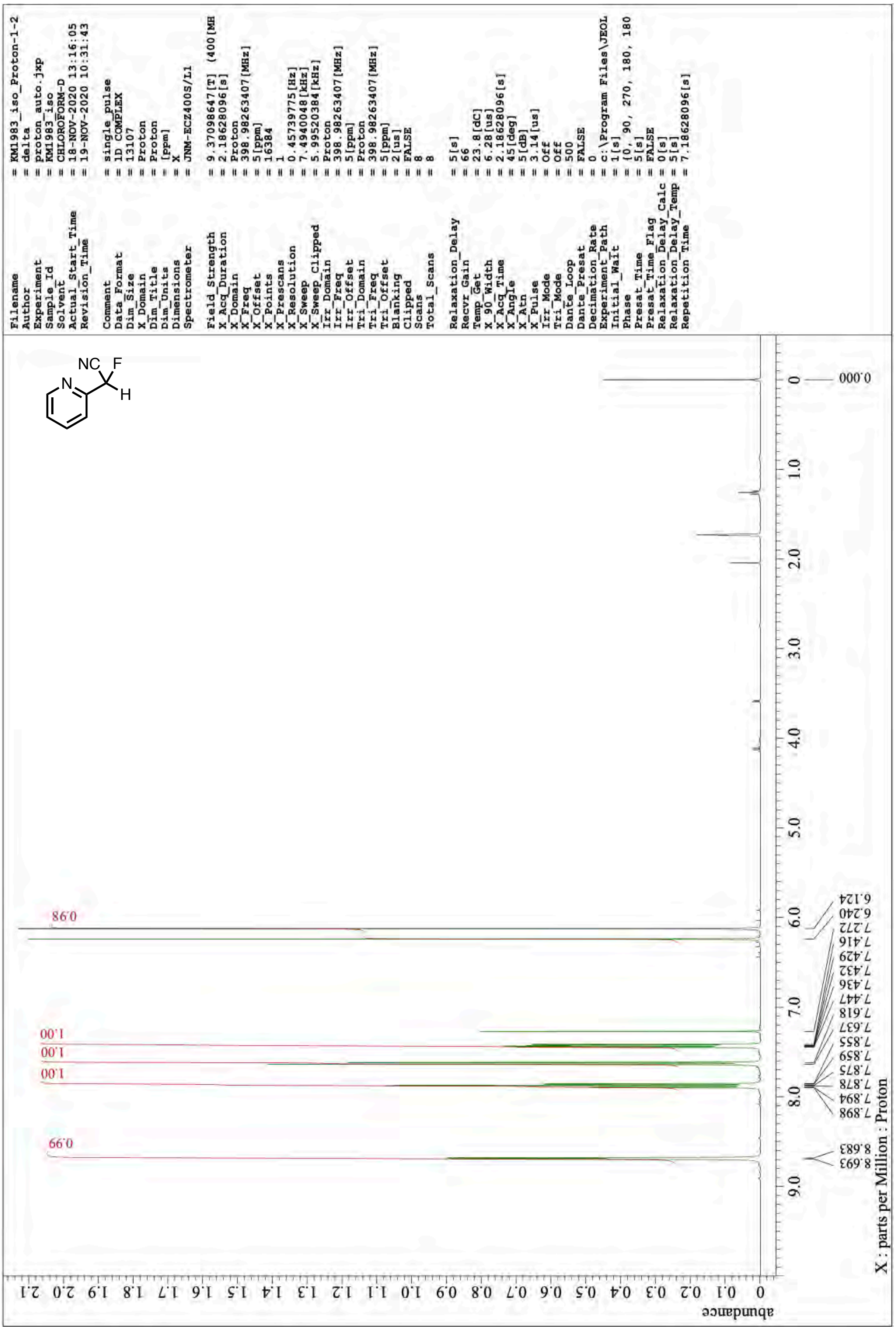

^{13}C NMR of **13** (101 MHz, $CDCl_3$)

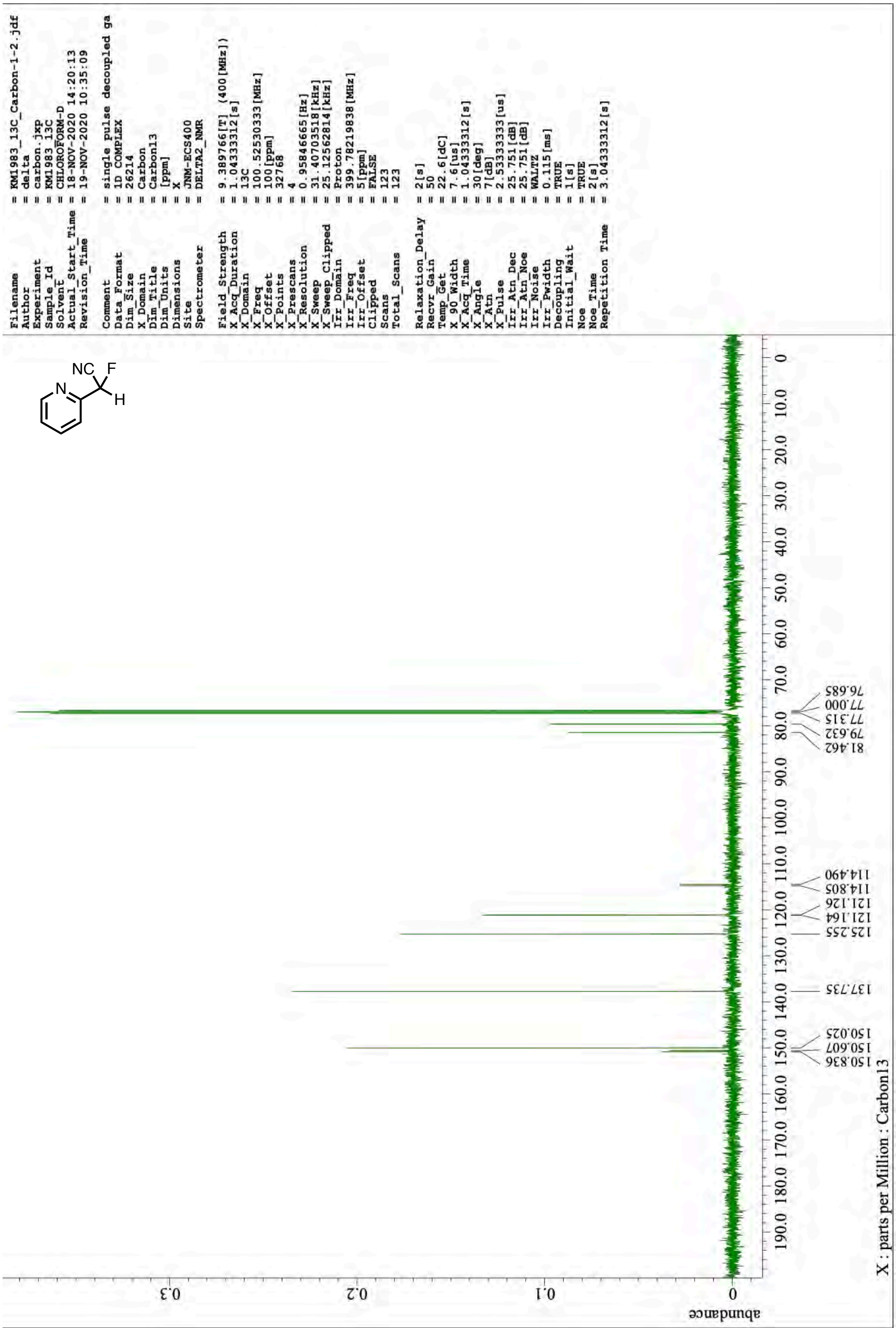

^{19}F NMR of **13** (376 MHz, $CDCl_3$)

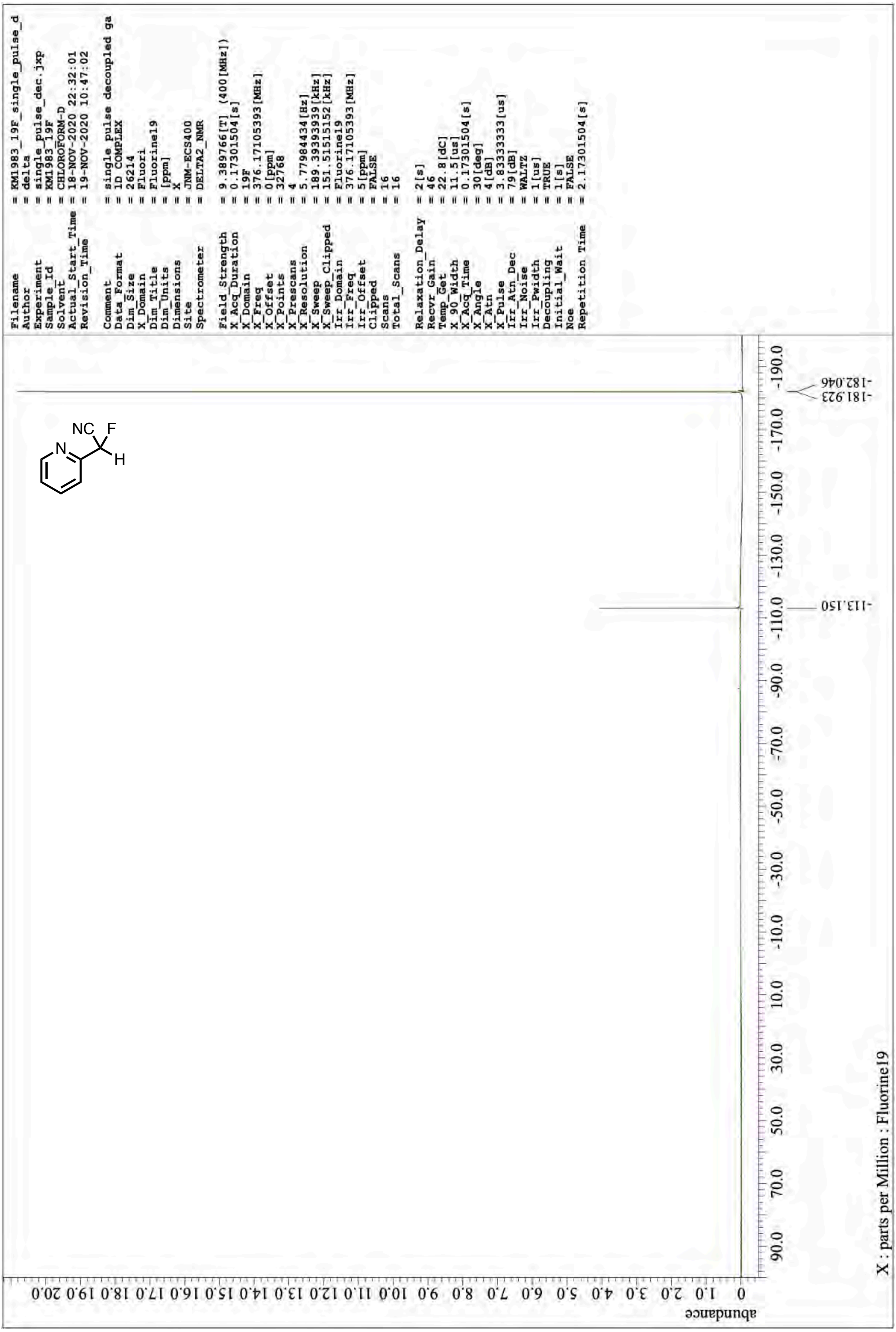

^{1}H NMR of **14** (400 MHz, $CDCl_3$)

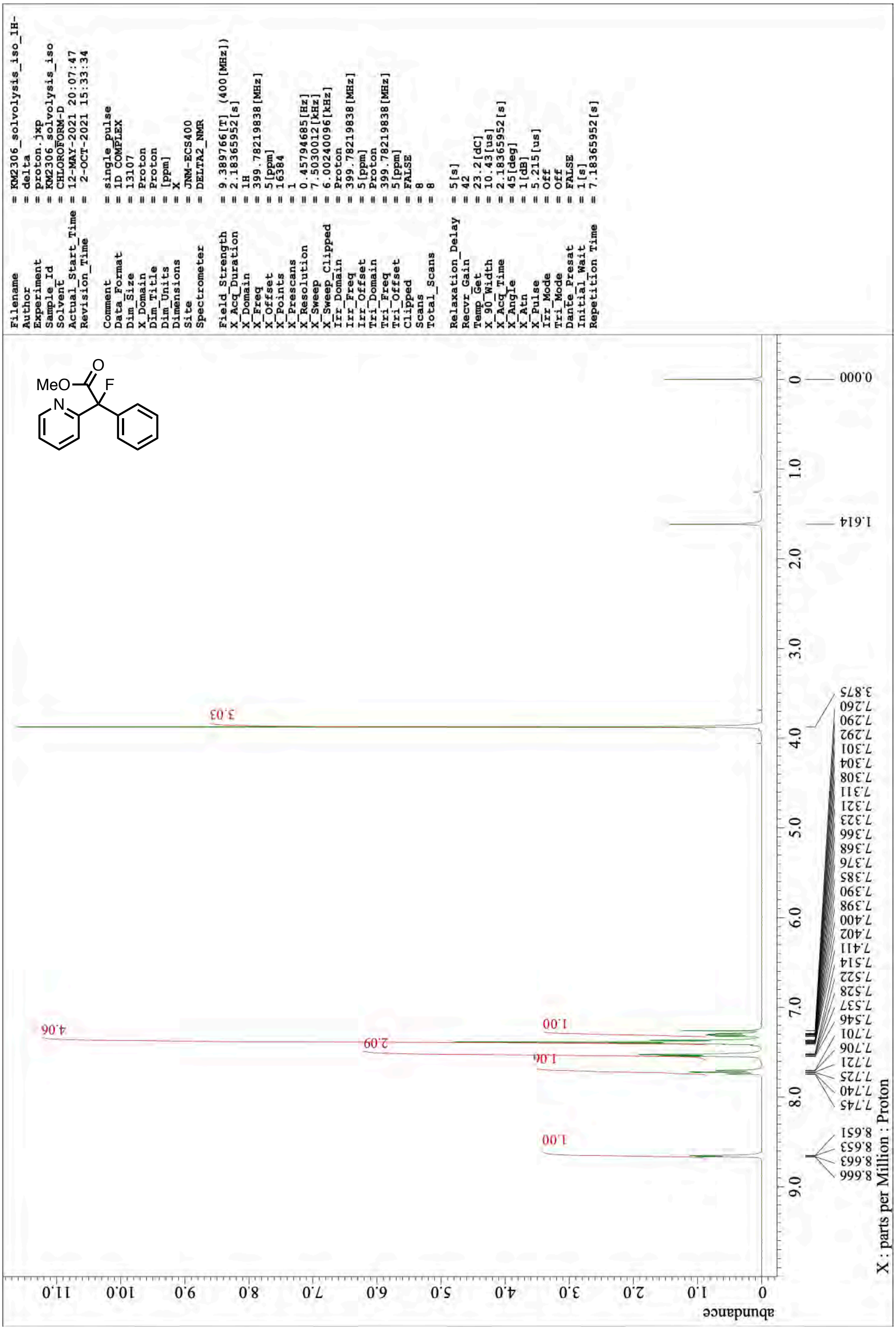

^{13}C NMR of **14** (101 MHz, $CDCl_3$)

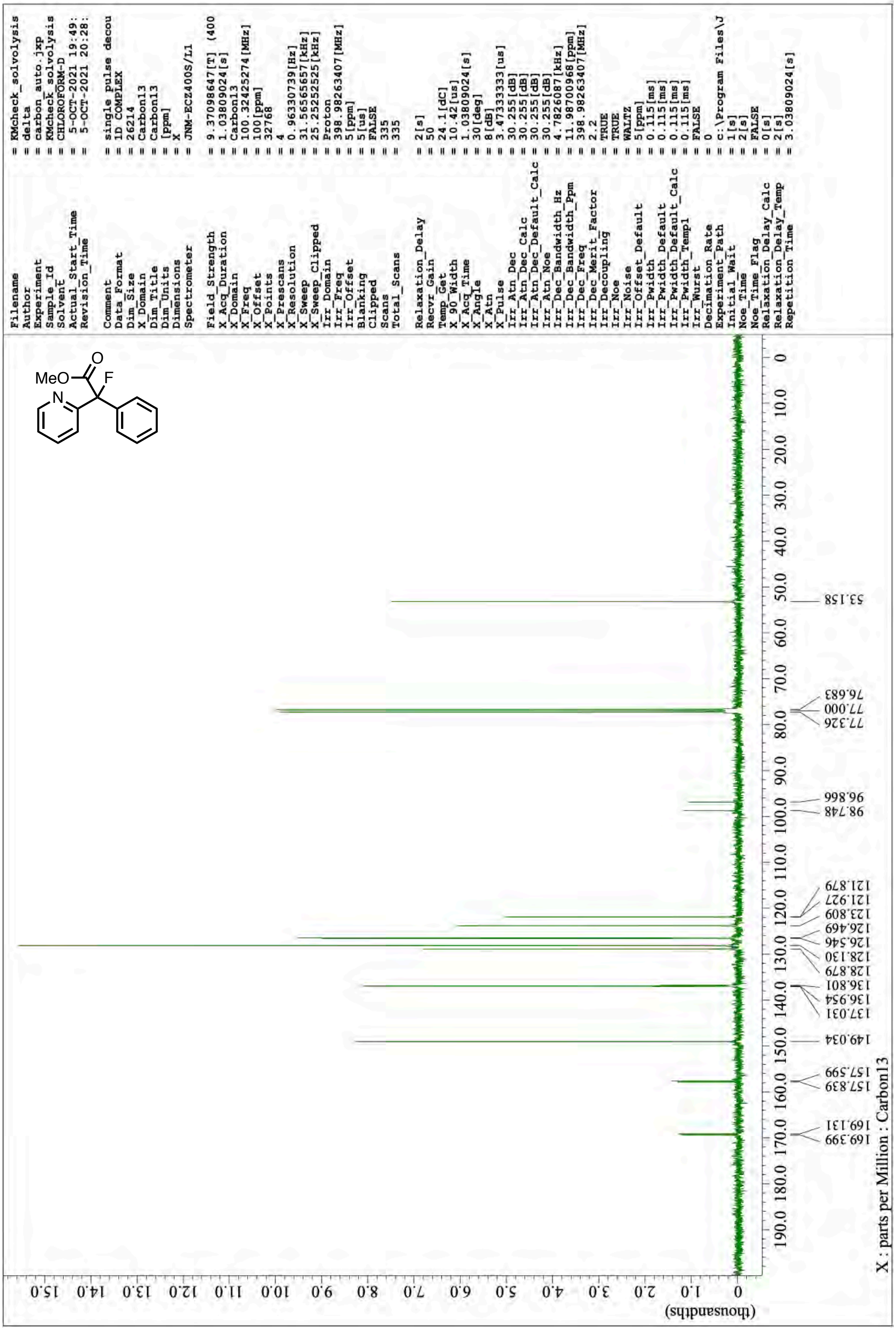

^{19}F NMR of **14** (376 MHz, $CDCl_3$)

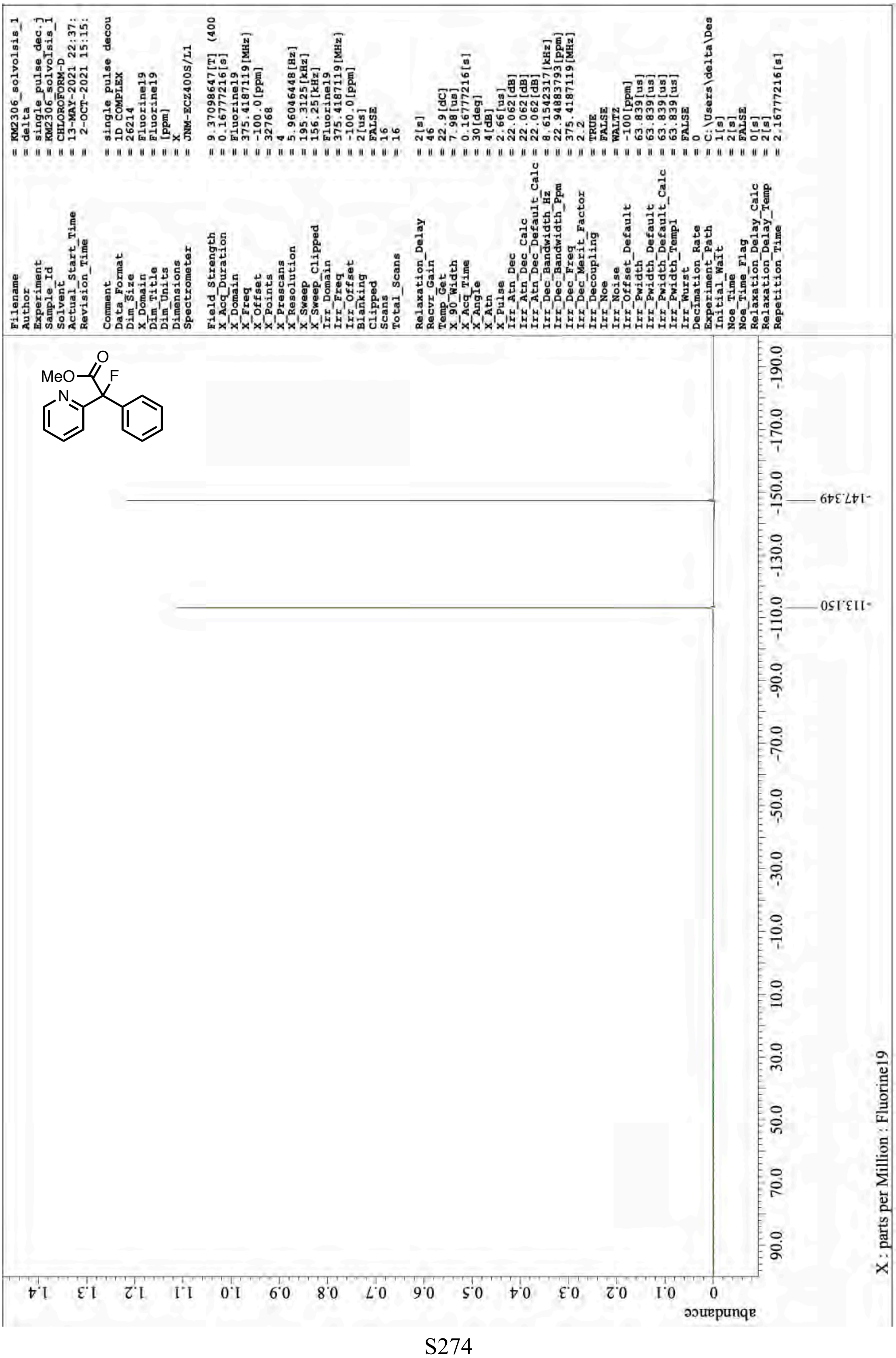

^{1}H NMR of **15** (400 MHz, $CDCl_3$)

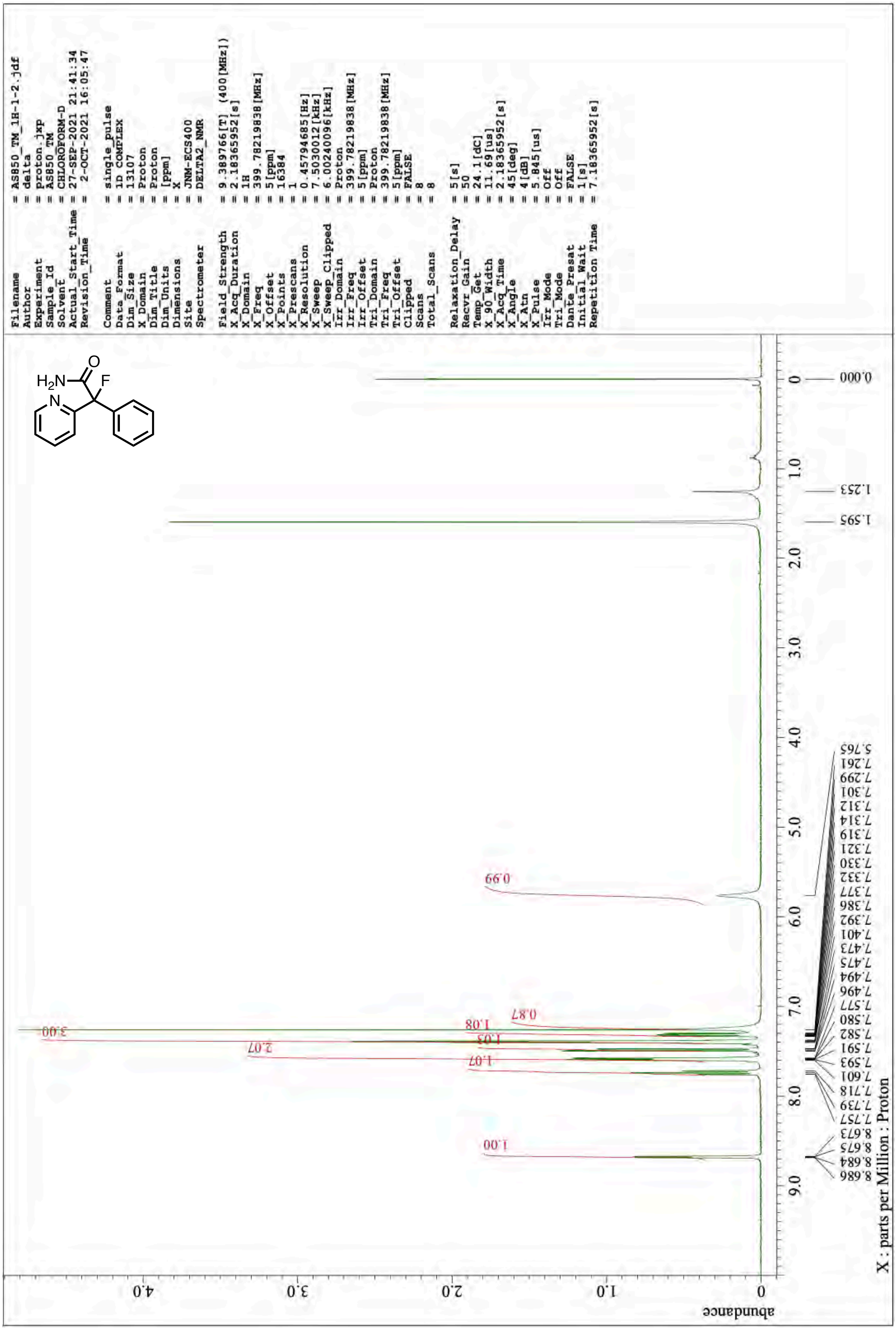

^{13}C NMR of **15** (101 MHz, $CDCl_3$)

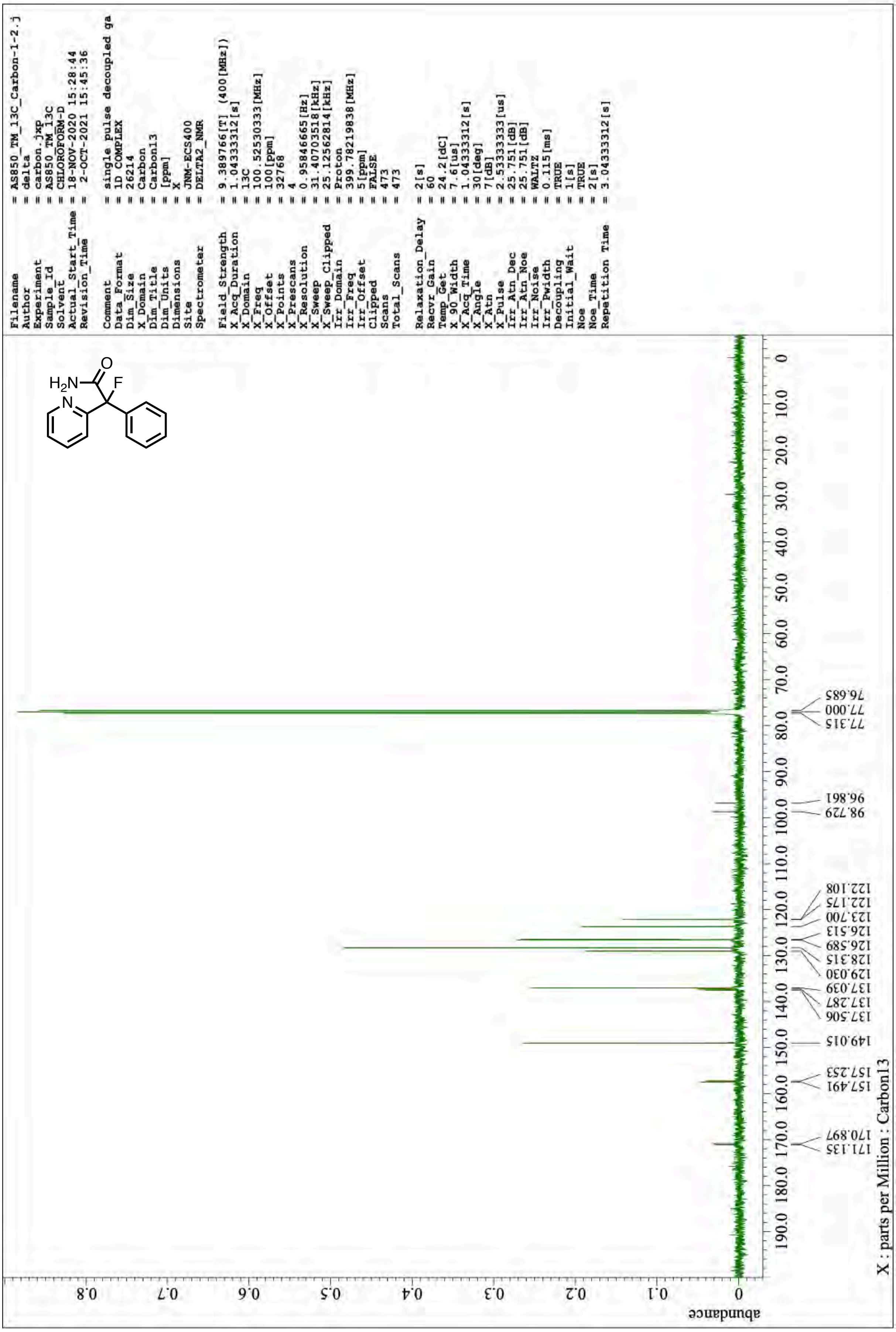

^{19}F NMR of **15** (376 MHz, $CDCl_3$)

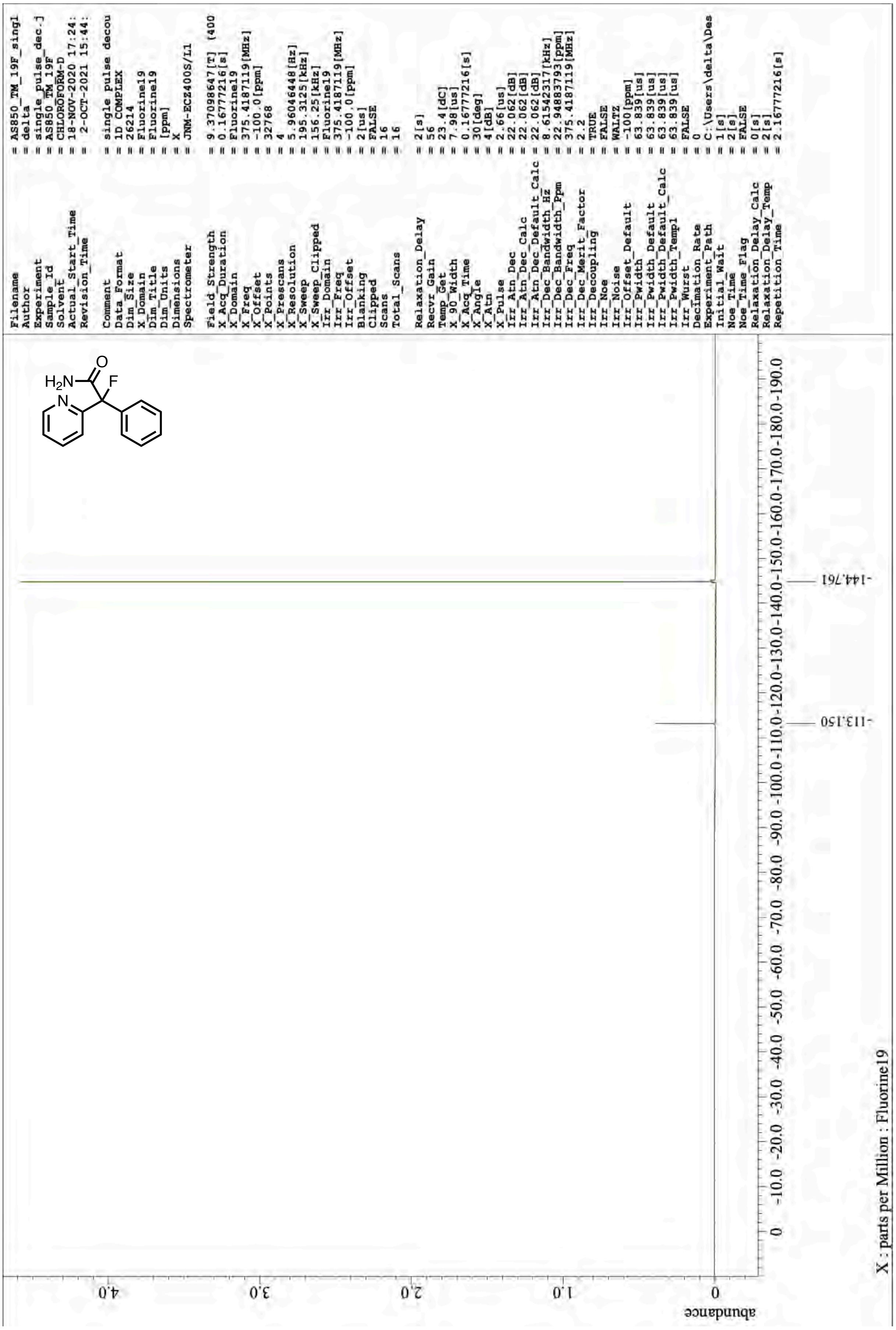

^{1}H NMR of **16** (400 MHz, $CDCl_3$)

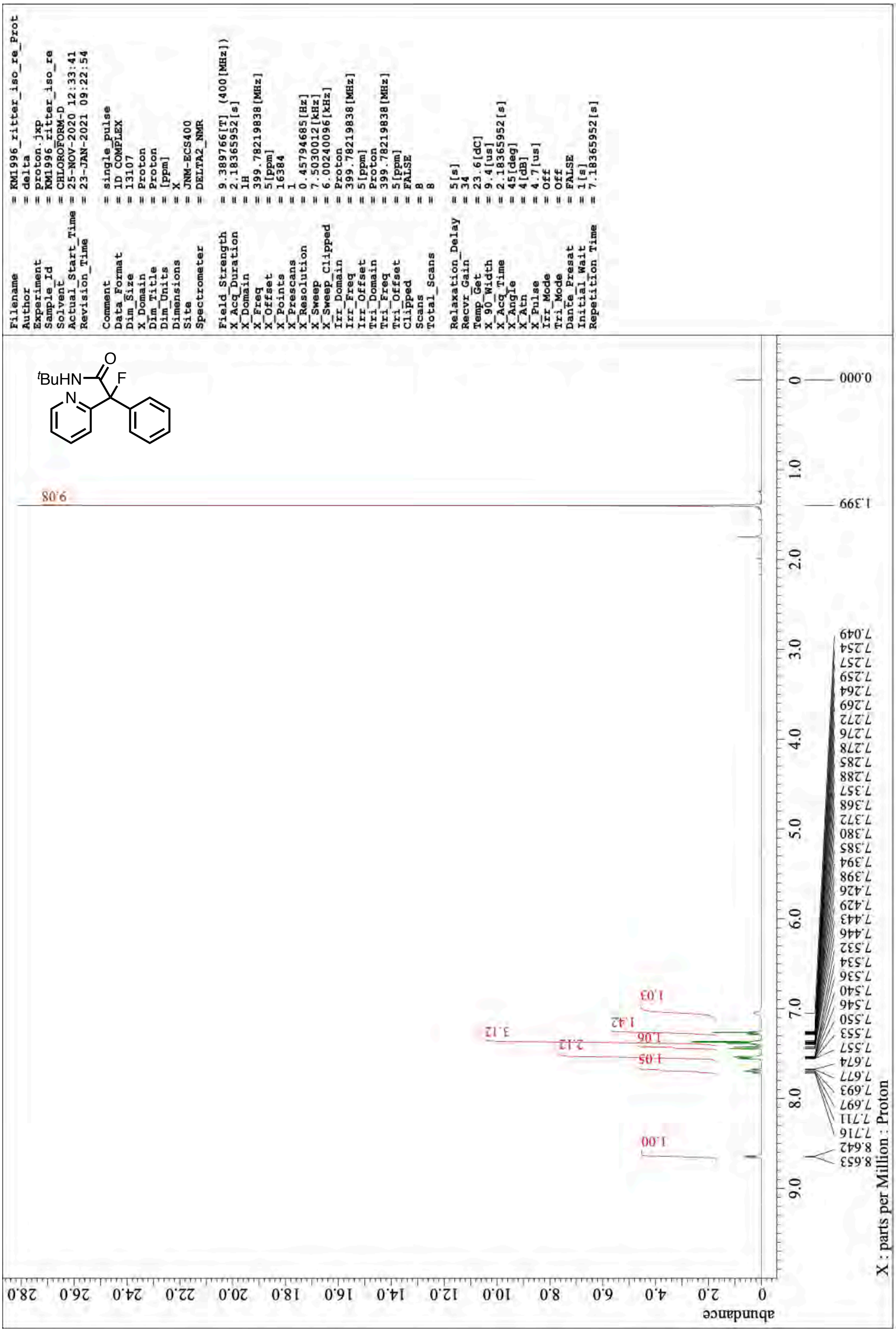

^{13}C NMR of **16** (101 MHz, $CDCl_3$)

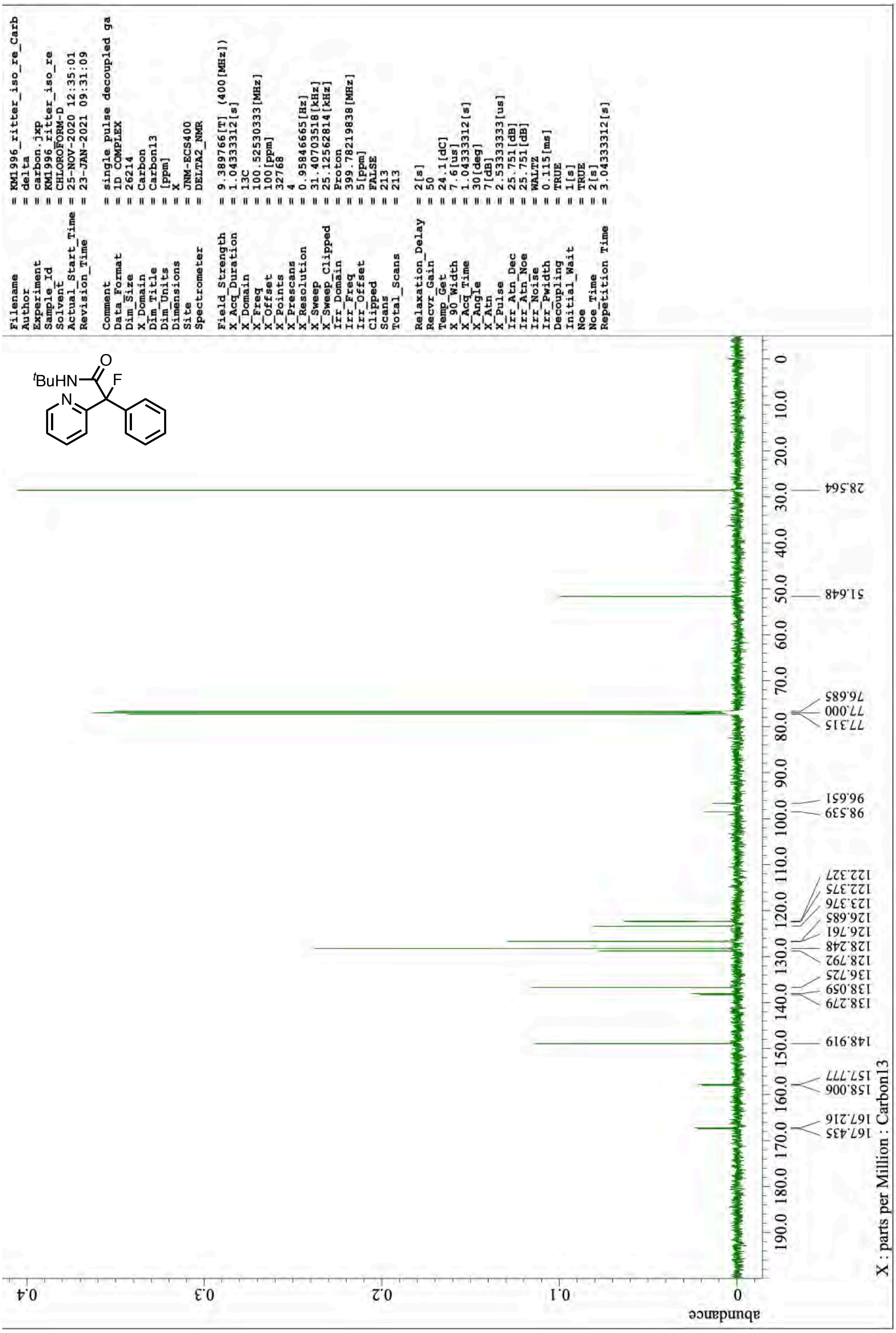

^{19}F NMR of **16** (376 MHz, $CDCl_3$)

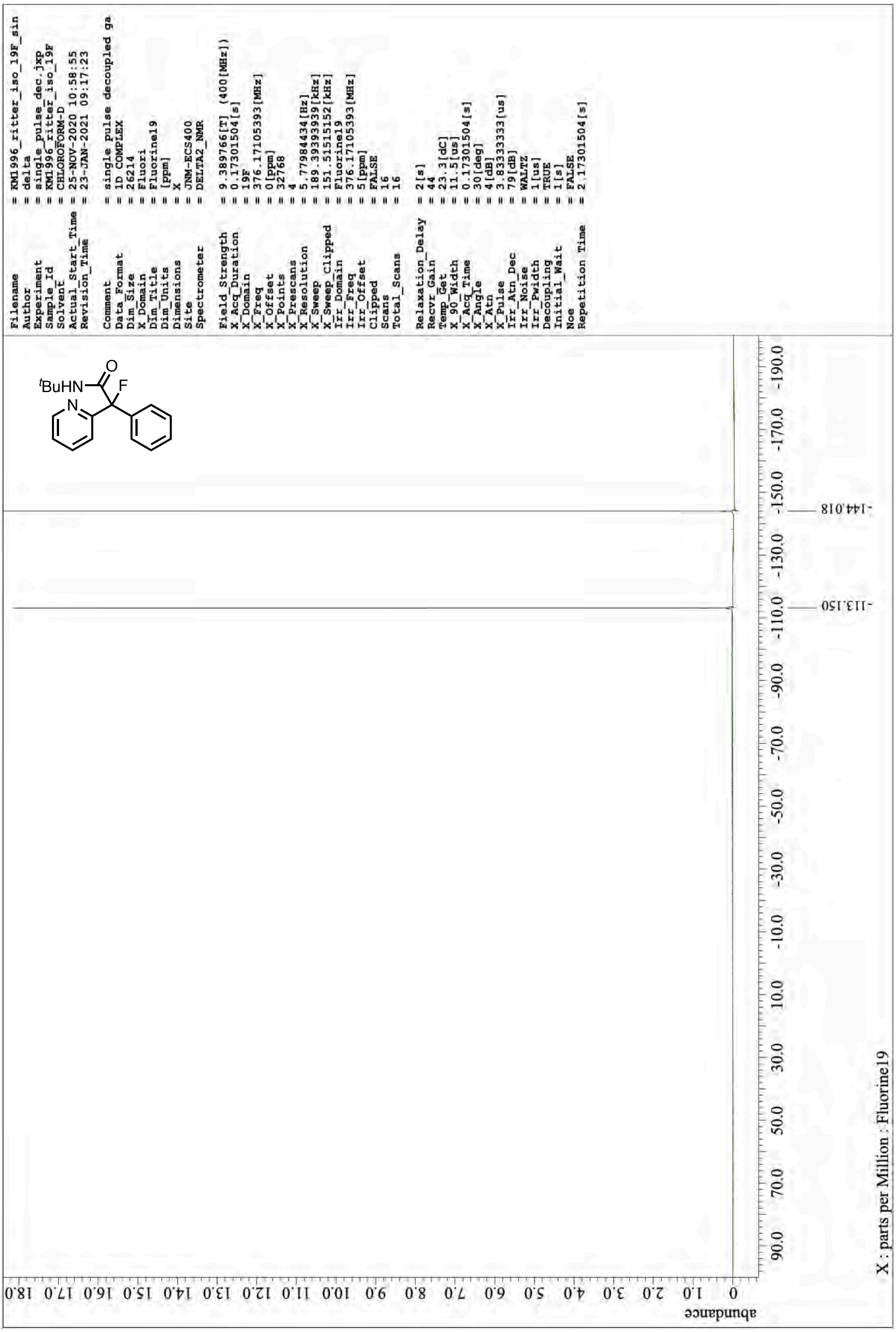

^{1}H NMR of **17** (400 MHz, $CDCl_3$)

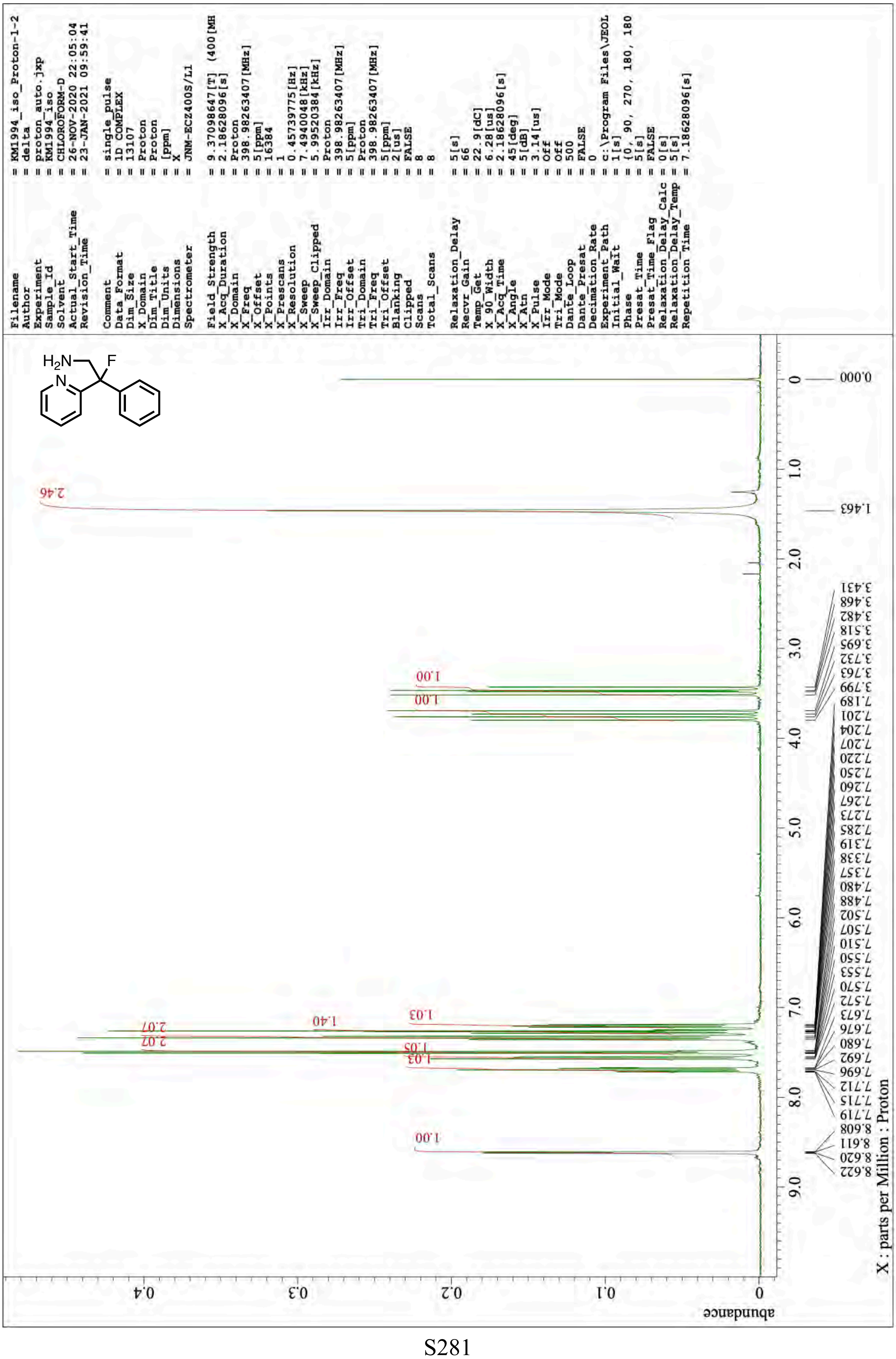

^{13}C NMR of **17** (101 MHz, $CDCl_3$)

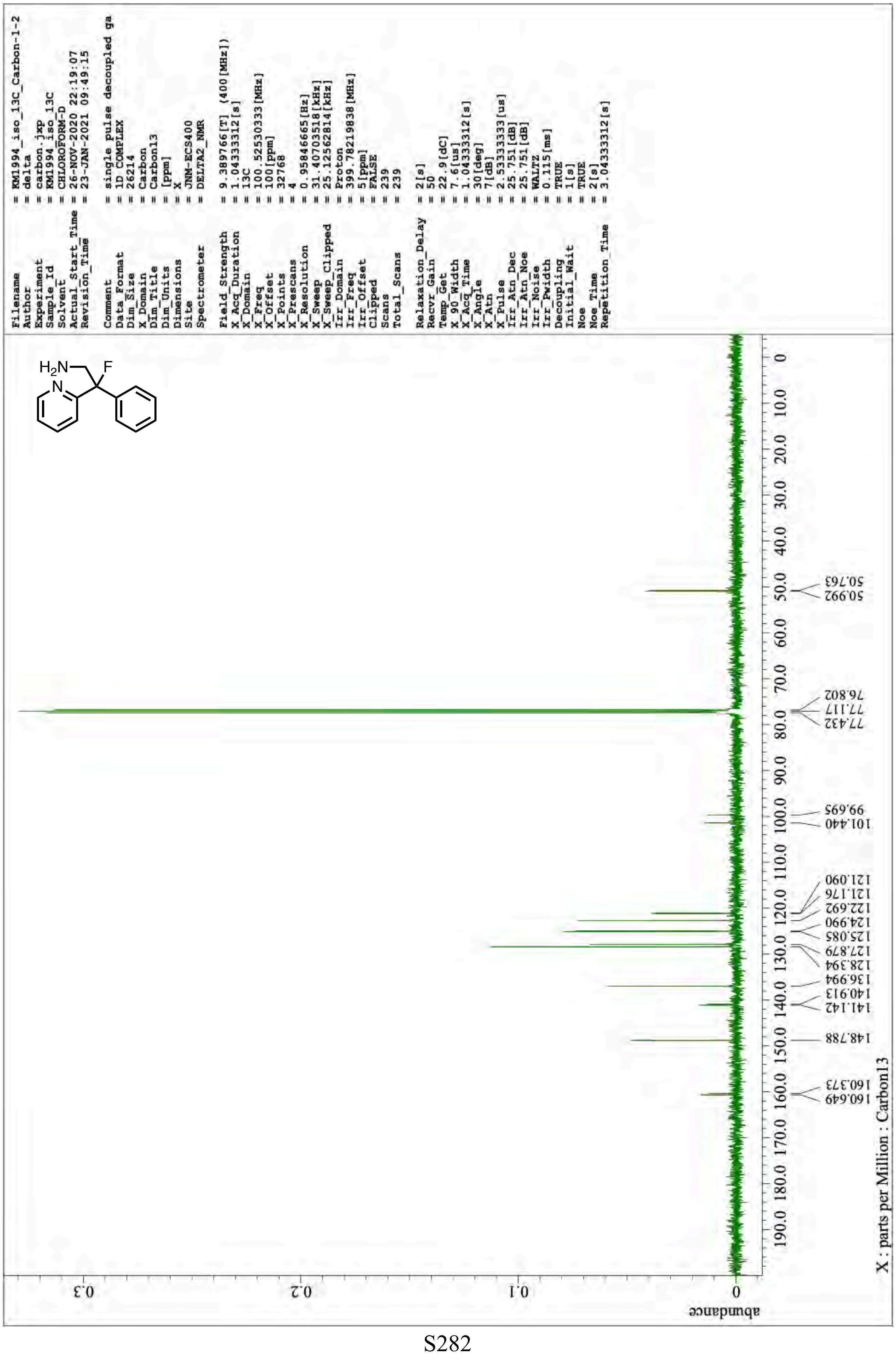

^{19}F NMR of **17** (376 MHz, $CDCl_3$)

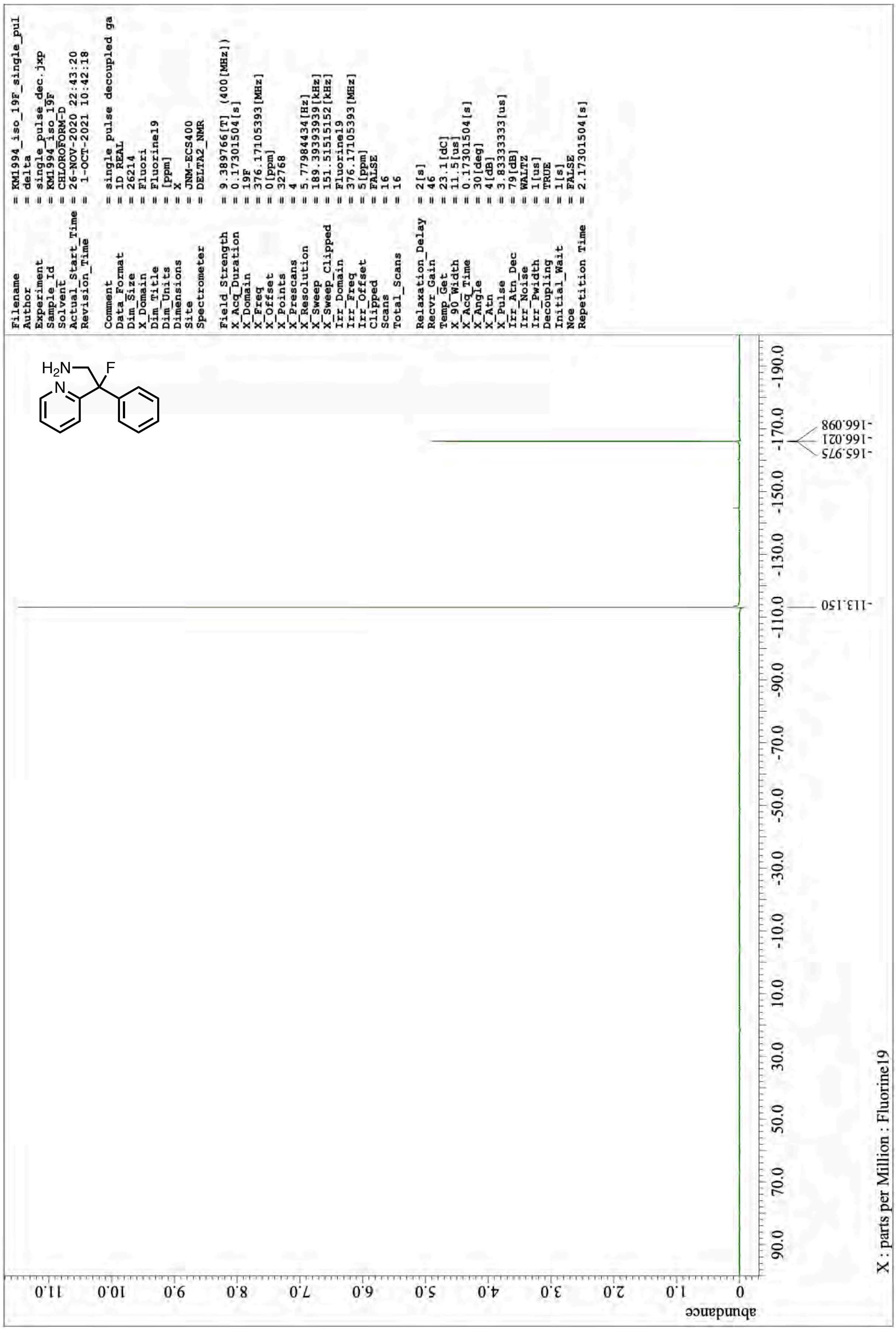

Supplement: SC-013-D1SC06273E-s001 [file SC-013-D1SC06273E-s001.pdf]
